# Seven New Lobane Diterpenoids from the Soft Coral *Lobophytum catalai*

**DOI:** 10.3390/md21040223

**Published:** 2023-03-30

**Authors:** Jiarui Zhang, Huixue Ma, Shuangshuang Jin, Xuehuan Liu, Lei Li, Zhaonan Liu, Guoqiang Li, Pinglin Li

**Affiliations:** 1 Key Laboratory of Marine Drugs, Chinese Ministry of Education, School of Medicine and Pharmacy, Ocean University of China, Qingdao 266003, China; 2 Laboratory of Marine Drugs and Biological Products, National Laboratory for Marine Science and Technology, Qingdao 266235, China; 3 Biology Institute, Qilu University of Technology (Shandong Academy of Sciences), Jinan 250103, China

**Keywords:** *Lobophytum catalai*, lobocatalens A–G, lobane diterpenoids, anti-inflammatory activity, cytotoxic activity

## Abstract

Seven new lobane diterpenoids, namely, lobocatalens A–G (**1**–**7**), were isolated from the Xisha soft coral *Lobophytum catalai*. Their structures, including their absolute configurations, were elucidated via spectroscopic analysis, comparison with the literature data, QM-MNR, and TDDFT-ECD calculations. Among them, lobocatalen A (**1**) is a new lobane diterpenoid with an unusual ether linkage between C-14 and C-18. In addition, compound **7** showed moderate anti-inflammatory activity in the zebrafish models and cytotoxic activity against the K562 human cancer cell line.

## 1. Introduction

Lobane diterpenoids are a group possessing a unique prenylated β-elemane carbon framework [1,2], which are seldom discovered in marine natural products. Since the first lobane diterpenoid, fuscol, was isolated from the Caribbean gorgonian coral in 1978 [3], no more than 20 lobane diterpenoids have been discovered in the last twenty years [4,5,6,7,8,9]. Nevertheless, these lobane-type diterpenoids show a wide range of biological activities such as antibacterial [10], cytotoxic [11], and anti-inflammatory activity [9], displaying impressive value worthy of further investigation.

**Figure 1:**
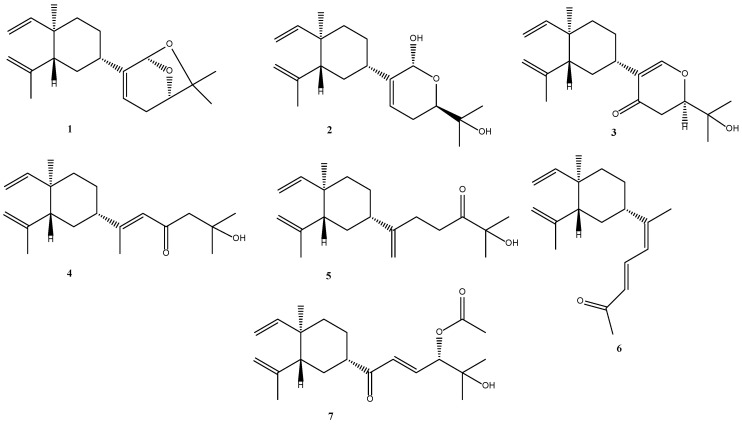
Structures of compounds **1**–**7**.

The soft corals of the genus *Lobophytum* (family Alcyoniidae) are well known as a rich source of lobane diterpenoids [4,5,6,7,8,9], cembranolides [12,13,14,15], and prenylgermacrane-type diterpenoids [16]. With the aim of seeking new bioactive lobane diterpenoids, our continuing investigation of the soft coral *Lobophytum catalai* collected from Yagong Island led to the isolation of seven new lobane diterpenoids. Considering that there have been no reports on lobane-based diterpenoids from the soft coral *Lobophytum catalai,* these new compounds, **1**–**7**, were named lobocatalens A–G (Figure 1). Among them, lobocatalen A (**1**) is a new lobane diterpenoid with an unusual ether linkage between C-14 and C-18. In addition, compound **7** showed moderate anti-inflammatory activity in the zebrafish models and moderate cytotoxic activity against the K562 human cancer cell line. Moreover, the isolation, structure elucidation, and biological activity of these isolated compounds are reported.

## 2. Results

**Figure 2:**
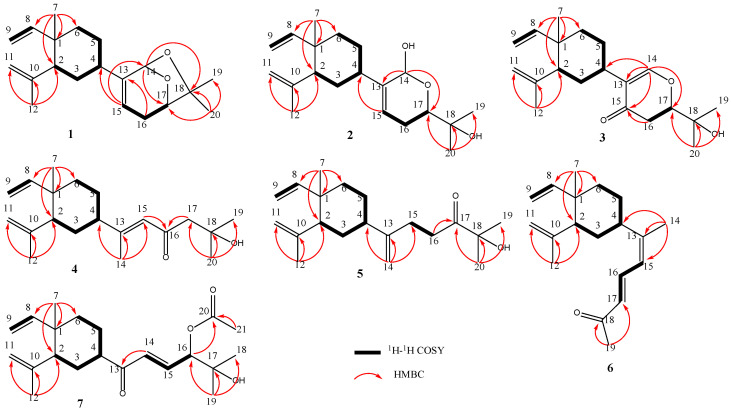
Key ^1^H–^1^H COSY and HMBC correlations for compounds **1**–**7** from *Lobophytum Catalai*.

Lobocatalen A (**1**) was isolated as a colorless oil. The HRESIMS experiment exhibited a pseudo-molecular ion peak at *m*/*z* 303.2320 [M + H]^+^, consistent with the molecular formula of C_20_H_30_O_2_, requiring six degrees of unsaturation. The clear IR absorption at 3079 and 1635 cm^−1^ together with the UV spectrum at λmax = 193 (log ε 1.65) nm indicated the presence of a double-bond group. The 1D NMR data (Table 1 and Table 2) revealed the presence of a monosubstituted terminal double bond (C-9 (*δ*_C_ 110.1, CH_2_) and C-8 (*δ*_C_ 150.3, CH); H_2_-9 (*δ*_H_ 4.91, d; *δ*_H_ 4.87, s) and H-8 (*δ*_H_ 5.80, dd)), a disubstituted terminal olefinic bond (C-11 (*δ*_C_ 112.4, CH_2_), C-10 (*δ*_C_ 147.5, CH), and C-12 (*δ*_C_ 24.9, CH_3_); H_2_-11 (*δ*_H_ 4.81, s; *δ*_H_ 4.57, s) and H_3_-12 (*δ*_H_ 1.69, s)), a ring-junction methyl (C-7 (*δ*_C_ 16.8, CH_3_); H_3_-7 (*δ*_H_ 0.99, s)), and a ring-junction methine (C-2 (*δ*_C_ 52.7, CH); H-2 (*δ*_H_ 1.99, dd)), which are the characteristic signals of the β-element segment of lobane diterpenoids [4,6,7]. This deduction was then proven by the ^1^H-^1^H COSY correlations from H-8 to H-9, and from H-2 to H-6, along with the HMBC correlations from H_3_-7 to C-1, C-2, C-6, and C-8, from H_3_-12 to C-2, C-10, and C-11, and from H_2_-15 to C-4 and C-14 (Figure 2).

The remaining two methyls (sp^3^-hybridized), methylene (sp^3^-hybridized), three methines (one olefinic and two oxygenated), and two non-protonated carbons (one oxygenated and one olefinic) are related to the side chain of lobane diterpenoids. Based on the above data, a six-membered ether ring was established based on the ^1^H-^1^H COSY correlations from H-15 to H-17, and the HMBC correlations from H_2_-16 to C-13, and from H-14 (*δ*_H_ 5.36, s) to C-17 (*δ*_C_ 80.1) (Figure 2). The above data accounted for five degrees of unsaturation; thus, the remaining degree was designated as a one-ring system. This deduction was further proven by the HMBC correlations from H-14 to C-18, and from H_3_-20 to C-17, C-18 (*δ*_C_ 81.2), and C-19, combined with the significant downfield shifts observed for C-14 (*δ*_C_ 99.2), C-17 (*δ*_C_ 80.1), and C-18 (*δ*_C_ 81.2). Thus, a new lobane diterpenoid (**1**) with an unusual ether linkage between C-14 and C-18 was established (Figure 2).

**Figure 3:**
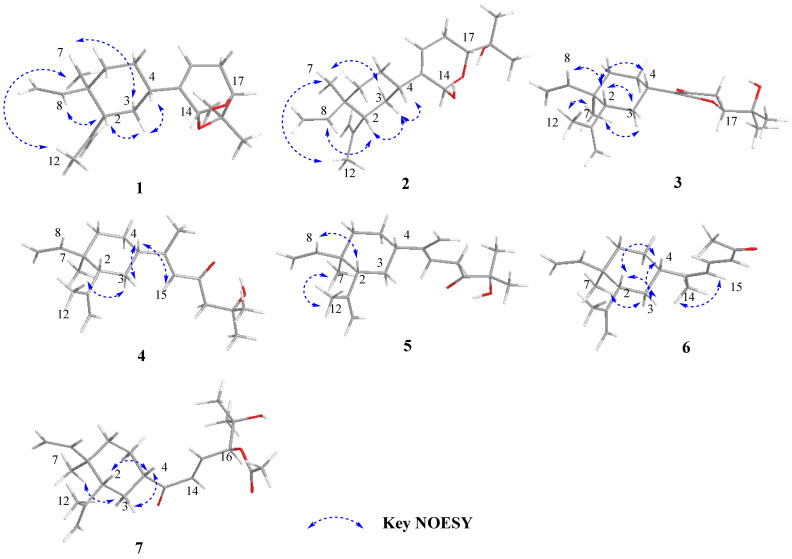
Key NOESY correlations for compounds **1**–**7**.

**Figure 4:**
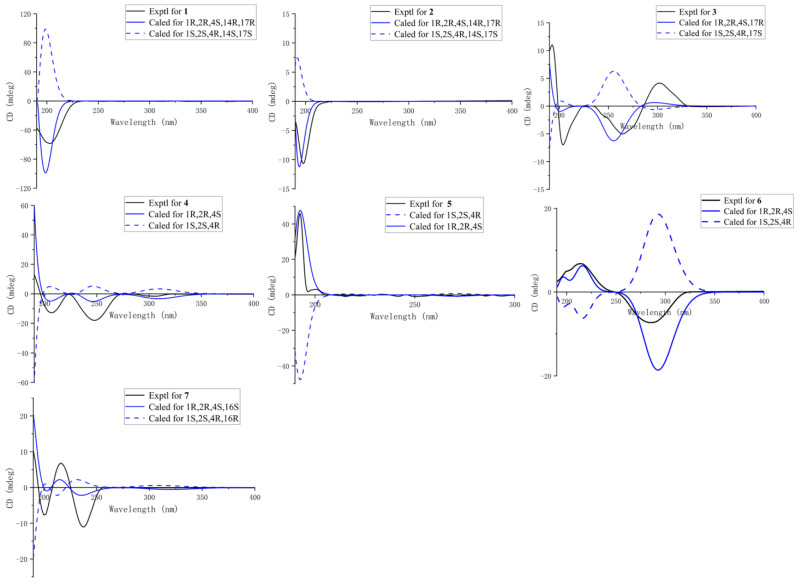
Experimental and calculated ECD spectra of compounds **1**–**7**.

In the NOESY spectrum of **1** (Figure 3), the clear correlations of H-3a (*δ*_H_ 1.57)/H-2, H-3a (*δ*_H_ 1.57)/H-4, H-3b (*δ*_H_ 1.49)/H_3_-7, H_3_-7/H_3_-12, and H-2/H-8 indicated the *β*-orientation of H-2 and H-4, and the *α*-orientation of H_3_-7. This deduction was further proven through a comparison of the chemical shifts with previously reported lobane-type diterpenoids [4,5,6,7]. The orientations of C-14 and C-17 were defined as 14R* and 17R* in the DP4^+^ calculations (Supplementary Materials, Figure S2) [17]. Finally, the absolute configurations of **1** were defined as 1*R*, 2*R*, 4*S*, 14*R,* and 17*R* in the TDDFT-ECD calculations (Figure 4).

Lobocatalen B (**2**) was obtained as a colorless oil. The molecular formula of **2** was determined to be C_20_H_32_O_3_ based on its HRESIMS ion peak at *m*/*z* 338.2685 [M + NH_4_]^+^. The IR absorption at 3080 and 1635 cm^−1^ together with the UV spectrum at λmax = 193 (log ε 0.38) nm indicated the presence of a double-bond group. Additionally, the IR absorption at 3410 cm^−1^ indicated the presence of a hydroxy group. The 1D NMR data of **2** (Table 1 and Table 2) were similar to those of lobatriene [18], a known lobane diterpenoid isolated from an Okinawan soft coral of the genus *Sinularia flexibilis.* The only difference between them is that one hydrogen atom of methylene at C-14 in lobatriene is replaced by a hydroxy group in **2**. The key HMBC correlations (Figure 2) from H-14 (*δ*_H_ 5.48) to C-13 and C-17 and the significant downfield chemical shifts of C-14 (*δ*_C_ 101.1) also supported the change in functional groups. Thus, the planar structure of **2** was constructed (Figure 2). Through a comparison with the NMR data of previously reported lobane-type diterpenoids for which the cyclohexane systems all have the same stereochemistry of 1R*, 2R*, and 4S* [4,5,6,7], the relative configurations of C-1, C-2, and C-4 of **2** were the same as those reported for lobane-type diterpenoids. The NOESY correlations (Figure 3) of H-3a (*δ*_H_ 1.60)/H-2, H-4/H-3a, H-3b (*δ*_H_ 1.52)/H_3_-7, H_3_-7/H_3_-12, and H-2/H-8 further confirmed this deduction. The optical rotation of **2** is [α]${}_{D}^{25}$ −40.1, which is similar to that of **1** ([α]${}_{D}^{25}$ −30.1). Because of the homologous structures, the similar optical rotation data may imply the same relative configuration. The relative configurations of all the asymmetric centers in **2** were established in the DP4^+^ calculations (Supplementary Materials, Figure S3). The absolute configurations were further determined as 1*R*, 2*R*, 4*S*, 14*R*, and 17*R* in the TDDFT-ECD calculations (Figure 4).

Lobocatalen C (**3**), a colorless oil, possesses a molecular formula of C_20_H_30_O_3_ on the basis of its HREIMS ion peak at *m*/*z* 319.2265 [M + H]^+^, requiring six degrees of unsaturation. The IR absorption at 1664 cm^−1^ together with the UV spectrum at λmax = 273 (log ε 0.12) nm indicated the presence of an α, β-unsaturated carbonyl group. The 1D NMR data (Table 1 and Table 2) of compound **3** resembled those of lobatrienolide [19], a known lobane diterpenoid isolated from an Okinawan soft coral of the genus *Sinularia flexibilis*. In fact, compound **3** has the same functional groups as lobatrienolide, except for the migration of the double bonds at C-13 and C-15 in lobatrienolide to C-13 and C-14 in **3**, and that of the carbonyl group at the C-14 position in lobatrienolide to C-15 in **3**. This deduction was proven by the key HMBC correlations (Figure 2) from H-14 to C-4, C-13, C-15 (*δ*_C_ 192.3), and C-17 (*δ*_C_ 84.8), and from H-16 to C-15. The 1R*, 2R*, and 4S* configurations of the β-elemene ring system, which were the same as those of the co-isolates, were determined based on the NOESY correlations (Figure 3) of H-2/H-4, H-2/H-3a (*δ*_H_ 1.61), H-7/H-3b (*δ*_H_ 1.50), H_3_-7/H_3_-12 (*δ*_H_ 1.69), and H-2/H-8, along with the similar chemical shifts of the β-elemene ring system compared with the co-isolates. The relative configuration of C-17 was deduced as R* in the DP4^+^ calculations (Supplementary Materials, Figure S4). Finally, the absolute configurations of **3** were defined as 1*R*, 2*R*, 4*S*, and 17*R* in the TDDFT-ECD calculations (Figure 4).

Lobocatalen D (**4**) was isolated as a colorless oil. Its molecular formula, C_20_H_32_O_2_, was established based on its HRESIMS ion peak at *m*/*z* 305.2475 [M + H]^+^. The IR absorption at 1631 cm^−1^ together with the UV spectrum at λmax = 271 (log ε 0.53) nm indicated the presence of an α, β-unsaturated carbonyl group. The 1D NMR data (Table 1 and Table 2) of **4** resembled those of loba-8,10,13(15)-triene-17,18-diol [20], a known lobane diterpenoid isolated from a soft coral of the genus *Lobophytum*. In fact, the structure of **4** is truly similar to that of loba-8,10,13(15)-triene-17,18-diol. The difference between them is that the 17-OH in loba-8,10,13(15)-triene-17,18-diol is replaced by one hydrogen atom of methylene in **4**, and there is a carbonyl group at C-16 in **4** instead of a methylene as in the known compound. Based on the HMBC correlations from H_3_-14 to C-4, C-13, and C-15, from H-15 to C-16 (*δ*_C_ 203.0), from H-17 to C-16, and from H_3_-20 to C-17 (*δ*_C_ 54.4), C-18 (*δ*_C_ 70.1), and C-19, this deduction was proven (Figure 2).

The relative configurations of **4** were determined through an analysis of its NOESY spectrum (Figure 3). The NOESY correlations of H-3a (*δ*_H_ 1.52)/H-4 and H_3_-7/H-3b *(δ*_H_ 1.64) indicated the *β*-orientation of H-4, and the *α*-orientation of H_3_-7. The *E* geometry of the Δ^13^ double bonds was established based on the NOESY correlations of H-15/H-4. Through a comparison of the NMR data with those of previously reported (-)-β-elemene-type diterpenoids and the co-isolates, the orientation of H-2 was found to be *β.* Then, the 1R*, 2R*, and 4S* configurations of the β-elemene ring system of **4** were further proven in the DP4^+^ calculations (Supplementary Materials, Figure S5). Finally, the absolute configurations of **4** were unambiguously determined in the TDDFT-ECD calculations as 1*R*, 2*R*, and 4*S* (Figure 4).

Lobocatalen E (**5**) has a molecular formula of C_20_H_32_O_2_, as determined by its HRESIMS ion peak at *m*/*z* 305.2469 [M + H]^+^, suggesting five degrees of unsaturation. The IR absorption at 1711 cm^−1^ indicated the presence of a carbonyl group. Analysis of the ^1^H and ^13^C NMR spectra (Table 1 and Table 2) indicated that **5** has a similar functional group to lobovarol G [4], a known lobane diterpenoid isolated from the soft coral *Lobophytum varium.* The only difference is that the 17-OH in lobovarol G is oxidized to a ketonic group in **5**. Furthermore, the HMBC correlations from H-16 to C-17 (*δ*_C_ 214.1) and from H_3_-20 to C-17, C-18 (*δ*_C_ 76.4), and C-19 and the ^1^H-^1^H COSY correlations from H-15 to H-16 confirmed this variation in the functional groups (Figure 2). The NOESY correlations (Figure 3) of H_3_-7/H-12 (*δ*_H_ 1.71) and H-2/H-8, along with the similar NMR data compared with the co-isolates, indicated the 1R* and 2R* configurations of the β-elemene ring system. Then, the ^13^C NMR chemical shift calculation in the DP4^+^ calculations clearly indicated that the relative configuration of C-4 is S*. Hence, the absolute configurations were confirmed as 1*R*, 2*R*, and 4*S* (Supplementary Materials, Figure S6) in the following TDDFT-ECD calculations.

Lobocatalen F (**6**) was isolated as a colorless oil. The HRESIMS ion peak at *m*/*z* 273.2213 [M + H]^+^ of **6** indicated that its molecular formula is C_19_H_28_O, requiring six degrees of unsaturation. The IR absorption at 1706 cm^−1^ together with the UV spectrum at λmax = 287 (log ε 0.18) nm indicated the presence of an α, β-unsaturated carbonyl group. A survey of the literature revealed that the 1D NMR data (Table 1 and Table 2) of compound **6** were similar to those of 3, 5-heptadien-2-one [21], a known lobane diterpenoid isolated from the soft coral *Lobophytum microlobulatum* from Havellock Island. The 2D NMR of **6** revealed that the planar structure of **6** is identical to that of 3, 5-heptadien-2-one, which suggested that **6** is a stereoisomer of 3, 5-heptadien-2-one. The same relative configurations of the β-elemene ring system were established in the NOESY experiment (Figure 3) of H-7/H-3a (*δ*_H_ 1.78), H-2/H-3b (*δ*_H_ 1.33), H-4/H-3b (*δ*_H_ 1.33), and H-2/H-4. The geometry of the Δ^16^ double bonds was designated as an *E*-configuration based on the large coupling constants (*J*_16,17_ = 15.4 Hz). The NOESY correlations of H-15/H_3_-14, along with the downfield chemical shift of C-19 (*δ*_C_ 21.1) [22], designated the *Z*-configuration of the Δ^13^ double bonds, revealing the only difference between 3, 5-heptadien-2-one and **6**. Then, the 1R*, 2R*, and 4S* configurations of the β-elemene ring system of **6** were further proven in the DP4^+^ calculations (Supplementary Materials, Figure S7). Finally, the absolute configurations of **6** were unambiguously determined as 1*R*, 2*R*, and 4*S* in the TDDFT-ECD calculations (Figure 4).

Lobocatalen G (**7**), a colorless oil, possesses the molecular formula C_21_H_32_O_4_, which was established based on the HRESIMS ion peak at *m*/*z* 371.2192 [M + Na]^+^. The IR absorption at 1700 cm^−1^ together with the UV spectrum at λmax = 270 (log ε 0.03) nm indicated the presence of an α, β-unsaturated carbonyl group. The 1D NMR data (Table 1 and Table 2) of **7** closely resembled those of (1R*,2R*,4S*,15*E*)-loba-8,10, 13(14),15(16)-tetraen-17,18-diol-17-acetate, a known lobane diterpenoid isolated from the Bowden Reef soft coral *Sinularia* sp. [8]. In fact, the structure of **7** is truly similar to that of the known compound, with the exception that the 1,1-disubstituted double bond at C-13 in the known compound is replaced by a carbonyl group in **7**. This deduction was proven by the ^1^H-^1^H COSY correlations from H-14 to H-16 and the HMBC correlations from H-14 to C-13 (*δ*_C_ 202.0) (Figure 3). Due to the absent HMBC correlations, the connection between C-4 and C-13 was established based on the molecular degrees of unsaturation.

In the NOESY experiment (Figure 3), the correlations of H-2/H-4, H-7/H-3a (*δ*_H_ 1.60), and H-4/H-3b (*δ*_H_ 1.72) established the 1R*, 2R*, and 4S* configurations of the β-elemene ring system. Moreover, the large coupling constants (*J*_14,15_ = 18.0 Hz) established the *E* geometry of the Δ^14^ double bonds. In the relative configuration of C-16 in **7**, the chiral center far away from the β-elemene ring system was difficult to assign in the NOESY experiment. Then, through the ^13^C NMR chemical shift calculation in the DP4^+^ calculations (Supplementary Materials, Figure S8), the relative configuration of C-16 in **7** was deduced as 16S*. Finally, the absolute configurations of **7** were defined as 1*R*, 2*R*, 4*S*, and 16*S* in the TDDFT-ECD calculations (Figure 4).

**Figure 5:**
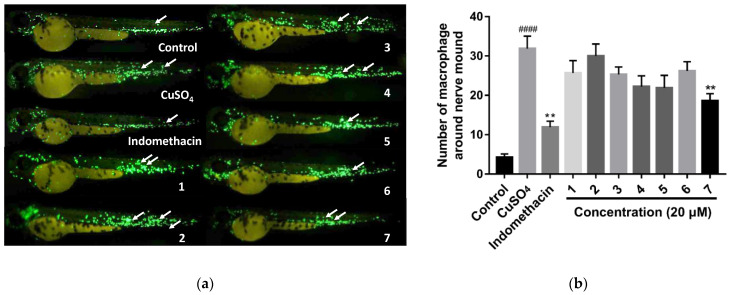
Anti-inflammatory assays of compounds **1**–**7**. (**a**) Images of inflammatory sites in CuSO_4_-induced transgenic fluorescent zebrafish (Tg: zlyz-EGFP) expressing enhanced green fluorescent protein (EGFP) treated with compounds **1**–**7**, using indomethacin as a positive control. (**b**) Quantitative analysis of macrophages in the region of inflammatory sites in zebrafish treated with compounds **1**–**7** at 20 μM. #### indicates that the CuSO_4_ model group has a very significant difference compared with the control group (*p* < 0.01). ** indicates that the sample groups have significant differences compared with the CuSO_4_ model group (*p* < 0.01).

On account of the fact that research on the anti-inflammatory activity of lobane diterpenoids in zebrafish models has not been reported up to now, we attempted to identify lobane diterpenoids with anti-inflammatory activity in zebrafish models. The anti-inflammatory effect of compounds **1**–**7** was assessed in CuSO_4_-induced transgenic fluorescent zebrafish. CuSO_4_ can produce an intense acute inflammatory response in the neuromasts and mechanosensorial cells in the lateral line of zebrafish, stimulating the infiltration of macrophages [22,23]. Thus, the number of macrophages surrounding the neuromasts in zebrafish can be observed and counted under a fluorescence microscope. Therefore, the anti-inflammatory activity of these lobane diterpenoids can be evaluated. As shown in Figure 5, compound **7** showed moderate anti-inflammatory activity by alleviating migration and decreasing the number of macrophages surrounding the neuromasts in CuSO_4_-induced transgenic fluorescent zebrafish. Meanwhile, the other compounds showed no anti-inflammatory activity.

Moreover, the cytotoxic activity of these isolated compounds (**1**–**7**) was evaluated against the human leukemia K562, normal human hepatocyte L-02, human pancreatic cancer ASPC-1, and human breast cancer MDA-MB-231 cell lines. The results (Table 3) demonstrated that compound **7** exhibited modest cytotoxicity against the K562 cell line, with an IC_50_ value of 27.96 μM.

## 3. Materials and Methods

### 3.1. General Experimental Procedures

Optical rotations were measured using a Jasco P-1020 digital polarimeter (Jasco, Tokyo, Japan). The UV spectra were recorded using a Beckman DU640 spectrophotometer (Beckman Ltd., Shanghai, China). The CD spectra were obtained using a Jasco J-810 spectropolarimeter (Jasco, Tokyo, Japan). The NMR spectra were measured using Agilent 500 MHz (Agilent, Beijing, China) and JEOL JNMECP 600 spectrometers (JEOL, Beijing, China). The 7.26 ppm and 77.16 ppm resonances of CDCl_3_ were used as internal references for the ^1^H and ^13^C NMR spectra, respectively. The HRESIMS spectra were measured using Micromass Q-Tof Ultima GLOBAL GAA076LC mass spectrometers (Autospec-Ultima-TOF, Waters, Shanghai, China). Semi-preparative HPLC was performed using a Waters 1525 pump (Waters, Singapore) equipped with a 2998 photodiode array detector and a YMC C18 column (YMC, 10 × 250 mm, 5 μm). Silica gel (200–300 mesh, 300–400 mesh, and silica gel H, Qingdao Marine Chemical Factory, Qingdao, China) was used for column chromatography.

### 3.2. Animal Material

The soft coral *Lobophytum catalai* was collected from Xisha Island (YaGong Island) in the South China Sea in 2018 and frozen immediately after collection. The specimen was identified by Ping-Jyun Sung, at the Institute of Marine Biotechnology, the National Museum of Marine Biology and Aquarium, Pingtung 944, Taiwan. The voucher specimen (No. xs-18-yg-113) was deposited at the State Key Laboratory of Marine Drugs, Ocean University of China, People’s Republic of China.

### 3.3. Extraction and Isolation

A frozen specimen of *Lobophytum catalai* (12.0 kg, wet weight) was homogenized and then exhaustively extracted with CH_3_OH six times (5 days each time) at room temperature. The combined solutions were concentrated in vacuo and subsequently desalted by redissolving with ethyl acetate to yield a residue (298.0 g). The crude extract was subjected to silica gel vacuum column chromatography eluted with a gradient of petroleum/ethyl acetate (200:1–1:1, *v*/*v*) and subsequently eluted with a gradient of CH_2_Cl_2_/MeOH (20:1–1:1, *v*/*v*) to obtain fourteen fractions (Frs.1–Frs.14). Each fraction was detected via TLC. Frs.3 was subjected to silica gel vacuum column chromatography (petroleum/ethyl acetate, from 20:1 to 1:1, *v*/*v*) to obtain eight subfractions, Frs.3.1–Frs.3.8. Frs.3.4 was separated via semi-preparative HPLC (ODS, 5 µm, 250 × 10 mm; MeOH/H_2_O, 80:20, *v*/*v*; 2.0 mL/min) to afford **1** (2.6 mg, t_R_ = 82 min). Frs.3.5 was separated via semi-preparative HPLC (ODS, 5 µm, 250 × 10 mm; CH_3_CN/H_2_O, 60:40, *v*/*v*; 2.0 mL/min) to afford **6** (3.2 mg, t_R_ = 70 min). Frs.3.6 was separated via semi-preparative HPLC (ODS, 5 µm, 250 × 10 mm; CH_3_CN/H_2_O, 50:50, *v*/*v*; 2.0 mL/min) to afford **5** (1.8 mg, t_R_ = 110 min). Frs.4 was subjected to silica gel vacuum column chromatography (petroleum/ethyl acetate, 20:1) to obtain six subfractions, Frs.4.1–Frs.4.6. Frs.4.6 was separated via semi-preparative HPLC (ODS, 5 µm, 250 × 10 mm; CH_3_CN/H_2_O, 65:35, *v*/*v*; 2 mL/min) to afford **2** (1.3 mg, t_R_ = 70 min) and **3** (2.4 mg, t_R_ = 90 min). Frs.5 was subjected to silica gel vacuum column chromatography (petroleum/ ethyl acetate, from 20:1 to 1:1, *v*/*v*) to obtain two subfractions, Frs.6.1–Frs.6.2. Frs.6.1 was separated via semi-preparative HPLC (ODS, 5 µm, 250 × 10 mm; MeOH/H_2_O, 65:35, *v*/*v*; 2.0 mL/min) to afford **4** (1.8 mg, t_R_ = 80 min) and **7** (0.9 mg, t_R_ = 121 min).

Lobocatalen A (**1**): colorless oil; [α]${}_{D}^{25}$ −30.3 (*c* 1.0, MeOH); ECD (*c* 0.50, MeOH) = Δε202 −58.2; UV (MeOH) λmax (log ε) = 193 (1.65) nm; IR νmax = 3424, 2362, 1635, 1382 cm^−1^; HRESIMS *m*/*z* 303.2320 [M + H]^+^ (calcd. for C_20_H_31_O_2_^+^, 303.2319). For ^1^H NMR and ^13^C NMR data, see Table 1 and Table 2.

Lobocatalen B (**2**): colorless oil; [α]${}_{D}^{25}$ −40.1 (*c* 1.0, MeOH); ECD (*c* 0.50, MeOH) = Δε197 −10.3; UV (MeOH) λmax (log ε) = 193 (0.38) nm; IR νmax = 3410, 2973, 2931, 2360, 1635 cm^−1^; HRESIMS *m*/*z* 338.2685 [M + NH_4_]^+^ (calcd. for C_20_H_36_O_3_N^+^, 338.2685). For ^1^H NMR and ^13^C NMR data, see Table 1 and Table 2.

Lobocatalen C (**3**): colorless oil; [α]${}_{D}^{25}$ −40.3 (*c* 1.0, MeOH); ECD (*c* 0.50, MeOH) = Δε192 +11, Δε203 −6.9, Δε265 −5.1, Δε302 +4.2; UV (MeOH) λmax (log ε) = 193 (0.48), 273 (0.12) nm; IR νmax = 3410, 2972, 2928, 2361, 1664, 1636 cm^−1^; HRESIMS *m*/*z* 319.2265 [M + H]^+^ (calcd. for C_20_H_31_O_3_^+^, 319.2268). For ^1^H NMR and ^13^C NMR data, see Table 1 and Table 2.

Lobocatalen D (**4**): colorless oil; [α]${}_{D}^{25}$ +50.3 (*c* 0.5, MeOH); ECD (*c* 0.50, MeOH) = Δε190 +13.1, Δε205 −12.2, Δε248 −18.8; UV (MeOH) λmax (log ε) = 201 (1.73) nm, 224 (1.63), 271 (0.53) nm; IR νmax = 3431, 2926, 2361, 1631 cm^−1^; HRESIMS *m*/*z* 305.2475 [M + H]^+^ (calcd. for C_20_H_33_O_2_^+^, 305.2475). For ^1^H NMR and ^13^C NMR data, see Table 1 and Table 2.

Lobocatalen E (**5**): colorless oil; [α]${}_{D}^{25}$ +30. 7 (*c* 1.0, MeOH); ECD (*c* 0.50, MeOH) = Δε193 +46.6; UV (MeOH) λmax (log ε) = 195 (2.03) nm; IR νmax = 3431, 2928, 2361, 1711, 1634 cm^−1^; HRESIMS *m*/*z* 305.2469 [M + H]^+^ (calcd. for C_20_H_33_O_2_^+^, 305.2475). For ^1^H NMR and ^13^C NMR data, see Table 1 and Table 2.

Lobocatalen F (**6**): colorless oil; [α]${}_{D}^{25}$ −10.4 (*c* 1.0, MeOH); ECD (*c* 0.50, MeOH) = Δε199 +4.9, Δε217 +6.7, Δε288 −7.9; UV (MeOH) λmax (log ε) = 195(1.37), 222(0.66), 287(0.18) nm; IR νmax = 3431, 2927, 2360, 1706, 1558 cm^−1^; HRESIMS *m*/*z* 273.2211 [M + H]^+^ (calcd. for C_19_H_29_O_1_^+^, 273.2213). For ^1^H NMR and ^13^C NMR data, see Table 1 and Table 2.

Lobocatalen G (**7**): colorless oil; [α]${}_{D}^{25}$ −30. 5 (*c* 0.5, MeOH); ECD (*c* 0.50, MeOH) = Δε191 +10.3, Δε200 −7.6, Δε216 +6.8, Δε238 −11.1; UV (MeOH) λmax (log ε) = 193 (0.09) nm, 227 (0.12), 270(0.03) nm; IR νmax = 2924, 2854, 1717, 1650, 1540 cm^−1^; HRESIMS *m*/*z* 371.2192 [M + Na]^+^ (calcd. for C_21_H_32_O_4_Na^+^, 371.2193). For ^1^H NMR and ^13^C NMR data, see Table 1 and Table 2.

### 3.4. Anti-Inflammatory Activity Assay

Healthy macrophage fluorescent transgenic zebrafish (Tg: zlyz-EGFP) were provided by the Biology Institute of Shandong Academy of Science (Jinan, China). The zebrafish maintenance and anti-inflammation assay were carried out as previously described [24]. Each zebrafish larva was photographed using a fluorescence microscope (AXIO, Zom.V16), and the number of macrophages around the nerve mound was calculated using Image-Pro Plus 6.0 software (Rockville, MD, USA). One-way analysis of variance was conducted using GraphPad Prism 7.00 software (San Diego, CA, USA). Lobocatalens A–G (**1**–**7**) were tested for anti-inflammatory activities in the zebrafish models. Three dpf (days post-fertilization), healthy macrophage fluorescent transgenic zebrafish were used as animal models to evaluate the anti-inflammatory effects of compounds **1**–**7**.

### 3.5. Cytotoxicity Activity Assay

The MTT method was used to evaluate cytotoxicity against the K562 (human leukemia) cell line, and the SRB method was used to evaluate cytotoxicity against the L-02 (normal human hepatocytes), ASPC-1 (human pancreatic cancer), and MDA-MB-231 (human breast cancer) cell lines. As a positive control, Adriamycin (doxorubicin) was used.

## 4. Conclusions

To the best of our knowledge, less than twenty new lobane diterpenoids have been discovered in the last two decades. In our continuing chemical investigation of the Xisha soft coral *Lobophytum catalai*, seven new lobane diterpenoids, named lobocatalens A–G (**1**–**7**), were isolated, enriching the chemical diversity of lobane diterpenoids. Among them, lobocatalen A (**1**) is a new lobane diterpenoid with an unusual ether linkage between C-14 and C-18. Moreover, extensive spectroscopic data analyses, spectral comparisons, quantum chemical calculations, and TDDFT-ECD calculations were combined to determine the structures and absolute configurations of **1**–**7**. In the bioassay, only compound **7** showed moderate anti-inflammatory activity at 20 µM in the zebrafish models. This is the first report on the anti-inflammatory activity of lobane diterpenoids in zebrafish. In addition, compound **7** showed modest cytotoxic activity against the K562 human cancer cell line. This research suggests that this species has great potential for further evaluation.

## Figures and Tables

**Table 1:** ^1^H NMR data of lobocatalens A–G (**1**–**7**) (CDCl_3_).

No.	1	2	3	4	5	6	7
	*δ*_H_ ^a^ (*J* in Hz)	*δ*_H_ ^b^ (*J* in Hz)	*δ*_H_ ^b^ (*J* in Hz)	*δ*_H_ ^b^ (*J* in Hz)	*δ*_H_ ^a^ (*J* in Hz)	*δ*_H_ ^a^ (*J* in Hz)	*δ*_H_ ^b^ (*J* in Hz)
1							
2	1.99, dd (4.0,12.4)	2.02, m	2.07, dd (3,12.6)	2.01, m	2.01, m	2.12, dd (3.5,12.5)	2.01, dd (4.5,16.5)
3a	1.57, m	1.60, m	1.61, m	1.52, m	1.57, m	1.78, m	1.60, m
3b	1.49, m	1.52, m	1.50, m	1.64, m	1.59, m	1.33, m	1.72, m
4	1.92, m	2.08, m	2.47, m	2.05, m	1.93, m	2.86, m	2.62, m
5a	1.61, m	1.63, m	1.54, m	1.60, m	1.47, m	1.42, m	1.66, m
5b	1.39, m	1.47, m		1.51, m	1.63, m	1.61, m	1.75, m
6a	1.44, m	1.43, m	1.52, m	1.49, m	1.49, m	1.47, m	1.50, m
6b			1.44, m				
7	0.99, s	1.00, s	1.01, s	1.01, s	1.01, s	1.03, s	1.01, s
8	5.80, dd (10.4,17.6)	5.80, dd (10.8,18)	5.80, dd (10.2,18)	5.81, dd (10.5,17.5)	5.82, dd (10.4,17.6)	5.84, m	5.81, dd (11.5,18)
9a	4.91, d (3.6)	4.91, d (3.2)	4.91, d (4.2)	4.93, d (3.0)	4.93, d (4.4)	4.94, m	4.94, m
9b	4.87, s	4.88, s	4.89, s	4.90, d (2.5)	4.89, s		4.90, m
10							
11a	4.81, s	4.81, s	4.57, s	4.84, s	4.82, s	4.84, s	4.84, s
11b	4.57, s	4.57, s	4.80, s	4.59, s	4.59, s	4.61, s	4.61, s
12	1.69, s	1.70, s	1.69, s	1.71, s	1.71, s	1.73, s	1.71, s
13							
14a	5.36, s	5.48, s	7.27, s	2.17, s	4.84, s	1.90, s	6.34, d (18.0)
14b					4.69, s		
15	5.28, m	5.86, m		6.04, s	2.37, m	5.97, d (11.6)	6.80, m
16a	2.62, d (18.0)	1.97, m	2.45, dd (3,16.8)		2.71, m	7.50, m	5.27, m
16b	2.13, d (4.4,18.4)	2.12, m	2.65, dd (15.6,16.8)				
17	4.19, d (4.8)	3.78, dd (3.6,11.2)	4.11, dd (3.0,15.0)	2.63, s		6.11, dd (4.05,15.4)	
18							1.26, s
19	1.29, s	1.27, s	1.31, s	1.26, s	1.40, s	2.29, s	1.23, s
20	1.36, s	1.23, s	1.24, s	1.26, s	1.40, s		
21							2.15, s

^a^ Spectra recorded at 500 MHz. ^b^ Spectra recorded at 600 MHz.

**Table 2:** ^13^C NMR data of lobocatalens A–G (**1**–**7**) (CDCl_3_) ^c^.

No.	1	2	3	4	5	6	7
	*δ*_C_ ^d^	*δ*_C_ ^e^	*δ*_C_ ^e^	*δ*_C_ ^d^	*δ*_C_ ^d^	*δ*_C_ ^d^	*δ*_C_ ^d^
1	39.8, C	39.9, C	39.8, C	39.8, C	40.0, C	39.5, C	39.8, C
2	52.7, CH	52.8, CH	52.8, CH	52.6, CH	52.9, CH	52.3, CH	52.1, CH
3	32.8, CH_2_	34.3, CH_2_	34.3, CH_2_	32.4, CH_2_	33.4, CH_2_	32.0, CH_2_	29.5, CH_2_
4	42.3, CH	40.7, CH	34.72, CH	49.4, CH	45.0, CH	41.2, CH	49.6, CH
5	26.4, CH_2_	26.7, CH_2_	26.6, CH_2_	26.4, CH_2_	27.4, CH_2_	26.2, CH_2_	24.0, CH_2_
6	39.8, CH_2_	39.8, CH_2_	40.0, CH_2_	39.7, CH_2_	40.1, CH_2_	39.7, CH_2_	39.2, CH_2_
7	16.8, CH_3_	16.7, CH_3_	16.7, CH_3_	16.8, CH_3_	16.8, CH_3_	16.7, CH_3_	16.6, CH_3_
8	150.3, CH	150.2, CH	150.3, CH	149.8, CH	150.3, CH	150.0, CH	149.8, CH
9	110.1, CH_2_	110.1, CH_2_	110.1, CH_2_	110.4, CH_2_	110.1, CH_2_	110.3, CH_2_	110.5, CH_2_
10	147.5, C	147.6, C	147.5, C	147.3, C	147.7, C	147.3, C	147.0, C
11	112.4, CH_2_	112.4, CH_2_	112.3, CH_2_	112.6, CH_2_	112.4, CH_2_	112.5, CH_2_	112.9, CH_2_
12	24.9, CH_3_	25.0, CH_3_	25.0, CH_3_	25.0, CH_3_	24.9, CH_3_	25.1, CH_3_	24.9, CH_3_
13	146.3, C	137.5, C	122.9, C	164.4, C	153.2, C	155.5, C	202.0, C
14	99.2, CH	101.0, CH	158.8, CH	18.5, CH_3_	107.6, CH_2_	21.1, CH_3_	130.7, CH
15	115.7, CH	124.6, CH	192.3, C	122.5, CH	28.4, CH_2_	124.2, CH	139.5, CH
16	28.0, CH_2_	25.6, CH_2_	37.3, CH_2_	203.0, C	34.4, CH_2_	138.2, CH	79.2, CH
17	80.1, CH	73.1, CH	84.8, CH	54.4, CH_2_	214.1, C	128.4, CH	72.3, C
18	81.3, C	72.3, C	71.4, C	70.1, C	76.4, C	199.0, C	26.4, CH_3_
19	29.9, CH_3_	26.4, CH_3_	26.0, CH_3_	29.6, CH_3_	26.8, CH_3_	28.3, CH_3_	25.6, CH_3_
20	24.0, CH_3_	23.5, CH_3_	24.6, CH_3_	29.6, CH_3_	26.8, CH_3_		170.1, C
21							21.2, CH_3_

^c^ The assignments were based on HMQC and HMBC spectra. ^d^ Spectra recorded at 125 MHz. ^e^ Spectra recorded at 150 MHz.

**Table 3:** Cytotoxic activity (IC_50_, μM) of compounds **1**–**7**.

Compounds	Cell Line			
	K562	L-02	ASPC-1	MDA-MB-231
**1**	>30	>30	NT ^a^	>30
**2**	>30	>30	NT ^a^	>30
**3**	>30	>30	>30	>30
**4**	>30	>30	>30	>30
**5**	>30	NT ^a^	NT ^a^	NT ^a^
**6**	NT ^a^	>30	NT ^a^	NT ^a^
**7**	27.96	>30	>30	>30
doxorubicin ^b^	<1	<1	<1	<1

^a^ NT: not tested. ^b^ Positive control.

## Data Availability

Data are contained within the article or Supplementary Materials.

## Supplementary Materials

The following supporting information can be downloaded at: https://www.mdpi.com/article/10.3390/md21040223/s1, Tables S1–S7: NMR data of **1**–**7**; Tables S8–S14 and Figures S2–S8: The determination of relative and absolute configurations for compounds **1**–**7**; Table S15: Anti-inflammation assay of **1**–**7**; Tables S16 and S17: Cytotoxic assay of **1**–**7**; Table S18 and Figures S9–S15: Computational details; Figures S16–S103: Spectra for compounds **1**–**7**.
