# Supplementary material for: Seven New Lobane Diterpenoids from the Soft Coral Lobophytum catalai
Source: Mar Drugs. 2023 Mar 30;21(4):223. doi: 10.3390/md21040223 (PMC10143306; doi:10.3390/md21040223)
Supplement: Supplementary file 1 [file marinedrugs-21-00223-s001.zip › Revised Supporting Information.pdf]

Supporting Information

Seven New Lobane Diterpenoids from the Soft Coral *Lobophytum catalai*

Jiarui Zhang [1,2], Huixue Ma [1,2], Shuangshuang Jin [1,2], Xuehuan Liu [1,2], Lei Li [3], Zhaonan Liu [1,2], Guoqiang Li [1,2,*] and Pinglin Li [1,2,*]

[1] Key Laboratory of Marine Drugs, Chinese Ministry of Education, School of Medicine and Pharmacy,Ocean University of China, Qingdao 266003, China

[2] Laboratory of Marine Drugs and Biological Products, National Laboratory for Marine Science and Technology, Qingdao 266235, China

[3] Biology Institute, Qilu University of Technology (Shandong Academy of Sciences), Jinan 250103, China;

* Correspondence: liguoqiang@ouc.edu.cn (G.L.); lipinglin@ouc.edu.cn (P.L.); Tel.: +86-532-8203-2323 (G.L.); +86-532-8203-3054 (P.L.)

Table of Contents

1. Material Information 3

2. NMR data of **1–7**. 4

3. The Determination of Relative and Absolute Configurations for Compounds **1–7** 18

2.1 Conformational search 18

2.2 Quantum chemical NMR calculation 18

2.3 Elucidation of absolute configurations by TDDFT-ECD 32

4. Anti-inflammation assay of **1–7** 33

5. Cytotoxic assay of **1–7** 33

6. Reference 34

7. Computational Details 35

8. The 1D and 2D NMR, MS, UV and IR spectra of **1–7** 222

1. Material Information

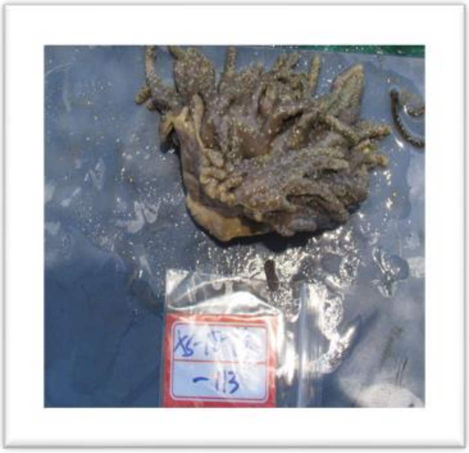

Figure S1. *Lobophytum catalai*

The Soft Coral *Lobophytum catalai* was collected from Xisha Islands (YaGong island) of South China Sea (112°01′E, 16°20′N) in July 2018, and was frozen immediately after collection. The sample is yellow-green in color with mucus adhering to its surface. The specimen was identified by Prof. Ping-Jyun Sung, Institute of Marine Biotechnology, National Museum of Marine Biology & Aquarium, Pingtung 944, Taiwan. The voucher specimen (No. xs-18-yg-113) was deposited at State Key Laboratory of Marine Drugs, Ocean University of China, People's Republic of China.

2. NMR data of 1–7.

Table S1. 1D and 2D NMR Data of Lobocatalen A (**1**)

Position	type	δ_H[b] (*J* in Hz)	δ_C[c]	^1H-^1H COSY	HMBC	NOESY/1D-NOE
		**1**[a]				
1	C		39.83			
2	CH	1.99, dd (3.96,12.39)	52.74	H-3a, H-3b		H-3a, H-8
3a	CH_2	1.57, m	32.75	H-2, H-4	C-1, C-2, C-4, C-5	H-2, H-4
3b		1.49, m		H-2, H-4		H-7
4	CH	1.92, m	42.27	H-3a, H-3b, H-5a, H-5b		H-3a
5a	CH_2	1.61, m	26.39	H-4, H-6		
5b		1.39, m		H-4, H-6		
6	CH_2	1.44, m	39.79	H-5a, H-5b	C-7	
7	CH_3	0.99, s	16.77		C-1, C-2, C-6, C-8	H-12
8	CH	5.80, dd (10.4,17.6)	150.26	H-9a, H-9b	C-7	
9a	CH_2	4.91, d (3.6)	110.07	H-8	C-1, C-8	
9b		4.87, s		H-8	C-1, C-8	
10	C		147.48			
11a	CH_2	4.81, s	112.4			
11b		4.57, s				
12	CH_3	1.69, s	24.89		C-2, C-10, C-11	
13	C		146.31			
14	CH	5.36, s	99.19		C-17, C-18	
15	CH	5.28, m	115.68	H-16a, H-16b	C-4, C-14, C-16	
16a	CH_2	2.62, d (18.0)	27.96	H-15, H-17	C-13, C-17	
16b		2.13, d (4.4,18.4)		H-15, H-17	C-13, C-17	
17	CH	4.19, d (4.8)	80.07	H-16a, H-16b		
18	C		81.24			
19	CH_3	1.29, s	29.88		C-17, C-18, C-20	
20	CH_3	1.35, s	23.95		C-17, C-18, C-19	

[a] Spectra recorded in chloroform -*d*. [b] Spectra recorded at 500 MHz. [c] Spectra recorded at 125 MHz.

Table S2. 1D and 2D NMR Data of Lobocatalen B (**2**)

Position	2[a]					
	type	**δ_H[b] (*J* in Hz)**	**δ_C[c]**	**^{1}H-^{1}H COSY**	**HMBC**	**NOESY/1D-NOE**
1	C		39.88			
2	CH	2.02, m	52.84	H-3a, H-3b		H-3a, H-8
3a	CH_2	1.60, m	34.32	H-2, H-4		H-2, H-4
3b		1.52, m		H-2, H-4		H-7
4	CH	2.08, m	40.68	H-3a, H-3b, H-5a, H-5b		H-3a
5a	CH_2	1.63, m	26.66	H-4, H-6		
5b		1.47, m		H-4, H-6		
6	CH_2	1.43, m	39.83	H-5a, H-5b		
7	CH_3	1.00, s	16.7		C-1, C-2, C-6, C-8	H-12
8	CH	5.80, dd (10.8,18)	150.24	H-9a, H-9b	C-1, C-7	H-2
9a	CH_2	4.91, d (3.2)	110.13	H-8	C-1, C-8	
9b		4.88, s		H-8	C-1, C-8	
10	C		147.55			
11a	CH_2	4.81, s	112.38			
11b		4.57, s				
12	CH_3	1.70, s	24.95		C-2, C-10, C-11	
13	C		137.51			
14	CH	5.48, s	101.04		C-13, C-15, C-17	
15	CH	5.86, m	124.58	H-16a, H-16b	C-4, C-14, C-16, C-17	
16a	CH_2	1.97, m	25.61	H-15, H-17	C-13, C-15, C-17	
16b		2.12, m		H-15, H-17	C-13, C-15, C-17	
17	CH	3.78, dd (3.6,11.2)	73.12	H-16a, H-16b		
18	C		72.27			
19	CH_3	1.27, s	26.41		C-17, C-18, C-20	
20	CH_3	1.23, s	23.54		C-17, C-18, C-19	

[a] Spectra recorded in chloroform -*d*. [b] Spectra recorded at 500 MHz. [c] Spectra recorded at 125 MHz.

Table S3. 1D and 2D NMR Data of Lobocatalen C (**3**)

Position	3[a]					
	type	δ_H[b] (*J* in Hz)	δ_C[c]	^{1}H-^{1}H COSY	HMBC	NOESY/1D-NOE
1	C		39.8			
2	CH	2.07, dd (3,12.6)	52.79	H-3a, H-3b		H-3b, H-4, H-8
3a	CH_2	1.61, m	34.28	H-2, H-4	C-1, C-2, C-4, C-5	H-7
3b		1.50, m		H-2, H-4	C-1, C-2, C-4, C-5	H-2
4	CH	2.47, m	34.72	H-3a, H-3b, H-5a, H-5b		H-2
5	CH_2	1.54, m	26.62	H-4, H-6		
6a	CH_2	1.52, m	40.02	H-5a, H-5b		
6b		1.44, m		H-5a, H-5b		
7	CH_3	1.01, s	16.69		C-1, C-2, C-6, C-8	H-3a, H-11a
8	CH	5.82, dd (10.2,18)	150.26	H-9a, H-9b	C-1, C-2, C-7	
9a	CH_2	4.91, d (4.2)	110.12	H-8	C-1, C-8	
9b		4.89, s		H-8	C-1, C-8	
10	C		147.54			
11a	CH_2	4.57, s	112.25			H-7
11b		4.80, s				
12	CH_3	1.69, s	25.01		C-2, C-10, C-11	
13	C		122.87			
14	CH	7.27, s	158.84		C-4, C-13, C-15, C-17	
15	C		192.28			
16a	CH_2	2.45, dd (3,16.8)	37.27	H-17	C-15, C-17, C-18	
16b		2.65, dd (15.6,16.8)		H-17	C-15, C-17, C-18	
17	CH	4.11, dd (3.0,15.0)	84.8	H-16a, H-16b		
18	C		71.36			
19	CH_3	1.31, s	25.96		C-17, C-18, C-20	
20	CH_3	1.24, s	24.63		C-17, C-18, C-19	

[a] Spectra recorded in chloroform -*d*. [b] Spectra recorded at 600 MHz. [c] Spectra recorded at 150 MHz.

Table S4. 1D and 2D NMR Data of Lobocatalen D (**4**)

Position	4[a]					
	type	δ_H[b] (*J* in Hz)	δ_C[c]	^{1}H-^{1}H COSY	HMBC	NOESY/1D-NOE
1	C		39.81			
2	CH	2.01, m	52.63	H-3a, H-3b		H-3a, H-8
3a	CH_2	1.52, m	32.38	H-2, H-4		H-2, H-4
3b		1.64, m		H-2, H-4		H-7
4	CH	2.05, m	49.41	H-3a, H-3b, H-5a, H-5b		H-3a
5a	CH_2	1.60, m	26.4	H-4, H-6		
5b		1.51, m		H-4, H-6		
6	CH_2	1.49, m	39.67	H-5a, H-5b		
7	CH_3	1.01, s	16.78		C-1, C-2, C-6, C-8	H-3b, H-12
8	CH	5.81, dd (10.5,17.5)	149.83	H-9a, H-9b	C-1, C-7	H-2
9a	CH_2	4.93, d (3.0)	110.44	H-8	C-1, C-8	
9b		4.90, d (2.5)		H-8	C-1, C-8	
10	C		147.26			
11a	CH_2	4.84, s	112.62		C-2, C-12	
11b		4.59, s			C-2, C-12	
12	CH_3	1.71, s	24.98		C-2, C-10, C-11	
13	C		164.43			
14	CH_3	2.17, s	18.48		C-4, C-13, C-15	
15	CH	6.04, s	122.55		C-4, C-14, C-16	
16	C		203			
17	CH_2	2.63, s	54.37		C-16, C-18, C-19, C-20	
18	C		70.08			
19	CH_3	1.26, s	29.58		C-17, C-18, C-20	
20	CH_3	1.26, s	29.58		C-17, C-18, C-19	

[a] Spectra recorded in chloroform -*d*. [b] Spectra recorded at 600 MHz. [c] Spectra recorded at 150 MHz.

Table S5. ^{1}H and ^{13}C NMR Data of Lobocatalen E (**5**)

Position	**5**[a]					
	type	**δ_H[d] (J in Hz)**	**δ_C[e]**	**^{1}H-^{1}H COSY**	**HMBC**	**NOESY/1D-NOE**
1	C		39.96			
2	CH	2.01, m	52.93	H-3a, H-3b		H-8
3a	CH_2	1.57, m	33.42	H-2, H-4	C-1, C-2, C-5	
3b		1.59, m		H-2, H-4	C-1, C-2, C-5	
4	CH	1.93, m	45.01	H-3a, H-3b, H-5a, H-5b		
5a	CH_2	1.47, m	27.37	H-4, H-6		
5b		1.63, m		H-4, H-6		
6	CH_2	1.49, m	40.06	H-5a, H-5b		
7	CH_3	1.01, s	16.77		C-1, C-2, C-6, C-8	H-12
8	CH	5.81, dd (10.4,17.6)	150.27	H-9a, H-9b	C-1, C-7	H-2
9a	CH_2	4.93, d (4.4)	110.13	H-8	C-1, C-8	
9b		4.89, s		H-8	C-1, C-8	
10	C		147.69			
11a	CH_2	4.82, s	112.35		C-2, C-12	H-7
11b		4.59, s			C-2, C-12	
12	CH_3	1.71, s	24.94		C-2, C-10, C-11	
13	C		153.16			
14a	CH_2	4.84, s	107.55		C-4, C-13, C-15	
14b		4.69, s			C-4, C-13, C-15	
15	CH_2	2.37, m	28.41	H-16	C-4, C-13, C-14, C-16, C-17	
16	CH_2	2.71, m	34.4	H-15	C-13, C-15, C-17	
17	C		214.08			
18	C		76.35			
19	CH_3	1.40, s	26.75		C-17, C-18, C-20	
20	CH_3	1.40, s	26.75		C-17, C-18, C-19	

[a] Spectra recorded in chloroform -d4. [d] Spectra recorded at 500 MHz. [e] Spectra recorded at 125 MHz.

Table S6. 1D and 2D NMR Data of Lobocatalen F (**6**)

Position	6^a					
	type	δ_H[b] (*J* in Hz)	δ_C[c]	^{1}H-^{1}H COSY	HMBC	NOESY/1D-NOE
1	C		39.51			
2	CH	2.12, dd (3.5,12.5)	52.3	H-3a, H-3b		H-3b
3a	CH_2	1.78, m	32.01	H-2, H-4	C-1, C-2, C-4, C-5	H-7
3b		1.33, m		H-2, H-4	C-1, C-2, C-4, C-5	H-2, H-4
4	CH	2.86, m	41.17	H-3a, H-3b, H-5a, H-5b		H-3b
5a	CH_2	1.42, m	26.19	H-4, H-6		
5b		1.61, m		H-4, H-6		
6	CH_2	1.47, m	39.67	H-5a, H-5b		
7	CH_3	1.03, s	16.73		C-1, C-2, C-6, C-8	H-3a
8	CH	5.84, m	150	H-9	C-1, C-7	
9	CH_2	4.94, m	110.33	H-8	C-1, C-8	
10	C		147.35			
11a	CH_2	4.84, s	112.49		C-2, C-12	
11b		4.61, s			C-2, C-12	
12	CH_3	1.73, s	25.05		C-2, C-10, C-11	
13	C		155.54			
14	CH_3	1.90, s	21.06		C-4, C-13, C-15	H-15
15	CH	5.97, d (12.0)	124.16	H-16	C-4, C-14	H-14
16	CH	7.50, m	138.24	H-15, H-17	C-13, C-15, C-17, C-18	
17	CH	6.11, d (15.0)	128.43	H-19	C-15, C-18	
18	C		198.96			
19	CH_3	2.29, s	28.27		C-17, C-18	

[a] Spectra recorded in chloroform -*d*. [b] Spectra recorded at 500 MHz. [c] Spectra recorded at 125 MHz.

Table S7.^{1}H and ^{13}C NMR Data of Lobocatalen G (**7**)

Position	7[a]					
	type	δ_H[b] (*J* in Hz)	δ_C[c]	^{1}H-^{1}H COSY	HMBC	NOESY/1D-NOE
1	C		39.82			
2	CH	2.01, dd (4.5,16.5)	52.12	H-3a, H-3b		H-4
3a	CH_2	1.60, m	29.51	H-2, H-4		
3b		1.72, m		H-2, H-4		
4	CH	2.62, m	49.62	H-3a, H-3b, H-5a, H-5b		H-2, H-3b
5a	CH_2	1.66, m	23. 98	H-4, H-6		
5b		1.75, m		H-4, H-6		
6	CH_2	1.50, m	39.19	H-5a, H-5b		
7	CH_3	1.01, s	16.64		C-1, C-2, C-6, C-8	H-3a
8	CH	5.81, dd (11.5,18)	149.77	H-9	C-1, C-7	
9a	CH_2	4.94, m	110.49	H-8	C-1, C-8	
9b		4.90, m		H-8	C-1, C-8	
10	C		146.95			
11a	CH_2	4.84, s	112.91		C-2, C-12	
11b		4.60, s			C-2, C-12	
12	CH_3	1.71, s	24.85		C-2, C-10, C-11	
13	C		202.04			
14	CH	6.34, d (15.0)	130.65	H-15	C-13, C-16	H-16
15	CH	6.80, m	139.53	H-14, H-16	C-13, C-16	
16	CH	5.27, m	79.16	H-15	C-14, C-15, C-17, C-19, C-20	H-14
17	C		72.32			
18	CH_3	1.26, s	26.38		C-16, C-17, C-19	
19	CH_3	1.23, s	25.59		C-16, C-17, C-18	
20	C		170.08			
21	CH_3	2.15, s	21.16		C-20	

[a] Spectra recorded in chloroform -d. [b] Spectra recorded at 600 MHz. [c] Spectra recorded at 150 MHz.

3. The Determination of Relative and Absolute Configurations for Compounds 1–7

3.1 Conformational search

Conformational search of all possible configurations were carried out by MacroModel integrated in Maestro V11.9 (Schrödinger Inc.)[1]. The OPLS3e force field[2] and an energy below a threshold of 10 kJ mol^{-1} were employed[3]. Eliminating redundant conformer used root-mean-squared-distance (RMSD) cutoff of 0.5 Å and the maximum iterations was 2500. After energy minimization, the unstable configurations were excluded. For compound **1**, The unstable configurations (14R,17S; 14S,17R) were deleted from all possible conformation by Maestro V11.9 (Schrodinger Inc.).

3.2 Quantum chemical NMR calculation

In order to establish the relative configuration of molecules **1–7**. ^{13}C NMR chemical shifts were calculated by Gaussian 16 program package[4]. Excluding unstable conformers by conformational search, the remaining conformers were optimized with the density functional theory (DFT) at the B3LYP/6-31G (d, p) level[5], and all minima displayed no imaginary frequencies by vibrational frequency analysis at the same level. The populations of conformers were calculated according to the Boltzmann distribution theory and their relative Gibbs free energy. GIAO calculations of NMR shielding were accomplished for all stable conformations by DFT GIAO model at PCM/mPW1PW91/6-31+G**[6] level for $DP4^{+}$ calculations. The qccNMR results were shown in the following Tables S8-S14 and Figures S2-S8.

Table S8. Experimental NMR data and calculated NMR data for **1**

Nuclei	SP²	Experiment **1**	Calculation	
			Conf.1（1*R*, 2*R*, 4*S*, 14*S*, 17*S*）	Conf.2（1*R*, 2*R*, 4*S*, 14*R*, 17*R*）
C	x	110.07	108.867	108.872
C	x	112.4	110.687	110.843
C	x	147.48	154.405	154.14
C		52.74	53.876	53.971
C		39.83	44.17	43.076
C	X	150.26	154.217	153.447
C		32.75	33.718	35.007
C		42.27	45.916	44.578
C		26.39	28.39	27.152
C		39.79	40.614	40.587
C		16.77	15.097	15.326
C		24.89	27.473	27.457
C	x	146.31	151.413	151.368
C	x	115.68	118.782	118.027
C		27.96	30.177	30.167
C		80.07	82.393	82.422
C		99.79	102.255	102.743
C		81.24	84.108	84.016
C		29.88	29.013	28.989
C		23.95	22.919	22.936
H		1.99	2.084	2.088
H		1.93	1.984	1.982
H	x	4.91	5.061	5.079
H	x	4.87	5.016	5.025
H	x	4.81	5.07	5.071
H	x	4.57	4.705	4.702
H	x	5.8	6.109	6.085
H		1.49	1.71	1.638
H		1.57	1.429	1.525
H		1.61	1.539	1.499
H		1.4	1.555	1.561
H		1.44	1.484	1.476
H		1.44	1.484	1.476
H		0.99	1.113	1.1
H		0.99	1.113	1.1
H		0.99	1.113	1.1
H		1.69	1.826	1.821
H		1.69	1.826	1.821
H		1.69	1.826	1.821
H	x	5.28	5.334	5.306
H		2.13	2.594	2.61
H		2.62	2.179	2.185
H		4.19	4.013	4.021
H		5.37	5.161	5.128
H		1.29	1.255	1.257
H		1.29	1.255	1.257
H		1.29	1.255	1.257
H		1.36	1.345	1.342
H		1.36	1.345	1.342
H		1.36	1.345	1.342

Functional	Solvent?	Basis Set	Type of Data
mPW1PW91	PCM	6-31+G(d,p)	Unscaled Shifts

	Isomer 1	Isomer 2	Isomer 3	Isomer 4	Isomer 5	Isomer 6
sDP4+ (H data)	29.92%	70.08%	–	–	–	–
sDP4+ (C data)	17.62%	82.38%	–	–	–	–
sDP4+ (all data)	8.37%	91.63%	–	–	–	–
uDP4+ (H data)	20.65%	79.35%	–	–	–	–
uDP4+ (C data)	11.17%	88.83%	–	–	–	–
uDP4+ (all data)	3.17%	96.83%	–	–	–	–
DP4+ (H data)	10.00%	90.00%	–	–	–	–
DP4+ (C data)	2.62%	97.38%	–	–	–	–
DP4+ (all data)	0.30%	99.70%	–	–	–	–

Figure S2. The $DP4^+$ results between calculated and experimental NMR data for **1**

Table S9. Experimental NMR data and calculated NMR data for **2**

Nuclei	SP[2]	Experiment **2**	Calculation			
			Conf.1（1*R*, 2*R*, 4*S*, 14*S*, 17*S*）	Conf.2（1*R*, 2*R*, 4*S*, 14*S*, 17*R*）	Conf.3（1*R*, 2*R*, 4*S*, 14*R*, 17*S*）	Conf.4（1*R*, 2*R*, 4*S*, 14*R*, 17*R*）
C	x	110.12	108.78	108.76	108.731	108.817
C	x	112.38	110.92	110.68	110.579	110.834
C	x	147.55	154.07	154.54	154.388	154.088
C		52.84	54.40	53.87	54.154	54.316
C		39.88	43.88	44.37	44.174	43.233
C	x	150.24	154.07	154.16	154.237	154.199
C		34.32	34.78	33.55	35.846	36.463
C		40.68	44.05	41.84	41.772	43.948
C		26.66	30.58	29.60	27.785	28.741
C		39.83	40.65	40.73	40.351	40.791
C		16.70	15.80	15.41	15.373	15.205
C		24.95	26.79	27.17	27.298	27.326
C	x	137.51	147.19	146.84	147.029	146.056
C	x	124.58	124.54	123.37	123.728	124.413
C		25.61	27.28	27.28	27.296	27.47
C		73.12	73.66	79.74	80.032	73.929
C		101.04	94.85	97.33	97.362	94.676
C		72.27	73.52	73.57	73.563	73.504
C		23.54	22.67	22.61	22.541	22.828
C		26.41	25.52	25.72	25.513	25.414
H		2.02	2.11	2.14	2.133	2.129
H		2.08	2.14	2.37	2.365	2.138
H		3.78	3.47	3.51	3.506	3.474
H	x	4.91	5.07	5.05	5.08	5.077
H	x	4.88	5.02	5.02	5.027	5.027
H	x	4.81	5.08	5.08	5.063	5.07
H	x	4.57	4.68	4.72	4.667	4.725
H	x	5.80	6.12	6.13	6.117	6.126
H		1.60	1.69	1.76	1.531	1.886
H		1.52	1.577	1.44	1.655	1.399
H		1.63	1.401	1.75	1.492	1.635
H		1.47	1.709	1.35	1.59	1.553
H		1.43	1.500	1.51	1.515	1.515
H		1.43	1.500	1.51	1.515	1.515
H		1.00	1.134	1.108	1.105	1.13
H		1.00	1.134	1.108	1.105	1.13
H		1.00	1.134	1.108	1.105	1.13
H		1.7	1.830	1.835	1.829	1.83
H		1.7	1.830	1.835	1.829	1.83
H		1.7	1.830	1.835	1.829	1.83
H	x	5.86	5.861	5.729	5.729	5.873
H		2.12	1.943	1.888	2.365	2.316
H		1.97	2.304	2.432	1.916	1.936
H		5.48	5.302	5.397	5.367	5.3
H		1.23	1.137	1.116	1.119	1.133
H		1.23	1.137	1.116	1.119	1.133
H		1.23	1.137	1.116	1.119	1.133
H		1.27	1.214	1.236	1.218	1.22

H	1.27	1.214	1.236	1.218	1.22
H	1.27	1.214	1.236	1.218	1.22

Functional	Solvent?	Basis Set	Type of Data			
mPW1PW91	PCM	6-31+G(d, p)	Unscaled Shifts			
	Isomer 1	Isomer 2	Isomer 3	Isomer 4	Isomer 5	Isomer 6
sDP4+ (H data)	4.55%	0.27%	5.45%	89.73%	–	–
sDP4+ (C data)	21.17%	2.53%	13.12%	63.18%	–	–
sDP4+ (all data)	1.65%	0.01%	1.23%	97.11%	–	–
uDP4+ (H data)	2.14%	0.54%	6.40%	90.92%	–	–
uDP4+ (C data)	6.95%	1.41%	1.99%	89.65%	–	–
uDP4+ (all data)	0.18%	0.01%	0.16%	99.65%	–	–
DP4+ (H data)	0.12%	0.00%	0.43%	99.45%	–	–
DP4+ (C data)	2.52%	0.06%	0.45%	96.97%	–	–
DP4+ (all data)	0.00%	0.00%	0.00%	99.99%	–	–

Figure S3. The $DP4^+$ results between calculated and experimental NMR data for **2**

Table S10. Experimental NMR data and calculated NMR data for **3**

Nuclei	SP^2	Experiment **3**	Calculation Conf.1 （1*R*, 2*R*, 4*S*, 17*S*）	Conf.2 （1*R*, 2*R*, 4*S*, 17*R*）	Conf.3 （1*R*, 2*R*, 4*R*, 17*S*）	Conf.4 （1*R*, 2*R*, 4*R*, 17*R*）
C	x	110.12	108.763	109.463	108.48	108.715
C	x	112.25	110.422	111.475	110.774	110.757
C	x	147.54	154.411	154.291	153.913	153.627
C		52.79	54.081	54.692	48.457	48.814
C		39.8	44.149	45.149	45.28	45.282
C	x	150.26	154.187	154.172	154.284	154.275
C		34.28	35.149	34.973	32.546	30.442
C		34.72	38.097	38.829	33.945	33.83
C		26.62	27.753	30.163	24.578	26.177
C		40.02	40.78	41.73	36.483	36.131
C		25.01	27.497	28.385	26.827	27.686
C		16.69	15.012	16.605	15.162	14.669
C	x	122.87	124.133	124.371	121.921	121.3
C	x	192.28	193.862	194.273	193.969	194.29
C		37.27	38.512	39.222	38.703	38.652
C		84.60	87.491	88.464	87.129	87.223
C	x	158.84	161.022	161.248	162.51	162.409
C		71.36	73.913	74.793	73.913	73.966
C		25.96	24.942	26.472	24.885	24.959
C		24.63	23.425	24.375	22.955	23.236
H		2.07	2.053	2.144	2.066	2.192
H		2.47	2.352	2.441	2.814	2.819
H		4.11	4.013	4.142	4.181	4.167
H	x	4.91	4.849	5.067	5.036	5.047
H	x	4.89	4.823	5.022	4.999	5.003
H	x	4.80	4.852	5.045	5.039	5.082
H	x	4.55	4.478	4.643	4.688	4.703
H	x	5.82	5.994	6.12	6.079	6.097
H		1.61	1.554	1.676	1.752	1.582
H		1.50	1.285	1.467	2.075	2.061
H		1.54	1.429	1.531	1.808	1.858
H		1.54	1.429	1.531	1.808	1.858
H		1.52	1.53	1.602	1.69	1.587
H		1.44	1.304	1.412	1.205	1.153
H		1.69	1.694	1.829	1.772	1.807
H		1.69	1.694	1.829	1.772	1.807
H		1.69	1.694	1.829	1.772	1.807
H		1.01	1.006	1.124	1.144	1.119
H		1.01	1.006	1.124	1.144	1.119
H		1.01	1.006	1.124	1.144	1.119
H		2.65	2.111	2.779	2.254	2.756
H		2.45	2.649	2.262	2.709	2.267
H	x	7.27	7.01	7.219	7.423	7.372
H		1.31	1.169	1.283	1.285	1.303
H		1.31	1.169	1.283	1.285	1.303
H		1.31	1.169	1.283	1.285	1.303
H		1.24	1.063	1.197	1.222	1.209

H	1.24	1.063	1.197	1.222	1.209
H	1.24	1.06	1.20	1.22	1.21

Functional	Solvent?	Basis Set	Type of Data
mPW1PW91	PCM	6-31+G(d, p)	Unscaled Shifts

	Isomer 1	Isomer 2	Isomer 3	Isomer 4	Isomer 5	Isomer 6
sDP4+ (H data)	2.11%	97.85%	0.01%	0.03%	–	–
sDP4+ (C data)	45.18%	54.79%	0.02%	0.00%	–	–
sDP4+ (all data)	1.75%	98.25%	0.00%	0.00%	–	–
uDP4+ (H data)	0.00%	100.00%	0.00%	0.00%	–	–
uDP4+ (C data)	2.10%	97.90%	0.00%	0.00%	–	–
uDP4+ (all data)	0.00%	100.00%	0.00%	0.00%	–	–
DP4+ (H data)	0.00%	100.00%	0.00%	0.00%	–	–
DP4+ (C data)	1.74%	98.26%	0.00%	0.00%	–	–
DP4+ (all data)	0.00%	100.00%	0.00%	0.00%	–	–

Figure S4. The DP4+ results between calculated and experimental NMR data for **3**

Table S11. Experimental NMR data and calculated NMR data for **4**

Nuclei	SP[2]	Experiment **4**	Calculation			
			Conf.1 (1*R*, 2*R*, 4*S*)	Conf.2 (1*R*, 2*R*, 4*R*)	Conf.3 (1*R*, 2*S*, 4*R*)	Conf.4 (1*R*, 2*S*, 4*S*)
C	x	110.44	108.809	108.995	111.238	111.384
C	x	112.62	111.352	110.521	111.348	111.026
C	x	147.26	153.694	154.193	153.755	153.520
C		52.63	54.254	48.389	52.730	58.111
C		39.81	43.872	45.203	43.935	43.663
C	x	149.83	153.472	153.871	146.771	146.907
C		32.38	33.430	31.464	31.419	33.841
C		49.41	53.714	47.441	47.752	53.972
C		26.40	27.655	24.747	25.651	27.840
C		39.67	39.172	36.070	37.372	43.501
C		24.98	26.725	27.551	22.657	23.361
C		16.78	16.646	14.522	26.842	25.042
C	x	164.43	177.158	174.512	174.447	176.862
C	x	122.55	120.515	122.622	122.816	120.223
C		18.48	22.873	23.223	23.212	23.060
C	x	202.00	207.004	206.043	205.996	206.890
C		54.37	53.942	54.500	54.468	54.052
C		70.08	72.703	72.641	72.646	72.715
C		29.58	27.665	28.044	28.033	28.009
C		29.58	28.406	28.044	28.033	28.009
H		2.01	2.127	2.019	2.158	2.141
H		2.05	2.201	2.544	2.609	2.276
H	x	4.93	5.094	5.028	5.252	5.230
H	x	4.90	5.046	5.006	5.146	5.121
H	x	4.84	5.088	5.083	4.947	4.920
H	x	4.59	4.713	4.690	4.753	4.744
H	x	5.81	6.114	6.045	6.840	6.885
H		1.64	1.825	1.840	2.207	1.943
H		1.52	1.482	2.136	1.809	1.505
H		1.60	1.558	1.901	1.865	1.506
H		1.51	1.669	1.916	2.034	1.826
H		1.49	1.546	1.453	1.518	1.563
H		1.49	1.546	1.453	1.518	1.563
H		1.71	1.835	1.785	1.798	1.799
H		1.71	1.835	1.785	1.798	1.799
H		1.71	1.835	1.785	1.798	1.799
H		1.01	1.137	1.146	1.005	1.073
H		1.01	1.137	1.146	1.005	1.073
H		1.01	1.137	1.146	1.005	1.073
H	x	6.04	6.025	6.220	6.188	6.067
H		2.17	2.296	2.320	2.322	2.316
H		2.17	2.296	2.320	2.322	2.316
H		2.17	2.296	2.320	2.322	2.316
H		2.63	2.644	2.692	2.694	2.650
H		2.63	2.644	2.692	2.694	2.650
H		1.26	1.179	1.192	1.202	1.179

H	1.26	1.179	1.192	1.202	1.179
H	1.26	1.179	1.192	1.202	1.179
H	1.26	1.184	1.224	1.201	1.188
H	1.26	1.184	1.224	1.201	1.188
H	1.26	1.184	1.224	1.201	1.188

Functional	Solvent?	Basis Set	Type of Data
mPW1PW91	PCM	6-31+G(d,p)	Unscaled Shifts

	Isomer 1	Isomer 2	Isomer 3	Isomer 4	Isomer 5	Isomer 6
sDP4+ (H data)	99.94%	0.01%	0.00%	0.04%	–	–
sDP4+ (C data)	98.29%	1.70%	0.01%	0.00%	–	–
sDP4+ (all data)	100.00%	0.00%	0.00%	0.00%	–	–
uDP4+ (H data)	99.65%	0.02%	0.00%	0.34%	–	–
uDP4+ (C data)	91.71%	0.00%	0.00%	8.29%	–	–
uDP4+ (all data)	99.97%	0.00%	0.00%	0.03%	–	–
DP4+ (H data)	100.00%	0.00%	0.00%	0.00%	–	–
DP4+ (C data)	100.00%	0.00%	0.00%	0.00%	–	–
DP4+ (all data)	100.00%	0.00%	0.00%	0.00%	–	–

Figure S5. The DP4+ results between calculated and experimental NMR data for **4**

Table S12. Experimental NMR data and calculated NMR data for **5**

Nuclei	SP²	Experiment 5	Calculation	
			Conf.1（1*R*, 2*R*, 4*S*）	Conf.2（1*R*, 2*R*, 4*R*）
C	x	110.13	109.019	108.523
C	x	112.35	110.371	110.068
C	x	147.69	154.506	154.706
C		52.93	54.063	47.644
C		39.96	44.542	45.433
C	x	150.27	154.336	154.420
C		33.42	35.228	31.193
C		45.01	48.390	41.535
C		27.37	29.113	24.939
C		40.06	41.070	35.848
C		16.77	14.839	14.694
C		24.94	27.744	27.633
C	x	153.16	160.335	155.080
C		28.41	29.856	31.529
C		34.20	37.579	36.841
C	x	214.08	221.240	221.152
C		76.35	78.880	78.867
C	x	107.55	106.202	109.175
C		26.75	26.371	26.741
C		26.75	26.371	26.741
H		2.01	2.102	2.205
H		1.93	2.102	2.470
H	x	4.93	5.064	5.006
H	x	4.89	5.031	4.986
H	x	4.82	5.059	5.051
H	x	4.58	4.695	4.684
H	x	5.82	6.111	6.092
H		1.57	1.622	1.870
H		1.57	1.622	1.870
H		1.63	1.640	1.857
H		1.47	1.618	1.825
H		1.47	1.494	1.439
H		1.47	1.494	1.439
H		1.01	1.141	1.135
H		1.01	1.141	1.135
H		1.01	1.141	1.135
H		1.71	1.822	1.804
H		1.71	1.822	1.804
H		1.71	1.822	1.804
H		2.37	2.434	2.405
H		2.37	2.434	2.405
H		2.71	2.805	2.819
H		2.71	2.805	2.819
H	x	4.84	4.829	5.127

H	x	4.69	4.929	5.044
H		1.4	1.361	1.383
H		1.4	1.361	1.383
H		1.4	1.361	1.383
H		1.40	1.374	1.386
H		1.40	1.374	1.386
H		1.40	1.374	1.386

Functional	Solvent?	Basis Set	Type of Data
mPW1PW91	PCM	6-31+G(d,p)	Unscaled Shifts

	Isomer 1	Isomer 2	Isomer 3	Isomer 4	Isomer 5	Isomer 6
sDP4+ (H data)	100.00%	0.00%	–	–	–	–
sDP4+ (C data)	100.00%	0.00%	–	–	–	–
sDP4+ (all data)	100.00%	0.00%	–	–	–	–
uDP4+ (H data)	99.93%	0.07%	–	–	–	–
uDP4+ (C data)	100.00%	0.00%	–	–	–	–
uDP4+ (all data)	100.00%	0.00%	–	–	–	–
DP4+ (H data)	100.00%	0.00%	–	–	–	–
DP4+ (C data)	100.00%	0.00%	–	–	–	–
DP4+ (all data)	100.00%	0.00%	–	–	–	–

Figure S6. The DP4+ results between calculated and experimental NMR data for **5**

Table S13. Experimental NMR data and calculated NMR data for **6**

Nuclei	SP²	Experiment **6**	Calculation			
			Conf.1 (1*R*, 2*R*, 4*S*)	Conf.2 (1*R*, 2*R*, 4*R*)	Conf.3 (1*S*, 2*R*, 4*R*)	Conf.4 (1*S*, 2*R*, 4*S*)
C	x	110.33	109.03	108.36	111.67	107.37
C	x	112.49	111.16	111.57	110.71	113.26
C	x	147.35	153.48	154.96	153.80	153.04
C		52.3	54.19	52.41	56.88	52.06
C		39.51	44.35	41.88	44.28	43.60
C	x	150	153.90	150.01	146.25	153.00
C		32.04	34.04	33.30	35.00	32.94
C		41.17	44.37	38.70	45.10	38.98
C		26.19	27.51	27.37	28.62	27.29
C		39.67	40.21	35.70	42.19	31.89
C		25.05	26.49	27.58	25.89	26.65
C		16.73	15.79	22.98	25.81	28.18
C	x	155.54	165.44	165.88	165.50	165.16
C	x	124.16	124.53	124.57	124.57	124.55
C		21.06	22.48	23.47	22.59	22.78
C	x	138.24	140.87	141.75	140.64	141.70
C	x	128.43	127.55	128.12	127.15	128.42
C	x	198.96	199.63	199.99	199.57	200.14
C		28.27	27.71	27.15	27.97	26.70
H		2.12	2.19	2.11	2.13	2.22
H		2.86	2.97	3.27	3.04	3.12
H	x	4.94	5.07	5.14	5.22	5.01
H	x	4.94	5.07	5.14	5.22	5.01
H	x	4.84	5.08	5.21	5.02	5.24
H	x	4.60	4.71	5.11	4.73	4.97
H	x	5.84	6.13	6.92	6.88	6.07
H		1.78	2.05	2.20	2.08	2.13
H		1.33	1.31	1.32	1.33	1.35
H		1.61	1.37	2.05	2.04	1.92
H		1.42	1.895	1.48	1.33	1.55
H		1.47	1.566	1.74	1.66	1.93
H		1.47	1.566	1.74	1.66	1.93
H		1.73	1.834	1.92	1.85	1.84
H		1.73	1.834	1.92	1.85	1.84
H		1.73	1.834	1.92	1.85	1.84
H		1.03	1.182	1.12	1.08	1.20
H		1.03	1.182	1.12	1.08	1.20
H		1.03	1.182	1.12	1.08	1.20
H	x	5.97	6.077	6.077	6.05	6.03

H		1.9	2.028	2.019	2.03	2.01
H		1.9	2.028	2.019	2.03	2.01
H		1.9	2.028	2.019	2.03	2.01
H		7.5	7.648	7.608	7.68	7.53
H	x	6.11	5.899	5.793	5.93	5.75
H		2.29	2.264	2.256	2.26	2.23
H		2.29	2.264	2.256	2.26	2.23
H		2.29	2.264	2.256	2.262	2.234

Functional	Solvent?	Basis Set	Type of Data
mPW1PW91	PCM	6-31+G(d,p)	Unscaled Shifts

	Isomer 1	Isomer 2	Isomer 3	Isomer 4	Isomer 5	Isomer 6
sDP4+ (H data)	99.92%	0.00%	0.07%	0.00%	-	-
sDP4+ (C data)	99.96%	0.00%	0.03%	0.00%	-	-
sDP4+ (all data)	100.00%	0.00%	0.00%	0.00%	-	-
uDP4+ (H data)	76.95%	0.04%	22.91%	0.10%	-	-
uDP4+ (C data)	58.83%	0.06%	41.12%	0.00%	-	-
uDP4+ (all data)	82.78%	0.00%	17.22%	0.00%	-	-
DP4+ (H data)	99.98%	0.00%	0.02%	0.00%	-	-
DP4+ (C data)	99.98%	0.00%	0.02%	0.00%	-	-
DP4+ (all data)	100.00%	0.00%	0.00%	0.00%	-	-

Figure S7 The DP4+ results between calculated and experimental NMR data for **6**

Table S14. Experimental NMR data and calculated NMR data for **7**

Nuclei	SP[2]	Experiment **7**	Calculation	
			Conf.1（1*R*, 2*R*, 4*S*, 16*S*）	Conf.2（1*R*, 2*R*, 4*S*, 16*R*）
C	x	110.16	109.33	109.37
C	x	112.91	111.03	111.07
C	x	146.95	153.76	153.71
C		52.12	53.21	53.24
C		39.82	44.18	44.29
C	x	149.77	154.11	154.01
C		23.98	31.64	31.92
C		46.62	51.30	50.39
C		29.51	25.96	26.17
C		39.19	40.48	40.37
C		16.64	14.66	14.56
C		24.85	27.71	27.86
C	x	202.04	206.19	206.55
C	x	130.65	135.04	135.57
C	x	139.53	143.09	142.91
C		79.16	82.83	83.14
C		72.32	74.44	74.41
C		26.38	25.59	25.46
C		25.59	25.44	23.45
C	x	170.08	172.27	172.30
C		21.16	21.19	21.19
H		2.02	2.09	2.08
H		2.62	2.83	2.88
H	x	4.94	5.08	5.08
H	x	4.90	5.04	5.05
H	x	4.84	5.07	5.08
H	x	4.60	4.71	4.72
H	x	5.81	6.10	6.07
H		1.72	1.92	1.92
H		1.69	1.46	1.43
H		1.75	1.63	1.63
H		1.66	1.63	1.63
H		1.50	1.50	1.54
H		1.50	1.50	1.44
H		1.01	1.13	1.13
H		1.01	1.13	1.13
H		1.01	1.13	1.13
H		1.71	1.84	1.82
H		1.71	1.84	1.82
H		1.71	1.84	1.82

H	x	6.34	6.29	6.24
H	x	6.80	6.92	6.88
H		5.27	5.12	5.01
H		1.26	1.21	1.22
H		1.26	1.21	1.22
H		1.26	1.21	1.22
H		1.23	1.22	1.22
H		1.23	1.22	1.22
H		1.23	1.22	1.22
H		2.15	2.11	2.12
H		2.15	2.11	2.12
H		2.15	2.11	2.12

	A	B	C	D	E	F	G	H
1	Functional		Solvent?		Basis Set		Type of Data	
2	mPW1PW91		PCM		6-31+G(d,p)		Unscaled Shifts	
3								
4			Isomer 1	Isomer 2	Isomer 3	Isomer 4	Isomer 5	Isomer 6
5	sDP4+ (H data)		92.18%	7.82%	-	-	-	-
6	sDP4+ (C data)		75.36%	24.64%	-	-	-	-
7	sDP4+ (all data)		97.30%	2.70%	-	-	-	-
8	uDP4+ (H data)		90.33%	9.67%	-	-	-	-
9	uDP4+ (C data)		88.29%	11.71%	-	-	-	-
10	uDP4+ (all data)		98.60%	1.40%	-	-	-	-
11	DP4+ (H data)		99.10%	0.90%	-	-	-	-
12	DP4+ (C data)		95.84%	4.16%	-	-	-	-
13	DP4+ (all data)		99.96%	0.04%	-	-	-	-

Figure S8. The DP4+ results between calculated and experimental NMR data for **7**

3.3 Elucidation of absolute configurations by TDDFT-ECD

To determine absolute configurations of compounds **1–7**, the spin-allowed excitation energies and rotatory (Rn) and oscillator strengths (fn) of the lowest excited states of stable conformers were calculated for ECD spectra using TD-DFT method at the CAM-B3LYP/6-311G(d,p) level[7] with IEFPCM solvent model for methanol in agreement with the experiment condition. All the calculations in this article were performed using the Gaussian 09. Electronic transitions were expanded as Gaussian curves with a FQHM (full width at half maximum) for each peak of 0.40 eV. The ECD spectra were combined after Boltzmann weighting according to their population contribution.

In particular: About 1.9nm red shift to calculated ECD spectra of compound **1**.

About 2.8nm red shift to calculated ECD spectra of compound **2**.

About 10nm red shift to calculated ECD spectra of compound **5**.

4. Anti-inflammation assay of 1–7

Table S15. Effects of samples on the anti-inflammatory effects of zebrafish internodes.

Group	Concentration	Number of macrophages around nerve mound（mean ± SEM）	Inhibition rate
Control	—	4.25 ± 0.8609	—
$CuSO_4$ model	20 μM	31.89 ± 3.16	—
Indomethacin	40 μM	11.88 ± 1.575	62.75%
**1**	20 μM	25.67 ± 3.14	19.50%
**2**	20 μM	30.00 ± 3.047	5.93%
**3**	20 μM	25.30 ± 1.904	20.66%
**4**	20 μM	22.22 ± 2.737	30.32%
**5**	20 μM	21.89 ± 3.199	31.36%
**6**	20 μM	26.2 ± 2.356	17.84%
7	20 μM	18.6 ± 1.805	41.67%

5. Cytotoxic assay of 1–7

Table S16. Original data of cytotoxic assay for compounds 1-7

Compounds	Concentration (μM)	Inhibition rate (%)			
		K562	L-02	ASPC-1	MDA-MB-231
1	30	39.85	24.89	NT[a]	21.85
2	30	48.66	30.19	NT[a]	24.20
3	30	43.47	22.10	18.60	19.95
4	30	36.69	21.59	18.85	18.42
5	30	34.28	NT[a]	NT[a]	NT[a]
6	30	NT[a]	25.40	NT[a]	NT[a]
7	30	63.55	29.76	17.53	10.36

doxorubicin[b]	1	100	82.95	51.95	55.85

[a] NT: not tested. [b] Positive control.

Table S17. Original data of cytotoxic assay for compound 7

Compound	Concentration (μM)	Inhibition rate (%)
		K562
7	30	73.31
	15	21.04
	7.5	15.51
	3.75	11.6
	1.875	9.55
	0.9375	5.87
doxorubicin[b]	1	100

[b] Positive control.

6. Reference

1. Schrödinger Release 2019-1: MacroModel, Schrödinger, LLC, New York, NY, 2019.
2. Roos, K.; Wu, C.; Damm, W.; Reboul, M.; Stevenson, J. M.; Lu, C.; Dahlgren, M. K.; Mondal, S.; Chen, W.; Wang, L.; Abel, R.; Friesner, R. A.; Harder, E. D., OPLS3e: Extending Force Field Coverage for Drug-Like Small Molecules. *J Chem Theory Comput.* **2019**, *15*, 1863-1874.
3. Smith, S. G.; Goodman, J. M., Assigning Stereochemistry to Single Diastereoisomers by GIAO NMR Calculation: The DP4 Probability. *J. Am. Chem. Soc*. **2010**, *132*, 12946-12959.
4. Frisch, M., Trucks, G., Schlegel, H., Scuseria, G., Robb, M., Cheeseman, J., Scalmani, G., Barone, V., Petersson, G., Nakatsuji, H. Gaussian, Inc. Wallingford, CT, **2016.**
5. Su, L.-H.; Geng, C.-A.; Li, T.-Z.; Ma, Y.-B.; Huang, X.-Y.; Zhang, X.-M.; Chen, J.-J., Artatrovirenols A and B: Two Cagelike Sesquiterpenoids from *Artemisia atrovirens*. *J. Org. Chem.* **2020**, *85*, 13466-13471.
6. Li, S.-W.; Cuadrado, C.; Yao, L.-G.; Daranas, A. H.; Guo, Y.-W., Quantum Mechanical-NMR-Aided Configuration and Conformation of Two Unreported Macrocycles Isolated from the Soft Coral *Lobophytum sp.*: Energy Calculations versus Coupling Constants. *Org. Lett.* **2020**, *22*, 4093-4096.
7. Suramitr, S.; Piriyagagoon, A.; Wolschann, P.; Hannongbua, S., Theoretical study on the structures and electronic properties of oligo(p-phenylenevinylene) carboxylic acid and its derivatives: effects of spacer and anchor groups. *Theor. Chem. Acc.* **2012**, *131*, 1-15.

7. Computational Details

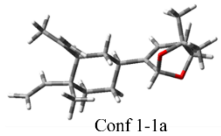

Conf 1-1a
31.20%

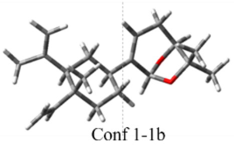

Conf 1-1b
36.54 %

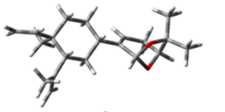

Conf 1-1c
21.05 %

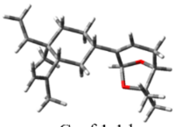

Conf 1-1d
4.21%

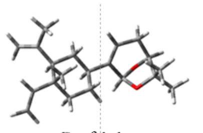

Conf 1-1e
3.20%

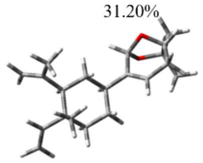

Conf 1-1f
3.172 %

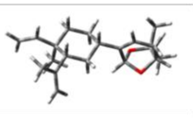

Conf 1-2a
21.79%

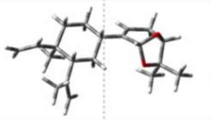

Conf 1-2b
44.81%

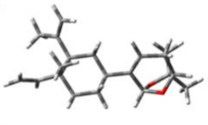

Conf 1-2c
20.68%

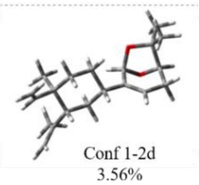

Conf 1-2d
3.56%

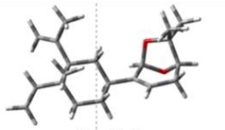

Conf 1-2e
6.16%

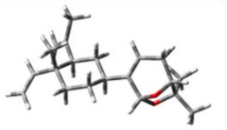

Conf 1-2f
3.00 %

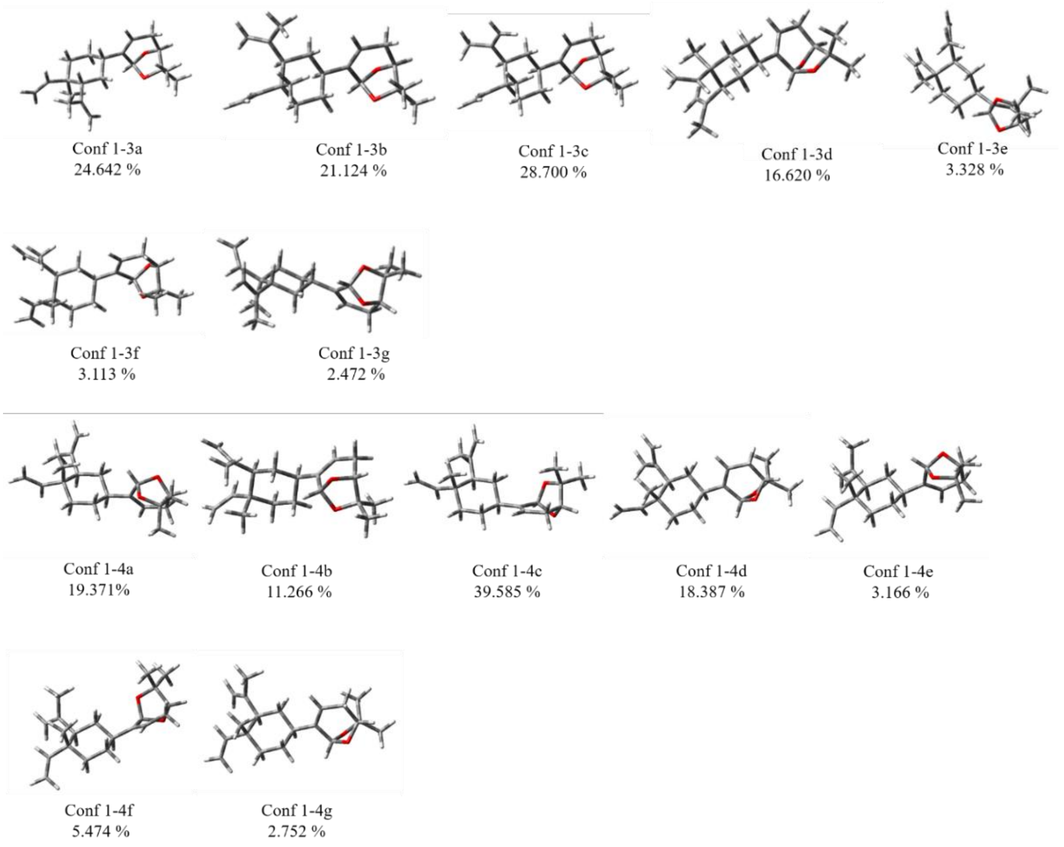

Figure S9. Stable conformers of compound **1** for relative configurations

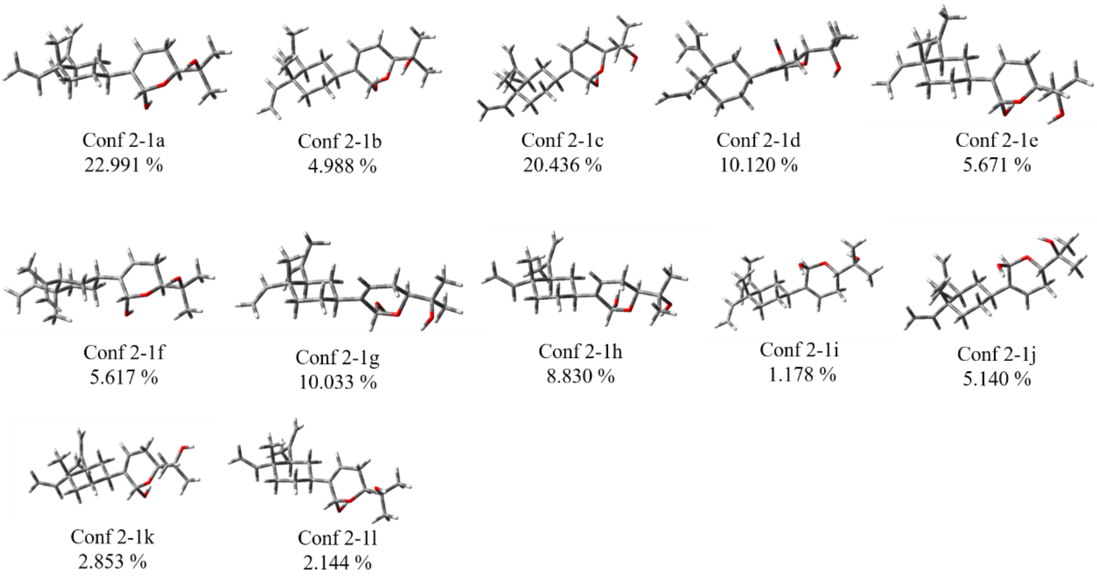

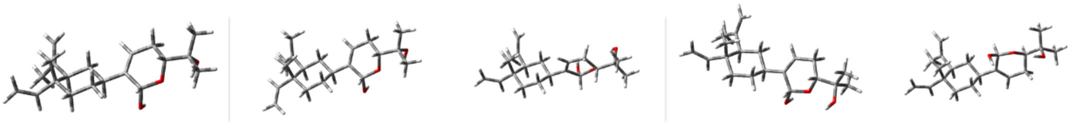

Conf 2-2a
16.352 %

Conf 2-2b
3.243 %

Conf 2-2c
4.640 %

Conf 2-2d
15.210 %

Conf 2-2e
2.383 %

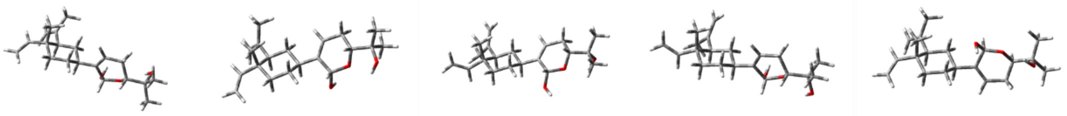

Conf 2-2f
0.657 %

Conf 2-2g
4.172 %

Conf 2-2h
30.723 %

Conf 2-2i
3.949 %

Conf 2-2j
0.382 %

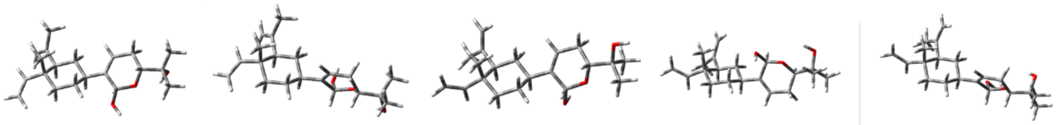

Conf 2-2k
5.768 %

Conf 2-2l
0.832 %

Conf 2-2m
1.663 %

Conf 2-2n
1.839 %

Conf 2-2o
8.185 %

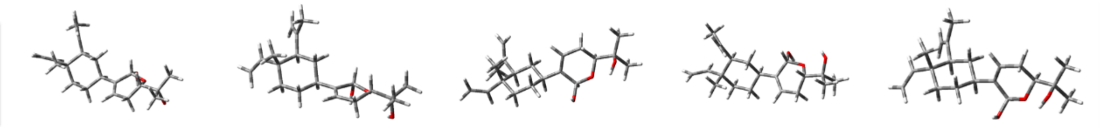

Conf 2-3a
14.300 %

Conf 2-3b
3.398 %

Conf 2-3c
4.239 %

Conf 2-3d
21.112 %

Conf 2-3e
0.452 %

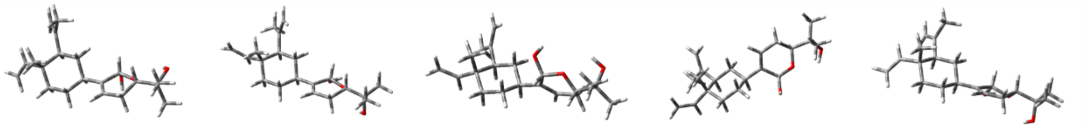

Conf 2-3f
4.235 %

Conf 2-3g
22.924 %

Conf 2-3h
1.992 %

Conf 2-3i
5.671 %

Conf 2-3j
4.891 %

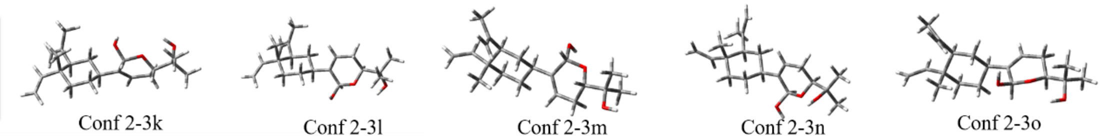

Conf 2-3k
0.223 %

Conf 2-3l
0.675 %

Conf 2-3m
2.549 %

Conf 2-3n
11.309 %

Conf 2-3o
2.031 %

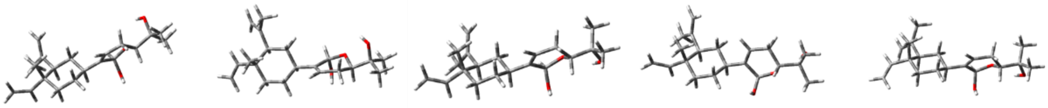

Conf 2-4a
23.618 %

Conf 2-4b
2.110 %

Conf 2-4c
21.495 %

Conf 2-4d
7.834 %

Conf 2-4e
2.957 %

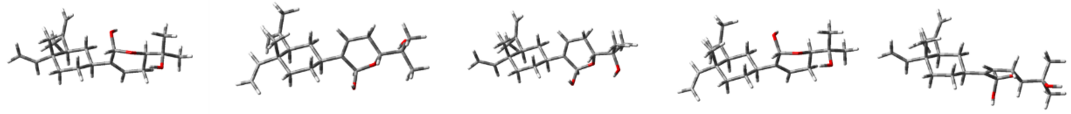

Conf 2-4f
3.805 %

Conf 2-4g
2.330 %

Conf 2-4h
13.609 %

Conf 2-4i
1.604 %

Conf 2-4j
1.606 %

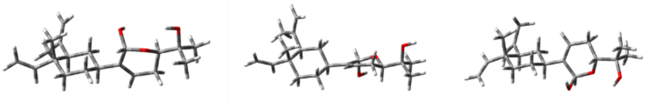

Conf 2-4k
5.718 %

Conf 2-4l
10.370 %

Conf 2-4m
2.945 %

Figure S10. Stable conformers of compound **2** for relative configurations

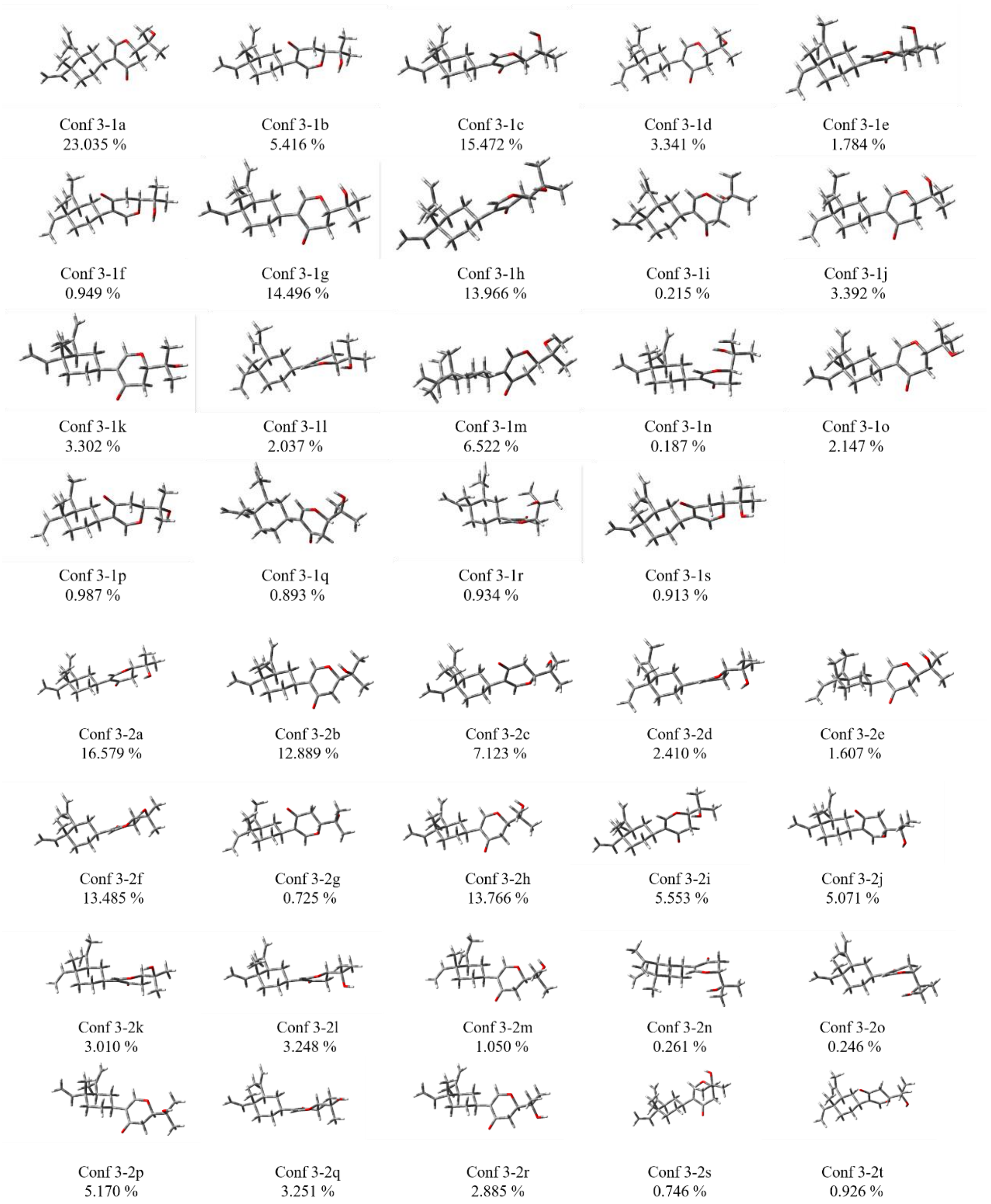

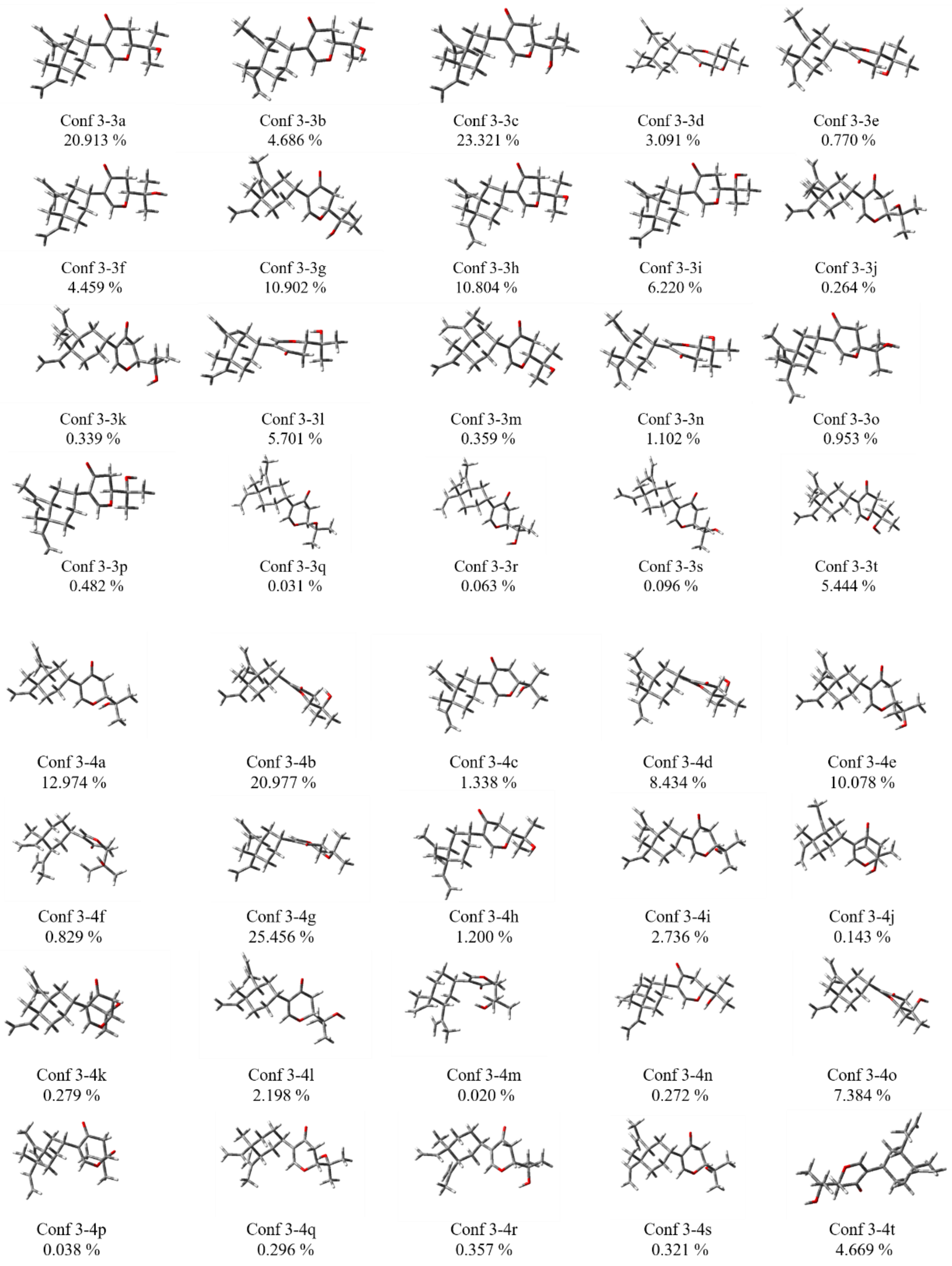

Figure S11. Stable conformers of compound **3** for relative configurations

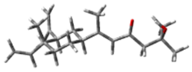
Conf 4-1a
7.858 %

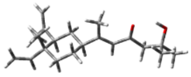
Conf 4-1b
12.060 %

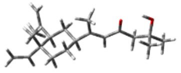
Conf 4-1c
10.268 %

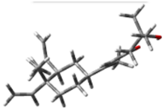
Conf 4-1d
4.076 %

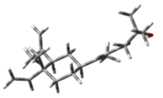
Conf 4-1e
4.095 %

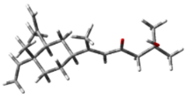
Conf 4-1f
6.227 %

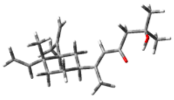
Conf 4-1g
16.101 %

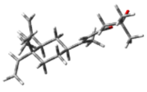
Conf 4-1h
3.525 %

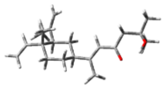
Conf 4-1i
3.858 %

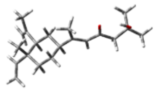
Conf 4-1j
2.590 %

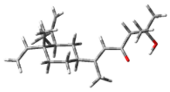
Conf 4-1k
4.023 %

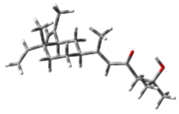
Conf 4-1l
2.444 %

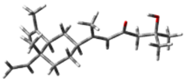
Conf 4-1m
3.860 %

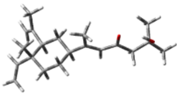
Conf 4-1n
2.345 %

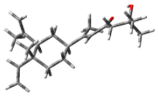
Conf 4-1o
3.413 %

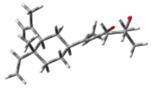
Conf 4-1p
3.173 %

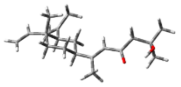
Conf 4-1q
2.316 %

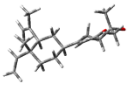
Conf 4-1r
2.630 %

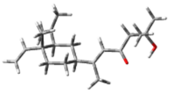
Conf 4-1s
2.559 %

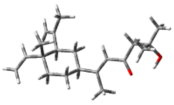
Conf 4-1t
2.579 %

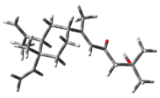
Conf 4-2a
10.375 %

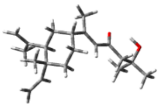
Conf 4-2b
24.570 %

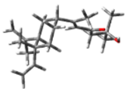
Conf 4-2c
4.665 %

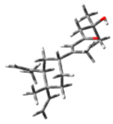
Conf 4-2d
7.016 %

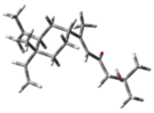
Conf 4-2e
0.786 %

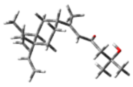
Conf 4-2f
1.421 %

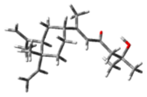
Conf 4-2g
24.751 %

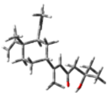
Conf 4-2h
1.088 %

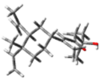
Conf 4-2i
0.781 %

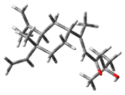
Conf 4-2j
1.416 %

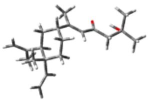
Conf 4-2k
10.370 %

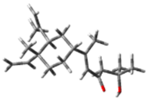
Conf 4-2l
0.062 %

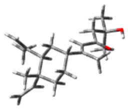
Conf 4-2m
7.009 %

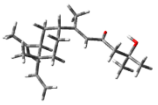
Conf 4-2n
1.923 %

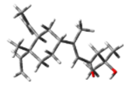
Conf 4-2o
0.022 %

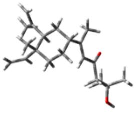
Conf 4-2p
0.615 %

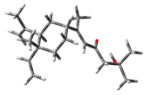
Conf 4-2q
0.800 %

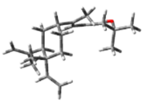
Conf 4-2r
0.107 %

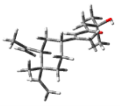
Conf 4-2s
0.785 %

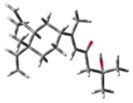
Conf 4-2t
1.439 %

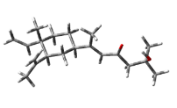
Conf 4-3a
9.172 %

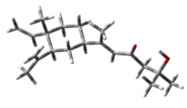
Conf 4-3b
6.296 %

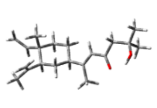
Conf 4-3c
2.100 %

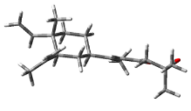
Conf 4-3d
13.127 %

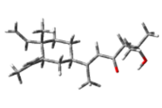
Conf 4-3e
3.189 %

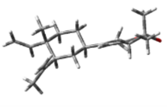
Conf 4-3f
9.332 %

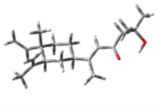
Conf 4-3g
3.196 %

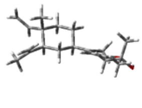
Conf 4-3h
9.322 %

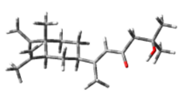
Conf 4-3i
10.518 %

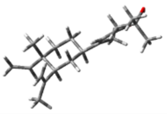
Conf 4-3j
13.066 %

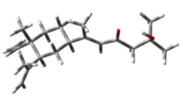
Conf 4-3k
1.958 %

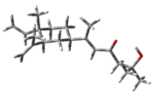
Conf 4-3l
4.512 %

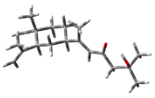
Conf 4-3m
3.937 %

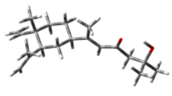
Conf 4-3n
1.649 %

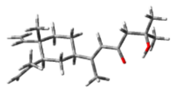
Conf 4-3o
2.481 %

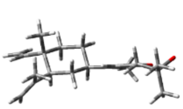
Conf 4-3p
1.787 %

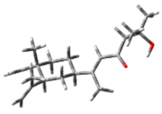
Conf 4-3q
2.237 %

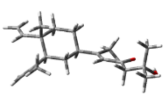
Conf 4-3r
2.119 %

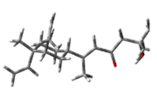
Conf 4-4a
4.214 %

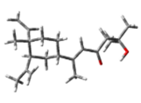
Conf 4-4b
5.498 %

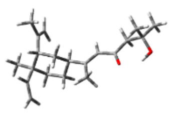
Conf 4-4c
12.946 %

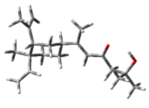
Conf 4-4d
5.687 %

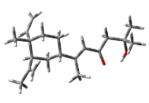
Conf 4-4e
16.014 %

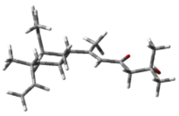
Conf 4-4f
0.811 %

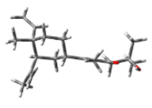
Conf 4-4g
5.891 %

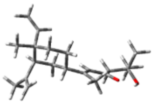
Conf 4-4h
5.745 %

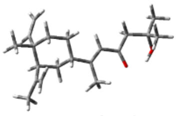
Conf 4-4i
23.109 %

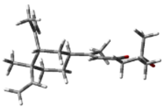
Conf 4-4j
2.749 %

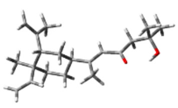
Conf 4-4k
4.277 %

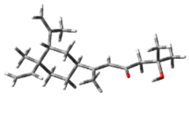
Conf 4-4l
3.573 %

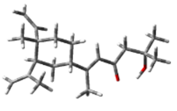
Conf 4-4m
1.362 %

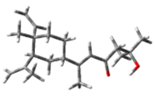
Conf 4-4n
1.627 %

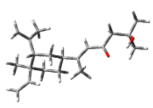
Conf 4-4o
1.720 %

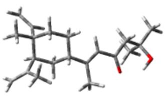
Conf 4-4p
1.622 %

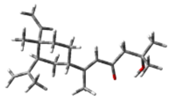
Conf 4-4q
1.366 %

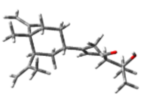
Conf 4-4r
1.788 %

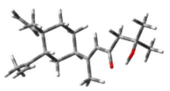
Conf 4-5a
8.359 %

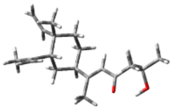
Conf 4-5b
2.959 %

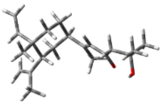
Conf 4-5c
8.983 %

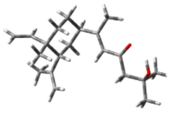
Conf 4-5d
5.718 %

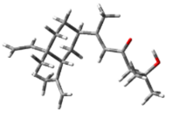
Conf 4-5e
5.971 %

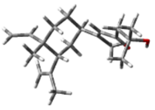
Conf 4-5f
5.725 %

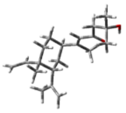
Conf 4-5g
19.343 %

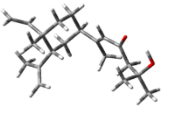
Conf 4-5h
0.202 %

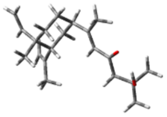
Conf 4-5i
2.281 %

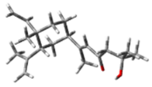
Conf 4-5j
8.372 %

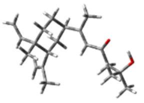
Conf 4-5k
4.547 %

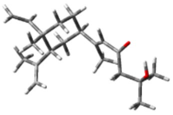
Conf 4-5l
0.049 %

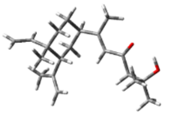
Conf 4-5m
5.911 %

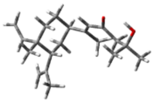
Conf 4-5n
0.451 %

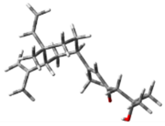
Conf 4-5o
8.920 %

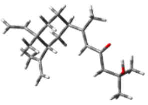
Conf 4-5p
5.995 %

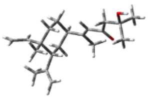
Conf 4-5q
0.765 %

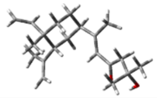
Conf 4-5r
0.963 %

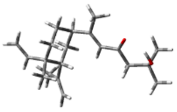
Conf 4-5s
1.540 %

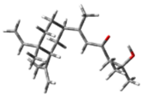
Conf 4-5t
2.945 %

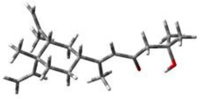
Conf 4-6a
6.884 %

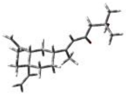
Conf 4-6b
4.358 %

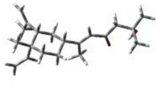
Conf 4-6c
4.356 %

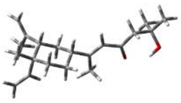
Conf 4-6d
7.049 %

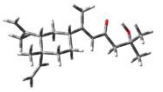
Conf 4-6e
7.535 %

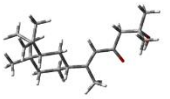
Conf 4-6f
2.880 %

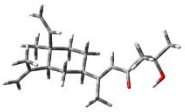
Conf 4-6g
3.758 %

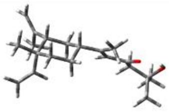
Conf 4-6h
6.053 %

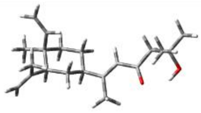
Conf 4-6i
3.751 %

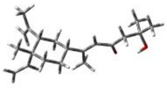
Conf 4-6j
5.639 %

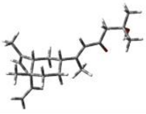
Conf 4-6k
5.413 %

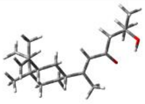
Conf 4-6l
6.759 %

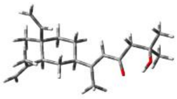
Conf 4-6m
7.514 %

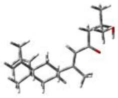
Conf 4-6n
2.875 %

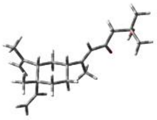
Conf 4-6o
5.414 %

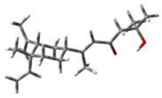
Conf 4-6p
7.224 %

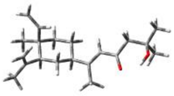
Conf 4-6q
2.907 %

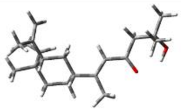
Conf 4-6r
3.212 %

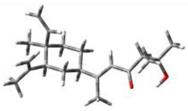
Conf 4-6s
2.730 %

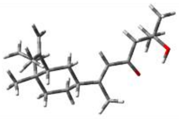
Conf 4-6t
3.691 %

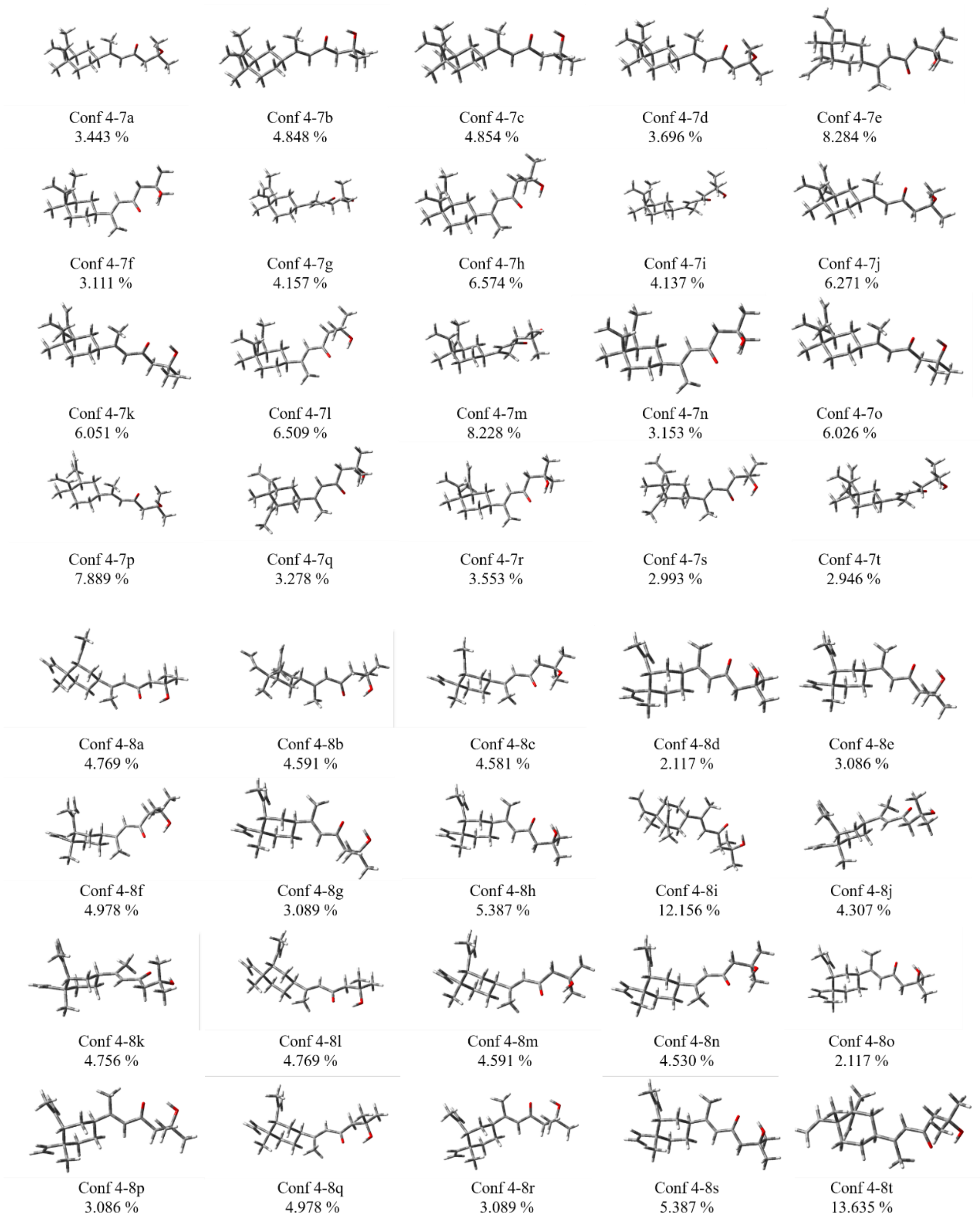

Figure S12. Stable conformers of compound **4** for relative configurations

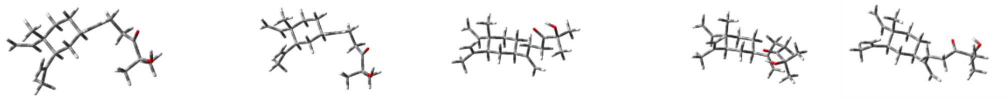

Conf 5-1a 0.993 % | Conf 5-1b 11.372 % | Conf 5-1c 17.348 % | Conf 5-1d 3.751 % | Conf 5-1e 7.122 %

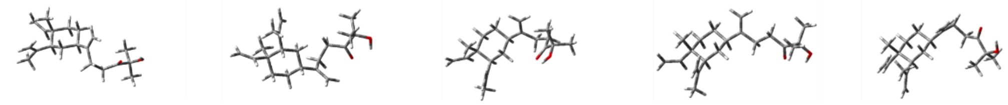

Conf 5-1f 4.126 % | Conf 5-1g 0.238 % | Conf 5-1h 3.260 % | Conf 5-1i 7.744 % | Conf 5-1j 0.167 %

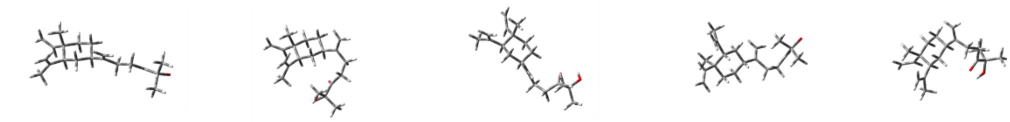

Conf 5-1k 8.195 % | Conf 5-1l 0.036 % | Conf 5-1m 2.910 % | Conf 5-1n 2.343 % | Conf 5-1o 0.170 %

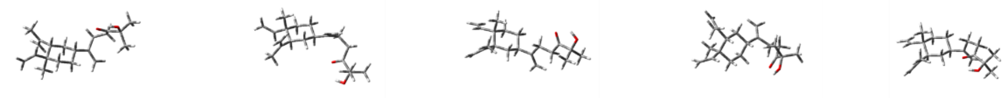

Conf 5-1p 13.088 % | Conf 5-1q 12.300 % | Conf 5-1r 0.518 % | Conf 5-1s 3.770 % | Conf 5-1t 0.548 %

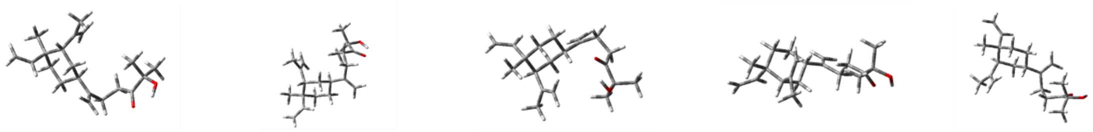

Conf 5-2a 20.487 % | Conf 5-2b 7.282 % | Conf 5-2c 2.079 % | Conf 5-2d 18.393 % | Conf 5-2e 7.282 %

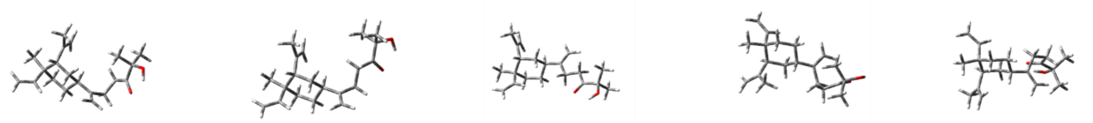

Conf 5-2f 20.097 % | Conf 5-2g 2.988 % | Conf 5-2h 10.847 % | Conf 5-2i 1.516 % | Conf 5-2j 9.030 %

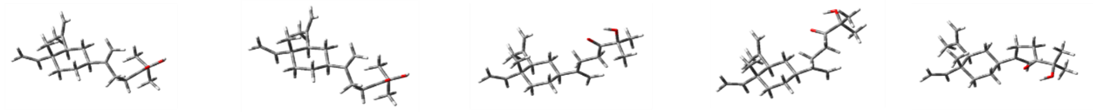

Conf 5-3a 1.103 % | Conf 5-3b 11.851 % | Conf 5-3c 15.486 % | Conf 5-3d 4.145 % | Conf 5-3e 6.463 %

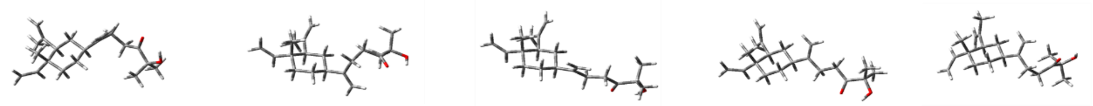

Conf 5-3f 4.382 % | Conf 5-3g 0.267 % | Conf 5-3h 8.418 % | Conf 5-3i 4.486 % | Conf 5-3j 0.178 %

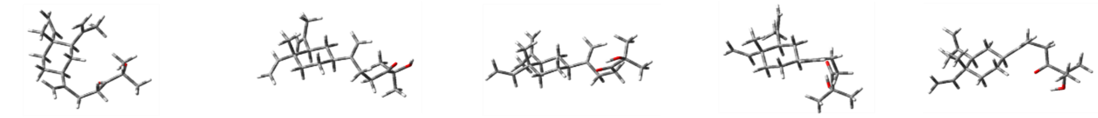

Conf 5-3k 0.041 % | Conf 5-3l 2.416 % | Conf 5-3m 3.167 % | Conf 5-3n 0.158 % | Conf 5-3o 13.532 %

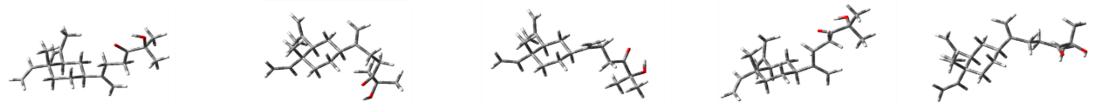

Conf 5-3p 2.317 % | Conf 5-3q 4.045 % | Conf 5-3r 4.912 % | Conf 5-3s 0.643 % | Conf 5-3t 11.991 %

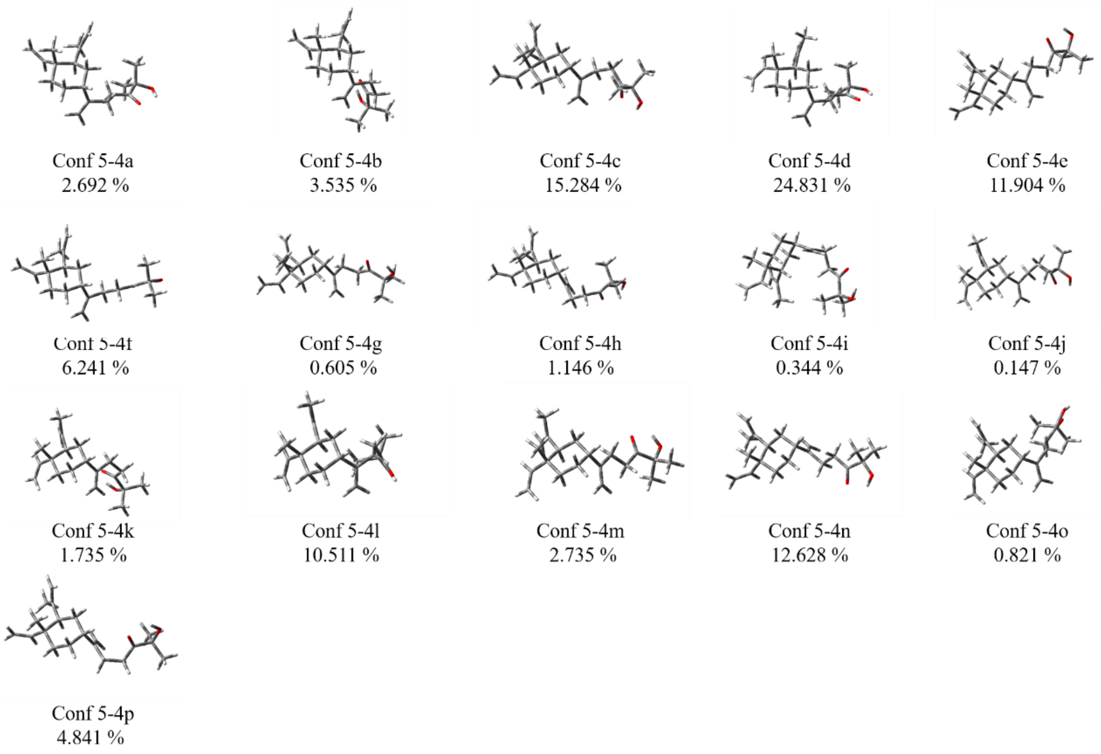

Figure S13. Stable conformers of compound **5** for relative configurations

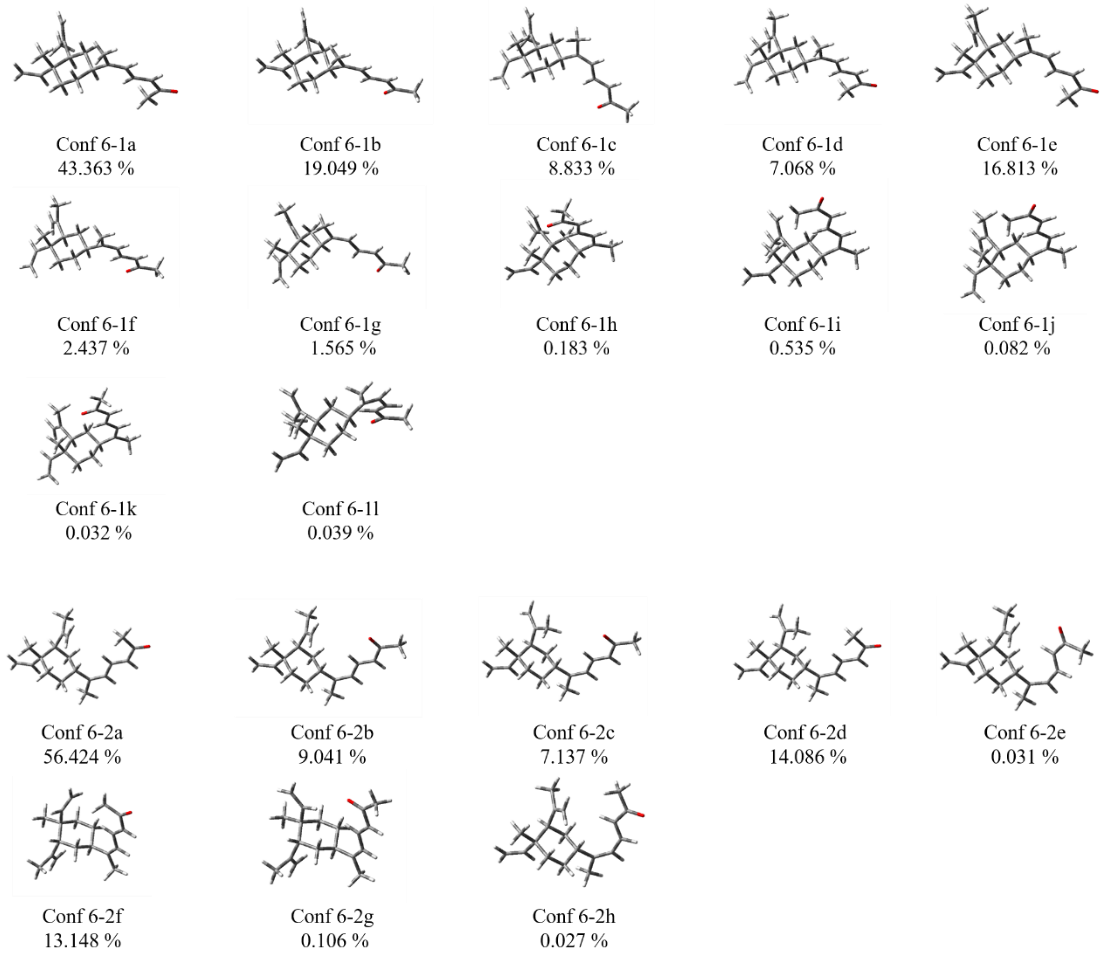

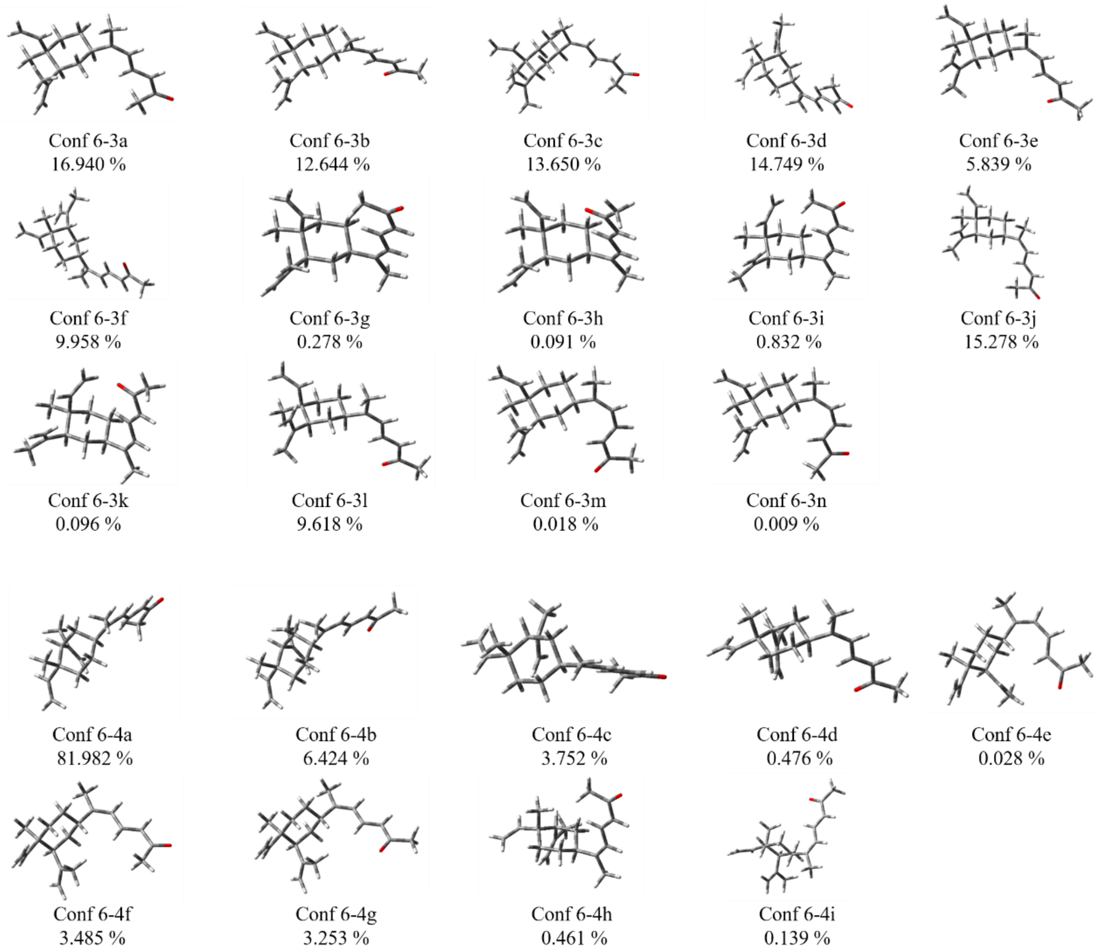

Figure S14. Stable conformers of compound **6** for relative configurations

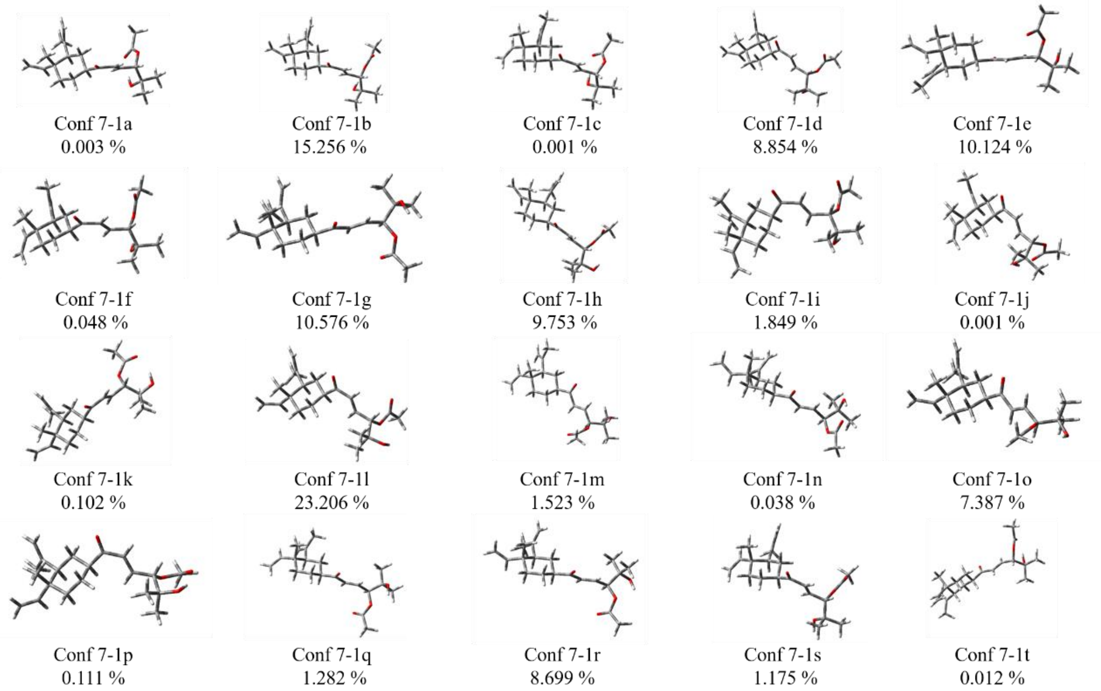

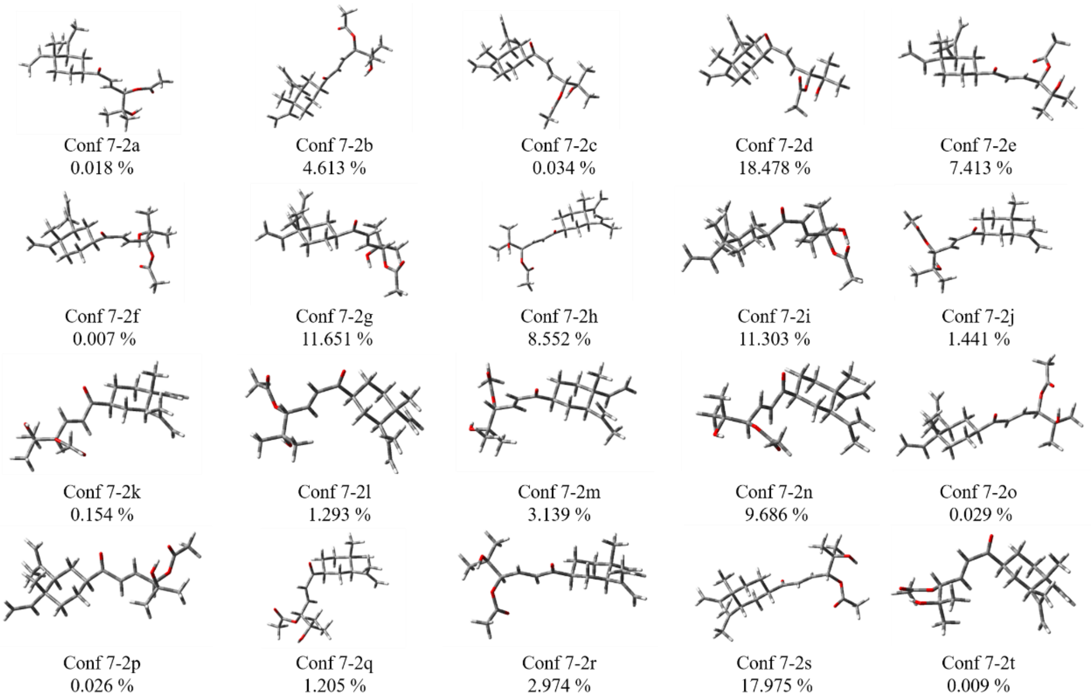

Figure S15. Stable conformers of compound **7** for relative configurations

Table S18. Optimized Z-Matrixes of compounds **1-7** in the Gas Phase (Å) at B3LYP/6-31G (d, p) level

atom	Con f. 1- **1**a			atom	Con f. 1- **1**b		
C	5.491	-0.6559	-0.63896	C	-5.16533	-1.95681	0.15293
C	2.4507	2.64178	-0.57501	C	-3.63345	2.11235	1.23791
C	2.83988	1.80795	0.39701	C	-3.66557	1.4902	0.05327
C	2.24848	0.41615	0.57994	C	-2.58341	0.52091	-0.40383
C	2.95772	-0.6907	-0.28891	C	-2.81105	-0.9591	0.08958
C	4.40867	-0.78126	0.13054	C	-4.10797	-1.47051	-0.49875
C	0.72208	0.39665	0.38692	C	-1.16688	1.02145	-0.069
C	0.10717	-0.97556	0.72383	C	-0.08219	0.13285	-0.6832
C	0.78727	-2.08413	-0.10117	C	-0.26601	-1.32284	-0.20547
C	2.30686	-2.05412	0.0766	C	-1.67545	-1.83133	-0.51429
C	2.82071	-0.42682	-1.79248	C	-2.80284	-1.07134	1.61835
C	3.88518	2.2221	1.40027	C	-4.78185	1.74676	-0.9261
C	-1.39009	-0.96632	0.55121	C	1.32539	0.61167	-0.43125
C	-2.26042	-1.24527	1.52869	C	1.7017	1.55846	0.43669
C	-3.74965	-1.16416	1.30819	C	3.15582	1.90348	0.64143
C	-4.05654	-0.56997	-0.06752	C	4.05815	0.94259	-0.135
O	-3.20173	-1.2209	-1.03536	O	3.5095	0.79287	-1.46473
C	-1.97015	-0.5581	-0.79694	C	2.43029	-0.09178	-1.20942
C	-3.67222	0.92631	-0.28262	C	4.08078	-0.53937	0.3516
O	-2.30182	0.83266	-0.79652	O	3.00644	-1.14873	-0.43903
C	-4.52062	1.53654	-1.39551	C	5.3808	-1.21824	-0.07229
C	-3.6785	1.79812	0.96601	C	3.79394	-0.75351	1.83162
H	2.43706	0.12559	1.62305	H	-2.64782	0.47095	-1.49985
H	0.31463	-1.1817	1.78301	H	-0.23141	0.12934	-1.7754
H	6.48861	-0.76026	-0.21995	H	-6.03985	-2.31025	-0.38742
H	5.43481	-0.44505	-1.70277	H	-5.20869	-2.02232	1.23598
H	2.8909	3.63093	-0.67623	H	-4.42088	2.80249	1.53125
H	1.68612	2.37801	-1.29885	H	-2.83181	1.9627	1.95419
H	4.54956	-0.99391	1.19162	H	-4.14218	-1.43758	-1.58909
H	0.46772	0.6714	-0.64111	H	-1.02809	1.06541	1.0158
H	0.27285	1.1631	1.02773	H	-1.06058	2.04744	-0.43789
H	0.40008	-3.06046	0.21242	H	0.46829	-1.97883	-0.68425
H	0.53118	-1.97373	-1.1611	H	-0.06378	-1.36778	0.87141

H	2.54314	-2.28121	1.12477	H	-1.80404	-1.86471	-1.60436
H	2.77339	-2.84084	-0.52793	H	-1.79323	-2.86009	-0.15363
H	3.31893	0.50094	-2.08247	H	-3.63024	-0.5157	2.06507
H	3.26918	-1.24672	-2.36281	H	-2.89438	-2.1198	1.92027
H	1.77504	-0.34911	-2.09726	H	-1.87586	-0.68639	2.04854
H	4.25659	3.23246	1.20473	H	-5.30519	0.81935	-1.1827
H	3.47414	2.19681	2.41808	H	-5.51489	2.45574	-0.52967
H	4.738	1.53482	1.39018	H	-4.38391	2.15207	-1.86578
H	-1.90598	-1.54104	2.51462	H	0.97091	2.11507	1.01703
H	-4.19703	-2.16585	1.37282	H	3.34989	2.93001	0.30071
H	-4.22661	-0.57013	2.09547	H	3.40986	1.8875	1.70709
H	-5.09135	-0.75585	-0.35967	H	5.06954	1.33987	-0.23489
H	-1.30061	-0.7576	-1.63529	H	2.07775	-0.49958	-2.16026
H	-4.53571	0.87558	-2.26722	H	6.22167	-0.85538	0.52757
H	-5.54971	1.69442	-1.05683	H	5.30228	-2.30117	0.06461
H	-4.10589	2.50276	-1.69878	H	5.58865	-1.01558	-1.12719
H	-3.36874	2.81503	0.70564	H	4.55845	-0.26905	2.44726
H	-4.68453	1.85195	1.394	H	2.81496	-0.35798	2.10908
H	-2.99254	1.4158	1.72457	H	3.80788	-1.82424	2.05784

atom	Con f. 1- **1**c			atom	Con f. 1- **1**d		
C	-5.51903	-1.28435	-0.59021	C	-5.26029	-1.49952	-0.31399
C	-3.39579	2.19184	1.34891	C	-3.72248	2.35126	-1.3405
C	-3.33888	1.71951	0.09781	C	-2.78236	1.92709	-0.48718
C	-2.41952	0.57761	-0.31491	C	-2.28272	0.48788	-0.54503
C	-3.0469	-0.85083	-0.09728	C	-3.01731	-0.51386	0.4289
C	-4.27711	-0.97907	-0.96937	C	-4.50181	-0.4937	0.12508
C	-1.02233	0.68927	0.32295	C	-0.75302	0.42108	-0.38375
C	-0.04749	-0.37864	-0.21536	C	-0.19782	-1.00039	-0.58887
C	-0.63385	-1.7755	0.00925	C	-0.90188	-1.98061	0.3668
C	-2.02229	-1.89529	-0.62289	C	-2.4198	-1.91678	0.18977
C	-3.36978	-1.12382	1.37631	C	-2.86414	-0.11881	1.91358
C	-4.1761	2.32767	-0.99792	C	-2.16575	2.87963	0.50833
C	1.33471	-0.18248	0.35534	C	1.30254	-1.03213	-0.45147
C	1.91388	-0.92094	1.30916	C	2.13579	-1.44037	-1.4157
C	3.32432	-0.64367	1.76751	C	3.63221	-1.39267	-1.2397
C	3.98346	0.41469	0.8819	C	3.9954	-0.67537	0.06179
O	3.04409	1.5017	0.71144	O	3.14601	-1.19536	1.1102
C	2.16274	0.94367	-0.24888	C	1.93166	-0.51518	0.83589
C	4.25223	0.01219	-0.60076	C	3.66705	0.84821	0.13343
O	3.01798	0.43199	-1.27354	O	2.31013	0.85606	0.69122
C	5.38217	0.85712	-1.18388	C	4.56883	1.53661	1.15483
C	4.47819	-1.47301	-0.85131	C	3.66245	1.58755	-1.19777
H	-2.27339	0.66496	-1.40064	H	-2.5184	0.11496	-1.54974
H	0.03452	-0.22306	-1.30336	H	-0.44076	-1.31047	-1.61461
H	-6.32115	-1.3647	-1.31947	H	-6.32516	-1.35864	-0.48264
H	-5.78988	-1.46385	0.44594	H	-4.86956	-2.49106	-0.52228
H	-4.0598	3.01267	1.60927	H	-4.09196	3.37398	-1.32068
H	-2.79072	1.7867	2.15395	H	-4.15641	1.689	-2.08544
H	-4.08598	-0.81339	-2.03107	H	-4.98064	0.46684	0.30863
H	-1.08028	0.58606	1.41144	H	-0.4635	0.78565	0.60685
H	-0.63194	1.6939	0.12843	H	-0.28697	1.09939	-1.10763
H	0.03349	-2.53298	-0.41801	H	-0.55012	-2.99985	0.17033
H	-0.69513	-1.98258	1.08356	H	-0.63348	-1.75114	1.40466
H	-1.92628	-1.77193	-1.70983	H	-2.66138	-2.23296	-0.83292
H	-2.42901	-2.89962	-0.45624	H	-2.90517	-2.63232	0.86357
H	-4.14117	-0.44584	1.74815	H	-3.42376	0.79255	2.14098
H	-3.72918	-2.15033	1.50177	H	-3.26218	-0.9156	2.55137
H	-2.49082	-1.00309	2.01273	H	-1.82201	0.0475	2.19728
H	-4.8326	1.58046	-1.45683	H	-2.2002	2.49372	1.53126
H	-4.79745	3.14742	-0.62519	H	-1.10812	3.05757	0.28006
H	-3.53649	2.71773	-1.80059	H	-2.67805	3.84573	0.49045
H	1.38654	-1.74493	1.78213	H	1.74434	-1.81904	-2.35833
H	3.32067	-0.28741	2.80709	H	4.04367	-2.4113	-1.2139
H	3.91841	-1.56422	1.76712	H	4.10693	-0.89661	-2.09349
H	4.88206	0.82152	1.34854	H	5.03096	-0.86794	0.34686
H	1.55048	1.74321	-0.67212	H	1.27766	-0.60757	1.70467
H	5.41612	0.74103	-2.27154	H	4.59182	0.96453	2.08719
H	5.2243	1.91468	-0.95212	H	5.5907	1.62598	0.77222
H	6.34935	0.54811	-0.77433	H	4.19349	2.54126	1.37255
H	4.63712	-1.64628	-1.92021	H	3.40146	2.63782	-1.03453
H	5.36717	-1.82358	-0.3175	H	4.65347	1.55473	-1.66133
H	3.61874	-2.06602	-0.53241	H	2.93488	1.15813	-1.88929

atom	Con f. 1- **1**e			atom	Con f. 1- **1**f		
C	-4.53802	-2.56613	-0.60202	C	-4.74928	-2.10706	-1.2882
C	-4.75738	1.66926	-0.92755	C	-4.12012	2.14061	-1.33334
C	-3.67871	1.54151	-0.14515	C	-3.3436	1.7733	-0.30654
C	-2.64106	0.46296	-0.43343	C	-2.47006	0.52932	-0.41138
C	-2.85112	-0.89144	0.34999	C	-3.10965	-0.79043	0.17153
C	-4.2305	-1.4328	0.03103	C	-4.42316	-1.05394	-0.53686
C	-1.2086	1.00423	-0.26451	C	-1.06149	0.77913	0.15998
C	-0.14511	-0.00211	-0.70816	C	-0.10902	-0.40799	-0.08499
C	-0.32937	-1.32738	0.06062	C	-0.71557	-1.68108	0.50993
C	-1.7495	-1.86742	-0.11262	C	-2.10733	-1.93935	-0.07111
C	-2.79033	-0.71061	1.88142	C	-3.434	-0.68047	1.67716
C	-3.44599	2.49662	1.00045	C	-3.28066	2.61479	0.94524
C	1.26966	0.50588	-0.58452	C	1.2841	-0.08932	0.39775
C	1.66051	1.6313	0.02586	C	1.87473	-0.57279	1.497
C	3.11903	2.00023	0.13615	C	3.29395	-0.20384	1.85162
C	4.00651	0.86552	-0.37857	C	3.94398	0.60011	0.72422
O	3.4525	0.40145	-1.63119	O	3.00554	1.61649	0.3011
C	2.363	-0.38	-1.16816	C	2.10922	0.84276	-0.47919
C	4.00935	-0.45142	0.45806	C	4.19248	-0.15733	-0.61598
O	2.92762	-1.22329	-0.16162	O	2.94798	0.08508	-1.35393
C	5.30033	-1.23007	0.2189	C	5.3126	0.51467	-1.40632
C	3.71956	-0.29124	1.94425	C	4.41639	-1.65876	-0.49501
H	-2.76106	0.18882	-1.48909	H	-2.34806	0.32423	-1.48256
H	-0.31697	-0.22759	-1.77349	H	-0.04092	-0.55101	-1.17575
H	-5.57589	-2.84921	-0.75994	H	-5.73835	-2.18662	-1.73277
H	-3.7905	-3.25421	-0.98541	H	-4.06506	-2.92524	-1.49281
H	-5.50987	2.43268	-0.74345	H	-4.74931	3.02645	-1.28566
H	-4.92568	1.01247	-1.7773	H	-4.15476	1.56521	-2.25504
H	-5.047	-0.80434	0.38303	H	-5.17431	-0.28095	-0.38271
H	-1.03139	1.27546	0.78144	H	-1.10771	0.9675	1.23805
H	-1.10903	1.92771	-0.84648	H	-0.6561	1.68949	-0.2961
H	0.38804	-2.07588	-0.29095	H	-0.06329	-2.53746	0.30374
H	-0.10244	-1.15585	1.11979	H	-0.77593	-1.58822	1.60047
H	-1.90232	-2.09328	-1.17546	H	-2.00661	-2.09646	-1.15243
H	-1.85758	-2.81409	0.42941	H	-2.51959	-2.8662	0.34416
H	-3.65181	-0.14683	2.24949	H	-4.26572	0.00642	1.856
H	-2.80814	-1.6911	2.36982	H	-3.73144	-1.66147	2.06359
H	-1.88471	-0.19283	2.20636	H	-2.58003	-0.33461	2.26461
H	-4.30893	3.15359	1.14093	H	-3.46038	2.02924	1.85146
H	-3.25378	1.97797	1.94403	H	-2.29043	3.07104	1.06278
H	-2.57077	3.12951	0.81121	H	-4.01771	3.42193	0.90798
H	0.9383	2.32024	0.45478	H	1.35069	-1.24675	2.16929
H	3.32309	2.90694	-0.4503	H	3.30915	0.39571	2.77252
H	3.37762	2.24812	1.17153	H	3.88356	-1.10167	2.06768
H	5.0234	1.21106	-0.57156	H	4.84917	1.1046	1.06599
H	2.00322	-1.00322	-1.99083	H	1.49512	1.5164	-1.08104
H	5.20758	-2.24505	0.6174	H	6.28579	0.31737	-0.9452
H	5.51077	-1.29537	-0.85281	H	5.3329	0.13091	-2.43096
H	6.14576	-0.74158	0.71408	H	5.15475	1.59666	-1.44351
H	4.49028	0.31994	2.42442	H	5.31425	-1.86928	0.09466
H	3.71946	-1.27354	2.42695	H	3.56282	-2.15264	-0.02625
H	2.74576	0.17268	2.11279	H	4.55843	-2.09103	-1.49041

atom	Con f. 1- **2**a			atom	Con f. 1- **2**b		
C	-5.1827	-1.87005	0.07777	C	-5.56377	-0.60704	0.33146
C	-3.37381	1.95957	1.59887	C	-2.40213	2.52608	0.98783
C	-3.54239	1.51355	0.34829	C	-2.74931	1.86631	-0.12362
C	-2.54949	0.58797	-0.34262	C	-2.20149	0.48891	-0.47332
C	-2.80381	-0.94002	-0.05278	C	-3.01302	-0.69715	0.17094
C	-4.16899	-1.31443	-0.58783	C	-4.42757	-0.66588	-0.36494
C	-1.08798	0.98544	-0.07042	C	-0.69412	0.3707	-0.18775
C	-0.08991	0.14357	-0.88855	C	-0.10734	-0.9717	-0.6774
C	-0.31423	-1.35635	-0.61918	C	-0.87731	-2.1379	-0.0527
C	-1.76859	-1.74964	-0.8832	C	-2.37693	-2.02285	-0.33391
C	-2.66765	-1.28253	1.43506	C	-2.9895	-0.65176	1.70329
C	-4.73465	1.93773	-0.47007	C	-3.69501	2.47848	-1.12456
C	1.33511	0.56057	-0.6333	C	1.38293	-1.0147	-0.44839
C	2.13159	1.11507	-1.5544	C	2.0346	-1.83386	0.38575
C	3.56248	1.47624	-1.24782	C	3.5275	-1.73578	0.57747
C	3.95954	0.97081	0.14146	C	4.09124	-0.52203	-0.16296
O	2.89465	1.3036	1.06172	O	3.51105	-0.50083	-1.48762
C	1.93892	0.30501	0.741	C	2.21876	0.01075	-1.20447
C	4.04071	-0.57566	0.32243	C	3.66926	0.88005	0.37623

O	2.67786	-0.9191	0.74227	O	2.45913	1.16761	-0.40096
C	4.96364	-0.9257	1.48703	C	4.70462	1.93261	-0.01095
C	4.39222	-1.36983	-0.92818	C	3.33361	0.94419	1.8603
H	-2.708	0.70352	-1.42398	H	-2.3249	0.36528	-1.55846
H	-0.30008	0.32434	-1.95182	H	-0.27002	-1.00991	-1.76717
H	-6.1154	-2.11118	-0.42587	H	-6.52724	-0.60817	-0.17195
H	-5.13193	-2.10441	1.1369	H	-5.58643	-0.55367	1.4158
H	-4.10144	2.62248	2.06093	H	-2.80758	3.51108	1.20629
H	-2.51519	1.68898	2.20531	H	-1.70719	2.11732	1.7146
H	-4.29859	-1.10932	-1.65191	H	-4.48972	-0.71765	-1.45334
H	-0.86228	0.89078	0.99614	H	-0.49064	0.46863	0.88389
H	-0.95875	2.04414	-0.32047	H	-0.18378	1.20821	-0.67291
H	0.35073	-1.94745	-1.25928	H	-0.50219	-3.08872	-0.44873
H	-0.03878	-1.59503	0.41446	H	-0.69922	-2.15828	1.02865
H	-1.98514	-1.59828	-1.9492	H	-2.5398	-2.09626	-1.41736
H	-1.91485	-2.81738	-0.68191	H	-2.91238	-2.8643	0.12117
H	-1.68963	-0.99877	1.82904	H	-3.49065	0.24085	2.08425
H	-3.42557	-0.77328	2.03452	H	-3.49659	-1.52982	2.11625
H	-2.78221	-2.36084	1.58571	H	-1.96915	-0.64812	2.09157
H	-4.41128	2.4636	-1.37805	H	-4.57098	1.8405	-1.2841
H	-5.31652	1.07091	-0.80124	H	-4.04272	3.46483	-0.80315
H	-5.39752	2.60092	0.09361	H	-3.20467	2.58724	-2.10097
H	1.75524	1.32422	-2.55435	H	1.5092	-2.60052	0.94842
H	3.69142	2.56709	-1.28166	H	4.01634	-2.64435	0.19918
H	4.23777	1.06988	-2.00879	H	3.77655	-1.68189	1.64305
H	4.86958	1.45729	0.4962	H	5.17442	-0.59162	-0.27505
H	1.19582	0.26621	1.53951	H	1.7515	0.32283	-2.142
H	4.72241	-0.3121	2.36015	H	4.96067	1.8419	-1.07081
H	4.84401	-1.97903	1.75858	H	5.61738	1.81502	0.58205
H	6.01111	-0.75622	1.21728	H	4.30622	2.93645	0.16562
H	4.4052	-2.43892	-0.69431	H	3.02709	1.96122	2.12392
H	5.38584	-1.09298	-1.2945	H	2.51678	0.26524	2.11307
H	3.66382	-1.20306	-1.72425	H	4.20794	0.68791	2.46691

atom	Con f. 1- **2**c			atom	Con f. 1- **2**d		
C	-5.54736	-1.21848	-0.49383	C	4.65963	2.41225	-0.6644
C	-3.31537	2.35548	1.15387	C	4.67828	-1.85443	-0.6933
C	-3.27863	1.77001	0.09781	C	3.571	-1.61414	0.01946
C	-2.39172	0.5712	-0.31491	C	2.60683	-0.51235	-0.404
C	-3.06506	-0.81481	-0.09728	C	2.84187	0.8846	0.29141
C	-4.29661	-0.97872	-0.96937	C	4.26259	1.33568	0.01611
C	-0.99535	0.69377	0.32295	C	1.14113	-0.9732	-0.2848
C	-0.0554	-0.42903	-0.21536	C	0.15116	0.0607	-0.8516
C	-0.68076	-1.79934	0.00925	C	0.36569	1.42291	-0.16656
C	-2.07419	-1.93193	-0.62289	C	1.8196	1.8769	-0.30251
C	-3.39949	-0.95423	1.37631	C	2.68592	0.8212	1.82584
C	-4.1065	2.29904	-0.99792	C	3.23306	-2.46577	1.21923
C	1.34548	-0.31506	0.35534	C	-1.27897	-0.40413	-0.74999
C	1.73404	0.41289	1.30916	C	-2.05931	-0.66233	-1.80592
C	3.18205	0.47451	1.76751	C	-3.50471	-1.05802	-1.64318
C	4.07438	-0.22606	0.8819	C	-3.93068	-0.93901	-0.17855
O	3.46213	-1.49433	0.71144	O	-2.90242	-1.54772	0.63609
C	2.42886	-1.07061	-0.24888	C	-1.91415	-0.52985	0.62865
C	4.17209	0.43869	-0.60076	C	-3.97655	0.50224	0.41759
O	3.07468	-0.20329	-1.27354	O	-2.62429	0.66493	0.96231
C	5.46801	0.02599	-1.18388	C	-4.93879	0.55686	1.6015
C	3.97735	1.94896	-0.85131	C	-4.25035	1.62257	-0.57702
H	-2.24116	0.56252	-1.40064	H	2.79812	-0.32249	-1.46783
H	0.02955	-0.37216	-1.30336	H	0.37965	0.18712	-1.9188
H	-6.34946	-1.33582	-1.31947	H	5.71724	2.63508	-0.78292
H	-5.82588	-1.30556	0.44594	H	3.96825	3.11066	-1.12614
H	-3.95696	3.21306	1.60927	H	5.37917	-2.63712	-0.41242
H	-2.71429	2.01074	2.15395	H	4.9225	-1.27501	-1.5801
H	-4.09823	-0.90549	-2.03107	H	5.02651	0.69029	0.44676
H	-1.07739	0.68455	1.41144	H	0.89688	-1.18155	0.76197
H	-0.56693	1.66489	0.12843	H	1.02234	-1.9196	-0.82495
H	-0.0437	-2.61118	-0.41801	H	-0.29922	2.16877	-0.61666
H	-0.72742	-1.91076	1.08356	H	0.08491	1.35791	0.89087
H	-1.97532	-1.90696	-1.70983	H	2.03944	2.01396	-1.3688
H	-2.51008	-2.90535	-0.45624	H	1.95152	2.85501	0.17455
H	-2.51742	-0.81382	1.74815	H	3.4966	0.24859	2.28443
H	-4.14692	-0.22141	1.50177	H	2.72304	1.83351	2.24296
H	-3.79495	-1.95389	2.01273	H	1.73922	0.36881	2.13129
H	-4.70826	3.16181	-1.45683	H	4.05716	-3.14322	1.46038

H	-3.46108	2.60359	-0.62519	H	3.01135	-1.86734	2.10747
H	-4.78098	1.52994	-1.80059	H	2.34329	-3.07753	1.02788
H	1.01934	0.9843	1.78213	H	-1.66132	-0.58675	-2.81643
H	3.31327	-0.01046	2.80709	H	-3.65502	-2.09418	-1.97692
H	3.50116	1.51391	1.76712	H	-4.14891	-0.43996	-2.27832
H	5.06518	-0.43145	1.34854	H	-4.86354	-1.47299	0.00984
H	2.04461	-1.93954	-0.67212	H	-1.19256	-0.74034	1.41985
H	5.61094	-1.05617	-2.27154	H	-4.75304	-0.28009	2.28124
H	6.32857	0.52611	-0.95212	H	-5.97762	0.50812	1.25955
H	5.43203	0.30007	-0.77433	H	-4.80202	1.49107	2.15482
H	4.75648	2.45222	-1.92021	H	-3.49645	1.64849	-1.36627
H	4.04472	2.3052	-0.3175	H	-5.23739	1.50109	-1.03434
H	3.00121	2.23443	-0.53241	H	-4.23593	2.58597	-0.05794

atom	Con f. 1- **2**e			atom	Con f. 1- **2**f		
C	-5.27343	-1.35037	-0.6798	C	4.80433	-2.04976	1.20577
C	-3.57097	2.54771	-1.10196	C	4.02165	2.14867	1.45902
C	-2.7114	1.98903	-0.24118	C	3.26292	1.81154	0.40887
C	-2.24391	0.55254	-0.44242	C	2.43329	0.53389	0.43937
C	-3.07126	-0.53781	0.34227	C	3.12566	-0.7298	-0.20606
C	-4.528	-0.43272	-0.06179	C	4.44615	-0.97909	0.4949
C	-0.73139	0.41571	-0.18881	C	1.02248	0.7643	-0.13373
C	-0.19952	-0.98944	-0.5399	C	0.11418	-0.4534	0.05376
C	-0.98681	-2.04726	0.23612	C	0.76682	-1.69117	-0.59306
C	-2.4873	-1.91747	-0.031	C	2.1679	-1.92699	-0.02605
C	-3.01946	-0.33761	1.87211	C	3.4501	-0.53208	-1.70245
C	-2.14939	2.78985	0.90854	C	3.17484	2.71908	-0.79414
C	1.29489	-1.05848	-0.35088	C	-1.29608	-0.25029	-0.44056
C	1.9493	-1.82748	0.52762	C	-1.7045	0.65956	-1.33265
C	3.45076	-1.7674	0.65949	C	-3.15976	0.78965	-1.70855
C	4.02799	-0.64217	-0.20064	C	-4.03039	-0.09542	-0.81541
O	3.39839	-0.70773	-1.50096	O	-3.41126	-1.40069	-0.74117
C	2.13568	-0.12786	-1.21569	C	-2.36166	-1.14511	0.17731
C	3.67679	0.81422	0.23281	C	-4.09873	0.29364	0.69397
O	2.444	1.07949	-0.51551	O	-2.99153	-0.47611	1.27196
C	4.72859	1.79097	-0.28657	C	-5.38356	-0.24037	1.3225
C	3.41055	1.0164	1.71858	C	-3.89722	1.77074	1.0063
H	-2.41328	0.31478	-1.50032	H	2.31192	0.26783	1.49711
H	-0.39779	-1.14515	-1.61328	H	0.04414	-0.65456	1.13489
H	-6.31915	-1.15554	-0.90506	H	5.79454	-2.11484	1.65014
H	-4.89046	-2.31966	-0.98471	H	4.14675	-2.8971	1.37561
H	-3.91496	3.57243	-0.98153	H	4.61932	3.05729	1.46385
H	-3.96137	1.99838	-1.9549	H	4.07326	1.5247	2.34776
H	-4.99743	0.51182	0.20866	H	5.17285	-0.1773	0.37435
H	-0.49222	0.62669	0.85924	H	1.08803	1.00902	-1.19871
H	-0.20959	1.17519	-0.78019	H	0.57468	1.63529	0.35878
H	-0.64953	-3.05041	-0.04914	H	0.14994	-2.58074	-0.42196
H	-0.78339	-1.94216	1.30836	H	0.80538	-1.54172	-1.67872
H	-2.66598	-2.09918	-1.09826	H	2.07177	-2.14357	1.04532
H	-3.03201	-2.69593	0.51573	H	2.61376	-2.8154	-0.48835
H	-3.56869	0.55815	2.17485	H	4.24889	0.20128	-1.84262
H	-3.48637	-1.19301	2.37259	H	3.7949	-1.4782	-2.13381
H	-1.99691	-0.24827	2.24722	H	2.58267	-0.19923	-2.27756
H	-1.07327	2.95774	0.78142	H	2.16811	3.14203	-0.89469
H	-2.63419	3.76797	0.97475	H	3.87907	3.55107	-0.70498
H	-2.27165	2.28195	1.86953	H	3.3838	2.19366	-1.73047
H	1.42057	-2.52717	1.16911	H	-1.00135	1.33738	-1.80929
H	3.89407	-2.72219	0.34407	H	-3.30835	0.49559	-2.75693
H	3.7412	-1.63281	1.70732	H	-3.48374	1.83392	-1.63988
H	5.1029	-0.76252	-0.34479	H	-5.02954	-0.22398	-1.23483
H	1.64495	0.12564	-2.15888	H	-1.96268	-2.0984	0.53193
H	5.66238	1.69097	0.27612	H	-5.53242	-1.28948	1.04992
H	4.37165	2.82009	-0.18135	H	-6.25015	0.33683	0.98415
H	4.93389	1.60048	-1.34419	H	-5.32732	-0.16947	2.4131
H	3.15575	2.06395	1.90709	H	-3.93924	1.92661	2.08881
H	2.58057	0.39624	2.06313	H	-2.92933	2.12661	0.64751
H	4.30094	0.77516	2.3078	H	-4.68778	2.37376	0.54854

atom	Con f. 1- **3**a			atom	Con f. 1- **3**b		
C	5.491	-0.65589	-0.63895	C	-5.16814	-1.98351	0.18335
C	2.4507	2.64177	-0.57502	C	-4.76383	1.66832	-0.82279
C	2.83988	1.80795	0.397	C	-3.65735	1.50296	-0.08854
C	2.24848	0.41615	0.57994	C	-2.5967	0.49161	-0.50652
C	2.95772	-0.6907	-0.28891	C	-2.83047	-0.94791	0.09149

C	4.40868	-0.78125	0.13054	C	-4.11796	-1.49983	-0.48046
C	0.72208	0.39665	0.38692	C	-1.17209	1.00815	-0.23441
C	0.10717	-0.97556	0.72383	C	-0.09176	0.06371	-0.76591
C	0.78728	-2.08413	-0.10117	C	-0.28458	-1.34003	-0.15592
C	2.30687	-2.05412	0.0766	C	-1.69286	-1.86696	-0.43341
C	2.82071	-0.42682	-1.79248	C	-2.84498	-0.95555	1.6255
C	3.88517	2.2221	1.40027	C	-3.42768	2.33441	1.15048
C	-1.39009	-0.96632	0.55121	C	1.31665	0.55823	-0.54894
C	-2.26042	-1.24527	1.52868	C	1.68661	1.6083	0.1942
C	-3.74965	-1.16417	1.30819	C	3.1405	1.9597	0.39026
C	-4.05653	-0.56997	-0.06752	C	4.04603	0.89063	-0.22379
O	-3.20172	-1.2209	-1.03536	O	3.52975	0.57073	-1.53623
C	-1.97015	-0.55809	-0.79694	C	2.4295	-0.25716	-1.19584
C	-3.67223	0.92631	-0.28261	C	4.02926	-0.51189	0.45851
O	-2.30183	0.83267	-0.79652	O	2.96605	-1.20783	-0.27346
C	-4.52063	1.53654	-1.39551	C	5.3277	-1.26061	0.16856
C	-3.67851	1.79812	0.96602	C	3.6992	-0.52019	1.94517
H	2.43706	0.12559	1.62305	H	-2.69053	0.36492	-1.59296
H	0.31463	-1.1817	1.78301	H	-0.23708	-0.04058	-1.85369
H	6.48862	-0.76025	-0.21994	H	-6.03145	-2.37661	-0.34766
H	5.43481	-0.44505	-1.70277	H	-5.21777	-2.00833	1.26799
H	2.8909	3.63093	-0.67624	H	-5.53973	2.37365	-0.53404
H	1.68613	2.37801	-1.29886	H	-4.93244	1.10602	-1.73726
H	4.54957	-0.99391	1.19163	H	-4.14683	-1.51018	-1.57133
H	0.46772	0.67139	-0.64111	H	-1.02382	1.15083	0.84129
H	0.27285	1.1631	1.02772	H	-1.05983	1.99525	-0.69741
H	0.40008	-3.06046	0.21242	H	0.45076	-2.04096	-0.56394
H	0.53119	-1.97373	-1.1611	H	-0.09494	-1.28503	0.92293
H	2.54314	-2.28121	1.12477	H	-1.81267	-1.98543	-1.51863
H	2.7734	-2.84084	-0.52793	H	-1.81816	-2.86361	0.00586
H	3.31893	0.50094	-2.08247	H	-3.71359	-0.42405	2.02196
H	3.26919	-1.24672	-2.3628	H	-2.88506	-1.9837	2.00016
H	1.77504	-0.34912	-2.09726	H	-1.94902	-0.48905	2.04031
H	4.73799	1.53482	1.39019	H	-4.31003	2.93677	1.38472
H	4.25658	3.23247	1.20473	H	-3.18992	1.72163	2.02499
H	3.47413	2.19682	2.41808	H	-2.5823	3.0195	1.01308
H	-1.90598	-1.54105	2.51461	H	0.95079	2.24568	0.67647
H	-4.19703	-2.16586	1.37281	H	3.36087	2.92821	-0.07983
H	-4.22661	-0.57014	2.09547	H	3.36633	2.0841	1.45517
H	-5.09135	-0.75585	-0.35967	H	5.0668	1.25603	-0.34706
H	-1.30061	-0.75759	-1.63529	H	2.09614	-0.78424	-2.09342
H	-5.54972	1.69442	-1.05683	H	6.15924	-0.83044	0.73606
H	-4.10589	2.50277	-1.69877	H	5.22637	-2.31317	0.45048
H	-4.53571	0.87558	-2.26722	H	5.56593	-1.20791	-0.89796
H	-2.99255	1.41578	1.72458	H	3.68826	-1.55073	2.31353
H	-3.36875	2.81502	0.70566	H	4.45525	0.03203	2.51227
H	-4.68454	1.85194	1.39401	H	2.71987	-0.07813	2.13951

atom	Con f. 1- **3**c			atom	Con f. 1- **3**d		
C	-5.16484	-1.95743	0.15276	C	-5.51903	-1.28434	-0.59021
C	-3.63362	2.11248	1.2379	C	-3.3958	2.19183	1.34892
C	-3.66568	1.49021	0.05332	C	-3.33888	1.71951	0.09782
C	-2.5834	0.52105	-0.4038	C	-2.41952	0.57761	-0.3149
C	-2.81087	-0.95903	0.08958	C	-3.0469	-0.85084	-0.09729
C	-4.10766	-1.47062	-0.49883	C	-4.27711	-0.97906	-0.96938
C	-1.16684	1.02175	-0.06916	C	-1.02233	0.68927	0.32296
C	-0.08214	0.13311	-0.6833	C	-0.04749	-0.37865	-0.21536
C	-0.26581	-1.32252	-0.20542	C	-0.63385	-1.7755	0.00924
C	-1.67519	-1.8312	-0.51426	C	-2.02229	-1.89529	-0.6229
C	-2.80278	-1.07135	1.61833	C	-3.36978	-1.12383	1.3763
C	-4.7822	1.74636	-0.92592	C	-4.17608	2.32768	-0.99792
C	1.32546	0.61204	-0.43144	C	1.33471	-0.18248	0.35535
C	1.70174	1.55865	0.43669	C	1.91388	-0.92095	1.30916
C	3.1559	1.90342	0.64171	C	3.32433	-0.64369	1.7675
C	4.05817	0.94253	-0.13477	C	3.98346	0.41468	0.8819
O	3.50972	0.79309	-1.4646	O	3.04409	1.5017	0.71145
C	2.4304	-0.09143	-1.20957	C	2.16273	0.94368	-0.24887
C	4.08052	-0.53953	0.3516	C	4.25223	0.01219	-0.60077
O	3.00626	-1.14862	-0.43929	O	3.01797	0.43201	-1.27354
C	5.38046	-1.21859	-0.07222	C	5.38217	0.85713	-1.18388
C	3.79347	-0.7539	1.83156	C	4.47818	-1.47301	-0.85133
H	-2.64795	0.47113	-1.49981	H	-2.27338	0.66497	-1.40063
H	-0.23139	0.12944	-1.77549	H	0.03452	-0.22306	-1.30336
H	-6.03933	-2.31086	-0.38766	H	-6.32116	-1.36468	-1.31947

H	-5.20809	-2.02335	1.2358	H	-5.78988	-1.46383	0.44594
H	-4.42119	2.80248	1.53121	H	-4.05981	3.01266	1.60929
H	-2.83193	1.96303	1.95418	H	-2.79073	1.78668	2.15396
H	-4.14204	-1.43724	-1.58917	H	-4.08598	-0.81339	-2.03107
H	-1.02801	1.06581	1.01562	H	-1.08028	0.58605	1.41144
H	-1.06061	2.0477	-0.43814	H	-0.63194	1.6939	0.12845
H	0.46862	-1.97847	-0.68405	H	0.03349	-2.53298	-0.41802
H	-0.06375	-1.36733	0.87149	H	-0.69513	-1.98259	1.08355
H	-1.80379	-1.86472	-1.60433	H	-1.92627	-1.77193	-1.70983
H	-1.7929	-2.85993	-0.15346	H	-2.42901	-2.89962	-0.45626
H	-3.6299	-0.51528	2.06505	H	-4.14117	-0.44585	1.74815
H	-2.89489	-2.11977	1.92022	H	-3.72918	-2.15034	1.50175
H	-1.87563	-0.68693	2.04861	H	-2.49082	-1.0031	2.01272
H	-5.30551	0.8188	-1.18209	H	-4.83257	1.58048	-1.45685
H	-5.51524	2.45538	-0.52956	H	-4.79745	3.14742	-0.62519
H	-4.38448	2.15141	-1.86581	H	-3.53647	2.71775	-1.80057
H	0.97094	2.11511	1.0172	H	1.38654	-1.74495	1.78211
H	3.35022	2.92998	0.30119	H	3.32068	-0.28744	2.80709
H	3.40975	1.88717	1.7074	H	3.91842	-1.56424	1.76711
H	5.06964	1.33968	-0.23446	H	4.88206	0.82151	1.34854
H	2.07786	-0.49905	-2.16051	H	1.55047	1.74322	-0.6721
H	6.2213	-0.85615	0.52792	H	6.34935	0.54812	-0.77433
H	5.30162	-2.30156	0.06431	H	5.41611	0.74105	-2.27154
H	5.58861	-1.01563	-1.12702	H	5.2243	1.91469	-0.95211
H	2.81464	-0.35798	2.10906	H	3.61874	-2.06602	-0.53243
H	3.80691	-1.82469	2.0575	H	4.63711	-1.64625	-1.92023
H	4.55815	-0.26993	2.44736	H	5.36716	-1.82358	-0.31753
atom	Con f. 1- **3**e			atom	Con f. 1- **3**f		
C	-5.26029	-1.49952	-0.31399	C	-4.53803	-2.56612	-0.602
C	-3.72248	2.35126	-1.3405	C	-4.75737	1.66928	-0.92753
C	-2.78236	1.92709	-0.48718	C	-3.67871	1.54151	-0.14513
C	-2.28272	0.48788	-0.54503	C	-2.64105	0.46297	-0.43344
C	-3.01731	-0.51386	0.4289	C	-2.85111	-0.89144	0.34998
C	-4.5018	-0.4937	0.12508	C	-4.2305	-1.4328	0.03104
C	-0.75302	0.42108	-0.38375	C	-1.2086	1.00423	-0.26452
C	-0.19782	-1.00039	-0.58887	C	-0.14511	-0.00211	-0.70818
C	-0.90188	-1.98061	0.36679	C	-0.32937	-1.32739	0.06059
C	-2.4198	-1.91678	0.18977	C	-1.7495	-1.86742	-0.11264
C	-2.86413	-0.11881	1.91358	C	-2.79029	-0.71061	1.88141
C	-2.16575	2.87963	0.50833	C	-3.44597	2.49661	1.00046
C	1.30254	-1.03213	-0.45147	C	1.26966	0.50588	-0.58455
C	2.13579	-1.44037	-1.4157	C	1.66051	1.6313	0.02582
C	3.63221	-1.39267	-1.2397	C	3.11903	2.00024	0.13611
C	3.9954	-0.67537	0.06179	C	4.00651	0.86551	-0.37858
O	3.14601	-1.19536	1.1102	O	3.45251	0.40142	-1.63119
C	1.93166	-0.51518	0.83589	C	2.363	-0.38002	-1.16816
C	3.66704	0.84821	0.13343	C	4.00934	-0.45142	0.45808
O	2.31013	0.85606	0.69122	O	2.92761	-1.2233	-0.16161
C	4.56883	1.53661	1.15483	C	5.30032	-1.23007	0.21895
C	3.66244	1.58755	-1.19777	C	3.71952	-0.2912	1.94426
H	-2.5184	0.11496	-1.54974	H	-2.76107	0.18882	-1.48909
H	-0.44076	-1.31047	-1.61461	H	-0.31697	-0.22759	-1.77351
H	-6.32517	-1.35863	-0.48263	H	-5.5759	-2.8492	-0.75991
H	-4.86956	-2.49106	-0.52228	H	-3.79052	-3.2542	-0.98542
H	-4.09196	3.37397	-1.32069	H	-5.50987	2.43269	-0.74342
H	-4.15641	1.689	-2.08544	H	-4.92569	1.0125	-1.77729
H	-4.98064	0.46684	0.30864	H	-5.04699	-0.80434	0.38307
H	-0.4635	0.78564	0.60685	H	-1.03138	1.27546	0.78143
H	-0.28697	1.09939	-1.10763	H	-1.10904	1.92771	-0.84649
H	-0.55012	-2.99985	0.17033	H	0.38803	-2.07589	-0.29099
H	-0.63348	-1.75115	1.40466	H	-0.10243	-1.15587	1.11976
H	-2.66138	-2.23296	-0.83292	H	-1.90233	-2.09327	-1.17548
H	-2.90517	-2.63232	0.86357	H	-1.85758	-2.8141	0.42937
H	-3.42375	0.79254	2.14098	H	-3.65178	-0.14683	2.2495
H	-3.26217	-0.91561	2.55137	H	-2.8081	-1.6911	2.36982
H	-1.822	0.04749	2.19727	H	-1.88467	-0.19283	2.20634
H	-2.20021	2.49373	1.53126	H	-3.25374	1.97796	1.94404
H	-1.10811	3.05756	0.28006	H	-2.57075	3.12951	0.81122
H	-2.67805	3.84573	0.49044	H	-4.30891	3.15359	1.14096
H	1.74434	-1.81904	-2.35833	H	0.9383	2.32025	0.45472
H	4.04367	-2.4113	-1.2139	H	3.3231	2.90693	-0.45035
H	4.10693	-0.89661	-2.09349	H	3.37761	2.24814	1.1715
H	5.03096	-0.86794	0.34686	H	5.0234	1.21104	-0.57156
H	1.27765	-0.60757	1.70467	H	2.00323	-1.00325	-1.99082
H	4.59182	0.96453	2.08719	H	5.51077	-1.29538	-0.85275

H	5.5907	1.62599	0.77222	H	6.14574	-0.74158	0.71414
H	4.19348	2.54126	1.37255	H	5.20756	-2.24504	0.61746
H	4.65347	1.55473	-1.66133	H	3.7194	-1.27349	2.42698
H	2.93488	1.15813	-1.88929	H	4.49023	0.31998	2.42443
H	3.40146	2.63782	-1.03453	H	2.74572	0.17272	2.11277

atom	Con f. 1- **3**g		
C	-4.74963	-2.10653	-1.2885
C	-4.11984	2.14105	-1.33299
C	-3.34337	1.77342	-0.30627
C	-2.47	0.52934	-0.41137
C	-3.10976	-0.79038	0.1714
C	-4.42335	-1.05359	-0.53698
C	-1.06146	0.77892	0.1601
C	-0.10909	-0.40825	-0.08506
C	-0.71576	-1.68142	0.5096
C	-2.10759	-1.93942	-0.07138
C	-3.43406	-0.68047	1.67707
C	-3.28013	2.61475	0.94561
C	1.28406	-0.08976	0.39773
C	1.87478	-0.57364	1.49676
C	3.29399	-0.20477	1.85148
C	3.94392	0.59978	0.72445
O	3.00534	1.61623	0.30176
C	2.10909	0.84275	-0.4788
C	4.19252	-0.15699	-0.61608
O	2.94795	0.08536	-1.35383
C	5.31245	0.51562	-1.4062
C	4.4168	-1.65843	-0.49574
H	-2.34803	0.3244	-1.48258
H	-0.04102	-0.55106	-1.17584
H	-5.7387	-2.18585	-1.73311
H	-4.06553	-2.92476	-1.49326
H	-4.74883	3.02703	-1.28517
H	-4.15462	1.56582	-2.25479
H	-5.17435	-0.28048	-0.38274
H	-1.10768	0.96714	1.2382
H	-0.65598	1.68931	-0.29584
H	-0.06357	-2.53781	0.30319
H	-0.77603	-1.58879	1.60016
H	-2.00692	-2.09646	-1.15271
H	-2.51997	-2.86626	0.34381
H	-2.58004	-0.33472	2.26452
H	-4.2657	0.00651	1.85595
H	-3.7316	-1.66146	2.06346
H	-2.28964	3.07039	1.06341
H	-4.0167	3.42232	0.90832
H	-3.46035	2.02918	1.85172
H	1.35081	-1.24792	2.16879
H	3.3092	0.39433	2.77268
H	3.88369	-1.10265	2.06706
H	4.84902	1.10425	1.06649
H	1.49499	1.51656	-1.08045
H	5.33264	0.13251	-2.43109
H	5.15451	1.59764	-1.44269
H	6.28573	0.31814	-0.94536
H	3.56345	-2.15265	-0.0269
H	4.55862	-2.0903	-1.49134
H	5.31486	-1.869	0.09358

atom	Con f. 1- **4**a			atom	Con f. 1- **4**b		
C	-5.18271	-1.87004	0.07776	C	5.21373	1.87291	0.1157
C	-3.37381	1.95958	1.59887	C	4.6791	-1.86029	-0.52154
C	-3.54239	1.51355	0.34829	C	3.54894	-1.56137	0.12979
C	-2.54948	0.58797	-0.34262	C	2.56409	-0.55637	-0.45489
C	-2.80381	-0.94002	-0.05278	C	2.83857	0.93116	-0.01081
C	-4.16899	-1.31443	-0.58784	C	4.18621	1.35182	-0.55535
C	-1.08798	0.98544	-0.07042	C	1.10268	-0.97191	-0.21156
C	-0.08991	0.14356	-0.88855	C	0.09899	-0.03728	-0.91176
C	-0.31423	-1.35635	-0.61917	C	0.33597	1.41673	-0.46499
C	-1.76858	-1.74965	-0.88319	C	1.78611	1.83376	-0.71468
C	-2.66767	-1.28252	1.43506	C	2.76212	1.11832	1.50933
C	-4.73465	1.93773	-0.47007	C	3.21732	-2.23806	1.43764
C	1.33511	0.56057	-0.6333	C	-1.32414	-0.4805	-0.69028

C	2.13159	1.11505	-1.55441	C	-2.12467	-0.94519	-1.65662
C	3.56248	1.47623	-1.24783	C	-3.55413	-1.33569	-1.37878
C	3.95954	0.97081	0.14144	C	-3.94428	-0.96179	0.05244
O	2.89466	1.30361	1.06171	O	-2.87743	-1.38278	0.93293
C	1.93892	0.30502	0.74099	C	-1.92075	-0.35992	0.70582
C	4.04071	-0.57566	0.32244	C	-4.02041	0.56167	0.37785
O	2.67786	-0.91909	0.74228	O	-2.65704	0.85953	0.82981
C	4.96364	-0.92568	1.48704	C	-4.94391	0.80322	1.56923
C	4.39222	-1.36984	-0.92816	C	-4.36745	1.4722	-0.79224
H	-2.708	0.70352	-1.42398	H	2.7184	-0.55817	-1.542
H	-0.30008	0.32433	-1.95182	H	0.29573	-0.08954	-1.99149
H	-6.1154	-2.11117	-0.42588	H	6.1276	2.16027	-0.39818
H	-5.13194	-2.10438	1.13689	H	5.19471	2.03332	1.18973
H	-4.10143	2.62249	2.06093	H	5.40237	-2.56395	-0.11572
H	-2.51519	1.68899	2.20531	H	4.92129	-1.41294	-1.4819
H	-4.29858	-1.10933	-1.65192	H	4.28587	1.22265	-1.63431
H	-0.86228	0.89079	0.99614	H	0.8918	-0.99616	0.86239
H	-0.95875	2.04414	-0.32048	H	0.95786	-1.99406	-0.57974
H	0.35073	-1.94746	-1.25927	H	-0.34057	2.08471	-1.01033
H	-0.03878	-1.59502	0.41447	H	0.08875	1.52639	0.59727
H	-1.98514	-1.59829	-1.94919	H	1.97544	1.80983	-1.79619
H	-1.91485	-2.81738	-0.6819	H	1.94329	2.86977	-0.39212
H	-3.42558	-0.77327	2.03452	H	3.57306	0.59198	2.01865
H	-2.78223	-2.36084	1.58572	H	2.84226	2.17978	1.76636
H	-1.68964	-0.99876	1.82906	H	1.81712	0.75322	1.91732
H	-5.39752	2.60091	0.09361	H	4.05726	-2.84651	1.78465
H	-4.41128	2.46359	-1.37805	H	2.9647	-1.52232	2.22538
H	-5.31652	1.0709	-0.80123	H	2.3486	-2.89866	1.32919
H	1.75524	1.32419	-2.55436	H	-1.75378	-1.05573	-2.67415
H	3.69142	2.56708	-1.28169	H	-3.68295	-2.41862	-1.51397
H	4.23777	1.06986	-2.0088	H	-4.23296	-0.85963	-2.09482
H	4.86958	1.4573	0.49618	H	-4.85459	-1.47695	0.36344
H	1.19583	0.26623	1.53951	H	-1.17382	-0.40055	1.50061
H	4.84401	-1.97902	1.7586	H	-5.99144	0.6637	1.28299
H	6.01111	-0.75621	1.21728	H	-4.70662	0.10879	2.3807
H	4.72241	-0.31208	2.36015	H	-4.8215	1.82562	1.93965
H	3.66382	-1.20309	-1.72423	H	-4.37554	2.51427	-0.45781
H	5.38584	-1.093	-1.29449	H	-3.63883	1.37853	-1.59998
H	4.4052	-2.43893	-0.69428	H	-5.36218	1.23634	-1.18338

atom	Con f. 1- **4**c			atom	Con f. 1- **4**d		
C	-5.56359	-0.60736	0.33197	C	-5.54736	-1.21848	-0.49383
C	-2.40246	2.526	0.98788	C	-3.31537	2.35549	1.15387
C	-2.74929	1.86634	-0.12373	C	-3.27863	1.77001	-0.04924
C	-2.20144	0.48892	-0.4734	C	-2.39172	0.5712	-0.35946
C	-3.01287	-0.69713	0.17091	C	-3.06506	-0.81481	-0.02632
C	-4.42752	-0.66581	-0.3647	C	-4.29661	-0.97872	-0.89059
C	-0.69404	0.37075	-0.18785	C	-0.99535	0.69377	0.27709
C	-0.10728	-0.97163	-0.67753	C	-0.0554	-0.42903	-0.17256
C	-0.87724	-2.13787	-0.0529	C	-0.68076	-1.79934	0.15981
C	-2.37687	-2.02282	-0.33412	C	-2.07419	-1.93193	-0.45763
C	-2.989	-0.65187	1.70325	C	-3.39949	-0.95423	1.463
C	-3.69481	2.47852	-1.12486	C	-4.1065	2.29904	-1.19226
C	1.38302	-1.01471	-0.44856	C	1.34548	-0.31506	0.37419
C	2.03461	-1.8339	0.38561	C	1.73404	0.41288	1.42778
C	3.52749	-1.73577	0.5775	C	3.18205	0.47451	1.84711
C	4.09119	-0.52196	-0.16283	C	4.07438	-0.22606	0.82156
O	3.51119	-0.50081	-1.48758	O	3.46213	-1.49433	0.49021
C	2.2189	0.01072	-1.20461	C	2.42885	-1.07061	-0.38348
C	3.66898	0.88008	0.37636	C	4.17209	0.43869	-0.58558
O	2.45912	1.16767	-0.40119	O	3.07468	-0.20328	-1.31796
C	4.70439	1.93276	-0.01041	C	5.46801	0.02599	-1.27912
C	3.33288	0.9441	1.86034	C	3.97735	1.94896	-0.61916
H	-2.32485	0.36527	-1.55854	H	-2.24115	0.56252	-1.44806
H	-0.27	-1.00981	-1.7673	H	0.02955	-0.37216	-1.27016
H	-6.52716	-0.60844	-0.17125	H	-6.34946	-1.33582	-1.21808
H	-5.58601	-0.55432	1.41633	H	-5.82588	-1.30556	0.55214
H	-2.808	3.51097	1.20631	H	-3.95695	3.21307	1.34174
H	-1.70781	2.11711	1.71488	H	-2.71428	2.01075	1.98936
H	-4.48992	-0.71719	-1.45311	H	-4.09824	-0.90549	-1.96129
H	-0.4906	0.46866	0.88381	H	-1.07739	0.68455	1.36846
H	-0.18372	1.20828	-0.67297	H	-0.56693	1.66489	0.00624
H	-0.50211	-3.08866	-0.44898	H	-0.0437	-2.61118	-0.2092
H	-0.69929	-2.15832	1.02847	H	-0.72742	-1.91076	1.24951

H	-2.53978	-2.09617	-1.41757	H	-1.97532	-1.90696	-1.55099
H	-2.91234	-2.86427	0.12095	H	-2.51008	-2.90535	-0.20384
H	-3.48969	0.24092	2.08439	H	-4.14691	-0.2214	1.77475
H	-3.49631	-1.52977	2.11628	H	-3.79495	-1.95389	1.67006
H	-1.96852	-0.64865	2.09123	H	-2.51741	-0.81382	2.0911
H	-3.20429	2.58727	-2.10117	H	-4.70827	3.1618	-0.8912
H	-4.57075	1.84053	-1.28453	H	-3.46109	2.60359	-2.02669
H	-4.04261	3.46486	-0.8035	H	-4.78098	1.52993	-1.5835
H	1.50914	-2.60048	0.94835	H	1.01934	0.98429	2.01388
H	4.01649	-2.64426	0.19924	H	3.31327	-0.01047	2.8245
H	3.77635	-1.68183	1.64313	H	3.50117	1.51391	1.9819
H	5.17439	-0.59141	-0.2748	H	5.06518	-0.43145	1.23009
H	1.75166	0.3227	-2.1422	H	2.04461	-1.93954	-0.92227
H	4.3059	2.93656	0.16629	H	5.61094	-1.05617	-1.20474
H	5.61704	1.81509	0.58274	H	5.43203	0.30007	-2.33803
H	4.96069	1.8423	-1.07024	H	6.32857	0.52611	-0.82311
H	4.20707	0.68789	2.46716	H	3.00121	2.23443	-0.22192
H	3.02618	1.96111	2.12386	H	4.75648	2.45222	-0.03796
H	2.51604	0.26506	2.11292	H	4.04472	2.3052	-1.65178

atom	Con f. 1- **4**e			atom	Con f. 1- **4**f		
C	4.65963	2.41224	-0.6644	C	-5.27343	-1.35036	-0.6798
C	4.67829	-1.85442	-0.69329	C	-3.57096	2.54772	-1.10196
C	3.57101	-1.61414	0.01946	C	-2.7114	1.98904	-0.24118
C	2.60683	-0.51235	-0.40401	C	-2.24391	0.55254	-0.44242
C	2.84186	0.8846	0.29141	C	-3.07126	-0.53781	0.34227
C	4.26259	1.33568	0.01612	C	-4.528	-0.43272	-0.0618
C	1.14113	-0.9732	-0.2848	C	-0.73139	0.41571	-0.18881
C	0.15116	0.06069	-0.8516	C	-0.19952	-0.98944	-0.5399
C	0.36569	1.42291	-0.16656	C	-0.98681	-2.04726	0.23612
C	1.81959	1.8769	-0.30251	C	-2.4873	-1.91747	-0.031
C	2.68591	0.8212	1.82584	C	-3.01946	-0.33761	1.87211
C	3.23305	-2.46577	1.21922	C	-2.14939	2.78985	0.90854
C	-1.27897	-0.40414	-0.74998	C	1.29489	-1.05848	-0.35088
C	-2.05931	-0.66234	-1.80592	C	1.9493	-1.82748	0.52762
C	-3.50471	-1.05803	-1.64317	C	3.45076	-1.7674	0.65949
C	-3.93068	-0.93901	-0.17854	C	4.02799	-0.64217	-0.20064
O	-2.90241	-1.54772	0.6361	O	3.39839	-0.70773	-1.50096
C	-1.91415	-0.52985	0.62866	C	2.13568	-0.12786	-1.21569
C	-3.97655	0.50224	0.41758	C	3.67678	0.81422	0.23281
O	-2.62429	0.66494	0.96231	O	2.444	1.07949	-0.51551
C	-4.93879	0.55687	1.60149	C	4.72859	1.79097	-0.28657
C	-4.25035	1.62257	-0.57703	C	3.41055	1.0164	1.71858
H	2.79812	-0.32249	-1.46783	H	-2.41328	0.31477	-1.50032
H	0.37964	0.18712	-1.9188	H	-0.39779	-1.14515	-1.61328
H	5.71724	2.63508	-0.78291	H	-6.31916	-1.15553	-0.90506
H	3.96826	3.11065	-1.12615	H	-4.89047	-2.31965	-0.98471
H	5.37918	-2.63711	-0.41241	H	-3.91495	3.57243	-0.98153
H	4.92251	-1.27499	-1.58009	H	-3.96136	1.99839	-1.95491
H	5.02651	0.6903	0.44677	H	-4.99743	0.51182	0.20866
H	0.89688	-1.18155	0.76196	H	-0.49222	0.62669	0.85924
H	1.02235	-1.9196	-0.82495	H	-0.20959	1.17519	-0.78019
H	-0.29923	2.16877	-0.61666	H	-0.64954	-3.05041	-0.04915
H	0.08491	1.35791	0.89087	H	-0.78339	-1.94216	1.30836
H	2.03944	2.01396	-1.3688	H	-2.66598	-2.09918	-1.09826
H	1.95152	2.85501	0.17454	H	-3.03201	-2.69593	0.51572
H	3.49659	0.24858	2.28443	H	-3.48637	-1.19301	2.37258
H	2.72304	1.83351	2.24297	H	-3.56869	0.55815	2.17485
H	1.7392	0.36881	2.13128	H	-1.99691	-0.24827	2.24722
H	4.05715	-3.14322	1.46038	H	-2.27165	2.28194	1.86953
H	3.01133	-1.86735	2.10747	H	-1.07327	2.95774	0.78142
H	2.34329	-3.07754	1.02787	H	-2.63419	3.76797	0.97475
H	-1.66132	-0.58677	-2.81642	H	1.42057	-2.52718	1.16911
H	-3.65502	-2.09419	-1.97691	H	3.89407	-2.72219	0.34407
H	-4.14891	-0.43997	-2.27831	H	3.7412	-1.63281	1.70732
H	-4.86354	-1.47299	0.00985	H	5.1029	-0.76252	-0.34479
H	-1.19256	-0.74034	1.41985	H	1.64495	0.12563	-2.15888
H	-5.97762	0.50813	1.25954	H	5.66238	1.69097	0.27613
H	-4.75305	-0.28007	2.28124	H	4.93389	1.60048	-1.34418
H	-4.80202	1.49109	2.15481	H	4.37165	2.82009	-0.18136
H	-3.49644	1.64848	-1.36629	H	2.58056	0.39624	2.06313
H	-4.23592	2.58597	-0.05796	H	4.30093	0.77517	2.3078
H	-5.23738	1.50108	-1.03435	H	3.15573	2.06395	1.90708

atom	Con f. 1- 4g		
C	4.80537	-2.04852	1.20563
C	4.02222	2.14897	1.45835
C	3.26307	1.81154	0.40862
C	2.43351	0.53384	0.43962
C	3.1257	-0.72977	-0.20629
C	4.44674	-0.97859	0.49388
C	1.02248	0.7642	-0.13296
C	0.11442	-0.45356	0.0548
C	0.76681	-1.69143	-0.59197
C	2.1682	-1.92705	-0.02545
C	3.44893	-0.53215	-1.70287
C	3.17422	2.71877	-0.79458
C	-1.29595	-0.2508	-0.43955
C	-1.70418	0.658	-1.33277
C	-3.15934	0.78751	-1.70934
C	-4.03021	-0.09659	-0.81555
O	-3.41085	-1.40171	-0.73948
C	-2.36171	-1.14493	0.17903
C	-4.09915	0.29431	0.69324
O	-2.99206	-0.47459	1.27262
C	-5.38411	-0.23893	1.32221
C	-3.89798	1.77187	1.00364
H	2.31274	0.26762	1.49739
H	0.04451	-0.65469	1.13593
H	5.79585	-2.1131	1.64948
H	4.14791	-2.89572	1.37668
H	4.61978	3.05768	1.46271
H	4.07429	1.52517	2.34718
H	5.17344	-0.17699	0.37203
H	1.0878	1.0089	-1.19794
H	0.57481	1.63516	0.35966
H	0.15002	-2.58094	-0.42023
H	0.80497	-1.54229	-1.67767
H	2.0724	-2.14325	1.04603
H	2.61395	-2.81559	-0.48761
H	4.24738	0.20148	-1.84377
H	3.79376	-1.47819	-2.13442
H	2.58098	-0.19972	-2.27747
H	3.87891	3.55049	-0.7064
H	3.38204	2.19287	-1.73089
H	2.16758	3.14214	-0.89433
H	-1.00102	1.33541	-1.81002
H	-3.30735	0.49199	-2.75743
H	-3.48348	1.83181	-1.64227
H	-5.0292	-0.22587	-1.23514
H	-1.96268	-2.09769	0.53512
H	-6.2507	0.33783	0.9831
H	-5.53296	-1.28839	1.05093
H	-5.32787	-0.16661	2.41272
H	-2.93023	2.1277	0.6443
H	-4.68879	2.37402	0.5452
H	-3.93989	1.92912	2.08596

atom	Con f. 2- 1a			atom	Con f. 2- 1b		
C	6.03513	-1.24258	-0.45925	C	5.41718	-2.21427	0.18254
C	3.74962	2.65574	-0.44182	C	4.79418	1.77455	1.64012
C	3.85163	1.69026	0.47981	C	3.86831	1.70566	0.67571
C	2.96711	0.45053	0.48185	C	3.02466	0.44771	0.50743
C	3.52967	-0.72898	-0.4003	C	3.57707	-0.59035	-0.54621
C	4.86082	-1.16941	0.16913	C	4.98088	-0.99702	-0.14571
C	1.50281	0.7713	0.13152	C	1.54705	0.79568	0.24341
C	0.59089	-0.44685	0.31298	C	0.64825	-0.44393	0.24742
C	1.1013	-1.61126	-0.55878	C	1.1604	-1.44803	-0.80425
C	2.56267	-1.93556	-0.24092	C	2.62531	-1.80514	-0.54539
C	3.64964	-0.34226	-1.87883	C	3.67731	0.00153	-1.96884
C	4.84491	1.79924	1.60786	C	3.60666	2.89128	-0.22128
C	-0.87526	-0.16425	0.05843	C	-0.82366	-0.13956	0.05815
C	-1.34727	0.79493	-0.74649	C	-1.31607	0.95343	-0.53639
C	-2.8141	1.04667	-0.96236	C	-2.78746	1.21603	-0.6998
C	-3.64334	0.3729	0.12265	C	-3.60678	0.31677	0.2165
O	-3.16713	-0.97862	0.2951	O	-3.0985	-1.03031	0.12026
C	-1.84012	-1.08117	0.77853	C	-1.7711	-1.19438	0.58693
O	-1.78449	-0.89837	2.18768	O	-1.72398	-1.27355	2.00592

C	-5.14823	0.24376	-0.18363	C	-5.10557	0.21779	-0.13057
O	-5.31773	-0.59128	-1.34577	O	-5.24253	-0.37762	-1.43553
C	-5.75832	1.59688	-0.53678	C	-5.74434	1.60023	-0.22134
C	-5.87971	-0.381	1.00958	C	-5.8319	-0.64603	0.90635
H	2.96633	0.06355	1.51058	H	3.06575	-0.08508	1.46591
H	0.67501	-0.76797	1.36125	H	0.75047	-0.92696	1.22994
H	-3.52596	0.91922	1.06855	H	-3.51097	0.66167	1.25484
H	6.92376	-1.59479	0.05839	H	6.4609	-2.37904	0.43896
H	6.1642	-0.95829	-1.49941	H	4.77381	-3.08854	0.21072
H	4.39308	3.53184	-0.41112	H	5.40589	2.6625	1.78206
H	3.03009	2.61408	-1.25337	H	4.97355	0.94358	2.31788
H	4.8142	-1.47406	1.21605	H	5.69889	-0.17856	-0.14943
H	1.43707	1.1308	-0.89955	H	1.45582	1.30951	-0.71861
H	1.15633	1.59149	0.76981	H	1.20501	1.50224	1.00855
H	0.49007	-2.50608	-0.39598	H	0.55721	-2.36254	-0.78651
H	0.98402	-1.34017	-1.61488	H	1.03537	-1.00971	-1.80184
H	2.62252	-2.29522	0.79494	H	2.68914	-2.30141	0.43108
H	2.91622	-2.75501	-0.87786	H	2.97036	-2.53109	-1.29079
H	2.69333	-0.01344	-2.29101	H	4.46479	0.75752	-2.03024
H	4.36762	0.46831	-2.02277	H	3.92815	-0.79173	-2.68147
H	3.98354	-1.2021	-2.46848	H	2.74173	0.4616	-2.29664
H	5.43462	2.71827	1.54051	H	4.3426	3.68133	-0.047
H	4.32929	1.79079	2.57717	H	3.6341	2.62621	-1.28191
H	5.53424	0.94828	1.61262	H	2.61208	3.31336	-0.03362
H	-0.65822	1.44212	-1.28291	H	-0.64159	1.70747	-0.93325
H	-3.12364	0.67552	-1.94895	H	-3.08687	1.05292	-1.74416
H	-3.00431	2.12535	-0.95957	H	-2.99896	2.26681	-0.47427
H	-1.57353	-2.13054	0.62048	H	-1.48368	-2.19062	0.23822
H	-1.7457	0.05399	2.36593	H	-1.67683	-0.37056	2.35612
H	-4.81016	-1.40015	-1.173	H	-4.70158	-1.18332	-1.42167
H	-5.32094	2.00492	-1.45147	H	-6.81684	1.49974	-0.41423
H	-6.83537	1.48579	-0.69579	H	-5.61465	2.1482	0.71665
H	-5.60421	2.31428	0.27471	H	-5.30641	2.18721	-1.03255
H	-6.936	-0.52271	0.76244	H	-5.37582	-1.63716	0.9749
H	-5.44777	-1.35157	1.26705	H	-6.88155	-0.76262	0.62075
H	-5.81401	0.26615	1.89047	H	-5.79207	-0.18282	1.8978

atom	Con f. 2- 1c			atom	Con f. 2- 1d		
C	6.03343	-1.26655	-0.38473	C	-6.10138	-1.02443	-0.06211
C	3.75704	2.6105	-0.62955	C	-3.3911	2.0503	1.77635
C	3.85265	1.7141	0.35989	C	-3.59979	1.73304	0.49287
C	2.96586	0.47912	0.44698	C	-2.86726	0.59744	-0.20972
C	3.52606	-0.75968	-0.35191	C	-3.5528	-0.80898	-0.02607
C	4.86083	-1.15562	0.24134	C	-4.93232	-0.75874	-0.64699
C	1.50148	0.77739	0.07681	C	-1.37109	0.55471	0.14921
C	0.58959	-0.4255	0.34395	C	-0.59953	-0.49796	-0.67054
C	1.09939	-1.65203	-0.43825	C	-1.24585	-1.87988	-0.49562
C	2.56127	-1.95265	-0.10116	C	-2.73327	-1.83981	-0.85203
C	3.63884	-0.47928	-1.8546	C	-3.61257	-1.24028	1.44358
C	4.84082	1.90236	1.48197	C	-4.56566	2.52225	-0.35284
C	-0.87557	-0.16791	0.05972	C	0.88089	-0.47968	-0.34006
C	-1.3426	0.67251	-0.87152	C	1.42411	-1.06813	0.73187
C	-2.81003	0.88534	-1.13123	C	2.88561	-0.9762	1.07199
C	-3.6301	0.36129	0.03826	C	3.54936	0.15834	0.30264
O	-3.16125	-0.95786	0.38945	O	3.141	0.08233	-1.07932
C	-1.83922	-0.97783	0.89982	C	1.75783	0.28682	-1.30205
O	-1.79971	-0.58569	2.26542	O	1.43159	1.67089	-1.32896
C	-5.14884	0.22975	-0.16786	C	5.09022	0.12183	0.2794
O	-5.69751	-0.25418	1.07592	O	5.51794	-1.07973	-0.39202
C	-5.51029	-0.74873	-1.28982	C	5.66221	0.04779	1.69192
C	-5.7778	1.59879	-0.4051	C	5.63704	1.34621	-0.46225
H	2.96504	0.16351	1.49982	H	-2.92347	0.80401	-1.28771
H	0.66999	-0.66663	1.41357	H	-0.70555	-0.22122	-1.72943
H	-3.48312	1.02253	0.90306	H	3.22929	1.12283	0.7199
H	6.92536	-1.5783	0.1528	H	-7.03122	-0.96852	-0.62243
H	6.15808	-1.05364	-1.44233	H	-6.18477	-1.30333	0.98406
H	4.40216	3.48542	-0.65929	H	-3.92787	2.87082	2.24656
H	3.04072	2.51246	-1.43932	H	-2.6855	1.51378	2.40286
H	4.81903	-1.38756	1.30693	H	-4.93194	-0.48465	-1.70343
H	1.43482	1.06598	-0.97616	H	-1.23138	0.3415	1.21401
H	1.15578	1.63925	0.65809	H	-0.93858	1.54423	-0.03049
H	0.48535	-2.53031	-0.20789	H	-0.73412	-2.61027	-1.13306
H	0.97999	-1.46012	-1.51124	H	-1.11745	-2.22371	0.53649
H	2.6246	-2.23407	0.95844	H	-2.83372	-1.59012	-1.9168

H	2.91318	-2.81671	-0.67702	H	-3.18173	-2.83151	-0.71946
H	2.67875	-0.18929	-2.28642	H	-2.62102	-1.26102	1.90016
H	4.34943	0.32488	-2.05832	H	-4.23382	-0.563	2.03368
H	3.9789	-1.3762	-2.38215	H	-4.03377	-2.24752	1.52529
H	5.5233	1.04915	1.55602	H	-5.3428	1.87609	-0.77495
H	5.43781	2.8093	1.34783	H	-5.05308	3.31583	0.22131
H	4.32002	1.97193	2.44605	H	-4.04507	2.98247	-1.20307
H	-0.65032	1.23718	-1.49072	H	0.79842	-1.64496	1.40916
H	-3.09569	0.38655	-2.06706	H	3.39116	-1.92434	0.84338
H	-3.01182	1.95265	-1.2736	H	3.00107	-0.81139	2.14866
H	-1.5613	-2.03616	0.90005	H	1.59098	-0.06029	-2.32705
H	-1.75612	0.38259	2.29934	H	1.32628	1.9729	-0.41348
H	-5.1307	-0.99746	1.33932	H	5.0361	-1.10382	-1.23399
H	-5.04479	-1.72388	-1.12102	H	5.36381	-0.87673	2.19246
H	-6.59584	-0.88308	-1.31458	H	6.75536	0.07443	1.64897
H	-5.19046	-0.37985	-2.26861	H	5.322	0.89638	2.29279
H	-6.86819	1.505	-0.41529	H	6.72727	1.28273	-0.52789
H	-5.50009	2.29813	0.39005	H	5.22806	1.40205	-1.4745
H	-5.46287	2.01639	-1.36479	H	5.37502	2.27059	0.06306

atom	Con f. 2- 1e			atom	Con f. 2- 1f		
C	-5.41319	-2.19833	-0.28244	C	-5.93679	-1.19894	0.6
C	-4.77693	1.82694	-1.60303	C	-3.49881	2.57857	0.68405
C	-3.85937	1.73018	-0.63307	C	-3.75808	1.73462	-0.32207
C	-3.01853	0.46684	-0.49339	C	-2.9404	0.47513	-0.57855
C	-3.58298	-0.60041	0.52418	C	-3.44094	-0.77884	0.23461
C	-4.98267	-0.99386	0.09676	C	-4.86021	-1.09786	-0.18114
C	-1.54292	0.80448	-0.205	C	-1.43144	0.70534	-0.38323
C	-0.64625	-0.43631	-0.23555	C	-0.59639	-0.51814	-0.7963
C	-1.17025	-1.46794	0.78323	C	-1.0685	-1.76668	-0.02416
C	-2.63253	-1.81572	0.49851	C	-2.5711	-1.99063	-0.20095
C	-3.69712	-0.05016	1.96244	C	-3.32937	-0.56863	1.74937
C	-3.6051	2.88957	0.29941	C	-4.87838	2.00765	-1.29251
C	0.82523	-0.1414	-0.02663	C	0.8998	-0.29642	-0.66932
C	1.31478	0.94749	0.57907	C	1.76205	-0.75355	-1.58585
C	2.78686	1.20301	0.76224	C	3.25454	-0.637	-1.45981
C	3.5934	0.31085	-0.16885	C	3.64344	-0.29705	-0.02728
O	3.09551	-1.0404	-0.07191	O	2.79722	0.77573	0.43387
C	1.77124	-1.20193	-0.55105	C	1.42092	0.45499	0.53691
O	1.73295	-1.2838	-1.96948	O	1.13516	-0.21255	1.75942
C	5.10936	0.22077	0.07615	C	5.08766	0.2087	0.15966
O	5.64143	-0.65954	-0.93597	O	5.24495	1.44431	-0.56489
C	5.45138	-0.34023	1.46016	C	6.09651	-0.76809	-0.43645
C	5.77166	1.57562	-0.15113	C	5.37682	0.4446	1.64605
H	-3.05084	-0.0379	-1.46721	H	-3.08853	0.21132	-1.6352
H	-0.74095	-0.8922	-1.23174	H	-0.79618	-0.70984	-1.85978
H	3.45901	0.65547	-1.20333	H	3.49518	-1.17594	0.6149
H	-6.45401	-2.35489	-0.55524	H	-6.90734	-1.45455	0.18242
H	-4.76763	-3.06969	-0.33804	H	-5.90196	-1.0328	1.67261
H	-5.38721	2.71883	-1.72485	H	-4.10039	3.47134	0.83703
H	-4.95078	1.01558	-2.30554	H	-2.68682	2.4173	1.38579
H	-5.70241	-0.17738	0.12476	H	-4.97758	-1.28034	-1.25075
H	-1.4601	1.29075	0.77205	H	-1.22817	0.95011	0.66281
H	-1.1926	1.53189	-0.94647	H	-1.12838	1.57763	-0.97335
H	-0.56897	-2.38293	0.74775	H	-0.51972	-2.64396	-0.38572
H	-1.0556	-1.05658	1.79344	H	-0.82294	-1.65573	1.03618
H	-2.68583	-2.28373	-0.49242	H	-2.77514	-2.20264	-1.25904
H	-2.98652	-2.56245	1.21877	H	-2.89093	-2.87444	0.36372
H	-2.76379	0.39727	2.31341	H	-2.30897	-0.32342	2.05099
H	-4.48314	0.70609	2.03792	H	-3.97813	0.24221	2.08834
H	-3.95738	-0.86336	2.64874	H	-3.61889	-1.48119	2.28055
H	-2.60835	3.31561	0.1334	H	-4.48945	2.09675	-2.3154
H	-4.3385	3.68519	0.14036	H	-5.60147	1.18518	-1.30311
H	-3.64326	2.59481	1.35186	H	-5.41247	2.93027	-1.04644
H	0.63836	1.70148	0.97208	H	1.37615	-1.25147	-2.47429
H	3.06475	1.03082	1.81067	H	3.6386	0.13432	-2.14124
H	3.00949	2.25461	0.55067	H	3.72193	-1.58119	-1.75933
H	1.4783	-2.19641	-0.2024	H	0.93105	1.42763	0.62832
H	1.68287	-0.38285	-2.32445	H	1.3288	-1.15492	1.64029
H	5.05197	-1.43138	-0.94668	H	4.5078	2.01304	-0.29064
H	4.96524	-1.30694	1.61976	H	5.96452	-0.86501	-1.51702
H	6.53381	-0.48106	1.53502	H	7.11314	-0.40927	-0.24862
H	5.13967	0.33665	2.26103	H	5.99267	-1.75743	0.01894
H	6.85956	1.46319	-0.1103	H	5.32002	-0.49336	2.20832

H	5.50403	1.97958	-1.13272	H	6.38166	0.8601	1.76658
H	5.47527	2.29508	0.61653	H	4.65529	1.14387	2.07672

atom	Con f. 2- 1g			atom	Con f. 2- 1h		
C	-5.61711	-1.6416	-1.00155	C	-6.10328	-1.01107	-0.02524
C	-4.48817	2.46048	-0.74266	C	-3.37834	2.11707	1.69775
C	-3.58793	1.90922	0.08054	C	-3.58511	1.75272	0.42656
C	-2.90862	0.59601	-0.28933	C	-2.85837	0.58621	-0.22967
C	-3.60948	-0.69995	0.27761	C	-3.55381	-0.8074	0.00572
C	-5.04481	-0.73499	-0.20754	C	-4.93252	-0.77145	-0.61789
C	-1.4057	0.62386	0.0461	C	-1.36314	0.54687	0.13386
C	-0.66293	-0.62816	-0.4554	C	-0.59883	-0.53938	-0.64824
C	-1.33002	-1.88899	0.11229	C	-1.25363	-1.90982	-0.42195
C	-2.81835	-1.92042	-0.24005	C	-2.7406	-1.8736	-0.78076
C	-3.66361	-0.71999	1.82055	C	-3.61748	-1.18363	1.49032
C	-3.19544	2.60464	1.36156	C	-4.54311	2.51498	-0.45217
C	0.82174	-0.54181	-0.16171	C	0.88343	-0.51825	-0.32508
C	1.37316	-0.7674	1.03547	C	1.42965	-1.0904	0.7548
C	2.84104	-0.59109	1.3131	C	2.89599	-1.00458	1.08261
C	3.50678	0.21378	0.20388	C	3.54303	0.12104	0.28989
O	3.08146	-0.32136	-1.06837	O	3.13977	0.0154	-1.09195
C	1.70026	-0.16101	-1.32787	C	1.75583	0.22647	-1.31032
O	1.39523	1.17769	-1.69061	O	1.43729	1.61077	-1.36386
C	5.04612	0.15672	0.18286	C	5.08008	0.18045	0.27626
O	5.46006	-1.20549	-0.04436	O	5.4454	1.3374	-0.50529
C	5.63231	0.56124	1.53184	C	5.71257	-1.06729	-0.34836
C	5.59552	1.05149	-0.9338	C	5.62028	0.43049	1.68035
H	-2.99261	0.49769	-1.37921	H	-2.91047	0.75193	-1.31487
H	-0.78264	-0.66107	-1.54785	H	-0.70652	-0.30051	-1.71612
H	3.19091	1.26262	0.2684	H	3.19095	1.08484	0.68296
H	-6.66789	-1.5606	-1.26901	H	-7.03235	-0.97058	-0.58815
H	-5.08178	-2.4913	-1.41467	H	-6.18896	-1.25213	1.0301
H	-4.98475	3.39746	-0.50089	H	-3.9108	2.95853	2.13469
H	-4.76124	1.99051	-1.68407	H	-2.67829	1.59974	2.34631
H	-5.65979	0.08368	0.16266	H	-4.92994	-0.53522	-1.6834
H	-1.25437	0.70897	1.12739	H	-1.22637	0.3699	1.2056
H	-0.95661	1.51639	-0.40247	H	-0.92452	1.52658	-0.08026
H	-0.83673	-2.78211	-0.28848	H	-0.74617	-2.66679	-1.03114
H	-1.19911	-1.92095	1.19955	H	-1.12841	-2.21576	0.62234
H	-2.91319	-1.96384	-1.33246	H	-2.83879	-1.66259	-1.85407
H	-3.27676	-2.8359	0.15189	H	-3.19553	-2.85679	-0.61201
H	-4.35211	0.03816	2.20382	H	-2.62659	-1.19542	1.94882
H	-4.0255	-1.695	2.16495	H	-4.23384	-0.48054	2.05484
H	-2.68532	-0.54709	2.2758	H	-4.04629	-2.18402	1.60885
H	-3.82424	3.48233	1.53566	H	-5.32316	1.85789	-0.8515
H	-3.27493	1.94865	2.23321	H	-5.0274	3.33321	0.08906
H	-2.15295	2.94239	1.32201	H	-4.01692	2.93828	-1.31795
H	0.75017	-1.08479	1.86883	H	0.80512	-1.6521	1.44539
H	3.33334	-1.56823	1.41117	H	3.38186	-1.96588	0.86871
H	2.97066	-0.07787	2.27219	H	3.02556	-0.82257	2.15511
H	1.5064	-0.8285	-2.17752	H	1.57936	-0.14172	-2.32621
H	1.79313	1.34309	-2.55971	H	1.33135	1.93258	-0.45522
H	4.96254	-1.51225	-0.81899	H	4.91073	1.28545	-1.31431
H	6.72521	0.55626	1.47557	H	5.31422	-1.24408	-1.35131
H	5.30805	1.5688	1.80869	H	6.79441	-0.92324	-0.42521
H	5.32946	-0.13229	2.32021	H	5.53008	-1.96089	0.25565
H	5.19378	0.75412	-1.906	H	6.69944	0.60653	1.63238
H	6.68619	0.97206	-0.96844	H	5.14829	1.31068	2.12875
H	5.33001	2.09971	-0.76074	H	5.44439	-0.43039	2.33039

atom	Con f. 2- 1i			atom	Con f. 2- 1j		
C	-5.52993	-2.04629	-0.21412	C	-5.9457	-1.24735	0.45882
C	-4.75083	1.95443	-1.46272	C	-3.51371	2.50829	0.91522
C	-3.74749	1.76016	-0.59809	C	-3.76273	1.75826	-0.16521
C	-2.98229	0.44237	-0.59143	C	-2.94051	0.52802	-0.52579
C	-3.50253	-0.62859	0.44475	C	-3.44531	-0.79545	0.16576
C	-4.96873	-0.90013	0.1744	C	-4.85862	-1.07804	-0.29544
C	-1.46611	0.6741	-0.45333	C	-1.43344	0.74232	-0.29927
C	-0.65938	-0.62283	-0.62026	C	-0.5955	-0.43678	-0.82129
C	-1.14985	-1.67186	0.39663	C	-1.0655	-1.74901	-0.1626
C	-2.65485	-1.90444	0.25337	C	-2.56662	-1.96129	-0.36773
C	-3.40364	-0.14394	1.90763	C	-3.35508	-0.72327	1.69438
C	-3.32379	2.86219	0.34273	C	-4.87349	2.11792	-1.118
C	0.84144	-0.40929	-0.56531	C	0.89944	-0.21337	-0.68648

C	1.67336	-1.02975	-1.41111	C	1.74599	-0.49608	-1.68549
C	3.16945	-0.91136	-1.34775	C	3.2399	-0.35716	-1.58003
C	3.59887	-0.33292	-0.00656	C	3.63987	-0.24854	-0.11725
O	2.7775	0.81824	0.27674	O	2.79723	0.73374	0.52015
C	1.4021	0.53862	0.47314	C	1.43294	0.35637	0.61246
O	1.14806	0.10519	1.80316	O	1.20739	-0.50046	1.7231
C	5.05398	0.17034	0.05904	C	5.08675	0.16694	0.19659
O	5.21545	1.25443	-0.87681	O	5.21825	0.16023	1.63396
C	6.03369	-0.91655	-0.37246	C	5.42794	1.56301	-0.33388
C	5.38087	0.66052	1.47386	C	6.06985	-0.87662	-0.32337
H	-3.15175	-0.02127	-1.57154	H	-3.07782	0.35951	-1.60321
H	-0.88027	-1.02386	-1.61926	H	-0.79901	-0.53275	-1.89692
H	3.45326	-1.08343	0.78238	H	3.4754	-1.21879	0.37179
H	-6.60468	-2.11491	-0.36384	H	-6.91009	-1.46477	0.00676
H	-4.96206	-2.95332	-0.39802	H	-5.92613	-1.17629	1.54225
H	-5.30914	2.88738	-1.49001	H	-4.11718	3.3838	1.14277
H	-5.04952	1.18551	-2.17073	H	-2.70761	2.28492	1.60688
H	-5.61778	-0.04106	0.33665	H	-4.96118	-1.16498	-1.37847
H	-1.24999	1.11614	0.52394	H	-1.2369	0.8899	0.76631
H	-1.14909	1.40567	-1.20622	H	-1.12856	1.66578	-0.8045
H	-0.61367	-2.61391	0.23354	H	-0.51229	-2.5904	-0.59609
H	-0.90679	-1.34097	1.41082	H	-0.82653	-1.7309	0.90463
H	-2.84746	-2.30948	-0.74821	H	-2.76364	-2.08116	-1.44153
H	-2.98577	-2.66515	0.97005	H	-2.88582	-2.89222	0.11574
H	-2.40655	0.2241	2.16121	H	-2.3394	-0.50843	2.03319
H	-4.12014	0.65647	2.11115	H	-4.00914	0.05362	2.09679
H	-3.63685	-0.97185	2.58619	H	-3.65244	-1.68013	2.13557
H	-3.28297	2.53042	1.3842	H	-5.59807	1.30164	-1.20769
H	-2.31963	3.22628	0.09452	H	-5.40741	3.01697	-0.79627
H	-4.0106	3.71111	0.28138	H	-4.47482	2.29548	-2.12548
H	1.25839	-1.66934	-2.18882	H	1.34637	-0.85543	-2.6325
H	3.53904	-0.27316	-2.16196	H	3.57641	0.52144	-2.14649
H	3.6212	-1.89868	-1.49266	H	3.7259	-1.227	-2.03564
H	0.92137	1.51756	0.40132	H	0.91869	1.28668	0.86591
H	1.33757	-0.84429	1.85368	H	1.41333	-1.40894	1.45394
H	4.50055	1.88266	-0.68708	H	4.44331	0.63969	1.9696
H	5.88422	-1.19284	-1.4193	H	4.71925	2.30634	0.04187
H	7.06034	-0.55498	-0.25936	H	6.43193	1.84135	0.00026
H	5.91558	-1.81159	0.24548	H	5.41124	1.59503	-1.42735
H	5.32538	-0.16154	2.19516	H	7.07885	-0.6365	0.02615
H	6.39393	1.07292	1.49699	H	5.80803	-1.87496	0.0417
H	4.67976	1.43775	1.78906	H	6.08365	-0.89555	-1.41618

atom	Con f. 2- 1k			atom	Con f. 2- 1l		
C	6.04488	-1.25252	-0.36387	C	6.04844	-1.22582	-0.44701
C	3.75362	2.6136	-0.61618	C	3.7421	2.66021	-0.44556
C	3.84605	1.71579	0.37229	C	3.84647	1.69762	0.47882
C	2.96416	0.47675	0.45049	C	2.9686	0.45316	0.48154
C	3.53479	-0.75766	-0.34772	C	3.54015	-0.72529	-0.3963
C	4.86712	-1.14943	0.25379	C	4.87201	-1.15717	0.17793
C	1.50078	0.76945	0.07203	C	1.50361	0.76497	0.12646
C	0.59293	-0.43808	0.33172	C	0.59745	-0.45747	0.30764
C	1.11212	-1.65999	-0.45133	C	1.11712	-1.62108	-0.55986
C	2.57341	-1.95542	-0.10672	C	2.57922	-1.93674	-0.23674
C	3.6567	-0.47297	-1.84885	C	3.66207	-0.3415	-1.87539
C	4.82471	1.90649	1.50224	C	4.83627	1.8146	1.60912
C	-0.8723	-0.18502	0.04357	C	-0.86935	-0.1829	0.04769
C	-1.34046	0.65979	-0.8832	C	-1.34377	0.77806	-0.75377
C	-2.80833	0.86789	-1.14037	C	-2.81154	1.02305	-0.97189
C	-3.62126	0.34664	0.03797	C	-3.63667	0.33041	0.10697
O	-3.16251	-0.97275	0.3787	O	-3.15561	-1.01873	0.26063
C	-1.83666	-1.00198	0.87598	C	-1.83421	-1.10862	0.75751
O	-1.78526	-0.62739	2.24782	O	-1.78917	-0.92718	2.16926
C	-5.14452	0.25669	-0.1714	C	-5.14687	0.25042	-0.16843
O	-5.5229	1.60392	-0.51668	O	-5.29181	-0.49857	-1.39157
C	-5.82552	-0.14597	1.13936	C	-5.7312	1.65441	-0.34883
C	-5.53139	-0.7018	-1.29935	C	-5.85912	-0.48497	0.97096
H	2.95785	0.15839	1.50248	H	2.96737	0.06855	1.51114
H	0.67033	-0.68193	1.40088	H	0.67954	-0.77621	1.35687
H	-3.4545	1.01495	0.89458	H	-3.50858	0.86652	1.05768
H	6.9343	-1.56166	0.1793	H	6.93754	-1.57182	0.07402
H	6.17638	-1.03557	-1.41983	H	6.17886	-0.94383	-1.48763
H	4.3948	3.49162	-0.63955	H	4.38095	3.53971	-0.41531
H	3.04367	2.51365	-1.43128	H	3.0253	2.6128	-1.25922

H	4.81865	-1.38505	1.31831	H	4.82421	-1.45908	1.22559
H	1.43886	1.05984	-0.98076	H	1.4391	1.12174	-0.90565
H	1.14794	1.62865	0.65298	H	1.15086	1.58492	0.7617
H	0.50078	-2.54158	-0.22661	H	0.51034	-2.5189	-0.39729
H	0.99778	-1.46576	-1.52449	H	1.00178	-1.35286	-1.61687
H	2.6315	-2.23997	0.95235	H	2.638	-2.29354	0.80018
H	2.93256	-2.81616	-0.68312	H	2.93911	-2.75582	-0.87058
H	2.69838	-0.18564	-2.28639	H	2.70511	-0.01909	-2.29106
H	4.36546	0.33451	-2.04569	H	4.37596	0.47268	-2.0194
H	4.00386	-1.36723	-2.37633	H	4.00242	-1.2009	-2.46202
H	5.51013	1.05601	1.58038	H	5.52993	0.96716	1.6176
H	5.41889	2.81615	1.37418	H	5.42146	2.73646	1.54097
H	4.29613	1.97239	2.46236	H	4.31835	1.80577	2.57719
H	-0.6488	1.23025	-1.49801	H	-0.65703	1.43242	-1.28447
H	-3.0946	0.36083	-2.0715	H	-3.11516	0.66632	-1.96492
H	-3.0195	1.93167	-1.28903	H	-3.00618	2.10076	-0.95484
H	-1.56226	-2.06127	0.86186	H	-1.55628	-2.15543	0.60143
H	-1.7401	0.34036	2.29311	H	-1.7429	0.0243	2.34935
H	-6.48412	1.60672	-0.63715	H	-6.23836	-0.52609	-1.5955
H	-5.57013	0.56259	1.934	H	-5.30885	2.14824	-1.22772
H	-6.91371	-0.14716	1.01364	H	-6.81596	1.58791	-0.48576
H	-5.51624	-1.14576	1.45477	H	-5.5408	2.28023	0.52902
H	-6.62238	-0.75631	-1.38133	H	-5.73624	0.04901	1.91903
H	-5.13706	-0.36187	-2.26053	H	-6.93123	-0.55331	0.75749
H	-5.15436	-1.7095	-1.10495	H	-5.4605	-1.49484	1.08737

atom	Con f. 2- 2a			atom	Con f. 2- 2b		
C	-5.96384	-1.21898	0.75257	C	-5.39598	-2.20933	-0.0333
C	-3.77947	2.66526	0.32617	C	-4.90328	1.77366	-1.50048
C	-3.90069	1.64855	-0.53597	C	-3.90679	1.71633	-0.60857
C	-2.99265	0.4261	-0.50975	C	-3.04458	0.46434	-0.49788
C	-3.47488	-0.69762	0.48376	C	-3.52244	-0.57908	0.58592
C	-4.83631	-1.18701	0.04035	C	-4.94789	-0.99213	0.27892
C	-1.51747	0.79588	-0.27253	C	-1.5563	0.82399	-0.3264
C	-0.58538	-0.41021	-0.4206	C	-0.64269	-0.40251	-0.37684
C	-1.0267	-1.53733	0.53624	C	-1.09013	-1.4256	0.68689
C	-2.49973	-1.8966	0.33013	C	-2.5662	-1.78638	0.51367
C	-3.49541	-0.21819	1.93965	C	-3.52937	0.00557	2.01476
C	-4.94535	1.67484	-1.62166	C	-3.59018	2.90603	0.26467
C	0.88234	-0.07658	-0.24075	C	0.83218	-0.07409	-0.24935
C	1.35206	1.11098	0.16121	C	1.32655	1.14642	-0.00746
C	2.80936	1.3982	0.39561	C	2.79173	1.43332	0.17452
C	3.61684	0.10444	0.44067	C	3.56768	0.14058	0.40306
O	3.1985	-0.73752	-0.64677	O	3.13547	-0.82951	-0.56598
C	1.85909	-1.20653	-0.52285	C	1.78486	-1.24919	-0.39761
O	1.56017	-1.90306	-1.70647	O	1.47316	-2.06601	-1.4987
C	5.13915	0.28265	0.27372	C	5.09551	0.25247	0.22736
O	5.40192	0.8541	-1.02368	O	5.38009	0.60281	-1.14134
C	5.69312	1.27098	1.29559	C	5.67121	1.37458	1.08554
C	5.8496	-1.07001	0.38934	C	5.76626	-1.08367	0.56376
H	-3.04747	-0.03084	-1.5076	H	-3.14295	-0.06795	-1.45237
H	-0.70795	-0.80039	-1.44159	H	-0.78489	-0.88636	-1.35423
H	3.42517	-0.41593	1.39063	H	3.35572	-0.24083	1.41265
H	-6.88311	-1.61114	0.32499	H	-6.45352	-2.3808	-0.21895
H	-6.02248	-0.86072	1.77593	H	-4.74852	-3.07708	-0.11644
H	-4.44273	3.52557	0.27654	H	-5.5318	2.65575	-1.59897
H	-3.02297	2.68316	1.10426	H	-5.12576	0.93862	-2.16017
H	-4.859	-1.56624	-0.98271	H	-5.67049	-0.18021	0.34257
H	-1.39478	1.23102	0.7242	H	-1.40591	1.34737	0.62374
H	-1.2336	1.5749	-0.98845	H	-1.27478	1.52856	-1.11742
H	-0.41984	-2.43358	0.3712	H	-0.49269	-2.33953	0.60969
H	-0.8452	-1.21965	1.57026	H	-0.9041	-1.009	1.68463
H	-2.6188	-2.31421	-0.67855	H	-2.68667	-2.27297	-0.46245
H	-2.79667	-2.68476	1.03215	H	-2.85997	-2.52289	1.27058
H	-4.22141	0.58462	2.08621	H	-4.30957	0.76268	2.13153
H	-3.76251	-1.04465	2.60619	H	-3.73529	-0.79119	2.73788
H	-2.52016	0.15714	2.25624	H	-2.5733	0.46158	2.28376
H	-4.47351	1.62053	-2.6116	H	-2.61814	3.3394	0.00035
H	-5.61661	0.81233	-1.54785	H	-4.34659	3.68727	0.14753
H	-5.55107	2.58494	-1.57956	H	-3.53251	2.6414	1.32431
H	0.66434	1.93114	0.34798	H	0.65563	1.99629	0.08002
H	2.92675	1.94679	1.33681	H	2.92873	2.11039	1.025
H	3.20201	2.04909	-0.39689	H	3.19297	1.9515	-0.70672
H	1.82397	-1.96368	0.27357	H	1.73009	-1.91233	0.47746

H	1.46826	-1.24783	-2.41697	H	1.41025	-1.4924	-2.27967
H	4.92866	0.30059	-1.6649	H	4.88937	-0.03055	-1.68907
H	5.2656	2.26728	1.15746	H	6.76029	1.39715	0.98097
H	6.77893	1.34796	1.1831	H	5.43172	1.21624	2.14128
H	5.47712	0.93584	2.31434	H	5.28025	2.34859	0.78086
H	6.92216	-0.94017	0.21692	H	5.60891	-1.34685	1.61498
H	5.70928	-1.50258	1.3855	H	5.36006	-1.88878	-0.05437
H	5.46094	-1.7776	-0.34779	H	6.84286	-1.01339	0.38211

atom	Con f. 2- 2c			atom	Con f. 2- 2d		
C	-6.04968	-1.21934	-0.25789	C	-5.95404	-1.23306	0.74868
C	-3.56712	1.81459	1.9348	C	-3.78366	2.65947	0.36929
C	-3.72268	1.61043	0.62109	C	-3.90616	1.6557	-0.50775
C	-2.90963	0.5862	-0.15962	C	-2.99433	0.43582	-0.50465
C	-3.51956	-0.8657	-0.12726	C	-3.46725	-0.70364	0.4751
C	-4.88478	-0.83135	-0.77923	C	-4.82973	-1.19042	0.03199
C	-1.4232	0.58953	0.24145	C	-1.51893	0.80708	-0.27127
C	-0.57006	-0.33874	-0.64498	C	-0.58347	-0.39374	-0.44014
C	-1.14641	-1.76195	-0.62619	C	-1.0163	-1.53583	0.50266
C	-2.6237	-1.76525	-1.0244	C	-2.48913	-1.89692	0.29836
C	-3.58662	-1.43713	1.29352	C	-3.48104	-0.24589	1.93794
C	-4.71029	2.42276	-0.17647	C	-4.95585	1.6957	-1.5881
C	0.89632	-0.27971	-0.25974	C	0.88433	-0.05697	-0.26451
C	1.43259	-0.94859	0.76732	C	1.35036	1.13481	0.13063
C	2.90192	-0.93203	1.08657	C	2.80926	1.43039	0.3529
C	3.69566	-0.41908	-0.11111	C	3.60524	0.13191	0.4069
O	3.07453	0.78461	-0.59558	O	3.19806	-0.71374	-0.68292
C	1.76546	0.61006	-1.12319	C	1.86112	-1.18879	-0.5466
O	1.22467	1.90081	-1.28687	O	1.55563	-1.89279	-1.7235
C	5.15863	-0.03629	0.19146	C	5.13642	0.24889	0.31162
O	5.16713	1.0511	1.13763	O	5.66763	-1.08802	0.42614
C	5.90505	-1.18878	0.85632	C	5.59659	0.85884	-1.01635
C	5.87139	0.39899	-1.09341	C	5.68959	1.02728	1.50044
H	-2.94834	0.8896	-1.21527	H	-3.05363	-0.0068	-1.50867
H	-0.65553	0.03417	-1.67549	H	-0.70975	-0.77035	-1.46588
H	3.68523	-1.1762	-0.90832	H	3.37865	-0.3877	1.35009
H	-6.96755	-1.16058	-0.83731	H	-6.87454	-1.62142	0.32028
H	-6.1416	-1.60502	0.75314	H	-6.00875	-0.88759	1.77664
H	-4.16082	2.55775	2.46174	H	-4.44961	3.51851	0.33618
H	-2.85032	1.2588	2.53098	H	-3.02346	2.66764	1.14397
H	-4.87587	-0.45461	-1.80355	H	-4.85641	-1.55685	-0.99564
H	-1.30067	0.28593	1.28613	H	-1.39133	1.23037	0.73001
H	-1.04487	1.61406	0.16932	H	-1.2424	1.59595	-0.97921
H	-0.57775	-2.39928	-1.31345	H	-0.4078	-2.42767	0.32123
H	-1.02976	-2.1989	0.37136	H	-0.83061	-1.23326	1.54044
H	-2.71123	-1.41694	-2.06232	H	-2.61182	-2.29934	-0.71606
H	-3.01919	-2.78764	-1.00294	H	-2.77979	-2.69678	0.98968
H	-2.60743	-1.43614	1.7765	H	-2.5049	0.12669	2.25492
H	-4.26593	-0.8603	1.92498	H	-4.20793	0.55302	2.10034
H	-3.94163	-2.47248	1.26864	H	-3.74286	-1.0828	2.59343
H	-5.24166	3.14449	0.45104	H	-5.56522	2.60231	-1.52781
H	-4.19936	2.97302	-0.9775	H	-4.48825	1.66042	-2.58092
H	-5.4516	1.77918	-0.66256	H	-5.62295	0.82907	-1.52599
H	0.799	-1.56681	1.39974	H	0.6601	1.95287	0.31627
H	3.22978	-1.94389	1.34865	H	2.93576	1.98317	1.2907
H	3.10621	-0.29852	1.95989	H	3.18188	2.08567	-0.4452
H	1.83318	0.19994	-2.14295	H	1.83287	-1.94005	0.25545
H	1.09546	2.27337	-0.39952	H	1.46273	-1.24262	-2.43848
H	4.57121	1.72652	0.77625	H	5.15893	-1.62499	-0.20271
H	6.95196	-0.91031	1.01105	H	5.29261	1.90526	-1.11002
H	5.8779	-2.08239	0.22574	H	5.18179	0.3039	-1.86264
H	5.47129	-1.43428	1.82888	H	6.68824	0.81488	-1.0743
H	6.89107	0.71912	-0.85953	H	6.78327	0.98724	1.48857
H	5.9236	-0.42665	-1.81084	H	5.38682	2.07673	1.46045
H	5.34482	1.23115	-1.56821	H	5.33968	0.59711	2.4444

atom	Con f. 2- 2e			atom	Con f. 2- 2f		
C	-6.00086	-0.97427	0.46887	C	-5.50297	-1.75278	-1.20528
C	-3.35269	2.60703	0.72914	C	-4.6399	2.37533	-0.53577
C	-3.62204	1.81568	-0.31638	C	-3.72656	1.79673	0.25358
C	-2.87499	0.5158	-0.58583	C	-2.95412	0.57574	-0.23082
C	-3.47219	-0.72736	0.17669	C	-3.57063	-0.81574	0.18781
C	-4.89856	-0.93884	-0.28162	C	-4.99674	-0.89423	-0.31844
C	-1.36119	0.65085	-0.34498	C	-1.46172	0.66947	0.13661

C	-0.58828	-0.61394	-0.75956	C	-0.62819	-0.46995	-0.47847
C	-1.16143	-1.85701	-0.04977	C	-1.21622	-1.82629	-0.06333
C	-2.66912	-1.97734	-0.27897	C	-2.69465	-1.91868	-0.44399
C	-3.38499	-0.57205	1.69944	C	-3.63319	-1.00288	1.71931
C	-4.67975	2.19344	-1.32103	C	-3.41139	2.37452	1.61201
C	0.91202	-0.47076	-0.57955	C	0.84536	-0.32169	-0.14934
C	1.77376	-0.88296	-1.51763	C	1.3983	-0.68006	1.01498
C	3.2634	-0.70723	-1.4259	C	2.87406	-0.58814	1.28938
C	3.60545	0.33301	-0.36362	C	3.64494	-0.45203	-0.02009
O	2.84564	0.0455	0.82262	O	3.02454	0.57306	-0.81627
C	1.43657	0.19254	0.68085	C	1.69959	0.2831	-1.24318
O	0.868	-0.31383	1.86548	O	1.16635	1.4905	-1.7371
C	5.08045	0.34749	0.08606	C	5.11789	-0.02137	0.13025
O	5.38832	-0.91793	0.70275	O	5.15918	1.29349	0.71889
C	6.02233	0.4843	-1.1061	C	5.86902	-0.9429	1.0863
C	5.31721	1.47838	1.09267	C	5.80537	0.00959	-1.23923
H	-3.00791	0.28865	-1.65295	H	-3.01545	0.58347	-1.32655
H	-0.76119	-0.76152	-1.83462	H	-0.73084	-0.39041	-1.56986
H	3.3365	1.33421	-0.73023	H	3.60961	-1.40537	-0.56686
H	-6.97556	-1.1501	0.02081	H	-6.5548	-1.71333	-1.47785
H	-5.98385	-0.83058	1.54511	H	-4.91048	-2.52149	-1.69236
H	-3.90286	3.53101	0.89052	H	-5.20155	3.24942	-0.21421
H	-2.58189	2.37136	1.45584	H	-4.85853	1.99176	-1.5292
H	-4.99983	-1.0918	-1.35748	H	-5.66629	-0.15665	0.12116
H	-1.16992	0.86953	0.70826	H	-1.32989	0.65294	1.22354
H	-0.9906	1.51031	-0.91517	H	-1.07382	1.63317	-0.21002
H	-0.65821	-2.75316	-0.43096	H	-0.65759	-2.63508	-0.54858
H	-0.94747	-1.80767	1.02137	H	-1.09665	-1.96926	1.01608
H	-2.85464	-2.13838	-1.34944	H	-2.77592	-1.85099	-1.53625
H	-3.06109	-2.85806	0.24355	H	-3.09356	-2.89913	-0.15886
H	-3.98558	0.2701	2.05059	H	-4.37556	-0.33915	2.17103
H	-3.75004	-1.47886	2.19273	H	-3.92779	-2.03161	1.95413
H	-2.35838	-0.40763	2.03338	H	-2.67251	-0.81186	2.20417
H	-5.44972	1.41819	-1.39671	H	-3.47329	1.62631	2.40755
H	-5.16786	3.13725	-1.06036	H	-2.39083	2.77462	1.64175
H	-4.24063	2.29763	-2.322	H	-4.09704	3.19078	1.85618
H	1.38502	-1.35351	-2.41987	H	0.77493	-1.08843	1.80764
H	3.65588	-0.39465	-2.39967	H	3.20243	-1.48417	1.82714
H	3.75354	-1.65844	-1.17916	H	3.10042	0.26906	1.93732
H	1.18741	1.26341	0.68154	H	1.73348	-0.38933	-2.11448
H	1.00841	-1.27405	1.86772	H	1.06884	2.09165	-0.98068
H	4.69536	-1.06964	1.36514	H	4.56177	1.84406	0.18807
H	5.94337	-0.3744	-1.7775	H	6.92288	-0.65114	1.12813
H	7.05656	0.54601	-0.75375	H	5.81437	-1.98127	0.74612
H	5.79908	1.39294	-1.67317	H	5.46027	-0.88449	2.09808
H	6.35142	1.44673	1.44809	H	5.83464	-0.99036	-1.68462
H	5.13894	2.45575	0.63215	H	5.27582	0.67432	-1.9269
H	4.64954	1.37896	1.95264	H	6.8328	0.36903	-1.12883

atom	Con f. 2- 2g			atom	Con f. 2- 2h		
C	-5.38625	-2.21681	-0.00574	C	-5.97132	-1.21181	0.729
C	-4.91319	1.75766	-1.50272	C	-3.78115	2.66984	0.3346
C	-3.91261	1.70989	-0.61483	C	-3.9005	1.65871	-0.5343
C	-3.0453	0.46182	-0.49966	C	-2.99353	0.43543	-0.5131
C	-3.51437	-0.5768	0.59275	C	-3.48033	-0.69499	0.4704
C	-4.94	-0.99619	0.29548	C	-4.84169	-1.17826	0.0202
C	-1.55786	0.82796	-0.33598	C	-1.51875	0.80181	-0.268
C	-0.64075	-0.39589	-0.38612	C	-0.58794	-0.40447	-0.423
C	-1.0786	-1.41489	0.68566	C	-1.03388	-1.5396	0.522
C	-2.5545	-1.78154	0.52313	C	-2.50702	-1.8945	0.3092
C	-3.51644	0.01667	2.01797	C	-3.50338	-0.2267	1.9298
C	-3.59613	2.90669	0.24874	C	-4.94094	1.69318	-1.6238
C	0.83387	-0.06358	-0.26754	C	0.87882	-0.07445	-0.2362
C	1.32239	1.15547	-0.00497	C	1.35243	1.11676	0.1494
C	2.78851	1.4477	0.17013	C	2.80989	1.40645	0.3856
C	3.55837	0.14765	0.36626	C	3.62526	0.11676	0.4386
O	3.13461	-0.79904	-0.63013	O	3.19402	-0.74093	-0.6307
C	1.7898	-1.23393	-0.44569	C	1.85822	-1.21087	-0.4624
O	1.46576	-2.03819	-1.55149	O	1.50985	-1.89442	-1.6381
C	5.09205	0.22136	0.26671	C	5.14388	0.30138	0.249
O	5.5942	-1.10844	0.51464	O	5.38631	0.86098	-1.0573
C	5.5709	0.68814	-1.11181	C	5.70594	1.3028	1.2537
C	5.65907	1.10029	1.37642	C	5.86371	-1.04604	0.3687
H	-3.14541	-0.0768	-1.45041	H	-3.04502	-0.01439	-1.5143

H	-0.78708	-0.8844	-1.36038	H	-0.7064	-0.78396	-1.4481
H	3.31835	-0.26386	1.35815	H	3.44933	-0.39104	1.3986
H	-6.44426	-2.39241	-0.1847	H	-6.89039	-1.59889	0.2964
H	-4.73687	-3.08339	-0.086	H	-6.03195	-0.85963	1.7544
H	-5.54522	2.63688	-1.60444	H	-4.44328	3.5312	0.2881
H	-5.13565	0.91739	-2.15574	H	-3.02698	2.68211	1.115
H	-5.66457	-0.18588	0.35708	H	-4.86242	-1.55107	-1.0052
H	-1.40625	1.35518	0.61174	H	-1.39795	1.22986	0.732
H	-1.2813	1.53053	-1.13055	H	-1.23241	1.58554	-0.9777
H	-0.47797	-2.32705	0.61075	H	-0.42782	-2.43473	0.3481
H	-0.88815	-0.99159	1.6797	H	-0.85487	-1.23308	1.5598
H	-2.67903	-2.27502	-0.449	H	-2.62434	-2.30315	-0.7032
H	-2.84156	-2.51383	1.28667	H	-2.80722	-2.68804	1.0038
H	-4.29752	0.77309	2.13323	H	-4.22742	0.57714	2.0805
H	-3.71792	-0.77595	2.74685	H	-3.7748	-1.05734	2.5894
H	-2.56014	0.47601	2.28027	H	-2.52794	0.14338	2.2519
H	-3.53302	2.64968	1.30995	H	-4.46491	1.64651	-2.6122
H	-2.62662	3.34125	-0.02289	H	-5.61245	0.83012	-1.5595
H	-4.35548	3.68472	0.12939	H	-5.54684	2.60295	-1.5773
H	0.64716	1.99947	0.1028	H	0.66721	1.94295	0.3168
H	2.93428	2.10256	1.03664	H	2.92487	1.96072	1.3239
H	3.16674	1.99733	-0.7016	H	3.20032	2.05489	-0.4099
H	1.75279	-1.90736	0.42237	H	1.85084	-1.90324	0.3957
H	1.38871	-1.45552	-2.32435	H	2.0176	-2.7195	-1.6593
H	5.07698	-1.69483	-0.06093	H	4.89893	0.30521	-1.686
H	5.28996	1.72663	-1.3092	H	5.50957	0.97423	2.2786
H	5.14869	0.0618	-1.90294	H	5.26768	2.29426	1.1146
H	6.66155	0.61565	-1.15785	H	6.78906	1.38804	1.1227
H	6.75181	1.03868	1.37128	H	6.93371	-0.91123	0.1846
H	5.37624	2.1464	1.23429	H	5.73596	-1.47146	1.3696
H	5.3003	0.76993	2.35652	H	5.4725	-1.76193	-0.359

atom	Con f. 2- 2i			atom	Con f. 2- 2j		
C	-6.04265	-1.24578	-0.25866	C	-5.6251	-1.83699	-0.36864
C	-3.6009	1.86545	1.87489	C	-4.573	2.12678	-1.47956
C	-3.73907	1.62005	0.56634	C	-3.60331	1.85287	-0.59819
C	-2.91177	0.57659	-0.17274	C	-2.91513	0.49327	-0.61
C	-3.51601	-0.87632	-0.10409	C	-3.52691	-0.57542	0.37734
C	-4.87236	-0.86822	-0.77557	C	-5.00145	-0.74305	0.07185
C	-1.43001	0.59971	0.24423	C	-1.39334	0.63292	-0.42098
C	-0.56412	-0.34963	-0.60692	C	-0.65415	-0.7053	-0.59094
C	-1.13221	-1.77513	-0.54705	C	-1.24742	-1.76623	0.35657
C	-2.60491	-1.7993	-0.96118	C	-2.75802	-1.89509	0.15486
C	-3.60074	-1.4044	1.33239	C	-3.42478	-0.14602	1.8574
C	-4.72076	2.40121	-0.26858	C	-3.1413	2.90237	0.38371
C	0.89981	-0.26776	-0.21668	C	0.85068	-0.55907	-0.46579
C	1.43722	-0.91205	0.82593	C	1.68504	-1.11421	-1.3539
C	2.90655	-0.87312	1.15086	C	3.17761	-0.94113	-1.33017
C	3.68288	-0.3893	-0.06824	C	3.55843	0.23112	-0.4302
O	3.06692	0.81199	-0.56696	O	2.82414	0.12156	0.80055
C	1.76167	0.61407	-1.09812	C	1.41652	0.28521	0.66266
O	1.20858	1.89517	-1.2897	O	0.86152	0.00054	1.92448
C	5.17043	-0.05247	0.13759	C	5.04573	0.2855	-0.02734
O	5.68934	0.35697	-1.14496	O	5.36916	-0.90317	0.72088
C	5.38305	1.07844	1.14872	C	5.94854	0.27376	-1.25705
C	5.9539	-1.30062	0.53004	C	5.31996	1.52303	0.83385
H	-2.93948	0.84769	-1.23745	H	-3.08154	0.0691	-1.60841
H	-0.64586	-0.01003	-1.64924	H	-0.84876	-1.05685	-1.61362
H	3.63021	-1.15849	-0.85286	H	3.2913	1.17612	-0.92528
H	-6.95266	-1.20908	-0.85214	H	-6.69894	-1.83295	-0.53917
H	-6.14678	-1.60146	0.76213	H	-5.11084	-2.77058	-0.57611
H	-4.20493	2.62125	2.37132	H	-5.07795	3.08993	-1.49289
H	-2.88861	1.33208	2.49655	H	-4.89743	1.39488	-2.21504
H	-4.85093	-0.52247	-1.81057	H	-5.59833	0.14919	0.25382
H	-1.31638	0.32742	1.29852	H	-1.17825	1.04659	0.56797
H	-1.05666	1.62376	0.14594	H	-1.0117	1.35543	-1.15244
H	-0.55268	-2.43139	-1.20681	H	-0.76527	-2.73172	0.16415
H	-1.02425	-2.17813	0.4657	H	-1.02695	-1.5057	1.39497
H	-2.68217	-1.48509	-2.01072	H	-2.94547	-2.24375	-0.86871
H	-2.99541	-2.82262	-0.91131	H	-3.15889	-2.66227	0.8276
H	-2.62848	-1.38496	1.82892	H	-4.09163	0.6927	2.0752
H	-4.29103	-0.81129	1.93631	H	-3.72338	-0.97807	2.50459
H	-3.95163	-2.44143	1.33399	H	-2.41069	0.14799	2.13859
H	-5.44977	1.73759	-0.74618	H	-3.13708	2.53657	1.41457

H	-5.26681	3.13683	0.32954	H	-2.11507	3.22062	0.16435
H	-4.2022	2.93167	-1.07803	H	-3.78178	3.78769	0.33762
H	0.80656	-1.5229	1.46803	H	1.26971	-1.71017	-2.16565
H	3.24775	-1.87364	1.43909	H	3.54225	-0.76898	-2.34879
H	3.08989	-0.21928	2.01337	H	3.66979	-1.8542	-0.96989
H	1.83613	0.1823	-2.10828	H	1.19615	1.3486	0.48957
H	1.08093	2.28737	-0.41059	H	0.98923	-0.94659	2.09333
H	5.0652	1.02132	-1.47911	H	4.70825	-0.96821	1.42834
H	6.44204	1.35271	1.16481	H	6.99466	0.35993	-0.94778
H	5.09378	0.77891	2.16007	H	5.71506	1.11517	-1.91604
H	4.80047	1.96192	0.87249	H	5.8357	-0.65487	-1.82198
H	7.02406	-1.07203	0.54589	H	5.12999	2.44212	0.26968
H	5.66578	-1.65187	1.52405	H	4.68206	1.52986	1.72172
H	5.78515	-2.1082	-0.18956	H	6.36568	1.52498	1.15553

atom	Con f. 2- 2k			atom	Con f. 2- 2l		
C	-5.40367	-2.19677	-0.0795	C	-5.48429	-1.81099	-1.16197
C	-4.89954	1.80682	-1.48705	C	-4.65959	2.34618	-0.59211
C	-3.90584	1.73263	-0.59327	C	-3.74197	1.79454	0.21143
C	-3.0468	0.47709	-0.50059	C	-2.95875	0.56889	-0.24356
C	-3.52752	-0.58065	0.56769	C	-3.56426	-0.81712	0.20803
C	-4.95375	-0.98585	0.25389	C	-4.98772	-0.92106	-0.30103
C	-1.55759	0.83077	-0.32403	C	-1.46752	0.68458	0.1222
C	-0.64585	-0.39672	-0.38905	C	-0.62303	-0.4646	-0.45836
C	-1.09764	-1.4338	0.65923	C	-1.20068	-1.81425	-0.00843
C	-2.57401	-1.78892	0.47766	C	-2.67678	-1.92851	-0.39222
C	-3.53332	-0.01743	2.00519	C	-3.6306	-0.96536	1.74375
C	-3.58886	2.90773	0.29937	C	-3.43355	2.40784	1.55582
C	0.82837	-0.07102	-0.25372	C	0.84724	-0.29328	-0.12552
C	1.32626	1.15138	-0.03016	C	1.38847	-0.5879	1.06239
C	2.79116	1.4419	0.15649	C	2.86202	-0.46928	1.3471
C	3.57525	0.15547	0.39954	C	3.62842	-0.40969	0.03122
O	3.13148	-0.83131	-0.54656	O	3.02394	0.58496	-0.81579
C	1.78394	-1.24621	-0.3338	C	1.70721	0.25778	-1.24488
O	1.42804	-2.06108	-1.42024	O	1.16715	1.42848	-1.81211
C	5.0999	0.27008	0.20221	C	5.12343	-0.05198	0.10454
O	5.36559	0.60293	-1.17462	O	5.62516	-0.07545	-1.24833
C	5.68294	1.40507	1.03825	C	5.36875	1.33536	0.70595
C	5.7796	-1.05944	0.5471	C	5.89757	-1.12715	0.85956
H	-3.14632	-0.04117	-1.46261	H	-3.01951	0.54997	-1.33919
H	-0.78491	-0.86582	-1.37386	H	-0.72136	-0.41651	-1.55164
H	3.37806	-0.20846	1.41876	H	3.55297	-1.3859	-0.47002
H	-6.46137	-2.36347	-0.26855	H	-6.53523	-1.78775	-1.43981
H	-4.75732	-3.06383	-0.17748	H	-4.88426	-2.59041	-1.62223
H	-5.52551	2.69201	-1.57328	H	-5.22909	3.22281	-0.29196
H	-5.12199	0.98316	-2.16088	H	-4.87397	1.93692	-1.57615
H	-5.67501	-0.17391	0.33124	H	-5.66416	-0.17493	0.11286
H	-1.40545	1.34252	0.63227	H	-1.33652	0.70001	1.20928
H	-1.27549	1.5443	-1.10669	H	-1.08649	1.6413	-0.25126
H	-0.50151	-2.34726	0.56852	H	-0.63349	-2.63067	-0.47043
H	-0.91387	-1.03253	1.66367	H	-1.08332	-1.92744	1.07482
H	-2.69367	-2.25942	-0.50643	H	-2.75424	-1.88919	-1.4862
H	-2.8709	-2.53684	1.2221	H	-3.06883	-2.90476	-0.0841
H	-4.31407	0.73711	2.13435	H	-4.38135	-0.29772	2.17539
H	-3.73766	-0.8251	2.71658	H	-3.9155	-1.9906	2.00427
H	-2.5774	0.4356	2.2798	H	-2.67361	-0.75163	2.22635
H	-3.53417	2.62657	1.35489	H	-3.49331	1.67966	2.36996
H	-2.61539	3.3433	0.04408	H	-2.41543	2.81456	1.57815
H	-4.34345	3.69223	0.19271	H	-4.12473	3.22582	1.77753
H	0.65767	2.00491	0.03329	H	0.75776	-0.95596	1.86873
H	2.92298	2.12786	1.00079	H	3.19635	-1.33022	1.93658
H	3.19235	1.95375	-0.72844	H	3.0635	0.42343	1.95335
H	1.75619	-1.82803	0.60237	H	1.75852	-0.46353	-2.07514
H	1.91619	-2.89399	-1.33373	H	1.05153	2.07039	-1.09271
H	4.86103	-0.03218	-1.70762	H	4.9987	0.45427	-1.76759
H	5.4615	1.25738	2.09946	H	5.09545	1.37356	1.7644
H	5.2816	2.37339	0.72902	H	4.79247	2.0981	0.17448
H	6.77006	1.43245	0.91575	H	6.43117	1.58193	0.62001
H	6.85367	-0.98765	0.35171	H	6.97054	-0.92259	0.79098
H	5.63587	-1.31035	1.60328	H	5.62136	-1.14446	1.91676
H	5.36937	-1.87358	-0.05638	H	5.70794	-2.11645	0.43094

atom	Con f. 2- 2m			atom	Con f. 2- 2n		
C	5.95545	-1.2602	-0.72084	C	-6.00416	-0.96788	0.41107

C	3.78901	2.64615	-0.43258	C	-3.31771	2.57162	0.86229
C	3.91364	1.66218	0.46638	C	-3.59622	1.83727	-0.22165
C	2.9998	0.44415	0.49428	C	-2.86165	0.54641	-0.55891
C	3.46987	-0.71983	-0.45771	C	-3.47277	-0.7289	0.13646
C	4.832	-1.19783	-0.00429	C	-4.90017	-0.90099	-0.33471
C	1.52455	0.81187	0.25408	C	-1.34696	0.65337	-0.30989
C	0.58757	-0.38318	0.45281	C	-0.58638	-0.5963	-0.78828
C	1.0178	-1.5486	-0.46216	C	-1.17318	-1.86899	-0.14531
C	2.49021	-1.90692	-0.25049	C	-2.6815	-1.9613	-0.38348
C	3.48255	-0.29817	-1.93123	C	-3.38726	-0.65493	1.66537
C	4.96774	1.72526	1.54138	C	-4.65251	2.27634	-1.20253
C	-0.88018	-0.04824	0.27302	C	0.91487	-0.47644	-0.59929
C	-1.34773	1.13732	-0.13866	C	1.77341	-0.84332	-1.55952
C	-2.80763	1.42765	-0.35804	C	3.26606	-0.68636	-1.45557
C	-3.59904	0.12343	-0.40139	C	3.59498	0.30028	-0.34158
O	-3.19652	-0.70519	0.69635	O	2.84759	-0.05949	0.83352
C	-1.85743	-1.17464	0.57298	C	1.44028	0.1155	0.69765
O	-1.55336	-1.85662	1.7641	O	0.8602	-0.44289	1.85219
C	-5.13237	0.26118	-0.32684	C	5.06523	0.39502	0.10353
O	-5.4549	1.14794	-1.41599	O	5.1229	1.39371	1.14353
C	-5.78893	-1.1013	-0.56172	C	5.60032	-0.93145	0.65248
C	-5.60201	0.86643	0.998	C	5.93824	0.91647	-1.03264
H	3.05977	0.02622	1.50883	H	-2.99458	0.37676	-1.63665
H	0.71535	-0.73541	1.48714	H	-0.7592	-0.68606	-1.86968
H	-3.36067	-0.39389	-1.3437	H	3.28529	1.30852	-0.65462
H	6.87567	-1.63976	-0.28401	H	-6.97954	-1.10967	-0.04747
H	6.00968	-0.94001	-1.75699	H	-5.98796	-0.88422	1.49363
H	4.45624	3.50479	-0.42159	H	-3.85965	3.49069	1.07237
H	3.02591	2.63798	-1.20446	H	-2.54703	2.29357	1.57426
H	4.85917	-1.539	1.03199	H	-5.001	-0.99307	-1.41751
H	1.39571	1.21237	-0.75648	H	-1.15502	0.81743	0.75313
H	1.25046	1.6175	0.94388	H	-0.96692	1.53668	-0.83573
H	0.40847	-2.4349	-0.25807	H	-0.67836	-2.74896	-0.57248
H	0.83141	-1.27189	-1.50701	H	-0.96108	-1.87814	0.92735
H	2.61348	-2.28409	0.77352	H	-2.86616	-2.0628	-1.46143
H	2.77896	-2.72421	-0.92199	H	-3.08365	-2.86462	0.09048
H	2.50541	0.06393	-2.25696	H	-2.35979	-0.51843	2.00881
H	4.20754	0.49828	-2.11316	H	-3.98063	0.17302	2.06006
H	3.74628	-1.15021	-2.56613	H	-3.76182	-1.5831	2.10931
H	5.63375	0.85678	1.49593	H	-4.21457	2.42829	-2.19789
H	5.57764	2.62968	1.45798	H	-5.43024	1.51356	-1.31625
H	4.50422	1.71296	2.53663	H	-5.13037	3.21009	-0.89154
H	-0.65806	1.95272	-0.33855	H	1.38177	-1.26088	-2.48607
H	-2.94486	1.97499	-1.29658	H	3.66796	-0.3239	-2.40816
H	-3.17819	2.08473	0.43953	H	3.73825	-1.65907	-1.26642
H	-1.82308	-1.94134	-0.21451	H	1.20444	1.18801	0.75052
H	-1.464	-1.1932	2.46718	H	0.99115	-1.40344	1.80898
H	-6.4179	1.25469	-1.42006	H	4.40665	1.16843	1.75938
H	-5.48151	-1.5094	-1.52994	H	6.60615	-0.776	1.0538
H	-6.87976	-1.00113	-0.55922	H	5.6585	-1.6984	-0.12522
H	-5.50946	-1.81263	0.21991	H	4.96087	-1.30584	1.45705
H	-5.27956	0.2555	1.84537	H	6.95142	1.10021	-0.66179
H	-5.21277	1.87928	1.1312	H	6.00069	0.19154	-1.84826
H	-6.69612	0.91927	1.0127	H	5.5413	1.85668	-1.42878

atom	Con f. 2- 2o		
C	-6.06418	-1.16639	-0.23879
C	-3.52151	1.79886	1.97403
C	-3.69097	1.61714	0.65875
C	-2.89891	0.5937	-0.14429
C	-3.52824	-0.85042	-0.12631
C	-4.89829	-0.78904	-0.76575
C	-1.40894	0.57104	0.24274
C	-0.5777	-0.35547	-0.66664
C	-1.17131	-1.77128	-0.65787
C	-2.65219	-1.7505	-1.04256
C	-3.591	-1.43979	1.28735
C	-4.67346	2.45592	-0.11757
C	0.89199	-0.3129	-0.2937
C	1.43664	-1.02584	0.6985
C	2.90557	-1.01844	1.02196
C	3.70633	-0.41595	-0.12963
O	3.05608	0.7931	-0.55706
C	1.77088	0.58483	-1.13534

O	1.16658	1.84823	-1.25322
C	5.15033	-0.00983	0.22629
O	5.10807	1.01376	1.24023
C	5.92045	-1.18058	0.82942
C	5.8725	0.5294	-1.0131
H	-2.94366	0.9127	-1.19517
H	-0.67007	0.03071	-1.69138
H	3.73508	-1.12636	-0.96876
H	-6.98618	-1.0872	-0.80917
H	-6.1529	-1.56285	0.76834
H	-4.0999	2.54223	2.51741
H	-2.80751	1.22411	2.55533
H	-4.89295	-0.39987	-1.78545
H	-1.28176	0.2445	1.28012
H	-1.01426	1.58985	0.18786
H	-0.61749	-2.40871	-1.35714
H	-1.05232	-2.2208	0.33375
H	-2.74457	-1.38864	-2.07545
H	-3.06099	-2.76781	-1.02978
H	-4.25705	-0.86222	1.93211
H	-3.96006	-2.46996	1.25223
H	-2.60779	-1.45801	1.76165
H	-4.16129	3.0114	-0.91419
H	-5.42813	1.83088	-0.60732
H	-5.18902	3.17536	0.52559
H	0.80633	-1.67194	1.30543
H	3.24184	-2.04284	1.21682
H	3.09651	-0.44537	1.93887
H	1.90814	0.14044	-2.13677
H	1.60116	2.31665	-1.9817
H	4.48913	1.68773	0.91669
H	5.93916	-2.02818	0.13796
H	5.47357	-1.50822	1.77138
H	6.9533	-0.87937	1.02971
H	6.87916	0.85937	-0.73973
H	5.95927	-0.24344	-1.78412
H	5.33145	1.3782	-1.43959

atom	Con f. 2- 3a			atom	Con f. 2- 3b		
C	-6.09346	-1.00695	0.0336	C	-5.63262	-1.55634	-1.00755
C	-3.33521	2.06922	1.80362	C	-4.46192	2.52199	-0.52354
C	-3.56457	1.74179	0.52631	C	-3.54974	1.91376	0.24453
C	-2.85114	0.59398	-0.17628	C	-2.89274	0.61874	-0.21901
C	-3.5436	-0.80435	0.0312	C	-3.59523	-0.70071	0.28473
C	-4.93169	-0.75145	-0.56998	C	-5.03843	-0.69735	-0.17759
C	-1.34873	0.54778	0.15755	C	-1.38174	0.61816	0.08262
C	-0.59735	-0.5196	-0.66576	C	-0.65967	-0.61402	-0.49905
C	-1.24884	-1.89014	-0.46239	C	-1.32822	-1.89076	0.01605
C	-2.74119	-1.8458	-0.7972	C	-2.82157	-1.89348	-0.31487
C	-3.5842	-1.22126	1.50559	C	-3.6242	-0.80691	1.82479
C	-4.53656	2.53021	-0.31312	C	-3.12068	2.52606	1.55604
C	0.8902	-0.48828	-0.3747	C	0.83294	-0.5426	-0.24616
C	1.58439	-1.50379	0.15347	C	1.52055	-1.41186	0.50447
C	3.04955	-1.4347	0.48384	C	2.99229	-1.29244	0.78813
C	3.54573	0.00769	0.45399	C	3.51038	0.08942	0.4018
O	3.02227	0.65048	-0.72075	O	2.9758	0.43753	-0.88697
C	1.60699	0.81147	-0.70277	C	1.56375	0.62481	-0.8898
O	1.24122	1.33602	-1.95438	O	1.18362	0.83811	-2.22599
C	5.07744	0.16684	0.36957	C	5.04236	0.19319	0.26176
O	5.5361	-0.41696	-0.86607	O	5.47367	-0.6901	-0.79263
C	5.77454	-0.59952	1.48935	C	5.74607	-0.28029	1.52982
C	5.46183	1.64943	0.40835	C	5.44975	1.63114	-0.07704
H	-2.92231	0.79086	-1.25515	H	-2.99977	0.58725	-1.31062
H	-0.72662	-0.2467	-1.72329	H	-0.80668	-0.58168	-1.58842
H	3.18345	0.54041	1.34547	H	3.17214	0.82966	1.14167
H	-7.03099	-0.95059	-0.51371	H	-6.68691	-1.45232	-1.2523
H	-6.1632	-1.27733	1.08301	H	-5.11264	-2.38764	-1.47397
H	-3.85877	2.89832	2.27364	H	-4.94375	3.44678	-0.21448
H	-2.62487	1.53295	2.42492	H	-4.75968	2.11375	-1.48609
H	-4.94519	-0.48561	-1.62845	H	-5.63878	0.10666	0.24501
H	-1.19097	0.3479	1.22277	H	-1.20275	0.65065	1.16289
H	-0.92934	1.53873	-0.04041	H	-0.95249	1.53542	-0.33382
H	-0.75751	-2.63703	-1.09668	H	-0.85415	-2.77121	-0.43285
H	-1.10665	-2.21874	0.5736	H	-1.18331	-1.97532	1.09954

H	-2.85682	-1.60618	-1.86259	H	-2.93489	-1.87505	-1.40606
H	-3.1908	-2.83454	-0.64815	H	-3.27847	-2.82704	0.03326
H	-2.58614	-1.24346	1.9478	H	-2.6379	-0.66103	2.27235
H	-4.19255	-0.53433	2.09795	H	-4.30405	-0.06943	2.26023
H	-4.01017	-2.22506	1.60354	H	-3.98334	-1.79872	2.12063
H	-5.00926	3.33327	0.26013	H	-3.17255	1.81489	2.38545
H	-4.02501	2.97777	-1.1755	H	-2.08039	2.86961	1.50701
H	-5.32492	1.88612	-0.71732	H	-3.74447	3.38911	1.80562
H	1.08616	-2.44328	0.37569	H	1.01206	-2.25887	0.9562
H	3.62902	-2.03702	-0.22856	H	3.17539	-1.47886	1.85239
H	3.22222	-1.87078	1.47419	H	3.54955	-2.06072	0.23573
H	1.35185	1.5957	0.02427	H	1.33317	1.5666	-0.37123
H	1.35095	0.6297	-2.61148	H	1.27689	-0.00632	-2.69598
H	4.99108	-0.02657	-1.56769	H	4.92789	-0.47639	-1.56593
H	5.59118	-1.6739	1.40957	H	6.82746	-0.15245	1.42097
H	6.85469	-0.4333	1.43331	H	5.41766	0.30295	2.39518
H	5.42552	-0.25701	2.46808	H	5.547	-1.33697	1.72491
H	6.54524	1.75368	0.29746	H	4.95313	1.97096	-0.98965
H	4.97391	2.19904	-0.40102	H	5.18003	2.31571	0.73396
H	5.1653	2.10543	1.35872	H	6.53211	1.68276	-0.22772

atom	Con f. 2- 3c			atom	Con f. 2- 3d		
C	6.05815	-1.0132	-0.51281	C	-6.09147	-0.96829	0.09941
C	3.61963	2.74856	-0.27333	C	-3.28305	2.13383	1.74424
C	3.75547	1.74842	0.60597	C	-3.52693	1.77168	0.47906
C	2.92487	0.47304	0.54668	C	-2.83426	0.59397	-0.19414
C	3.53177	-0.63278	-0.39845	C	-3.54017	-0.78868	0.06605
C	4.89074	-1.03418	0.13233	C	-4.93377	-0.73943	-0.52245
C	1.44592	0.74767	0.22039	C	-1.32888	0.54076	0.1249
C	0.58377	-0.5195	0.32552	C	-0.59893	-0.56251	-0.67047
C	1.15003	-1.62189	-0.58855	C	-1.26347	-1.91751	-0.41269
C	2.62547	-1.89066	-0.28658	C	-2.7582	-1.86599	-0.73611
C	3.6106	-0.17014	-1.85781	C	-3.56972	-1.15736	1.55361
C	4.7377	1.84723	1.74453	C	-4.49729	2.54583	-0.37531
C	-0.88537	-0.25891	0.05185	C	0.8917	-0.53479	-0.39565
C	-1.3441	0.46757	-0.97347	C	1.57165	-1.51778	0.20849
C	-2.80223	0.76783	-1.18401	C	3.04006	-1.44122	0.53089
C	-3.58574	0.50632	0.09856	C	3.52067	-0.00042	0.41472
O	-3.20919	-0.78165	0.6176	O	3.03672	0.55094	-0.82279
C	-1.85226	-0.89172	1.02876	C	1.62232	0.7247	-0.83841
O	-1.60109	-2.2654	1.21727	O	1.27554	1.12533	-2.1402
C	-5.11729	0.44717	-0.07254	C	5.04116	0.23707	0.42451
O	-5.44392	-0.65855	-0.9378	O	5.24129	1.66159	0.31426
C	-5.64728	1.70109	-0.76111	C	5.75445	-0.46039	-0.73826
C	-5.79613	0.24978	1.28722	C	5.64	-0.17183	1.76609
H	2.94837	0.03358	1.55392	H	-2.91492	0.75706	-1.27793
H	0.66607	-0.88644	1.35811	H	-0.73574	-0.32441	-1.73493
H	-3.34158	1.27991	0.84078	H	3.09656	0.5862	1.24334
H	6.97123	-1.34581	-0.02564	H	-7.03393	-0.91919	-0.44007
H	6.1567	-0.66776	-1.53759	H	-6.15295	-1.20846	1.15664
H	4.22625	3.648	-0.19885	H	-3.79224	2.98305	2.19367
H	2.90681	2.71413	-1.0911	H	-2.57441	1.60691	2.37538
H	4.87431	-1.3956	1.16192	H	-4.9558	-0.50351	-1.58782
H	1.35456	1.17056	-0.78399	H	-1.16155	0.37335	1.19417
H	1.06553	1.50766	0.91187	H	-0.89971	1.51974	-0.10908
H	0.57501	-2.54459	-0.45333	H	-0.78555	-2.69171	-1.02417
H	1.0179	-1.31978	-1.63414	H	-1.11723	-2.21147	0.63295
H	2.7076	-2.28967	0.7333	H	-2.88036	-1.65839	-1.80748
H	3.01478	-2.66532	-0.95791	H	-3.21849	-2.84381	-0.55191
H	2.63411	0.13639	-2.23856	H	-2.56784	-1.17312	1.98757
H	4.29098	0.67667	-1.97264	H	-4.16726	-0.4473	2.12945
H	3.97119	-0.98489	-2.49408	H	-4.00202	-2.15427	1.68789
H	4.21791	1.77579	2.70902	H	-3.99015	2.95587	-1.25863
H	5.45965	1.02405	1.71664	H	-5.30005	1.90021	-0.74767
H	5.29158	2.79043	1.72159	H	-4.95127	3.37477	0.17578
H	-0.6452	0.89682	-1.68847	H	1.0592	-2.42875	0.50443
H	-3.21462	0.15517	-1.99662	H	3.60632	-2.09661	-0.14357
H	-2.91964	1.81374	-1.48788	H	3.21628	-1.81344	1.54642
H	-1.73378	-0.442	2.02685	H	1.3629	1.57748	-0.19533
H	-1.6271	-2.68471	0.34181	H	1.40089	0.35972	-2.72428
H	-4.98323	-1.43054	-0.57201	H	4.67745	1.94671	-0.4232
H	-5.37207	2.59777	-0.19786	H	5.71701	-1.54937	-0.64344
H	-5.25545	1.79279	-1.7771	H	5.30314	-0.18272	-1.69514
H	-6.73907	1.65573	-0.82034	H	6.80521	-0.15589	-0.74996

H	-5.60462	1.1018	1.94791	H	5.57863	-1.25321	1.91341
H	-6.87737	0.15444	1.15016	H	6.69493	0.11758	1.80112
H	-5.42558	-0.65368	1.77878	H	5.11937	0.3243	2.59141
atom	Con f. 2- 3e			atom	Con f. 2- 3f		
C	-5.49564	-2.02953	-0.2787	C	-5.64415	-1.56061	-0.93946
C	-4.65595	1.92096	-1.70552	C	-4.4376	2.51157	-0.59445
C	-3.7469	1.80555	-0.72962	C	-3.52377	1.92308	0.1869
C	-2.96873	0.50718	-0.55137	C	-2.8805	0.60775	-0.23691
C	-3.58904	-0.50449	0.48992	C	-3.59001	-0.68829	0.31462
C	-5.00556	-0.83762	0.06624	C	-5.03731	-0.68649	-0.13481
C	-1.48028	0.7813	-0.26371	C	-1.36763	0.60452	0.05436
C	-0.6403	-0.50311	-0.24267	C	-0.65985	-0.65037	-0.49526
C	-1.22553	-1.4864	0.78749	C	-1.33514	-1.906	0.06174
C	-2.70137	-1.76691	0.4992	C	-2.83179	-1.90566	-0.25479
C	-3.68027	0.08789	1.91277	C	-3.60519	-0.74638	1.85736
C	-3.43892	2.97621	0.17224	C	-3.07938	2.57651	1.47304
C	0.83585	-0.24207	-0.01029	C	0.83578	-0.58454	-0.2565
C	1.31913	0.61175	0.89945	C	1.52552	-1.45686	0.48958
C	2.78546	0.90765	1.05619	C	3.00291	-1.34538	0.75583
C	3.54959	0.44969	-0.18249	C	3.49918	0.04354	0.3737
O	3.14262	-0.89157	-0.50585	O	2.97431	0.38623	-0.92117
C	1.78189	-1.02433	-0.89648	C	1.56231	0.58015	-0.91284
O	1.49951	-2.4046	-0.90378	O	1.16955	0.78913	-2.24546
C	5.08171	0.387	-0.01999	C	5.02224	0.24608	0.28588
O	5.40072	-0.59268	0.98788	O	5.24116	1.63078	-0.05613
C	5.64022	1.7147	0.4823	C	5.67239	-0.64185	-0.78009
C	5.74022	-0.00898	-1.34611	C	5.67144	0.03977	1.65002
H	-3.02055	-0.0215	-1.5117	H	-2.99514	0.54067	-1.32616
H	-0.73806	-0.97758	-1.22921	H	-0.81476	-0.64835	-1.58419
H	3.31042	1.11326	-1.02605	H	3.11804	0.77254	1.10463
H	-6.54192	-2.14126	-0.55262	H	-6.69973	-1.45512	-1.17791
H	-4.89457	-2.9335	-0.30451	H	-5.1336	-2.4061	-1.39055
H	-5.22079	2.83842	-1.85401	H	-4.90986	3.45024	-0.31373
H	-4.86774	1.10057	-2.38672	H	-4.74644	2.07218	-1.5396
H	-5.68266	0.015	0.06554	H	-5.62886	0.13126	0.27362
H	-1.37455	1.30017	0.69368	H	-1.18057	0.6699	1.13184
H	-1.08874	1.46081	-1.02972	H	-0.93546	1.505	-0.39398
H	-0.66362	-2.42612	0.76424	H	-0.87371	-2.80328	-0.3665
H	-1.09754	-1.0675	1.79296	H	-1.17982	-1.96095	1.14573
H	-2.77233	-2.25427	-0.48136	H	-2.95534	-1.91993	-1.34491
H	-3.09729	-2.47861	1.23313	H	-3.29292	-2.82429	0.1261
H	-2.72643	0.49512	2.25735	H	-2.61266	-0.59973	2.29055
H	-4.42598	0.88592	1.96423	H	-4.27121	0.01291	2.27652
H	-3.98605	-0.69209	2.61859	H	-3.97385	-1.72391	2.18709
H	-3.49005	2.71131	1.23205	H	-3.13158	1.89468	2.32669
H	-2.42361	3.35109	-0.00428	H	-2.03634	2.90823	1.40477
H	-4.13479	3.80046	-0.00822	H	-3.69355	3.45332	1.69707
H	0.63615	1.15428	1.54957	H	1.0167	-2.2998	0.94802
H	2.92664	1.98329	1.20829	H	3.54183	-2.12128	0.19654
H	3.19425	0.40749	1.94414	H	3.20208	-1.53422	1.81692
H	1.66932	-0.70628	-1.94475	H	1.33904	1.52356	-0.39445
H	1.5252	-2.70748	0.01827	H	1.25819	-0.05633	-2.71444
H	4.91762	-1.39787	0.74259	H	4.65615	1.80839	-0.81046
H	6.73194	1.66011	0.53369	H	6.72993	-0.37756	-0.87335
H	5.36931	2.52998	-0.19502	H	5.60781	-1.70241	-0.52032
H	5.26389	1.95025	1.48089	H	5.19244	-0.49688	-1.75217
H	5.34373	-0.96059	-1.71023	H	5.19071	0.66558	2.40876
H	5.56216	0.75137	-2.11381	H	5.60586	-1.00484	1.96449
H	6.82038	-0.112	-1.20598	H	6.72965	0.31418	1.59796
atom	Con f. 2- 3g			atom	Con f. 2- 3h		
C	-6.09426	-1.0189	0.01853	C	-5.93688	-1.34464	0.04687
C	-3.34137	2.0492	1.81046	C	-3.55686	2.109	1.56943
C	-3.57	1.72939	0.53105	C	-3.77315	1.71071	0.31
C	-2.85416	0.58761	-0.17887	C	-2.93569	0.63992	-0.37656
C	-3.54458	-0.81325	0.01829	C	-3.43681	-0.82898	-0.10102
C	-4.93254	-0.75825	-0.58299	C	-4.84787	-0.97089	-0.6268
C	-1.35215	0.54116	0.15639	C	-1.43398	0.78526	-0.0741
C	-0.59766	-0.5189	-0.67353	C	-0.5749	-0.20106	-0.89139
C	-1.24771	-1.8919	-0.4824	C	-1.05182	-1.64665	-0.66701
C	-2.74004	-1.84721	-0.8173	C	-2.54964	-1.78297	-0.94827
C	-3.58507	-1.24071	1.48965	C	-3.3449	-1.201	1.38355
C	-4.54326	2.52156	-0.30336	C	-4.86295	2.33399	-0.52334
C	0.88808	-0.48861	-0.37857	C	0.91176	-0.01647	-0.65033
C	1.59017	-1.51574	0.11513	C	1.67996	0.67386	-1.50241
C	3.05651	-1.45269	0.44616	C	3.167	0.83191	-1.35414

C	3.55789	-0.0107	0.45131	C	3.71154	-0.21036	-0.38246
O	3.01502	0.66531	-0.6951	O	2.87668	-0.22708	0.78778
C	1.59961	0.82742	-0.62948	C	1.54212	-0.67111	0.56521
O	1.18746	1.35044	-1.86504	O	0.84521	-0.43336	1.76558
C	5.08841	0.14596	0.34721	C	5.13949	0.05994	0.13405
O	5.52712	-0.40611	-0.90993	O	5.13649	1.2938	0.87882
C	5.79972	-0.65133	1.43607	C	6.11762	0.25862	-1.01955
C	5.47784	1.62608	0.41911	C	5.59984	-1.08477	1.04289
H	-2.92448	0.79176	-1.25645	H	-3.05806	0.78689	-1.45896
H	-0.72129	-0.23779	-1.72938	H	-0.75042	0.0357	-1.95002
H	3.21234	0.49668	1.36416	H	3.68824	-1.2023	-0.85645
H	-7.03166	-0.96013	-0.52874	H	-6.90021	-1.42533	-0.45031
H	-6.16406	-1.29621	1.06613	H	-5.92003	-1.58204	1.10643
H	-3.86676	2.87414	2.28573	H	-4.17295	2.87842	2.02877
H	-2.63008	1.51042	2.42849	H	-2.76661	1.69196	2.18561
H	-4.94599	-0.48555	-1.63972	H	-4.94827	-0.74944	-1.69079
H	-1.19616	0.33434	1.22063	H	-1.24777	0.63742	0.9912
H	-0.93365	1.53381	-0.0348	H	-1.12332	1.80942	-0.3085
H	-0.7559	-2.63279	-1.12323	H	-0.49391	-2.32543	-1.32325
H	-1.10462	-2.22925	0.55076	H	-0.83757	-1.95789	0.36026
H	-2.85563	-1.59999	-1.88093	H	-2.73232	-1.57457	-2.01083
H	-3.18822	-2.83773	-0.67575	H	-2.8734	-2.81537	-0.77069
H	-2.58705	-1.26455	1.93183	H	-2.33123	-1.07555	1.76907
H	-4.19468	-0.55897	2.08675	H	-4.00892	-0.58191	1.99095
H	-4.00955	-2.24581	1.58023	H	-3.62719	-2.24866	1.5305
H	-4.03216	2.97644	-1.16221	H	-5.57187	1.57746	-0.8764
H	-5.32983	1.87863	-0.71276	H	-5.41986	3.0913	0.0365
H	-5.01814	3.31947	0.27527	H	-4.43985	2.80903	-1.41831
H	1.09855	-2.46587	0.30347	H	1.21741	1.13441	-2.37451
H	3.63324	-2.03851	-0.28205	H	3.41698	1.83752	-0.99067
H	3.23054	-1.91384	1.425	H	3.64697	0.72248	-2.33269
H	1.38086	1.54428	0.17928	H	1.54407	-1.76453	0.45045
H	1.5018	2.26592	-1.9149	H	0.77736	0.52828	1.87881
H	4.96971	-0.00024	-1.59277	H	4.41536	1.2171	1.52381
H	5.46732	-0.33252	2.42851	H	6.11987	-0.61357	-1.68008
H	5.61038	-1.72257	1.33188	H	5.86269	1.14258	-1.60947
H	6.8796	-0.48801	1.36737	H	7.12983	0.3928	-0.62589
H	5.19566	2.0583	1.38486	H	5.66264	-2.02548	0.48593
H	6.55993	1.73032	0.29603	H	6.5892	-0.85792	1.45108
H	4.98062	2.19753	-0.36922	H	4.9029	-1.22552	1.87337

atom	Con f. 2- 3i			atom	Con f. 2- 3j		
C	-6.06719	-0.99736	0.45128	C	-5.64171	-1.54807	-1.02638
C	-3.59275	2.74794	0.35554	C	-4.4612	2.51987	-0.47447
C	-3.72875	1.77533	-0.55413	C	-3.55087	1.89398	0.28155
C	-2.90938	0.49181	-0.52474	C	-2.89432	0.60923	-0.20986
C	-3.53573	-0.63828	0.37811	C	-3.59269	-0.72215	0.26665
C	-4.89224	-1.01021	-0.18015	C	-5.0398	-0.70802	-0.18276
C	-1.43206	0.74334	-0.17419	C	-1.38291	0.60335	0.08816
C	-0.5809	-0.5279	-0.31379	C	-0.66032	-0.6102	-0.53063
C	-1.16414	-1.65467	0.55848	C	-1.32648	-1.90329	-0.05433
C	-2.63921	-1.9003	0.23558	C	-2.82303	-1.89733	-0.37115
C	-3.62707	-0.22177	1.85052	C	-3.60804	-0.86791	1.80362
C	-4.69793	1.91687	-1.69932	C	-3.1223	2.47728	1.6063
C	0.88875	-0.2908	-0.02253	C	0.83258	-0.54865	-0.27791
C	1.34666	0.38378	1.03858	C	1.53799	-1.48832	0.36326
C	2.80782	0.66558	1.26665	C	3.01294	-1.38845	0.64412
C	3.57056	0.47002	-0.03812	C	3.52128	0.03137	0.40784
O	3.20845	-0.80338	-0.60361	O	2.96054	0.51697	-0.82314
C	1.85311	-0.88109	-1.03029	C	1.54814	0.69892	-0.7606
O	1.59003	-2.23959	-1.29204	O	1.11435	1.01026	-2.05922
C	5.10777	0.47918	0.03676	C	5.0495	0.15977	0.25234
O	5.59297	0.29388	-1.30953	O	5.46126	-0.57992	-0.91397
C	5.66149	-0.6379	0.92696	C	5.78032	-0.46453	1.43688
C	5.6172	1.8455	0.48304	C	5.44367	1.63179	0.08876
H	-2.92451	0.0839	-1.54531	H	-3.00388	0.60087	-1.30165
H	-0.65917	-0.85974	-1.35837	H	-0.80627	-0.54356	-1.61868
H	3.27603	1.25996	-0.74469	H	3.1948	0.68187	1.23272
H	-6.97728	-1.30554	-0.05711	H	-6.69817	-1.43858	-1.25911
H	-6.17542	-0.68271	1.485	H	-5.12614	-2.3686	-1.5161
H	-4.1904	3.65484	0.30179	H	-4.94226	3.43849	-0.14642
H	-2.88874	2.6829	1.17913	H	-4.7583	2.13286	-1.446
H	-4.86689	-1.33981	-1.22017	H	-5.63603	0.08644	0.26317
H	-1.34709	1.1322	0.84449	H	-1.20233	0.60747	1.16866
H	-1.03826	1.52207	-0.83671	H	-0.95651	1.53132	-0.3061
H	-0.59549	-2.57726	0.39669	H	-0.85869	-2.76847	-0.53788

H	-1.03782	-1.38968	1.61483	H	-1.16901	-2.02463	1.02416
H	-2.71542	-2.26667	-0.79691	H	-2.94661	-1.84962	-1.46029
H	-3.04145	-2.6918	0.87906	H	-3.2755	-2.8407	-0.04432
H	-2.65145	0.05739	2.25386	H	-3.96354	-1.8674	2.07714
H	-4.2967	0.63086	1.98416	H	-2.61806	-0.73228	2.24617
H	-4.00782	-1.05132	2.45507	H	-4.28493	-0.14288	2.26387
H	-5.24515	2.86307	-1.65284	H	-3.74422	3.33648	1.87329
H	-4.16825	1.87151	-2.66001	H	-3.17701	1.74887	2.42044
H	-5.42565	1.09851	-1.70442	H	-2.08107	2.81919	1.56591
H	0.64712	0.78059	1.77126	H	1.04329	-2.38721	0.71964
H	3.20608	0.00993	2.05185	H	3.56806	-2.0872	0.00404
H	2.93438	1.69374	1.62349	H	3.20942	-1.69016	1.67922
H	1.74342	-0.3765	-2.00272	H	1.34769	1.54365	-0.08108
H	1.60732	-2.7045	-0.43986	H	1.4234	1.90558	-2.26509
H	5.10091	-0.46532	-1.66205	H	4.88395	-0.28699	-1.63714
H	5.39305	-0.49049	1.97698	H	6.85894	-0.31795	1.32426
H	5.28285	-1.61357	0.60908	H	5.46715	0.00387	2.37465
H	6.75301	-0.64965	0.85305	H	5.58835	-1.53848	1.50148
H	5.34777	2.04792	1.52271	H	4.92575	2.08007	-0.76337
H	6.70827	1.87364	0.40226	H	5.18991	2.20763	0.98495
H	5.20504	2.63983	-0.14749	H	6.52208	1.70973	-0.07831
atom	Con f. 2- 3k			atom	Con f. 2- 3l		
C	-5.50078	-2.03833	-0.58558	C	-5.49626	-2.00876	-0.35108
C	-4.78005	2.1288	-1.11514	C	-4.64195	1.97126	-1.65708
C	-3.76943	1.80357	-0.2997	C	-3.73431	1.8283	-0.68354
C	-2.98359	0.51706	-0.5244	C	-2.96144	0.52259	-0.53633
C	-3.48839	-0.72931	0.30206	C	-3.59056	-0.51333	0.47574
C	-4.95183	-0.96765	-0.0093	C	-5.00617	-0.83006	0.03668
C	-1.47208	0.74419	-0.33783	C	-1.47336	0.78359	-0.23505
C	-0.63756	-0.49791	-0.70659	C	-0.63771	-0.50386	-0.24153
C	-1.12562	-1.7226	0.08547	C	-1.23246	-1.51036	0.76061
C	-2.62614	-1.93881	-0.117	C	-2.7073	-1.77863	0.45649
C	-3.38685	-0.51226	1.8278	C	-3.68586	0.04198	1.91315
C	-3.35964	2.72903	0.81995	C	-3.42333	2.97521	0.24724
C	0.85496	-0.25562	-0.57816	C	0.8384	-0.25458	0.00519
C	1.61259	0.02916	-1.64466	C	1.31737	0.58745	0.92871
C	3.10401	0.20503	-1.59416	C	2.78543	0.87187	1.10279
C	3.65818	-0.37467	-0.29601	C	3.54077	0.42493	-0.14343
O	2.84583	0.08702	0.79671	O	3.14346	-0.91782	-0.47499
C	1.50465	-0.39226	0.78686	C	1.78597	-1.03405	-0.88483
O	0.83292	0.30285	1.81085	O	1.49227	-2.41117	-0.91451
C	5.0989	0.05498	0.04831	C	5.07749	0.40708	-0.06027
O	5.12515	1.4839	0.2347	O	5.56317	-0.01601	-1.35117
C	6.05434	-0.24423	-1.10245	C	5.59749	-0.55657	1.01116
C	5.56696	-0.63585	1.33355	C	5.61885	1.81692	0.15303
H	-3.144	0.23317	-1.57234	H	-3.01107	0.01914	-1.51021
H	-0.83035	-0.70235	-1.7688	H	-0.73147	-0.95345	-1.2401
H	3.6164	-1.47277	-0.3368	H	3.26979	1.08489	-0.98082
H	-6.57507	-2.0938	-0.74387	H	-6.54157	-2.10946	-0.63288
H	-4.92269	-2.89452	-0.9203	H	-4.89632	-2.91222	-0.40544
H	-5.35426	3.04203	-0.97712	H	-5.20317	2.89427	-1.78323
H	-5.06971	1.49017	-1.94594	H	-4.85614	1.16898	-2.35873
H	-5.60998	-0.15554	0.29537	H	-5.68244	0.02286	0.06252
H	-1.26032	1.03261	0.69382	H	-1.3712	1.27871	0.73517
H	-1.15753	1.58343	-0.96915	H	-1.07565	1.48043	-0.98205
H	-0.57901	-2.61584	-0.23958	H	-0.67385	-2.45118	0.71822
H	-0.90761	-1.59023	1.14991	H	-1.10957	-1.11584	1.7765
H	-2.80857	-2.15134	-1.17802	H	-2.7738	-2.23998	-0.53686
H	-2.95315	-2.82304	0.44231	H	-3.10985	-2.50796	1.1692
H	-2.39122	-0.18595	2.13826	H	-4.00232	-0.75389	2.5962
H	-4.10911	0.2329	2.17221	H	-2.73075	0.43176	2.27418
H	-3.60972	-1.4494	2.34965	H	-4.42519	0.84467	1.98131
H	-2.36585	3.1538	0.63437	H	-4.115	3.80676	0.08501
H	-4.06426	3.55992	0.91689	H	-3.47879	2.68497	1.3002
H	-3.3011	2.21645	1.78442	H	-2.40587	3.34964	0.08266
H	1.13648	0.11644	-2.62056	H	0.63163	1.12572	1.57914
H	3.56094	-0.29407	-2.45558	H	3.17105	0.3615	1.99484
H	3.37304	1.26723	-1.66566	H	2.93715	1.9445	1.2669
H	1.49895	-1.44391	1.10753	H	1.68614	-0.69896	-1.92894
H	0.77479	1.23334	1.54145	H	1.51765	-2.7295	0.00242
H	4.41893	1.68606	0.86879	H	5.06503	-0.82089	-1.56724
H	6.03856	-1.30958	-1.35106	H	6.68908	-0.60363	0.95509
H	5.79138	0.32718	-1.99605	H	5.32146	-0.23124	2.01837
H	7.07504	0.02525	-0.81414	H	5.19896	-1.56309	0.85481
H	5.60176	-1.72264	1.20405	H	5.22453	2.50381	-0.60268

H	6.5698	-0.2867	1.59687	H	5.35362	2.19636	1.14305
H	4.89059	-0.41232	2.16289	H	6.71029	1.80655	0.07282

atom	Con f. 2- 3m			atom	Con f. 2- 3n		
C	-6.09616	-0.96978	0.10706	C	6.05022	-1.05544	-0.53779
C	-3.29508	2.1586	1.71419	C	3.65803	2.74309	-0.21889
C	-3.53588	1.77952	0.45338	C	3.78415	1.72185	0.63728
C	-2.84028	0.59413	-0.2029	C	2.93787	0.45816	0.55164
C	-3.54518	-0.78582	0.07349	C	3.53068	-0.63354	-0.41876
C	-4.93844	-0.74537	-0.51639	C	4.88171	-1.06834	0.1056
C	-1.3354	0.5463	0.11961	C	1.46165	0.7583	0.23411
C	-0.6033	-0.56506	-0.66221	C	0.58513	-0.50052	0.31409
C	-1.26699	-1.91779	-0.39037	C	1.13424	-1.5822	-0.6354
C	-2.76153	-1.87166	-0.71534	C	2.60566	-1.88025	-0.34115
C	-3.575	-1.13665	1.56538	C	3.62052	-0.13622	-1.86608
C	-4.50586	2.54081	-0.41294	C	4.77186	1.78309	1.77383
C	0.88747	-0.53469	-0.38835	C	0.88422	-0.22387	0.05968
C	1.57041	-1.51597	0.21507	C	1.34448	0.59449	-0.89326
C	3.04004	-1.43789	0.52718	C	2.80288	0.90706	-1.09058
C	3.51857	0.00571	0.40823	C	3.6097	0.49722	0.13902
O	3.0318	0.55847	-0.82079	O	3.20696	-0.82642	0.52976
C	1.61747	0.72365	-0.83463	C	1.86044	-0.91252	0.98647
O	1.26818	1.11935	-2.13823	O	1.5434	-2.27966	1.06367
C	5.04516	0.2163	0.42754	C	5.13426	0.42001	-0.07779
O	5.46258	-0.39854	1.66196	O	5.41079	-0.60106	-1.05683
C	5.36263	1.71305	0.46623	C	5.67847	1.72378	-0.65345
C	5.74525	-0.45452	-0.75643	C	5.84037	0.07314	1.23745
H	-2.91884	0.74322	-1.2889	H	2.95762	-0.00336	1.54909
H	-0.73973	-0.33868	-1.72939	H	0.67265	-0.89554	1.33589
H	3.09751	0.57933	1.24839	H	3.40431	1.19644	0.96276
H	-7.03828	-0.92855	-0.43367	H	6.95653	-1.41411	-0.0565
H	-6.15799	-1.19849	1.1668	H	6.15625	-0.69164	-1.55544
H	-3.80642	3.01289	2.15142	H	4.27622	3.63291	-0.12636
H	-2.58676	1.64111	2.35348	H	2.9425	2.7357	-1.03495
H	-4.96018	-0.52132	-1.58432	H	4.8575	-1.45003	1.12775
H	-1.1699	0.39143	1.19104	H	1.37549	1.20296	-0.76122
H	-0.90694	1.52296	-0.12501	H	1.09232	1.50894	0.94161
H	-0.78806	-2.69813	-0.99319	H	0.5451	-2.49912	-0.5342
H	-1.12088	-2.20035	0.65846	H	1.00894	-1.24051	-1.66976
H	-2.88315	-1.67656	-1.78913	H	2.68172	-2.30828	0.66743
H	-3.221	-2.84772	-0.5201	H	2.98445	-2.64189	-1.03311
H	-2.57343	-1.14509	2.00027	H	2.65038	0.19826	-2.23961
H	-4.17448	-0.42122	2.13253	H	4.31691	0.70017	-1.95913
H	-4.00514	-2.13289	1.7113	H	3.96621	-0.94152	-2.52236
H	-5.30754	1.88931	-0.77733	H	5.48627	0.9543	1.72282
H	-4.96148	3.37685	0.12593	H	5.33472	2.72126	1.77166
H	-3.99795	2.93893	-1.30124	H	4.25551	1.69286	2.73859
H	1.0596	-2.42682	0.51477	H	0.64455	1.09244	-1.5605
H	3.6003	-2.09241	-0.15305	H	3.19429	0.38685	-1.9749
H	3.23041	-1.80899	1.53968	H	2.92439	1.97964	-1.27818
H	1.35429	1.57814	-0.19474	H	1.80361	-0.45779	1.99101
H	1.39863	0.35257	-2.71957	H	2.00508	-2.64727	1.83235
H	6.42241	-0.2836	1.72678	H	4.9223	-1.389	-0.76945
H	5.0047	2.21443	-0.43678	H	5.44312	2.56587	0.00415
H	4.88788	2.18026	1.33512	H	5.25957	1.92505	-1.64249
H	6.44458	1.86832	0.53856	H	6.76651	1.65871	-0.75091
H	5.3651	-0.06942	-1.70648	H	5.69287	0.86338	1.98104
H	6.82096	-0.25247	-0.7132	H	6.91433	-0.04013	1.06202
H	5.604	-1.53842	-0.73893	H	5.45465	-0.86211	1.65204

atom	Con f. 2- 3o		
C	-5.93018	-1.36382	0.11787
C	-3.58108	2.20551	1.41404
C	-3.78767	1.72027	0.18384
C	-2.94037	0.6091	-0.42184
C	-3.4323	-0.8391	-0.04401
C	-4.83819	-1.02672	-0.57008
C	-1.44079	0.78895	-0.1263
C	-0.57151	-0.24948	-0.86429
C	-1.03789	-1.67756	-0.53291
C	-2.53271	-1.84675	-0.81268
C	-3.35118	-1.10456	1.46385
C	-4.87375	2.28104	-0.69763
C	0.91378	-0.03824	-0.63688
C	1.67917	0.57658	-1.54806
C	3.16683	0.75404	-1.41474

C	3.70033	-0.20097	-0.35345
O	2.87331	-0.10413	0.81893
C	1.54485	-0.5845	0.63289
O	0.84107	-0.25435	1.80631
C	5.14524	0.02462	0.12623
O	5.42709	-0.9978	1.10441
C	5.34268	1.4007	0.77033
C	6.13164	-0.19917	-1.01495
H	-3.05998	0.67724	-1.51225
H	-0.74648	-0.09533	-1.93813
H	3.64189	-1.23085	-0.7358
H	-6.88864	-1.4842	-0.38072
H	-5.92056	-1.53195	1.19073
H	-4.20307	3.002	1.81542
H	-2.793	1.83573	2.06232
H	-4.93133	-0.87584	-1.64706
H	-1.25821	0.72475	0.94801
H	-1.13687	1.79469	-0.43723
H	-0.46986	-2.39979	-1.13148
H	-0.82897	-1.90709	0.51673
H	-2.70971	-1.71984	-1.88906
H	-2.85022	-2.86544	-0.56027
H	-2.34198	-0.94427	1.84794
H	-4.0252	-0.45047	2.02152
H	-3.62664	-2.14181	1.68051
H	-5.5775	1.49982	-1.0044
H	-5.43729	3.07223	-0.19411
H	-4.44549	2.69654	-1.6194
H	1.21426	0.95724	-2.45653
H	3.40297	1.79437	-1.15621
H	3.652	0.55376	-2.37653
H	1.55971	-1.68359	0.60915
H	0.76877	0.71302	1.83892
H	4.67693	-0.98212	1.72069
H	5.2278	2.21026	0.04399
H	4.62203	1.55862	1.57788
H	6.35133	1.4604	1.18986
H	6.03369	0.57541	-1.77985
H	7.15438	-0.16676	-0.62661
H	5.97211	-1.17611	-1.48255

atom	Con f. 2- 4a			atom	Con f. 2- 4b		
C	-6.11139	-1.02487	-0.09506	C	5.59228	-1.72872	0.89728
C	-3.47477	2.14998	1.6984	C	4.49743	2.41001	0.96287
C	-3.64375	1.77446	0.42487	C	3.61705	1.93133	0.07527
C	-2.88318	0.61483	-0.20458	C	2.92207	0.59847	0.32503
C	-3.56712	-0.78664	0.01801	C	3.62148	-0.65389	-0.33297
C	-4.92503	-0.77395	-0.65087	C	5.04487	-0.74697	0.1788
C	-1.39815	0.5955	0.20134	C	1.42496	0.66759	-0.03278
C	-0.60112	-0.48462	-0.5589	C	0.66341	-0.61475	0.35618
C	-1.23906	-1.8588	-0.32874	C	1.32485	-1.8233	-0.31318
C	-2.71499	-1.84819	-0.73288	C	2.80575	-1.90316	0.06393
C	-3.67543	-1.1527	1.50262	C	3.71063	-0.5445	-1.8706
C	-4.59017	2.51659	-0.48308	C	3.26019	2.72873	-1.15579
C	0.87215	-0.41286	-0.21437	C	-0.81685	-0.46513	0.07235
C	1.48793	-1.17537	0.69646	C	-1.4381	-0.89527	-1.03187
C	2.95491	-1.06939	1.00874	C	-2.91018	-0.71019	-1.27805
C	3.68876	-0.30666	-0.08602	C	-3.6368	-0.35046	0.01138
O	2.93638	0.88185	-0.40768	O	-2.88595	0.67373	0.69653
C	1.65753	0.63955	-0.96651	C	-1.6024	0.26904	1.13666
O	1.74432	0.33923	-2.3534	O	-1.67855	-0.48156	2.34191
C	5.09835	0.18973	0.29192	C	-5.05219	0.23225	-0.17183
O	4.98194	1.13592	1.37283	O	-4.95278	1.4768	-0.89163
C	5.96566	-0.95175	0.81392	C	-5.92323	-0.68607	-1.02339
C	5.76255	0.86505	-0.91287	C	-5.70225	0.47881	1.19396
H	-2.90574	0.77481	-1.29162	H	2.98493	0.41183	1.40461
H	-0.68962	-0.25671	-1.63084	H	0.76995	-0.74193	1.44296
H	3.76747	-0.93582	-0.98304	H	-3.7057	-1.23804	0.65488
H	-7.02189	-1.00101	-0.68847	H	6.63733	-1.68285	1.19386
H	-6.22881	-1.25896	0.95881	H	5.04156	-2.60794	1.2181
H	-4.03067	2.98615	2.11589	H	5.00468	3.35972	0.80947
H	-2.78347	1.6477	2.36785	H	4.74234	1.86616	1.87176
H	-4.89064	-0.54555	-1.71755	H	5.67447	0.09627	-0.10071
H	-1.28916	0.41714	1.27625	H	1.29014	0.83529	-1.10681

H	-0.97227	1.58561	0.00665	H	0.98531	1.53399	0.47425
H	-0.70308	-2.61996	-0.90762	H	0.81491	-2.74458	-0.0085
H	-1.14554	-2.14441	0.72461	H	1.22112	-1.75341	-1.40169
H	-2.78355	-1.64941	-1.8108	H	2.87813	-2.04045	1.15016
H	-3.16106	-2.83585	-0.56723	H	3.26102	-2.78799	-0.39586
H	-2.70112	-1.14236	1.99537	H	2.74521	-0.31965	-2.3303
H	-4.32368	-0.45664	2.03936	H	4.41608	0.23425	-2.17307
H	-4.09007	-2.1598	1.61449	H	4.06893	-1.4915	-2.28885
H	-4.0476	2.9461	-1.33561	H	3.90071	3.61053	-1.24678
H	-5.34718	1.8445	-0.90112	H	3.35242	2.14273	-2.07471
H	-5.10347	3.3281	0.04131	H	2.22032	3.07423	-1.11204
H	0.92333	-1.91741	1.2552	H	-0.87497	-1.40347	-1.81048
H	3.38183	-2.0728	1.11131	H	-3.33102	-1.63023	-1.69796
H	3.10144	-0.56534	1.97382	H	-3.07113	0.07843	-2.02583
H	1.15285	1.60932	-0.91987	H	-1.10318	1.20173	1.41631
H	1.9301	-0.60793	-2.44662	H	-1.86183	-1.40486	2.10899
H	4.30933	1.77851	1.09571	H	-4.28886	2.00648	-0.422
H	6.97324	-0.58172	1.02725	H	-6.93477	-0.27447	-1.09401
H	6.04477	-1.7502	0.07009	H	-5.98928	-1.68187	-0.57519
H	5.55598	-1.37233	1.73565	H	-5.52514	-0.78455	-2.03649
H	5.91515	0.14867	-1.72689	H	-5.84702	-0.46317	1.73333
H	5.14384	1.68328	-1.29051	H	-5.07764	1.13108	1.80987
H	6.73631	1.26888	-0.62026	H	-6.67854	0.95362	1.05869
atom	Con f. 2- 4c			atom	Con f. 2- 4d		
C	-6.10906	-1.04581	-0.10693	C	-6.00218	-1.21999	0.58944
C	-3.50589	2.17704	1.64897	C	-3.77597	2.66711	0.37385
C	-3.65839	1.77388	0.38183	C	-3.88839	1.68341	-0.52706
C	-2.88587	0.60431	-0.21356	C	-2.98926	0.45413	-0.52866
C	-3.56707	-0.79476	0.0304	C	-3.50384	-0.70645	0.40547
C	-4.9173	-0.80131	-0.65397	C	-4.85672	-1.16632	-0.09208
C	-1.40546	0.59939	0.20938	C	-1.51707	0.80087	-0.24213
C	-0.59669	-0.49157	-0.52293	C	-0.59166	-0.4135	-0.41074
C	-1.23067	-1.86335	-0.2694	C	-1.06709	-1.56912	0.49023
C	-2.70239	-1.86811	-0.68882	C	-2.53549	-1.90936	0.22934
C	-3.69064	-1.13047	1.52094	C	-3.5588	-0.28557	1.87852
C	-4.59691	2.49252	-0.5528	C	-4.91179	1.75838	-1.63066
C	0.87448	-0.40561	-0.17195	C	0.8703	-0.08255	-0.17861
C	1.49538	-1.17372	0.73152	C	1.32904	0.66358	0.83307
C	2.96191	-1.05663	1.04989	C	2.79162	0.91653	1.07494
C	3.67534	-0.28611	-0.0504	C	3.64833	-0.07606	0.29996
O	2.92493	0.91018	-0.34811	O	3.15087	-0.15908	-1.05227
C	1.64796	0.66568	-0.91285	C	1.83978	-0.6796	-1.17058
O	1.73441	0.38761	-2.30368	O	1.83585	-2.10143	-1.12197
C	5.11418	0.17795	0.23263	C	5.1367	0.30224	0.16908
O	5.57332	0.85819	-0.95452	O	5.24175	1.53992	-0.56194
C	5.20104	1.13526	1.42533	C	5.7634	0.55971	1.53612
C	6.03863	-1.021	0.41636	C	5.89894	-0.79957	-0.57527
H	-2.89588	0.74133	-1.30393	H	-3.02238	0.03866	-1.54563
H	-0.67898	-0.28507	-1.59977	H	-0.6902	-0.75693	-1.45069
H	3.71362	-0.90731	-0.95591	H	3.58064	-1.06645	0.77039
H	-7.01263	-1.03759	-0.71124	H	-6.91247	-1.58803	0.12297
H	-6.23805	-1.25894	0.95004	H	-6.08487	-0.90354	1.6249
H	-4.06982	3.01964	2.042	H	-4.43171	3.53407	0.34338
H	-2.82054	1.69195	2.33691	H	-3.03494	2.65059	1.16674
H	-4.87152	-0.59436	-1.72458	H	-4.85545	-1.50303	-1.13015
H	-1.30716	0.44126	1.2885	H	-1.41598	1.20052	0.77077
H	-0.98299	1.58802	0.00122	H	-1.20169	1.59931	-0.92274
H	-0.68575	-2.63492	-0.82568	H	-0.44907	-2.45506	0.30825
H	-1.14716	-2.12472	0.79111	H	-0.91432	-1.28852	1.53904
H	-2.76049	-1.69271	-1.77141	H	-2.63129	-2.28508	-0.79816
H	-3.14562	-2.8541	-0.50666	H	-2.85936	-2.72174	0.89082
H	-2.72191	-1.10615	2.02417	H	-2.58875	0.06576	2.23593
H	-4.3475	-0.42616	2.03604	H	-4.28026	0.51897	2.03765
H	-4.1027	-2.13669	1.64906	H	-3.85243	-1.13476	2.50409
H	-4.04574	2.90698	-1.40725	H	-4.42148	1.73641	-2.61279
H	-5.34541	1.80817	-0.96619	H	-5.59127	0.89975	-1.6009
H	-5.12049	3.31206	-0.05158	H	-5.51065	2.67171	-1.56629
H	0.93639	-1.92721	1.27996	H	0.63169	1.11839	1.53312
H	3.4016	-2.05539	1.14628	H	3.00427	0.83369	2.14622
H	3.09161	-0.56384	2.02261	H	3.05494	1.94167	0.78083
H	1.13537	1.63014	-0.85037	H	1.53791	-0.43213	-2.1936
H	1.91835	-0.55798	-2.41452	H	1.84555	-2.36766	-0.18961
H	4.8574	1.46668	-1.19975	H	4.72134	1.42048	-1.37221
H	6.22332	1.51607	1.50844	H	6.83036	0.77294	1.41886
H	4.94545	0.63694	2.36497	H	5.65844	-0.31723	2.18172

H	4.52455	1.98405	1.29186	H	5.29874	1.41595	2.03158
H	5.80353	-1.56525	1.3347	H	5.88286	-1.73671	-0.00896
H	5.9562	-1.71022	-0.43025	H	5.45385	-0.98671	-1.55593
H	7.07611	-0.67815	0.47989	H	6.94116	-0.49789	-0.71547
atom	Con f. 2- 4e			atom	Con f. 2- 4f		
C	5.57953	-1.76625	0.88009	C	-5.98993	-1.13042	0.25238
C	4.51579	2.38016	0.99842	C	-3.32893	2.3041	1.41882
C	3.63455	1.9196	0.10212	C	-3.62537	1.81014	0.21062
C	2.92881	0.58885	0.33266	C	-2.88547	0.63176	-0.40848
C	3.62006	-0.65991	-0.34051	C	-3.46934	-0.7706	0.01021
C	5.04145	-0.77108	0.17314	C	-4.90051	-0.86658	-0.4708
C	1.43334	0.67504	-0.02824	C	-1.3665	0.70297	-0.17566
C	0.65972	-0.60644	0.33994	C	-0.60394	-0.40704	-0.929
C	1.31451	-1.81114	-0.34282	C	-1.15859	-1.7866	-0.53961
C	2.79339	-1.90773	0.03777	C	-2.6701	-1.85472	-0.7656
C	3.7137	-0.5304	-1.87633	C	-3.36202	-1.0154	1.51999
C	3.28668	2.73548	-1.11932	C	-4.71349	2.4248	-0.63144
C	-0.81808	-0.44121	0.05007	C	0.89791	-0.27653	-0.75531
C	-1.43564	-0.86486	-1.05931	C	1.6598	0.36431	-1.65044
C	-2.90508	-0.66412	-1.31636	C	3.13344	0.5994	-1.47643
C	-3.62209	-0.31651	-0.02059	C	3.53982	0.36818	-0.02773
O	-2.87961	0.71355	0.66577	O	2.95631	-0.87441	0.41611
C	-1.60024	0.29994	1.11404	C	1.5401	-0.88839	0.47073
O	-1.68402	-0.44655	2.32047	O	1.06484	-0.31324	1.68083
C	-5.06552	0.20345	-0.13219	C	5.05449	0.22041	0.21687
O	-5.53188	0.42989	1.2146	O	5.5261	-0.94833	-0.48194
C	-5.16306	1.51095	-0.92426	C	5.82589	1.40431	-0.35795
C	-5.97728	-0.86857	-0.71987	C	5.33512	0.06971	1.716
H	2.98766	0.38753	1.40982	H	-3.04232	0.69514	-1.49459
H	0.75953	-0.74879	1.42543	H	-0.80924	-0.26123	-1.99873
H	-3.6553	-1.20786	0.62102	H	3.16261	1.18849	0.59823
H	6.62431	-1.73246	1.17924	H	-6.96898	-1.19231	-0.21593
H	5.02135	-2.64511	1.18869	H	-5.95805	-1.29315	1.32564
H	5.03051	3.32798	0.85895	H	-3.87707	3.14851	1.82997
H	4.75373	1.82297	1.90108	H	-2.53877	1.89341	2.03929
H	5.67844	0.07052	-0.09431	H	-5.01675	-0.71617	-1.54553
H	1.30313	0.85875	-1.1002	H	-1.13973	0.63587	0.89122
H	0.99971	1.53805	0.48949	H	-1.00209	1.67944	-0.5133
H	0.79624	-2.73232	-0.05225	H	-0.66408	-2.56805	-1.129
H	1.2152	-1.72615	-1.43068	H	-0.93479	-1.99696	0.51159
H	2.86147	-2.05975	1.12231	H	-2.87495	-1.73768	-1.83803
H	3.24285	-2.79008	-0.43236	H	-3.04966	-2.84272	-0.4796
H	2.75113	-0.29182	-2.33502	H	-2.33364	-0.91955	1.87362
H	4.42581	0.24695	-2.16651	H	-3.97228	-0.30602	2.08347
H	4.06561	-1.4744	-2.30662	H	-3.70231	-2.02686	1.7646
H	3.93556	3.61227	-1.19907	H	-5.196	3.26422	-0.12194
H	3.37405	2.15991	-2.04526	H	-4.3055	2.78794	-1.58392
H	2.25012	3.09038	-1.07203	H	-5.48337	1.68712	-0.88197
H	-0.8714	-1.37601	-1.83487	H	1.19778	0.75698	-2.5552
H	-3.3357	-1.5762	-1.74398	H	3.37648	1.62483	-1.7755
H	-3.04284	0.12665	-2.06595	H	3.71023	-0.06542	-2.13378
H	-1.09549	1.2299	1.39226	H	1.28981	-1.9495	0.54828
H	-1.86294	-1.37172	2.09177	H	1.04317	0.64932	1.56868
H	-4.82629	0.93112	1.65469	H	4.93058	-1.67115	-0.22703
H	-4.8986	1.36863	-1.97617	H	5.72205	1.45648	-1.44466
H	-4.49924	2.27128	-0.50312	H	6.88972	1.30161	-0.12257
H	-6.19036	1.88465	-0.87879	H	5.46896	2.3444	0.07306
H	-5.74045	-1.05651	-1.77019	H	5.0424	0.9732	2.26116
H	-5.88221	-1.80756	-0.16519	H	4.77968	-0.77456	2.13268
H	-7.01868	-0.53737	-0.65942	H	6.4038	-0.10054	1.87665
atom	Con f. 2- 4g			atom	Con f. 2- 4h		
C	-5.42612	-2.18921	-0.12882	C	-5.99248	-1.2644	0.55069
C	-4.84787	1.78183	-1.61535	C	-3.78627	2.65136	0.47098
C	-3.88571	1.72028	-0.68667	C	-3.89986	1.69734	-0.4612
C	-3.03651	0.46312	-0.54038	C	-2.99342	0.47438	-0.50991
C	-3.55584	-0.57177	0.53338	C	-3.49872	-0.72277	0.3826
C	-4.97482	-0.97345	0.18432	C	-4.84892	-1.17399	-0.13007
C	-1.55133	0.81187	-0.32427	C	-1.52288	0.81995	-0.21435
C	-0.64801	-0.42894	-0.33609	C	-0.58994	-0.38144	-0.42864
C	-1.13758	-1.43785	0.71893	C	-1.05563	-1.57118	0.43229
C	-2.60939	-1.7902	0.49955	C	-2.5223	-1.91179	0.16215
C	-3.60219	0.02219	1.95776	C	-3.55474	-0.35541	1.86973
C	-3.59207	2.91174	0.19237	C	-4.93202	1.80304	-1.55406
C	0.82086	-0.09315	-0.16086	C	0.87102	-0.04891	-0.1907
C	1.29934	0.76628	0.74624	C	1.32491	0.6777	0.8379

C	2.76724	1.02634	0.943	C	2.78686	0.94029	1.08176
C	3.60208	-0.06889	0.29265	C	3.63436	-0.0409	0.28593
O	3.09194	-0.31419	-1.03528	O	3.14805	-0.08749	-1.07268
C	1.77011	-0.81935	-1.08391	C	1.84024	-0.61745	-1.20169
O	1.74021	-2.22602	-0.87543	O	1.84564	-2.0391	-1.19044
C	5.09409	0.26888	0.10243	C	5.142	0.24834	0.18504
O	5.20637	1.39407	-0.7909	O	5.72015	-0.82328	-0.58955
C	5.73561	0.70222	1.41709	C	5.44174	1.5842	-0.50234
C	5.83805	-0.93312	-0.48965	C	5.79573	0.17678	1.56105
H	-3.10771	-0.07368	-1.49496	H	-3.02661	0.0962	-1.54137
H	-0.76233	-0.91084	-1.31743	H	-0.69015	-0.68855	-1.47977
H	3.52356	-0.99148	0.88383	H	3.53242	-1.04196	0.7273
H	-6.479	-2.35121	-0.34666	H	-6.90041	-1.62185	0.0716
H	-4.78624	-3.06488	-0.18153	H	-6.07605	-0.98942	1.5979
H	-5.46513	2.66848	-1.74041	H	-4.44711	3.51499	0.4742
H	-5.05242	0.94589	-2.27973	H	-3.03967	2.61292	1.25796
H	-5.69012	-0.1532	0.2153	H	-4.84688	-1.46963	-1.18057
H	-1.4274	1.34183	0.62484	H	-1.42289	1.18401	0.81198
H	-1.23056	1.50464	-1.11094	H	-1.21423	1.64438	-0.86644
H	-0.53002	-2.34787	0.66738	H	-0.43307	-2.44668	0.21852
H	-0.98361	-1.01157	1.71752	H	-0.9023	-1.32634	1.48991
H	-2.70366	-2.28195	-0.47687	H	-2.61749	-2.25116	-0.878
H	-2.9355	-2.51851	1.25125	H	-2.83902	-2.74954	0.79479
H	-2.652	0.47478	2.25223	H	-2.58457	-0.01904	2.24089
H	-4.38092	0.78463	2.0456	H	-4.27527	0.44393	2.05685
H	-3.83459	-0.76824	2.6798	H	-3.85029	-1.22618	2.46389
H	-4.33617	3.69914	0.04234	H	-4.44923	1.81768	-2.54003
H	-3.57777	2.65262	1.25484	H	-5.60568	0.93946	-1.54809
H	-2.60663	3.33489	-0.03637	H	-5.53588	2.70981	-1.45391
H	0.61452	1.31312	1.39049	H	0.62474	1.11172	1.54793
H	2.98886	1.07484	2.01458	H	3.00809	0.83681	2.14977
H	3.04274	2.00249	0.52095	H	3.02827	1.97735	0.81317
H	1.46294	-0.6843	-2.12626	H	1.53322	-0.34464	-2.21661
H	1.76765	-2.38912	0.08001	H	1.85235	-2.33204	-0.26616
H	4.67528	1.1723	-1.57232	H	5.1467	-0.92088	-1.36705
H	6.80335	0.88618	1.26315	H	6.52245	1.68459	-0.64021
H	5.62869	-0.07847	2.17612	H	5.09384	2.43356	0.09285
H	5.2841	1.62243	1.79617	H	4.96129	1.63341	-1.48349
H	5.81326	-1.78524	0.19765	H	5.46263	0.99884	2.19978
H	5.38556	-1.24433	-1.43481	H	5.55911	-0.77043	2.05624
H	6.88328	-0.66717	-0.67326	H	6.88275	0.24687	1.4547
atom	Con f. 2- 4i			atom	Con f. 2- 4j		
C	-5.61485	-1.8241	-0.58104	C	-6.11282	-1.04528	-0.12535
C	-4.61318	2.31153	-1.05325	C	-3.51987	2.16534	1.66455
C	-3.62502	1.90012	-0.24943	C	-3.66875	1.77391	0.39331
C	-2.93521	0.56474	-0.50009	C	-2.89276	0.6115	-0.21153
C	-3.52163	-0.65456	0.31254	C	-3.57147	-0.79117	0.01855
C	-4.99735	-0.79269	-0.00251	C	-4.92049	-0.79433	-0.66824
C	-1.40876	0.68121	-0.3279	C	-1.41295	0.6058	0.21367
C	-0.66825	-0.61096	-0.72513	C	-0.60048	-0.47713	-0.52623
C	-1.22905	-1.79608	0.07573	C	-1.23246	-1.85239	-0.28648
C	-2.74129	-1.91464	-0.11834	C	-2.70338	-1.85629	-0.70865
C	-3.41028	-0.46028	1.84052	C	-3.69705	-1.14052	1.50579
C	-3.14569	2.77449	0.88384	C	-4.6068	2.49943	-0.53647
C	0.83807	-0.45726	-0.6219	C	0.86956	-0.39216	-0.17021
C	1.58998	-0.13452	-1.68137	C	1.48681	-1.15965	0.73621
C	3.071	0.10561	-1.61123	C	2.95123	-1.03855	1.05861
C	3.50878	0.30977	-0.16723	C	3.66685	-0.27862	-0.05061
O	2.91058	-0.72342	0.64272	O	2.9284	0.91776	-0.35147
C	1.49608	-0.68374	0.72155	C	1.64836	0.67692	-0.90845
O	1.05948	0.24216	1.70817	O	1.72799	0.39903	-2.30146
C	5.02616	0.20071	0.08221	C	5.11728	0.14741	0.24458
O	5.45713	-1.13404	-0.24838	O	5.78208	-1.09343	0.55357
C	5.80564	1.14075	-0.83208	C	5.74253	0.76672	-1.00802
C	5.34309	0.49181	1.55298	C	5.22006	1.10682	1.43197
H	-3.12295	0.30839	-1.55063	H	-2.90147	0.7584	-1.30062
H	-0.8928	-0.79439	-1.78509	H	-0.68066	-0.26157	-1.60137
H	3.16468	1.29143	0.18695	H	3.6952	-0.91715	-0.94513
H	-6.69017	-1.80883	-0.74128	H	-7.01535	-1.0335	-0.73116
H	-5.09326	-2.71614	-0.91504	H	-6.24328	-1.26744	0.92958
H	-5.11905	3.26148	-0.89657	H	-4.08643	3.00298	2.06435
H	-4.95206	1.71018	-1.89312	H	-2.83499	1.67513	2.34934
H	-5.60022	0.06078	0.30352	H	-4.87322	-0.57835	-1.73701
H	-1.16035	0.93374	0.70608	H	-1.31618	0.43887	1.29164
H	-1.04343	1.507	-0.94948	H	-0.99179	1.5969	0.01451

H	-0.74305	-2.72646	-0.24125	H	-0.68491	-2.61762	-0.84891
H	-1.00183	-1.66761	1.13964	H	-1.1502	-2.12366	0.77164
H	-2.94242	-2.11004	-1.17924	H	-2.75986	-1.6713	-1.78974
H	-3.12145	-2.77862	0.43883	H	-3.14513	-2.84472	-0.53623
H	-2.39796	-0.19686	2.15709	H	-2.72932	-1.11823	2.01099
H	-4.08774	0.32316	2.19086	H	-4.35661	-0.44237	2.02577
H	-3.6908	-1.38755	2.35207	H	-4.1069	-2.14885	1.62413
H	-2.12259	3.12595	0.70428	H	-5.35296	1.81744	-0.95791
H	-3.78598	3.65435	0.9933	H	-5.13321	3.31309	-0.02865
H	-3.12672	2.24434	1.84034	H	-4.05469	2.92317	-1.38575
H	1.11452	-0.02499	-2.65508	H	0.92548	-1.91241	1.28362
H	3.32733	0.98799	-2.20768	H	3.3973	-2.03197	1.1699
H	3.61842	-0.74018	-2.04899	H	3.07452	-0.53188	2.02498
H	1.22667	-1.6647	1.12103	H	1.13933	1.64335	-0.84465
H	1.05489	1.12698	1.31212	H	1.91051	-0.54687	-2.41182
H	4.85691	-1.73405	0.22224	H	6.71216	-0.88324	0.72448
H	6.87334	1.08086	-0.59946	H	6.79258	1.01646	-0.82081
H	5.48176	2.17599	-0.68961	H	5.21902	1.68124	-1.29808
H	5.67146	0.87398	-1.88332	H	5.69842	0.0614	-1.8442
H	5.08167	1.5229	1.8131	H	4.62912	2.01016	1.2584
H	4.78367	-0.17781	2.21142	H	4.87119	0.63385	2.35379
H	6.41235	0.34968	1.73561	H	6.26393	1.40519	1.57783
atom	Con f. 2- 4k			atom	Con f. 2- 4l		
C	-6.00166	-1.08755	0.28736	C	-6.11857	-1.0478	-0.1752
C	-3.3189	2.37505	1.28623	C	-3.51847	2.07072	1.77507
C	-3.60916	1.81983	0.10346	C	-3.67295	1.74444	0.48623
C	-2.87632	0.60134	-0.44233	C	-2.89923	0.61501	-0.18092
C	-3.47468	-0.76945	0.05324	C	-3.57731	-0.79788	-0.02181
C	-4.90128	-0.88232	-0.43814	C	-4.92735	-0.76704	-0.70581
C	-1.35783	0.67542	-0.2057	C	-1.41816	0.58804	0.23939
C	-0.60288	-0.48238	-0.89107	C	-0.60605	-0.45433	-0.55719
C	-1.1647	-1.83066	-0.41267	C	-1.23792	-1.83969	-0.38639
C	-2.6752	-1.9054	-0.64421	C	-2.70998	-1.82371	-0.80412
C	-3.38801	-0.92332	1.57637	C	-3.70006	-1.22154	1.44619
C	-4.6809	2.39974	-0.78301	C	-4.61639	2.51541	-0.40064
C	0.90103	-0.35386	-0.73704	C	0.86517	-0.39167	-0.20352
C	1.66236	0.18238	-1.69992	C	1.49619	-1.23177	0.62422
C	3.14322	0.40796	-1.56511	C	2.96812	-1.14664	0.92443
C	3.53916	0.32588	-0.09924	C	3.67042	-0.2548	-0.09366
O	2.95971	-0.86677	0.46898	O	2.9041	0.9572	-0.24279
C	1.54347	-0.84678	0.54347	C	1.63833	0.74526	-0.83543
O	1.09385	-0.13909	1.69068	O	1.75443	0.47046	-2.22573
C	5.04063	0.26585	0.22855	C	5.11005	0.14678	0.2616
O	5.15017	0.21196	1.66649	O	5.03171	0.86196	1.51137
C	5.72823	-0.9628	-0.37441	C	5.98432	-1.0992	0.43086
C	5.73498	1.55415	-0.20043	C	5.69122	1.06568	-0.81737
H	-3.02622	0.603	-1.53122	H	-2.91168	0.81804	-1.26093
H	-0.81267	-0.40386	-1.96692	H	-0.68914	-0.1836	-1.61996
H	3.13234	1.19763	0.43138	H	3.70012	-0.76899	-1.06268
H	-6.97601	-1.16909	-0.18771	H	-7.02198	-1.00618	-0.77839
H	-5.98373	-1.18039	1.36919	H	-6.24707	-1.32535	0.86678
H	-3.86058	3.24692	1.64515	H	-4.08356	2.88655	2.21962
H	-2.53977	1.98944	1.93616	H	-2.83016	1.54682	2.43087
H	-5.00366	-0.80038	-1.52167	H	-4.88194	-0.49553	-1.76198
H	-1.13618	0.66798	0.86422	H	-1.31843	0.36574	1.30712
H	-0.98539	1.62867	-0.59691	H	-0.9998	1.58914	0.09178
H	-0.67058	-2.65207	-0.94522	H	-0.6915	-2.57552	-0.9877
H	-0.94722	-1.96993	0.6518	H	-1.15329	-2.16391	0.65667
H	-2.87087	-1.85735	-1.72367	H	-2.77019	-1.58517	-1.87439
H	-3.06312	-2.87069	-0.29828	H	-3.15013	-2.82016	-0.68022
H	-2.3631	-0.81639	1.93678	H	-2.73171	-1.21978	1.95056
H	-3.99758	-0.17521	2.08793	H	-4.36167	-0.55219	2.00041
H	-3.74162	-1.91478	1.87752	H	-4.10597	-2.23599	1.51485
H	-4.25703	2.7112	-1.74682	H	-5.36882	1.85613	-0.84651
H	-5.45337	1.65609	-1.00682	H	-5.13537	3.30626	0.14913
H	-5.16306	3.2675	-0.32304	H	-4.07047	2.97667	-1.23421
H	1.19583	0.48749	-2.6353	H	0.94565	-2.03039	1.11427
H	3.40579	1.39259	-1.96744	H	3.40124	-2.15249	0.8988
H	3.69254	-0.33128	-2.16334	H	3.13089	-0.76246	1.94008
H	1.27505	-1.88887	0.73416	H	1.11306	1.69705	-0.69108
H	1.07778	0.80655	1.47766	H	2.07223	1.28073	-2.65328
H	4.51866	-0.46876	1.95066	H	5.9389	1.0876	1.7654
H	6.76173	-1.00888	-0.01836	H	7.01885	-0.79921	0.63008
H	5.74597	-0.92146	-1.46733	H	5.98116	-1.71311	-0.47558
H	5.21725	-1.88181	-0.07382	H	5.64304	-1.71279	1.2685

H	5.75586	1.6483	-1.28917	H	5.7285	0.55747	-1.78656
H	5.22584	2.42854	0.21753	H	5.08658	1.96895	-0.92134
H	6.76796	1.552	0.16123	H	6.71224	1.35723	-0.54846

atom	Con f. 2- 4m		
C	-5.40326	-2.20902	-0.18211
C	-4.87675	1.80959	-1.55029
C	-3.90986	1.72879	-0.62805
C	-3.0472	0.47664	-0.52575
C	-3.54643	-0.59389	0.52214
C	-4.96399	-1.0003	0.17278
C	-1.56393	0.83444	-0.31127
C	-0.64667	-0.39482	-0.36775
C	-1.11757	-1.43842	0.66282
C	-2.58726	-1.79995	0.44427
C	-3.58597	-0.0433	1.96425
C	-3.6244	2.89514	0.28654
C	0.82137	-0.05041	-0.19768
C	1.29238	0.8452	0.67882
C	2.7595	1.12204	0.86943
C	3.58307	-0.0077	0.2708
O	3.09486	-0.28859	-1.05845
C	1.77606	-0.80627	-1.09475
O	1.75594	-2.2053	-0.84271
C	5.09759	0.22003	0.12626
O	5.64887	-0.98987	-0.43452
C	5.43361	1.39793	-0.79375
C	5.74711	0.38702	1.49603
H	-3.12156	-0.03354	-1.49452
H	-0.76503	-0.84998	-1.36166
H	3.45443	-0.90877	0.8863
H	-6.4561	-2.376	-0.39635
H	-4.75299	-3.07382	-0.27384
H	-5.50367	2.69327	-1.6446
H	-5.07553	0.99306	-2.24005
H	-5.68905	-0.1909	0.24125
H	-1.44027	1.33608	0.6531
H	-1.25723	1.55514	-1.07811
H	-0.50296	-2.34089	0.58213
H	-0.96026	-1.03683	1.67116
H	-2.68361	-2.26341	-0.54568
H	-2.8994	-2.55407	1.17617
H	-2.6396	0.41523	2.26204
H	-4.37531	0.70372	2.08408
H	-3.79785	-0.8588	2.66437
H	-2.64479	3.33611	0.06654
H	-4.37763	3.67873	0.16468
H	-3.602	2.6032	1.34026
H	0.60262	1.41438	1.29723
H	2.98282	1.21658	1.93774
H	3.01966	2.08491	0.40966
H	1.47022	-0.70606	-2.14125
H	1.78538	-2.34069	0.11703
H	5.07182	-1.21815	-1.18147
H	5.11463	2.35195	-0.36406
H	4.95141	1.28122	-1.76833
H	6.51636	1.43815	-0.94577
H	5.42868	1.31797	1.97179
H	5.48949	-0.44932	2.15379
H	6.83559	0.41507	1.38539

atom	Con f. 3- 1a			atom	Con f. 3- 1b		
C	-5.96211	-0.99504	0.85201	C	-5.99353	-1.10205	0.07835
C	-3.53534	2.75285	0.12462	C	-3.42828	2.31191	1.37394
C	-3.74935	1.68262	-0.65018	C	-3.64441	1.82748	0.1451
C	-2.91942	0.40902	-0.55151	C	-2.86239	0.65568	-0.43359
C	-3.4544	-0.61156	0.52438	C	-3.46418	-0.75155	-0.05708
C	-4.84536	-1.05381	0.12486	C	-4.87374	-0.84102	-0.59796
C	-1.41939	0.69987	-0.3618	C	-1.36217	0.72991	-0.10262
C	-0.5757	-0.57679	-0.43958	C	-0.5485	-0.37951	-0.79645
C	-1.05983	-1.58743	0.61789	C	-1.13447	-1.77261	-0.50258
C	-2.55369	-1.87657	0.45744	C	-2.63123	-1.8236	-0.81324
C	-4.82516	1.69371	-1.70526	C	-4.67691	2.44697	-0.76108
C	-3.434	-0.02615	1.94081	C	-3.41787	-1.01505	1.45286

C	0.91545	-0.34383	-0.33214	C	0.92346	-0.28145	-0.45455
C	1.8212	-1.3535	-0.87072	C	1.42094	-0.50153	0.89914
C	3.2969	-1.00391	-0.86555	C	2.93113	-0.53993	1.05155
C	3.65886	-0.12626	0.31985	C	3.62031	0.40107	0.07979
O	2.76624	1.02464	0.37614	O	3.15257	0.14119	-1.27739
C	1.45821	0.79147	0.1742	C	1.83397	-0.04926	-1.43434
O	1.42313	-2.40602	-1.38453	O	0.69266	-0.72018	1.87576
C	5.08595	0.45605	0.29092	C	5.1559	0.27672	0.02591
C	5.33113	1.32743	1.52755	C	5.73439	1.26034	-0.99668
O	5.24932	1.24136	-0.90231	O	5.50978	-1.06916	-0.33411
C	6.12203	-0.6617	0.20946	C	5.761	0.52176	1.40594
H	-3.0199	-0.10978	-1.51499	H	-2.95238	0.72346	-1.52712
H	-0.75031	-1.03567	-1.42276	H	-0.63913	-0.21398	-1.8793
H	3.50616	-0.67984	1.25504	H	3.3451	1.43932	0.30428
H	-6.90458	-1.36654	0.45779	H	-6.95254	-1.15822	-0.43042
H	-5.98959	-0.58	1.85512	H	-6.00675	-1.26815	1.15145
H	-4.14348	3.64879	0.02526	H	-4.00453	3.15127	1.75587
H	-2.75582	2.78018	0.87943	H	-2.67734	1.89915	2.04004
H	-4.90117	-1.48671	-0.87534	H	-4.94461	-0.68705	-1.67612
H	-1.25769	1.192	0.60257	H	-1.20704	0.66899	0.97613
H	-1.09762	1.40922	-1.13255	H	-0.978	1.70606	-0.41949
H	-0.49478	-2.52022	0.52384	H	-0.60838	-2.51944	-1.10899
H	-0.84688	-1.18583	1.61604	H	-0.95483	-2.03519	0.54304
H	-2.71161	-2.36628	-0.51275	H	-2.77768	-1.67727	-1.89188
H	-2.88717	-2.58587	1.22409	H	-3.03192	-2.81574	-0.57367
H	-5.37062	2.64202	-1.71751	H	-5.19884	3.27651	-0.27474
H	-4.39044	1.53314	-2.7006	H	-4.20692	2.82577	-1.67832
H	-5.5464	0.88511	-1.54496	H	-5.42275	1.70852	-1.07407
H	-2.43388	0.30527	2.22768	H	-2.40385	-0.92741	1.84748
H	-4.10442	0.83181	2.02848	H	-4.04817	-0.31089	2.00087
H	-3.75044	-0.78158	2.66727	H	-3.77081	-2.02834	1.67104
H	3.87656	-1.92849	-0.85245	H	3.18195	-0.28217	2.08166
H	3.52565	-0.4751	-1.80011	H	3.26676	-1.56908	0.86868
H	0.86558	1.65084	0.47039	H	1.55032	0.00487	-2.48277
H	5.24723	0.7359	2.44491	H	5.50567	2.2938	-0.71725
H	4.61132	2.14852	1.58324	H	5.32773	1.07841	-1.99506
H	6.33795	1.75191	1.47952	H	6.82109	1.14436	-1.03842
H	4.57374	1.93712	-0.87774	H	5.12314	-1.24087	-1.20704
H	6.04521	-1.20555	-0.73518	H	5.46566	-0.25705	2.11332
H	7.12634	-0.23336	0.27471	H	6.85226	0.51771	1.33169
H	5.99563	-1.36979	1.03353	H	5.44634	1.49208	1.80064
atom		Con f. 3- 1c		atom		Con f. 3- 1d	
C	-5.99392	-1.26608	-0.10423	C	-5.47764	-2.02983	0.0593
C	-3.44412	1.80575	1.98685	C	-4.74199	1.85557	-1.5808
C	-3.66752	1.59935	0.68341	C	-3.74346	1.77093	-0.6935
C	-2.88007	0.58933	-0.14077	C	-2.96585	0.46967	-0.5315
C	-3.46658	-0.87183	-0.07969	C	-3.50596	-0.48993	0.6002
C	-4.85849	-0.85891	-0.67372	C	-4.95436	-0.82808	0.3076
C	-1.37462	0.61448	0.1795	C	-1.45563	0.737	-0.3831
C	-0.5641	-0.30302	-0.75583	C	-0.63586	-0.55602	-0.3865
C	-1.10386	-1.7357	-0.68122	C	-1.13098	-1.4796	0.7422
C	-2.59802	-1.76449	-1.01103	C	-2.62775	-1.7586	0.5967
C	-4.71126	2.39445	-0.05805	C	-3.33923	2.97515	0.122
C	-3.46656	-1.4415	1.34345	C	-3.48144	0.16533	1.9978
C	0.92067	-0.17407	-0.49323	C	0.8605	-0.34515	-0.3102
C	1.62512	0.98954	-1.02044	C	1.74016	-1.41749	-0.7653
C	3.06248	1.16579	-0.57183	C	3.22356	-1.10401	-0.7942
C	3.74367	-0.16985	-0.32883	C	3.61017	-0.13264	0.3072
O	2.92763	-0.99347	0.55559	O	2.7467	1.04023	0.2575
C	1.60659	-1.02331	0.31119	C	1.43182	0.81913	0.0873
O	1.0767	1.83907	-1.73323	O	1.31536	-2.49981	-1.1876
C	5.12879	-0.08387	0.34168	C	5.05202	0.40607	0.2285
C	5.70935	-1.48717	0.54807	C	5.3176	1.38987	1.3731
O	4.99965	0.58577	1.60733	O	5.24334	1.0619	-1.0362
C	6.0777	0.76945	-0.49625	C	6.05652	-0.74283	0.268
H	-2.97767	0.89326	-1.19237	H	-3.10443	-0.09587	-1.4615
H	-0.72571	0.05887	-1.78013	H	-0.83075	-1.07814	-1.3337
H	3.82668	-0.72403	-1.27239	H	3.44405	-0.59552	1.2883
H	-6.93611	-1.22174	-0.64458	H	-6.54361	-2.14436	-0.1218
H	-6.03715	-1.65334	0.90942	H	-4.88574	-2.9396	0.0264
H	-4.0209	2.53796	2.54677	H	-5.3103	2.77302	-1.7154
H	-2.6856	1.26272	2.54164	H	-5.02634	1.00884	-2.2005
H	-4.89877	-0.48155	-1.69702	H	-5.62474	0.02967	0.3241
H	-1.18906	0.30955	1.21497	H	-1.27093	1.28713	0.5457
H	-1.01399	1.64385	0.08629	H	-1.12928	1.38776	-1.2031

H	-0.55731	-2.37916	-1.38072	H	-0.57879	-2.42415	0.7161
H	-0.94173	-2.15145	0.31942	H	-0.91192	-1.00941	1.7088
H	-2.73586	-1.42529	-2.04632	H	-2.78412	-2.2933	-0.3486
H	-2.97681	-2.79211	-0.96402	H	-2.963	-2.42915	1.3965
H	-5.22263	3.10375	0.59953	H	-3.29055	2.75719	1.1926
H	-4.25344	2.95685	-0.88236	H	-2.3426	3.3297	-0.1674
H	-5.46461	1.73833	-0.5075	H	-4.04213	3.79962	-0.0274
H	-2.46789	-1.43086	1.78468	H	-4.2071	0.98025	2.0677
H	-4.12423	-0.8712	2.00301	H	-3.74856	-0.5774	2.7573
H	-3.8117	-2.48044	1.33427	H	-2.49767	0.56599	2.2549
H	3.59831	1.73993	-1.32972	H	3.78092	-2.03762	-0.7006
H	3.05799	1.75408	0.35522	H	3.4604	-0.66637	-1.7728
H	1.132	-1.83478	0.85419	H	0.86096	1.71502	0.3082
H	5.85548	-1.99393	-0.41124	H	6.33771	1.77577	1.2917
H	5.048	-2.10322	1.16304	H	5.20903	0.89717	2.3446
H	6.67789	-1.40973	1.05015	H	4.62404	2.23425	1.3384
H	4.37227	0.0712	2.13889	H	4.59138	1.77846	-1.0864
H	5.74405	1.80916	-0.53821	H	5.90767	-1.35668	1.1609
H	7.07622	0.7503	-0.05015	H	5.96762	-1.38011	-0.6151
H	6.14891	0.38203	-1.51661	H	7.07235	-0.33804	0.294
atom	Con f. 3- 1e			atom	Con f. 3- 1f		
C	-5.5134	-1.71983	-1.1085	C	-5.53718	-1.92311	-0.61528
C	-4.62684	2.39624	-0.33376	C	-4.63337	2.15549	-1.34538
C	-3.66455	1.79446	0.37606	C	-3.6733	1.8449	-0.46565
C	-2.92529	0.59003	-0.19412	C	-2.925	0.52177	-0.57838
C	-3.51997	-0.8132	0.21755	C	-3.50977	-0.65445	0.29714
C	-4.9676	-0.88156	-0.22575	C	-4.96964	-0.8533	-0.05563
C	-1.41311	0.67289	0.08478	C	-1.41618	0.71081	-0.33846
C	-0.62532	-0.45334	-0.60944	C	-0.60688	-0.57737	-0.57502
C	-1.17859	-1.81349	-0.17221	C	-1.18477	-1.75068	0.23597
C	-2.67493	-1.90196	-0.47884	C	-2.6822	-1.9167	-0.02453
C	-3.25997	2.33103	1.72785	C	-3.28304	2.81885	0.61943
C	-3.51323	-1.03613	1.74539	C	-3.45221	-0.35574	1.81107
C	0.86505	-0.27918	-0.4127	C	0.87035	-0.36681	-0.31633
C	1.55393	0.75292	-1.18027	C	1.38674	-0.05047	1.01056
C	2.99894	1.01639	-0.80506	C	2.89796	-0.07235	1.15602
C	3.69339	-0.24896	-0.33335	C	3.59964	0.38792	-0.1094
O	2.90503	-0.89055	0.71239	O	3.09235	-0.35212	-1.25975
C	1.57691	-0.96178	0.51806	C	1.76806	-0.55599	-1.31669
O	0.98864	1.43778	-2.04149	O	0.67185	0.1547	1.99998
C	5.09551	-0.03815	0.27188	C	5.12701	0.17591	-0.12135
C	5.68627	-1.37648	0.72808	C	5.72215	0.66413	-1.44617
O	4.99725	0.86086	1.38933	O	5.4089	-1.22197	0.0581
C	6.01889	0.63999	-0.73693	C	5.77984	0.89535	1.05612
H	-3.0517	0.63267	-1.28328	H	-3.05229	0.17939	-1.61344
H	-0.80523	-0.35391	-1.68869	H	-0.71491	-0.83669	-1.63764
H	3.75378	-0.97128	-1.15721	H	3.37304	1.44423	-0.30122
H	-6.57637	-1.67466	-1.33267	H	-6.60691	-1.94643	-0.80867
H	-4.94391	-2.47664	-1.63942	H	-4.97946	-2.80965	-0.9019
H	-5.16601	3.25848	0.05183	H	-5.18075	3.09349	-1.2865
H	-4.90912	2.04589	-1.32343	H	-4.90691	1.47973	-2.1519
H	-5.61651	-0.15492	0.26045	H	-5.60846	-0.00918	0.19919
H	-1.21342	0.62518	1.16099	H	-1.23499	1.06445	0.6783
H	-1.04432	1.64383	-0.26194	H	-1.04569	1.49281	-1.01206
H	-0.64495	-2.62037	-0.68816	H	-0.65869	-2.6722	-0.03994
H	-1.01319	-1.96333	0.90109	H	-0.99894	-1.59055	1.30133
H	-2.81053	-1.81485	-1.56404	H	-2.82827	-2.17233	-1.0819
H	-3.05737	-2.88807	-0.19124	H	-3.07002	-2.75927	0.55984
H	-3.25218	1.55575	2.49923	H	-3.27582	2.35679	1.61072
H	-2.24605	2.74768	1.69691	H	-2.27159	3.20823	0.45252
H	-3.93731	3.12717	2.04979	H	-3.96818	3.67103	0.64379
H	-4.23672	-0.38605	2.24505	H	-4.15925	0.43085	2.0883
H	-3.79331	-2.07126	1.96938	H	-3.72542	-1.25424	2.37544
H	-2.53223	-0.85085	2.18993	H	-2.45698	-0.04565	2.13828
H	3.51368	1.44121	-1.66849	H	3.17366	0.56193	2.00006
H	3.0109	1.76536	-0.00231	H	3.20146	-1.10062	1.39258
H	1.12298	-1.65495	1.21911	H	1.46757	-0.90828	-2.30064
H	5.04915	-1.85576	1.47597	H	5.27852	0.13915	-2.29635
H	6.67253	-1.20734	1.16948	H	6.80051	0.48193	-1.45012
H	5.79689	-2.06258	-0.11772	H	5.55052	1.73715	-1.57894
H	4.39178	0.45456	2.02898	H	4.98705	-1.69372	-0.67706
H	7.0272	0.70916	-0.31874	H	5.46316	0.46511	2.00937
H	6.06995	0.06418	-1.66551	H	6.86705	0.79923	0.98423
H	5.67761	1.65167	-0.9696	H	5.52696	1.95944	1.04952
atom	Con f. 3- 1g			atom	Con f. 3- 1h		

C	-5.94281	-1.02784	0.87199	C	-5.98841	-1.28161	-0.10651
C	-3.55351	2.74081	0.18135	C	-3.46127	1.82901	1.96361
C	-3.76478	1.68343	-0.61161	C	-3.67992	1.60556	0.66218
C	-2.92463	0.41483	-0.54253	C	-2.88388	0.59095	-0.14806
C	-3.44079	-0.62839	0.52077	C	-3.4636	-0.87245	-0.07414
C	-4.83465	-1.06998	0.13061	C	-4.85278	-0.87307	-0.67461
C	-1.42494	0.71283	-0.36168	C	-1.38018	0.62668	0.17863
C	-0.57393	-0.55645	-0.4744	C	-0.56028	-0.29829	-0.74079
C	-1.04015	-1.59331	0.56557	C	-1.09415	-1.73271	-0.65516
C	-2.53445	-1.8871	0.41782	C	-2.58654	-1.77086	-0.99193
C	-4.8481	1.70545	-1.65885	C	-4.72713	2.38485	-0.09109
C	-3.40594	-0.06904	1.94745	C	-3.4672	-1.42696	1.35502
C	0.91675	-0.31572	-0.37541	C	0.92187	-0.15995	-0.46827
C	1.82228	-1.29884	-0.96389	C	1.63386	0.97385	-1.05064
C	3.29598	-0.93173	-0.96653	C	3.06671	1.17264	-0.59004
C	3.64519	-0.12098	0.26816	C	3.72443	-0.16036	-0.28272
O	2.7636	1.03477	0.36954	O	2.90803	-0.91616	0.65897
C	1.45471	0.80379	0.17156	C	1.59051	-0.96098	0.39881
O	1.42546	-2.33486	-1.50982	O	1.09418	1.78489	-1.81236
C	5.07896	0.42952	0.36169	C	5.13896	-0.11775	0.32105
C	6.07892	-0.71947	0.44877	C	6.12176	0.46887	-0.68782
O	5.18654	1.13302	1.6137	O	5.55821	-1.47991	0.52504
C	5.41624	1.37797	-0.79184	C	5.1839	0.63857	1.65119
H	-3.03044	-0.08863	-1.51353	H	-2.97845	0.88336	-1.2032
H	-0.75491	-0.9944	-1.46582	H	-0.71461	0.05185	-1.7699
H	3.45168	-0.7218	1.16652	H	3.76162	-0.76886	-1.19576
H	-6.88855	-1.39631	0.48279	H	-6.9283	-1.24824	-0.65163
H	-5.95984	-0.6299	1.88222	H	-6.03403	-1.65941	0.91061
H	-4.16918	3.63368	0.10275	H	-4.04445	2.56402	2.51317
H	-2.76863	2.76041	0.93084	H	-2.70097	1.29743	2.52705
H	-4.9009	-1.48623	-0.87599	H	-4.89047	-0.50586	-1.70168
H	-1.25728	1.18627	0.61092	H	-1.19837	0.33596	1.21884
H	-1.11493	1.43968	-1.12083	H	-1.02333	1.65662	0.07447
H	-0.47117	-2.52064	0.44339	H	-0.54139	-2.37951	-1.34658
H	-0.81653	-1.21335	1.56979	H	-0.93409	-2.13891	0.34971
H	-2.70209	-2.35852	-0.55979	H	-2.72039	-1.44247	-2.03123
H	-2.85459	-2.61312	1.17442	H	-2.96155	-2.79946	-0.93669
H	-5.56318	0.88971	-1.50732	H	-5.47516	1.71837	-0.53408
H	-5.39956	2.65033	-1.65128	H	-5.24459	3.09855	0.5569
H	-4.41941	1.5645	-2.65975	H	-4.2713	2.9403	-0.9212
H	-2.40493	0.26524	2.22764	H	-4.13012	-0.85266	2.00582
H	-4.08164	0.78196	2.05924	H	-3.80771	-2.46747	1.35545
H	-3.70737	-0.83994	2.66396	H	-2.47047	-1.40744	1.80037
H	3.89284	-1.84455	-1.01103	H	3.62141	1.70852	-1.36267
H	3.4904	-0.35527	-1.88007	H	3.03441	1.80882	0.3039
H	0.85845	1.64594	0.50703	H	1.10449	-1.7356	0.98406
H	5.81038	-1.40939	1.25496	H	5.93104	1.53106	-0.85773
H	7.07336	-0.31467	0.65831	H	6.05812	-0.05802	-1.64512
H	6.12774	-1.27666	-0.48963	H	7.13983	0.36146	-0.30214
H	4.50763	1.82527	1.60969	H	4.90477	-1.89237	1.1109
H	4.69484	2.19878	-0.84738	H	4.95213	1.69957	1.52152
H	6.41148	1.80147	-0.62942	H	4.47028	0.21593	2.36492
H	5.41922	0.85804	-1.75408	H	6.18835	0.55787	2.0763
atom	Con f. 3- 1i			atom	Con f. 3- 1j		
C	-5.99643	-1.06937	0.13973	C	-5.87712	-0.86847	-0.11537
C	-3.39477	2.37664	1.2691	C	-3.25206	2.63173	0.83151
C	-3.62029	1.83964	0.06404	C	-3.32146	1.88237	-0.27541
C	-2.85184	0.63482	-0.46322	C	-2.56799	0.56859	-0.43514
C	-3.46471	-0.74757	-0.02006	C	-3.34052	-0.67804	0.14446
C	-4.87713	-0.84828	-0.55146	C	-4.64013	-0.83802	-0.61375
C	-1.34963	0.71126	-0.14139	C	-1.13006	0.64746	0.10921
C	-0.5481	-0.43772	-0.78293	C	-0.3266	-0.61596	-0.2177
C	-1.14629	-1.81016	-0.42397	C	-1.04641	-1.85526	0.34677
C	-2.64444	-1.86157	-0.72781	C	-2.48593	-1.93896	-0.16412
C	-4.65023	2.42783	-0.8658	C	-4.14209	2.32388	-1.45975
C	-3.41582	-0.94102	1.5004	C	-3.57985	-0.56281	1.65421
C	0.92551	-0.33805	-0.44759	C	1.11918	-0.56446	0.22548
C	1.42042	-0.50328	0.91614	C	2.07034	-1.4953	-0.37669
C	2.93264	-0.55478	1.0656	C	3.42802	-1.57356	0.29197
C	3.60531	0.35742	0.05684	C	3.90415	-0.22597	0.8192
O	3.1559	0.02382	-1.28985	O	2.84772	0.39801	1.61354
C	1.8358	-0.16162	-1.44012	C	1.5742	0.26568	1.19594
O	0.6908	-0.67014	1.90124	O	1.77251	-2.25245	-1.30708
C	5.14306	0.3372	0.01129	C	4.40411	0.75654	-0.2697
C	5.71253	0.86235	1.3258	C	4.57346	2.16507	0.30343

O	5.55473	1.28268	-0.99391	O	3.49689	0.79104	-1.38875
C	5.69905	-1.04876	-0.3255	C	5.72946	0.24838	-0.83853
H	-2.94546	0.65285	-1.55841	H	-2.48621	0.37935	-1.51451
H	-0.63888	-0.32225	-1.87222	H	-0.30972	-0.7262	-1.31112
H	3.28356	1.393	0.22745	H	4.71364	-0.36635	1.53796
H	-6.95788	-1.13985	-0.36261	H	-6.73741	-1.00561	-0.76556
H	-6.00695	-1.18688	1.21925	H	-6.08646	-0.75809	0.94439
H	-3.96069	3.23858	1.61436	H	-3.79978	3.56766	0.91138
H	-2.6461	1.98618	1.95103	H	-2.65355	2.34779	1.69141
H	-4.95086	-0.74209	-1.6352	H	-4.51284	-0.95535	-1.69125
H	-1.19038	0.70244	0.93856	H	-1.15497	0.8028	1.19245
H	-0.95826	1.66735	-0.50674	H	-0.63506	1.5258	-0.32042
H	-0.62892	-2.58897	-0.99669	H	-0.50419	-2.76154	0.05788
H	-0.9666	-2.02641	0.63213	H	-1.02478	-1.80948	1.44231
H	-2.79361	-1.76538	-1.81163	H	-2.46241	-2.0857	-1.25213
H	-3.05335	-2.83748	-0.44025	H	-2.98829	-2.81678	0.25923
H	-5.40725	1.6846	-1.13825	H	-3.50543	2.44332	-2.3463
H	-5.15868	3.28655	-0.41729	H	-4.89944	1.57572	-1.71735
H	-4.18098	2.75546	-1.80284	H	-4.64934	3.2746	-1.27046
H	-2.39926	-0.84858	1.88706	H	-4.22103	0.28829	1.89377
H	-4.03482	-0.20348	2.01645	H	-4.06361	-1.47026	2.02983
H	-3.78113	-1.93815	1.76711	H	-2.64472	-0.4381	2.20417
H	3.19784	-0.26664	2.08432	H	3.32152	-2.2682	1.13543
H	3.24223	-1.59712	0.91619	H	4.15239	-2.00751	-0.39823
H	1.55114	-0.15581	-2.48946	H	0.92294	0.93059	1.75438
H	5.2876	1.84097	1.57029	H	4.98393	2.82455	-0.46638
H	6.79672	0.97125	1.22971	H	5.25622	2.16305	1.15887
H	5.50916	0.17485	2.14993	H	3.61462	2.57219	0.63553
H	5.15098	1.0043	-1.83027	H	2.69218	1.25365	-1.11197
H	5.27939	-1.42178	-1.2647	H	5.61359	-0.72819	-1.31582
H	6.78549	-0.98336	-0.43319	H	6.09098	0.95225	-1.5935
H	5.47664	-1.7749	0.46149	H	6.48407	0.1658	-0.05169
atom		Con f. 3- 1k		atom		Con f. 3- 1l	
C	-5.46649	-2.04406	0.0583	C	-5.95023	-1.02442	0.88077
C	-4.75964	1.86808	-1.53784	C	-3.55529	2.74811	0.14008
C	-3.75406	1.77464	-0.65938	C	-3.77088	1.6782	-0.63473
C	-2.96953	0.47454	-0.52343	C	-2.93163	0.40988	-0.54817
C	-3.49449	-0.50456	0.59889	C	-3.44926	-0.61732	0.52995
C	-4.94454	-0.84369	0.3159	C	-4.84043	-1.06928	0.14201
C	-1.45914	0.74611	-0.38494	C	-1.43158	0.71015	-0.37295
C	-0.63392	-0.543	-0.41677	C	-0.57994	-0.56027	-0.46255
C	-1.11446	-1.48705	0.70136	C	-1.0468	-1.57552	0.59844
C	-2.61155	-1.76953	0.56616	C	-2.53986	-1.87539	0.45174
C	-3.3486	2.96841	0.17075	C	-4.8585	1.68465	-1.67772
C	-3.45823	0.1284	2.00642	C	-3.42058	-0.0351	1.94755
C	0.86223	-0.32629	-0.34947	C	0.91073	-0.31803	-0.36872
C	1.7415	-1.38594	-0.83709	C	1.81754	-1.33236	-0.89532
C	3.22361	-1.05724	-0.88061	C	3.29372	-0.98232	-0.88848
C	3.59701	-0.12762	0.25974	C	3.63838	-0.08573	0.28935
O	2.74476	1.05348	0.2352	O	2.7595	1.07447	0.29542
C	1.42936	0.83323	0.06985	C	1.45248	0.83238	0.10685
O	1.31773	-2.46184	-1.275	O	1.42172	-2.39008	-1.40106
C	5.04479	0.39239	0.29329	C	5.08063	0.44098	0.31756
C	6.01242	-0.76643	0.5157	C	5.28019	1.40676	1.49006
O	5.17153	1.23278	1.45583	O	5.2648	1.13896	-0.9264
C	5.40825	1.19055	-0.96117	C	6.06493	-0.72905	0.41819
H	-3.11422	-0.07791	-1.46031	H	-3.03834	-0.10697	-1.51203
H	-0.83473	-1.05053	-1.37058	H	-0.76201	-1.01891	-1.44467
H	3.39087	-0.6235	1.21744	H	3.44313	-0.62128	1.22735
H	-6.53384	-2.15987	-0.11372	H	-6.89326	-1.40239	0.49411
H	-4.8721	-2.95144	0.00884	H	-5.97127	-0.6149	1.8863
H	-5.33287	2.78493	-1.65448	H	-4.17045	3.64019	0.04955
H	-5.04504	1.02939	-2.16788	H	-2.7676	2.77899	0.8862
H	-5.61742	0.01162	0.34957	H	-4.9025	-1.49766	-0.85976
H	-1.26827	1.28229	0.5508	H	-1.26269	1.20026	0.59136
H	-1.14324	1.41131	-1.19742	H	-1.12304	1.42398	-1.14497
H	-0.5588	-2.42879	0.65454	H	-0.47619	-2.50415	0.49821
H	-0.8876	-1.03156	1.67317	H	-0.82745	-1.17335	1.59498
H	-2.77541	-2.28989	-0.38591	H	-2.70332	-2.36443	-0.51789
H	-2.93619	-2.45395	1.35855	H	-2.86088	-2.58865	1.22003
H	-3.29156	2.73527	1.2378	H	-4.43395	1.52865	-2.67816
H	-2.35537	3.33088	-0.12053	H	-5.573	0.87143	-1.51104
H	-4.05564	3.79224	0.03812	H	-5.40977	2.62967	-1.68203
H	-4.18544	0.93996	2.09643	H	-4.09834	0.81616	2.04366
H	-3.71616	-0.62719	2.75645	H	-3.72259	-0.79521	2.67527

H	-2.47315	0.52761	2.26082	H	-2.42098	0.30538	2.2254
H	3.79801	-1.98403	-0.82979	H	3.87255	-1.90681	-0.85654
H	3.42735	-0.58615	-1.85085	H	3.52785	-0.47079	-1.83053
H	0.85581	1.72116	0.31397	H	0.85772	1.69765	0.38089
H	7.01876	-0.36871	0.67618	H	5.0945	0.90748	2.44647
H	6.04171	-1.43142	-0.35062	H	4.61073	2.26542	1.40765
H	5.72749	-1.34942	1.39722	H	6.31296	1.77048	1.4923
H	4.51716	1.94265	1.36563	H	6.18115	1.45253	-0.94601
H	5.3981	0.56231	-1.85626	H	6.01987	-1.36532	-0.46912
H	4.70848	2.01766	-1.11415	H	7.08458	-0.33949	0.50429
H	6.41407	1.60406	-0.84505	H	5.86256	-1.34265	1.30163
atom		Con f. 3- 1m		atom		Con f. 3- 1n	
C	-5.49527	-1.77174	-1.08123	C	-5.99856	-1.27801	-0.12618
C	-4.64547	2.37189	-0.41434	C	-3.47659	1.81536	1.96868
C	-3.684	1.79725	0.31868	C	-3.68716	1.59898	0.66473
C	-2.93077	0.58394	-0.21318	C	-2.88926	0.58557	-0.14503
C	-3.51797	-0.81229	0.23159	C	-3.47262	-0.87669	-0.08027
C	-4.96168	-0.90359	-0.22019	C	-4.85889	-0.8713	-0.68747
C	-1.42143	0.68647	0.07451	C	-1.38696	0.61669	0.18907
C	-0.62014	-0.45259	-0.58193	C	-0.56557	-0.30321	-0.73443
C	-1.1666	-1.80474	-0.1124	C	-1.10173	-1.737	-0.65516
C	-2.65954	-1.91281	-0.429	C	-2.59288	-1.77272	-0.99778
C	-3.29461	2.37312	1.65879	C	-4.72645	2.38548	-0.09203
C	-3.52101	-0.99433	1.76489	C	-3.48458	-1.43729	1.34644
C	0.86758	-0.26215	-0.37886	C	0.917	-0.16793	-0.4621
C	1.55645	0.74082	-1.18602	C	1.61726	1.00423	-0.974
C	2.99749	1.02876	-0.8054	C	3.05223	1.18097	-0.51641
C	3.6769	-0.2277	-0.29242	C	3.73057	-0.16162	-0.29674
O	2.89693	-0.80968	0.7928	O	2.9224	-0.98564	0.59134
C	1.57088	-0.89771	0.59194	C	1.60431	-1.02032	0.33966
O	0.9944	1.38452	-2.07973	O	1.06871	1.85948	-1.6805
C	5.10925	-0.07421	0.24874	C	5.1391	-0.09441	0.31126
C	6.05416	0.33417	-0.87764	C	5.68483	-1.50312	0.56703
O	5.5497	-1.37808	0.67239	O	4.99121	0.60578	1.5591
C	5.18855	0.90184	1.42505	C	6.07756	0.67996	-0.61947
H	-3.04889	0.59615	-1.30405	H	-2.97772	0.88248	-1.19948
H	-0.79181	-0.38421	-1.66479	H	-0.72139	0.05197	-1.76214
H	3.69114	-0.98629	-1.08588	H	3.78615	-0.70505	-1.24874
H	-6.55682	-1.74062	-1.31439	H	-6.93575	-1.2397	-0.67562
H	-4.91671	-2.54016	-1.5851	H	-6.05022	-1.65911	0.88942
H	-5.19444	3.23985	-0.05637	H	-4.06077	2.54994	2.51778
H	-4.91695	1.9925	-1.39627	H	-2.72176	1.27861	2.53453
H	-5.61901	-0.16721	0.23938	H	-4.89045	-0.50054	-1.71349
H	-1.22968	0.6709	1.15316	H	-1.20997	0.31748	1.22766
H	-1.05725	1.6503	-0.29656	H	-1.02866	1.64659	0.09399
H	-0.62219	-2.62066	-0.60211	H	-0.54718	-2.38377	-1.34531
H	-1.00948	-1.92439	0.96581	H	-0.94716	-2.14527	0.34978
H	-2.78675	-1.85535	-1.51723	H	-2.72275	-1.44092	-2.03649
H	-3.03719	-2.89386	-0.11874	H	-2.96875	-2.80126	-0.94729
H	-2.283	2.79517	1.62533	H	-5.47379	1.7237	-0.54323
H	-3.97972	3.17335	1.95279	H	-5.24581	3.09817	0.55554
H	-3.28894	1.61924	2.45119	H	-4.26295	2.94303	-0.91649
H	-4.25292	-0.33657	2.24168	H	-2.49007	-1.42152	1.79683
H	-3.79529	-2.02517	2.01448	H	-4.14956	-0.86423	1.99624
H	-2.5447	-0.79016	2.21164	H	-3.82738	-2.47703	1.34077
H	3.52583	1.42716	-1.67354	H	3.58873	1.76876	-1.26307
H	2.98253	1.80741	-0.03184	H	3.04117	1.75433	0.41916
H	1.11177	-1.55738	1.32122	H	1.1298	-1.83689	0.87512
H	7.08411	0.30866	-0.50993	H	6.68648	-1.43314	1.00354
H	5.84141	1.34686	-1.22791	H	5.76078	-2.07088	-0.36594
H	5.97273	-0.35526	-1.72383	H	5.04256	-2.05173	1.259
H	4.92506	-1.68156	1.34914	H	5.87659	0.70131	1.94041
H	4.94536	1.9234	1.11987	H	5.76648	1.72261	-0.71967
H	4.5006	0.60885	2.22399	H	7.09131	0.66653	-0.20598
H	6.20625	0.89841	1.82562	H	6.1138	0.22692	-1.61516
atom		Con f. 3- 1o		atom		Con f. 3- 1p	
C	5.747	-0.52256	-0.59974	C	-5.94838	-1.01413	0.87521
C	2.5344	2.63582	-0.83542	C	-3.54624	2.75137	0.15701
C	2.94673	1.88397	0.19231	C	-3.76248	1.687	-0.62521
C	2.42336	0.47776	0.45232	C	-2.92593	0.41652	-0.54542
C	3.20648	-0.64475	-0.32889	C	-3.4457	-0.61612	0.52644
C	4.64504	-0.65077	0.14095	C	-4.83862	-1.06091	0.1365
C	0.90418	0.36584	0.23529	C	-1.42553	0.71246	-0.36705
C	0.35628	-1.01737	0.64035	C	-0.57707	-0.55949	-0.46617
C	1.09006	-2.11867	-0.13125	C	-1.04577	-1.5822	0.58672

C	2.60103	-2.01554	0.08817	C	-2.53998	-1.8764	0.43899
C	3.94827	2.41398	1.18579	C	-4.84783	1.70247	-1.67047
C	3.11017	-0.46754	-1.84842	C	-3.41434	-0.04312	1.94771
C	-1.14924	-1.04514	0.50807	C	0.91426	-0.32101	-0.37162
C	-1.96006	-0.46451	1.5749	C	1.81791	-1.32467	-0.9295
C	-3.45659	-0.66896	1.45064	C	3.29002	-0.95674	-0.94318
C	-3.93332	-0.62699	0.00432	C	3.63389	-0.10819	0.26847
O	-3.11543	-1.52404	-0.81008	O	2.76496	1.05177	0.32946
C	-1.79199	-1.568	-0.56479	C	1.45562	0.8161	0.13444
O	-1.47056	0.0716	2.5754	O	1.41791	-2.37423	-1.44678
C	-3.9704	0.78912	-0.62468	C	5.08137	0.41333	0.34152
C	-4.15118	0.7069	-2.14174	C	5.45536	1.27348	-0.8668
O	-2.77042	1.52538	-0.31573	O	5.86282	-0.79417	0.35704
C	-5.10979	1.59024	0.00643	C	5.29417	1.18293	1.64768
H	2.60153	0.26556	1.51578	H	-3.03294	-0.09455	-1.51231
H	0.58111	-1.15192	1.70705	H	-0.76022	-1.00959	-1.45191
H	-4.9349	-1.05316	-0.07678	H	3.43369	-0.69155	1.17757
H	6.73312	-0.56148	-0.14395	H	-6.89302	-1.38619	0.48676
H	5.71878	-0.37347	-1.67505	H	-5.96781	-0.60876	1.88245
H	2.9254	3.63862	-0.98996	H	-4.1593	3.64537	0.07105
H	1.79829	2.28938	-1.55415	H	-2.75995	2.77569	0.90487
H	4.75832	-0.79796	1.21642	H	-4.90245	-1.48466	-0.86712
H	0.64488	0.54897	-0.81347	H	-1.25663	1.19502	0.60097
H	0.40489	1.14295	0.82248	H	-1.11439	1.4312	-1.13344
H	0.73534	-3.10403	0.19329	H	-0.47775	-2.51158	0.47811
H	0.86909	-2.04306	-1.20207	H	-0.82418	-1.18905	1.58632
H	2.81458	-2.18132	1.15239	H	-2.70563	-2.35828	-0.53385
H	3.11866	-2.80899	-0.46338	H	-2.86251	-2.59398	1.20261
H	4.27424	3.42691	0.93137	H	-5.56464	0.88994	-1.51031
H	3.51563	2.43409	2.19472	H	-5.39685	2.6488	-1.67015
H	4.8332	1.77072	1.23792	H	-4.42149	1.5516	-2.67094
H	3.58735	0.45975	-2.17266	H	-4.08925	0.80975	2.04961
H	3.60234	-1.30136	-2.35945	H	-3.71861	-0.80683	2.6707
H	2.0725	-0.44068	-2.18711	H	-2.41355	0.29236	2.22735
H	-3.67782	-1.659	1.87038	H	3.89847	-1.86208	-0.95872
H	-3.9781	0.06821	2.06241	H	3.4784	-0.40595	-1.8737
H	-1.27554	-2.09609	-1.3602	H	0.8622	1.67274	0.43611
H	-4.24052	1.71613	-2.55349	H	4.78788	2.13502	-0.95812
H	-5.05368	0.14481	-2.40141	H	5.40944	0.69553	-1.79358
H	-3.29663	0.213	-2.61239	H	6.47815	1.64685	-0.75
H	-2.04254	1.15435	-0.83599	H	6.79347	-0.53183	0.41605
H	-4.96484	1.71613	1.08252	H	5.03703	0.55535	2.50686
H	-5.14447	2.58437	-0.44799	H	6.34522	1.47782	1.73583
H	-6.07151	1.09806	-0.16166	H	4.68332	2.08885	1.68155
atom	Con f. 3- 1q			atom	Con f. 3- 1r		
C	-5.55607	-1.87991	-0.62913	C	-5.90994	-0.8618	-0.05855
C	-4.58816	2.18866	-1.34714	C	-3.2559	2.65318	0.80033
C	-3.63651	1.8616	-0.46424	C	-3.34731	1.88376	-0.29107
C	-2.90883	0.52694	-0.57612	C	-2.60015	0.56503	-0.43959
C	-3.51587	-0.64096	0.29543	C	-3.37	-0.6705	0.16693
C	-4.97638	-0.81831	-0.06628	C	-4.67928	-0.83773	-0.57269
C	-1.39818	0.69215	-0.32979	C	-1.15617	0.64634	0.08824
C	-0.60813	-0.60824	-0.56402	C	-0.36114	-0.62407	-0.23153
C	-1.20657	-1.77134	0.24642	C	-1.07735	-1.85419	0.35655
C	-2.70505	-1.91518	-0.02168	C	-2.52372	-1.93891	-0.13457
C	-3.2352	2.82754	0.62397	C	-4.18832	2.30584	-1.46825
C	-3.46324	-0.34355	1.80974	C	-3.58992	-0.53227	1.67772
C	0.87173	-0.41854	-0.30315	C	1.09111	-0.57347	0.19114
C	1.38829	-0.10795	1.02657	C	2.02943	-1.50025	-0.42631
C	2.90154	-0.14788	1.1709	C	3.40039	-1.58217	0.2146
C	3.58011	0.33083	-0.09886	C	3.8961	-0.25767	0.78275
O	3.09296	-0.43133	-1.2424	O	2.84092	0.41899	1.53423
C	1.76693	-0.62301	-1.3038	C	1.56285	0.2739	1.13827
O	0.67409	0.1032	2.01445	O	1.72483	-2.24432	-1.36648
C	5.11508	0.23681	-0.1491	C	4.50598	0.75498	-0.21782
C	5.73195	1.15867	0.89884	C	5.80007	0.18605	-0.79623
O	5.53396	0.76134	-1.42321	O	4.89965	1.91096	0.54408
C	5.61726	-1.20106	0.00629	C	3.5551	1.18695	-1.33748
H	-3.03724	0.18802	-1.61217	H	-2.53091	0.35981	-1.51693
H	-0.71791	-0.8666	-1.62665	H	-0.35741	-0.74772	-1.32356
H	3.29645	1.37305	-0.29506	H	4.66363	-0.44429	1.53623
H	-6.62481	-1.88773	-0.8291	H	-6.77827	-1.00643	-0.69632
H	-5.0095	-2.77455	-0.91204	H	-6.10622	-0.73855	1.00232
H	-5.12098	3.13502	-1.28866	H	-3.7994	3.59217	0.87268
H	-4.86896	1.51883	-2.15611	H	-2.6425	2.38332	1.65422

H	-5.60423	0.03518	0.18459	H	-4.56569	-0.96822	-1.65023
H	-1.2156	1.04167	0.68816	H	-1.16867	0.81605	1.16954
H	-1.013	1.46904	-1.00099	H	-0.66309	1.51707	-0.35857
H	-0.69318	-2.70119	-0.02541	H	-0.54181	-2.76564	0.07069
H	-1.0237	-1.61199	1.31243	H	-1.04107	-1.79665	1.45114
H	-2.84917	-2.16893	-1.07977	H	-2.51464	-2.10073	-1.22066
H	-3.10833	-2.75183	0.56072	H	-3.02395	-2.80882	0.30722
H	-3.23618	2.36306	1.61413	H	-3.56874	2.40226	-2.36955
H	-2.21827	3.20368	0.45996	H	-4.95569	1.55786	-1.69478
H	-3.90875	3.6889	0.64901	H	-4.68555	3.26378	-1.28903
H	-4.16066	0.45302	2.08292	H	-4.22523	0.32465	1.91246
H	-3.75282	-1.23821	2.37203	H	-4.0718	-1.43226	2.07315
H	-2.46564	-0.04786	2.14312	H	-2.64749	-0.40243	2.21385
H	3.19361	0.47205	2.0204	H	3.31125	-2.30771	1.03366
H	3.18127	-1.18491	1.39634	H	4.116	-1.99136	-0.50026
H	1.46346	-0.97746	-2.28587	H	0.9214	0.95563	1.68733
H	6.8153	1.19521	0.75162	H	6.31307	0.9703	-1.36052
H	5.53671	0.79687	1.91099	H	5.60355	-0.64697	-1.47513
H	5.33699	2.17524	0.80572	H	6.46733	-0.15827	0.00017
H	5.09438	0.23192	-2.10637	H	4.1028	2.23011	0.99578
H	5.38402	-1.60466	0.99559	H	2.66035	1.67046	-0.93639
H	5.16735	-1.85556	-0.74627	H	4.07088	1.90761	-1.97922
H	6.70314	-1.21849	-0.12311	H	3.24381	0.34195	-1.95854

atom	Con f. 3- 1s		
C	5.76976	-0.60793	-0.63769
C	2.59394	2.60144	-0.93559
C	3.01781	1.8798	0.10889
C	2.47923	0.49192	0.4299
C	3.2324	-0.66955	-0.3218
C	4.67822	-0.68304	0.1254
C	0.95494	0.39626	0.24286
C	0.39226	-0.96099	0.71126
C	1.09626	-2.1002	-0.0327
C	2.61261	-2.01394	0.15494
C	4.04842	2.42898	1.06158
C	3.11477	-0.54458	-1.84507
C	-1.11695	-0.97236	0.60336
C	-1.89745	-0.28394	1.62121
C	-3.40001	-0.46805	1.54575
C	-3.93145	-0.61036	0.12461
O	-3.10656	-1.53479	-0.65063
C	-1.78221	-1.57138	-0.41489
O	-1.38492	0.34815	2.55325
C	-4.11245	0.68956	-0.69778
C	-5.19812	1.55146	-0.05615
O	-4.63661	0.3015	-1.98121
C	-2.82794	1.49871	-0.90036
H	2.67347	0.31716	1.49734
H	0.63346	-1.05887	1.77855
H	-4.90799	-1.0977	0.14359
H	6.76258	-0.64754	-0.19674
H	5.7261	-0.5031	-1.7177
H	2.99608	3.59208	-1.13374
H	1.83762	2.24089	-1.62575
H	4.8067	-0.78789	1.20413
H	0.67973	0.54306	-0.80704
H	0.48191	1.20483	0.80782
H	0.73308	-3.0686	0.33135
H	0.85677	-2.05718	-1.10143
H	2.84403	-2.14538	1.22019
H	3.10635	-2.8352	-0.37736
H	4.92668	1.77668	1.11492
H	4.38084	3.42882	0.76682
H	3.63957	2.4875	2.07891
H	3.60603	0.36035	-2.20925
H	3.58062	-1.40636	-2.3339
H	2.07271	-0.50668	-2.16884
H	-3.62651	-1.38713	2.10193
H	-3.89673	0.352	2.06707
H	-1.28152	-2.1618	-1.17555
H	-5.45682	2.36506	-0.74009
H	-4.85481	1.99352	0.882
H	-6.10126	0.96493	0.13977
H	-4.00562	-0.32995	-2.36079
H	-2.07357	0.92104	-1.44059

H	-3.06201	2.38839	-1.49285
H	-2.3971	1.82747	0.04954

atom	Con f. 3-2a			atom	Con f. 3-2b		
C	-6.00491	-1.1863	0.04077	C	-5.99539	-0.96399	0.72119
C	-3.33064	1.90441	1.95317	C	-3.53186	2.75919	0.20142
C	-3.60149	1.65045	0.66722	C	-3.72107	1.72548	-0.62757
C	-2.85731	0.59701	-0.14269	C	-2.90285	0.44291	-0.55339
C	-3.46977	-0.84903	-0.01788	C	-3.47457	-0.6114	0.47001
C	-4.87685	-0.8289	-0.5749	C	-4.86095	-1.01978	0.02167
C	-1.34386	0.60113	0.13745	C	-1.40603	0.71249	-0.31239
C	-0.57674	-0.35793	-0.79412	C	-0.57416	-0.57087	-0.41879
C	-1.13963	-1.77625	-0.65607	C	-1.09467	-1.61844	0.58302
C	-2.64343	-1.78895	-0.9408	C	-2.587	-1.88402	0.37519
C	-4.655	2.4358	-0.07089	C	-4.75665	1.7887	-1.7203
C	-3.44234	-1.37019	1.42346	C	-3.48058	-0.08108	1.90832
C	0.91416	-0.24258	-0.56594	C	0.9179	-0.35956	-0.27771
C	1.62374	0.89699	-1.13875	C	1.81821	-1.37935	-0.80568
C	3.13267	0.89451	-0.987	C	3.28697	-1.21893	-0.46702
C	3.56085	0.22282	0.30583	C	3.68223	0.24335	-0.36093
O	2.94567	-1.0951	0.40543	O	2.77218	0.94394	0.53738
C	1.64205	-1.18055	0.08971	C	1.46136	0.67516	0.40991
O	1.05559	1.78488	-1.78618	O	1.41766	-2.36746	-1.43291
C	5.07735	-0.01024	0.45116	C	5.09654	0.4965	0.19797
C	5.3851	-0.71344	1.77746	C	5.38097	2.00027	0.27102
O	5.54097	-0.81192	-0.64853	O	5.19522	-0.0847	1.50909
C	5.83345	1.31312	0.36008	C	6.14638	-0.2066	-0.65868
H	-2.977	0.86869	-1.20088	H	-2.98064	-0.04004	-1.53745
H	-0.76305	-0.02335	-1.82394	H	-0.72993	-0.98581	-1.42423
H	3.19421	0.7983	1.16534	H	3.58095	0.73001	-1.33926
H	-6.96049	-1.14082	-0.47542	H	-6.93307	-1.30758	0.29178
H	-6.0289	-1.53264	1.06974	H	-6.04219	-0.57899	1.73545
H	-3.87646	2.66814	2.50191	H	-4.13139	3.66262	0.11877
H	-2.5633	1.37021	2.50447	H	-2.78062	2.74875	0.98481
H	-4.93721	-0.49059	-1.61079	H	-4.89725	-1.42106	-0.99253
H	-1.13685	0.31958	1.17539	H	-1.26915	1.16666	0.67398
H	-0.96643	1.6197	0.00678	H	-1.05411	1.44684	-1.0456
H	-0.62839	-2.45468	-1.34938	H	-0.5374	-2.55346	0.46464
H	-0.95242	-2.15877	0.35391	H	-0.90205	-1.26073	1.60179
H	-2.80598	-1.48461	-1.98316	H	-2.72609	-2.33318	-0.61727
H	-3.03735	-2.80751	-0.8459	H	-2.9473	-2.61892	1.10471
H	-5.1364	3.1751	0.57616	H	-5.2969	2.74002	-1.71204
H	-4.21306	2.96275	-0.92679	H	-4.28586	1.66793	-2.70481
H	-5.4305	1.77687	-0.47609	H	-5.48696	0.97827	-1.6213
H	-4.06734	-0.76148	2.08054	H	-2.48441	0.23033	2.22935
H	-3.81185	-2.4001	1.46024	H	-4.14563	0.77888	2.01468
H	-2.43137	-1.36901	1.83605	H	-3.81954	-0.86103	2.5978
H	3.56294	0.35729	-1.84228	H	3.47032	-1.72199	0.4916
H	3.48912	1.92529	-1.02471	H	3.88084	-1.7242	-1.23049
H	1.2248	-2.12334	0.42824	H	0.86531	1.40955	0.94237
H	6.46177	-0.88954	1.85407	H	6.37795	2.16213	0.69063
H	5.07237	-0.09846	2.62738	H	5.34589	2.45391	-0.72472
H	4.87066	-1.67571	1.84377	H	4.65104	2.51065	0.90499
H	5.05297	-1.6495	-0.6162	H	4.51825	0.34014	2.05881
H	5.73315	1.7614	-0.63147	H	6.07105	0.11005	-1.70294
H	5.46324	2.02316	1.10515	H	7.14514	0.04874	-0.29306
H	6.89687	1.1395	0.54837	H	6.03457	-1.29266	-0.61247

atom	Con f. 3-2c			atom	Con f. 3-2d		
C	-5.95616	-1.25344	0.15179	C	-5.57903	-1.67251	-0.91785
C	-3.54013	2.35537	1.17591	C	-4.56127	2.45021	-0.37332
C	-3.72832	1.76677	-0.01134	C	-3.58929	1.85808	0.33138
C	-2.89692	0.58378	-0.49051	C	-2.90109	0.60883	-0.20554
C	-3.44196	-0.81025	0.00082	C	-3.51605	-0.75736	0.29106
C	-4.84205	-1.0042	-0.53825	C	-4.97982	-0.80682	-0.09855
C	-1.40187	0.7512	-0.16734	C	-1.37869	0.66429	0.01893
C	-0.53894	-0.37854	-0.76006	C	-0.64043	-0.51353	-0.64498
C	-1.06861	-1.76467	-0.34786	C	-1.21551	-1.83691	-0.13154
C	-2.55937	-1.90687	-0.65749	C	-2.72333	-1.89779	-0.3831
C	-4.77625	2.27306	-0.96883	C	-3.12323	2.44813	1.64046
C	-3.39925	-0.9445	1.52755	C	-3.46092	-0.91031	1.82652
C	0.92763	-0.20926	-0.42336	C	0.85566	-0.36564	-0.4782
C	1.40905	-0.19768	0.95403	C	1.56888	0.5626	-1.3492
C	2.87362	0.15617	1.14138	C	3.08017	0.57348	-1.22514
C	3.72538	-0.34086	-0.01289	C	3.52307	0.27401	0.19618

O	3.16078	0.11809	-1.27724	O	2.89181	-0.95442	0.66354
C	1.82946	0.01999	-1.4117	C	1.5821	-1.10108	0.39989
O	0.68514	-0.38681	1.93995	O	0.99905	1.25218	-2.20365
C	5.18827	0.14443	-0.00555	C	5.03826	0.05928	0.37878
C	5.93851	-0.40434	-1.22389	C	5.36035	-0.24659	1.84526
O	5.21063	1.58181	-0.02216	O	5.46364	-1.03238	-0.4546
C	5.88441	-0.27573	1.28647	C	5.81486	1.28482	-0.09616
H	-2.98577	0.55578	-1.58587	H	-3.06433	0.60454	-1.29064
H	-0.62869	-0.31316	-1.85359	H	-0.84395	-0.45614	-1.72305
H	3.7036	-1.43744	-0.04861	H	3.18127	1.07045	0.86937
H	-6.90747	-1.39287	-0.35546	H	-6.64784	-1.60857	-1.10701
H	-5.97232	-1.32737	1.23509	H	-5.04943	-2.47044	-1.42955
H	-4.15023	3.20013	1.48668	H	-5.06535	3.34366	-0.01211
H	-2.77951	2.02564	1.87643	H	-4.88677	2.06049	-1.33458
H	-4.90958	-0.94602	-1.62606	H	-5.59133	-0.03889	0.37244
H	-1.24805	0.79613	0.91244	H	-1.14372	0.6617	1.08916
H	-1.05988	1.71081	-0.57134	H	-0.99609	1.60735	-0.38506
H	-0.50625	-2.53911	-0.88292	H	-0.72347	-2.68026	-0.63033
H	-0.88491	-1.92419	0.71767	H	-1.01375	-1.94482	0.94084
H	-2.70515	-1.86221	-1.74521	H	-2.8952	-1.85526	-1.4658
H	-2.91862	-2.89095	-0.33351	H	-3.12002	-2.85982	-0.03891
H	-5.32802	3.12174	-0.55389	H	-3.76967	3.27549	1.94693
H	-4.31429	2.59042	-1.91303	H	-2.10185	2.83776	1.55312
H	-5.49535	1.48643	-1.22169	H	-3.10487	1.71096	2.44829
H	-4.06086	-0.22134	2.00986	H	-4.14881	-0.21808	2.31964
H	-3.71591	-1.94858	1.82817	H	-3.75983	-1.92584	2.10842
H	-2.39303	-0.7857	1.91995	H	-2.46014	-0.73253	2.22751
H	2.95419	1.24853	1.21624	H	3.48525	-0.18554	-1.90703
H	3.21495	-0.27148	2.08543	H	3.45108	1.54742	-1.54911
H	1.52775	0.14928	-2.44843	H	1.15815	-1.90761	0.98951
H	6.96732	-0.03336	-1.21424	H	5.07858	0.59185	2.49029
H	5.96389	-1.4986	-1.20663	H	6.43463	-0.42123	1.95312
H	5.46442	-0.08779	-2.15666	H	4.82791	-1.13705	2.18972
H	4.75338	1.86446	-0.82958	H	4.95293	-1.81124	-0.1833
H	5.82062	-1.35839	1.42816	H	5.47454	2.18532	0.42315
H	6.94088	0.00351	1.23836	H	6.87905	1.14663	0.11563
H	5.44137	0.21837	2.15458	H	5.69891	1.43562	-1.17226
atom	Con f. 3-2e			atom	Con f. 3-2f		
C	-5.51261	-1.96679	-0.1524	C	-6.00309	-1.17156	0.09219
C	-4.63734	1.97015	-1.6105	C	-3.32065	1.97176	1.884
C	-3.68854	1.82928	-0.6768	C	-3.58989	1.67596	0.60672
C	-2.93767	0.51021	-0.5348	C	-2.85035	0.59162	-0.16578
C	-3.53891	-0.48768	0.5313	C	-3.46691	-0.84729	0.01209
C	-4.97907	-0.78601	0.1649	C	-4.87575	-0.84304	-0.5407
C	-1.43163	0.7461	-0.311	C	-1.33643	0.60073	0.11155
C	-0.63388	-0.56102	-0.3395	C	-0.57516	-0.39272	-0.78818
C	-1.18797	-1.52411	0.7259	C	-1.14135	-1.80393	-0.59895
C	-2.68168	-1.77096	0.5076	C	-2.64607	-1.82212	-0.87878
C	-3.30975	2.99028	0.2104	C	-4.638	2.44056	-0.16027
C	-3.5605	0.1046	1.9569	C	-3.43646	-1.31574	1.47136
C	0.86313	-0.37736	-0.2144	C	0.91739	-0.27392	-0.5716
C	1.73729	-1.46274	-0.6486	C	1.62702	0.83688	-1.20009
C	3.213	-1.29941	-0.3415	C	3.14009	0.82182	-1.06996
C	3.63239	0.15914	-0.391	C	3.55587	0.22257	0.26125
O	2.75291	0.95682	0.4553	O	2.94801	-1.0914	0.42674
C	1.43489	0.70727	0.3649	C	1.64241	-1.18615	0.12364
O	1.3102	-2.49739	-1.1751	O	1.05755	1.70215	-1.87541
C	5.06339	0.44666	0.1035	C	5.0632	0.02443	0.49792
C	5.36251	1.94774	0.0269	C	5.77069	1.37605	0.53202
O	5.19647	-0.01148	1.4597	O	5.22059	-0.53161	1.81654
C	6.08122	-0.34487	-0.7137	C	5.69771	-0.91251	-0.53317
H	-3.0433	-0.01439	-1.4928	H	-2.97108	0.82545	-1.23284
H	-0.80186	-1.03625	-1.3161	H	-0.76476	-0.09403	-1.82837
H	3.51214	0.54781	-1.4103	H	3.15989	0.83821	1.0795
H	-6.57154	-2.05318	-0.3836	H	-6.96028	-1.14225	-0.42219
H	-4.93592	-2.88583	-0.1963	H	-6.02484	-1.47776	1.13386
H	-5.18363	2.90252	-1.7333	H	-3.86356	2.75661	2.40519
H	-4.9014	1.15668	-2.2816	H	-2.55746	1.4524	2.45497
H	-5.63386	0.08366	0.186	H	-4.93846	-0.54502	-1.58873
H	-1.28011	1.25189	0.6483	H	-1.12884	0.35435	1.15827
H	-1.05749	1.42634	-1.0853	H	-0.95576	1.61278	-0.05524
H	-0.64993	-2.47634	0.682	H	-0.63401	-2.50725	-1.26992
H	-1.00306	-1.09888	1.7202	H	-0.95214	-2.15221	0.423
H	-2.80726	-2.26168	-0.4657	H	-2.81058	-1.55471	-1.93087
H	-3.0608	-2.46924	1.2628	H	-3.04266	-2.83547	-0.74641

H	-3.9889	3.83388	0.0578	H	-4.19158	2.93769	-1.03155
H	-3.32481	2.72736	1.2718	H	-5.41438	1.77114	-0.54624
H	-2.29248	3.33652	-0.0082	H	-5.11901	3.20228	0.46054
H	-4.27789	0.92596	2.0361	H	-2.42393	-1.30363	1.88004
H	-3.86641	-0.66723	2.6715	H	-4.05639	-0.68019	2.10745
H	-2.58244	0.47922	2.2693	H	-3.81008	-2.34203	1.54778
H	3.39881	-1.70268	0.6624	H	3.53609	0.22989	-1.90517
H	3.78805	-1.89264	-1.0545	H	3.51768	1.8409	-1.17116
H	0.85971	1.50366	0.8264	H	1.22122	-2.10941	0.50759
H	4.65784	2.52355	0.6327	H	6.8104	1.2302	0.83933
H	6.37386	2.13595	0.3983	H	5.76836	1.84959	-0.45245
H	5.29939	2.30604	-1.0054	H	5.29033	2.05001	1.24835
H	4.53621	0.46572	1.9861	H	4.70644	-1.3534	1.84014
H	5.96538	-1.42019	-0.5574	H	5.1816	-1.87706	-0.55434
H	7.0929	-0.06335	-0.4076	H	5.66884	-0.48561	-1.53962
H	5.97255	-0.13145	-1.781	H	6.74402	-1.085	-0.26535
atom	Con f. 3-2g			atom	Con f. 3-2h		
C	-5.49401	-2.04998	-0.47158	C	-6.00378	-0.95136	0.6835
C	-4.68493	1.98857	-1.48228	C	-3.51915	2.7571	0.24452
C	-3.72686	1.75946	-0.57571	C	-3.70528	1.73958	-0.60496
C	-2.94293	0.45296	-0.59942	C	-2.89408	0.45181	-0.54669
C	-3.49531	-0.67874	0.35158	C	-3.47848	-0.61697	0.45438
C	-4.95114	-0.93437	0.0188	C	-4.865	-1.00779	-0.0089
C	-1.43932	0.69628	-0.37526	C	-1.39789	0.70945	-0.28987
C	-0.59969	-0.58335	-0.53724	C	-0.57175	-0.57645	-0.41264
C	-1.14448	-1.71958	0.34687	C	-1.10583	-1.64095	0.56372
C	-2.63822	-1.93854	0.10526	C	-2.59829	-1.89329	0.34118
C	-3.37435	2.81121	0.44814	C	-4.72979	1.82784	-1.70638
C	-3.43878	-0.28483	1.84378	C	-3.48939	-0.11269	1.90201
C	0.87467	-0.33228	-0.30072	C	0.92018	-0.3759	-0.25363
C	1.38323	0.16101	0.97441	C	1.81927	-1.38439	-0.80819
C	2.85705	0.52618	1.00431	C	3.28699	-1.24186	-0.44698
C	3.6804	-0.36009	0.08676	C	3.66641	0.22158	-0.31511
O	3.09349	-0.37275	-1.2485	O	2.7711	0.88849	0.62162
C	1.75894	-0.48693	-1.31893	C	1.45974	0.63194	0.47699
O	0.67556	0.35696	1.97092	O	1.41888	-2.34946	-1.46931
C	5.14597	0.07705	-0.10623	C	5.0938	0.53239	0.16819
C	5.86597	-0.88036	-1.06168	C	6.1125	0.04156	-0.85632
O	5.17518	1.41239	-0.63726	O	5.23297	1.96529	0.19987
C	5.86797	0.13768	1.23745	C	5.37753	-0.04439	1.55737
H	-3.05964	0.03917	-1.60936	H	-2.96697	-0.01392	-1.53936
H	-0.70611	-0.91156	-1.58079	H	-0.72018	-0.97059	-1.42737
H	3.65177	-1.39745	0.44313	H	3.51578	0.72603	-1.27864
H	-6.56334	-2.11074	-0.65893	H	-6.94149	-1.28049	0.24297
H	-4.91576	-2.93873	-0.70573	H	-6.05418	-0.57968	1.70253
H	-5.25622	2.91405	-1.48889	H	-4.11347	3.66489	0.17252
H	-4.93311	1.25584	-2.24599	H	-2.77537	2.72905	1.03459
H	-5.60972	-0.09181	0.22352	H	-4.89775	-1.39454	-1.02885
H	-1.26693	1.11756	0.61711	H	-1.26645	1.14652	0.70486
H	-1.09039	1.44393	-1.09757	H	-1.03641	1.45411	-1.00784
H	-0.59578	-2.64274	0.12596	H	-0.5532	-2.57674	0.4295
H	-0.95751	-1.48823	1.39886	H	-0.91747	-1.30602	1.59095
H	-2.78144	-2.2642	-0.93306	H	-2.73377	-2.32267	-0.66048
H	-3.00286	-2.75174	0.74371	H	-2.96779	-2.63961	1.05435
H	-2.36348	3.20121	0.2797	H	-5.46382	1.0184	-1.63003
H	-3.38785	2.42005	1.46957	H	-5.26656	2.78094	-1.6852
H	-4.06991	3.65361	0.39551	H	-4.24962	1.72415	-2.6883
H	-4.16737	0.49622	2.07749	H	-2.49318	0.18643	2.23433
H	-3.68162	-1.15439	2.46459	H	-4.14959	0.74944	2.02019
H	-2.45178	0.0746	2.14442	H	-3.83747	-0.90281	2.57518
H	2.9532	1.57378	0.69037	H	3.44193	-1.77398	0.50046
H	3.21272	0.45026	2.03326	H	3.89424	-1.73096	-1.21091
H	1.43733	-0.72841	-2.32925	H	0.86195	1.34725	1.03272
H	6.89605	-0.54267	-1.20681	H	7.10786	0.39429	-0.57068
H	5.37133	-0.91351	-2.03607	H	6.13647	-1.04981	-0.90097
H	5.88769	-1.89615	-0.6541	H	5.88194	0.43014	-1.85327
H	4.70056	1.39116	-1.4829	H	4.54615	2.30416	0.79473
H	5.44951	0.91845	1.87728	H	4.64351	0.31224	2.28625
H	5.79588	-0.82041	1.76006	H	5.35295	-1.13775	1.55221
H	6.92589	0.36205	1.07372	H	6.37204	0.27372	1.88303
atom	Con f. 3-2i			atom	Con f. 3-2j		
C	5.98432	-0.84061	-0.87925	C	-5.95626	-1.25365	0.11313
C	3.3936	2.75702	-0.14195	C	-3.53912	2.31815	1.25269
C	3.65509	1.70204	0.63919	C	-3.72665	1.76758	0.04727
C	2.88472	0.3913	0.54545	C	-2.89517	0.60031	-0.46872

C	3.46112	-0.60498	-0.53092	C	-3.44174	-0.8086	-0.02326
C	4.87813	-0.96976	-0.14516	C	-4.84174	-0.98368	-0.56837
C	1.37178	0.60812	0.36609	C	-1.40081	0.75457	-0.13704
C	0.58212	-0.71063	0.42626	C	-0.538	-0.35511	-0.76684
C	1.12277	-1.70733	-0.6119	C	-1.06863	-1.75376	-0.40149
C	2.62918	-1.91581	-0.44457	C	-2.55956	-1.88386	-0.71657
C	4.72592	1.76818	1.69724	C	-4.77418	2.30375	-0.89428
C	3.39328	-0.02963	-1.94986	C	-3.39866	-0.99207	1.49847
C	-0.9101	-0.48707	0.3017	C	0.92849	-0.19214	-0.42534
C	-1.80582	-0.89581	1.37706	C	1.40888	-0.2277	0.95269
C	-3.29018	-0.78191	1.08571	C	2.8683	0.14848	1.15258
C	-3.59527	0.38486	0.16217	C	3.71482	-0.30797	-0.02138
O	-2.75373	0.31354	-1.02724	O	3.15394	0.19935	-1.26803
C	-1.46044	0.01098	-0.83413	C	1.82453	0.08909	-1.40648
O	-1.40882	-1.38191	2.44302	O	0.68853	-0.46584	1.93
C	-5.04584	0.44492	-0.35547	C	5.19507	0.11297	-0.01935
C	-5.23083	1.64662	-1.28813	C	5.9225	-0.53497	1.15484
O	-5.34825	-0.77252	-1.05706	O	5.79966	-0.43992	-1.20366
C	-6.02769	0.51866	0.81132	C	5.3708	1.63363	-0.01612
H	3.0139	-0.12137	1.50903	H	-2.98221	0.60802	-1.56454
H	0.7428	-1.1485	1.41845	H	-0.62789	-0.25329	-1.85758
H	-3.3433	1.33053	0.65845	H	3.67113	-1.4019	-0.10148
H	6.94979	-1.15853	-0.49392	H	-6.90769	-1.37527	-0.39846
H	5.98018	-0.41833	-1.87979	H	-5.97254	-1.36323	1.19342
H	3.95951	3.68069	-0.04675	H	-4.14936	3.15255	1.58991
H	2.61478	2.74404	-0.89792	H	-2.77902	1.96612	1.94283
H	4.96679	-1.40436	0.85199	H	-4.90914	-0.88966	-1.65368
H	1.16611	1.10964	-0.58548	H	-1.24984	0.76145	0.94416
H	1.01594	1.28309	1.1525	H	-1.05643	1.72707	-0.50666
H	0.60457	-2.6674	-0.50441	H	-0.50678	-2.51058	-0.96167
H	0.90019	-1.34533	-1.62254	H	-0.88632	-1.94883	0.65832
H	2.81173	-2.379	0.53434	H	-2.70474	-1.80367	-1.80228
H	3.00128	-2.61929	-1.19877	H	-2.91989	-2.87759	-0.42479
H	5.23142	2.73847	1.70246	H	-5.49195	1.52487	-1.17333
H	4.29436	1.59909	2.69258	H	-5.32755	3.13778	-0.45252
H	5.48038	0.98838	1.54698	H	-4.31162	2.65234	-1.8271
H	4.01647	0.86194	-2.04929	H	-4.06122	-0.28623	2.00467
H	3.74081	-0.77088	-2.67681	H	-3.71316	-2.00601	1.76646
H	2.37394	0.24769	-2.2258	H	-2.39245	-0.84382	1.89486
H	-3.6178	-1.71883	0.61655	H	2.91182	1.23939	1.2649
H	-3.82551	-0.67469	2.0306	H	3.22491	-0.29943	2.08185
H	-0.87413	0.21687	-1.72583	H	1.51837	0.25668	-2.43626
H	-6.25876	1.6623	-1.66135	H	6.99504	-0.33931	1.0658
H	-5.03951	2.58524	-0.75832	H	5.57854	-0.1279	2.10836
H	-4.55215	1.59211	-2.14346	H	5.7687	-1.61865	1.15854
H	-4.71997	-0.8416	-1.79296	H	5.31357	-0.08466	-1.96373
H	-6.00283	-0.39444	1.41116	H	4.83592	2.09076	-0.85406
H	-5.79641	1.37004	1.45781	H	5.00358	2.08242	0.91117
H	-7.0435	0.6445	0.42578	H	6.43396	1.87181	-0.11219
atom	Con f. 3-2k			atom	Con f. 3-2l		
C	-5.58827	-1.67872	-0.84337	C	-6.01164	-1.15693	0.08054
C	-4.54399	2.44309	-0.45989	C	-3.30321	1.93086	1.93588
C	-3.5686	1.87557	0.26018	C	-3.58246	1.66564	0.654
C	-2.89183	0.60094	-0.22993	C	-2.8521	0.59681	-0.14831
C	-3.51244	-0.74001	0.32509	C	-3.47403	-0.84308	-0.00166
C	-4.97947	-0.79423	-0.05165	C	-4.88504	-0.8188	-0.54857
C	-1.36773	0.65511	-0.01688	C	-1.33675	0.59304	0.1209
C	-0.64125	-0.55369	-0.63683	C	-0.58316	-0.38274	-0.80435
C	-1.22248	-1.8521	-0.06922	C	-1.15522	-1.79514	-0.64526
C	-2.73223	-1.91145	-0.30963	C	-2.66125	-1.8003	-0.91877
C	-3.08705	2.51719	1.53898	C	-4.63196	2.45285	-0.08779
C	-3.44632	-0.83219	1.86477	C	-3.44007	-1.34677	1.44572
C	0.85683	-0.40987	-0.48368	C	0.90997	-0.27484	-0.5876
C	1.57037	0.47492	-1.40016	C	1.62271	0.85193	-1.17992
C	3.08497	0.46966	-1.28953	C	3.13259	0.84241	-1.03215
C	3.51166	0.25122	0.15052	C	3.54825	0.19006	0.27597
O	2.89313	-0.96064	0.67265	O	2.94258	-1.12999	0.37856
C	1.5812	-1.11214	0.42373	C	1.63759	-1.21085	0.07317
O	0.99986	1.13437	-2.27683	O	1.05754	1.73348	-1.83917
C	5.01988	0.10865	0.42032	C	5.06033	0.00827	0.47227
C	5.7381	1.41209	0.08351	C	5.34829	-0.74429	1.77527
O	5.18574	-0.07086	1.83933	O	5.50833	-0.77039	-0.65125
C	5.63736	-1.07748	-0.32491	C	5.75674	1.3731	0.47973
H	-3.06197	0.55375	-1.31292	H	-2.97757	0.8566	-1.20882
H	-0.84983	-0.53747	-1.7154	H	-0.77455	-0.05923	-1.83681

H	3.1303	1.07123	0.77312	H	3.15392	0.77264	1.11849
H	-6.6581	-1.61485	-1.02672	H	-6.97065	-1.10998	-0.42915
H	-5.06585	-2.4923	-1.33751	H	-6.03108	-1.48851	1.11445
H	-5.04009	3.35371	-0.13202	H	-3.83938	2.7055	2.47884
H	-4.88032	2.01475	-1.40072	H	-2.53821	1.39562	2.48943
H	-5.58448	-0.01129	0.40274	H	-4.95023	-0.49465	-1.58869
H	-1.12658	0.69557	1.0511	H	-1.12467	0.32249	1.16075
H	-0.98125	1.57806	-0.46156	H	-0.95274	1.60712	-0.02478
H	-0.7395	-2.71754	-0.53815	H	-0.65412	-2.48541	-1.33422
H	-1.01516	-1.9205	1.00527	H	-0.96289	-2.16705	0.36773
H	-2.90998	-1.90847	-1.39226	H	-2.82915	-1.50762	-1.96361
H	-3.1341	-2.85671	0.07294	H	-3.06188	-2.81471	-0.80848
H	-3.72587	3.36053	1.81645	H	-5.10557	3.2017	0.55403
H	-2.06426	2.89643	1.42612	H	-4.1884	2.96875	-0.9496
H	-3.06543	1.81425	2.37666	H	-5.41378	1.79652	-0.485
H	-4.12122	-0.11181	2.33501	H	-4.05544	-0.72495	2.09958
H	-3.75544	-1.83177	2.18956	H	-3.8173	-2.37324	1.49809
H	-2.44006	-0.65182	2.25058	H	-2.42604	-1.34841	1.85079
H	3.4605	-0.33568	-1.93373	H	3.55653	0.28782	-1.87864
H	3.47392	1.41539	-1.6715	H	3.49559	1.86988	-1.08869
H	1.15468	-1.88881	1.05007	H	1.21664	-2.14858	0.42138
H	6.7782	1.34516	0.41576	H	6.42984	-0.8635	1.89751
H	5.26749	2.25971	0.59164	H	4.96828	-0.19135	2.6403
H	5.73453	1.6007	-0.99247	H	4.88964	-1.73508	1.76414
H	4.67678	-0.85794	2.08775	H	6.46982	-0.86007	-0.57343
H	5.11252	-2.00701	-0.08481	H	5.66108	1.87365	-0.48691
H	5.6044	-0.93418	-1.40868	H	5.34406	2.02581	1.25538
H	6.68414	-1.18339	-0.02615	H	6.8232	1.23598	0.68653
atom		Con f. 3-2m		atom		Con f. 3-2n	
C	-5.51776	-1.94516	-0.23085	C	5.36759	-1.83204	-0.63754
C	-4.60594	2.01098	-1.59508	C	3.67802	2.3088	-1.08667
C	-3.6684	1.8468	-0.65382	C	3.81203	1.54049	0.00106
C	-2.9269	0.52064	-0.52815	C	2.78568	0.49003	0.40548
C	-3.54603	-0.49269	0.5131	C	3.00794	-0.90926	-0.28524
C	-4.98333	-0.77553	0.12412	C	4.35412	-1.45262	0.14244
C	-1.42226	0.7434	-0.28353	C	1.33852	0.98696	0.22987
C	-0.63122	-0.56753	-0.32501	C	0.32203	-0.00755	0.80114
C	-1.20348	-1.54526	0.71741	C	0.50057	-1.38286	0.12904
C	-2.69552	-1.78017	0.47582	C	1.93764	-1.88558	0.27816
C	-3.29286	2.98886	0.25907	C	4.99454	1.70039	0.9214
C	-3.57992	0.07372	1.94894	C	2.89183	-0.82741	-1.81132
C	0.86538	-0.3931	-0.18013	C	-1.11649	0.4487	0.71233
C	1.73765	-1.48484	-0.60637	C	-2.0937	-0.13298	1.62908
C	3.21142	-1.32894	-0.27555	C	-3.48533	0.46606	1.58194
C	3.61849	0.13109	-0.35158	C	-3.88757	0.90588	0.18034
O	2.75335	0.93089	0.50679	O	-2.8322	1.73658	-0.39587
C	1.43446	0.68963	0.40702	C	-1.55455	1.36832	-0.18197
O	1.31119	-2.51462	-1.14146	O	-1.79761	-0.98894	2.47024
C	5.06111	0.47897	0.05465	C	-4.25196	-0.25127	-0.78471
C	6.04812	-0.1521	-0.92285	C	-4.31771	0.24543	-2.23048
O	5.21852	1.90192	-0.09953	O	-3.30285	-1.33151	-0.6828
C	5.37418	0.08054	1.49903	C	-5.59333	-0.85249	-0.36346
H	-3.0253	0.01415	-1.49657	H	2.92725	0.30621	1.47952
H	-0.79008	-1.02523	-1.31152	H	0.54562	-0.14094	1.86847
H	3.45233	0.50465	-1.37047	H	-4.74412	1.58074	0.22596
H	-6.5742	-2.02074	-0.47672	H	6.28512	-2.22578	-0.20765
H	-4.94424	-2.86514	-0.29268	H	5.33211	-1.76752	-1.72103
H	-5.14525	2.94886	-1.70614	H	4.42739	3.05351	-1.34384
H	-4.86735	1.21207	-2.28432	H	2.82694	2.23009	-1.75563
H	-5.63492	0.09603	0.16073	H	4.46899	-1.54925	1.22349
H	-1.27903	1.23183	0.68606	H	1.13413	1.15827	-0.83199
H	-1.03548	1.43472	-1.04167	H	1.23356	1.95695	0.72856
H	-0.67016	-2.49956	0.66474	H	-0.19062	-2.1048	0.57553
H	-1.02933	-1.13723	1.72072	H	0.22908	-1.29674	-0.93032
H	-2.81118	-2.25205	-0.50807	H	2.14137	-2.04857	1.34479
H	-3.08798	-2.49028	1.21299	H	2.05398	-2.85759	-0.21542
H	-2.27076	3.33219	0.05914	H	4.66195	1.97369	1.93149
H	-3.32275	2.70684	1.31532	H	5.55064	0.76182	1.0189
H	-3.9646	3.83963	0.11378	H	5.68385	2.47231	0.56639
H	-4.29329	0.89786	2.03528	H	3.67233	-0.1936	-2.23784
H	-3.89801	-0.70906	2.64615	H	2.98365	-1.82509	-2.25262
H	-2.60317	0.43688	2.27856	H	1.92778	-0.41897	-2.12082
H	3.36586	-1.72244	0.73738	H	-4.20189	-0.24412	1.99674
H	3.8002	-1.93454	-0.96693	H	-3.47464	1.3436	2.24172
H	0.85848	1.48718	0.86505	H	-0.88622	1.92124	-0.83428

H	7.05651	0.20788	-0.69854	H	-4.64398	-0.57051	-2.88148
H	6.04803	-1.24131	-0.84029	H	-5.02506	1.07453	-2.33089
H	5.80513	0.12312	-1.95399	H	-3.33812	0.5919	-2.57103
H	4.55046	2.32435	0.46208	H	-2.47043	-1.0437	-1.08567
H	4.65476	0.52559	2.19308	H	-6.38521	-0.09938	-0.3973
H	5.35329	-1.00477	1.63231	H	-5.85562	-1.66325	-1.04894
H	6.3744	0.43729	1.76064	H	-5.54771	-1.26612	0.64739
atom		Con f. 3-2o		atom		Con f. 3-2p	
C	-5.66425	-1.58722	-0.53936	C	6.0126	-0.94713	-0.67953
C	-3.77611	1.92219	1.57677	C	3.52392	2.76115	-0.22993
C	-3.73533	1.55531	0.29023	C	3.71042	1.73986	0.61494
C	-2.73849	0.53786	-0.24862	C	2.90033	0.45163	0.55008
C	-3.22242	-0.9557	-0.11261	C	3.48697	-0.61192	-0.4553
C	-4.47525	-1.13621	-0.94219	C	4.87201	-1.00582	0.0097
C	-1.32578	0.73473	0.32843	C	1.40431	0.70951	0.2928
C	-0.29306	-0.19919	-0.33039	C	0.57803	-0.57708	0.40644
C	-0.73265	-1.65901	-0.17531	C	1.11415	-1.6342	-0.57684
C	-2.1343	-1.86217	-0.75539	C	2.60595	-1.88847	-0.35182
C	-4.67239	2.16642	-0.71951	C	4.73434	1.82399	1.71719
C	-3.44971	-1.36202	1.34784	C	3.5018	-0.09991	-1.90015
C	1.10479	0.09678	0.1661	C	-0.91363	-0.37402	0.24867
C	1.7996	1.27152	-0.35309	C	-1.81522	-1.39127	0.77755
C	3.07774	1.65125	0.36636	C	-3.28326	-1.23294	0.42991
C	3.86883	0.4425	0.85044	C	-3.66623	0.23351	0.3178
O	2.98549	-0.47007	1.57458	O	-2.76345	0.91284	-0.60072
C	1.73089	-0.64049	1.11522	C	-1.45466	0.65013	-0.45844
O	1.33846	1.98336	-1.25235	O	-1.418	-2.37612	1.41272
C	4.63354	-0.33038	-0.25276	C	-5.09483	0.5084	-0.17512
C	5.19568	-1.64224	0.29977	C	-5.3351	2.01531	-0.31573
O	3.77765	-0.60218	-1.37903	O	-5.18994	-0.12322	-1.46433
C	5.76133	0.5445	-0.80122	C	-6.11379	-0.10642	0.78925
H	-2.65814	0.7153	-1.33024	H	2.97269	-0.01852	1.54072
H	-0.29007	0.03348	-1.40423	H	0.72622	-0.97884	1.41843
H	4.5959	0.7481	1.60512	H	-3.5325	0.72192	1.29164
H	-6.48869	-1.68986	-1.24039	H	6.94895	-1.27898	-0.23816
H	-5.86856	-1.86833	0.48946	H	6.06581	-0.57092	-1.69676
H	-4.49746	2.65545	1.92942	H	4.11742	3.66911	-0.15339
H	-3.1005	1.51319	2.32136	H	2.78075	2.73607	-1.02067
H	-4.35024	-0.87476	-1.99445	H	4.9019	-1.3972	1.02797
H	-1.31588	0.56055	1.40967	H	1.27408	1.15184	-0.69979
H	-1.02726	1.77666	0.17529	H	1.0416	1.45035	1.01419
H	-0.02201	-2.32041	-0.68534	H	0.56112	-2.57076	-0.45139
H	-0.7253	-1.94789	0.88196	H	0.92889	-1.2911	-1.60192
H	-2.10063	-1.65414	-1.833	H	2.73863	-2.32374	0.64771
H	-2.44179	-2.90875	-0.64646	H	2.97721	-2.63077	-1.06826
H	-5.27474	1.39871	-1.21724	H	4.25404	1.71507	2.69848
H	-5.35143	2.88874	-0.2568	H	5.46931	1.01573	1.63718
H	-4.10681	2.68049	-1.50777	H	5.27011	2.77775	1.70072
H	-4.25821	-0.78481	1.80174	H	4.16262	0.76258	-2.01218
H	-3.71232	-2.42314	1.40883	H	3.85119	-0.88661	-2.57665
H	-2.55478	-1.20898	1.95454	H	2.50648	0.20129	-2.23331
H	3.68415	2.29354	-0.27313	H	-3.46183	-1.7438	-0.52465
H	2.77743	2.24855	1.23739	H	-3.88038	-1.73208	1.19478
H	1.25459	-1.47682	1.61733	H	-0.85528	1.37658	-0.99821
H	5.77111	-2.14908	-0.47987	H	-6.35579	2.19146	-0.67059
H	5.85432	-1.45893	1.15449	H	-5.21863	2.52352	0.64689
H	4.39145	-2.30667	0.62714	H	-4.63898	2.45741	-1.03147
H	3.14321	-1.28686	-1.11937	H	-6.10286	-0.01448	-1.76976
H	5.37074	1.435	-1.3004	H	-6.03553	-1.19612	0.80873
H	6.43601	0.85916	-0.00028	H	-5.97917	0.2741	1.80666
H	6.33718	-0.02698	-1.53469	H	-7.12569	0.15526	0.46289
atom		Con f. 3-2q		atom		Con f. 3-2r	
C	-6.00606	-1.17064	0.08614	C	-6.01396	-0.95608	0.63996
C	-3.31661	1.95318	1.90387	C	-3.51722	2.7584	0.31042
C	-3.58822	1.66828	0.62461	C	-3.70093	1.7617	-0.56392
C	-2.85106	0.58951	-0.1579	C	-2.89312	0.471	-0.53199
C	-3.46923	-0.84999	0.00836	C	-3.48642	-0.6209	0.43843
C	-4.87821	-0.83888	-0.54413	C	-4.86986	-0.99875	-0.04462
C	-1.33678	0.59458	0.11758	C	-1.39802	0.71847	-0.25998
C	-0.57783	-0.39226	-0.79129	C	-0.57454	-0.5659	-0.41096
C	-1.14497	-1.80429	-0.612	C	-1.11554	-1.65082	0.53885
C	-2.65001	-1.81879	-0.8906	C	-2.60696	-1.89542	0.30135
C	-4.63593	2.44116	-0.13459	C	-4.71888	1.87885	-1.66878
C	-3.43922	-1.33079	1.4636	C	-3.50787	-0.15214	1.89783
C	0.91503	-0.27547	-0.57651	C	0.91764	-0.37319	-0.24545

C	1.62375	0.84069	-1.19829	C	1.81407	-1.38977	-0.79171
C	3.13563	0.81652	-1.0735	C	3.27771	-1.25111	-0.41853
C	3.54613	0.20974	0.25681	C	3.65744	0.21498	-0.30806
O	2.94849	-1.103	0.4139	O	2.77371	0.89491	0.62033
C	1.64227	-1.19426	0.10787	C	1.46106	0.63835	0.4773
O	1.05236	1.71116	-1.86539	O	1.41105	-2.35469	-1.45161
C	5.06025	0.04203	0.48418	C	5.09906	0.50766	0.14763
C	5.71543	-0.8473	-0.57374	C	5.41537	-0.09948	1.51519
O	5.55734	1.38826	0.38197	O	5.89517	-0.11876	-0.87406
C	5.32126	-0.49742	1.89244	C	5.35315	2.01716	0.14655
H	-2.9727	0.83255	-1.22281	H	-2.96078	0.02998	-1.53623
H	-0.76926	-0.08521	-1.82874	H	-0.72225	-0.93639	-1.43492
H	3.14382	0.83073	1.06874	H	3.5045	0.69441	-1.28466
H	-6.96327	-1.13514	-0.42783	H	-6.94873	-1.2734	0.18478
H	-6.02823	-1.48564	1.12517	H	-6.07158	-0.60743	1.66671
H	-3.8576	2.73448	2.43234	H	-4.10891	3.66917	0.25645
H	-2.55322	1.42816	2.46934	H	-2.77808	2.71022	1.10393
H	-4.94053	-0.53177	-1.58957	H	-4.89507	-1.36214	-1.07333
H	-1.12798	0.33944	1.16197	H	-1.27086	1.1288	0.74679
H	-0.95522	1.60754	-0.04137	H	-1.03099	1.48087	-0.95628
H	-0.6391	-2.50311	-1.28877	H	-0.56428	-2.58448	0.38697
H	-0.95486	-2.16019	0.40715	H	-0.93262	-1.3393	1.57436
H	-2.81501	-1.54313	-1.9405	H	-2.73663	-2.30146	-0.71076
H	-3.04768	-2.83268	-0.76577	H	-2.98212	-2.65742	0.9947
H	-5.1155	3.1978	0.49351	H	-5.45512	1.06966	-1.61563
H	-4.18924	2.94578	-1.00144	H	-5.25336	2.83258	-1.62798
H	-5.4134	1.77643	-0.52636	H	-4.23338	1.79714	-2.65015
H	-2.42654	-1.32386	1.87205	H	-2.51418	0.1391	2.24437
H	-4.058	-0.69982	2.10535	H	-4.16969	0.70627	2.03262
H	-3.81449	-2.35709	1.53118	H	-3.86012	-0.95889	2.54875
H	3.52159	0.22106	-1.91096	H	3.41688	-1.76582	0.54108
H	3.53125	1.82866	-1.16898	H	3.89963	-1.75047	-1.16279
H	1.22245	-2.12221	0.4818	H	0.86575	1.36051	1.0266
H	6.78203	-0.95784	-0.3517	H	6.43732	0.16377	1.80781
H	5.26564	-1.84413	-0.58396	H	4.73406	0.28099	2.28154
H	5.62089	-0.4107	-1.57146	H	5.33977	-1.18969	1.49027
H	6.51354	1.35177	0.53337	H	6.82441	0.03197	-0.64553
H	4.86032	0.15405	2.64165	H	5.13246	2.44119	-0.83815
H	4.92152	-1.5076	2.01462	H	4.73714	2.52624	0.89239
H	6.39951	-0.53325	2.08078	H	6.40433	2.21431	0.38197
atom	Con f. 3-2s			atom	Con f. 3-2t		
C	5.47773	-1.65715	-0.80053	C	-5.47669	-2.06068	-0.53164
C	3.66146	2.43773	-0.8511	C	-4.71093	2.01789	-1.40449
C	3.81305	1.57077	0.15715	C	-3.74597	1.76806	-0.51082
C	2.81781	0.45519	0.44703	C	-2.94988	0.47047	-0.58111
C	3.08369	-0.85978	-0.38104	C	-3.48895	-0.69836	0.33229
C	4.4428	-1.4048	0.00226	C	-4.94295	-0.95675	-0.00606
C	1.35752	0.9253	0.31393	C	-1.44828	0.7206	-0.35185
C	0.36848	-0.15041	0.77609	C	-0.59695	-0.54472	-0.55873
C	0.58707	-1.44275	-0.03384	C	-1.12796	-1.71558	0.28772
C	2.03788	-1.91712	0.07131	C	-2.62028	-1.94064	0.04222
C	4.98347	1.67729	1.10043	C	-3.39759	2.78811	0.54595
C	2.97595	-0.62956	-1.89276	C	-3.43306	-0.35381	1.83651
C	-1.08187	0.28056	0.73669	C	0.87609	-0.28765	-0.31885
C	-2.04465	-0.42236	1.57234	C	1.38281	0.1686	0.9718
C	-3.43387	0.1807	1.63672	C	2.85504	0.54876	1.00666
C	-3.88058	0.84302	0.33911	C	3.66528	-0.32075	0.06339
O	-2.81419	1.67375	-0.21678	O	3.08934	-0.27284	-1.27529
C	-1.53578	1.28857	-0.04842	C	1.75598	-0.39758	-1.34755
O	-1.74909	-1.40051	2.26992	O	0.67669	0.32582	1.97562
C	-4.41333	-0.08065	-0.78356	C	5.15464	0.02761	-0.10313
C	-5.71185	-0.74344	-0.32808	C	5.89262	-0.17941	1.21603
O	-4.7809	0.77137	-1.8843	O	5.7196	-0.92985	-1.01861
C	-3.41018	-1.13038	-1.27118	C	5.36589	1.44347	-0.64528
H	2.96081	0.16708	1.49768	H	-3.06523	0.09026	-1.6043
H	0.59675	-0.38695	1.82472	H	-0.70368	-0.83836	-1.61247
H	-4.67746	1.55936	0.54739	H	3.59355	-1.36989	0.37834
H	6.40438	-2.06496	-0.40475	H	-6.54584	-2.12512	-0.7188
H	5.45099	-1.47026	-1.86991	H	-4.89093	-2.93599	-0.79593
H	4.38832	3.22701	-1.02707	H	-5.29098	2.93757	-1.37754
H	2.81754	2.39939	-1.53265	H	-4.95596	1.30849	-2.19093
H	4.54917	-1.61894	1.06714	H	-5.60865	-0.12734	0.2273
H	1.15149	1.19596	-0.72654	H	-1.278	1.11066	0.65351
H	1.22313	1.83697	0.90681	H	-1.10794	1.49506	-1.04962
H	-0.08128	-2.22738	0.33423	H	-0.57104	-2.6253	0.03418

H	0.31348	-1.25723	-1.07943	H	-0.94035	-1.51825	1.34654
H	2.24212	-2.18358	1.11685	H	-2.76305	-2.23223	-1.00622
H	2.18289	-2.82983	-0.51856	H	-2.97561	-2.77835	0.65365
H	5.56731	0.7507	1.11461	H	-3.39451	2.36049	1.55272
H	5.65114	2.49985	0.82712	H	-2.39399	3.19803	0.38112
H	4.63515	1.84362	2.12838	H	-4.10515	3.62194	0.53052
H	2.00246	-0.22164	-2.17211	H	-4.16584	0.415	2.09613
H	3.73991	0.06504	-2.24874	H	-3.67035	-1.2447	2.42858
H	3.10095	-1.57651	-2.42774	H	-2.44769	0.00089	2.14783
H	-4.14923	-0.57976	1.9537	H	2.9271	1.60625	0.72172
H	-3.40104	0.9411	2.42798	H	3.22086	0.451	2.03043
H	-0.87645	1.90752	-0.64813	H	1.43157	-0.60449	-2.36447
H	-6.17686	-1.24019	-1.18467	H	5.71007	-1.18352	1.6115
H	-5.52831	-1.49643	0.44161	H	6.96753	-0.06517	1.04815
H	-6.4151	-0.00189	0.06386	H	5.58342	0.55475	1.96362
H	-3.98778	1.27713	-2.12094	H	5.21842	-0.86379	-1.84618
H	-2.50931	-0.66213	-1.67609	H	4.83229	1.58423	-1.59009
H	-3.11524	-1.81668	-0.47225	H	6.43306	1.60434	-0.82254
H	-3.87541	-1.71767	-2.06856	H	5.01867	2.20269	0.06109

atom	Con f. 3- 3a			atom	Con f. 3- 3b		
C	-4.46043	2.51724	-0.57342	C	-3.07782	3.22348	0.40408
C	-3.77315	-0.77895	2.34249	C	-2.72962	-0.17818	2.93334
C	-2.90846	0.11832	1.85392	C	-2.96316	-0.75972	1.75039
C	-2.17941	-0.07063	0.53107	C	-2.24899	-0.26632	0.49827
C	-2.99966	0.42144	-0.7207	C	-3.05095	0.78065	-0.3686
C	-3.27749	1.90094	-0.56679	C	-3.44992	1.94695	0.51297
C	-1.67631	-1.51006	0.33957	C	-1.74853	-1.44037	-0.35964
C	-0.75155	-1.66651	-0.89025	C	-0.82847	-0.99834	-1.51968
C	-1.4688	-1.1255	-2.13984	C	-1.53416	0.09171	-2.34481
C	-2.07537	0.27351	-1.96137	C	-2.11295	1.23812	-1.50457
C	-2.57781	1.37321	2.62012	C	-3.89287	-1.94416	1.64906
C	-4.29302	-0.3757	-0.92478	C	-4.35848	0.20647	-0.95487
C	0.62431	-1.08385	-0.61613	C	0.56034	-0.64815	-1.01449
C	1.48587	-1.77361	0.34249	C	1.41688	-1.74044	-0.55673
C	2.89304	-1.231	0.50409	C	2.84282	-1.3764	-0.19022
C	2.94593	0.26297	0.24771	C	2.93358	0.03747	0.35145
O	2.33922	0.55314	-1.04474	O	2.30406	0.95787	-0.58652
C	1.16258	-0.04313	-1.30127	C	1.11042	0.59059	-1.0827
O	1.1435	-2.80524	0.93332	O	1.05205	-2.92254	-0.55087
C	4.35945	0.87302	0.19625	C	4.36352	0.57086	0.565
C	4.28092	2.37701	-0.08669	C	4.32135	2.00447	1.10418
O	5.11943	0.2192	-0.83418	O	5.07124	0.53792	-0.68588
C	5.10385	0.61272	1.50336	C	5.13791	-0.33836	1.51552
H	-1.29092	0.57489	0.5655	H	-1.36564	0.28473	0.84455
H	-0.5964	-2.74324	-1.0322	H	-0.6951	-1.87505	-2.16528
H	2.34347	0.79245	0.99717	H	2.36829	0.11664	1.28884
H	-4.52717	3.59688	-0.46546	H	-3.45086	3.97064	1.10037
H	-5.40084	1.98537	-0.68224	H	-2.40165	3.58287	-0.36579
H	-4.2746	-0.61653	3.29359	H	-3.22208	-0.51493	3.84272
H	-4.01101	-1.70182	1.82297	H	-2.04051	0.65707	3.03088
H	-2.37849	2.50905	-0.45157	H	-4.13541	1.68091	1.31601
H	-1.14762	-1.82783	1.24086	H	-1.215	-2.15084	0.27741
H	-2.5226	-2.19357	0.22013	H	-2.59801	-1.98473	-0.78464
H	-0.79014	-1.12413	-3.001	H	-0.85205	0.49811	-3.10114
H	-2.26987	-1.8305	-2.38836	H	-2.34788	-0.3904	-2.89819
H	-1.28107	1.02205	-1.87542	H	-1.30036	1.81717	-1.05704
H	-2.64785	0.53789	-2.85831	H	-2.65557	1.92707	-2.1625
H	-1.50477	1.4115	2.85007	H	-3.34469	-2.85105	1.36738
H	-2.80331	2.2686	2.03073	H	-4.38553	-2.13566	2.60656
H	-3.13331	1.43269	3.56078	H	-4.66722	-1.80303	0.88924
H	-4.97609	-0.25544	-0.08059	H	-5.09541	0.01088	-0.17129
H	-4.80707	-0.03457	-1.82907	H	-4.80083	0.93257	-1.64572
H	-4.09729	-1.44349	-1.04282	H	-4.19993	-0.72372	-1.50625
H	3.24233	-1.46597	1.51103	H	3.20987	-2.09803	0.54159
H	3.54566	-1.75197	-0.20855	H	3.46197	-1.46671	-1.09224
H	0.68664	0.41138	-2.16219	H	0.63083	1.42831	-1.57534
H	3.73812	2.89648	0.70951	H	3.81876	2.04082	2.07605
H	3.77193	2.57594	-1.03369	H	3.78801	2.6687	0.41878
H	5.29207	2.78992	-0.1437	H	5.34185	2.37831	1.22636
H	4.64412	0.3566	-1.66852	H	4.57389	1.09208	-1.30755
H	4.53427	0.99225	2.3565	H	6.1198	0.0983	1.71957
H	5.28721	-0.45447	1.65073	H	4.6065	-0.45004	2.465
H	6.07043	1.12414	1.47999	H	5.28977	-1.32908	1.08013

atom	Con f. 3- 3c			atom	Con f. 3- 3d		
C	-4.45548	2.41389	-0.89369	C	-4.61007	-0.62455	2.43078
C	-3.78457	-0.44085	2.41092	C	-4.16047	-1.25025	-2.02317
C	-2.90414	0.37149	1.8137	C	-3.32378	-1.51948	-1.01404
C	-2.17347	0.00169	0.53018	C	-2.37451	-0.48723	-0.41804
C	-2.99094	0.31949	-0.77819	C	-3.02966	0.40451	0.70172
C	-3.27016	1.80614	-0.82364	C	-3.41039	-0.48708	1.86397
C	-1.67256	-1.45147	0.53851	C	-1.68619	0.36216	-1.50931
C	-0.74907	-1.77772	-0.65866	C	-0.67082	1.40123	-0.99958
C	-1.46404	-1.40867	-1.97074	C	-1.30636	2.23794	0.13532
C	-2.06291	0.00522	-1.98423	C	-1.92288	1.36104	1.22336
C	-2.55538	1.71001	2.41116	C	-3.25389	-2.89976	-0.41322
C	-4.28356	-0.49776	-0.87666	C	-4.23183	1.20365	0.18713
C	0.63011	-1.16926	-0.46941	C	0.69268	0.85428	-0.61066
C	1.48555	-1.72057	0.58115	C	1.77253	1.81214	-0.37744
C	2.90263	-1.17937	0.65403	C	3.05788	1.24841	0.19482
C	2.94945	0.26381	0.19249	C	3.35617	-0.12937	-0.36965
O	2.35901	0.36803	-1.13492	O	2.19362	-0.99373	-0.21536
C	1.17564	-0.24617	-1.30279	C	0.98399	-0.4628	-0.46954
O	1.13305	-2.64901	1.31801	O	1.64996	3.02631	-0.57668
C	4.33318	0.92836	0.09679	C	4.52312	-0.87402	0.30894
C	4.96237	1.03383	1.48301	C	4.72319	-2.24961	-0.33537
O	4.1275	2.28538	-0.33955	O	4.23542	-1.02601	1.70885
C	5.2609	0.20741	-0.88435	C	5.80562	-0.05058	0.22491
H	-1.28342	0.64392	0.47751	H	-1.60062	-1.05451	0.11012
H	-0.59842	-2.86452	-0.65207	H	-0.47204	2.09154	-1.82901
H	2.31913	0.87897	0.84832	H	3.54575	-0.05897	-1.4483
H	-4.52521	3.49813	-0.92934	H	-4.75327	-1.28767	3.28015
H	-5.395	1.86969	-0.91607	H	-5.4899	-0.09324	2.08026
H	-4.28548	-0.14744	3.3305	H	-4.82192	-2.0152	-2.42277
H	-4.03648	-1.41982	2.01538	H	-4.22105	-0.27098	-2.48684
H	-2.37185	2.42569	-0.80268	H	-2.57507	-1.05607	2.27626
H	-1.14368	-1.64223	1.47468	H	-1.20526	-0.31152	-2.2278
H	-2.51989	-2.14384	0.51525	H	-2.45105	0.90688	-2.07047
H	-0.78589	-1.52867	-2.82387	H	-0.55801	2.90168	0.57561
H	-2.26829	-2.13681	-2.12129	H	-2.07421	2.88592	-0.30185
H	-1.26365	0.75365	-1.99508	H	-1.13144	0.7571	1.68682
H	-2.63009	0.15009	-2.91136	H	-2.34429	1.98844	2.01755
H	-1.48297	1.76015	2.64164	H	-2.24435	-3.31769	-0.52294
H	-2.76212	2.52379	1.7077	H	-3.46577	-2.8727	0.66113
H	-3.11385	1.90243	3.33211	H	-3.96282	-3.58572	-0.88626
H	-4.96311	-0.27256	-0.05164	H	-5.05103	0.54409	-0.10747
H	-4.80133	-0.2723	-1.81463	H	-4.60099	1.8787	0.96619
H	-4.08816	-1.57191	-0.86119	H	-3.97341	1.81272	-0.68137
H	3.26549	-1.27362	1.67945	H	3.87245	1.94047	-0.02401
H	3.52996	-1.81455	0.01566	H	2.94795	1.18361	1.28524
H	0.7031	0.08114	-2.22139	H	0.24474	-1.24947	-0.56315
H	5.22957	0.04947	1.87427	H	4.98376	-2.15073	-1.39401
H	4.27835	1.51911	2.18635	H	3.81665	-2.85627	-0.26143
H	5.87414	1.63485	1.41884	H	5.53548	-2.77575	0.17409
H	3.66232	2.24391	-1.18927	H	3.41367	-1.5371	1.77515
H	5.50334	-0.80296	-0.54302	H	6.63717	-0.62725	0.64007
H	4.80153	0.13395	-1.87465	H	6.04163	0.19471	-0.81454
H	6.19419	0.77021	-0.97586	H	5.71912	0.87867	0.79346
atom	Con f. 3- 3e			atom	Con f. 3- 3f		
C	3.04643	0.44015	3.17492	C	-4.44879	2.51171	-0.63417
C	3.28086	2.88302	-0.33048	C	-3.77748	-0.69907	2.36335
C	3.32461	1.65675	-0.86595	C	-2.90469	0.17625	1.84998
C	2.41154	0.55784	-0.33649	C	-2.17904	-0.05473	0.532
C	3.0529	-0.38373	0.75231	C	-2.99804	0.40616	-0.7323
C	3.50444	0.46745	1.92227	C	-3.26841	1.89065	-0.61881
C	1.78023	-0.24289	-1.49783	C	-1.68364	-1.50158	0.38058
C	0.77302	-1.3273	-1.0774	C	-0.76096	-1.69685	-0.84513
C	1.4067	-2.21277	0.02028	C	-1.47922	-1.19	-2.10852
C	1.96033	-1.38602	1.17914	C	-2.07534	0.21804	-1.96892
C	4.23786	1.36229	-2.03131	C	-2.56055	1.44788	2.58163
C	4.30039	-1.13649	0.24243	C	-4.29561	-0.38969	-0.91323
C	-0.6051	-0.81884	-0.68567	C	0.61762	-1.10993	-0.59302
C	-1.6716	-1.79721	-0.48085	C	1.4758	-1.76274	0.39256
C	-2.96446	-1.26464	0.10507	C	2.88581	-1.21996	0.5311
C	-3.28709	0.11786	-0.43478	C	2.94257	0.26123	0.20296
O	-2.13853	0.99851	-0.27001	O	2.34634	0.48894	-1.10466
C	-0.91973	0.49098	-0.52547	C	1.1644	-0.10661	-1.32746
O	-1.53433	-3.00386	-0.71372	O	1.13126	-2.76901	1.02538
C	-4.46273	0.83311	0.2604	C	4.34843	0.87722	0.17114

C	-4.68701	2.21498	-0.36245	C	4.28535	2.34382	-0.26812
O	-4.17072	0.96869	1.66102	O	5.0873	0.10989	-0.79593
C	-5.73251	-0.00889	0.16971	C	5.00518	0.76124	1.55006
H	1.61163	1.06711	0.20873	H	-1.28673	0.58627	0.54663
H	0.60473	-1.97127	-1.95008	H	-0.6097	-2.77798	-0.95557
H	-3.47996	0.06286	-1.51373	H	2.32914	0.82117	0.92087
H	3.46158	1.10029	3.93261	H	-4.51027	3.59419	-0.5555
H	2.25378	-0.2262	3.50203	H	-5.39216	1.98116	-0.72201
H	3.91924	3.68669	-0.6902	H	-4.2761	-0.50608	3.31026
H	2.60758	3.12318	0.48856	H	-4.02487	-1.63322	1.86901
H	4.30209	1.16869	1.68199	H	-2.36615	2.49777	-0.52603
H	1.30733	0.45797	-2.19641	H	-1.15557	-1.79655	1.28985
H	2.57691	-0.7396	-2.05992	H	-2.5337	-2.18373	0.28127
H	0.67266	-2.9306	0.39382	H	-0.80405	-1.21888	-2.97189
H	2.21071	-2.80031	-0.43714	H	-2.28561	-1.89674	-2.33312
H	1.13461	-0.83305	1.64439	H	-1.27491	0.96224	-1.90354
H	2.36515	-2.05333	1.94889	H	-2.64617	0.46203	-2.87269
H	3.66174	1.16487	-2.94317	H	-1.48732	1.48022	2.81163
H	4.89782	2.21095	-2.23215	H	-2.7752	2.32896	1.96717
H	4.85942	0.47883	-1.86056	H	-3.11603	1.53958	3.51969
H	5.14097	-0.45358	0.0917	H	-4.97773	-0.24172	-0.07278
H	4.61167	-1.88109	0.98334	H	-4.80834	-0.0718	-1.82673
H	4.11942	-1.659	-0.7002	H	-4.10567	-1.46145	-1.0005
H	-3.76893	-1.96547	-0.123	H	3.23033	-1.40714	1.54959
H	-2.85132	-1.2163	1.19598	H	3.53579	-1.78156	-0.15148
H	-0.19335	1.29122	-0.60459	H	0.68966	0.30864	-2.20873
H	-4.95136	2.12781	-1.42119	H	3.68381	2.93726	0.42796
H	-3.78937	2.83418	-0.28393	H	3.85492	2.4343	-1.26754
H	-5.50459	2.72125	0.15855	H	5.29637	2.76383	-0.28589
H	-3.35695	1.49196	1.73121	H	5.99644	0.44441	-0.79366
H	-6.57127	0.54898	0.59589	H	5.97955	1.26043	1.53094
H	-5.96877	-0.24357	-0.87212	H	4.3959	1.24115	2.32237
H	-5.62896	-0.94425	0.72518	H	5.16494	-0.28339	1.82798
atom		Con f. 3- 3g		atom		Con f. 3- 3h	
C	-4.35421	2.69571	0.39946	C	-3.14981	3.14893	-0.17698
C	-2.82144	0.17877	2.85173	C	-3.85689	-1.38882	2.06535
C	-2.95133	-0.57332	1.75254	C	-2.94253	-0.43354	1.85854
C	-2.18275	-0.22461	0.48485	C	-2.2454	-0.22447	0.52241
C	-2.96464	0.73835	-0.48698	C	-3.07776	0.61206	-0.52247
C	-3.19964	2.05154	0.22724	C	-3.53019	1.90589	0.12371
C	-1.67606	-1.47913	-0.24449	C	-1.74688	-1.54115	-0.09355
C	-0.74099	-1.16304	-1.43402	C	-0.83586	-1.31448	-1.32401
C	-1.4432	-0.17681	-2.38287	C	-1.55065	-0.40169	-2.33817
C	-2.01942	1.05872	-1.67866	C	-2.15131	0.87327	-1.72718
C	-3.83907	-1.79422	1.76022	C	-2.50853	0.48812	2.96856
C	-4.27771	0.1341	-0.99954	C	-4.35732	-0.11915	-0.97754
C	0.64121	-0.74782	-0.95918	C	0.54864	-0.85881	-0.89626
C	1.48343	-1.76401	-0.32896	C	1.41274	-1.82622	-0.22286
C	2.90765	-1.35326	0.00138	C	2.8293	-1.3775	0.08083
C	2.98053	0.12647	0.32278	C	2.90306	0.11906	0.31715
O	2.39387	0.89186	-0.76875	O	2.27612	0.8179	-0.79701
C	1.20342	0.46093	-1.21793	C	1.08885	0.34529	-1.21309
O	1.11439	-2.93176	-0.15699	O	1.06307	-2.98919	0.01331
C	4.37836	0.72391	0.55887	C	4.32723	0.69691	0.42606
C	5.00246	0.11488	1.81077	C	4.27354	2.21317	0.64053
O	4.20323	2.12288	0.85163	O	5.0498	0.39833	-0.78016
C	5.29429	0.5633	-0.65704	C	5.09422	0.02199	1.56037
H	-1.30045	0.34785	0.79926	H	-1.35747	0.38873	0.72559
H	-0.59713	-2.10585	-1.97641	H	-0.69328	-2.29431	-1.79621
H	2.36259	0.34189	1.20446	H	2.3273	0.38639	1.21258
H	-4.38819	3.6517	0.91598	H	-3.55986	4.00107	0.35926
H	-5.30379	2.30778	0.04247	H	-2.42857	3.37442	-0.95699
H	-3.37073	-0.04388	3.76366	H	-4.33159	-1.51307	3.03582
H	-2.16122	1.04172	2.8789	H	-4.1583	-2.08307	1.28743
H	-2.28932	2.51041	0.6167	H	-4.26793	1.77186	0.91504
H	-1.15473	-2.12491	0.4663	H	-1.20562	-2.11028	0.66572
H	-2.52211	-2.05907	-0.62684	H	-2.59505	-2.16195	-0.39865
H	-0.76256	0.13862	-3.18247	H	-0.86846	-0.13241	-3.15304
H	-2.25893	-0.7181	-2.87513	H	-2.35273	-0.98773	-2.79964
H	-1.20792	1.68894	-1.30055	H	-1.34982	1.54136	-1.39891
H	-2.56862	1.66925	-2.40505	H	-2.7082	1.41531	-2.50071
H	-4.39492	-1.86583	2.69947	H	-1.4489	0.32344	3.20636
H	-4.5591	-1.79122	0.93647	H	-2.59663	1.53842	2.66823
H	-3.24745	-2.71109	1.65079	H	-3.09218	0.33579	3.88127
H	-5.00651	0.01509	-0.19434	H	-5.04277	-0.27378	-0.14021

H	-4.72443	0.7866	-1.75696	H	-4.87686	0.48365	-1.73018
H	-4.12318	-0.84416	-1.45952	H	-4.14632	-1.09477	-1.42117
H	3.26394	-1.95221	0.84171	H	3.18839	-1.92709	0.95272
H	3.5288	-1.59465	-0.87075	H	3.46425	-1.64914	-0.77264
H	0.73954	1.21062	-1.84803	H	0.60609	1.06019	-1.86891
H	5.23632	-0.94197	1.66337	H	5.29155	2.60966	0.69186
H	4.32886	0.21027	2.66825	H	3.75847	2.458	1.57502
H	5.93244	0.64215	2.0428	H	3.74864	2.71041	-0.17952
H	3.74194	2.51526	0.09434	H	4.55754	0.80156	-1.51223
H	5.51418	-0.48859	-0.86021	H	6.07098	0.50058	1.67579
H	4.83626	0.99709	-1.55108	H	4.55119	0.11579	2.50521
H	6.23917	1.07976	-0.46585	H	5.25752	-1.03848	1.3535

atom	Con f. 3- 3i			atom	Con f. 3- 3j		
C	-4.43326	2.42125	-0.9093	C	4.92424	-1.65196	1.34346
C	-3.76976	-0.40497	2.41956	C	3.9637	2.30659	-0.40582
C	-2.88539	0.39423	1.81058	C	3.26852	1.5825	0.47978
C	-2.16372	0.0061	0.52734	C	2.29822	0.48308	0.07011
C	-2.98505	0.31627	-0.78049	C	2.983	-0.91822	-0.15161
C	-3.25252	1.80458	-0.84005	C	3.64172	-1.34575	1.14196
C	-1.67298	-1.45037	0.54836	C	1.43826	0.88144	-1.13923
C	-0.75616	-1.79416	-0.64902	C	0.30263	-0.12747	-1.42591
C	-1.47602	-1.43599	-1.96138	C	0.89211	-1.54254	-1.56296
C	-2.06595	-0.01845	-1.98796	C	1.84896	-1.94326	-0.43152
C	-2.52214	1.73518	2.39379	C	3.38324	1.84758	1.95864
C	-4.28461	-0.49149	-0.86371	C	3.98361	-0.90374	-1.31258
C	0.62542	-1.18795	-0.47109	C	-0.83837	0.01934	-0.43391
C	1.48195	-1.72763	0.58593	C	-1.61266	1.26062	-0.456
C	2.89724	-1.18398	0.6458	C	-2.54711	1.4688	0.71756
C	2.92966	0.25564	0.17038	C	-3.2508	0.18645	1.14226
O	2.3546	0.34293	-1.15786	O	-2.27248	-0.88706	1.29489
C	1.17252	-0.27811	-1.31812	C	-1.22049	-0.93252	0.45229
O	1.12974	-2.6488	1.33212	O	-1.46828	2.13354	-1.31848
C	4.31568	0.92245	0.10902	C	-4.39012	-0.28814	0.20768
C	5.29376	0.16198	-0.78781	C	-4.93283	-1.64219	0.67178
O	4.75482	0.87991	1.47841	O	-3.92288	-0.38868	-1.15053
C	4.17608	2.37807	-0.34308	C	-5.50841	0.75343	0.1803
H	-1.26947	0.64139	0.46399	H	1.6106	0.33818	0.91503
H	-0.61002	-2.88147	-0.63118	H	-0.12539	0.14865	-2.39778
H	2.28792	0.86095	0.82546	H	-3.66889	0.30429	2.14402
H	-4.49446	3.50562	-0.95555	H	5.27751	-1.95758	2.32504
H	-5.37722	1.88445	-0.92073	H	5.66919	-1.60987	0.55438
H	-4.26361	-0.09888	3.33883	H	4.64367	3.0905	-0.08072
H	-4.03186	-1.38537	2.03433	H	3.88101	2.15383	-1.47728
H	-2.34908	2.41693	-0.83057	H	2.95475	-1.41053	1.98759
H	-1.14194	-1.63538	1.48441	H	1.01584	1.87405	-0.96657
H	-2.52508	-2.13716	0.53493	H	2.05985	0.96315	-2.036
H	-0.80358	-1.57018	-2.81691	H	0.08656	-2.28272	-1.63979
H	-2.28571	-2.16076	-2.09881	H	1.43409	-1.58218	-2.51424
H	-1.26165	0.7242	-2.01265	H	1.29381	-2.08726	0.50095
H	-2.63763	0.11926	-2.91344	H	2.3036	-2.91271	-0.66747
H	-1.44828	1.7779	2.61904	H	4.09302	2.65204	2.17342
H	-2.72483	2.54408	1.68351	H	2.407	2.12928	2.37506
H	-3.07459	1.94146	3.3154	H	3.70623	0.95081	2.49831
H	-4.9575	-0.2531	-0.03694	H	4.80539	-0.209	-1.12411
H	-4.80594	-0.27095	-1.80088	H	4.40896	-1.90224	-1.45594
H	-4.09749	-1.56697	-0.83904	H	3.51066	-0.61163	-2.25242
H	3.27801	-1.26087	1.66541	H	-1.92713	1.8219	1.55198
H	3.51854	-1.82208	0.00433	H	-3.26262	2.25806	0.4857
H	0.70177	0.03411	-2.24286	H	-0.69985	-1.87572	0.569
H	4.90591	0.07387	-1.80674	H	-5.32399	-1.57953	1.69209
H	5.48758	-0.84136	-0.39948	H	-4.15106	-2.40643	0.65363
H	6.24701	0.69908	-0.83206	H	-5.74345	-1.95684	0.00854
H	5.6276	1.2988	1.51199	H	-3.32933	-1.15221	-1.21009
H	3.81468	2.44343	-1.37256	H	-5.85889	0.97804	1.19162
H	3.47865	2.9153	0.30744	H	-5.17588	1.68224	-0.28968
H	5.15037	2.87546	-0.29242	H	-6.35063	0.36473	-0.39903

atom	Con f. 3- 3k			atom	Con f. 3- 3l		
C	4.90681	-1.66888	1.38483	C	-4.64017	-0.60723	2.40811
C	3.99601	2.30167	-0.36834	C	-4.14214	-1.26802	-2.03406
C	3.28263	1.57978	0.50448	C	-3.31544	-1.52863	-1.01448
C	2.31302	0.48651	0.07697	C	-2.37367	-0.49066	-0.41633
C	2.99454	-0.9174	-0.13818	C	-3.0421	0.40992	0.6883
C	3.62986	-1.3524	1.16469	C	-3.43422	-0.47177	1.85428

C	1.47362	0.89317	-1.14417	C	-1.67358	0.35	-1.50704
C	0.33919	-0.11019	-1.45356	C	-0.66456	1.39389	-0.9946
C	0.9262	-1.52656	-1.58753	C	-1.31508	2.24056	0.12415
C	1.86024	-1.93597	-0.4403	C	-1.94266	1.37298	1.21337
C	3.37545	1.84088	1.98551	C	-3.24989	-2.90422	-0.40251
C	4.01433	-0.90443	-1.28239	C	-4.23986	1.20239	0.15344
C	-0.81697	0.03499	-0.47825	C	0.69402	0.8502	-0.58341
C	-1.58698	1.27411	-0.5065	C	1.76796	1.81289	-0.33599
C	-2.56164	1.47843	0.63575	C	3.04329	1.24956	0.26432
C	-3.24413	0.19612	1.09587	C	3.34237	-0.11788	-0.32187
O	-2.28225	-0.89659	1.21069	O	2.18969	-0.99321	-0.16008
C	-1.22073	-0.931	0.38325	C	0.98206	-0.46727	-0.43252
O	-1.43059	2.14993	-1.36561	O	1.64717	3.02548	-0.54202
C	-4.44346	-0.30674	0.25573	C	4.54241	-0.88122	0.26587
C	-5.59964	0.68456	0.36644	C	5.83597	-0.12464	-0.02105
O	-4.91083	-1.51667	0.87993	O	4.65153	-2.1244	-0.45205
C	-4.10659	-0.59134	-1.21039	C	4.37959	-1.16277	1.76178
H	1.61182	0.34241	0.91084	H	-1.60554	-1.05303	0.1253
H	-0.07302	0.17192	-2.4305	H	-0.45538	2.07637	-1.82795
H	-3.6132	0.31999	2.11576	H	3.49675	-0.02662	-1.40505
H	5.24294	-1.97944	2.37087	H	-4.79169	-1.26282	3.26187
H	5.66376	-1.63068	0.60702	H	-5.51719	-0.0816	2.04229
H	4.67528	3.08088	-0.03072	H	-4.79842	-2.03686	-2.43471
H	3.92992	2.15139	-1.4413	H	-4.19941	-0.29226	-2.50546
H	2.92953	-1.41403	1.99954	H	-2.60236	-1.0346	2.28172
H	1.05218	1.88672	-0.974	H	-1.18466	-0.32936	-2.21472
H	2.10952	0.97621	-2.03071	H	-2.43253	0.88994	-2.08073
H	0.12038	-2.26397	-1.68477	H	-0.57377	2.91056	0.5667
H	1.48625	-1.5622	-2.52835	H	-2.07906	2.88203	-0.32906
H	1.28876	-2.07965	0.48242	H	-1.15561	0.77458	1.6913
H	2.31392	-2.90707	-0.67111	H	-2.37404	2.00709	1.99681
H	3.68517	0.9411	2.52793	H	-2.2389	-3.32184	-0.49935
H	4.08613	2.64096	2.21322	H	-3.47207	-2.86917	0.66954
H	2.39434	2.12687	2.38727	H	-3.9535	-3.59462	-0.87705
H	4.83741	-0.21604	-1.07723	H	-5.05547	0.53883	-0.14206
H	4.43536	-1.90512	-1.42313	H	-4.61743	1.88564	0.92129
H	3.55916	-0.60498	-2.22866	H	-3.97347	1.80204	-0.71916
H	-1.97652	1.88675	1.46996	H	3.86533	1.94167	0.07359
H	-3.29832	2.23318	0.35639	H	2.89648	1.18327	1.34993
H	-0.7117	-1.88219	0.48289	H	0.24418	-1.2543	-0.53184
H	-5.8149	0.92016	1.41344	H	5.88393	0.80778	0.54593
H	-5.38349	1.61335	-0.16631	H	5.92526	0.10585	-1.08735
H	-6.49466	0.23894	-0.07761	H	6.68791	-0.7462	0.2697
H	-4.15355	-2.1221	0.90999	H	3.81044	-2.59315	-0.33857
H	-5.00406	-0.96348	-1.7135	H	5.21412	-1.78173	2.10334
H	-3.32765	-1.35357	-1.29802	H	4.37652	-0.24006	2.34886
H	-3.77206	0.30835	-1.73507	H	3.44659	-1.69893	1.95944
atom	Con f. 3- 3m			atom	Con f. 3- 3n		
C	4.49262	-2.21394	1.4556	C	-3.1033	-0.37213	3.16933
C	3.9439	1.40684	1.9151	C	-3.26861	-2.89329	-0.29129
C	2.73319	1.45783	1.3471	C	-3.31023	-1.67798	-0.85132
C	2.18761	0.4831	0.31162	C	-2.40912	-0.56483	-0.33143
C	3.06118	-0.75162	-0.08171	C	-3.06919	0.39603	0.7288
C	3.36224	-1.57228	1.15685	C	-3.53662	-0.43199	1.90903
C	1.68619	1.22673	-0.944	C	-1.76511	0.21572	-1.4996
C	0.80076	0.33929	-1.85018	C	-0.76501	1.30928	-1.08581
C	1.54599	-0.96257	-2.18772	C	-1.41644	2.21443	-0.01514
C	2.16223	-1.66616	-0.9696	C	-1.98553	1.40965	1.15168
C	1.75757	2.53312	1.77602	C	-4.20847	-1.41061	-2.03472
C	4.3318	-0.38938	-0.86746	C	-4.31089	1.1349	0.1853
C	-0.57834	0.16477	-1.23799	C	0.60787	0.80881	-0.66461
C	-1.46204	1.32767	-1.18181	C	1.66736	1.79434	-0.44986
C	-2.86509	1.09172	-0.65527	C	2.94955	1.26752	0.16968
C	-2.89869	-0.04494	0.34847	C	3.27516	-0.10805	-0.38323
O	-2.27184	-1.22351	-0.2355	O	2.13726	-0.99941	-0.20738
C	-1.09533	-1.03026	-0.85437	C	0.92041	-0.49997	-0.48629
O	-1.14005	2.43851	-1.62133	O	1.53197	2.99761	-0.69772
C	-4.30419	-0.49211	0.79369	C	4.4838	-0.83833	0.22842
C	-4.20424	-1.66463	1.7751	C	5.76703	-0.06731	-0.06586
O	-5.06521	-0.88385	-0.36121	O	4.61739	-2.09455	-0.4623
C	-5.06077	0.67386	1.42499	C	4.31471	-1.09121	1.72865
H	1.28744	0.05825	0.78624	H	-1.61484	-1.05947	0.23508
H	0.64752	0.89483	-2.78361	H	-0.58447	1.93647	-1.96824
H	-2.30033	0.21686	1.23079	H	3.4345	-0.03926	-1.46735
H	4.57067	-2.81864	2.35551	H	-3.52873	-1.01731	3.93417

H	5.38109	-2.15936	0.83296	H	-2.3217	0.30736	3.49599
H	4.22408	2.13319	2.67488	H	-3.89857	-3.70671	-0.64383
H	4.68313	0.65442	1.67719	H	-2.6055	-3.11442	0.54127
H	2.51243	-1.67556	1.8341	H	-4.32504	-1.14402	1.67003
H	1.11306	2.10722	-0.65693	H	-1.28278	-0.49652	-2.1799
H	2.54099	1.59117	-1.52373	H	-2.55594	0.70097	-2.07973
H	0.88464	-1.65833	-2.71758	H	-0.69014	2.94174	0.35485
H	2.34516	-0.71077	-2.89325	H	-2.21507	2.79046	-0.49607
H	1.37466	-2.08322	-0.33337	H	-1.16587	0.86835	1.64083
H	2.76448	-2.51726	-1.3076	H	-2.40361	2.09144	1.90139
H	1.64873	3.31587	1.0164	H	-4.83544	-0.52636	-1.89017
H	0.75614	2.11606	1.94078	H	-3.62057	-1.22915	-2.94234
H	2.08602	3.01497	2.70096	H	-4.86239	-2.26578	-2.22747
H	5.0235	0.22419	-0.28799	H	-5.14807	0.44705	0.03791
H	4.8604	-1.29903	-1.16919	H	-4.63408	1.89485	0.90526
H	4.09539	0.15923	-1.78115	H	-4.11809	1.6371	-0.76599
H	-3.22777	2.01845	-0.20679	H	3.76093	1.96836	-0.03418
H	-3.51573	0.85396	-1.50686	H	2.79891	1.2236	1.25588
H	-0.60017	-1.97773	-1.03292	H	0.19645	-1.30197	-0.56759
H	-5.2094	-1.98222	2.06643	H	5.79684	0.87673	0.48291
H	-3.65678	-1.37334	2.6773	H	5.85951	0.14346	-1.13596
H	-3.68884	-2.516	1.32251	H	6.62671	-0.66955	0.24229
H	-4.59135	-1.62063	-0.77746	H	3.7819	-2.57272	-0.34645
H	-6.02243	0.32008	1.80767	H	5.15796	-1.68693	2.08958
H	-4.49376	1.09889	2.25807	H	4.28962	-0.15636	2.29579
H	-5.25563	1.4629	0.69439	H	3.39032	-1.64049	1.93066

atom	Con f. 3- 3o			atom	Con f. 3- 3p		
C	-3.03374	3.24089	0.34821	C	-3.03997	3.25278	0.22698
C	-2.68908	-0.12179	2.94466	C	-2.71072	-0.02894	2.93867
C	-2.93962	-0.72226	1.77468	C	-2.95579	-0.66243	1.78508
C	-2.23549	-0.25603	0.50657	C	-2.24062	-0.23599	0.50934
C	-3.03926	0.78153	-0.37003	C	-3.0366	0.77104	-0.40843
C	-3.41286	1.96999	0.49288	C	-3.41975	1.98715	0.41089
C	-1.75276	-1.44924	-0.33537	C	-1.74944	-1.45564	-0.28907
C	-0.84435	-1.03595	-1.51476	C	-0.83326	-1.08245	-1.47591
C	-1.55875	0.03719	-2.35396	C	-1.53946	-0.03585	-2.35455
C	-2.11441	1.2062	-1.52962	C	-2.1	1.15879	-1.57162
C	-3.87868	-1.90169	1.70474	C	-3.89827	-1.84056	1.74325
C	-4.36136	0.20849	-0.92393	C	-4.35251	0.17906	-0.95757
C	0.5506	-0.67589	-1.03378	C	0.56085	-0.71103	-1.00082
C	1.40629	-1.75508	-0.5462	C	1.41207	-1.78014	-0.47769
C	2.83428	-1.38132	-0.19523	C	2.84604	-1.39986	-0.15909
C	2.91716	0.05222	0.29746	C	2.92076	0.04365	0.30105
O	2.30843	0.93406	-0.68718	O	2.31968	0.9075	-0.69692
C	1.10934	0.55591	-1.15727	C	1.12066	0.51559	-1.1617
O	1.04062	-2.93655	-0.50397	O	1.03758	-2.9557	-0.39195
C	4.33245	0.5828	0.56567	C	4.33075	0.59582	0.58217
C	4.28572	2.06128	0.96495	C	5.24536	0.51451	-0.64093
O	5.04655	0.43309	-0.67416	O	4.81593	-0.26585	1.62713
C	5.00466	-0.24596	1.6648	C	4.23583	2.03254	1.10188
H	-1.3444	0.29418	0.83386	H	-1.35347	0.32611	0.82704
H	-0.7208	-1.92729	-2.14226	H	-0.7087	-1.99415	-2.07323
H	2.3239	0.16082	1.21467	H	2.31853	0.15778	1.21282
H	-3.38793	4.00633	1.03442	H	-3.4006	4.04085	0.88358
H	-2.36995	3.57767	-0.44236	H	-2.36904	3.5628	-0.56856
H	-3.17381	-0.43894	3.86517	H	-3.20247	-0.31738	3.86488
H	-1.99272	0.70975	3.01997	H	-2.01166	0.80191	2.99422
H	-4.08481	1.72714	1.3145	H	-4.0989	1.77124	1.23415
H	-1.21666	-2.15137	0.30862	H	-1.2177	-2.13545	0.38181
H	-2.61066	-1.99536	-0.74073	H	-2.60385	-2.01559	-0.68245
H	-0.88946	0.42146	-3.13289	H	-0.86339	0.32292	-3.13971
H	-2.38579	-0.45299	-2.87995	H	-2.36297	-0.54131	-2.87151
H	-1.2899	1.78657	-1.10618	H	-1.27837	1.7501	-1.15775
H	-2.66084	1.88635	-2.19352	H	-2.63876	1.8192	-2.26119
H	-3.33957	-2.81782	1.43563	H	-3.36026	-2.76601	1.50561
H	-4.36353	-2.07117	2.67034	H	-4.3906	-1.97973	2.70991
H	-4.65919	-1.76979	0.94962	H	-4.67284	-1.72881	0.97891
H	-5.08867	0.03939	-0.12536	H	-5.09072	0.04227	-0.16284
H	-4.80595	0.92309	-1.62522	H	-4.78635	0.86695	-1.69155
H	-4.22025	-0.736	-1.45519	H	-4.20639	-0.78528	-1.4503
H	3.20086	-2.07749	0.56096	H	3.24217	-2.06796	0.60701
H	3.45072	-1.50538	-1.09436	H	3.43313	-1.54894	-1.07441
H	0.63183	1.37452	-1.68322	H	0.64627	1.31901	-1.71268

H	3.70499	2.20135	1.88234	H	4.82479	1.06895	-1.48463
H	3.83926	2.66599	0.17283	H	5.4039	-0.52261	-0.94792
H	5.30246	2.42407	1.14822	H	6.22078	0.95005	-0.40007
H	5.95483	0.73295	-0.52049	H	5.70983	0.03255	1.8518
H	4.41242	-0.24128	2.58523	H	3.83261	2.70706	0.34205
H	5.1518	-1.28124	1.34763	H	3.59169	2.07644	1.98583
H	5.98625	0.18185	1.89328	H	5.23214	2.39104	1.38144
atom	Con f. 3- 3q			atom	Con f. 3- 3r		
C	3.57688	-2.62408	1.55569	C	3.56888	-2.65127	1.53807
C	3.496	1.60584	2.08427	C	3.50762	1.57383	2.12063
C	3.32312	1.5038	0.76075	C	3.34272	1.49001	0.79484
C	2.36616	0.46583	0.18866	C	2.38396	0.46457	0.20321
C	3.03306	-0.89412	-0.25311	C	3.04642	-0.89379	-0.25016
C	3.79986	-1.4732	0.91875	C	3.80092	-1.49191	0.9201
C	1.50508	1.05965	-0.93935	C	1.53504	1.07706	-0.92405
C	0.36911	0.11978	-1.3991	C	0.40033	0.14839	-1.40788
C	0.96517	-1.25118	-1.76335	C	0.99276	-1.22039	-1.78452
C	1.88913	-1.83489	-0.68512	C	1.90042	-1.82217	-0.70262
C	4.0302	2.44765	-0.18067	C	4.06066	2.4423	-0.1297
C	4.04896	-0.72776	-1.40381	C	4.07229	-0.71917	-1.39074
C	-0.78323	0.09007	-0.40842	C	-0.76334	0.11115	-0.4302
C	-1.52347	1.3277	-0.162	C	-1.50036	1.34585	-0.18288
C	-2.47601	1.29671	1.01615	C	-2.48429	1.30907	0.96912
C	-3.2228	-0.02615	1.13109	C	-3.2228	-0.01773	1.09284
O	-2.27805	-1.13671	1.0495	O	-2.29957	-1.14172	0.96276
C	-1.21183	-1.02493	0.23293	C	-1.21696	-1.01601	0.17208
O	-1.34021	2.36647	-0.8055	O	-1.31387	2.38828	-0.82169
C	-4.35886	-0.23079	0.09847	C	-4.41246	-0.25404	0.12993
C	-4.89049	-1.66478	0.15704	C	-5.52894	0.74068	0.44026
O	-3.895	0.06272	-1.23328	O	-4.95455	-1.55048	0.44352
C	-5.4851	0.76671	0.36913	C	-4.03894	-0.20816	-1.35423
H	1.68798	0.18531	1.00429	H	1.69819	0.17819	1.01041
H	-0.04802	0.55754	-2.31534	H	-0.00691	0.60023	-2.3218
H	-3.65598	-0.1276	2.12817	H	-3.61894	-0.12732	2.10419
H	4.2143	-2.93453	2.38002	H	4.19838	-2.97598	2.36299
H	2.76856	-3.30096	1.29502	H	2.76071	-3.32162	1.2606
H	4.16966	2.34141	2.51785	H	4.18233	2.30012	2.56803
H	2.97152	0.95423	2.7788	H	2.97533	0.91591	2.80318
H	4.63696	-0.85978	1.24832	H	4.63711	-0.88603	1.26545
H	1.08445	2.01193	-0.60767	H	1.1151	2.02633	-0.58271
H	2.13163	1.28586	-1.80782	H	2.17039	1.31334	-1.78336
H	0.16623	-1.96695	-1.9898	H	0.1931	-1.92855	-2.03116
H	1.53414	-1.12726	-2.69157	H	1.57313	-1.08667	-2.70423
H	1.30745	-2.08791	0.20597	H	1.30681	-2.07864	0.17983
H	2.31672	-2.77595	-1.05049	H	2.32458	-2.76299	-1.07252
H	3.31377	3.10315	-0.68977	H	4.6134	1.92093	-0.91658
H	4.73768	3.08203	0.36089	H	3.35075	3.10566	-0.63773
H	4.57745	1.91905	-0.96648	H	4.76527	3.06835	0.42514
H	4.94321	-0.1929	-1.07266	H	4.96696	-0.19381	-1.04586
H	4.37035	-1.71471	-1.7542	H	4.39063	-1.70358	-1.75088
H	3.63214	-0.19039	-2.25931	H	3.66553	-0.16885	-2.24282
H	-1.86589	1.43714	1.91793	H	-1.89863	1.47396	1.88276
H	-3.16581	2.13884	0.95269	H	-3.18597	2.13954	0.87855
H	-0.72222	-1.98674	0.13682	H	-0.73933	-1.9812	0.0516
H	-4.10981	-2.384	-0.1051	H	-6.42783	0.45062	-0.11159
H	-5.71782	-1.77542	-0.54962	H	-5.76621	0.74335	1.50883
H	-5.25534	-1.90888	1.15964	H	-5.25765	1.75469	0.13769
H	-3.30081	-0.65065	-1.50944	H	-4.23147	-2.1883	0.33883
H	-5.15114	1.79712	0.22387	H	-3.64425	0.76895	-1.64691
H	-6.30884	0.57824	-0.32514	H	-3.29501	-0.97057	-1.60116
H	-5.86182	0.6618	1.39028	H	-4.93525	-0.4043	-1.95028
atom	Con f. 3- 3s			atom	Con f. 3- 3t		
C	4.90532	-1.75057	1.30872	C	-4.44618	2.42385	-0.90096
C	4.01578	2.29469	-0.27305	C	-3.79246	-0.4316	2.40961
C	3.29807	1.54233	0.56993	C	-2.90882	0.3772	1.8123
C	2.32059	0.47433	0.09869	C	-2.17783	0.00342	0.53011
C	2.99283	-0.92384	-0.17504	C	-2.99275	0.32199	-0.77973
C	3.63046	-1.41407	1.10692	C	-3.26417	1.80996	-0.82866
C	1.48179	0.93583	-1.10342	C	-1.68111	-1.45115	0.54122
C	0.34017	-0.04704	-1.45095	C	-0.75709	-1.78143	-0.65427
C	0.91348	-1.46347	-1.63617	C	-1.47327	-1.41648	-1.96678
C	1.85128	-1.92325	-0.51147	C	-2.06598	-0.00005	-1.9848
C	3.39489	1.74036	2.06047	C	-2.55638	1.71526	2.40857
C	4.00768	-0.87187	-1.32234	C	-4.28947	-0.48886	-0.87725
C	-0.81828	0.07546	-0.47577	C	0.62171	-1.17076	-0.46847

C	-1.59712	1.30882	-0.49144	C	1.47584	-1.70805	0.58777
C	-2.59458	1.48605	0.63489	C	2.89057	-1.16083	0.66078
C	-3.23931	0.18549	1.10061	C	2.94137	0.27391	0.17125
O	-2.26379	-0.88392	1.21466	O	2.35244	0.35809	-1.15592
C	-1.20665	-0.89971	0.38327	C	1.17182	-0.2604	-1.31402
O	-1.42932	2.20331	-1.32979	O	1.12767	-2.63289	1.33217
C	-4.43906	-0.3094	0.25362	C	4.33276	0.9158	0.08964
C	-4.12875	-0.47765	-1.23493	C	4.98441	0.93208	1.47514
O	-5.40101	0.74693	0.43492	O	4.06123	2.26866	-0.32681
C	-4.98999	-1.61043	0.84163	C	5.23377	0.23001	-0.93963
H	1.61946	0.30026	0.92688	H	-1.28581	0.64284	0.47756
H	-0.06647	0.27377	-2.41799	H	-0.60651	-2.86832	-0.64402
H	-3.61711	0.31528	2.11701	H	2.31277	0.90162	0.81726
H	5.24269	-2.10221	2.28044	H	-4.5101	3.50839	-0.93886
H	5.65935	-1.68935	0.52958	H	-5.38858	1.88464	-0.92315
H	4.70118	3.05409	0.09581	H	-4.29341	-0.13552	3.32832
H	3.94751	2.18913	-1.35114	H	-4.0471	-1.41023	2.01496
H	2.93328	-1.50203	1.94198	H	-2.3626	2.42475	-0.80822
H	1.06591	1.92398	-0.89172	H	-1.154	-1.64197	1.47835
H	2.11685	1.05227	-1.9869	H	-2.53043	-2.14113	0.51765
H	0.09917	-2.18877	-1.75206	H	-0.79787	-1.54322	-2.82111
H	1.467	-1.47267	-2.58155	H	-2.28075	-2.14219	-2.11199
H	1.28414	-2.1006	0.40787	H	-1.26305	0.74437	-1.99805
H	2.29804	-2.88684	-0.78354	H	-2.63262	0.14473	-2.91229
H	3.70358	0.81752	2.56331	H	-1.48411	1.76197	2.64045
H	4.10839	2.52802	2.32027	H	-2.75924	2.52882	1.70373
H	2.41557	2.0114	2.4766	H	-3.11546	1.9108	3.32851
H	4.83309	-0.19272	-1.09654	H	-4.96883	-0.25812	-0.0536
H	4.42628	-1.86766	-1.50028	H	-4.80503	-0.2633	-1.81642
H	3.54909	-0.53865	-2.25561	H	-4.09947	-1.56397	-0.85869
H	-2.0391	1.93068	1.47079	H	3.243	-1.23518	1.69108
H	-3.36657	2.19728	0.33736	H	3.52568	-1.80758	0.0416
H	-0.68158	-1.84239	0.48033	H	0.70054	0.05159	-2.23885
H	-3.35614	-1.23231	-1.40172	H	5.25233	-0.07289	1.81185
H	-3.80625	0.46371	-1.68727	H	4.31603	1.38523	2.21419
H	-5.03471	-0.80716	-1.75572	H	5.90274	1.52684	1.43266
H	-6.17358	0.52721	-0.10684	H	4.9159	2.71729	-0.4094
H	-5.22898	-1.48188	1.90213	H	6.18404	0.76994	-1.0088
H	-5.90613	-1.89349	0.3129	H	5.45718	-0.80217	-0.6553
H	-4.27259	-2.42892	0.7421	H	4.76343	0.22785	-1.92598

atom	Con f. 3- 4a			atom	Con f. 3- 4b		
C	4.6151	-2.31564	0.94122	C	-4.39345	-1.03591	2.4465
C	3.86495	2.045	1.29898	C	-4.21131	-0.94198	-2.07622
C	3.01984	1.02607	1.49687	C	-3.30839	-1.34587	-1.17466
C	2.24465	0.36288	0.36664	C	-2.34283	-0.3958	-0.47664
C	3.04946	-0.77391	-0.36867	C	-2.94206	0.28904	0.80785
C	3.40347	-1.84823	0.6369	C	-3.23594	-0.78469	1.83293
C	1.67814	1.38283	-0.63409	C	-1.73877	0.6354	-1.45544
C	0.71129	0.74796	-1.66031	C	-0.69639	1.58701	-0.84154
C	1.40015	-0.4435	-2.34996	C	-1.27738	2.22686	0.44085
C	2.08068	-1.43064	-1.39129	C	-1.8207	1.17996	1.41148
C	2.76078	0.49068	2.88128	C	-3.17552	-2.80269	-0.81215
C	4.29535	-0.24086	-1.08461	C	-4.18577	1.12935	0.49907
C	-0.64904	0.46192	-1.04734	C	0.6726	0.97896	-0.58712
C	-1.46669	1.59971	-0.63027	C	1.78933	1.88427	-0.3226
C	-2.71617	1.27249	0.16266	C	3.16325	1.2526	-0.20199
C	-3.36558	-0.01053	-0.32563	C	3.07966	-0.16096	0.34117
O	-2.37865	-1.08153	-0.37686	O	2.13518	-0.93101	-0.4577
C	-1.15389	-0.7801	-0.8437	C	0.96092	-0.33928	-0.73467
O	-1.14823	2.77446	-0.84868	O	1.66372	3.11457	-0.29067
C	-4.50776	-0.54151	0.56426	C	4.39808	-0.95881	0.31517
C	-5.08012	-1.83735	-0.0197	C	4.17995	-2.36626	0.88024
O	-3.99972	-0.7829	1.88701	O	4.87251	-1.04248	-1.03931
C	-5.60401	0.5095	0.71523	C	5.48257	-0.22692	1.10136
H	1.38494	-0.14359	0.82716	H	-1.52583	-1.01605	-0.0932
H	0.52455	1.51002	-2.42704	H	-0.531	2.40079	-1.55868
H	-3.73078	0.12033	-1.3521	H	2.67928	-0.14519	1.36297
H	4.73984	-3.10512	1.67803	H	-4.47249	-1.82934	3.18537
H	5.52328	-1.93909	0.48006	H	-5.30041	-0.47399	2.24394
H	4.40107	2.49996	2.12846	H	-4.88129	-1.65169	-2.55559
H	4.05073	2.46451	0.31525	H	-4.32029	0.09697	-2.37068
H	2.53944	-2.27943	1.14576	H	-2.36919	-1.39458	2.09398
H	1.1665	2.17597	-0.0842	H	-1.30859	0.1016	-2.31028

H	2.49208	1.86556	-1.18341	H	-2.54268	1.25567	-1.86257
H	0.6829	-0.98286	-2.98009	H	-0.5116	2.82904	0.93645
H	2.15688	-0.03608	-3.02934	H	-2.07632	2.91823	0.15004
H	1.32979	-1.99054	-0.8245	H	-0.99579	0.53129	1.73447
H	2.63681	-2.17442	-1.97419	H	-2.2065	1.66843	2.31399
H	1.69869	0.59329	3.14049	H	-3.89522	-3.42342	-1.35411
H	2.99574	-0.57737	2.94361	H	-2.16518	-3.16677	-1.04107
H	3.3512	1.01656	3.6375	H	-3.32773	-2.95747	0.26156
H	5.00583	0.1966	-0.37946	H	-5.0134	0.50394	0.15742
H	4.80083	-1.05336	-1.61625	H	-4.51308	1.66814	1.39434
H	4.04327	0.52567	-1.82008	H	-3.992	1.86997	-0.2793
H	-2.43123	1.1615	1.21716	H	3.62265	1.24038	-1.19893
H	-3.41071	2.11044	0.08555	H	3.77936	1.88213	0.44239
H	-0.60092	-1.68731	-1.05601	H	0.25265	-1.06408	-1.11927
H	-4.30783	-2.60584	-0.11042	H	3.42258	-2.90908	0.30837
H	-5.86953	-2.21455	0.63664	H	5.11798	-2.92659	0.83298
H	-5.50794	-1.66277	-1.01214	H	3.85356	-2.32166	1.92425
H	-3.28222	-1.42994	1.80025	H	4.18209	-1.48893	-1.5541
H	-6.43865	0.08735	1.28224	H	6.38443	-0.84456	1.14099
H	-5.2385	1.38846	1.25173	H	5.74175	0.7234	0.62816
H	-5.97687	0.82522	-0.26334	H	5.15393	-0.03094	2.12603
atom		Con f. 3- 4c		atom		Con f. 3- 4d	
C	-3.20343	3.05925	1.05004	C	-2.74445	-1.23231	3.0298
C	-2.87332	-0.81633	2.77958	C	-3.27207	-2.70378	-0.9698
C	-3.05781	-1.11904	1.48875	C	-3.34983	-1.38135	-1.1641
C	-2.30446	-0.35696	0.40635	C	-2.39353	-0.44411	-0.4368
C	-3.08838	0.84965	-0.24039	C	-2.94856	0.17546	0.9028
C	-3.55095	1.78566	0.85797	C	-3.29915	-0.9557	1.8485
C	-1.75674	-1.30852	-0.67049	C	-1.84607	0.63817	-1.3952
C	-0.79213	-0.61686	-1.66015	C	-0.80348	1.58804	-0.7823
C	-1.46995	0.63489	-2.24504	C	-1.36193	2.16479	0.5387
C	-2.10733	1.5582	-1.19775	C	-1.83128	1.06424	1.4882
C	-3.97133	-2.25243	1.08999	C	-4.34576	-0.80952	-2.1442
C	-4.35595	0.40981	-1.00489	C	-4.23699	0.99996	0.6986
C	0.58332	-0.40046	-1.05166	C	0.58099	0.99507	-0.5872
C	1.38022	-1.57961	-0.71839	C	1.68928	1.90582	-0.3078
C	2.64735	-1.32994	0.07425	C	3.07381	1.28994	-0.2366
C	3.32301	-0.03666	-0.34738	C	3.01702	-0.14464	0.2522
O	2.3633	1.05952	-0.32724	O	2.07307	-0.89532	-0.5656
C	1.12395	0.81518	-0.78894	C	0.88506	-0.31135	-0.7963
O	1.03242	-2.73114	-1.00545	O	1.54644	3.13184	-0.2232
C	4.4859	0.41405	0.55934	C	4.34556	-0.92196	0.1801
C	5.08884	1.724	0.04112	C	4.15351	-2.35362	0.691
O	3.99388	0.59731	1.8974	O	4.80592	-0.94495	-1.1815
C	5.55435	-0.67247	0.64367	C	5.42833	-0.20621	0.9834
H	-1.44286	0.10983	0.90024	H	-1.55862	-1.06654	-0.1022
H	-0.63173	-1.3229	-2.48455	H	-0.67506	2.42999	-1.4741
H	3.67555	-0.11767	-1.38335	H	2.62863	-0.17469	1.2784
H	-3.62166	3.63001	1.87555	H	-3.09352	-2.07346	3.6238
H	-2.50359	3.58708	0.40909	H	-1.93443	-0.64813	3.4562
H	-3.39448	-1.34875	3.57189	H	-3.93983	-3.39608	-1.4774
H	-2.1964	-0.023	3.08701	H	-2.53918	-3.13807	-0.2943
H	-4.26298	1.33896	1.55009	H	-4.10856	-1.59525	1.4994
H	-1.24873	-2.14663	-0.18683	H	-1.43465	0.14866	-2.286
H	-2.58283	-1.73875	-1.24533	H	-2.6779	1.25568	-1.7479
H	-0.75388	1.20544	-2.84852	H	-0.60118	2.77793	1.0283
H	-2.24879	0.29444	-2.93661	H	-2.19436	2.83548	0.2964
H	-1.32932	2.02956	-0.59041	H	-0.97165	0.43177	1.7424
H	-2.63116	2.37266	-1.71187	H	-2.18199	1.50904	2.4267
H	-4.71382	-1.94693	0.34714	H	-4.93604	0.00335	-1.7126
H	-3.40347	-3.0751	0.63922	H	-3.83898	-0.39258	-3.0229
H	-4.50155	-2.65056	1.95975	H	-5.03363	-1.58415	-2.4946
H	-5.12667	0.04252	-0.32195	H	-5.08401	0.35553	0.4475
H	-4.77678	1.2672	-1.54135	H	-4.49008	1.52691	1.6253
H	-4.1534	-0.37515	-1.73742	H	-4.13676	1.74599	-0.0935
H	2.37827	-1.2747	1.13724	H	3.51606	1.32147	-1.2408
H	3.31985	-2.17819	-0.06236	H	3.69291	1.90278	0.4209
H	0.59251	1.74673	-0.9427	H	0.1799	-1.02983	-1.1985
H	5.50604	1.59194	-0.96235	H	5.09857	-2.89815	0.6109
H	4.33607	2.51562	-0.0028	H	3.83901	-2.35499	1.7395
H	5.89168	2.04533	0.71079	H	3.39698	-2.88424	0.1067
H	3.29101	1.26442	1.85074	H	4.11649	-1.38098	-1.7064
H	5.17107	-1.56761	1.13948	H	5.6709	0.76443	0.5439
H	6.4065	-0.30199	1.22066	H	5.10678	-0.05286	2.0176
H	5.90792	-0.94779	-0.35412	H	6.33825	-0.81313	0.992

atom	Con f. 3- 4e			atom	Con f. 3- 4f		
C	4.6469	-2.30201	0.92713	C	-4.21932	1.63058	-1.75786
C	3.90229	2.0664	1.23632	C	-3.158	0.94546	2.46195
C	3.06285	1.04917	1.46483	C	-2.34902	1.25124	1.44046
C	2.26701	0.36903	0.35904	C	-1.78244	0.20503	0.49115
C	3.05933	-0.77673	-0.37572	C	-2.75262	-0.1732	-0.68961
C	3.43029	-1.83832	0.6373	C	-3.03485	1.07592	-1.49608
C	1.67951	1.37475	-0.64385	C	-1.27849	-1.044	1.2303
C	0.69511	0.72559	-1.64384	C	-0.50759	-2.02124	0.31178
C	1.37407	-0.47283	-2.33138	C	-1.37479	-2.36249	-0.91328
C	2.07398	-1.44696	-1.37341	C	-1.97493	-1.14053	-1.62412
C	2.83093	0.53346	2.86142	C	-1.91402	2.67207	1.19134
C	4.29287	-0.25291	-1.11918	C	-4.0477	-0.83237	-0.20366
C	-0.65468	0.44538	-1.00482	C	0.892	-1.51358	0.00272
C	-1.46673	1.59048	-0.59233	C	1.86636	-1.46889	1.08588
C	-2.70618	1.26953	0.22314	C	3.30002	-1.16707	0.69398
C	-3.34953	-0.0114	-0.2746	C	3.44261	-0.266	-0.52446
O	-2.3697	-1.08882	-0.28625	O	2.52192	-0.67645	-1.58157
C	-1.15042	-0.79613	-0.77201	C	1.32146	-1.17876	-1.23977
O	-1.15106	2.76174	-0.82959	O	1.59632	-1.76619	2.25633
C	-4.5544	-0.54268	0.5218	C	3.29358	1.25974	-0.30784
C	-5.72049	0.43628	0.42413	C	4.42176	1.76087	0.59136
O	-4.99995	-1.74637	-0.13074	O	3.514	1.88518	-1.58617
C	-4.202	-0.84936	1.97936	C	1.92937	1.69412	0.23598
H	1.41718	-0.13221	0.84317	H	-0.90947	0.66063	0.00731
H	0.4934	1.47768	-2.41656	H	-0.3654	-2.94467	0.88722
H	-3.66245	0.11663	-1.31916	H	4.42633	-0.4109	-0.97525
H	4.78402	-3.08217	1.67162	H	-4.29216	2.53184	-2.36128
H	5.54718	-1.93131	0.44616	H	-5.15469	1.22102	-1.38823
H	4.45331	2.53399	2.04882	H	-3.54086	1.71619	3.1267
H	4.06881	2.47198	0.24335	H	-3.46357	-0.0736	2.67698
H	2.57484	-2.26296	1.16576	H	-2.14025	1.55025	-1.90348
H	1.17711	2.17422	-0.09462	H	-0.63946	-0.7343	2.05954
H	2.48156	1.85138	-1.21538	H	-2.11996	-1.58507	1.67434
H	0.64664	-1.02132	-2.94173	H	-0.8009	-2.95919	-1.63224
H	2.11755	-0.07198	-3.02922	H	-2.19028	-3.00714	-0.56785
H	1.33457	-2.00192	-0.78714	H	-1.18543	-0.56329	-2.11611
H	2.62154	-2.19632	-1.95726	H	-2.64817	-1.47901	-2.42066
H	1.77346	0.63687	3.13857	H	-2.17864	2.99559	0.17869
H	3.07035	-0.53286	2.93501	H	-2.36729	3.36775	1.90387
H	3.43351	1.07204	3.59886	H	-0.82245	2.75879	1.27241
H	5.01532	0.19334	-0.43189	H	-4.62505	-0.16018	0.43527
H	4.78918	-1.07209	-1.64923	H	-4.6733	-1.10753	-1.05898
H	4.02835	0.50439	-1.85982	H	-3.85223	-1.74415	0.36491
H	-2.39452	1.16637	1.27045	H	3.83004	-0.75076	1.55221
H	-3.40487	2.10536	0.15743	H	3.7643	-2.13711	0.473
H	-0.59588	-1.7051	-0.97164	H	0.70842	-1.29858	-2.12515
H	-5.94446	0.67286	-0.62085	H	5.39456	1.40441	0.23814
H	-6.60908	-0.01698	0.87325	H	4.42891	2.85468	0.57545
H	-5.5046	1.36538	0.95664	H	4.28232	1.43781	1.62549
H	-4.24648	-2.35667	-0.14135	H	2.86832	1.50189	-2.20006
H	-5.06605	-1.30804	2.46845	H	1.9276	2.78169	0.35614
H	-3.35944	-1.54479	2.03965	H	1.12771	1.42846	-0.457
H	-3.93883	0.05738	2.53139	H	1.71277	1.24278	1.20839

atom	Con f. 3- 4g			atom	Con f. 3- 4h		
C	-4.33134	-1.07429	2.46406	C	-3.23622	3.05783	1.0259
C	-4.21919	-0.92827	-2.06088	C	-2.931	-0.81413	2.76624
C	-3.3006	-1.33647	-1.17729	C	-3.09386	-1.11931	1.4731
C	-2.33058	-0.3878	-0.48358	C	-2.32405	-0.35794	0.40186
C	-2.91467	0.27817	0.81771	C	-3.09889	0.84647	-0.25952
C	-3.18538	-0.80921	1.83483	C	-3.5801	1.78387	0.82973
C	-1.74875	0.65905	-1.45948	C	-1.75735	-1.31084	-0.66399
C	-0.70008	1.60662	-0.85	C	-0.77724	-0.61985	-1.63854
C	-1.26668	2.22974	0.44707	C	-1.447	0.62988	-2.2369
C	-1.79044	1.16992	1.4145	C	-2.10346	1.55433	-1.20239
C	-3.1539	-2.79612	-0.83197	C	-3.99892	-2.25477	1.06124
C	-4.16897	1.11284	0.53804	C	-4.35351	0.4037	-1.04323
C	0.6723	0.99625	-0.61893	C	0.58833	-0.4009	-1.00853
C	1.7891	1.89883	-0.33922	C	1.37889	-1.5814	-0.66014
C	3.16491	1.26241	-0.23916	C	2.63382	-1.32601	0.1542
C	3.05869	-0.15388	0.29005	C	3.30564	-0.04177	-0.29673
O	2.13599	-0.91307	-0.54275	O	2.35399	1.06001	-0.25938
C	0.95964	-0.31833	-0.80355	C	1.12071	0.81739	-0.73705
O	1.66281	3.12764	-0.28224	O	1.03512	-2.73308	-0.94912
C	4.35358	-0.9806	0.35836	C	4.52836	0.42639	0.51216

C	5.31681	-0.36141	1.36668	C	5.66505	-0.58203	0.37552
O	4.00867	-2.2727	0.89288	O	5.00746	1.6382	-0.10043
C	5.01228	-1.1502	-1.01284	C	4.18964	0.69254	1.98077
H	-1.50382	-1.00687	-0.11946	H	-1.47122	0.11071	0.90908
H	-0.54585	2.42883	-1.56008	H	-0.60247	-1.32697	-2.45907
H	2.6112	-0.13747	1.29266	H	3.60952	-0.13598	-1.34755
H	-4.39382	-1.87641	3.1951	H	-3.66846	3.62951	1.84353
H	-5.2447	-0.51559	2.2824	H	-2.52596	3.5851	0.39607
H	-4.89228	-1.63656	-2.53801	H	-3.46433	-1.34595	3.55083
H	-4.33812	0.11324	-2.34215	H	-2.26049	-0.01914	3.08326
H	-2.31075	-1.41651	2.07478	H	-4.3034	1.33789	1.51054
H	-1.3321	0.13883	-2.32926	H	-1.25676	-2.14737	-0.17003
H	-2.56239	1.28118	-1.84383	H	-2.57296	-1.7435	-1.25193
H	-0.49796	2.83138	0.93844	H	-0.72176	1.20039	-2.82934
H	-2.07357	2.91901	0.17368	H	-2.21363	0.28679	-2.94071
H	-0.95734	0.52317	1.71978	H	-1.33652	2.02881	-0.58352
H	-2.16669	1.64722	2.32694	H	-2.62012	2.36661	-1.7271
H	-2.14521	-3.1521	-1.08016	H	-4.54193	-2.65307	1.92298
H	-3.28839	-2.96265	0.24234	H	-4.73034	-1.95146	0.30656
H	-3.87847	-3.41549	-1.36895	H	-3.42259	-3.07676	0.62008
H	-4.99672	0.48542	0.2003	H	-5.13442	0.03628	-0.37205
H	-4.48719	1.63719	1.44509	H	-4.76689	1.25965	-1.58768
H	-3.99288	1.86548	-0.23302	H	-4.13812	-0.38214	-1.77106
H	3.606	1.27189	-1.24407	H	2.33475	-1.25942	1.20808
H	3.79333	1.87619	0.40896	H	3.31043	-2.17546	0.04561
H	0.25136	-1.03241	-1.20737	H	0.58822	1.74752	-0.89517
H	4.82727	-0.2163	2.33485	H	5.87776	-0.78924	-0.67801
H	6.16908	-1.03239	1.50837	H	6.56821	-0.17005	0.8351
H	5.6946	0.60184	1.01597	H	5.42445	-1.52216	0.87715
H	3.34655	-2.65876	0.29878	H	4.27621	2.27455	-0.0788
H	5.87031	-1.82169	-0.91823	H	5.06937	1.10461	2.48321
H	4.3107	-1.58249	-1.73258	H	3.37045	1.41256	2.06807
H	5.36774	-0.19562	-1.41074	H	3.89753	-0.22404	2.50104
atom	Con f. 3- 4i			atom	Con f. 3- 4j		
C	4.67742	-2.26376	0.98334	C	-2.91879	1.82218	-2.50414
C	3.90039	2.09884	1.19368	C	-1.8223	2.4732	1.59308
C	3.06714	1.08184	1.44496	C	-2.25367	1.20559	1.5995
C	2.27541	0.37199	0.35504	C	-1.79588	0.23995	0.51612
C	3.07539	-0.7845	-0.35451	C	-2.79259	0.0229	-0.68507
C	3.45639	-1.81914	0.68237	C	-3.15553	1.37239	-1.2707
C	1.68103	1.35122	-0.66953	C	-1.34369	-1.10072	1.11756
C	0.70192	0.67326	-1.65591	C	-0.61822	-2.00531	0.0951
C	1.38514	-0.5395	-2.31363	C	-1.50157	-2.16967	-1.155
C	2.09406	-1.48559	-1.33456	C	-2.05143	-0.85139	-1.71858
C	2.83798	0.59675	2.85289	C	-3.14212	0.69941	2.70879
C	4.30362	-0.26874	-1.11218	C	-4.118	-0.64992	-0.26927
C	-0.64952	0.40837	-1.01498	C	0.79976	-1.51977	-0.15844
C	-1.47529	1.56156	-0.66315	C	1.77193	-1.63482	0.92157
C	-2.7285	1.26896	0.13847	C	3.21239	-1.31511	0.57206
C	-3.35076	-0.05041	-0.28843	C	3.37489	-0.27229	-0.52492
O	-2.35265	-1.10737	-0.22668	O	2.45486	-0.53781	-1.62804
C	-1.13525	-0.82414	-0.71955	C	1.24093	-1.04779	-1.35127
O	-1.16093	2.72553	-0.93988	O	1.49176	-2.07364	2.04427
C	-4.5522	-0.51404	0.54842	C	3.24784	1.21753	-0.12138
C	-5.02823	-1.89658	0.09027	C	4.38023	1.58459	0.83555
O	-4.07996	-0.58581	1.90531	O	3.48379	1.99254	-1.31223
C	-5.69036	0.50501	0.43926	C	1.88835	1.60716	0.46543
H	1.42943	-0.12428	0.85074	H	-0.91659	0.70055	0.05321
H	0.50096	1.40562	-2.44747	H	-0.51514	-2.99176	0.56424
H	-3.66276	0.01273	-1.3389	H	4.35869	-0.37543	-0.9866
H	4.8219	-3.02507	1.74576	H	-3.24615	2.81435	-2.80519
H	5.57429	-1.89516	0.49441	H	-2.40175	1.23942	-3.26064
H	4.44814	2.58801	1.99566	H	-2.12037	3.18246	2.36187
H	4.0649	2.48329	0.19203	H	-1.16109	2.84557	0.81465
H	2.6049	-2.23994	1.22017	H	-3.68488	2.02719	-0.57998
H	1.17139	2.15845	-0.13824	H	-0.68581	-0.91018	1.96863
H	2.47997	1.82172	-1.25049	H	-2.20595	-1.64911	1.51095
H	0.65756	-1.10723	-2.90604	H	-0.95374	-2.70385	-1.94038
H	2.12382	-0.15334	-3.02494	H	-2.34258	-2.81581	-0.87946
H	1.35975	-2.03304	-0.73491	H	-1.23504	-0.25482	-2.1354
H	2.6469	-2.24379	-1.90187	H	-2.72692	-1.07372	-2.55292
H	1.77993	0.70044	3.12759	H	-3.42725	1.5117	3.38341
H	3.08332	-0.46627	2.95041	H	-4.05633	0.2348	2.32802
H	3.4375	1.15529	3.5779	H	-2.63033	-0.06674	3.30302
H	5.02384	0.19904	-0.43695	H	-4.73306	0.01857	0.33899

H	4.80544	-1.09612	-1.62404	H	-4.69757	-0.90246	-1.16396
H	4.03186	0.469	-1.86968	H	-3.96092	-1.57094	0.29723
H	-2.45258	1.22645	1.19963	H	3.74636	-1.01845	1.47635
H	-3.43149	2.09248	0.00421	H	3.65987	-2.25868	0.23323
H	-0.56732	-1.73546	-0.86456	H	0.62804	-1.04465	-2.24486
H	-4.23621	-2.63979	0.20283	H	5.34906	1.26059	0.44226
H	-5.88522	-2.20747	0.69672	H	4.40377	2.67152	0.9566
H	-5.34399	-1.87622	-0.95776	H	4.23271	1.13716	1.82104
H	-4.83864	-0.81888	2.46087	H	2.81998	1.71357	-1.96197
H	-5.98824	0.65831	-0.60272	H	1.90368	2.67242	0.71477
H	-6.56255	0.13566	0.98855	H	1.08912	1.44098	-0.26024
H	-5.40322	1.46847	0.86745	H	1.65187	1.04836	1.37524
atom		Con f. 3- 4k		atom		Con f. 3- 4l	
C	-4.24194	1.65571	-1.71481	C	4.64555	-2.28105	0.99627
C	-3.14453	0.91157	2.47409	C	3.89977	2.08529	1.1926
C	-2.34035	1.23093	1.45297	C	3.05739	1.07546	1.44218
C	-1.7821	0.19796	0.48463	C	2.26587	0.36896	0.35001
C	-2.76144	-0.16389	-0.6936	C	3.06102	-0.79418	-0.35419
C	-3.05406	1.09783	-1.47634	C	3.42918	-1.83003	0.68616
C	-1.27281	-1.0609	1.20278	C	1.68293	1.35045	-0.67911
C	-0.50845	-2.02467	0.26467	C	0.70153	0.6772	-1.66637
C	-1.38001	-2.34558	-0.96306	C	1.38085	-0.53915	-2.32133
C	-1.98844	-1.11377	-1.64958	C	2.07978	-1.4892	-1.33867
C	-1.90165	2.654	1.22426	C	2.81678	0.59594	2.85013
C	-4.05044	-0.83458	-0.20722	C	4.29686	-0.28802	-1.10606
C	0.89077	-1.51361	-0.03838	C	-0.65079	0.41752	-1.02463
C	1.87092	-1.50775	1.04011	C	-1.46491	1.57636	-0.65501
C	3.30072	-1.18091	0.65662	C	-2.69866	1.27982	0.17534
C	3.43501	-0.25835	-0.54776	C	-3.33764	-0.021	-0.27811
O	2.51359	-0.63364	-1.60632	O	-2.36408	-1.09553	-0.25096
C	1.31467	-1.14213	-1.27259	C	-1.14414	-0.81506	-0.74435
O	1.6058	-1.85235	2.19917	O	-1.14912	2.73838	-0.93438
C	3.30031	1.25772	-0.25466	C	-4.55136	-0.49851	0.54183
C	1.99283	1.65812	0.43235	C	-4.21624	-0.6934	2.02122
O	4.41005	1.49861	0.63137	O	-5.49703	0.57356	0.38117
C	3.49342	2.05582	-1.54638	C	-5.11977	-1.7831	-0.06565
H	-0.91274	0.6594	-0.00081	H	1.41432	-0.12074	0.84276
H	-0.36548	-2.95753	0.82424	H	0.50409	1.40978	-2.45863
H	4.42246	-0.39238	-0.99438	H	-3.65275	0.08322	-1.32534
H	-4.32258	2.56712	-2.30178	H	4.78039	-3.04203	1.76078
H	-5.1726	1.23899	-1.34129	H	5.54787	-1.91822	0.51312
H	-3.52142	1.67291	3.15292	H	4.44711	2.57241	1.99608
H	-3.45159	-0.10989	2.6751	H	4.07236	2.46567	0.19076
H	-2.16466	1.58003	-1.8858	H	2.57168	-2.24528	1.21879
H	-0.62707	-0.76191	2.03105	H	1.17898	2.16371	-0.15174
H	-2.11086	-1.60727	1.64676	H	2.48765	1.81209	-1.25916
H	-0.80638	-2.92626	-1.69523	H	0.6533	-1.10287	-2.91755
H	-2.1916	-3	-0.6269	H	2.12508	-0.1568	-3.0288
H	-1.20421	-0.52642	-2.13803	H	1.33965	-2.03218	-0.74216
H	-2.66675	-1.44122	-2.44643	H	2.6305	-2.25086	-1.90337
H	-2.35137	3.34032	1.94802	H	3.05218	-0.46896	2.95151
H	-0.80965	2.73571	1.30499	H	3.41744	1.15097	3.57686
H	-2.16685	2.9935	0.21699	H	1.75828	0.70979	3.11917
H	-4.62352	-0.17502	0.4485	H	5.01686	0.17529	-0.42756
H	-4.68329	-1.09655	-1.06136	H	4.79535	-1.11955	-1.61445
H	-3.84737	-1.75544	0.34368	H	4.03415	0.4508	-1.86572
H	3.82076	-0.75141	1.51435	H	-2.37674	1.21189	1.22247
H	3.78372	-2.14054	0.43121	H	-3.40973	2.10264	0.09053
H	0.69714	-1.23617	-2.15799	H	-0.58636	-1.72941	-0.90654
H	1.85294	1.13067	1.37953	H	-3.91952	0.25031	2.48647
H	2.01728	2.73255	0.64602	H	-5.09654	-1.07139	2.55158
H	1.13095	1.46618	-0.20963	H	-3.40551	-1.41625	2.14935
H	4.37095	2.43113	0.89131	H	-6.29375	0.32814	0.87479
H	4.44464	1.79406	-2.02048	H	-4.40959	-2.61026	0.01314
H	2.68501	1.86611	-2.25718	H	-6.03461	-2.06824	0.4644
H	3.504	3.12709	-1.31979	H	-5.3641	-1.63081	-1.12171
atom		Con f. 3- 4m		atom		Con f. 3- 4n	
C	-4.28021	1.46802	-1.64116	C	4.6787	-1.97872	1.62864
C	-2.81314	1.22517	2.44679	C	4.1299	1.6625	1.53564
C	-2.07271	1.37783	1.34259	C	2.8824	1.6295	1.05245
C	-1.64285	0.21142	0.46624	C	2.27272	0.50318	0.22786
C	-2.72753	-0.26026	-0.57006	C	3.12454	-0.78471	-0.01645
C	-3.08096	0.91456	-1.45607	C	3.52376	-1.39063	1.31416
C	-1.09538	-0.96746	1.28505	C	1.69125	1.03618	-1.09776
C	-0.41416	-2.04165	0.40231	C	0.75357	0.0208	-1.7925

C	-1.37141	-2.47354	-0.72429	C	1.46071	-1.33826	-1.9398
C	-2.04564	-1.31862	-1.48016	C	2.1677	-1.82866	-0.66818
C	-1.56489	2.73177	0.91945	C	1.93403	2.76732	1.36549
C	-3.97056	-0.85268	0.10225	C	4.3289	-0.56462	-0.94604
C	0.96009	-1.56519	-0.03018	C	-0.60845	-0.01762	-1.11977
C	2.01176	-1.49448	0.98505	C	-1.44895	1.17606	-1.19844
C	3.39458	-1.11148	0.49506	C	-2.70199	1.16906	-0.34633
C	3.35065	-0.15063	-0.6835	C	-3.32167	-0.21568	-0.28714
O	2.45716	-0.68496	-1.70861	O	-2.3171	-1.19166	0.11434
C	1.2946	-1.2289	-1.30053	C	-1.09143	-1.07929	-0.42726
O	1.83296	-1.8316	2.16082	O	-1.14628	2.17075	-1.86793
C	2.96477	1.30812	-0.31618	C	-4.48034	-0.36877	0.71855
C	2.53854	2.09296	-1.55775	C	-5.01321	-1.80529	0.70245
O	1.90424	1.33838	0.66243	O	-4.00965	-0.03521	2.0351
C	4.1575	1.97941	0.36483	C	-5.5979	0.62195	0.4038
H	-0.81825	0.58238	-0.15804	H	1.4064	0.16648	0.82164
H	-0.24039	-2.91428	1.04302	H	0.56438	0.40838	-2.80111
H	4.31854	-0.11927	-1.18645	H	-3.66176	-0.51761	-1.28594
H	-4.40745	2.31218	-2.31411	H	4.82891	-2.42826	2.60687
H	-5.17491	1.11275	-1.13862	H	5.51808	-2.0294	0.94107
H	-3.09322	2.07943	3.05862	H	4.4602	2.5	2.14623
H	-3.16024	0.25387	2.78511	H	4.85284	0.87537	1.37149
H	-2.22979	1.33085	-1.99795	H	2.72735	-1.37903	2.06045
H	-0.38233	-0.59021	2.02172	H	1.13549	1.95659	-0.92181
H	-1.90427	-1.44042	1.85027	H	2.50788	1.29796	-1.77919
H	-0.84685	-3.11854	-1.43923	H	0.749	-2.10039	-2.27897
H	-2.14955	-3.09571	-0.26823	H	2.20399	-1.23839	-2.73805
H	-1.31415	-0.79668	-2.10573	H	1.43301	-2.12617	0.08725
H	-2.79564	-1.72801	-2.16739	H	2.74709	-2.72953	-0.90033
H	-0.46678	2.73606	0.89862	H	1.76907	3.41505	0.49671
H	-1.89816	2.97824	-0.0951	H	2.32357	3.39307	2.17319
H	-1.90132	3.52428	1.59443	H	0.94859	2.38996	1.66599
H	-4.47538	-0.11531	0.73068	H	5.05845	0.13314	-0.53114
H	-4.68135	-1.19486	-0.65687	H	4.8402	-1.514	-1.13358
H	-3.72281	-1.71086	0.73023	H	4.01727	-0.17364	-1.91622
H	3.97003	-0.69818	1.32475	H	-2.42791	1.49158	0.66681
H	3.89143	-2.03937	0.18289	H	-3.40844	1.89354	-0.7541
H	0.63632	-1.38487	-2.14736	H	-0.51768	-1.98016	-0.24536
H	1.62173	1.67919	-1.98734	H	-4.22651	-2.52155	0.95407
H	2.35369	3.13554	-1.28375	H	-5.81916	-1.90294	1.43537
H	3.31617	2.06767	-2.32732	H	-5.40867	-2.06439	-0.28492
H	1.0894	1.04528	0.2297	H	-3.28971	-0.65103	2.24281
H	3.88082	2.99737	0.65327	H	-5.94564	0.50168	-0.62628
H	4.45778	1.44061	1.26763	H	-6.44268	0.44355	1.07527
H	5.01369	2.02998	-0.31304	H	-5.2648	1.6533	0.54351
atom	Con f. 3- 4o			atom	Con f. 3- 4p		
C	-4.36178	-1.07479	2.45348	C	-2.91442	1.93682	-2.42058
C	-4.22017	-0.92821	-2.07067	C	-1.79124	2.4005	1.68882
C	-3.30747	-1.33718	-1.18134	C	-2.23555	1.13813	1.6429
C	-2.34123	-0.38941	-0.48129	C	-1.7901	0.2147	0.51872
C	-2.93263	0.27631	0.81689	C	-2.79219	0.05495	-0.68708
C	-3.21152	-0.81126	1.83144	C	-3.15461	1.43006	-1.21011
C	-1.753	0.65772	-1.45275	C	-1.34399	-1.15371	1.05994
C	-0.70674	1.60334	-0.8362	C	-0.62211	-2.01303	-0.00324
C	-1.27944	2.22584	0.4583	C	-1.50675	-2.11666	-1.25905
C	-1.81068	1.16588	1.42148	C	-2.05471	-0.77278	-1.76075
C	-3.16379	-2.79713	-0.836	C	-3.12838	0.59579	2.73128
C	-4.18371	1.11323	0.52958	C	-4.11723	-0.63377	-0.29689
C	0.66422	0.99182	-0.60023	C	0.7969	-1.51827	-0.23229
C	1.78176	1.89053	-0.32159	C	1.76675	-1.68539	0.84289
C	3.15565	1.25405	-0.21924	C	3.20489	-1.32689	0.5246
C	3.0625	-0.17043	0.29701	C	3.36438	-0.25161	-0.54205
O	2.12808	-0.92029	-0.5289	O	2.45492	-0.4725	-1.65321
C	0.95384	-0.32266	-0.78406	C	1.24152	-0.99381	-1.40202
O	1.6587	3.12063	-0.26498	O	1.48481	-2.19171	1.93696
C	4.37951	-0.95986	0.3172	C	3.24019	1.21259	-0.04774
C	4.13421	-2.40448	0.76447	C	1.93707	1.52629	0.68968
O	4.86397	-0.944	-1.03711	O	4.35366	1.323	0.85955
C	5.38829	-0.27738	1.2466	C	3.43877	2.17574	-1.22086
H	-1.5168	-1.00898	-0.11276	H	-0.90942	0.68805	0.07113
H	-0.54829	2.42637	-1.54444	H	-0.5222	-3.02052	0.41944
H	2.64095	-0.16655	1.31049	H	4.35662	-0.3342	-0.99044
H	-4.43021	-1.8774	3.18344	H	-3.24114	2.9418	-2.67635
H	-5.27304	-0.51437	2.26665	H	-2.39496	1.38952	-3.20151
H	-4.89041	-1.63607	-2.55242	H	-2.08043	3.07954	2.48766

H	-4.33682	0.1134	-2.35255	H	-1.12824	2.79885	0.92502
H	-2.33955	-1.42043	2.07627	H	-3.68606	2.05163	-0.49083
H	-1.33152	0.13785	-2.32038	H	-0.68496	-1.00441	1.91821
H	-2.56393	1.28076	-1.84139	H	-2.20884	-1.71514	1.42813
H	-0.51193	2.82525	0.95447	H	-0.961	-2.61533	-2.06887
H	-2.08349	2.91725	0.18184	H	-2.34904	-2.77308	-1.01294
H	-0.98045	0.5174	1.73099	H	-1.23727	-0.15968	-2.15072
H	-2.19174	1.64268	2.33222	H	-2.73171	-0.9557	-2.60341
H	-3.88474	-3.41579	-1.37867	H	-4.05353	0.16832	2.33349
H	-2.15349	-3.15326	-1.0773	H	-2.62946	-0.20814	3.28517
H	-3.30611	-2.96453	0.23718	H	-3.39467	1.37995	3.44567
H	-5.01016	0.48748	0.18561	H	-4.73041	0.00921	0.34005
H	-4.50726	1.63737	1.43487	H	-4.69906	-0.84855	-1.19991
H	-4.00101	1.86619	-0.23961	H	-3.95917	-1.57734	0.23067
H	3.61022	1.26433	-1.2177	H	3.71663	-1.02195	1.4391
H	3.77515	1.86831	0.43639	H	3.68276	-2.2528	0.17921
H	0.24432	-1.03599	-1.18772	H	0.63252	-0.95448	-2.29749
H	3.71599	-2.43657	1.77567	H	1.79215	0.87383	1.55452
H	3.44672	-2.91263	0.08515	H	1.96872	2.56064	1.04967
H	5.08265	-2.95168	0.77197	H	1.07572	1.43082	0.02648
H	5.70102	-1.43134	-1.04683	H	4.32058	2.21115	1.24521
H	6.2996	-0.88195	1.29937	H	4.38543	1.97038	-1.73076
H	5.66092	0.71425	0.87724	H	2.62611	2.09308	-1.94726
H	4.99046	-0.1776	2.2614	H	3.46203	3.20645	-0.85169

atom	Con f. 3- 4q			atom	Con f. 3- 4r		
C	5.06047	-0.4143	1.83391	C	5.04234	-0.32791	1.89781
C	3.69434	1.98045	-1.74355	C	3.71848	1.93732	-1.77994
C	3.11997	1.79556	-0.54888	C	3.12495	1.78665	-0.58983
C	2.24732	0.5906	-0.21948	C	2.25733	0.58501	-0.23654
C	3.05846	-0.66933	0.26228	C	3.07155	-0.65129	0.29913
C	3.75838	-0.32385	1.55855	C	3.74504	-0.25678	1.59556
C	1.27811	0.24806	-1.37224	C	1.31047	0.19745	-1.39311
C	0.30897	-0.91438	-1.09015	C	0.34726	-0.96379	-1.08833
C	1.10467	-2.13364	-0.57095	C	1.14373	-2.15912	-0.51762
C	2.01759	-1.77283	0.59964	C	2.03506	-1.75328	0.65497
C	3.28696	2.80848	0.55432	C	3.26395	2.83668	0.48217
C	4.04666	-1.17237	-0.79537	C	4.08286	-1.17813	-0.72401
C	-0.87197	-0.57626	-0.19495	C	-0.84955	-0.60845	-0.22078
C	-1.94856	-1.55869	-0.06566	C	-1.92134	-1.58972	-0.08918
C	-2.96821	-1.27437	1.01893	C	-2.96811	-1.29113	0.96529
C	-3.31974	0.20444	1.11909	C	-3.31258	0.18875	1.07808
O	-2.09555	0.99877	1.17877	O	-2.10284	1.00693	1.07886
C	-1.03089	0.60457	0.45242	C	-1.02769	0.59336	0.38169
O	-1.99834	-2.60031	-0.72834	O	-1.9657	-2.63853	-0.74303
C	-4.2357	0.74304	-0.00785	C	-4.28629	0.76959	0.0232
C	-4.34315	2.26804	0.06876	C	-5.66595	0.13725	0.19415
O	-3.73831	0.35166	-1.30158	O	-4.45943	2.16367	0.33762
C	-5.62028	0.10464	0.10572	C	-3.80025	0.63247	-1.42244
H	1.65042	0.87451	0.65394	H	1.64269	0.89056	0.61713
H	-0.1339	-1.20821	-2.05062	H	-0.07991	-1.29163	-2.04478
H	-3.80907	0.41073	2.07314	H	-3.75892	0.39118	2.05365
H	5.44249	-0.15242	2.81735	H	5.40389	-0.0298	2.87869
H	5.79341	-0.74733	1.10509	H	5.79126	-0.67971	1.19446
H	4.30803	2.85605	-1.94134	H	4.32813	2.81137	-1.99618
H	3.57816	1.2788	-2.56344	H	3.62247	1.20789	-2.5779
H	3.08525	0.01823	2.34674	H	3.05483	0.10662	2.35905
H	0.71782	1.1503	-1.64238	H	0.74678	1.08547	-1.70088
H	1.8603	-0.02235	-2.25784	H	1.90866	-0.09532	-2.26067
H	0.41741	-2.92888	-0.27189	H	0.45748	-2.94985	-0.20358
H	1.69985	-2.53522	-1.39881	H	1.75484	-2.58308	-1.32239
H	1.40142	-1.42581	1.43972	H	1.40321	-1.38546	1.4743
H	2.55034	-2.66508	0.94921	H	2.56988	-2.6293	1.04052
H	3.91599	3.64779	0.24322	H	3.70066	2.41483	1.394
H	2.31145	3.20559	0.86473	H	3.89187	3.67056	0.15462
H	3.73636	2.3531	1.44357	H	2.27987	3.23556	0.76175
H	4.83296	-0.43878	-0.98649	H	4.86193	-0.44061	-0.92913
H	4.52078	-2.10067	-0.46043	H	4.56426	-2.08662	-0.34759
H	3.552	-1.37925	-1.74648	H	3.60699	-1.42727	-1.67462
H	-3.85647	-1.88603	0.85812	H	-3.8618	-1.88684	0.77346
H	-2.51581	-1.59342	1.967	H	-2.54884	-1.62984	1.92155
H	-0.28531	1.39103	0.43918	H	-0.29124	1.38738	0.34291
H	-5.0252	2.62474	-0.70809	H	-5.66788	-0.90929	-0.11853
H	-4.72889	2.58607	1.0424	H	-5.99782	0.19482	1.23561

H	-3.36766	2.73868	-0.08091	H	-6.38536	0.67877	-0.42704
H	-2.94063	0.86761	-1.49107	H	-3.57575	2.56374	0.32831
H	-5.57895	-0.97317	-0.07027	H	-2.84599	1.1448	-1.57188
H	-6.0525	0.28203	1.09435	H	-3.68136	-0.41449	-1.71587
H	-6.28279	0.5432	-0.64578	H	-4.53867	1.08927	-2.08816
atom	Con f. 3- 4s			atom	Con f. 3- 4t		
C	-3.29195	3.05562	1.00481	C	-4.29968	-1.18786	2.4309
C	-2.93606	-0.79329	2.77718	C	-4.23824	-0.83738	-2.08129
C	-3.09462	-1.11255	1.4869	C	-3.30796	-1.28494	-1.22959
C	-2.33255	-0.3528	0.40904	C	-2.33162	-0.36737	-0.50363
C	-3.12058	0.83684	-0.26374	C	-2.90487	0.24009	0.83081
C	-3.61844	1.77589	0.81669	C	-3.16129	-0.89212	1.8016
C	-1.75407	-1.30859	-0.64788	C	-1.75711	0.71961	-1.43886
C	-0.78092	-0.61436	-1.62728	C	-0.7048	1.64188	-0.79746
C	-1.45712	0.62884	-2.2325	C	-1.26452	2.20979	0.52765
C	-2.13142	1.55124	-1.208	C	-1.77796	1.10989	1.45496
C	-3.98718	-2.26136	1.08496	C	-3.15418	-2.75891	-0.956
C	-4.3662	0.37228	-1.04916	C	-4.16473	1.08137	0.60112
C	0.58539	-0.39067	-1.0019	C	0.66829	1.02296	-0.5966
C	1.39746	-1.5674	-0.70005	C	1.78779	1.91379	-0.28739
C	2.66929	-1.3208	0.08738	C	3.15986	1.26812	-0.21805
C	3.30745	0.00057	-0.30935	C	3.04138	-0.16255	0.2702
O	2.32867	1.07184	-0.20135	O	2.12774	-0.89355	-0.58648
C	1.09791	0.82415	-0.67974	C	0.95407	-0.28422	-0.82974
O	1.05815	-2.71701	-1.00623	O	1.66253	3.13996	-0.18612
C	4.52732	0.41928	0.52463	C	4.3474	-0.97423	0.33616
C	5.02043	1.80703	0.1012	C	5.05563	-1.05993	-1.01652
O	4.07305	0.45786	1.88891	O	5.14746	-0.22784	1.27051
C	5.64742	-0.61407	0.37054	C	4.06616	-2.37042	0.89761
H	-1.48546	0.13009	0.91245	H	-1.50229	-1.00199	-0.17378
H	-0.60409	-1.32376	-2.44514	H	-0.55486	2.49279	-1.47375
H	3.60386	-0.03603	-1.36556	H	2.59016	-0.15911	1.27194
H	-3.73523	3.62769	1.81624	H	-4.35247	-2.02164	3.12643
H	-2.58542	3.58737	0.37454	H	-5.21614	-0.62409	2.28387
H	-3.46436	-1.32314	3.5665	H	-4.91578	-1.52369	-2.58359
H	-2.27413	0.01171	3.08687	H	-4.36255	0.21617	-2.31045
H	-4.33902	1.3258	1.49766	H	-2.28252	-1.50687	2.00455
H	-1.24236	-2.13419	-0.14624	H	-1.34595	0.2356	-2.33194
H	-2.56401	-1.75754	-1.23151	H	-2.57376	1.35608	-1.79204
H	-0.73096	1.20401	-2.81936	H	-0.49408	2.79378	1.03742
H	-2.21475	0.27771	-2.94227	H	-2.07548	2.9067	0.28771
H	-1.3744	2.04279	-0.59051	H	-0.94127	0.45322	1.72772
H	-2.65771	2.35155	-1.74157	H	-2.14859	1.54865	2.38881
H	-3.40117	-3.08254	0.65512	H	-2.14781	-3.10012	-1.23283
H	-4.52926	-2.65532	1.94931	H	-3.27541	-2.97734	0.11071
H	-4.71867	-1.97365	0.3243	H	-3.88392	-3.35384	-1.51327
H	-5.14432	-0.00273	-0.37891	H	-4.99276	0.46739	0.24013
H	-4.78971	1.21937	-1.59968	H	-4.47725	1.55996	1.53506
H	-4.13695	-0.41422	-1.77199	H	-3.99818	1.87142	-0.13394
H	2.41202	-1.30664	1.15394	H	3.5892	1.30258	-1.22752
H	3.3553	-2.15161	-0.08453	H	3.80602	1.84826	0.44258
H	0.54388	1.74907	-0.78755	H	0.24558	-0.98132	-1.26151
H	4.24245	2.55931	0.24645	H	5.95499	-1.67743	-0.9223
H	5.89044	2.08596	0.70466	H	4.40908	-1.51495	-1.77227
H	5.32188	1.81267	-0.95124	H	5.35972	-0.06986	-1.3662
H	4.84217	0.661	2.4418	H	5.99355	-0.69212	1.35608
H	5.93009	-0.74009	-0.67929	H	3.57072	-2.29791	1.87097
H	5.34966	-1.58551	0.77261	H	3.43018	-2.95121	0.22443
H	6.53198	-0.2757	0.91999	H	5.00832	-2.91309	1.02845

atom	Con f. 4- 1a			atom	Con f. 4- 1b		
C	-6.14953	-0.69337	-0.86366	C	-5.96183	-1.6208	-0.36677
C	-3.72744	2.10165	1.64343	C	-4.17324	2.3614	0.70599
C	-3.6606	1.8519	0.3303	C	-4.03036	1.61712	-0.39708
C	-2.86285	0.69273	-0.25303	C	-3.01572	0.4862	-0.50917
C	-3.65734	-0.6669	-0.29051	C	-3.54313	-0.89353	0.03733
C	-4.86945	-0.49256	-1.18003	C	-4.73453	-1.31961	-0.79333
C	-1.48746	0.53442	0.41986	C	-1.65634	0.86305	0.10872
C	-0.62694	-0.53541	-0.28281	C	-0.5904	-0.22183	-0.13827
C	-1.37476	-1.88289	-0.32986	C	-1.07633	-1.58543	0.39264
C	-2.74708	-1.71718	-0.98572	C	-2.43233	-1.95063	-0.21463
C	-4.36176	2.74004	-0.66466	C	-4.86299	1.88438	-1.62421
C	-4.04961	-1.15329	1.10905	C	-3.8865	-0.83587	1.53032
C	0.73276	-0.66514	0.36109	C	0.74834	0.15289	0.45361

C	1.83397	-0.37347	-0.37687	C	1.84006	0.15598	-0.35242
C	0.78488	-1.10674	1.79748	C	0.79394	0.47457	1.9213
C	3.22042	-0.44188	0.07266	C	3.21092	0.45963	0.04555
C	4.28413	-0.10126	-0.95832	C	4.28904	0.21875	-0.99849
O	3.54914	-0.78558	1.22302	O	3.51292	0.91679	1.16311
C	5.60161	0.4495	-0.37457	C	5.67381	-0.14341	-0.4215
O	6.1724	-0.51908	0.52622	O	6.13854	0.92782	0.42214
C	6.62219	0.64097	-1.49314	C	5.61011	-1.44713	0.38512
C	5.36273	1.77071	0.36846	C	6.69026	-0.26236	-1.55382
H	-2.66924	0.93971	-1.30604	H	-2.84091	0.32245	-1.58154
H	-0.47868	-0.20766	-1.31917	H	-0.46371	-0.32237	-1.22336
H	-6.93556	-0.54852	-1.60043	H	-6.73414	-1.9327	-1.06526
H	-6.4685	-1.00398	0.12679	H	-6.2498	-1.56998	0.67906
H	-4.30368	2.94035	2.02667	H	-4.9056	3.16391	0.75022
H	-3.21695	1.4902	2.38074	H	-3.57436	2.20574	1.59767
H	-4.63012	-0.18029	-2.19797	H	-4.52436	-1.39546	-1.86158
H	-0.96655	1.49736	0.38592	H	-1.32077	1.80958	-0.32886
H	-1.60679	0.28127	1.47689	H	-1.76575	1.03688	1.18259
H	-0.78229	-2.60923	-0.89771	H	-0.34097	-2.35741	0.13942
H	-1.48476	-2.28847	0.68111	H	-1.14449	-1.56122	1.48531
H	-2.60346	-1.41471	-2.0314	H	-2.31045	-2.07628	-1.2985
H	-3.27096	-2.67985	-1.00589	H	-2.77032	-2.91688	0.17754
H	-4.90802	3.54922	-0.17097	H	-5.43998	0.99887	-1.91169
H	-3.63863	3.1863	-1.35996	H	-5.56066	2.7123	-1.46738
H	-5.07045	2.16911	-1.27439	H	-4.22066	2.13189	-2.47957
H	-4.5435	-2.12821	1.045	H	-4.17657	-1.82817	1.89063
H	-3.17916	-1.26904	1.75809	H	-3.0359	-0.50485	2.1297
H	-4.73502	-0.45678	1.59698	H	-4.71324	-0.14899	1.72399
H	1.69121	-0.05726	-1.40657	H	1.70813	-0.12009	-1.3953
H	1.2612	-0.3433	2.42098	H	-0.1289	0.18324	2.42559
H	1.39364	-2.00962	1.90592	H	0.9341	1.55182	2.06989
H	-0.21122	-1.30677	2.19442	H	1.63996	-0.01411	2.40973
H	4.49954	-1.03356	-1.50042	H	3.95892	-0.55199	-1.70111
H	3.87505	0.59784	-1.69383	H	4.38139	1.1505	-1.57493
H	5.43462	-0.7629	1.11787	H	5.38567	1.11302	1.01605
H	6.80451	-0.30497	-2.01322	H	4.91624	-1.35442	1.22577
H	7.57151	0.99461	-1.07751	H	6.60067	-1.68527	0.78518
H	6.26992	1.37658	-2.22237	H	5.28048	-2.28285	-0.24107
H	6.30786	2.1454	0.77395	H	7.68347	-0.47482	-1.14465
H	4.94909	2.5308	-0.30266	H	6.41955	-1.0712	-2.23892
H	4.66581	1.63272	1.20034	H	6.74456	0.67189	-2.12196
atom	Con f. 4- 1c			atom	Con f. 4- 1d		
C	-6.05456	-1.23192	-0.76292	C	-5.66515	-1.92055	0.15431
C	-4.03761	2.21546	1.23806	C	-3.49585	1.45974	2.22199
C	-3.90254	1.75433	-0.01111	C	-3.88332	1.31737	0.94886
C	-2.96358	0.61196	-0.37709	C	-3.05036	0.58259	-0.09349
C	-3.59818	-0.81658	-0.18552	C	-3.3077	-0.97083	-0.12791
C	-4.79774	-0.93905	-1.10006	C	-4.75007	-1.21697	-0.51407
C	-1.60032	0.7306	0.32913	C	-1.5509	0.91192	0.02046
C	-0.6034	-0.3368	-0.16612	C	-0.73137	0.28364	-1.13184
C	-1.19479	-1.74966	0.00815	C	-0.94874	-1.2313	-1.15507
C	-2.55655	-1.85748	-0.68146	C	-2.43846	-1.56156	-1.27316
C	-4.66695	2.37379	-1.15259	C	-5.17881	1.91301	0.46156
C	-3.97059	-1.09653	1.27484	C	-2.95588	-1.64544	1.20289
C	0.73834	-0.20207	0.51428	C	0.70192	0.75716	-1.04575
C	1.82956	0.07775	-0.24295	C	1.70482	-0.08775	-0.69678
C	0.78691	-0.39562	2.00476	C	0.91241	2.2172	-1.34259
C	3.19776	0.23836	0.23791	C	3.11232	0.26821	-0.54867
C	4.24478	0.59942	-0.80322	C	4.07147	-0.86982	-0.24057
O	3.51927	0.12058	1.43487	O	3.54384	1.42781	-0.68526
C	5.67531	0.12224	-0.47703	C	5.3102	-0.46335	0.58513
O	6.09895	0.69254	0.77623	O	6.05804	0.53943	-0.12945
C	5.73847	-1.40888	-0.39761	C	4.89884	0.07936	1.96037
C	6.64814	0.64465	-1.53055	C	6.24491	-1.6601	0.73917
H	-2.7678	0.69654	-1.45491	H	-3.37471	0.95108	-1.0766
H	-0.45499	-0.17318	-1.24066	H	-1.1343	0.69798	-2.06898
H	-6.83178	-1.30725	-1.51915	H	-6.67136	-2.04252	-0.23851
H	-6.36242	-1.40583	0.2638	H	-5.45822	-2.39795	1.10742
H	-4.71485	3.03593	1.46288	H	-4.11362	1.99332	2.94027
H	-3.48651	1.80195	2.07683	H	-2.56004	1.05685	2.59595
H	-4.56845	-0.77799	-2.15481	H	-5.03588	-0.76759	-1.46661
H	-1.1895	1.72758	0.13718	H	-1.43495	1.99969	0.01876
H	-1.72517	0.64704	1.41217	H	-1.14195	0.55471	0.97109
H	-0.50706	-2.48809	-0.41937	H	-0.40655	-1.67669	-1.99699
H	-1.29197	-1.9887	1.07212	H	-0.54211	-1.68325	-0.24395

H	-2.4174	-1.72303	-1.76217	H	-2.81251	-1.17564	-2.23058
H	-2.9683	-2.8636	-0.54035	H	-2.58095	-2.64803	-1.29627
H	-5.31263	3.18907	-0.81287	H	-5.71545	2.42652	1.2648
H	-3.97686	2.77262	-1.90774	H	-4.99276	2.63392	-0.34532
H	-5.29092	1.6304	-1.66055	H	-5.83706	1.14199	0.04701
H	-4.33748	-2.12239	1.3822	H	-3.06689	-2.73127	1.11804
H	-3.11282	-0.98301	1.94088	H	-1.92493	-1.44196	1.49981
H	-4.75161	-0.41783	1.62436	H	-3.60497	-1.29924	2.01011
H	1.69236	0.19798	-1.3141	H	1.47373	-1.12939	-0.50797
H	1.19723	0.49021	2.49936	H	1.70149	2.35563	-2.08694
H	1.45217	-1.22435	2.26708	H	1.24063	2.75838	-0.44859
H	-0.20155	-0.59973	2.41857	H	-0.00765	2.67832	-1.70924
H	3.93524	0.22554	-1.78353	H	3.53551	-1.68289	0.25728
H	4.24557	1.69685	-0.87351	H	4.40881	-1.26326	-1.21034
H	5.35928	0.50908	1.38732	H	5.38782	1.19959	-0.39292
H	5.07593	-1.78967	0.38533	H	4.26519	0.96554	1.85958
H	6.75946	-1.72712	-0.16437	H	5.78979	0.36056	2.53052
H	5.44321	-1.86533	-1.34829	H	4.34633	-0.67377	2.53213
H	7.66975	0.34	-1.28062	H	7.14402	-1.3648	1.2901
H	6.40328	0.24943	-2.52092	H	5.75605	-2.47101	1.28725
H	6.61594	1.73806	-1.57436	H	6.55092	-2.03793	-0.24176
atom		Con f. 4- 1e		atom		Con f. 4- 1f	
C	-5.66475	-1.9209	0.15438	C	-5.42699	-1.10771	-1.91201
C	-3.49516	1.4596	2.22231	C	-3.73911	2.28995	1.54556
C	-3.88304	1.31715	0.94931	C	-3.64067	1.93111	0.26002
C	-3.05024	0.58266	-0.09335	C	-2.88241	0.69528	-0.20478
C	-3.3075	-0.97075	-0.12805	C	-3.69427	-0.65134	-0.08385
C	-4.74993	-1.21693	-0.51395	C	-5.0195	-0.49255	-0.80062
C	-1.55077	0.91201	0.02036	C	-1.49599	0.57878	0.45294
C	-0.73144	0.28405	-1.13226	C	-0.67176	-0.56712	-0.1695
C	-0.94876	-1.23091	-1.15585	C	-1.44513	-1.8988	-0.08678
C	-2.4385	-1.5612	-1.27364	C	-2.83315	-1.76292	-0.71853
C	-5.1789	1.91242	0.46253	C	-4.25132	2.76663	-0.83515
C	-2.95534	-1.64559	1.20256	C	-4.02544	-1.00564	1.38061
C	0.70186	0.75756	-1.04611	C	0.69974	-0.66286	0.45435
C	1.70473	-0.08745	-0.69733	C	1.78741	-0.44184	-0.3273
C	0.91233	2.21767	-1.34267	C	0.77796	-0.98468	1.92103
C	3.11223	0.26845	-0.54881	C	3.18179	-0.47567	0.09986
C	4.07118	-0.86975	-0.24069	C	4.22804	-0.21456	-0.9712
O	3.54383	1.42805	-0.68503	O	3.53195	-0.72696	1.26792
C	5.30985	-0.4636	0.58529	C	5.53259	0.42921	-0.45616
O	6.05797	0.53914	-0.12903	O	6.13835	-0.42972	0.52889
C	4.89831	0.07895	1.96055	C	6.5388	0.54155	-1.59835
C	6.24433	-1.66054	0.73933	C	5.25853	1.80849	0.1578
H	-3.37479	0.95134	-1.07633	H	-2.71027	0.81977	-1.28209
H	-1.1345	0.69861	-2.06923	H	-0.54115	-0.3319	-1.23303
H	-6.67103	-2.0429	-0.23826	H	-6.41344	-0.91332	-2.32544
H	-5.45754	-2.39859	1.10728	H	-4.81193	-1.82098	-2.45237
H	-4.11284	1.99296	2.94082	H	-4.28574	3.18304	1.83884
H	-2.55909	1.05697	2.59591	H	-3.27933	1.72006	2.34688
H	-5.03603	-0.76724	-1.46625	H	-5.70525	0.20289	-0.31661
H	-1.43488	1.99979	0.0189	H	-0.96071	1.52489	0.31776
H	-1.14158	0.55458	0.9708	H	-1.59774	0.42814	1.53114
H	-0.40676	-1.67604	-1.99803	H	-0.87946	-2.67932	-0.60803
H	-0.54187	-1.68308	-0.24496	H	-1.53313	-2.21916	0.95641
H	-2.81279	-1.17509	-2.23088	H	-2.70867	-1.54844	-1.78712
H	-2.58096	-2.64767	-1.29695	H	-3.36581	-2.71838	-0.64793
H	-4.99337	2.63344	-0.34437	H	-4.84273	3.59638	-0.43705
H	-5.83707	1.14123	0.04816	H	-3.46476	3.18281	-1.47891
H	-5.71539	2.42572	1.26601	H	-4.8918	2.16056	-1.48576
H	-3.06631	-2.73141	1.11754	H	-4.53199	-1.97604	1.4193
H	-1.92435	-1.4421	1.49929	H	-3.13399	-1.07063	2.00891
H	-3.60428	-1.29956	2.00999	H	-4.69201	-0.26194	1.82426
H	1.47362	-1.12914	-0.50881	H	1.62487	-0.20959	-1.37625
H	1.23992	2.7588	-0.4484	H	1.27194	-0.17615	2.46951
H	-0.0076	2.67869	-1.70976	H	1.38243	-1.88055	2.09263
H	1.70181	2.35631	-2.08655	H	-0.21141	-1.14295	2.35251
H	3.53498	-1.68277	0.25698	H	4.4689	-1.19101	-1.41581
H	4.4086	-1.26315	-1.21044	H	3.79707	0.39464	-1.77093
H	5.38798	1.19962	-0.39223	H	5.40946	-0.64808	1.14151
H	4.2648	0.96523	1.85982	H	6.74397	-0.44412	-2.02844
H	5.78921	0.35991	2.5309	H	7.48108	0.95869	-1.22787
H	4.34561	-0.67423	2.53206	H	6.16048	1.19483	-2.39017
H	7.14336	-1.3655	1.29052	H	6.19425	2.24703	0.5186
H	5.75521	-2.47148	1.28713	H	4.82052	2.4886	-0.58033

H	6.5505	-2.03823	-0.2416	H	4.56933	1.73179	1.00394
atom	Con f. 4- 1g			atom	Con f. 4- 1h		
C	-6.0501	-0.14534	0.87613	C	5.26249	2.22504	-0.95569
C	-2.75822	2.84589	0.21608	C	3.88948	-1.87794	1.85364
C	-3.26123	1.91079	-0.59893	C	4.09689	-1.45077	0.60249
C	-2.79062	0.4621	-0.58617	C	3.14469	-0.51163	-0.1252
C	-3.54312	-0.43762	0.46694	C	3.33858	1.01353	0.22175
C	-5.01237	-0.47985	0.10767	C	4.78351	1.39524	-0.02739
C	-1.26191	0.34542	-0.44232	C	1.67464	-0.94352	0.02346
C	-0.78579	-1.09886	-0.61766	C	0.73954	-0.09161	-0.86792
C	-1.48599	-2.00859	0.41866	C	0.90924	1.39216	-0.52993
C	-3.00692	-1.88752	0.30869	C	2.37398	1.81563	-0.67678
C	-4.31912	2.25751	-1.61448	C	5.28636	-1.91788	-0.19628
C	-3.3281	0.04636	1.90556	C	3.05576	1.31591	1.70843
C	0.70851	-1.31936	-0.5636	C	-0.6661	-0.64338	-0.79901
C	1.5478	-0.37106	-0.07751	C	-1.66022	0.02916	-0.16635
C	1.1724	-2.66084	-1.06358	C	-0.85951	-1.98071	-1.46147
C	2.99609	-0.49619	0.05947	C	-3.03932	-0.42375	-0.01411
C	3.72119	0.71698	0.61952	C	-3.97689	0.50557	0.73996
O	3.62524	-1.53163	-0.22374	O	-3.4527	-1.51826	-0.43903
C	5.2153	0.81461	0.2533	C	-5.46204	0.39438	0.33745
O	5.90386	-0.36299	0.71766	O	-5.92477	-0.95178	0.56162
C	5.4022	0.96139	-1.26269	C	-6.31058	1.28793	1.23796
C	5.85217	1.99188	0.98668	C	-5.66048	0.77206	-1.13658
H	-3.04951	0.03596	-1.56556	H	3.38503	-0.58708	-1.19406
H	-1.11096	-1.4439	-1.61131	H	1.07537	-0.24041	-1.90573
H	-7.06881	-0.23383	0.50734	H	6.32867	2.4257	-1.02516
H	-5.93469	0.22759	1.88943	H	4.62982	2.73558	-1.67575
H	-3.11328	3.87296	0.17883	H	4.58838	-2.55516	2.33866
H	-1.98131	2.63042	0.94278	H	3.02265	-1.58022	2.43511
H	-5.21085	-0.84566	-0.90107	H	5.48956	0.93299	0.66251
H	-0.95123	0.72234	0.53634	H	1.59419	-2.00145	-0.24394
H	-0.78755	0.98856	-1.19128	H	1.3445	-0.85555	1.06353
H	-1.19537	-3.05327	0.26827	H	0.28245	2.00089	-1.19143
H	-1.14465	-1.72641	1.42146	H	0.56873	1.58647	0.49273
H	-3.31669	-2.26989	-0.67278	H	2.67136	1.68323	-1.72442
H	-3.48761	-2.52434	1.06048	H	2.47661	2.88349	-0.4523
H	-3.96253	2.04459	-2.63084	H	5.8504	-1.07076	-0.60285
H	-5.22251	1.65619	-1.46734	H	5.96464	-2.53377	0.4015
H	-4.59843	3.31403	-1.56277	H	4.95533	-2.51007	-1.05998
H	-2.26825	0.08457	2.16559	H	3.12142	2.39524	1.88296
H	-3.74286	1.04514	2.0582	H	2.06233	0.9877	2.02322
H	-3.81464	-0.63588	2.61004	H	3.7875	0.82671	2.3561
H	1.13893	0.57901	0.24675	H	-1.44419	0.99552	0.27466
H	1.95989	-2.54727	-1.81393	H	0.05301	-2.30055	-1.96984
H	1.60738	-3.25686	-0.25419	H	-1.67367	-1.94019	-2.19107
H	0.34387	-3.2221	-1.50194	H	-1.14181	-2.74806	-0.73317
H	3.1958	1.62808	0.31667	H	-3.87894	0.24735	1.80446
H	3.62552	0.65493	1.71349	H	-3.63468	1.53951	0.63809
H	5.37178	-1.10544	0.3729	H	-5.24933	-1.5144	0.13597
H	6.46866	1.02448	-1.50099	H	-6.17835	1.0109	2.28882
H	4.9112	1.86727	-1.63345	H	-7.36987	1.1776	0.9835
H	4.98385	0.10267	-1.7957	H	-6.03523	2.33996	1.11807
H	6.92191	2.04154	0.75822	H	-6.71862	0.68637	-1.40324
H	5.39101	2.93702	0.68502	H	-5.33873	1.80155	-1.32567
H	5.73631	1.8782	2.06936	H	-5.08905	0.10872	-1.79255
atom	Con f. 4- 1i			atom	Con f. 4- 1j		
C	-5.8977	-1.091	-0.02905	C	-5.48634	-1.29732	-1.7781
C	-2.74646	2.95673	0.37357	C	-4.20189	2.6908	-0.96458
C	-3.22471	2.04526	-0.48191	C	-3.53757	2.01554	-0.01878
C	-2.79219	0.58553	-0.47068	C	-2.86158	0.69197	-0.35511
C	-3.541	-0.30674	0.59255	C	-3.72512	-0.5953	-0.05885
C	-5.03595	-0.16359	0.39178	C	-5.03564	-0.4942	-0.81295
C	-1.26464	0.42844	-0.35668	C	-1.46322	0.5986	0.28473
C	-0.83356	-1.02665	-0.55995	C	-0.69569	-0.6564	-0.17563
C	-1.55609	-1.93986	0.45933	C	-1.52415	-1.92182	0.11436
C	-3.0745	-1.76069	0.37751	C	-2.90751	-1.81603	-0.53034
C	-4.20881	2.42473	-1.55827	C	-3.40674	2.57752	1.37579
C	-3.24757	0.13703	2.04048	C	-4.08309	-0.74168	1.436
C	0.65449	-1.29009	-0.52234	C	0.67953	-0.70919	0.44731
C	1.52575	-0.36503	-0.04751	C	1.7643	-0.55087	-0.35257
C	1.07617	-2.6425	-1.03019	C	0.76367	-0.91263	1.93455
C	2.97242	-0.52561	0.06588	C	3.15975	-0.54629	0.07396
C	3.73879	0.67664	0.59406	C	4.19358	-0.22358	-0.99248
O	3.57025	-1.58063	-0.21321	O	3.51899	-0.81556	1.23485

C	5.22281	0.73947	0.18091	C	5.49363	0.41503	-0.46079
O	5.9012	-0.44746	0.63633	O	6.11866	-0.47734	0.48159
C	5.36467	0.86711	-1.34156	C	6.48961	0.58967	-1.6039
C	5.906	1.91065	0.88174	C	5.20729	1.76301	0.21442
H	-3.08333	0.16422	-1.44222	H	-2.71967	0.68234	-1.44298
H	-1.17362	-1.34138	-1.55881	H	-0.57103	-0.58466	-1.26317
H	-6.95671	-0.86406	-0.12288	H	-6.45823	-1.12523	-2.23418
H	-5.59773	-2.10065	-0.29344	H	-4.92545	-2.14801	-2.15378
H	-3.07438	3.99265	0.33211	H	-4.69341	3.63739	-0.75213
H	-2.0134	2.7124	1.13577	H	-4.28026	2.31633	-1.98214
H	-5.42526	0.82224	0.6461	H	-5.66808	0.33407	-0.49773
H	-0.92703	0.78751	0.61997	H	-0.88879	1.49132	0.01239
H	-0.78733	1.0672	-1.1077	H	-1.54534	0.59948	1.37546
H	-1.30592	-2.98881	0.27185	H	-1.00116	-2.80082	-0.27911
H	-1.1896	-1.70398	1.46511	H	-1.62031	-2.07001	1.19502
H	-3.40636	-2.09842	-0.61207	H	-2.77946	-1.75591	-1.61829
H	-3.56239	-2.41066	1.11324	H	-3.47822	-2.73001	-0.32993
H	-5.09399	1.7791	-1.53426	H	-4.01827	3.47676	1.49088
H	-4.53381	3.46538	-1.46764	H	-3.70637	1.85943	2.14453
H	-3.75722	2.2911	-2.55052	H	-2.36724	2.85001	1.5945
H	-3.63014	1.1433	2.22849	H	-4.55104	-1.71673	1.61016
H	-3.73918	-0.5463	2.74117	H	-3.2074	-0.6704	2.08587
H	-2.17965	0.13757	2.27001	H	-4.79714	0.02461	1.74973
H	1.14721	0.59602	0.28075	H	1.59953	-0.38594	-1.41404
H	1.87684	-2.55127	-1.7692	H	1.16432	-1.90734	2.16187
H	1.47891	-3.26045	-0.22021	H	1.44958	-0.19683	2.39403
H	0.23338	-3.17058	-1.48234	H	-0.21469	-0.82519	2.4099
H	3.22396	1.59637	0.2996	H	4.44204	-1.17586	-1.48317
H	3.67598	0.62481	1.69091	H	3.74551	0.41434	-1.76004
H	5.33964	-1.18205	0.32254	H	5.40048	-0.71913	1.09762
H	6.42403	0.90528	-1.61434	H	6.70513	-0.37371	-2.07717
H	4.88081	1.77926	-1.70627	H	7.42863	1.00376	-1.22189
H	4.91189	0.01205	-1.85181	H	6.09687	1.27101	-2.36445
H	6.96939	1.93446	0.62184	H	6.1407	2.19863	0.58466
H	5.45664	2.8623	0.58278	H	4.7526	2.46794	-0.48949
H	5.81941	1.81124	1.96856	H	4.52813	1.64052	1.06324
atom		Con f. 4- 1k		atom		Con f. 4- 1l	
C	-5.77563	-1.23787	0.57152	C	-5.14794	-2.46203	-0.95335
C	-2.84961	2.95811	-0.04926	C	-4.73608	1.67538	-1.88474
C	-3.38165	1.90552	-0.68181	C	-3.97323	1.59383	-0.78784
C	-2.8596	0.48484	-0.51878	C	-3.03354	0.40983	-0.59199
C	-3.41218	-0.26942	0.75198	C	-3.63203	-0.7856	0.24731
C	-4.92634	-0.22234	0.73654	C	-4.90654	-1.2639	-0.41861
C	-1.32308	0.41322	-0.5846	C	-1.66721	0.86529	-0.0446
C	-0.83454	-1.03756	-0.63662	C	-0.65115	-0.29119	0.00647
C	-1.36295	-1.81765	0.59118	C	-1.21799	-1.45904	0.83768
C	-2.88713	-1.71857	0.68907	C	-2.57027	-1.90494	0.27983
C	-4.52315	2.07193	-1.65173	C	-3.99562	2.69463	0.2447
C	-2.96532	0.40039	2.06786	C	-4.0103	-0.37415	1.68664
C	0.65823	-1.23571	-0.76299	C	0.69407	0.16294	0.52045
C	1.53148	-0.23463	-0.48601	C	1.77954	0.02654	-0.28321
C	1.08523	-2.61737	-1.17866	C	0.75556	0.73193	1.91069
C	2.98681	-0.32873	-0.53556	C	3.15219	0.39048	0.05006
C	3.75858	0.94022	-0.21168	C	4.20307	0.1356	-1.01806
O	3.59737	-1.36806	-0.84706	O	3.477	0.90689	1.13542
C	5.15371	0.70844	0.40568	C	5.61129	-0.18185	-0.47215
O	5.96345	-0.05746	-0.50693	O	6.07962	0.92405	0.32353
C	5.86767	2.04531	0.58667	C	5.59594	-1.46003	0.37658
C	5.04613	-0.02982	1.74664	C	6.59613	-0.31842	-1.63014
H	-3.24203	-0.0895	-1.37319	H	-2.8566	-0.01656	-1.58729
H	-1.28195	-1.50947	-1.52482	H	-0.51493	-0.65696	-1.01883
H	-6.84991	-1.07245	0.59039	H	-6.11634	-2.68837	-1.3928
H	-5.45024	-2.26185	0.41408	H	-4.41198	-3.26007	-0.97906
H	-3.2451	3.96031	-0.19618	H	-5.41442	2.50981	-2.04743
H	-2.00481	2.86697	0.62616	H	-4.71065	0.90838	-2.65461
H	-5.34085	0.77252	0.89955	H	-5.70528	-0.52429	-0.4353
H	-0.97973	0.95416	-1.473	H	-1.27601	1.66144	-0.68833
H	-0.89212	0.92508	0.28052	H	-1.78691	1.2992	0.95225
H	-1.07271	-2.87106	0.52457	H	-0.51493	-2.29926	0.81446
H	-0.88726	-1.41658	1.49352	H	-1.31882	-1.16008	1.88639
H	-3.31997	-2.21579	-0.18764	H	-2.41793	-2.27801	-0.74048
H	-3.23731	-2.26909	1.57	H	-2.95365	-2.74573	0.86922
H	-4.90127	3.09834	-1.6676	H	-4.16568	2.31449	1.25615
H	-4.19865	1.80986	-2.66783	H	-3.03845	3.22908	0.27102
H	-5.35253	1.39757	-1.41104	H	-4.77813	3.424	0.01754

H	-3.32598	-0.18558	2.92002	H	-4.28557	-1.26373	2.26367
H	-1.87873	0.47482	2.15169	H	-3.18924	0.12201	2.21023
H	-3.37687	1.40897	2.15592	H	-4.87091	0.30014	1.69461
H	1.14475	0.73512	-0.19503	H	1.63612	-0.40206	-1.27117
H	1.73532	-2.57485	-2.05756	H	-0.22043	0.7161	2.3974
H	1.67091	-3.10532	-0.39251	H	1.1181	1.76486	1.89045
H	0.21952	-3.24227	-1.40933	H	1.46558	0.17458	2.52895
H	3.87893	1.48219	-1.16082	H	3.86854	-0.66299	-1.68651
H	3.15917	1.58244	0.44003	H	4.25983	1.05046	-1.6253
H	5.39441	-0.8087	-0.76397	H	5.33718	1.12046	0.92733
H	5.95703	2.56644	-0.37192	H	4.92714	-1.35171	1.23555
H	6.87462	1.88147	0.98434	H	6.6025	-1.66872	0.75245
H	5.32283	2.68935	1.28337	H	5.26155	-2.32083	-0.21195
H	6.04483	-0.18249	2.16767	H	7.60522	-0.49733	-1.2444
H	4.45209	0.54374	2.46597	H	6.32254	-1.15489	-2.2801
H	4.5771	-1.01006	1.62001	H	6.61443	0.59718	-2.2301
atom	Con f. 4- 1m			atom	Con f. 4- 1n		
C	-5.96368	-1.68256	-0.34839	C	-5.33117	-1.86379	-1.54997
C	-4.85095	1.69346	-1.68745	C	-4.42648	2.29949	-1.48486
C	-4.00173	1.56437	-0.66148	C	-3.727	1.88196	-0.42278
C	-3.00645	0.41043	-0.62406	C	-2.93569	0.58056	-0.48738
C	-3.5691	-0.88586	0.07334	C	-3.70274	-0.69328	0.04097
C	-4.72388	-1.4075	-0.75388	C	-4.98683	-0.85493	-0.74787
C	-1.65134	0.84463	-0.03542	C	-1.5546	0.73462	0.17764
C	-0.5999	-0.27857	-0.10719	C	-0.67676	-0.51682	-0.01311
C	-1.12277	-1.54268	0.60332	C	-1.40927	-1.7648	0.51674
C	-2.46302	-1.97526	0.00716	C	-2.77406	-1.90975	-0.15826
C	-3.99668	2.57347	0.46131	C	-3.67201	2.71592	0.83441
C	-3.9826	-0.64137	1.52952	C	-4.10612	-0.57205	1.52601
C	0.73372	0.16002	0.45019	C	0.68243	-0.34662	0.62231
C	1.83077	0.08228	-0.34522	C	1.78537	-0.43933	-0.16363
C	0.77041	0.64172	1.8737	C	0.7327	-0.08396	2.10158
C	3.19727	0.43272	0.02721	C	3.17157	-0.31028	0.27313
C	4.27615	0.15474	-1.00691	C	4.2411	-0.59695	-0.76747
O	3.49626	0.95558	1.11672	O	3.49484	-0.01034	1.43737
C	5.66234	-0.17835	-0.41575	C	5.5689	0.16121	-0.55951
O	6.12366	0.92798	0.38337	O	6.12376	-0.17893	0.72596
C	5.60189	-1.4478	0.4441	C	6.59177	-0.29439	-1.59666
C	6.67914	-0.34086	-1.54224	C	5.35397	1.67807	-0.64504
H	-2.82411	0.11449	-1.66514	H	-2.76096	0.3734	-1.55052
H	-0.45692	-0.53417	-1.16447	H	-0.52954	-0.65768	-1.09101
H	-6.70185	-2.07851	-1.04129	H	-6.29489	-1.86786	-2.05334
H	-6.29743	-1.52774	0.67356	H	-4.68741	-2.71704	-1.74157
H	-5.57926	2.50027	-1.72568	H	-4.99903	3.2239	-1.46346
H	-4.84754	1.00033	-2.52436	H	-4.45277	1.72939	-2.41002
H	-4.46967	-1.59129	-1.79908	H	-5.69756	-0.0412	-0.61338
H	-1.28628	1.71612	-0.59075	H	-1.04667	1.60207	-0.25917
H	-1.77774	1.16511	1.00241	H	-1.67107	0.94461	1.24475
H	-0.39318	-2.35291	0.49318	H	-0.80374	-2.65596	0.31695
H	-1.22703	-1.35494	1.67726	H	-1.52615	-1.69979	1.60366
H	-2.30585	-2.24872	-1.04447	H	-2.6135	-2.05892	-1.23318
H	-2.8269	-2.87564	0.51556	H	-3.27682	-2.80972	0.21378
H	-4.83141	3.27283	0.3598	H	-3.92358	2.1399	1.72961
H	-4.06323	2.1025	1.44644	H	-2.66426	3.11664	0.99674
H	-3.06925	3.15899	0.45986	H	-4.36044	3.56314	0.76877
H	-4.26001	-1.5856	2.00963	H	-4.50623	-1.52863	1.87929
H	-3.17134	-0.20159	2.11373	H	-3.26297	-0.30514	2.16766
H	-4.84343	0.02851	1.59392	H	-4.88672	0.18068	1.66514
H	1.70565	-0.2969	-1.35574	H	1.64468	-0.64135	-1.22189
H	0.97814	1.71738	1.90775	H	-0.25137	-0.18214	2.56249
H	1.57578	0.15922	2.43296	H	1.42727	-0.7664	2.59896
H	-0.17635	0.46267	2.38565	H	1.10593	0.92677	2.29988
H	3.9507	-0.64487	-1.67864	H	4.44002	-1.67727	-0.71727
H	4.36443	1.06364	-1.61931	H	3.84659	-0.39922	-1.76831
H	5.36756	1.14007	0.9643	H	5.3796	-0.06688	1.34877
H	5.27266	-2.30939	-0.14618	H	6.75594	-1.37448	-1.52611
H	4.90899	-1.32122	1.28121	H	7.54804	0.21125	-1.42676
H	6.59321	-1.66767	0.85268	H	6.25315	-0.05945	-2.61009
H	7.67321	-0.53168	-1.12458	H	6.3061	2.19721	-0.49682
H	6.41201	-1.17898	-2.19273	H	4.95281	1.96486	-1.62278
H	6.72946	0.56889	-2.14918	H	4.65634	2.02032	0.12511
atom	Con f. 4- 1o			atom	Con f. 4- 1p		
C	-5.26213	-2.22534	-0.95583	C	5.29167	2.3002	-0.82401
C	-3.88972	1.8781	1.85334	C	5.2217	-1.90801	-0.08626
C	-4.09705	1.45069	0.60226	C	4.06404	-1.54118	0.47673

C	-3.14468	0.51163	-0.12527	C	3.16599	-0.51919	-0.21064
C	-3.33851	-1.01352	0.22177	C	3.38229	0.97487	0.2489
C	-4.78336	-1.39542	-0.02752	C	4.82675	1.36026	0.00059
C	-1.67467	0.94361	0.02351	C	1.68468	-0.93525	-0.13021
C	-0.73943	0.09168	-0.86774	C	0.76895	0.00423	-0.94702
C	-0.90904	-1.39206	-0.52956	C	0.95294	1.44384	-0.46336
C	-2.37374	-1.81565	-0.67653	C	2.42186	1.85817	-0.57575
C	-5.28657	1.91749	-0.19662	C	3.60612	-2.1692	1.77062
C	-3.0559	-1.31573	1.70853	C	3.12227	1.17482	1.75762
C	0.66616	0.64352	-0.79882	C	-0.64291	-0.53662	-0.93863
C	1.66027	-0.02894	-0.16607	C	-1.62838	0.07597	-0.23584
C	0.85947	1.9808	-1.46141	C	-0.85115	-1.79391	-1.7389
C	3.03934	0.42402	-0.0137	C	-3.01244	-0.37536	-0.12728
C	3.97695	-0.5053	0.74033	C	-3.92104	0.45539	0.76442
O	3.45271	1.5186	-0.43848	O	-3.44747	-1.39503	-0.69283
C	5.46197	-0.39448	0.33723	C	-5.42402	0.36733	0.43142
O	5.9251	0.95159	0.56114	O	-5.85839	-1.00431	0.51743
C	6.31066	-1.28814	1.23749	C	-6.23089	1.1321	1.4772
C	5.65975	-0.77236	-1.13683	C	-5.70919	0.91451	-0.97357
H	-3.3849	0.58701	-1.19416	H	3.44221	-0.52398	-1.27243
H	-1.07522	0.24033	-1.90559	H	1.11061	-0.04643	-1.99223
H	-6.32827	-2.42616	-1.02537	H	6.35934	2.49	-0.90286
H	-4.62931	-2.73585	-1.67579	H	4.64889	2.91507	-1.44679
H	-4.58877	2.55523	2.33826	H	5.87983	-2.63509	0.38384
H	-3.02281	1.58067	2.43483	H	5.55113	-1.49103	-1.03454
H	-5.48956	-0.93322	0.66227	H	5.54594	0.79315	0.58919
H	-1.59425	2.00152	-0.24394	H	1.59114	-1.96347	-0.49442
H	-1.3446	0.85571	1.06361	H	1.33787	-0.93681	0.90867
H	-0.28214	-2.00083	-1.1909	H	0.33175	2.12299	-1.05827
H	-0.56862	-1.58616	0.49318	H	0.61871	1.53864	0.57565
H	-2.671	-1.68342	-1.72422	H	2.71572	1.8116	-1.63178
H	-2.4763	-2.88349	-0.45191	H	2.53808	2.90174	-0.26213
H	-5.96528	2.53288	0.40119	H	4.39112	-2.8008	2.19589
H	-4.95557	2.51017	-1.06001	H	3.3235	-1.42326	2.51866
H	-5.8501	1.07026	-0.60366	H	2.72188	-2.79771	1.61023
H	-3.12168	-2.39503	1.88319	H	3.13857	2.24362	1.9973
H	-2.06247	-0.98759	2.02342	H	2.15426	0.77609	2.07075
H	-3.78767	-0.82637	2.35605	H	3.89586	0.69199	2.36108
H	1.44422	-0.99528	0.27497	H	-1.39796	0.983	0.31117
H	1.67371	1.94034	-2.1909	H	0.06917	-2.09228	-2.24628
H	1.14148	2.74829	-0.73314	H	-1.18677	-2.61993	-1.10377
H	-0.05304	2.30043	-1.96992	H	-1.63442	-1.65071	-2.48959
H	3.87943	-0.24681	1.80479	H	-3.76272	0.09138	1.79013
H	3.63453	-1.5392	0.63879	H	-3.5917	1.49874	0.75344
H	5.24951	1.51436	0.13594	H	-5.20953	-1.49659	-0.02147
H	6.17891	-1.011	2.28838	H	-6.04442	0.72781	2.47737
H	7.36987	-1.17807	0.98261	H	-7.3012	1.04319	1.26382
H	6.03502	-2.34011	1.11778	H	-5.96664	2.19379	1.47649
H	6.7178	-0.68697	-1.40393	H	-6.77926	0.83915	-1.19118
H	5.33765	-1.80178	-1.32567	H	-5.41364	1.96591	-1.05427
H	5.08822	-0.10894	-1.79265	H	-5.1658	0.34704	-1.73473
atom	Con f. 4- 1q			atom	Con f. 4- 1r		
C	-5.91297	-1.15262	-0.05454	C	-5.40546	-2.16866	-0.575
C	-4.14898	2.45528	-1.46865	C	-5.06439	2.05588	0.06472
C	-3.20681	2.04566	-0.61069	C	-3.89202	1.63053	0.55075
C	-2.81487	0.57429	-0.54503	C	-3.10263	0.55206	-0.18187
C	-3.58444	-0.27214	0.54308	C	-3.3581	-0.92136	0.32155
C	-5.07386	-0.17152	0.28163	C	-4.83491	-1.23471	0.18775
C	-1.28776	0.40618	-0.42259	C	-1.60078	0.89356	-0.22422
C	-0.8615	-1.05747	-0.54413	C	-0.79845	-0.10304	-1.09151
C	-1.58081	-1.89458	0.53852	C	-1.01683	-1.52387	-0.5683
C	-3.09712	-1.73124	0.42436	C	-2.5087	-1.86473	-0.55649
C	-2.48538	3.0391	0.26742	C	-3.31032	2.24814	1.79934
C	-3.35111	0.24728	1.9783	C	-2.99609	-1.11174	1.80996
C	0.62572	-1.32352	-0.49752	C	0.63566	0.36266	-1.20104
C	1.50061	-0.38394	-0.0592	C	1.64489	-0.31399	-0.5973
C	1.04084	-2.69794	-0.94853	C	0.84049	1.62445	-1.99479
C	2.94574	-0.54826	0.06667	C	3.05598	0.05876	-0.61169
C	3.71381	0.65871	0.5813	C	4.01824	-0.91266	0.05205
O	3.54104	-1.61018	-0.19107	O	3.48613	1.09407	-1.15253
C	5.19993	0.71109	0.17411	C	5.29715	-0.26845	0.62616
O	5.8723	-0.47232	0.64718	O	6.02095	0.39069	-0.43093
C	5.34893	0.81874	-1.3492	C	4.95661	0.7428	1.72863
C	5.88408	1.88866	0.86327	C	6.22601	-1.35413	1.16302
H	-3.11509	0.12837	-1.50167	H	-3.45859	0.55392	-1.21956

H	-1.21013	-1.43425	-1.5179	H	-1.21588	-0.04892	-2.10886
H	-6.97093	-0.95064	-0.2032	H	-6.48429	-2.30386	-0.57277
H	-5.59804	-2.1824	-0.19405	H	-4.84118	-2.8316	-1.22397
H	-4.44579	3.49942	-1.53512	H	-5.64535	2.8248	0.56869
H	-4.65775	1.75919	-2.13072	H	-5.48397	1.64828	-0.8516
H	-5.4798	0.83141	0.40273	H	-5.47926	-0.61562	0.80976
H	-0.80216	1.00046	-1.20498	H	-1.48677	1.90992	-0.61446
H	-0.949	0.81154	0.53562	H	-1.17288	0.89443	0.78392
H	-1.32197	-2.95342	0.4377	H	-0.48041	-2.24398	-1.1965
H	-1.2248	-1.57401	1.52477	H	-0.60604	-1.61907	0.44287
H	-3.40984	-2.13086	-0.54818	H	-2.88272	-1.82113	-1.58695
H	-3.5931	-2.3388	1.1901	H	-2.65045	-2.89587	-0.21352
H	-1.42668	3.11241	-0.0087	H	-2.40737	2.8261	1.56814
H	-2.92528	4.03556	0.1694	H	-4.02821	2.92694	2.26839
H	-2.51058	2.75726	1.32408	H	-3.01599	1.49756	2.53813
H	-3.79359	-0.44903	2.69901	H	-3.04931	-2.1744	2.07052
H	-2.28993	0.34737	2.21966	H	-1.98808	-0.75958	2.04246
H	-3.82492	1.22078	2.13088	H	-3.69641	-0.57987	2.45966
H	1.12557	0.59038	0.23206	H	1.41722	-1.2225	-0.05216
H	1.81812	-2.6388	-1.7157	H	1.18655	2.44251	-1.35382
H	0.18883	-3.25153	-1.34989	H	-0.08675	1.93515	-2.48199
H	1.47238	-3.27293	-0.12226	H	1.61367	1.48738	-2.7559
H	3.20364	1.57612	0.27198	H	3.49649	-1.4765	0.83079
H	3.64619	0.62197	1.67846	H	4.30673	-1.64006	-0.72054
H	5.30813	-1.20887	0.34254	H	5.34736	0.93466	-0.88305
H	4.89529	-0.04123	-1.85043	H	4.32718	1.54911	1.34078
H	6.40961	0.84955	-1.61781	H	5.87626	1.18719	2.12216
H	4.87007	1.72783	-1.7279	H	4.42463	0.2606	2.55551
H	6.94884	1.90509	0.60846	H	7.15379	-0.90492	1.53237
H	5.43991	2.83812	0.54995	H	5.7556	-1.89983	1.98638
H	5.7917	1.80346	1.95082	H	6.47972	-2.06787	0.37252
atom		Con f. 4- 1s		atom		Con f. 4- 1t	
C	-5.86526	-1.24187	0.31572	C	-5.86501	-1.24204	0.31649
C	-4.30167	2.30319	-1.51916	C	-4.30194	2.30288	-1.51923
C	-3.29387	1.97381	-0.70204	C	-3.29395	1.97367	-0.70227
C	-2.8605	0.51923	-0.56316	C	-2.8605	0.51912	-0.56322
C	-3.52197	-0.26235	0.63829	C	-3.52174	-0.26229	0.63846
C	-5.02878	-0.21632	0.48352	C	-5.02858	-0.2164	0.48393
C	-1.32473	0.39335	-0.54928	C	-1.32472	0.39329	-0.5496
C	-0.8696	-1.06588	-0.59848	C	-0.86955	-1.06593	-0.59862
C	-1.4776	-1.83323	0.59774	C	-1.47733	-1.83309	0.59784
C	-3.00229	-1.71471	0.59118	C	-3.00202	-1.71463	0.59153
C	-2.53887	3.04257	0.05084	C	-2.5388	3.0426	0.05023
C	-3.19991	0.36827	2.01019	C	-3.19951	0.36856	2.01023
C	0.62286	-1.29568	-0.66308	C	0.62291	-1.2957	-0.6634
C	1.50611	-0.29942	-0.40207	C	1.50615	-0.29937	-0.4026
C	1.03584	-2.69916	-1.01584	C	1.03588	-2.69922	-1.01604
C	2.9612	-0.41568	-0.42374	C	2.96125	-0.41556	-0.42438
C	3.74011	0.87299	-0.21458	C	3.7402	0.87308	-0.21531
O	3.56056	-1.48621	-0.63232	O	3.56064	-1.48609	-0.63292
C	5.16964	0.69206	0.33466	C	5.16936	0.69206	0.33498
O	5.92812	-0.14366	-0.56101	O	5.92831	-0.14401	-0.55992
C	5.88698	2.03892	0.3655	C	5.88686	2.03883	0.36596
C	5.14512	0.06348	1.73439	C	5.14377	0.06387	1.73487
H	-3.22013	-0.00063	-1.4601	H	-3.22027	-0.0009	-1.46001
H	-1.28625	-1.52213	-1.50977	H	-1.28634	-1.52234	-1.50976
H	-6.93649	-1.07552	0.2323	H	-6.93626	-1.07579	0.2332
H	-5.53511	-2.27461	0.25635	H	-5.53479	-2.27477	0.25731
H	-4.62687	3.33424	-1.63721	H	-4.6272	3.3339	-1.63739
H	-4.83731	1.55283	-2.09525	H	-4.83768	1.5524	-2.09505
H	-5.45026	0.78621	0.53574	H	-5.45011	0.78612	0.536
H	-0.91781	0.93906	-1.40811	H	-0.91797	0.93887	-1.40858
H	-0.92288	0.87442	0.34787	H	-0.92273	0.87451	0.34741
H	-1.19675	-2.89036	0.5576	H	-1.19646	-2.89022	0.55784
H	-1.05741	-1.42862	1.52612	H	-1.057	-1.42831	1.52607
H	-3.37808	-2.19598	-0.32019	H	-3.37795	-2.19608	-0.3197
H	-3.41903	-2.27238	1.43781	H	-3.4186	-2.27216	1.43832
H	-3.01139	4.01958	-0.08426	H	-3.01145	4.01956	-0.08486
H	-2.48147	2.83688	1.1236	H	-2.48105	2.83706	1.123
H	-1.5048	3.11945	-0.30595	H	-1.50485	3.11952	-0.3069
H	-3.57095	-0.28147	2.81019	H	-3.5705	-0.28103	2.81037
H	-2.12722	0.50874	2.16478	H	-2.12681	0.50902	2.16469
H	-3.68731	1.34004	2.1253	H	-3.68688	1.34037	2.12521
H	1.12993	0.68948	-0.16707	H	1.12994	0.68953	-0.16768
H	1.75804	-2.69896	-1.83693	H	1.53529	-3.18799	-0.17246

H	1.53532	-3.18799	-0.17233	H	0.17081	-3.30129	-1.3032
H	0.17079	-3.30122	-1.30304	H	1.75808	-2.69912	-1.83711
H	3.79608	1.36165	-1.19832	H	3.79702	1.36125	-1.19924
H	3.16786	1.54603	0.43126	H	3.16772	1.54657	0.42985
H	5.356	-0.92186	-0.70578	H	5.35601	-0.92198	-0.7052
H	5.92254	2.47822	-0.63667	H	5.92315	2.47787	-0.63629
H	6.91435	1.90842	0.72123	H	6.91399	1.90829	0.7224
H	5.37744	2.73905	1.03425	H	5.37698	2.7392	1.03421
H	6.16795	-0.06236	2.10325	H	6.16632	-0.06195	2.1045
H	4.59874	0.69713	2.44099	H	4.59691	0.69773	2.4409
H	4.66606	-0.91978	1.71533	H	4.66468	-0.91939	1.71572

atom	Con f. 4- 2a			atom	Con f. 4- 2b		
C	-4.01283	2.81509	0.9695	C	-3.52416	3.15657	-0.01229
C	-4.71036	-1.59337	0.6702	C	-4.56819	-0.9139	1.45689
C	-3.6546	-0.91866	1.141	C	-3.40526	-0.25614	1.53481
C	-2.53236	-0.40961	0.2472	C	-2.3812	-0.25142	0.40795
C	-2.81887	1.00476	-0.3863	C	-2.65028	0.84961	-0.68629
C	-2.9743	2.01263	0.7319	C	-2.58326	2.21134	-0.02917
C	-2.1314	-1.43381	-0.8285	C	-2.20604	-1.64105	-0.22808
C	-0.85837	-1.0152	-1.6036	C	-1.01751	-1.69269	-1.21969
C	-1.07872	0.38066	-2.2064	C	-1.21432	-0.60473	-2.28653
C	-1.54984	1.42055	-1.1813	C	-1.47318	0.78968	-1.69999
C	-3.50042	-0.64919	2.6155	C	-3.02424	0.50426	2.77821
C	-4.0424	0.99409	-1.3093	C	-3.98442	0.6405	-1.41118
C	0.39397	-1.21254	-0.7657	C	0.31234	-1.70302	-0.48495
C	1.29642	-0.21869	-0.573	C	1.29563	-0.81028	-0.76171
C	0.57906	-2.60334	-0.2218	C	0.46409	-2.79841	0.53518
C	2.5395	-0.30809	0.1896	C	2.60362	-0.73038	-0.11577
C	3.47634	0.88479	0.084	C	3.62267	0.19018	-0.76768
O	2.83192	-1.26992	0.9222	O	2.90813	-1.37535	0.90381
C	4.96886	0.55565	0.2936	C	4.65616	0.8019	0.20128
O	5.15802	-0.01835	1.6011	O	5.38766	-0.25224	0.85573
C	5.47131	-0.42135	-0.7775	C	5.68715	1.60866	-0.58318
C	5.78953	1.84218	0.2735	C	3.96746	1.68348	1.25165
H	-1.6552	-0.26461	0.893	H	-1.41564	0.01843	0.85766
H	-0.74887	-1.73274	-2.4306	H	-1.06712	-2.67394	-1.71496
H	-4.00058	3.51173	1.8039	H	-3.35184	4.10706	0.48622
H	-4.90853	2.8134	0.3555	H	-4.49338	3.02823	-0.48489
H	-5.49315	-1.94759	1.3366	H	-5.27724	-0.90161	2.28119
H	-4.83566	-1.81918	-0.3841	H	-4.85669	-1.48554	0.58043
H	-2.11022	2.07261	1.3959	H	-1.63435	2.41544	0.46984
H	-1.99443	-2.40878	-0.3553	H	-2.08234	-2.38128	0.56561
H	-2.93904	-1.55902	-1.555	H	-3.10948	-1.9272	-0.77375
H	-0.17054	0.73643	-2.7045	H	-0.3556	-0.56703	-2.96548
H	-1.83369	0.28095	-2.9939	H	-2.07031	-0.90227	-2.90198
H	-0.74906	1.61795	-0.4611	H	-0.57272	1.15022	-1.19221
H	-1.75071	2.37116	-1.6892	H	-1.67406	1.49705	-2.5131
H	-3.42407	0.42497	2.8152	H	-2.10079	0.09685	3.21061
H	-4.34198	-1.04647	3.1906	H	-3.80951	0.45844	3.53863
H	-2.57679	-1.10524	2.9956	H	-2.82524	1.55723	2.55188
H	-4.95483	0.74559	-0.7627	H	-4.83058	0.72389	-0.72555
H	-4.17747	1.97934	-1.7668	H	-4.1079	1.39334	-2.19631
H	-3.93312	0.26937	-2.1188	H	-4.03924	-0.34061	-1.88752
H	1.12919	0.74422	-1.0401	H	1.13971	-0.08167	-1.54674
H	-0.00871	-3.32577	-0.7938	H	0.2554	-2.41971	1.54336
H	1.62828	-2.90057	-0.2305	H	-0.23593	-3.61386	0.33622
H	0.24779	-2.65705	0.8225	H	1.48194	-3.18931	0.55501
H	3.33112	1.38394	-0.8786	H	4.15459	-0.41777	-1.51386
H	3.15899	1.59915	0.8575	H	3.10906	0.98202	-1.32066
H	4.48465	-0.72343	1.6565	H	4.69592	-0.86084	1.18002
H	6.53003	-0.64322	-0.6106	H	6.44536	2.00956	0.09757
H	5.36119	0.00263	-1.7812	H	5.21525	2.44656	-1.10491
H	4.9158	-1.36312	-0.741	H	6.18818	0.97566	-1.32262
H	5.44111	2.53226	1.0487	H	3.25525	1.10372	1.84633
H	6.84437	1.61685	0.4618	H	4.71473	2.10476	1.93147
H	5.7114	2.34108	-0.697	H	3.42587	2.50986	0.77925
atom	Con f. 4- 2c			atom	Con f. 4- 2d		
C	-4.38412	-1.19093	2.28584	C	-3.8026	-1.71976	2.33968
C	-3.23384	-2.05418	-2.00997	C	-3.25708	-1.77058	-2.17227
C	-2.48001	-1.88938	-0.91651	C	-2.36447	-1.73049	-1.17605
C	-2.04211	-0.52371	-0.40453	C	-1.9317	-0.43778	-0.49671
C	-3.09218	0.16619	0.5446	C	-2.86564	-0.00116	0.69282
C	-3.25567	-0.68729	1.78359	C	-2.79265	-1.05508	1.77633

C	-1.59849	0.41157	-1.549	C	-1.71593	0.70706	-1.50987
C	-1.04268	1.76741	-1.08604	C	-1.16622	2.00344	-0.89441
C	-2.06549	2.4364	-0.13347	C	-2.07778	2.42469	0.28486
C	-2.46709	1.50938	1.0138	C	-2.25621	1.29422	1.29855
C	-1.98602	-3.07583	-0.12935	C	-1.69825	-2.98832	-0.68082
C	-4.43496	0.41808	-0.14979	C	-4.30931	0.24886	0.24334
C	0.35487	1.76711	-0.49103	C	0.30064	2.00228	-0.49946
C	1.08114	0.62996	-0.35245	C	1.09348	0.91591	-0.6819
C	0.88292	3.12626	-0.11859	C	0.80878	3.30132	0.06318
C	2.42297	0.51671	0.21361	C	2.50465	0.79898	-0.32802
C	3.06396	-0.86002	0.1367	C	3.21975	-0.46117	-0.78742
O	3.01727	1.45511	0.77379	O	3.12939	1.6626	0.31529
C	4.60606	-0.86128	0.14285	C	4.26788	-0.9931	0.21477
O	5.08507	-0.22455	1.34331	O	5.29114	0.00078	0.41248
C	5.1217	-2.29697	0.19148	C	3.61471	-1.33799	1.55983
C	5.16061	-0.13141	-1.08776	C	4.96922	-2.21589	-0.37035
H	-1.17139	-0.69851	0.23631	H	-0.96638	-0.6457	-0.02302
H	-0.96339	2.41743	-1.97017	H	-1.24438	2.79668	-1.6531
H	-4.37443	-1.77721	3.20117	H	-3.62192	-2.44309	3.13074
H	-5.3525	-1.04672	1.8161	H	-4.83772	-1.57774	2.0436
H	-3.52379	-3.04635	-2.34749	H	-3.53802	-2.71282	-2.63663
H	-3.58862	-1.21939	-2.60638	H	-3.7386	-0.87908	-2.56148
H	-2.32203	-0.87645	2.31642	H	-1.77981	-1.25349	2.1314
H	-0.86422	-0.10993	-2.17137	H	-1.0613	0.35427	-2.31313
H	-2.45093	0.618	-2.20291	H	-2.6678	0.95532	-1.98816
H	-1.66646	3.36992	0.2724	H	-1.68084	3.30995	0.7886
H	-2.94683	2.71013	-0.72237	H	-3.04911	2.71605	-0.12773
H	-1.58097	1.28874	1.62324	H	-1.28022	1.04935	1.73781
H	-3.18088	2.01927	1.67111	H	-2.89505	1.63138	2.12301
H	-2.32029	-3.02489	0.91254	H	-2.05189	-3.87387	-1.21718
H	-0.8883	-3.09253	-0.10514	H	-0.60914	-2.92197	-0.80493
H	-2.3349	-4.02081	-0.55622	H	-1.88199	-3.13809	0.38861
H	-4.3148	1.00307	-1.06396	H	-4.77876	-0.66941	-0.11659
H	-4.92491	-0.51963	-0.42088	H	-4.90608	0.63299	1.07703
H	-5.10667	0.97444	0.51206	H	-4.36143	0.98219	-0.56408
H	0.66231	-0.30846	-0.69401	H	0.67385	0.02897	-1.13811
H	0.31524	3.91454	-0.6191	H	0.85736	3.25586	1.15819
H	0.80367	3.29108	0.96295	H	1.82041	3.51746	-0.28251
H	1.93974	3.22608	-0.37044	H	0.14712	4.12796	-0.20712
H	2.70438	-1.41736	1.01404	H	2.49333	-1.24351	-1.0229
H	2.68873	-1.39202	-0.7428	H	3.73307	-0.20829	-1.72578
H	4.59249	0.61775	1.38291	H	4.79984	0.83164	0.56411
H	6.21589	-2.30091	0.23302	H	2.83807	-2.10051	1.43838
H	4.8085	-2.85641	-0.69507	H	3.15718	-0.45311	2.01249
H	4.74136	-2.81147	1.07983	H	4.36999	-1.72177	2.2528
H	6.25487	-0.13905	-1.0624	H	5.43752	-1.96935	-1.3287
H	4.83336	-0.61584	-2.0137	H	5.74957	-2.56319	0.31489
H	4.8276	0.91042	-1.11028	H	4.26126	-3.0348	-0.52916

atom	Con f. 4- 2e			atom	Con f. 4- 2f		
C	-2.44064	3.16024	1.12369	C	-1.89386	3.34845	0.33958
C	-3.72947	-0.69103	2.48984	C	-3.31256	0.08131	2.73631
C	-3.75709	-0.85979	1.16218	C	-3.53477	-0.3774	1.49863
C	-2.61766	-0.3338	0.29872	C	-2.48122	-0.19763	0.41305
C	-2.85452	1.09382	-0.33089	C	-2.67887	1.06519	-0.51284
C	-3.14525	2.08	0.78258	C	-2.7276	2.30722	0.35456
C	-2.20974	-1.36107	-0.77364	C	-2.31231	-1.48416	-0.41626
C	-0.92275	-0.96081	-1.53264	C	-1.11171	-1.4191	-1.38973
C	-1.11693	0.44206	-2.12625	C	-1.26498	-0.17724	-2.28052
C	-1.56706	1.47851	-1.08939	C	-1.47964	1.11487	-1.48281
C	-4.88699	-1.61838	0.5093	C	-4.80439	-1.12562	1.17237
C	-4.06727	1.13622	-1.28553	C	-4.00394	1.03143	-1.30483
C	0.32109	-1.18507	-0.68859	C	0.21009	-1.58135	-0.65752
C	1.24699	-0.21239	-0.49684	C	1.2264	-0.69474	-0.80175
C	0.47338	-2.57932	-0.14381	C	0.31623	-2.81984	0.18961
C	2.48977	-0.3332	0.26097	C	2.53991	-0.76563	-0.165
C	3.47169	0.82127	0.13951	C	3.60457	0.16683	-0.71922
O	2.75625	-1.29741	1.00091	O	2.81316	-1.54543	0.76506
C	4.95627	0.40377	0.21868	C	4.69524	0.58576	0.28801
O	5.21478	-0.22324	1.48907	O	5.36493	-0.58648	0.79038
C	5.31472	-0.56329	-0.91758	C	5.76411	1.41188	-0.42254
C	5.84519	1.64327	0.16878	C	4.08986	1.37701	1.4548
H	-1.75815	-0.20024	0.96764	H	-1.53098	-0.01342	0.92979
H	-0.81868	-1.67528	-2.36319	H	-1.18083	-2.31238	-2.0287
H	-2.76694	3.80018	1.9399	H	-2.04834	4.19238	1.00746
H	-1.52614	3.45784	0.61911	H	-1.0356	3.4128	-0.32254

H	-4.52787	-1.06041	3.12929	H	-4.04591	-0.03889	3.53037
H	-2.9088	-0.17454	2.98139	H	-2.39037	0.59518	2.99613
H	-4.0523	1.86126	1.34384	H	-3.56443	2.32975	1.05076
H	-2.08912	-2.33945	-0.30151	H	-2.21145	-2.33522	0.26244
H	-3.00943	-1.47524	-1.51123	H	-3.21219	-1.67199	-1.00873
H	-0.20231	0.78414	-2.62196	H	-0.39997	-0.06667	-2.94323
H	-1.87389	0.36194	-2.91432	H	-2.12589	-0.34859	-2.93621
H	-0.76914	1.62687	-0.35594	H	-0.57962	1.33451	-0.90141
H	-1.71856	2.44458	-1.58467	H	-1.61424	1.95171	-2.17788
H	-5.3527	-1.05462	-0.30403	H	-5.32406	-0.70442	0.30708
H	-4.52876	-2.55709	0.07028	H	-4.59153	-2.17275	0.92654
H	-5.66187	-1.86632	1.2401	H	-5.49208	-1.11563	2.02272
H	-5.00824	1.02137	-0.74093	H	-4.86634	1.15798	-0.64494
H	-4.10035	2.10591	-1.79401	H	-4.02029	1.85558	-2.02619
H	-4.02417	0.35866	-2.05231	H	-4.13881	0.09991	-1.86022
H	1.10319	0.75347	-0.96533	H	1.09836	0.14709	-1.47013
H	1.51639	-2.89769	-0.14287	H	0.09037	-2.59112	1.23847
H	0.13137	-2.62621	0.89749	H	-0.39549	-3.57944	-0.1439
H	-0.12394	-3.28948	-0.72122	H	1.32511	-3.23348	0.17134
H	3.28521	1.3752	-0.78469	H	4.0804	-0.37163	-1.55176
H	3.25537	1.50735	0.97077	H	3.13126	1.05226	-1.15345
H	4.49389	-0.87449	1.59148	H	4.64041	-1.18395	1.05901
H	5.13968	-0.10461	-1.89649	H	6.5624	1.67433	0.27942
H	4.71842	-1.47866	-0.85699	H	5.34128	2.33713	-0.82527
H	6.37129	-0.84092	-0.85023	H	6.20414	0.84238	-1.24749
H	5.59584	2.3275	0.9861	H	3.35448	0.77617	1.99813
H	6.89631	1.35364	0.27003	H	4.87845	1.66627	2.1566
H	5.72374	2.1745	-0.77984	H	3.5936	2.28593	1.09879
atom		Con f. 4- 2g		atom		Con f. 4- 2h	
C	-3.52414	3.15657	-0.01199	C	3.03026	0.24999	3.11881
C	-4.56833	-0.91403	1.45664	C	2.02409	3.10341	0.07988
C	-3.40538	-0.25633	1.53471	C	2.45767	2.07999	-0.66656
C	-2.38125	-0.25147	0.40792	C	2.06882	0.65169	-0.30623
C	-2.65028	0.84968	-0.68623	C	3.11091	-0.12412	0.58816
C	-2.58325	2.21134	-0.02897	C	3.31286	0.64716	1.87704
C	-2.20605	-1.64104	-0.22823	C	1.67761	-0.15304	-1.56521
C	-1.0175	-1.69256	-1.21981	C	1.1504	-1.56563	-1.27475
C	-1.21427	-0.60452	-2.28656	C	2.19	-2.31072	-0.40299
C	-1.47315	0.78984	-1.69991	C	2.52393	-1.52483	0.86466
C	-3.02444	0.50392	2.77824	C	3.28953	2.33128	-1.90075
C	-3.98441	0.64069	-1.41117	C	4.49879	-0.24714	-0.07568
C	0.31232	-1.70292	-0.48502	C	-0.24807	-1.66208	-0.68831
C	1.2956	-0.81012	-0.76165	C	-1.00988	-0.56274	-0.46397
C	0.46407	-2.79841	0.535	C	-0.73405	-3.06237	-0.42941
C	2.6036	-0.73031	-0.11571	C	-2.3586	-0.53357	0.09726
C	3.62272	0.19016	-0.76765	C	-3.04413	0.82369	0.09696
O	2.90808	-1.3753	0.90385	O	-2.92144	-1.52443	0.5952
C	4.65625	0.80183	0.20131	C	-4.58378	0.78117	0.15937
O	5.38773	-0.25236	0.85572	O	-4.99972	0.0776	1.34629
C	5.68726	1.60855	-0.58314	C	-5.13505	2.19807	0.29277
C	3.9676	1.68342	1.2517	C	-5.16567	0.09269	-1.08235
H	-1.41573	0.01835	0.8577	H	1.18442	0.72909	0.33225
H	-1.06706	-2.67377	-1.71517	H	1.08782	-2.10719	-2.23069
H	-3.3518	4.10702	0.4866	H	3.23481	0.89697	3.96836
H	-4.49337	3.02829	-0.48459	H	2.59223	-0.71689	3.34774
H	-5.27742	-0.90183	2.2809	H	2.28397	4.13274	-0.1562
H	-4.8568	-1.48553	0.58008	H	1.39731	2.94582	0.95407
H	-1.63433	2.41539	0.47004	H	3.75283	1.63384	1.74136
H	-2.08234	-2.38133	0.5654	H	0.94378	0.418	-2.14429
H	-3.10947	-1.92716	-0.77395	H	2.55219	-0.26117	-2.21393
H	-0.35552	-0.56676	-2.96547	H	1.83269	-3.30875	-0.1357
H	-2.07024	-0.90201	-2.90208	H	3.09227	-2.45739	-1.00595
H	-0.5727	1.15034	-1.19208	H	1.61004	-1.4145	1.46138
H	-1.67401	1.49727	-2.51297	H	3.23212	-2.09544	1.47634
H	-2.10089	0.09661	3.21053	H	4.21227	1.74395	-1.90885
H	-3.80967	0.45785	3.53867	H	2.7395	2.05693	-2.80883
H	-2.82563	1.55697	2.55207	H	3.5572	3.38866	-1.98066
H	-4.83057	0.72402	-0.72554	H	5.00052	0.72312	-0.12485
H	-4.10785	1.39363	-2.19622	H	5.13464	-0.91519	0.51547
H	-4.03925	-0.34037	-1.88763	H	4.4455	-0.6475	-1.09104
H	1.13969	-0.08141	-1.5466	H	-0.61905	0.41251	-0.7271
H	-0.23594	-3.61384	0.33593	H	-1.79709	-3.16567	-0.64965
H	1.48192	-3.18929	0.55481	H	-0.16826	-3.78568	-1.02214
H	0.25534	-2.41982	1.54321	H	-0.60667	-3.32622	0.62785
H	4.15463	-0.41786	-1.51378	H	-2.66478	1.36025	0.97905

H	3.10919	0.98202	-1.32067	H	-2.71588	1.39754	-0.77547
H	4.69596	-0.86095	1.18	H	-4.49625	-0.75841	1.3198
H	6.44551	2.00939	0.0976	H	-6.22672	2.16996	0.37292
H	5.2154	2.44649	-1.10484	H	-4.86879	2.80527	-0.5775
H	6.18824	0.97555	-1.32261	H	-4.7361	2.68249	1.18987
H	3.2554	1.10367	1.84641	H	-6.25779	0.06583	-1.01428
H	4.71492	2.10467	1.93151	H	-4.88997	0.63017	-1.99574
H	3.42602	2.50982	0.77933	H	-4.80328	-0.93583	-1.16773
atom	Con f. 4- 2i			atom	Con f. 4- 2j		
C	-1.9597	-1.21415	3.03102	C	-3.77943	1.4601	-2.52909
C	-1.54592	-2.94791	-0.85838	C	-3.95879	1.30683	1.94489
C	-2.21203	-1.82224	-1.14444	C	-2.9129	1.47841	1.12762
C	-1.83068	-0.51541	-0.46152	C	-2.15359	0.31747	0.501
C	-2.67654	-0.14818	0.81709	C	-2.78076	-0.19343	-0.8514
C	-2.53084	-1.26938	1.82671	C	-2.75266	0.93659	-1.85774
C	-1.78287	0.64844	-1.47737	C	-1.93937	-0.84331	1.48844
C	-1.2755	1.97523	-0.89324	C	-0.97745	-1.92105	0.93133
C	-2.12208	2.31967	0.35588	C	-1.52874	-2.42446	-0.41201
C	-2.11209	1.17697	1.37151	C	-1.84248	-1.30055	-1.40822
C	-3.30502	-1.82141	-2.18531	C	-2.39776	2.85736	0.80531
C	-4.18513	-0.00436	0.525	C	-4.19904	-0.74424	-0.66454
C	0.21236	2.0597	-0.60032	C	0.46184	-1.42616	0.95006
C	1.05371	1.02939	-0.87093	C	1.23735	-1.45937	-0.15878
C	0.67965	3.3683	-0.02537	C	0.93421	-0.97045	2.30749
C	2.4799	0.96775	-0.57342	C	2.61989	-1.01716	-0.36211
C	3.23234	-0.2707	-1.02252	C	3.30915	-0.03061	0.56078
O	3.09587	1.83649	0.0732	O	3.21847	-1.4407	-1.3686
C	3.8715	-1.01819	0.17732	C	4.34432	0.88	-0.13851
O	4.8604	-0.17323	0.79241	O	5.38015	0.07884	-0.73546
C	2.8073	-1.4031	1.21363	C	3.67417	1.74974	-1.21079
C	4.61474	-2.25401	-0.3212	C	5.03322	1.75495	0.90576
H	-0.8208	-0.66177	-0.06872	H	-1.1589	0.69924	0.23153
H	-1.46025	2.7675	-1.63432	H	-0.99736	-2.75851	1.64422
H	-1.92352	-2.09374	3.66913	H	-3.62644	2.2666	-3.24177
H	-1.51126	-0.31117	3.43432	H	-4.80176	1.11664	-2.40193
H	-1.792	-3.89443	-1.33413	H	-4.47636	2.15794	2.38102
H	-0.73476	-2.96087	-0.13466	H	-4.3356	0.32439	2.21179
H	-2.95648	-2.21795	1.50307	H	-1.75477	1.33832	-2.04241
H	-1.1741	0.35099	-2.33812	H	-1.57032	-0.44177	2.43468
H	-2.78769	0.83013	-1.87097	H	-2.89205	-1.32996	1.71479
H	-1.76402	3.23712	0.82993	H	-0.84111	-3.14361	-0.87027
H	-3.14535	2.52406	0.02384	H	-2.44644	-2.98274	-0.1968
H	-1.07964	1.01152	1.70351	H	-0.91151	-0.82428	-1.73215
H	-2.68543	1.46671	2.25953	H	-2.30074	-1.72745	-2.30808
H	-4.23023	-1.36886	-1.81774	H	-2.99254	3.63638	1.29151
H	-3.00699	-1.24348	-3.06828	H	-2.40656	3.04145	-0.27424
H	-3.52945	-2.83984	-2.51467	H	-1.35521	2.96458	1.13316
H	-4.63935	-0.97328	0.30015	H	-4.88664	0.02933	-0.31511
H	-4.6962	0.39962	1.40583	H	-4.57967	-1.13461	-1.61376
H	-4.38831	0.66399	-0.31536	H	-4.22509	-1.56154	0.05905
H	0.65793	0.13496	-1.33378	H	0.83641	-1.90572	-1.06238
H	0.78859	3.29349	1.06387	H	0.77619	0.10623	2.4425
H	1.6578	3.65067	-0.416	H	0.36493	-1.47692	3.09189
H	-0.03943	4.16444	-0.23372	H	1.99136	-1.1769	2.47175
H	2.58058	-0.94972	-1.57644	H	2.56996	0.58979	1.06947
H	4.04131	0.04939	-1.68957	H	3.82628	-0.61343	1.33517
H	4.46989	0.7216	0.81032	H	4.89564	-0.59912	-1.24534
H	2.03183	-2.03586	0.76893	H	2.89307	2.38107	-0.77451
H	2.32427	-0.51377	1.63099	H	3.21922	1.13184	-1.99043
H	3.27146	-1.95357	2.03797	H	4.41957	2.39837	-1.68183
H	5.36368	-1.97639	-1.06961	H	4.31482	2.4173	1.39794
H	5.12654	-2.74339	0.51412	H	5.51536	1.1353	1.66875
H	3.92252	-2.9729	-0.76919	H	5.80036	2.37265	0.42741
atom	Con f. 4- 2k			atom	Con f. 4- 2l		
C	-4.01427	2.81346	0.9709	C	4.18033	0.0444	2.70631
C	-4.71056	-1.59308	0.6698	C	4.01488	1.97635	-1.34086
C	-3.65454	-0.91889	1.14074	C	3.05373	1.79782	-0.42656
C	-2.53237	-0.40957	0.24703	C	2.24349	0.51374	-0.31948
C	-2.81901	1.00496	-0.38625	C	2.92428	-0.59474	0.57131
C	-2.97486	2.01243	0.73232	C	3.06657	-0.06035	1.97993
C	-2.13135	-1.43345	-0.82903	C	1.84331	-0.04426	-1.6964
C	-0.8583	-1.01457	-1.60383	C	0.83349	-1.21102	-1.58288
C	-1.07864	0.38144	-2.20633	C	1.4607	-2.31057	-0.71173
C	-1.54993	1.42104	-1.18106	C	1.9413	-1.79729	0.6516
C	-3.49989	-0.65031	2.61535	C	2.68839	2.89474	0.53958

C	-4.04239	0.99425	-1.30949	C	4.27295	-1.05163	0.00434
C	0.39399	-1.21212	-0.76597	C	-0.54267	-0.70942	-1.16334
C	1.29655	-0.21836	-0.57308	C	-1.23446	-1.29879	-0.16269
C	0.57901	-2.60304	-0.22239	C	-1.07996	0.4127	-2.01594
C	2.53967	-0.30803	0.18953	C	-2.55383	-0.96409	0.38906
C	3.47657	0.88485	0.08423	C	-3.01827	0.46923	0.55551
O	2.83211	-1.27	0.92188	O	-3.24746	-1.88435	0.85628
C	4.96906	0.55556	0.29381	C	-4.53733	0.67883	0.36058
O	5.15813	-0.01874	1.60112	O	-5.25922	-0.09957	1.33309
C	5.47157	-0.4213	-0.77754	C	-4.89286	2.13776	0.63316
C	5.78977	1.84206	0.27393	C	-4.97406	0.26994	-1.05166
H	-1.6552	-0.26465	0.89289	H	1.31172	0.77153	0.20279
H	-0.74866	-1.73194	-2.43099	H	0.69603	-1.61051	-2.5985
H	-4.00237	3.50976	1.8056	H	4.1501	0.43483	3.72036
H	-4.91034	2.81094	0.35746	H	5.15672	-0.24671	2.33082
H	-5.49329	-1.94748	1.3362	H	4.57145	2.9088	-1.39703
H	-4.83616	-1.81832	-0.38467	H	4.28132	1.21126	-2.06322
H	-2.11048	2.07318	1.39583	H	2.12353	0.25446	2.43
H	-1.99438	-2.40856	-0.3561	H	1.43781	0.76637	-2.30573
H	-2.93895	-1.55849	-1.55552	H	2.72383	-0.41348	-2.22978
H	-0.17047	0.73737	-2.70436	H	0.76558	-3.14494	-0.56967
H	-1.83354	0.28189	-2.99388	H	2.31048	-2.72176	-1.26751
H	-0.74922	1.61835	-0.46074	H	1.07763	-1.49286	1.252
H	-1.75079	2.37176	-1.68871	H	2.42937	-2.61102	1.20069
H	-4.34175	-1.047	3.19052	H	1.63683	3.18428	0.41252
H	-3.4224	0.42368	2.81553	H	3.3093	3.78416	0.39684
H	-2.57665	-1.10748	2.99514	H	2.79781	2.56176	1.57722
H	-4.95485	0.74528	-0.76315	H	4.99358	-0.2313	-0.02644
H	-4.1777	1.97963	-1.76666	H	4.69149	-1.8494	0.62604
H	-3.93274	0.26981	-2.11918	H	4.17545	-1.44454	-1.00985
H	1.12941	0.74467	-1.03992	H	-0.82727	-2.1915	0.30105
H	0.24782	-2.65687	0.82206	H	-0.72586	0.29592	-3.04503
H	-0.00882	-3.32533	-0.79442	H	-2.16857	0.43659	-2.04035
H	1.6282	-2.90036	-0.23123	H	-0.72031	1.38786	-1.66728
H	3.33139	1.38443	-0.87819	H	-2.76969	0.7231	1.59597
H	3.15931	1.59888	0.85805	H	-2.45238	1.15465	-0.07234
H	4.48462	-0.72369	1.65638	H	-4.82793	-0.97609	1.32101
H	5.36172	0.00285	-1.78118	H	-5.97538	2.28001	0.55026
H	4.91596	-1.36303	-0.74131	H	-4.40243	2.80137	-0.08518
H	6.53023	-0.64332	-0.61043	H	-4.5845	2.42721	1.64284
H	5.71166	2.34117	-0.69648	H	-4.7478	-0.7836	-1.24334
H	5.44137	2.53199	1.04928	H	-6.05413	0.41032	-1.16024
H	6.84462	1.61671	0.46225	H	-4.47024	0.87444	-1.81289

atom	Con f. 4- 2m			atom	Con f. 4- 2n		
C	-3.80247	-1.71978	2.33971	C	-1.75557	3.35103	0.07363
C	-3.25747	-1.77025	-2.17224	C	-3.12259	0.33765	2.76234
C	-2.36468	-1.73032	-1.17617	C	-3.41287	-0.22194	1.58155
C	-1.93171	-0.43774	-0.49673	C	-2.4122	-0.15582	0.43493
C	-2.86554	-0.00114	0.69288	C	-2.63141	1.03397	-0.57823
C	-2.79253	-1.05513	1.77632	C	-2.61966	2.34115	0.18927
C	-1.71591	0.70717	-1.5098	C	-2.30942	-1.50725	-0.29441
C	-1.16611	2.0035	-0.89431	C	-1.14886	-1.54808	-1.31827
C	-2.07758	2.42473	0.28505	C	-1.31122	-0.37477	-2.29638
C	-2.25603	1.29418	1.29866	C	-1.47631	0.98208	-1.60068
C	-1.69858	-2.9883	-0.68111	C	-4.70996	-0.96874	1.38533
C	-4.30922	0.24893	0.24348	C	-3.99041	0.96436	-1.30771
C	0.30077	2.00228	-0.49946	C	0.19345	-1.68534	-0.62031
C	1.09357	0.9159	-0.682	C	1.21025	-0.81252	-0.83308
C	0.80898	3.30126	0.06323	C	0.31395	-2.87575	0.29156
C	2.50477	0.79898	-0.32823	C	2.52715	-0.84254	-0.20214
C	3.21984	-0.46121	-0.78757	C	3.56544	0.12053	-0.75496
O	3.12953	1.66262	0.31502	O	2.825	-1.60617	0.73452
C	4.26776	-0.99322	0.21478	C	4.57526	0.64533	0.28889
O	5.29111	0.00053	0.4126	O	5.29445	-0.46234	0.86307
C	3.6144	-1.33796	1.5598	C	5.62078	1.51992	-0.39782
C	4.96903	-2.21613	-0.37018	C	3.85962	1.42943	1.39665
H	-0.96639	-0.64581	-0.02311	H	-1.43366	0.04674	0.88776
H	-1.24431	2.79677	-1.65295	H	-1.26429	-2.48564	-1.88219
H	-3.62178	-2.44314	3.13074	H	-1.86667	4.24781	0.67826
H	-4.8376	-1.57769	2.04371	H	-0.91504	3.3386	-0.61355
H	-3.53858	-2.71242	-2.63662	H	-3.81827	0.30213	3.5976
H	-3.73901	-0.87867	-2.56127	H	-2.18027	0.85257	2.93191
H	-1.77967	-1.2536	2.13131	H	-3.43485	2.444	0.90367
H	-1.06132	0.3544	-2.31311	H	-2.19648	-2.30411	0.44542
H	-2.66779	0.95551	-1.98804	H	-3.23824	-1.71886	-0.83167

H	-1.68053	3.30992	0.78883	H	-0.46689	-0.33422	-2.99309
H	-3.0489	2.71619	-0.12746	H	-2.1967	-0.58076	-2.90796
H	-1.28004	1.04925	1.73788	H	-0.54943	1.23671	-1.07974
H	-2.89482	1.63131	2.12317	H	-1.63214	1.75978	-2.35736
H	-0.60947	-2.92204	-0.80522	H	-5.25888	-0.62629	0.50346
H	-1.88233	-3.13818	0.3883	H	-4.5297	-2.04084	1.24119
H	-2.05233	-3.87373	-1.21758	H	-5.35948	-0.85484	2.25773
H	-4.77874	-0.66933	-0.11641	H	-4.82208	1.14912	-0.62246
H	-4.90594	0.63311	1.07718	H	-4.02896	1.73593	-2.08436
H	-4.36136	0.98224	-0.56396	H	-4.15921	-0.00155	-1.79032
H	0.67389	0.02897	-1.13818	H	1.07177	-0.00506	-1.54027
H	0.85797	3.2556	1.15821	H	1.30921	-3.31895	0.24075
H	1.82048	3.51756	-0.28277	H	0.15771	-2.58012	1.33637
H	0.14715	4.1279	-0.20668	H	-0.43517	-3.63206	0.044
H	2.49339	-1.24349	-1.02317	H	4.11715	-0.42846	-1.53145
H	3.73331	-0.20837	-1.72585	H	3.07052	0.95813	-1.25414
H	4.79987	0.83145	0.5641	H	4.59933	-1.10495	1.10441
H	2.83775	-2.10046	1.43835	H	6.362	1.861	0.33246
H	3.15686	-0.45301	2.01232	H	5.15734	2.39957	-0.85448
H	4.36958	-1.7217	2.25289	H	6.14138	0.95605	-1.17858
H	5.43747	-1.96972	-1.3285	H	3.31858	2.28804	0.98511
H	5.74927	-2.56349	0.31517	H	3.14274	0.79487	1.92629
H	4.26099	-3.03497	-0.52903	H	4.59039	1.79761	2.12354
atom	Con f. 4- 2o			atom	Con f. 4- 2p		
C	2.00148	0.96487	3.14662	C	-3.78467	3.05372	0.42691
C	2.55423	2.8734	-0.64372	C	-4.67872	-1.26557	1.15387
C	2.98469	1.63401	-0.90879	C	-3.56483	-0.56584	1.4014
C	2.20986	0.42818	-0.39309	C	-2.48641	-0.3272	0.35391
C	2.72816	-0.17379	0.97126	C	-2.75738	0.929	-0.5589
C	2.7198	0.91692	2.02344	C	-2.79744	2.16373	0.31545
C	2.08293	-0.66072	-1.47344	C	-2.20474	-1.58102	-0.49178
C	1.10376	-1.78717	-1.06688	C	-0.97244	-1.41403	-1.4139
C	1.58595	-2.38272	0.26436	C	-1.16906	-0.16822	-2.29218
C	1.77357	-1.32379	1.35784	C	-1.52871	1.0931	-1.49628
C	4.20725	1.41361	-1.76586	C	-3.29684	0.00687	2.76906
C	4.17943	-0.69346	0.88583	C	-4.04035	0.78413	-1.38555
C	-0.34265	-1.31041	-1.12095	C	0.32423	-1.5024	-0.62529
C	-1.18846	-1.50592	-0.08279	C	1.2651	-0.53014	-0.67391
C	-0.73899	-0.68707	-2.4346	C	0.49297	-2.76981	0.17093
C	-2.58614	-1.10052	0.0908	C	2.55467	-0.51843	0.0296
C	-3.2162	0.02873	-0.70279	C	3.47275	0.61407	-0.40003
O	-3.24825	-1.67312	0.97605	O	2.87039	-1.3365	0.90114
C	-4.24947	0.85699	0.09621	C	4.86861	0.70417	0.2273
O	-5.32835	0.00942	0.52901	O	5.43955	1.86666	-0.42203
C	-3.59493	1.52785	1.31094	C	4.81122	0.95939	1.73507
C	-4.87519	1.9056	-0.8201	C	5.72602	-0.51942	-0.10456
H	1.19733	0.78575	-0.16795	H	-1.56172	-0.09384	0.89955
H	1.17757	-2.56343	-1.84287	H	-0.95595	-2.29542	-2.07218
H	2.09647	1.80488	3.83041	H	-3.6886	3.91446	1.0838
H	1.30057	0.18809	3.43725	H	-4.71864	2.9659	-0.11997
H	3.08505	3.75187	-1.00325	H	-5.42733	-1.42116	1.92707
H	1.65835	3.05077	-0.05384	H	-4.88653	-1.70937	0.18528
H	3.4011	1.74062	1.81582	H	-1.88884	2.32157	0.89898
H	1.77304	-0.19883	-2.41405	H	-2.07545	-2.43707	0.17498
H	3.05746	-1.11905	-1.6655	H	-3.06667	-1.81359	-1.12301
H	0.90216	-3.16262	0.6157	H	-0.27819	0.02015	-2.9011
H	2.53998	-2.88598	0.07165	H	-1.97594	-0.39065	-2.99901
H	0.80057	-0.89173	1.60723	H	-0.67468	1.39952	-0.88364
H	2.1452	-1.8036	2.27057	H	-1.72525	1.91976	-2.18903
H	4.69405	2.36436	-2.00074	H	-4.11443	-0.2045	3.46459
H	4.94305	0.76377	-1.28359	H	-2.37164	-0.41155	3.18694
H	3.94215	0.93067	-2.71398	H	-3.15609	1.092	2.72332
H	4.89092	0.12806	0.76715	H	-4.92312	0.71504	-0.74592
H	4.43675	-1.21774	1.81261	H	-4.16437	1.65019	-2.0436
H	4.3275	-1.39006	0.05685	H	-4.01913	-0.10652	-2.01693
H	-0.84085	-2.07036	0.77607	H	1.09563	0.33287	-1.3064
H	-0.14653	-1.12054	-3.24564	H	0.24427	-2.60343	1.22617
H	-1.7917	-0.83669	-2.67086	H	-0.16714	-3.55562	-0.20527
H	-0.54101	0.39167	-2.4382	H	1.52479	-3.12252	0.14607
H	-2.44533	0.69408	-1.09321	H	3.57797	0.55692	-1.49162
H	-3.72583	-0.41948	-1.56648	H	2.93568	1.55384	-0.21135
H	-4.88184	-0.75651	0.93884	H	6.32962	1.98523	-0.0569
H	-4.33889	2.12157	1.85123	H	5.82243	1.12157	2.12592
H	-2.7801	2.19203	1.00355	H	4.37186	0.11154	2.2645
H	-3.18841	0.78266	2.00091	H	4.21443	1.85251	1.94723

H	-5.35121	1.42812	-1.68268	H	5.77988	-0.66191	-1.18885
H	-5.63855	2.47036	-0.27491	H	6.7447	-0.37867	0.27439
H	-4.12077	2.60925	-1.18436	H	5.31515	-1.42514	0.34652
atom		Con f. 4- 2q		atom		Con f. 4- 2r	
C	-2.44063	3.16064	1.12316	C	-2.14646	-1.04776	3.05906
C	-3.73076	-0.6904	2.48966	C	-3.91041	-1.58543	-1.76454
C	-3.75775	-0.85946	1.16202	C	-2.80744	-1.6359	-1.00838
C	-2.61792	-0.33372	0.29893	C	-2.13292	-0.39886	-0.43278
C	-2.85447	1.09384	-0.33093	C	-2.79136	0.14953	0.89012
C	-3.1452	2.08028	0.78233	C	-2.8281	-0.96544	1.91513
C	-2.20982	-1.36121	-0.77316	C	-1.96225	0.71647	-1.48593
C	-0.92281	-0.96115	-1.53219	C	-1.12935	1.90634	-0.98182
C	-1.11692	0.44159	-2.1261	C	-1.77548	2.43845	0.32063
C	-1.56688	1.47822	-1.08934	C	-1.92576	1.33465	1.36941
C	-4.8872	-1.61841	0.50876	C	-2.12171	-2.94402	-0.70936
C	-4.06711	1.13625	-1.2857	C	-4.24989	0.6044	0.67941
C	0.32107	-1.18528	-0.68819	C	0.36373	1.6542	-0.80604
C	1.2471	-0.21264	-0.49686	C	0.93476	0.49901	-1.21878
C	0.47333	-2.57941	-0.14306	C	1.1293	2.81394	-0.22381
C	2.49003	-0.33339	0.26068	C	2.32372	0.04356	-1.11685
C	3.47194	0.82105	0.13876	C	3.27887	0.56566	-0.06003
O	2.75662	-1.2975	1.00072	O	2.69563	-0.86247	-1.88541
C	4.95652	0.40387	0.21868	C	4.22414	-0.51915	0.51063
O	5.21464	-0.22261	1.48942	O	5.04972	-1.05014	-0.54161
C	5.31554	-0.56358	-0.9171	C	5.17649	0.1198	1.51798
C	5.84529	1.64346	0.16864	C	3.42623	-1.6524	1.17004
H	-1.75862	-0.20018	0.96812	H	-1.13073	-0.71017	-0.12192
H	-0.81874	-1.67582	-2.36258	H	-1.19687	2.7131	-1.72679
H	-2.76698	3.80077	1.93921	H	-2.26688	-1.90401	3.7179
H	-1.52615	3.45817	0.61852	H	-1.45139	-0.28188	3.38991
H	-4.52947	-1.05963	3.1288	H	-4.35995	-2.4909	-2.16523
H	-2.9103	-0.17386	2.98148	H	-4.40039	-0.6502	-2.01679
H	-4.05225	1.86166	1.34364	H	-3.51341	-1.77591	1.66643
H	-2.08924	-2.33947	-0.30077	H	-1.52785	0.28902	-2.39502
H	-3.00945	-1.47559	-1.51079	H	-2.94364	1.10127	-1.7791
H	-0.20232	0.78352	-2.62194	H	-1.19444	3.26536	0.73622
H	-1.87396	0.36139	-2.91408	H	-2.75552	2.85367	0.06458
H	-0.76896	1.62644	-0.35586	H	-0.92753	0.96342	1.63288
H	-1.71814	2.44428	-1.5847	H	-2.35861	1.75112	2.28637
H	-5.35185	-1.05546	-0.30572	H	-1.97107	-3.07513	0.36821
H	-4.5287	-2.55772	0.07122	H	-1.12169	-2.96174	-1.16328
H	-5.66293	-1.86534	1.239	H	-2.68573	-3.80068	-1.08988
H	-5.00815	1.02146	-0.7412	H	-4.34691	1.34269	-0.1199
H	-4.10011	2.10591	-1.79424	H	-4.89289	-0.24284	0.4284
H	-4.02395	0.35865	-2.05245	H	-4.633	1.05608	1.60086
H	1.10328	0.75312	-0.96557	H	0.32432	-0.22992	-1.74116
H	1.5163	-2.89788	-0.14224	H	2.16532	2.84285	-0.55872
H	0.13153	-2.62597	0.89833	H	1.12757	2.78159	0.87243
H	-0.12418	-3.28967	-0.72014	H	0.65476	3.75629	-0.51355
H	3.28568	1.37435	-0.78587	H	3.8928	1.35029	-0.52206
H	3.25513	1.50771	0.96943	H	2.72549	1.02661	0.75886
H	4.49399	-0.87417	1.59156	H	4.42098	-1.25749	-1.25995
H	5.14083	-0.10525	-1.89622	H	5.75052	0.9244	1.04733
H	4.71932	-1.47899	-0.85644	H	5.87945	-0.63031	1.89492
H	6.37212	-0.84107	-0.84926	H	4.62683	0.53458	2.36795
H	5.5955	2.32801	0.98556	H	2.77537	-2.15059	0.44556
H	6.8964	1.35401	0.27047	H	4.11319	-2.39947	1.57989
H	5.7242	2.1743	-0.78025	H	2.80255	-1.27255	1.98616
atom		Con f. 4- 2s		atom		Con f. 4- 2t	
C	-1.96085	-1.21366	3.0312	C	-2.37002	3.23307	0.64146
C	-1.54581	-2.94807	-0.85799	C	-4.73718	-1.28747	1.09447
C	-2.21207	-1.82251	-1.14414	C	-3.59995	-0.64014	1.37571
C	-1.83082	-0.51553	-0.46144	C	-2.54909	-0.30862	0.32621
C	-2.67696	-0.14798	0.81691	C	-2.8495	0.99468	-0.50872
C	-2.53165	-1.26902	1.82673	C	-3.11363	2.14314	0.44397
C	-1.78269	0.64809	-1.47754	C	-2.24915	-1.50062	-0.59775
C	-1.2754	1.975	-0.89356	C	-1.01711	-1.24681	-1.50367
C	-2.12218	2.31969	0.35539	C	-1.21282	0.06956	-2.27452
C	-2.11246	1.17718	1.37121	C	-1.61251	1.25948	-1.39051
C	-3.30522	-1.82194	-2.18484	C	-3.25953	-0.22868	2.7846
C	-4.18543	-0.00395	0.52438	C	-4.11356	0.85547	-1.38191
C	0.21241	2.05948	-0.60042	C	0.27108	-1.39773	-0.71408
C	1.0538	1.02914	-0.8708	C	1.18249	-0.39797	-0.61809
C	0.67965	3.36817	-0.02562	C	0.46862	-2.74707	-0.07702
C	2.47997	0.9676	-0.57318	C	2.43758	-0.43646	0.12827

C	3.23256	-0.27074	-1.02234	C	3.32014	0.79583	0.0101
O	3.0958	1.83638	0.07351	O	2.7754	-1.38293	0.86158
C	3.87206	-1.01807	0.17741	C	4.82267	0.54845	0.2533
O	4.861	-0.17292	0.7922	O	5.0162	-0.00108	1.57076
C	2.80815	-1.40298	1.21397	C	5.39719	-0.41072	-0.79749
C	4.61533	-2.25384	-0.32119	C	5.575	1.87634	0.23556
H	-0.82104	-0.66183	-0.06836	H	-1.62285	-0.08398	0.87145
H	-1.46006	2.76716	-1.63476	H	-0.99212	-2.06882	-2.23392
H	-1.92496	-2.09315	3.66947	H	-2.67902	3.99501	1.35264
H	-1.51244	-0.31065	3.43447	H	-1.43834	3.41754	0.1152
H	-1.79182	-3.89468	-1.33361	H	-5.46264	-1.51336	1.87239
H	-0.73457	-2.96082	-0.13438	H	-4.98481	-1.62066	0.09137
H	-2.95731	-2.21759	1.50312	H	-4.04076	2.04486	1.00893
H	-1.1737	0.35044	-2.33807	H	-2.10169	-2.39543	0.01203
H	-2.78738	0.82977	-1.87147	H	-3.10789	-1.70883	-1.24163
H	-1.76414	3.2372	0.82932	H	-0.30803	0.31805	-2.84011
H	-3.14538	2.52408	0.0231	H	-1.99543	-0.10325	-3.02128
H	-1.08008	1.01169	1.70343	H	-0.77786	1.52516	-0.73576
H	-2.68593	1.46715	2.25907	H	-1.80151	2.13316	-2.02532
H	-3.00743	-1.24401	-3.06789	H	-3.00735	0.83653	2.83581
H	-3.52953	-2.84043	-2.5141	H	-4.07975	-0.42787	3.48052
H	-4.23043	-1.36952	-1.81714	H	-2.37168	-0.77257	3.13429
H	-4.63971	-0.97277	0.29922	H	-5.00258	0.69849	-0.76561
H	-4.69673	0.39992	1.40513	H	-4.26301	1.7743	-1.95898
H	-4.38827	0.6646	-0.3159	H	-4.0475	0.02648	-2.0905
H	0.65809	0.13464	-1.33357	H	0.99978	0.53346	-1.13879
H	-0.03905	4.16443	-0.23488	H	1.49749	-3.09252	-0.19145
H	0.78759	3.29381	1.06374	H	0.27715	-2.69949	1.00172
H	1.6582	3.65009	-0.41555	H	-0.21084	-3.48661	-0.50741
H	2.58081	-0.94984	-1.57617	H	3.16752	1.26499	-0.96657
H	4.0414	0.04947	-1.6895	H	2.95137	1.51174	0.75918
H	4.47016	0.72175	0.81043	H	4.38466	-0.74422	1.61918
H	3.27253	-1.95336	2.03825	H	4.89	-1.37942	-0.76331
H	2.03266	-2.03585	0.76946	H	6.46223	-0.57633	-0.6069
H	2.3251	-0.51367	1.63137	H	5.28641	-0.00152	-1.80719
H	3.9231	-2.9728	-0.76905	H	5.1765	2.55378	0.99773
H	5.36414	-1.97618	-1.06972	H	6.63658	1.70856	0.44498
H	5.12731	-2.74316	0.51406	H	5.48887	2.3625	-0.74081

atom	Con f. 4- 3a			atom	Con f. 4- 3b		
C	6.05468	-1.23102	-0.76398	C	6.1496	-0.69382	-0.86333
C	4.03859	2.21399	1.23962	C	3.72767	2.10177	1.64313
C	3.90273	1.75428	-0.00999	C	3.66075	1.85191	0.33002
C	2.9637	0.61219	-0.37666	C	2.86299	0.69269	-0.25317
C	3.59825	-0.81647	-0.18611	C	3.65733	-0.66704	-0.29042
C	4.79778	-0.93818	-1.10082	C	4.86956	-0.49293	-1.17984
C	1.60052	0.73047	0.32979	C	1.48759	0.53458	0.41974
C	0.60351	-0.3365	-0.16616	C	0.62695	-0.5352	-0.28283
C	1.19485	-1.74951	0.00711	C	1.37459	-1.88278	-0.32983
C	2.55656	-1.85699	-0.68267	C	2.747	-1.71728	-0.98561
C	4.66637	2.37496	-1.1513	C	4.36183	2.74002	-0.66504
C	3.97073	-1.09747	1.27403	C	4.04941	-1.15335	1.10922
C	-0.73824	-0.20243	0.51431	C	-0.73274	-0.66483	0.36111
C	-1.82947	0.07781	-0.24274	C	-1.83398	-0.37333	-0.37687
C	-0.78681	-0.39711	2.00464	C	-0.78484	-1.10615	1.7976
C	-3.19781	0.23738	0.23813	C	-3.22042	-0.44173	0.07273
C	-4.24469	0.59923	-0.80288	C	-4.28424	-0.10156	-0.95829
O	-3.51936	0.11816	1.43492	O	-3.54905	-0.78507	1.22323
C	-5.67555	0.12293	-0.47694	C	-5.6017	0.4493	-0.3746
O	-6.09865	0.69217	0.77697	O	-6.17225	-0.51899	0.52668
C	-6.64813	0.64722	-1.52974	C	-5.36286	1.77081	0.36788
C	-5.73994	-1.40822	-0.39925	C	-6.62248	0.64022	-1.49308
H	2.76782	0.69751	-1.4544	H	2.66941	0.93954	-1.30622
H	0.45508	-0.17209	-1.24058	H	0.47872	-0.20749	-1.31922
H	6.83186	-1.30571	-1.52033	H	6.9357	-0.54913	-1.60006
H	6.36265	-1.40563	0.26259	H	6.46846	-1.00436	0.12718
H	4.71593	3.03424	1.46493	H	4.30391	2.94052	2.02626
H	3.48808	1.79944	2.07826	H	3.21725	1.49036	2.38053
H	4.56837	-0.7765	-2.15545	H	4.63036	-0.18072	-2.19782
H	1.72549	0.64612	1.41276	H	1.60692	0.28152	1.47679
H	1.18978	1.72762	0.13856	H	0.96678	1.49757	0.38569
H	1.29209	-1.98921	1.07093	H	1.48448	-2.28836	0.68114
H	0.50704	-2.48763	-0.42082	H	0.78205	-2.60904	-0.8977
H	2.41735	-1.72186	-1.76329	H	2.60348	-1.41489	-2.03132

H	2.96824	-2.86322	-0.54221	H	3.27077	-2.68002	-1.00564
H	5.31258	3.18963	-0.81114	H	3.63864	3.18622	-1.36032
H	5.28965	1.63201	-1.66075	H	4.90809	3.54924	-0.17144
H	3.97573	2.77495	-1.90534	H	5.07051	2.16908	-1.27478
H	3.11293	-0.98464	1.94016	H	3.17886	-1.26897	1.75817
H	4.7516	-0.41888	1.62411	H	4.73482	-0.45686	1.59718
H	4.33787	-2.12333	1.38057	H	4.5432	-2.12833	1.0453
H	-1.69224	0.19904	-1.31377	H	-1.69128	-0.05735	-1.40665
H	0.20175	-0.601	2.41833	H	-1.39386	-2.00882	1.90627
H	-1.45162	-1.2264	2.26633	H	0.21126	-1.30636	2.19445
H	-1.19765	0.48812	2.49988	H	-1.26085	-0.34244	2.421
H	-4.24467	1.69665	-0.87321	H	-3.87531	0.59724	-1.69416
H	-3.93537	0.2251	-1.78318	H	-4.49966	-1.03412	-1.49993
H	-5.3593	0.50699	1.38794	H	-5.43431	-0.76251	1.11825
H	-7.66998	0.34333	-1.27984	H	-6.30794	2.14555	0.77342
H	-6.61492	1.74064	-1.57265	H	-4.66576	1.63318	1.19968
H	-6.40388	0.2526	-2.5205	H	-4.94941	2.53069	-0.30359
H	-5.44533	-1.8638	-1.35054	H	-6.27045	1.37568	-2.22259
H	-5.07745	-1.79039	0.38305	H	-6.80474	-0.30589	-2.01285
H	-6.76111	-1.7259	-0.16605	H	-7.57178	0.99385	-1.0774
atom		Con f. 4- 3c		atom		Con f. 4- 3d	
C	5.69944	-1.99255	0.04632	C	6.05025	-0.14528	0.87557
C	3.84426	1.64541	1.94731	C	2.75801	2.84585	0.21656
C	4.07893	1.36066	0.66083	C	3.261	1.91093	-0.59868
C	3.12077	0.54199	-0.19472	C	2.79051	0.4622	-0.5861
C	3.34065	-1.01337	-0.08188	C	3.54321	-0.43764	0.46677
C	4.7258	-1.34392	-0.59434	C	5.0124	-0.47975	0.10725
C	1.65095	0.93328	0.04076	C	1.26183	0.3454	-0.44207
C	0.6953	0.20919	-0.93736	C	0.78577	-1.09888	-0.61757
C	0.87547	-1.30526	-0.80835	C	1.48614	-2.00874	0.41849
C	2.33526	-1.69683	-1.05064	C	3.00707	-1.88755	0.30839
C	5.31498	1.87089	-0.03389	C	4.31876	2.25793	-1.61428
C	3.12842	-1.52726	1.34663	C	3.32841	0.04614	1.90549
C	-0.70838	0.73918	-0.75254	C	-0.70852	-1.31944	-0.56338
C	-1.68604	-0.0256	-0.20542	C	-1.54781	-0.37119	-0.07719
C	-0.91936	2.16092	-1.19855	C	-1.17241	-2.66091	-1.06341
C	-3.06048	0.39348	0.05224	C	-2.99611	-0.49629	0.05977
C	-3.98585	-0.66127	0.63743	C	-3.72121	0.71698	0.61957
O	-3.47472	1.54867	-0.15317	O	-3.62527	-1.53177	-0.2233
C	-5.48434	-0.45381	0.33714	C	-5.21527	0.81464	0.25314
O	-5.90682	0.82436	0.8515	O	-5.90397	-0.36284	0.71757
C	-5.7577	-0.52244	-1.17105	C	-5.85217	1.99208	0.98623
C	-6.30976	-1.50138	1.07936	C	-5.40195	0.96118	-1.26292
H	3.34204	0.78863	-1.24248	H	3.04931	0.03624	-1.56558
H	1.00152	0.50125	-1.95375	H	1.11082	-1.44374	-1.61133
H	6.65331	-2.18599	-0.43778	H	7.0689	-0.2337	0.5066
H	5.59375	-2.35329	1.06514	H	5.93498	0.22755	1.88892
H	4.54755	2.2369	2.52858	H	3.11296	3.87297	0.1794
H	2.95425	1.30576	2.46755	H	1.98124	2.63018	0.94335
H	4.90956	-1.01316	-1.61804	H	5.21072	-0.84546	-0.90157
H	1.34103	0.69901	1.06424	H	0.95125	0.72216	0.53668
H	1.55955	2.01749	-0.07289	H	0.78733	0.98862	-1.19088
H	0.56462	-1.63712	0.18824	H	1.1449	-1.72679	1.42138
H	0.23249	-1.82402	-1.52854	H	1.1956	-3.05342	0.26794
H	2.60692	-1.43055	-2.08068	H	3.31677	-2.26977	-0.67316
H	2.45145	-2.78346	-0.96685	H	3.48786	-2.52444	1.06005
H	5.91134	1.04529	-0.43714	H	3.96227	2.04473	-2.63061
H	5.04344	2.50705	-0.88655	H	4.59768	3.31455	-1.56268
H	5.94819	2.45378	0.64153	H	5.22238	1.65697	-1.46704
H	3.21233	-2.61848	1.37305	H	3.81501	-0.63624	2.6098
H	2.14003	-1.26308	1.72827	H	2.26859	0.08435	2.16567
H	3.86878	-1.11224	2.03393	H	3.74323	1.04487	2.05822
H	-1.46148	-1.04912	0.07166	H	-1.13894	0.57887	0.24708
H	-1.12535	2.81579	-0.34496	H	-1.60728	-3.25701	-0.25402
H	-0.0389	2.54089	-1.72203	H	-0.34389	-3.22209	-1.5019
H	-1.78748	2.24057	-1.85861	H	-1.95997	-2.54733	-1.81368
H	-3.66717	-1.65433	0.306	H	-3.62567	0.65509	1.71356
H	-3.83557	-0.63483	1.72657	H	-3.19577	1.62803	0.31664
H	-5.24727	1.45499	0.50407	H	-5.37181	-1.10537	0.3731
H	-6.82448	-0.37017	-1.36279	H	-6.92187	2.04175	0.75763
H	-5.20103	0.25203	-1.70676	H	-5.73643	1.8786	2.06894
H	-5.46867	-1.49664	-1.57928	H	-5.39091	2.93713	0.68444
H	-6.05837	-2.51059	0.73985	H	-4.91084	1.86695	-1.63379
H	-6.12707	-1.44128	2.15714	H	-4.98358	0.10232	-1.7957
H	-7.37689	-1.33309	0.90096	H	-6.46838	1.02428	-1.50136

atom	Con f. 4- 3e			atom	Con f. 4- 3f		
C	5.66519	-1.92049	0.15432	C	5.92406	-0.14449	1.222
C	3.4957	1.45985	2.222	C	2.81403	2.83934	-0.15596
C	3.88324	1.31744	0.94889	C	3.36459	1.78755	-0.77405
C	3.05033	0.58262	-0.09347	C	2.83843	0.36657	-0.61906
C	3.30773	-0.9708	-0.12788	C	3.45346	-0.4038	0.61105
C	4.75011	-1.21688	-0.51403	C	4.94665	-0.53818	0.40416
C	1.55085	0.91189	0.02045	C	1.29981	0.30873	-0.61115
C	0.73138	0.28357	-1.13186	C	0.78828	-1.13394	-0.64438
C	0.94879	-1.23136	-1.15505	C	1.34869	-1.9128	0.56843
C	2.43853	-1.56157	-1.27313	C	2.87687	-1.84698	0.59547
C	5.17874	1.91308	0.46163	C	4.53845	1.96252	-1.7028
C	2.95592	-1.64543	1.20292	C	3.12739	0.27803	1.94439
C	-0.70193	0.75704	-1.04584	C	-0.71118	-1.30782	-0.71921
C	-1.70482	-0.08787	-0.69685	C	-1.5576	-0.28155	-0.45161
C	-0.91248	2.21706	-1.34276	C	-1.17456	-2.69264	-1.08204
C	-3.11232	0.26809	-0.54878	C	-3.0158	-0.34612	-0.46604
C	-4.07147	-0.86989	-0.2405	C	-3.75191	0.9604	-0.2165
O	-3.54385	1.42767	-0.68554	O	-3.65446	-1.38973	-0.695
C	-5.31016	-0.46328	0.58521	C	-5.15411	0.80628	0.4077
O	-6.05801	0.53943	-0.12946	O	-5.98292	0.00683	-0.45818
C	-6.24488	-1.66001	0.73943	C	-5.06943	0.15165	1.79309
C	-4.89873	0.07956	1.96038	C	-5.83167	2.17077	0.5017
H	3.37468	0.95111	-1.07658	H	3.17099	-0.19199	-1.50519
H	1.13431	0.6979	-2.069	H	1.19673	-1.61677	-1.54558
H	6.67141	-2.0424	-0.23847	H	6.96683	-0.31532	0.96691
H	5.45824	-2.39796	1.10739	H	5.73532	0.35956	2.16517
H	4.11342	1.99348	2.94029	H	3.21199	3.84196	-0.29272
H	2.55989	1.05693	2.59592	H	1.95586	2.74715	0.50219
H	5.03594	-0.76741	-1.46652	H	5.22059	-1.03809	-0.52638
H	1.1419	0.55468	0.97107	H	0.91249	0.81743	0.27654
H	1.43486	1.99966	0.01873	H	0.92194	0.85952	-1.47908
H	0.54217	-1.6833	-0.24392	H	0.92449	-1.48832	1.4857
H	0.40663	-1.6768	-1.99696	H	1.03361	-2.96044	0.52979
H	2.81257	-1.17564	-2.23055	H	3.26335	-2.36853	-0.28989
H	2.58106	-2.64803	-1.29623	H	3.25853	-2.38685	1.4699
H	5.71523	2.42679	1.26484	H	5.3947	1.36296	-1.37634
H	5.8371	1.14201	0.04734	H	4.28334	1.62028	-2.7144
H	4.99276	2.63379	-0.34544	H	4.85474	3.00809	-1.76328
H	3.605	-1.29921	2.01015	H	2.05092	0.38713	2.09132
H	3.06696	-2.73125	1.11806	H	3.57098	1.27441	2.00244
H	1.92497	-1.44197	1.49983	H	3.51426	-0.31682	2.77797
H	-1.47373	-1.1295	-0.50798	H	-1.14476	0.69034	-0.20753
H	-1.7015	2.3554	-2.08719	H	-1.8777	-2.66121	-1.91921
H	0.00758	2.67824	-1.70932	H	-0.32946	-3.32996	-1.35183
H	-1.24085	2.75824	-0.4488	H	-1.71339	-3.16034	-0.25105
H	-4.40889	-1.26344	-1.21018	H	-3.13466	1.62058	0.39993
H	-3.53552	-1.68291	0.25745	H	-3.8523	1.45134	-1.19541
H	-5.3878	1.19953	-0.39307	H	-5.43711	-0.7783	-0.65766
H	-7.14395	-1.36465	1.2904	H	-6.07291	0.05119	2.21843
H	-6.55097	-2.0379	-0.24145	H	-4.62527	-0.84616	1.73059
H	-5.756	-2.47088	1.28753	H	-4.46296	0.7546	2.47717
H	-4.34624	-0.67355	2.53219	H	-5.26862	2.84337	1.15564
H	-4.26504	0.9657	1.85948	H	-5.9082	2.63116	-0.48858
H	-5.78965	0.36087	2.53052	H	-6.84214	2.06009	0.90882

atom	Con f. 4- 3g			atom	Con f. 4- 3h		
C	5.66488	-1.92076	0.15445	C	5.92394	-0.14453	1.2223
C	3.49535	1.45953	2.22234	C	2.81421	2.83932	-0.1564
C	3.88313	1.31718	0.94931	C	3.36478	1.78739	-0.77424
C	3.05029	0.58266	-0.09333	C	2.83849	0.36648	-0.61906
C	3.30756	-0.97077	-0.12811	C	3.45338	-0.4038	0.6112
C	4.75002	-1.21693	-0.51397	C	4.94657	-0.53825	0.40444
C	1.55083	0.91199	0.02052	C	1.29986	0.30877	-0.61123
C	0.73141	0.28402	-1.13206	C	0.78826	-1.13388	-0.64437
C	0.9488	-1.23091	-1.15575	C	1.3485	-1.91264	0.56857
C	2.43855	-1.5611	-1.27374	C	2.87668	-1.84694	0.59574
C	5.17885	1.91265	0.46241	C	4.53876	1.9621	-1.7029
C	2.95531	-1.6457	1.20241	C	3.12723	0.27819	1.94445
C	-0.70193	0.75745	-1.04596	C	-0.7112	-1.30772	-0.71937
C	-1.7048	-0.08761	-0.69726	C	-1.55762	-0.28143	-0.45182
C	-0.91248	2.21753	-1.34259	C	-1.17458	-2.69254	-1.08219
C	-3.11232	0.26819	-0.54901	C	-3.0158	-0.34597	-0.46627
C	-4.07127	-0.86993	-0.2406	C	-3.75191	0.96049	-0.2164
O	-3.54404	1.4277	-0.68575	O	-3.6545	-1.38952	-0.69551
C	-5.30985	-0.4635	0.58538	C	-5.15407	0.80623	0.40784

O	-6.05813	0.53892	-0.12923	O	-5.98291	0.00696	-0.45818
C	-6.24422	-1.66043	0.74007	C	-5.06938	0.1513	1.79308
C	-4.89817	0.0797	1.96033	C	-5.8316	2.17072	0.50215
H	3.37478	0.9514	-1.0763	H	3.17104	-0.19225	-1.50509
H	1.1344	0.69864	-2.06904	H	1.19679	-1.6168	-1.54547
H	6.67118	-2.04269	-0.23814	H	6.96672	-0.31541	0.96728
H	5.45767	-2.39841	1.10738	H	5.73516	0.3596	2.16542
H	4.11303	1.99294	2.94084	H	3.21222	3.8419	-0.2933
H	2.55936	1.05679	2.59602	H	1.95596	2.74728	0.50167
H	5.03614	-0.76726	-1.46628	H	5.22057	-1.03826	-0.52604
H	1.14176	0.55457	0.97101	H	0.91254	0.81757	0.27642
H	1.43492	1.99976	0.01906	H	0.92207	0.85952	-1.4792
H	0.54206	-1.68319	-0.24484	H	0.92427	-1.48801	1.48576
H	0.40674	-1.67602	-1.99789	H	1.03335	-2.96026	0.53005
H	2.81275	-1.17479	-2.23095	H	3.26321	-2.36862	-0.28952
H	2.58107	-2.64755	-1.29724	H	3.25822	-2.38673	1.47027
H	5.71536	2.42599	1.26584	H	4.28381	1.6195	-2.71441
H	5.83709	1.14156	0.04798	H	4.85503	3.00766	-1.76367
H	4.99315	2.63366	-0.34446	H	5.39497	1.36266	-1.37609
H	3.60409	-1.29964	2.00994	H	3.51402	-0.3166	2.77811
H	3.06639	-2.73151	1.11736	H	2.05076	0.38735	2.09128
H	1.92424	-1.44234	1.499	H	3.57086	1.27455	2.00243
H	-1.47362	-1.12927	-0.50864	H	-1.14475	0.69044	-0.20768
H	0.00761	2.67877	-1.70897	H	-1.8777	-2.66112	-1.91938
H	-1.24087	2.75853	-0.44853	H	-0.32949	-3.32989	-1.3519
H	-1.70146	2.35603	-2.08703	H	-1.71349	-3.16019	-0.25121
H	-4.40882	-1.26349	-1.21025	H	-3.1346	1.62052	0.40014
H	-3.53512	-1.68289	0.25721	H	-3.85238	1.45165	-1.19518
H	-5.38815	1.1992	-0.39299	H	-5.43713	-0.77815	-0.65783
H	-7.14316	-1.36522	1.29134	H	-6.07286	0.05077	2.21842
H	-6.5506	-2.03853	-0.24064	H	-4.62522	-0.84649	1.73034
H	-5.75496	-2.47113	1.2881	H	-4.46289	0.75408	2.47729
H	-4.34529	-0.67315	2.53211	H	-5.26853	2.84315	1.15625
H	-4.26479	0.96602	1.85912	H	-5.90811	2.63135	-0.48802
H	-5.78903	0.36081	2.53069	H	-6.84207	2.05998	0.90925
atom	Con f. 4- 3i			atom	Con f. 4- 3j		
C	5.69741	-1.99113	0.04433	C	6.04999	-0.14468	0.87605
C	3.826	1.62729	1.97274	C	2.75816	2.84581	0.21602
C	4.07068	1.3549	0.68546	C	3.26132	1.91068	-0.59886
C	3.1189	0.54525	-0.18562	C	2.79065	0.46201	-0.58617
C	3.33928	-1.01116	-0.08909	C	3.54309	-0.43774	0.46694
C	4.72805	-1.33543	-0.59564	C	5.01235	-0.47989	0.10776
C	1.6471	0.9323	0.04469	C	1.26193	0.34542	-0.4423
C	0.69904	0.21884	-0.94847	C	0.78578	-1.09885	-0.61772
C	0.87943	-1.29684	-0.83513	C	1.48596	-2.00871	0.41851
C	2.34111	-1.68428	-1.07252	C	3.00691	-1.88764	0.30863
C	5.31234	1.87147	0.00548	C	4.31924	2.25732	-1.61441
C	3.11743	-1.54127	1.33206	C	3.32801	0.04618	1.90558
C	-0.70625	0.74583	-0.76781	C	-0.7085	-1.31936	-0.56355
C	-1.68377	-0.0213	-0.2237	C	-1.54773	-0.37106	-0.0773
C	-0.91857	2.1677	-1.21264	C	-1.17245	-2.66079	-1.0636
C	-3.05942	0.39517	0.03141	C	-2.99601	-0.49608	0.05968
C	-3.97565	-0.65306	0.64219	C	-3.72112	0.71728	0.6193
O	-3.48108	1.54368	-0.19538	O	-3.62519	-1.5316	-0.22324
C	-5.47769	-0.45703	0.35265	C	-5.21528	0.81463	0.25322
O	-5.90022	0.82944	0.84571	O	-5.90372	-0.36285	0.71803
C	-5.76564	-0.55663	-1.15112	C	-5.85217	1.99214	0.9862
C	-6.29202	-1.49285	1.12318	C	-5.40235	0.96086	-1.26282
H	3.34688	0.80413	-1.229	H	3.04952	0.03589	-1.56557
H	1.0123	0.52238	-1.95929	H	1.11088	-1.44381	-1.61143
H	6.65462	-2.17896	-0.43538	H	7.06872	-0.23313	0.50731
H	5.58486	-2.3632	1.05833	H	5.93445	0.22882	1.88912
H	4.525	2.21277	2.56517	H	3.11322	3.87289	0.17876
H	2.93166	1.28341	2.48266	H	1.9811	2.63036	0.94257
H	4.9187	-0.99318	-1.61428	H	5.21095	-0.84619	-0.90079
H	1.33048	0.6851	1.06311	H	0.95127	0.72231	0.53637
H	1.55504	2.01774	-0.05638	H	0.78758	0.98859	-1.19124
H	0.56313	-1.64012	0.15581	H	1.1446	-1.72666	1.42133
H	0.24114	-1.80797	-1.56489	H	1.19533	-3.05337	0.26797
H	2.61936	-1.40585	-2.09757	H	3.31673	-2.27001	-0.67283
H	2.45788	-2.77168	-1.00054	H	3.48753	-2.52448	1.06044
H	5.91136	1.04977	-0.40172	H	3.96235	2.04501	-2.6308
H	5.04754	2.51653	-0.8426	H	4.59907	3.31368	-1.56232
H	5.94064	2.44703	0.6917	H	5.22235	1.65549	-1.46768
H	3.20078	-2.63275	1.3467	H	3.74266	1.04499	2.05825

H	2.12664	-1.28098	1.71016	H	3.81463	-0.63605	2.61002
H	3.85339	-1.13441	2.02891	H	2.26816	0.08427	2.16561
H	-1.45672	-1.04339	0.05659	H	-1.13877	0.57898	0.24693
H	-0.02718	2.55968	-1.70793	H	-0.34398	-3.22197	-1.50218
H	-1.76614	2.24023	-1.90005	H	-1.96007	-2.54712	-1.81382
H	-1.15953	2.81621	-0.36364	H	-1.60736	-3.2569	-0.25425
H	-3.65572	-1.65024	0.32447	H	-3.62527	0.65578	1.71328
H	-3.81488	-0.60808	1.72925	H	-3.19588	1.62829	0.3159
H	-5.24722	1.4555	0.47825	H	-5.37157	-1.10542	0.37359
H	-6.83482	-0.41177	-1.33511	H	-6.92192	2.04168	0.75779
H	-5.2171	0.20874	-1.70793	H	-5.73623	1.87885	2.06891
H	-5.47744	-1.53788	-1.54276	H	-5.39106	2.93718	0.68416
H	-6.03854	-2.50773	0.80272	H	-4.91145	1.86662	-1.63401
H	-6.10093	-1.40943	2.19794	H	-4.98406	0.10195	-1.79557
H	-7.36135	-1.33323	0.95003	H	-6.46885	1.02382	-1.50101
atom	Con f. 4- 3k			atom	Con f. 4- 3l		
C	-5.14815	2.46187	-0.95359	C	6.15244	-0.70579	-0.92333
C	-4.73636	-1.67577	-1.88432	C	4.31581	2.63581	-0.78307
C	-3.9734	-1.5941	-0.7875	C	3.58656	1.92537	0.08527
C	-3.03371	-0.41005	-0.59189	C	2.83624	0.68233	-0.37736
C	-3.63215	0.78558	0.24714	C	3.68127	-0.64344	-0.26574
C	-4.90667	1.26375	-0.41887	C	4.86096	-0.53634	-1.20745
C	-1.66736	-0.86542	-0.04449	C	1.46132	0.55461	0.30291
C	-0.65128	0.29106	0.00626	C	0.6455	-0.62595	-0.25958
C	-1.21807	1.45914	0.83725	C	1.44312	-1.93558	-0.11545
C	-2.57039	1.90492	0.27942	C	2.80384	-1.80835	-0.80277
C	-3.99583	-2.6947	0.24524	C	3.47325	2.35765	1.527
C	-4.01044	0.37446	1.68656	C	4.13122	-0.93995	1.16981
C	0.69401	-0.1627	0.52033	C	-0.71779	-0.70712	0.385
C	1.77949	-0.02624	-0.28332	C	-1.81436	-0.48178	-0.38211
C	0.75575	-0.73133	1.91074	C	-0.77811	-1.01941	1.85442
C	3.1522	-0.38961	0.05027	C	-3.20307	-0.50234	0.0651
C	4.20309	-0.13499	-1.0179	C	-4.25339	-0.14528	-0.97412
O	3.47695	-0.90546	1.13591	O	-3.54354	-0.81937	1.21952
C	5.6116	0.18141	-0.47219	C	-5.55978	0.44243	-0.40182
O	6.07937	-0.92496	0.32317	O	-6.15727	-0.50331	0.50589
C	6.59632	0.31756	-1.63032	C	-5.29304	1.7651	0.32913
C	5.59725	1.45948	0.37674	C	-6.57075	0.64713	-1.52677
H	-2.85678	0.01612	-1.58729	H	2.64883	0.80722	-1.45168
H	-0.5151	0.65658	-1.01913	H	0.4997	-0.4449	-1.33176
H	-6.11655	2.68814	-1.39308	H	6.90944	-0.62603	-1.69944
H	-4.41224	3.25996	-0.97928	H	6.51097	-0.92589	0.07791
H	-5.41473	-2.5102	-2.04683	H	4.87325	3.5166	-0.47281
H	-4.71101	-0.90886	-2.65429	H	4.38213	2.36141	-1.83242
H	-5.70533	0.52406	-0.43561	H	4.58298	-0.3172	-2.23963
H	-1.27622	-1.66172	-0.68806	H	1.58087	0.43678	1.38383
H	-1.78701	-1.2991	0.95246	H	0.90231	1.48448	0.14832
H	-1.31877	1.16043	1.88603	H	1.57549	-2.18139	0.94348
H	-0.51499	2.29935	0.81374	H	0.88018	-2.76146	-0.56504
H	-2.41813	2.27782	-0.74096	H	2.6405	-1.65525	-1.87764
H	-2.95371	2.7458	0.86871	H	3.36457	-2.74468	-0.70077
H	-3.03865	-3.2291	0.27181	H	3.70264	1.54654	2.22422
H	-4.16605	-2.31438	1.25659	H	4.14898	3.19148	1.73729
H	-4.77828	-3.42416	0.01814	H	2.45349	2.68759	1.75997
H	-3.18944	-0.12174	2.21021	H	3.29005	-0.9468	1.86623
H	-4.87116	-0.29969	1.69469	H	4.85396	-0.20066	1.52319
H	-4.28555	1.26419	2.26343	H	4.61032	-1.92323	1.22215
H	1.63601	0.40201	-1.27142	H	-1.66531	-0.24697	-1.43266
H	-0.2204	-0.71676	2.39713	H	-1.24178	-1.99826	2.02041
H	1.46471	-0.17271	2.5291	H	-1.40155	-0.29522	2.3855
H	1.11993	-1.76368	1.89096	H	0.21519	-1.0317	2.30577
H	4.25917	-1.04966	-1.62551	H	-3.82328	0.53754	-1.71298
H	3.86888	0.664	-1.68603	H	-4.48824	-1.07652	-1.50996
H	5.33699	-1.12087	0.92721	H	-5.42879	-0.75565	1.10533
H	7.60558	0.49593	-1.24476	H	-6.23083	2.16552	0.72698
H	6.61404	-0.598	-2.23035	H	-4.60268	1.61962	1.16518
H	6.32303	1.15422	-2.28018	H	-4.85952	2.50878	-0.3478
H	6.60401	1.66738	0.75251	H	-6.19787	1.36653	-2.26191
H	5.26339	2.32062	-0.2116	H	-6.77387	-0.29933	-2.0381
H	4.92847	1.35146	1.23576	H	-7.51295	1.02779	-1.11889
atom	Con f. 4- 3m			atom	Con f. 4- 3n		
C	5.28763	-1.94217	-1.55275	C	5.32706	-1.85021	-1.56822
C	4.50603	2.24204	-1.48039	C	4.41837	2.30796	-1.47466
C	3.79755	1.84657	-0.4158	C	3.7198	1.88676	-0.4134
C	2.96532	0.57128	-0.4798	C	2.93012	0.58466	-0.48172

C	3.69317	-0.72901	0.04017	C	3.70211	-0.69139	0.03482
C	4.97263	-0.92575	-0.74808	C	4.98327	-0.8458	-0.76024
C	1.59176	0.76332	0.19042	C	1.55159	0.73114	0.18999
C	0.67532	-0.45977	-0.01252	C	0.67625	-0.52071	-0.00798
C	1.36785	-1.73331	0.50709	C	1.41249	-1.77076	0.51241
C	2.7278	-1.91505	-0.16883	C	2.77524	-1.90863	-0.16809
C	3.77606	2.67892	0.84322	C	3.66518	2.71676	0.8464
C	4.0985	-0.63009	1.52634	C	4.11184	-0.57863	1.51871
C	-0.67975	-0.23859	0.61733	C	-0.68326	-0.35731	0.62849
C	-1.76044	-0.10931	-0.19376	C	-1.78552	-0.44578	-0.1589
C	-0.74641	-0.16811	2.11698	C	-0.73421	-0.10451	2.10934
C	-3.1377	0.12677	0.22774	C	-3.17206	-0.31863	0.27671
C	-4.13479	0.45165	-0.872	C	-4.24104	-0.60226	-0.76539
O	-3.50053	0.10283	1.41819	O	-3.4971	-0.02117	1.44116
C	-5.60124	0.09528	-0.55188	C	-5.56307	0.16757	-0.56338
O	-6.01999	0.79602	0.63547	O	-6.12378	-0.16381	0.72198
C	-6.5099	0.58592	-1.67605	C	-5.33554	1.68227	-0.65297
C	-5.76786	-1.41623	-0.34684	C	-6.58689	-0.28293	-1.60191
H	2.77955	0.37346	-1.54291	H	2.75067	0.3836	-1.54526
H	0.52781	-0.58141	-1.09285	H	0.52871	-0.65479	-1.08671
H	6.25174	-1.97427	-2.05439	H	6.28892	-1.84962	-2.07514
H	4.61838	-2.77483	-1.74766	H	4.6846	-2.70409	-1.7613
H	5.10881	3.14709	-1.4609	H	4.99015	3.23281	-1.45096
H	4.51006	1.67163	-2.4058	H	4.44469	1.74026	-2.4013
H	5.70748	-0.13449	-0.60945	H	5.6929	-0.03134	-0.62445
H	1.71525	0.95865	1.25984	H	1.67116	0.93267	1.25847
H	1.10919	1.64915	-0.23756	H	1.04022	1.60092	-0.23808
H	1.48864	-1.67972	1.59401	H	1.53295	-1.712	1.59928
H	0.73586	-2.60493	0.30216	H	0.80817	-2.66187	0.30893
H	2.56411	-2.0512	-1.24499	H	2.61116	-2.05208	-1.24325
H	3.20274	-2.8325	0.19694	H	3.28128	-2.80958	0.19704
H	2.78237	3.11083	1.01236	H	3.91893	2.13814	1.73935
H	4.01463	2.0927	1.73543	H	4.35259	3.56491	0.78227
H	4.49072	3.50394	0.77508	H	2.65715	3.11572	1.01142
H	3.26462	-0.33718	2.16868	H	3.27069	-0.3192	2.16602
H	4.90467	0.09424	1.67093	H	4.89024	0.17615	1.65928
H	4.46477	-1.6018	1.87516	H	4.51712	-1.53605	1.86372
H	-1.60241	-0.16106	-1.26757	H	-1.64355	-0.64147	-1.2182
H	0.24606	-0.06331	2.55845	H	-1.10192	0.90739	2.31324
H	-1.19893	-1.08239	2.51937	H	0.24897	-0.20999	2.57049
H	-1.37912	0.65743	2.44982	H	-1.43332	-0.78567	2.60168
H	-4.06295	1.5361	-1.04074	H	-3.84318	-0.41283	-1.76643
H	-3.82316	-0.02754	-1.80473	H	-4.44928	-1.68054	-0.70978
H	-5.31486	0.61368	1.28623	H	-5.3795	-0.05827	1.34585
H	-7.55567	0.3701	-1.43339	H	-6.28426	2.20934	-0.511
H	-6.40406	1.66705	-1.81189	H	-4.63879	2.02157	0.1193
H	-6.26654	0.09102	-2.62096	H	-4.9276	1.96264	-1.62982
H	-5.47717	-1.96983	-1.24584	H	-6.24319	-0.055	-2.61524
H	-5.15369	-1.77022	0.48642	H	-6.76079	-1.36126	-1.52764
H	-6.81388	-1.64743	-0.12207	H	-7.53919	0.23179	-1.43692
atom	Con f. 4- 3o			atom	Con f. 4- 3p		
C	-5.29161	2.30027	-0.82408	C	5.91304	-1.15253	-0.05485
C	-5.22175	-1.90794	-0.08642	C	4.14884	2.45548	-1.46849
C	-4.06412	-1.54116	0.47665	C	3.20675	2.04574	-0.6105
C	-3.166	-0.51917	-0.21066	C	2.81484	0.57435	-0.54499
C	-3.38229	0.97488	0.24889	C	3.58451	-0.27218	0.54296
C	-4.82674	1.36029	0.00051	C	5.07391	-0.17149	0.28143
C	-1.68469	-0.93526	-0.13017	C	1.28774	0.40622	-0.42244
C	-0.76892	0.00419	-0.94693	C	0.86149	-1.05743	-0.54412
C	-0.95291	1.44381	-0.46327	C	1.58091	-1.89465	0.53837
C	-2.42181	1.85817	-0.57572	C	3.0972	-1.73128	0.42409
C	-3.60632	-2.16919	1.77057	C	2.48533	3.03905	0.26775
C	-3.12234	1.17483	1.75762	C	3.35123	0.24706	1.97825
C	0.64294	-0.53666	-0.9385	C	-0.62571	-1.32351	-0.49741
C	1.62841	0.07591	-0.23569	C	-1.50061	-0.38388	-0.0592
C	0.85119	-1.79395	-1.73877	C	-1.04082	-2.69796	-0.9483
C	3.01247	-0.37544	-0.12714	C	-2.94573	-0.54822	0.06677
C	3.92111	0.4553	0.76452	C	-3.71382	0.65889	0.58106
O	3.44746	-1.39514	-0.69266	O	-3.541	-1.61025	-0.19058
C	5.42405	0.36736	0.43134	C	-5.19997	0.71107	0.17399
O	5.85853	-1.00425	0.51729	O	-5.87226	-0.47224	0.64744
C	5.70903	0.91456	-0.97368	C	-5.88413	1.8888	0.86286
C	6.23099	1.1322	1.47703	C	-5.34911	0.8183	-1.34934
H	-3.44217	-0.52394	-1.27246	H	3.11499	0.12855	-1.50172
H	-1.11053	-0.04645	-1.99216	H	1.21005	-1.43408	-1.51797

H	-6.35928	2.49006	-0.90298	H	6.97099	-0.9505	-0.20353
H	-4.6488	2.91516	-1.44679	H	5.59816	-2.18231	-0.19442
H	-5.87993	-2.63501	0.38361	H	4.44563	3.49964	-1.53484
H	-5.55112	-1.49093	-1.03472	H	4.65759	1.75949	-2.13067
H	-5.54596	0.79315	0.58904	H	5.47982	0.83145	0.4026
H	-1.33794	-0.93684	0.90873	H	0.94906	0.81145	0.53585
H	-1.59117	-1.96349	-0.49437	H	0.80208	1.00059	-1.20473
H	-0.61872	1.53859	0.57575	H	1.22498	-1.5742	1.52468
H	-0.33168	2.12294	-1.05815	H	1.32208	-2.95348	0.43745
H	-2.71563	1.81161	-1.63176	H	3.40984	-2.13077	-0.54853
H	-2.53802	2.90174	-0.2621	H	3.59326	-2.33892	1.1897
H	-2.72201	-2.79763	1.61029	H	2.92521	4.03553	0.16985
H	-3.32384	-1.42324	2.51867	H	1.42662	3.11239	-0.00836
H	-4.39133	-2.80084	2.19573	H	2.51054	2.75708	1.32437
H	-2.15436	0.77609	2.07081	H	2.29005	0.34708	2.21967
H	-3.89599	0.69202	2.36104	H	3.82499	1.22057	2.13091
H	-3.13864	2.24364	1.99729	H	3.79378	-0.4493	2.69886
H	1.39801	0.98293	0.31133	H	-1.12559	0.5905	0.23187
H	1.18681	-2.61997	-1.10363	H	-1.47181	-3.27308	-0.12182
H	1.63448	-1.65075	-2.48944	H	-0.18892	-3.25139	-1.3501
H	-0.06912	-2.09231	-2.24616	H	-1.81854	-2.63894	-1.71504
H	3.59169	1.49863	0.75367	H	-3.64607	0.62255	1.67822
H	3.76294	0.09119	1.79022	H	-3.20372	1.57621	0.27136
H	5.20964	-1.49659	-0.02154	H	-5.30808	-1.20885	0.34295
H	6.77907	0.83927	-1.19141	H	-6.94889	1.90514	0.60807
H	5.16558	0.34706	-1.73478	H	-5.79173	1.80387	1.95043
H	5.41339	1.96595	-1.05434	H	-5.43999	2.8382	0.54929
H	5.96669	2.19387	1.47631	H	-4.87033	1.72731	-1.72833
H	6.04461	0.72792	2.47723	H	-4.89547	-0.04178	-1.85038
H	7.30128	1.04331	1.26356	H	-6.40981	0.84898	-1.61786
atom		Con f. 4- 3q		atom		Con f. 4- 3r	
C	5.40591	-2.16787	-0.57353	C	5.86502	-1.24208	0.31607
C	5.0634	2.0566	0.065	C	4.30191	2.30271	-1.51937
C	3.89092	1.63117	0.5507	C	3.29396	1.97359	-0.70233
C	3.1021	0.55224	-0.18183	C	2.86048	0.51908	-0.56317
C	3.35787	-0.92099	0.32205	C	3.52185	-0.26225	0.63849
C	4.83481	-1.23393	0.18881	C	5.02866	-0.21642	0.48367
C	1.60017	0.89327	-0.22475	C	1.3247	0.3933	-0.54938
C	0.79843	-0.10379	-1.09207	C	0.86953	-1.06593	-0.59823
C	1.0171	-1.52443	-0.56846	C	1.47731	-1.83295	0.59831
C	2.50907	-1.86483	-0.55607	C	3.00201	-1.71457	0.59185
C	3.3085	2.2492	1.79875	C	2.53889	3.04258	0.05016
C	2.99542	-1.11117	1.81038	C	3.19994	0.36883	2.01021
C	-0.63582	0.36142	-1.20217	C	-0.62292	-1.29575	-0.66298
C	-1.64499	-0.3151	-0.59819	C	-1.5062	-0.29938	-0.4025
C	-0.84093	1.62259	-1.99682	C	-1.03583	-2.69936	-1.01529
C	-3.05612	0.05734	-0.61308	C	-2.96129	-0.41563	-0.4244
C	-4.01847	-0.91332	0.05166	C	-3.74033	0.87303	-0.21571
O	-3.48649	1.09169	-1.15562	O	-3.56061	-1.48625	-0.63273
C	-5.29601	-0.26769	0.62731	C	-5.16949	0.69208	0.33462
O	-6.02069	0.39162	-0.42904	O	-5.92856	-0.14389	-0.56028
C	-6.22508	-1.35229	1.16598	C	-5.14389	0.06389	1.73451
C	-4.95295	0.74378	1.72879	C	-5.88689	2.0389	0.36564
H	3.45839	0.55391	-1.21941	H	3.22015	-0.001	-1.45996
H	1.21618	-0.04976	-2.10929	H	1.28636	-1.52246	-1.50928
H	6.48478	-2.30274	-0.57089	H	6.93627	-1.07584	0.23256
H	4.84206	-2.83116	-1.22252	H	5.53478	-2.27481	0.25699
H	5.64394	2.82591	0.56887	H	4.6272	3.33371	-1.63762
H	5.48347	1.6487	-0.85096	H	4.8376	1.55217	-2.09517
H	5.47877	-0.6145	0.8109	H	5.45023	0.78609	0.53562
H	1.17193	0.89427	0.78325	H	0.92281	0.87463	0.34761
H	1.48596	1.9095	-0.61527	H	0.91787	0.93879	-1.40839
H	0.60599	-1.61954	0.44259	H	1.05705	-1.42799	1.52649
H	0.48111	-2.24485	-1.19669	H	1.19635	-2.89006	0.55848
H	2.88343	-1.82134	-1.58641	H	3.37783	-2.19621	-0.31932
H	2.65101	-2.89585	-0.21282	H	3.41864	-2.27197	1.43871
H	2.40537	2.82662	1.56693	H	1.50492	3.11951	-0.30692
H	3.01426	1.49887	2.53783	H	2.48119	2.83711	1.12295
H	4.02593	2.92856	2.2677	H	3.01155	4.01952	-0.08502
H	1.98723	-0.75927	2.0425	H	2.12728	0.50935	2.16489
H	3.69539	-0.57898	2.46018	H	3.68737	1.34063	2.12494
H	3.04887	-2.17376	2.07116	H	3.57107	-0.28066	2.81038
H	-1.41717	-1.22315	-0.05233	H	-1.13003	0.68961	-0.16787
H	-1.18888	2.4406	-1.35686	H	-1.75787	-2.69948	-1.83651
H	0.08661	1.93402	-2.48299	H	-1.53545	-3.18785	-0.17167

H	-1.61287	1.48414	-2.75897	H	-0.1707	-3.30152	-1.30206
H	-4.30882	-1.64015	-0.72075	H	-3.1679	1.54676	0.42922
H	-3.49651	-1.47785	0.82973	H	-3.7972	1.36087	-1.1998
H	-5.34716	0.9343	-0.88281	H	-5.35628	-0.92187	-0.70566
H	-7.15198	-0.90207	1.53631	H	-6.16643	-0.06184	2.10419
H	-6.48046	-2.06621	0.37621	H	-4.6649	-0.91941	1.71534
H	-5.75407	-1.89796	1.98903	H	-4.59693	0.69769	2.44053
H	-4.42024	0.2615	2.55516	H	-5.37692	2.7392	1.03388
H	-4.32333	1.54934	1.33966	H	-5.92319	2.47796	-0.6366
H	-5.8716	1.18925	2.12342	H	-6.914	1.90842	0.72212

atom	Con f. 4- 4a			atom	Con f. 4- 4b		
C	5.50952	-1.98966	-0.58285	C	3.76448	3.2697	1.14908
C	2.53329	2.59566	0.89479	C	3.97538	-1.95835	-1.01889
C	2.93105	2.09013	-0.27916	C	3.87275	-1.48258	0.22846
C	3.0912	0.60991	-0.61669	C	3.16189	-0.19984	0.64962
C	3.8596	-0.30417	0.40963	C	3.43158	1.10897	-0.1815
C	4.26944	-1.5578	-0.347	C	3.00071	2.27388	0.6963
C	1.71203	0.01456	-0.99953	C	1.64597	-0.48615	0.79819
C	0.84413	-0.29988	0.23314	C	0.91328	-0.51687	-0.56256
C	1.58105	-1.28745	1.1625	C	1.09462	0.82824	-1.27127
C	2.94592	-0.73539	1.58851	C	2.57997	1.1459	-1.47986
C	3.2082	3.00429	-1.44654	C	4.43115	-2.26691	1.38974
C	5.09039	0.42161	0.96456	C	4.91537	1.22551	-0.54733
C	-0.51949	-0.81779	-0.15889	C	-0.50836	-0.98261	-0.35608
C	-1.618	-0.1674	0.30254	C	-1.55921	-0.13567	-0.49689
C	-0.57658	-2.03604	-1.0369	C	-0.6532	-2.43012	0.02941
C	-3.00901	-0.52459	0.04451	C	-2.96436	-0.47611	-0.3014
C	-4.06265	0.21491	0.85226	C	-3.97294	0.62115	-0.60158
O	-3.34784	-1.40477	-0.76831	O	-3.35246	-1.60194	0.06138
C	-5.42536	0.37866	0.14705	C	-5.30151	0.51418	0.17492
O	-5.96163	-0.91884	-0.17507	O	-5.93111	-0.74796	-0.11909
C	-6.42573	1.02651	1.10052	C	-5.06622	0.63582	1.68612
C	-5.28375	1.21211	-1.13332	C	-6.26996	1.59197	-0.30481
H	3.69834	0.58291	-1.52927	H	3.52972	0.02109	1.65876
H	0.6983	0.63287	0.78718	H	1.39841	-1.29436	-1.16927
H	5.68091	-2.90759	-1.13979	H	3.34217	4.05226	1.77472
H	6.39588	-1.46326	-0.24173	H	4.82371	3.35419	0.92566
H	2.41166	3.66907	1.02108	H	4.46769	-2.90971	-1.20726
H	2.32762	1.98672	1.76628	H	3.59944	-1.43566	-1.88975
H	3.44882	-2.15956	-0.73212	H	1.94991	2.28157	0.97904
H	1.86242	-0.89725	-1.58519	H	1.17962	0.28393	1.42224
H	1.18557	0.72061	-1.65123	H	1.51656	-1.43551	1.32661
H	0.97088	-1.47687	2.05261	H	0.58598	0.81444	-2.2418
H	1.70304	-2.25027	0.65531	H	0.62445	1.62436	-0.68311
H	3.47734	-1.4906	2.17991	H	2.68363	2.13919	-1.93246
H	2.79298	0.12083	2.25266	H	2.99943	0.43737	-2.20093
H	2.98495	4.04908	-1.21273	H	3.66026	-2.44431	2.15081
H	4.26134	2.93463	-1.74887	H	4.8332	-3.23449	1.07636
H	2.61493	2.71283	-2.32288	H	5.23208	-1.70347	1.88611
H	5.75075	0.76189	0.15949	H	5.11631	2.17617	-1.05176
H	4.79612	1.2966	1.54585	H	5.55202	1.17398	0.34255
H	5.66853	-0.24006	1.61786	H	5.21463	0.41894	-1.21808
H	-1.46816	0.69463	0.94672	H	-1.37204	0.89155	-0.78749
H	0.38988	-2.54078	-1.08601	H	-1.34301	-2.94547	-0.64529
H	-1.33555	-2.74176	-0.69233	H	0.31327	-2.93905	0.00317
H	-0.86072	-1.75422	-2.05798	H	-1.07654	-2.53459	1.03382
H	-4.21052	-0.36831	1.77262	H	-3.51347	1.59981	-0.43386
H	-3.6777	1.1912	1.16032	H	-4.18623	0.55828	-1.67863
H	-5.22109	-1.3873	-0.6071	H	-5.22316	-1.40785	0.01365
H	-6.10187	2.03234	1.38413	H	-4.61334	1.60052	1.93823
H	-6.53419	0.42645	2.00973	H	-4.40457	-0.15748	2.04618
H	-7.40626	1.10305	0.61914	H	-6.01923	0.55266	2.21797
H	-4.90103	2.21384	-0.91157	H	-7.22738	1.49517	0.21763
H	-4.59986	0.73309	-1.84042	H	-5.87078	2.59195	-0.11056
H	-6.25903	1.31506	-1.61944	H	-6.45254	1.49309	-1.37972

atom	Con f. 4- 4c			atom	Con f. 4- 4d		
C	-4.37921	-2.64731	0.52651	C	-3.76447	-3.26946	1.14948
C	-2.58558	2.57358	-1.09952	C	-3.97536	1.95829	-1.01912
C	-2.83917	2.18856	0.15716	C	-3.87277	1.48256	0.22826
C	-3.0293	0.75695	0.64864	C	-3.16199	0.19982	0.64953
C	-3.91766	-0.22028	-0.19478	C	-3.43155	-1.10905	-0.18157
C	-4.35338	-1.33292	0.7492	C	-3.00061	-2.27391	0.69626
C	-1.6569	0.11176	0.95793	C	-1.64607	0.4861	0.79827

C	-0.91505	-0.31537	-0.32336	C	-0.91331	0.51691	-0.56244
C	-1.7693	-1.32699	-1.11349	C	-1.09458	-0.82815	-1.27126
C	-3.16291	-0.76635	-1.42496	C	-2.5799	-1.14593	-1.47989
C	-2.90688	3.21224	1.26403	C	-4.43123	2.26694	1.38947
C	-5.21259	0.48331	-0.64914	C	-4.91533	-1.22569	-0.54741
C	0.45579	-0.86207	-0.00373	C	0.50834	0.98263	-0.3559
C	1.54623	-0.19521	-0.46145	C	1.55921	0.13575	-0.4969
C	0.53424	-2.11594	0.82223	C	0.65316	2.43008	0.02987
C	2.94009	-0.56189	-0.2393	C	2.96436	0.47614	-0.30135
C	3.99158	0.3605	-0.83442	C	3.97296	-0.62106	-0.60173
O	3.2869	-1.58502	0.38	O	3.35247	1.6019	0.06164
C	5.31441	0.42263	-0.04128	C	5.30152	-0.51416	0.17482
O	5.89764	-0.8926	0.02863	O	5.93111	0.748	-0.11904
C	6.32247	1.29488	-0.78441	C	5.06623	-0.63599	1.68601
C	5.07794	0.96035	1.3764	C	6.27	-1.59189	-0.30501
H	-3.54192	0.85294	1.61368	H	-3.52994	-0.02109	1.65863
H	-0.7832	0.57809	-0.94218	H	-1.39841	1.29444	-1.16911
H	-4.76658	-3.33011	1.27846	H	-3.34216	-4.05201	1.77513
H	-4.03156	-3.09975	-0.39757	H	-4.82379	-3.35374	0.9264
H	-2.43063	3.62445	-1.33323	H	-4.46766	2.90966	-1.2075
H	-2.53112	1.88691	-1.93462	H	-3.59943	1.43557	-1.88997
H	-4.73208	-0.97015	1.70657	H	-1.94973	-2.28179	0.97866
H	-1.82005	-0.76261	1.59678	H	-1.17981	-0.28404	1.42229
H	-1.0396	0.81534	1.52691	H	-1.5167	1.43544	1.32674
H	-1.26071	-1.58438	-2.04954	H	-0.58592	-0.81424	-2.24178
H	-1.86125	-2.25165	-0.53671	H	-0.62437	-1.62429	-0.68317
H	-3.7719	-1.5414	-1.90411	H	-2.68345	-2.13924	-1.9325
H	-3.07117	0.03235	-2.16604	H	-2.9994	-0.43746	-2.20098
H	-2.65559	4.21625	0.91074	H	-3.6604	2.44435	2.1506
H	-3.91393	3.24114	1.70038	H	-4.83324	3.23451	1.07604
H	-2.22252	2.95359	2.08224	H	-5.23222	1.70353	1.8858
H	-5.76755	0.87652	0.2104	H	-5.11626	-2.17648	-1.05158
H	-4.99597	1.31843	-1.31956	H	-5.55201	-1.17391	0.34243
H	-5.86368	-0.22341	-1.17413	H	-5.21457	-0.41929	-1.21838
H	1.38689	0.70916	-1.04227	H	1.37206	-0.89141	-0.78771
H	0.9991	-2.92664	0.25095	H	1.07668	2.53436	1.03422
H	1.16733	-1.96546	1.70145	H	1.34282	2.94559	-0.64485
H	-0.45215	-2.44683	1.14971	H	-0.31334	2.93896	0.0039
H	4.2046	-0.01879	-1.84421	H	3.51352	-1.59976	-0.4342
H	3.57886	1.36585	-0.95765	H	4.18632	-0.55797	-1.67874
H	5.16237	-1.46905	0.31439	H	5.22313	1.40786	0.01369
H	5.96018	2.32335	-0.87359	H	4.61339	-1.60074	1.93801
H	6.50354	0.90103	-1.78972	H	4.40454	0.15724	2.04617
H	7.27469	1.31032	-0.24393	H	6.01924	-0.55285	2.21787
H	4.65521	1.97024	1.34932	H	5.87085	-2.59189	-0.11082
H	4.38974	0.31574	1.93139	H	6.45256	-1.49294	-1.37992
H	6.02549	0.99708	1.92306	H	7.22743	-1.4951	0.21742
atom	Con f. 4- 4e			atom	Con f. 4- 4f		
C	-2.56882	3.31443	-0.93475	C	-4.24972	-2.2634	2.18755
C	-3.98974	-1.76667	1.35559	C	-3.47673	2.18698	-1.22029
C	-3.94936	-1.48714	0.04696	C	-3.23359	1.94787	0.07441
C	-3.31614	-0.26024	-0.59956	C	-2.83572	0.60702	0.68678
C	-3.51458	1.14236	0.06877	C	-3.65902	-0.6702	0.27452
C	-3.26502	2.18039	-1.01763	C	-3.37834	-1.72911	1.32997
C	-1.81527	-0.52907	-0.8641	C	-1.31166	0.38796	0.51023
C	-0.96822	-0.41558	0.42405	C	-0.94444	-0.05054	-0.90875
C	-1.10852	0.99639	0.99881	C	-1.67597	-1.37219	-1.24791
C	-2.57768	1.33902	1.27996	C	-3.19492	-1.22929	-1.09725
C	-4.51439	-2.46412	-0.95536	C	-3.27806	3.07432	1.07666
C	-4.98124	1.33297	0.5069	C	-5.15665	-0.35053	0.21106
C	0.4363	-0.89127	0.13837	C	0.53252	-0.19477	-1.19407
C	1.48231	-0.02852	0.08486	C	1.44964	-0.22373	-0.19446
C	0.56879	-2.37218	-0.09591	C	0.89116	-0.31279	-2.65087
C	2.86517	-0.37886	-0.22122	C	2.89195	-0.3759	-0.35663
C	3.86154	0.76952	-0.23902	C	3.72011	-0.33329	0.91775
O	3.23577	-1.53645	-0.48992	O	3.44312	-0.56072	-1.45708
C	5.32562	0.36734	0.03196	C	5.17671	0.13879	0.73031
O	5.74995	-0.59789	-0.95004	O	5.85138	-0.73465	-0.1959
C	6.23651	1.57888	-0.14527	C	5.22459	1.58134	0.20992
C	5.48122	-0.22354	1.43937	C	5.9326	0.01972	2.05101
H	-3.7875	-0.1818	-1.58723	H	-3.00645	0.7172	1.76396
H	-1.3878	-1.1294	1.14714	H	-1.3118	0.71171	-1.60928
H	-2.51565	3.99675	-1.7793	H	-3.93253	-3.01759	2.90358
H	-2.0341	3.61677	-0.03923	H	-5.29842	-1.9834	2.21885
H	-4.43366	-2.69495	1.70785	H	-3.71497	3.19216	-1.56052

H	-3.6085	-1.10094	2.11947	H	-3.46289	1.41719	-1.98198
H	-3.77682	1.96276	-1.95691	H	-2.34933	-2.07988	1.37824
H	-1.4399	0.20297	-1.58895	H	-0.97898	-0.37018	1.22659
H	-1.6995	-1.51715	-1.31941	H	-0.78571	1.31378	0.76797
H	-0.52785	1.08656	1.92394	H	-1.44614	-1.68524	-2.27125
H	-0.69405	1.72065	0.29088	H	-1.29177	-2.15811	-0.58697
H	-2.65545	2.37493	1.62858	H	-3.66901	-2.20595	-1.25364
H	-2.93626	0.72314	2.10958	H	-3.57079	-0.57821	-1.8925
H	-5.3611	-2.01748	-1.49285	H	-3.45451	4.04262	0.59984
H	-3.76709	-2.72449	-1.71562	H	-4.0721	2.90264	1.81521
H	-4.85661	-3.38758	-0.47998	H	-2.33861	3.13413	1.64122
H	-5.66433	1.19852	-0.33961	H	-5.7392	-1.25688	0.01628
H	-5.26094	0.6149	1.28116	H	-5.51257	0.08416	1.15128
H	-5.12971	2.34373	0.9008	H	-5.36676	0.36426	-0.58608
H	1.30825	1.02375	0.27833	H	1.11539	-0.11526	0.83059
H	0.92164	-2.58462	-1.11035	H	1.31877	-1.29549	-2.8763
H	1.31058	-2.8078	0.58009	H	0.01364	-0.16039	-3.28343
H	-0.38758	-2.87816	0.05627	H	1.65539	0.42038	-2.92468
H	3.79629	1.21986	-1.24028	H	3.2152	0.28669	1.66461
H	3.54333	1.54184	0.46787	H	3.72826	-1.35772	1.3175
H	5.04511	-1.27397	-0.94222	H	5.24785	-0.78793	-0.96202
H	5.98773	2.36507	0.57381	H	4.74033	2.26946	0.91068
H	6.13861	1.9884	-1.15589	H	4.72037	1.66632	-0.75708
H	7.28102	1.2886	0.00894	H	6.26515	1.89565	0.08128
H	5.19003	0.50219	2.20602	H	5.49062	0.66639	2.81487
H	4.86058	-1.11622	1.55972	H	5.91057	-1.01289	2.4143
H	6.52456	-0.5068	1.61037	H	6.97827	0.31391	1.91308
atom	Con f. 4- 4g			atom	Con f. 4- 4h		
C	-3.84589	3.36097	-0.09923	C	3.84606	-3.36084	-0.0993
C	-3.8443	-2.27957	-0.33832	C	3.8442	2.27962	-0.33856
C	-3.50298	-1.32294	-1.21012	C	3.50282	1.32292	-1.21027
C	-2.90699	0.04054	-0.8708	C	2.90696	-0.04058	-0.87081
C	-3.53475	0.85524	0.32094	C	3.53484	-0.85515	0.32096
C	-3.08023	2.29697	0.14921	C	3.08039	-2.29691	0.14937
C	-1.36933	-0.09184	-0.72508	C	1.3693	0.0917	-0.72501
C	-0.96124	-0.70229	0.61718	C	0.96125	0.70225	0.61722
C	-1.49172	0.18137	1.77244	C	1.49185	-0.18127	1.77253
C	-3.01263	0.35304	1.69427	C	3.01277	-0.35287	1.69428
C	-3.6351	-1.56317	-2.69366	C	3.63477	1.56306	-2.69384
C	-5.06444	0.76361	0.29487	C	5.06452	-0.76344	0.29477
C	0.51682	-0.95307	0.80562	C	-0.51681	0.95297	0.80573
C	1.44262	-0.36193	0.009	C	-1.44262	0.36184	0.00911
C	0.86937	-1.87036	1.94521	C	-0.86935	1.87024	1.94534
C	2.88983	-0.51416	0.11665	C	-2.88983	0.51405	0.1168
C	3.71935	0.2083	-0.93265	C	-3.71936	-0.2082	-0.93264
O	3.44355	-1.21285	0.98512	O	-3.44355	1.21255	0.98543
C	5.16128	0.54919	-0.50597	C	-5.1613	-0.54914	-0.50603
O	5.85938	-0.66173	-0.15578	O	-5.85936	0.66173	-0.15559
C	5.92129	1.14929	-1.68578	C	-5.92132	-1.14896	-1.68597
C	5.16898	1.51532	0.68626	C	-5.16905	-1.51552	0.686
H	-3.07773	0.66093	-1.75851	H	3.0777	-0.66102	-1.75848
H	-1.44896	-1.68243	0.70984	H	1.44892	1.68242	0.70976
H	-3.40508	4.34933	-0.20411	H	3.40532	-4.34924	-0.20403
H	-4.92485	3.30336	-0.20807	H	4.92498	-3.30313	-0.20846
H	-4.22444	-3.23642	-0.6888	H	4.22424	3.23647	-0.68916
H	-3.77697	-2.15745	0.73559	H	3.777	2.15756	0.73536
H	-2.00892	2.4626	0.24163	H	2.00913	-2.46265	0.24215
H	-0.91822	0.89997	-0.83203	H	0.91827	-0.90017	-0.83182
H	-0.98204	-0.70054	-1.54934	H	0.9819	0.70028	-1.5493
H	-1.22988	-0.25777	2.74041	H	1.23006	0.25795	2.74048
H	-0.98648	1.15333	1.72269	H	0.98666	-1.15326	1.72291
H	-3.34058	1.05675	2.46891	H	3.3408	-1.0565	2.46895
H	-3.4884	-0.60392	1.9298	H	3.48851	0.60412	1.92972
H	-2.68307	-1.37911	-3.208	H	3.95374	2.58488	-2.91801
H	-3.95417	-2.58498	-2.91773	H	4.36316	0.86882	-3.13301
H	-4.36349	-0.86891	-3.1328	H	2.6827	1.37891	-3.20807
H	-5.46659	1.07715	-0.67456	H	5.46662	-1.07693	-0.67469
H	-5.39628	-0.25944	0.4764	H	5.39631	0.25963	0.47629
H	-5.50423	1.40463	1.06589	H	5.5044	-1.40445	1.06575
H	1.11043	0.28639	-0.79339	H	-1.11043	-0.28641	-0.79333
H	1.48845	-2.70205	1.59567	H	-1.46045	1.35139	2.70698
H	1.46078	-1.35164	2.70669	H	0.03066	2.27379	2.41502
H	-0.03063	-2.27369	2.41508	H	-1.48871	2.70175	1.59591
H	3.75583	-0.45564	-1.80862	H	-3.75585	0.45591	-1.80847
H	3.19508	1.11363	-1.25357	H	-3.19511	-1.11348	-1.25374

H	5.25924	-1.11479	0.46726	H	-5.25921	1.11465	0.46754
H	5.46274	2.08834	-2.00968	H	-5.46279	-2.08794	-2.0101
H	5.92774	0.45508	-2.53231	H	-5.92778	-0.45455	-2.53234
H	6.95837	1.35288	-1.39938	H	-6.95841	-1.3526	-1.39962
H	4.66767	2.45576	0.43453	H	-6.20051	-1.74105	0.97458
H	4.66116	1.0775	1.5506	H	-4.66775	-2.45592	0.43408
H	6.20043	1.7408	0.97492	H	-4.66125	-1.0779	1.55044
atom		Con f. 4- 4i		atom		Con f. 4- 4j	
C	-2.56799	3.30946	-0.94768	C	4.22979	2.38067	2.06986
C	-3.98377	-1.75858	1.37109	C	3.47832	-2.24609	-1.09802
C	-3.94736	-1.48613	0.06078	C	3.22624	-1.93951	0.1805
C	-3.31776	-0.26195	-0.59399	C	2.82638	-0.56834	0.71962
C	-3.51159	1.14333	0.06981	C	3.64959	0.6862	0.2426
C	-3.2667	2.17641	-1.02221	C	3.36271	1.79913	1.23918
C	-1.81834	-0.53256	-0.86647	C	1.30257	-0.35968	0.5268
C	-0.96442	-0.41466	0.41653	C	0.94139	0.0024	-0.91511
C	-1.10058	0.99962	0.9868	C	1.67246	1.305	-1.32192
C	-2.56778	1.34443	1.27501	C	3.19074	1.17147	-1.15888
C	-4.51195	-2.47036	-0.93449	C	3.26355	-3.01185	1.24067
C	-4.97588	1.33698	0.51489	C	5.14806	0.36669	0.20308
C	0.43861	-0.8913	0.1245	C	-0.53429	0.1282	-1.21413
C	1.48518	-0.02936	0.06776	C	-1.45378	0.22947	-0.22113
C	0.57008	-2.37174	-0.11247	C	-0.88928	0.143	-2.67632
C	2.86562	-0.38134	-0.24613	C	-2.8948	0.37265	-0.39894
C	3.86377	0.76441	-0.28802	C	-3.72914	0.47708	0.86781
O	3.2357	-1.5416	-0.50502	O	-3.44541	0.43598	-1.51372
C	5.31828	0.36764	0.04308	C	-5.15756	-0.09538	0.74595
O	5.77121	-0.62705	-0.89553	O	-5.87247	0.6059	-0.28951
C	6.23713	1.57249	-0.13881	C	-5.12108	-1.59565	0.4255
C	5.4271	-0.18159	1.4717	C	-5.92817	0.15786	2.03889
H	-3.79397	-0.18708	-1.57953	H	2.99378	-0.62116	1.80165
H	-1.37981	-1.12605	1.14427	H	1.31351	-0.79602	-1.57153
H	-2.51814	3.98844	-1.79508	H	3.90822	3.17101	2.74374
H	-2.02767	3.61417	-0.05631	H	5.2791	2.10573	2.11964
H	-4.42468	-2.68587	1.72955	H	3.71742	-3.26781	-1.3841
H	-3.60288	-1.08733	2.13033	H	3.47106	-1.5165	-1.89829
H	-3.78403	1.95565	-1.95768	H	2.33251	2.14859	1.26648
H	-1.44717	0.19648	-1.5964	H	0.96745	0.43495	1.2013
H	-1.70586	-1.52265	-1.31846	H	0.77555	-1.2706	0.8309
H	-0.5136	1.09276	1.90767	H	1.44545	1.56282	-2.36125
H	-0.69043	1.72081	0.27336	H	1.28585	2.12458	-0.70479
H	-2.64272	2.38178	1.6198	H	3.66476	2.13916	-1.36336
H	-2.92206	0.7326	2.10931	H	3.57029	0.48055	-1.91823
H	-5.36123	-2.0292	-1.47232	H	3.44189	-4.00383	0.8161
H	-3.76597	-2.7328	-1.69545	H	4.05363	-2.80174	1.97351
H	-4.84991	-3.3923	-0.45319	H	2.32087	-3.04193	1.80234
H	-5.12154	2.34946	0.90524	H	5.7293	1.26229	-0.03947
H	-5.66362	1.19934	-0.32731	H	5.50046	-0.01359	1.16794
H	-5.25154	0.62229	1.29369	H	5.36387	-0.39097	-0.55168
H	1.31287	1.02292	0.26207	H	-1.12107	0.19959	0.80963
H	1.2978	-2.81047	0.57707	H	-1.61771	-0.64033	-2.90618
H	-0.38965	-2.87611	0.02168	H	-1.36124	1.08875	-2.96228
H	0.94241	-2.58293	-1.12	H	-0.00277	-0.00877	-3.29634
H	3.83376	1.16515	-1.31146	H	-3.2033	0.00289	1.70147
H	3.53194	1.57057	0.37263	H	-3.7987	1.5478	1.10784
H	5.05693	-1.29357	-0.90364	H	-5.25964	0.60527	-1.05033
H	5.97146	2.37687	0.5536	H	-4.59994	-2.15401	1.21028
H	6.16911	1.95679	-1.16159	H	-4.61056	-1.78226	-0.524
H	7.27578	1.28287	0.05166	H	-6.1414	-1.98368	0.34508
H	5.10655	0.56421	2.20674	H	-5.46049	-0.35972	2.88173
H	4.80759	-1.07439	1.59705	H	-5.96088	1.22899	2.26285
H	6.46497	-0.45444	1.68699	H	-6.95651	-0.20483	1.93906
atom		Con f. 4- 4k		atom		Con f. 4- 4l	
C	-5.4329	-2.01763	0.77589	C	5.45393	-2.05007	-0.63108
C	-3.70165	2.9472	1.19342	C	3.78678	2.88514	-1.25537
C	-3.02378	2.11665	0.39095	C	3.07222	2.09151	-0.44734
C	-2.99644	0.62735	0.71271	C	3.0412	0.59169	-0.71515
C	-3.85624	-0.28539	-0.25135	C	3.85363	-0.29336	0.31453
C	-4.21269	-1.53217	0.54086	C	4.22703	-1.57236	-0.41575
C	-1.56031	0.07854	0.91301	C	1.60797	0.04942	-0.95106
C	-0.82604	-0.33382	-0.3828	C	0.82055	-0.30587	0.32962
C	-1.67976	-1.32564	-1.1934	C	1.63097	-1.27605	1.209
C	-3.04894	-0.71822	-1.50511	C	2.99517	-0.67355	1.55075
C	-2.2711	2.66486	-0.79555	C	2.28263	2.69094	0.68946
C	-5.1177	0.45015	-0.71527	C	5.10404	0.44803	0.7986

C	0.54717	-0.87957	-0.06639	C	-0.54631	-0.85079	-0.01442
C	1.63416	-0.17374	-0.46994	C	-1.64079	-0.16065	0.3964
C	0.63116	-2.17342	0.69435	C	-0.61629	-2.12804	-0.80442
C	3.03085	-0.53131	-0.24658	C	-3.03364	-0.52239	0.15634
C	4.07119	0.46057	-0.74063	C	-4.08577	0.37758	0.78367
O	3.38567	-1.59665	0.29055	O	-3.37572	-1.5236	-0.49957
C	5.40242	0.44399	0.03923	C	-5.43106	0.42585	0.0295
O	5.99278	-0.86737	-0.04393	O	-5.99072	-0.89948	-0.04444
C	6.39681	1.39893	-0.61536	C	-6.43416	1.26606	0.81524
C	5.18258	0.82122	1.51024	C	-5.24627	0.99037	-1.38518
H	-3.48635	0.5453	1.68911	H	3.56774	0.46922	-1.66777
H	-0.68842	0.5548	-1.0033	H	0.66995	0.60564	0.91273
H	-5.56678	-2.92391	1.36138	H	5.6002	-2.98169	-1.17216
H	-6.33907	-1.54747	0.40568	H	6.35374	-1.54844	-0.28764
H	-3.74237	4.01708	1.00225	H	3.83113	3.96108	-1.10309
H	-4.23752	2.58787	2.06852	H	4.34977	2.48829	-2.09658
H	-3.36769	-2.07535	0.9604	H	3.38923	-2.14906	-0.80352
H	-1.61552	-0.79307	1.57266	H	1.68059	-0.84686	-1.57535
H	-0.96564	0.82893	1.4455	H	1.04303	0.78492	-1.53444
H	-1.16452	-1.57146	-2.1288	H	1.07739	-1.48052	2.13228
H	-1.7975	-2.26468	-0.64275	H	1.75607	-2.23633	0.69824
H	-3.65297	-1.43746	-2.07081	H	3.56849	-1.37987	2.16287
H	-2.91085	0.15173	-2.15688	H	2.84442	0.21945	2.16737
H	-2.5302	2.14704	-1.72379	H	2.5009	2.20577	1.64543
H	-2.48215	3.72968	-0.92987	H	2.50079	3.75788	0.79207
H	-1.18847	2.55591	-0.66475	H	1.20439	2.58848	0.52203
H	-5.72592	0.78505	0.13072	H	5.74415	0.75211	-0.0354
H	-4.85822	1.33089	-1.30758	H	4.83111	1.34917	1.35296
H	-5.73524	-0.20127	-1.34216	H	5.69399	-0.1879	1.46665
H	1.4709	0.75909	-1.00296	H	-1.48641	0.75304	0.96382
H	1.05789	-2.96117	0.06333	H	0.37422	-2.5499	-0.98046
H	1.2976	-2.08159	1.55599	H	-1.23009	-2.87257	-0.28895
H	-0.34978	-2.50534	1.03721	H	-1.09691	-1.95813	-1.77361
H	4.27358	0.20257	-1.79029	H	-4.26227	-0.0113	1.79705
H	3.64748	1.46912	-0.74441	H	-3.68444	1.38806	0.90423
H	5.26871	-1.47503	0.20164	H	-5.25667	-1.45433	-0.37203
H	6.03117	2.42947	-0.57975	H	-6.08766	2.29912	0.91382
H	6.56236	1.12419	-1.6621	H	-6.58094	0.85107	1.81765
H	7.35754	1.35586	-0.09174	H	-7.40081	1.27413	0.30092
H	4.75761	1.8264	1.60026	H	-4.84441	2.0086	-1.35335
H	4.50282	0.11721	1.99915	H	-4.5605	0.36961	-1.9694
H	6.13712	0.80058	2.04543	H	-6.20964	1.01517	-1.90414
atom		Con f. 4- 4m		atom		Con f. 4- 4n	
C	-3.89124	3.25523	-1.02941	C	3.54809	3.41849	0.80101
C	-5.08543	-1.85771	-0.96885	C	4.94676	-1.59502	1.5045
C	-4.05343	-1.45685	-0.21557	C	4.01163	-1.3431	0.5796
C	-3.23386	-0.25084	-0.65771	C	3.09273	-0.14144	0.75983
C	-3.44398	1.05944	0.20392	C	3.37509	1.07504	-0.21065
C	-3.10357	2.22834	-0.70548	C	2.85941	2.31698	0.49711
C	-1.7341	-0.58366	-0.86399	C	1.59197	-0.52645	0.79447
C	-0.86162	-0.52385	0.41585	C	0.90457	-0.6487	-0.58943
C	-1.02113	0.84049	1.08849	C	1.11511	0.63692	-1.39162
C	-2.49053	1.09972	1.42765	C	2.60855	0.92723	-1.55312
C	-3.69638	-2.21845	1.03693	C	3.85881	-2.26465	-0.60484
C	-4.8882	1.15705	0.70665	C	4.87182	1.19667	-0.51416
C	0.54963	-0.93984	0.06772	C	-0.52972	-1.08022	-0.38157
C	1.56573	-0.04137	0.04122	C	-1.55817	-0.20981	-0.54007
C	0.72432	-2.40008	-0.25122	C	-0.71196	-2.5135	0.03874
C	2.95498	-0.32931	-0.30036	C	-2.97173	-0.50587	-0.33107
C	3.91687	0.84749	-0.26509	C	-3.94173	0.63996	-0.5711
O	3.35884	-1.45841	-0.63395	O	-3.3954	-1.62893	-0.00191
C	5.38438	0.47765	0.03474	C	-5.26241	0.55094	0.22026
O	5.86575	-0.43801	-0.96786	O	-5.94623	-0.67079	-0.11963
C	6.26246	1.72151	-0.07106	C	-5.00253	0.59613	1.73181
C	5.51657	-0.15931	1.42432	C	-6.19284	1.68672	-0.19648
H	-3.62073	-0.0008	-1.65171	H	3.32379	0.23651	1.76185
H	-1.2304	-1.28366	1.11533	H	1.38588	-1.46535	-1.14046
H	-3.53806	4.03723	-1.69711	H	3.06905	4.24857	1.31442
H	-4.90459	3.36545	-0.65478	H	4.59934	3.54626	0.56036
H	-5.69333	-2.71601	-0.69187	H	5.62071	-2.44411	1.41675
H	-5.35733	-1.34368	-1.88748	H	5.07052	-0.96283	2.38022
H	-2.10442	2.20538	-1.13691	H	1.81033	2.28534	0.78599
H	-1.30997	0.1302	-1.57924	H	1.0464	0.23734	1.36
H	-1.65769	-1.56978	-1.33249	H	1.48423	-1.45954	1.35639
H	-0.41997	0.87971	2.00396	H	0.64733	0.54227	-2.37804

H	-0.645	1.63251	0.43178	H	0.62704	1.4804	-0.89216
H	-2.59336	2.07902	1.90961	H	2.74763	1.84944	-2.12928
H	-2.82311	0.35689	2.16144	H	3.06768	0.12328	-2.13925
H	-3.61542	-1.56372	1.90981	H	4.65884	-3.01041	-0.61917
H	-4.44934	-2.98202	1.25234	H	2.90504	-2.8034	-0.57022
H	-2.73065	-2.72609	0.9329	H	3.88156	-1.72349	-1.55565
H	-5.608	1.12537	-0.11696	H	5.0652	2.06854	-1.14753
H	-5.12071	0.33201	1.38437	H	5.46473	1.30149	0.39969
H	-5.04083	2.0914	1.25655	H	5.23333	0.31339	-1.04617
H	1.36176	0.99212	0.29584	H	-1.34391	0.80839	-0.84372
H	1.07623	-2.54449	-1.27777	H	0.24885	-3.03036	0.09269
H	1.4837	-2.85049	0.39532	H	-1.20663	-2.58577	1.01239
H	-0.2144	-2.9436	-0.12253	H	-1.35677	-3.04456	-0.66861
H	3.86678	1.31892	-1.25736	H	-3.44209	1.59214	-0.36861
H	3.5613	1.59358	0.45166	H	-4.17142	0.63182	-1.64657
H	5.17757	-1.12953	-1.01506	H	-5.26546	-1.36419	-0.02201
H	5.97083	2.47117	0.67052	H	-4.37062	-0.23914	2.04758
H	6.17892	2.16704	-1.06761	H	-5.95136	0.52982	2.27343
H	7.31079	1.45597	0.10091	H	-4.50593	1.52911	2.01852
H	5.17722	0.52776	2.20666	H	-7.1475	1.60266	0.33317
H	4.92363	-1.07605	1.49298	H	-5.75254	2.66013	0.03944
H	6.56313	-0.41419	1.61876	H	-6.39091	1.64609	-1.27243
atom		Con f. 4- 4o		atom		Con f. 4- 4p	
C	5.34693	-2.28353	-0.26078	C	3.54787	3.41855	0.80101
C	4.11777	2.72502	-1.25683	C	4.94687	-1.59491	1.50446
C	3.26879	2.02399	-0.49455	C	4.01171	-1.34303	0.57959
C	3.16052	0.51732	-0.69648	C	3.09274	-0.14142	0.75981
C	3.81077	-0.3642	0.44428	C	3.37502	1.07509	-0.21067
C	4.1471	-1.70498	-0.18617	C	2.85928	2.31699	0.49712
C	1.72303	0.0542	-1.04546	C	1.59201	-0.52648	0.79446
C	0.79529	-0.18159	0.16757	C	0.90457	-0.64883	-0.58941
C	1.45243	-1.14278	1.17592	C	1.11506	0.63678	-1.39166
C	2.8197	-0.61265	1.61274	C	2.60848	0.92721	-1.55314
C	2.41042	2.73366	0.52263	C	3.85885	-2.2646	-0.60482
C	5.06654	0.31131	1.00423	C	4.87173	1.19683	-0.5142
C	-0.56186	-0.67048	-0.28239	C	-0.5297	-1.08033	-0.38146
C	-1.64418	0.11577	-0.05057	C	-1.55816	-0.20997	-0.54013
C	-0.63735	-2.0088	-0.96313	C	-0.71191	-2.51358	0.03897
C	-3.02473	-0.1878	-0.40977	C	-2.97175	-0.506	-0.33112
C	-4.06211	0.87894	-0.09951	C	-3.94163	0.63995	-0.57102
O	-3.36716	-1.24784	-0.96624	O	-3.39548	-1.62907	-0.00211
C	-5.4671	0.33233	0.23209	C	-5.26235	0.55101	0.22027
O	-5.96417	-0.42807	-0.88595	O	-5.94635	-0.67055	-0.11986
C	-5.42886	-0.5536	1.48418	C	-5.00245	0.59586	1.73182
C	-6.44136	1.49132	0.42312	C	-6.1926	1.68701	-0.19629
H	3.76468	0.31386	-1.58732	H	3.3238	0.23656	1.76184
H	0.64469	0.76998	0.68271	H	1.38586	-1.4655	-1.14041
H	5.46811	-3.25119	-0.74145	H	3.06878	4.24859	1.31445
H	6.24827	-1.83233	0.14343	H	4.59911	3.54642	0.56035
H	4.22365	3.80215	-1.14991	H	5.62082	-2.44399	1.41672
H	4.73539	2.24717	-2.01323	H	5.07062	-0.9627	2.38018
H	3.30478	-2.23679	-0.62463	H	1.81019	2.28528	0.78601
H	1.79312	-0.87623	-1.61788	H	1.04639	0.2373	1.35996
H	1.26597	0.79188	-1.71422	H	1.48431	-1.45954	1.35644
H	0.80305	-1.2503	2.05201	H	0.64732	0.54206	-2.3781
H	1.55499	-2.14028	0.73656	H	0.62691	1.48023	-0.89225
H	3.28239	-1.31919	2.31201	H	2.74748	1.84946	-2.12928
H	2.67877	0.32281	2.16526	H	3.06768	0.12332	-2.13929
H	2.68501	3.79025	0.5907	H	4.65878	-3.01045	-0.61911
H	1.34962	2.68416	0.25209	H	2.90501	-2.80322	-0.57025
H	2.50196	2.29403	1.52038	H	3.8817	-1.72346	-1.55564
H	5.54453	-0.32774	1.7538	H	5.06503	2.06877	-1.14748
H	5.79922	0.5215	0.21891	H	5.46466	1.30159	0.39964
H	4.81524	1.25914	1.48642	H	5.23329	0.31363	-1.04632
H	-1.48861	1.06793	0.4489	H	-1.34391	0.80821	-0.84386
H	-1.28026	-2.69264	-0.39974	H	-1.20696	-2.58579	1.01244
H	-1.08957	-1.91731	-1.95546	H	-1.35636	-3.04481	-0.66858
H	0.34751	-2.46592	-1.06825	H	0.24894	-3.03032	0.09334
H	-3.70638	1.52162	0.7109	H	-3.44187	1.59203	-0.36836
H	-4.13493	1.51257	-0.99509	H	-4.17121	0.63203	-1.64652
H	-5.22792	-1.02633	-1.1196	H	-5.26573	-1.36407	-0.02215
H	-4.76719	-1.41225	1.33604	H	-4.37072	-0.23962	2.04738
H	-6.43259	-0.93077	1.70384	H	-5.95129	0.52966	2.27345
H	-5.07246	0.00761	2.35437	H	-4.50563	1.52867	2.01868
H	-6.14713	2.11557	1.27213	H	-7.14727	1.60302	0.33337

H	-6.47469	2.11754	-0.47429	H	-5.75215	2.66031	0.03976
H	-7.4489	1.10604	0.61154	H	-6.39071	1.64655	-1.27224

atom	Con f. 4- 4q			atom	Con f. 4- 4r		
C	-3.89146	3.25524	-1.02919	C	4.16729	3.11289	0.68843
C	-5.08533	-1.85776	-0.96908	C	3.99542	-1.85522	2.31668
C	-4.05343	-1.45688	-0.21569	C	3.38652	-1.57398	1.15745
C	-3.23386	-0.25084	-0.65776	C	2.80838	-0.17983	0.94257
C	-3.44405	1.05937	0.2039	C	3.61205	0.73438	-0.06805
C	-3.10374	2.22832	-0.70546	C	3.30045	2.17308	0.30737
C	-1.73409	-0.58363	-0.86401	C	1.28905	-0.18369	0.63718
C	-0.86161	-0.52376	0.41585	C	0.91669	-0.4443	-0.82734
C	-1.02116	0.8406	1.08844	C	1.64119	0.56896	-1.74347
C	-2.49059	1.09969	1.42762	C	3.15515	0.50423	-1.53268
C	-3.69644	-2.21845	1.03683	C	3.25552	-2.64262	0.10067
C	-4.88825	1.15691	0.70669	C	5.11592	0.45562	0.02452
C	0.54964	-0.93977	0.06775	C	-0.56487	-0.44238	-1.13375
C	1.56572	-0.0413	0.04124	C	-1.47529	0.05031	-0.25646
C	0.72436	-2.40002	-0.25102	C	-0.93466	-1.01052	-2.47777
C	2.95501	-0.32935	-0.30013	C	-2.91718	0.12791	-0.46831
C	3.9169	0.84747	-0.26499	C	-3.73457	0.7185	0.66984
O	3.35883	-1.45854	-0.63343	O	-3.47698	-0.23118	-1.52038
C	5.38444	0.47767	0.03461	C	-5.19425	0.22449	0.74478
O	5.86565	-0.438	-0.96808	O	-5.87408	0.54273	-0.48495
C	6.26253	1.7215	-0.07136	C	-5.24904	-1.28889	0.99106
C	5.51689	-0.15922	1.42421	C	-5.93867	0.97416	1.84625
H	-3.62066	-0.00081	-1.65177	H	2.91606	0.31505	1.91372
H	-1.2304	-1.28351	1.11537	H	1.27527	-1.43739	-1.12132
H	-3.53836	4.03729	-1.69687	H	3.82666	4.11475	0.93809
H	-4.90475	3.36546	-0.6544	H	5.23554	2.93537	0.76907
H	-5.69322	-2.71609	-0.69218	H	4.41964	-2.83708	2.51367
H	-5.35716	-1.34373	-1.88773	H	4.08981	-1.1156	3.10795
H	-2.10465	2.20539	-1.13701	H	2.24883	2.44921	0.26053
H	-1.30997	0.13021	-1.57929	H	0.89118	0.79419	0.9281
H	-1.65764	-1.56978	-1.33246	H	0.80061	-0.91935	1.28585
H	-0.42	0.87985	2.00391	H	1.41429	0.36165	-2.79392
H	-0.64508	1.63264	0.43174	H	1.2585	1.57418	-1.53216
H	-2.5935	2.07895	1.90961	H	3.64738	1.24918	-2.16928
H	-2.82308	0.35681	2.16136	H	3.51354	-0.47555	-1.86737
H	-4.44922	-2.98227	1.25202	H	3.81387	-3.53867	0.38688
H	-2.73053	-2.72578	0.93302	H	2.20948	-2.93725	-0.04116
H	-3.61594	-1.56374	1.90977	H	3.62547	-2.30986	-0.87384
H	-5.6081	1.12507	-0.11688	H	5.67366	1.13116	-0.63231
H	-5.12066	0.3319	1.38449	H	5.4903	0.58597	1.04443
H	-5.04093	2.0913	1.25649	H	5.34058	-0.56855	-0.28314
H	1.36175	0.99221	0.29576	H	-1.13583	0.42659	0.70112
H	1.48338	-2.85042	0.39597	H	-1.68459	-1.80018	-2.37387
H	-0.21446	-2.94347	-0.12271	H	-1.38431	-0.24739	-3.12156
H	1.07676	-2.54457	-1.27737	H	-0.05798	-1.42161	-2.98342
H	3.86664	1.31897	-1.25722	H	-3.22483	0.54006	1.62136
H	3.56136	1.59352	0.45183	H	-3.73672	1.80748	0.51648
H	5.1777	-1.12976	-1.01487	H	-5.27474	0.21576	-1.18349
H	5.97105	2.47117	0.67028	H	-6.29109	-1.62052	1.03784
H	6.17882	2.16706	-1.06789	H	-4.75991	-1.55243	1.93476
H	7.31087	1.45591	0.10041	H	-4.75325	-1.83599	0.18391
H	5.17768	0.52791	2.20655	H	-6.98576	0.65521	1.87479
H	4.92396	-1.07593	1.49303	H	-5.49162	0.77757	2.8253
H	6.56348	-0.41407	1.61849	H	-5.91217	2.05299	1.66194

atom	Con f. 4- 5a			atom	Con f. 4- 5b		
C	5.01339	-2.17292	-0.14361	C	4.84607	-2.08586	-0.66142
C	3.23815	1.88181	2.18494	C	2.69868	0.75445	2.88108
C	3.49247	1.35307	0.98173	C	3.15705	0.74415	1.62338
C	2.4522	0.48476	0.29418	C	2.24779	0.31575	0.48424
C	2.76526	-1.05612	0.30554	C	2.55613	-1.10585	-0.11216
C	3.98815	-1.40386	-0.51772	C	3.89551	-1.16134	-0.81752
C	2.13119	0.99479	-1.12328	C	2.16375	1.39381	-0.61248
C	0.89328	0.2893	-1.72982	C	1.03761	1.09559	-1.63429
C	1.12143	-1.23094	-1.70358	C	1.25362	-0.30974	-2.21997
C	1.53301	-1.76733	-0.3259	C	1.44116	-1.40173	-1.15779
C	4.7935	1.63362	0.27309	C	4.56266	1.18861	1.30762
C	2.89837	-1.54125	1.75321	C	2.4558	-2.15894	0.99658
C	-0.39504	0.8	-1.10634	C	-0.32972	1.38994	-1.04162
C	-1.31609	-0.03708	-0.56777	C	-1.31809	0.46087	-1.01324
C	-0.59001	2.29019	-1.18275	C	-0.50678	2.79125	-0.52405

C	-2.58936	0.34353	0.03983	C	-2.65507	0.63333	-0.45104
C	-3.52633	-0.79927	0.39719	C	-3.65348	-0.48143	-0.71697
O	-2.90625	1.51778	0.30052	O	-3.00031	1.62879	0.21168
C	-5.02463	-0.43272	0.39581	C	-4.66362	-0.72072	0.42611
O	-5.26389	0.6244	1.34509	O	-5.43001	0.47787	0.65124
C	-5.47964	0.01208	-1.00055	C	-5.66728	-1.79416	0.0136
C	-5.8488	-1.62528	0.8733	C	-3.94217	-1.12484	1.71858
H	1.53186	0.58103	0.88448	H	1.24228	0.21924	0.91347
H	0.83536	0.60901	-2.78104	H	1.15145	1.82729	-2.44777
H	5.82612	-2.39052	-0.83208	H	5.75845	-2.05087	-1.25152
H	5.09217	-2.61098	0.84698	H	4.76474	-2.90329	0.04912
H	3.96103	2.5156	2.69351	H	3.32324	1.06963	3.71386
H	2.29898	1.69963	2.70166	H	1.68323	0.44816	3.12029
H	3.99965	-1.01234	-1.53293	H	4.07195	-0.38241	-1.55651
H	2.97757	0.82582	-1.79465	H	3.10705	1.46324	-1.16132
H	1.98054	2.07688	-1.08881	H	2.012	2.3697	-0.1438
H	0.23096	-1.7611	-2.05762	H	0.42729	-0.58042	-2.88605
H	1.91148	-1.45497	-2.42853	H	2.14736	-0.26557	-2.85199
H	1.74761	-2.83954	-0.40128	H	1.66655	-2.35408	-1.65143
H	0.701	-1.66207	0.3783	H	0.50838	-1.5485	-0.60363
H	4.62369	2.11528	-0.69703	H	5.08442	1.51203	2.2128
H	5.43077	2.29228	0.87006	H	5.14103	0.38273	0.84544
H	5.3452	0.71044	0.07139	H	4.5665	2.02318	0.59677
H	3.78214	-1.12554	2.24422	H	3.25049	-2.04729	1.73896
H	2.02006	-1.23762	2.33197	H	1.49669	-2.0662	1.51634
H	2.96494	-2.63321	1.79806	H	2.5146	-3.17139	0.58398
H	-1.13818	-1.10508	-0.59354	H	-1.13669	-0.51324	-1.44883
H	-1.63303	2.54806	-1.36973	H	-1.50795	3.17066	-0.7322
H	-0.3157	2.76455	-0.23244	H	-0.38286	2.81959	0.56553
H	0.03767	2.72512	-1.96457	H	0.23586	3.46335	-0.9612
H	-3.34421	-1.64754	-0.26929	H	-4.20917	-0.19382	-1.62086
H	-3.24334	-1.12895	1.40744	H	-3.12559	-1.40934	-0.95308
H	-4.59135	1.29921	1.13092	H	-4.76213	1.18945	0.69738
H	-6.54482	0.26321	-0.98212	H	-6.41445	-1.93046	0.80247
H	-5.32495	-0.78296	-1.7375	H	-5.16858	-2.75284	-0.15727
H	-4.92651	0.89664	-1.32958	H	-6.18623	-1.50274	-0.90524
H	-5.73723	-2.47559	0.19392	H	-4.6731	-1.29374	2.51562
H	-6.90895	-1.3546	0.91576	H	-3.25591	-0.33883	2.04792
H	-5.5322	-1.93595	1.87422	H	-3.36608	-2.04539	1.57744

atom	Con f. 4- 5c			atom	Con f. 4- 5d		
C	4.44243	-2.47951	-0.43135	C	5.55818	0.02775	0.58251
C	4.50337	1.97396	0.03253	C	1.64775	3.09025	0.17144
C	3.37186	1.66048	0.67621	C	2.29582	2.10325	-0.46024
C	2.42042	0.57077	0.20259	C	1.96239	0.65389	-0.1473
C	2.78035	-0.88344	0.6833	C	3.01619	-0.09535	0.74578
C	4.1272	-1.31599	0.1401	C	4.35846	-0.23349	0.05827
C	2.21999	0.58778	-1.32203	C	1.60979	-0.13547	-1.42777
C	1.02796	-0.30079	-1.75636	C	1.0842	-1.55293	-1.15295
C	1.25672	-1.73059	-1.24028	C	2.10812	-2.30242	-0.26384
C	1.62234	-1.80827	0.24981	C	2.44438	-1.51988	1.0063
C	2.93533	2.43324	1.89458	C	3.33255	2.40932	-1.51136
C	2.88659	-0.93315	2.22104	C	3.14122	0.61979	2.09545
C	-0.29207	0.35986	-1.40301	C	-0.32713	-1.65584	-0.59822
C	-1.21612	-0.24726	-0.61798	C	-1.10003	-0.56088	-0.39111
C	-0.50167	1.71875	-2.0155	C	-0.8134	-3.05877	-0.35387
C	-2.50402	0.30644	-0.20626	C	-2.46214	-0.53985	0.1374
C	-3.41535	-0.6369	0.56274	C	-3.15688	0.8125	0.11497
O	-2.8464	1.48379	-0.41425	O	-3.02908	-1.53286	0.62611
C	-4.91331	-0.27381	0.53133	C	-4.69704	0.76027	0.14752
O	-5.10007	1.05301	1.06151	O	-5.13166	0.05901	1.32906
C	-5.46717	-0.34406	-0.89781	C	-5.25983	2.17412	0.26424
C	-5.69121	-1.2096	1.45266	C	-5.25029	0.06322	-1.1025
H	1.44686	0.78606	0.66274	H	1.06654	0.68059	0.4797
H	1.04118	-0.32935	-2.85585	H	1.04351	-2.09184	-2.11144
H	5.46543	-2.69288	-0.73156	H	6.46533	-0.13755	0.00664
H	3.71549	-3.2652	-0.61602	H	5.69138	0.41329	1.58892
H	5.15185	2.77002	0.39052	H	1.85613	4.13676	-0.03919
H	4.82019	1.46277	-0.87129	H	0.88817	2.88468	0.92173
H	4.92409	-0.59186	0.30042	H	4.32938	-0.61529	-0.96002
H	3.11994	0.21043	-1.81887	H	2.49606	-0.23221	-2.06223
H	2.08242	1.61659	-1.66304	H	0.88348	0.43574	-2.01587
H	0.38048	-2.35702	-1.44057	H	1.73887	-3.29624	0.00291
H	2.07485	-2.15336	-1.83083	H	3.01472	-2.46103	-0.85694
H	1.86706	-2.84426	0.51014	H	3.16687	-2.08326	1.60785
H	0.75401	-1.54218	0.86097	H	1.53734	-1.41307	1.61587

H	1.98976	2.95455	1.69366	H	4.30486	1.98028	-1.25062
H	3.67979	3.17795	2.19096	H	3.05334	1.9859	-2.48324
H	2.74755	1.7738	2.74833	H	3.4558	3.48876	-1.63708
H	3.73964	-0.35473	2.58811	H	2.15327	0.73377	2.55319
H	1.97733	-0.53875	2.68814	H	3.57801	1.61632	1.98962
H	3.01924	-1.96811	2.55397	H	3.76395	0.04523	2.78863
H	-1.02187	-1.24731	-0.24977	H	-0.70904	0.41734	-0.643
H	-0.32259	2.50918	-1.27665	H	-0.70546	-3.32646	0.70456
H	0.18456	1.87853	-2.85065	H	-1.87169	-3.16437	-0.59488
H	-1.52848	1.84656	-2.36203	H	-0.23406	-3.77801	-0.93841
H	-3.2655	-1.66042	0.20466	H	-2.79811	1.35712	1.00076
H	-3.06551	-0.6212	1.60536	H	-2.81514	1.38307	-0.75449
H	-4.46378	1.60255	0.56548	H	-4.6233	-0.77431	1.31545
H	-4.94151	0.35184	-1.55793	H	-6.35269	2.13915	0.32223
H	-6.52904	-0.07855	-0.89778	H	-4.97981	2.77983	-0.60275
H	-5.36375	-1.35291	-1.31102	H	-4.88223	2.66431	1.16739
H	-5.61053	-2.24722	1.11534	H	-6.34333	0.02958	-1.05555
H	-6.7502	-0.9314	1.46235	H	-4.87991	-0.9633	-1.17702
H	-5.30893	-1.146	2.47661	H	-4.96031	0.59904	-2.01244
atom	Con f. 4- 5e			atom	Con f. 4- 5f		
C	-5.36342	-0.23337	0.96576	C	4.94954	-1.90992	-0.69176
C	-1.44843	-3.05309	-0.36729	C	4.47475	1.09081	1.39714
C	-2.20725	-2.01717	-0.74708	C	3.21588	0.68441	1.59685
C	-1.87251	-0.61542	-0.26601	C	2.27736	0.31205	0.45857
C	-2.82278	-0.04278	0.84589	C	2.58877	-1.0833	-0.1943
C	-4.2416	0.15114	0.35241	C	3.92529	-1.08134	-0.90444
C	-1.69837	0.36208	-1.45027	C	2.17338	1.42868	-0.59529
C	-1.1762	1.74752	-1.03855	C	1.0426	1.1577	-1.6202
C	-2.0857	2.31999	0.07848	C	1.25973	-0.22502	-2.25773
C	-2.24447	1.34543	1.24693	C	1.4701	-1.35498	-1.23921
C	-3.37289	-2.2153	-1.68259	C	2.62816	0.59924	2.9811
C	-2.7703	-0.95376	2.07733	C	2.52112	-2.17685	0.87748
C	0.29439	1.83766	-0.66831	C	-0.32173	1.42331	-1.00758
C	1.1111	0.7542	-0.69141	C	-1.30248	0.48584	-0.99877
C	0.77692	3.22023	-0.32344	C	-0.50522	2.80709	-0.4468
C	2.53344	0.72669	-0.36002	C	-2.63794	0.63179	-0.42555
C	3.26735	-0.57059	-0.659	C	-3.63	-0.47948	-0.72768
O	3.14806	1.69195	0.12899	O	-2.9864	1.60314	0.27027
C	4.45277	-0.87358	0.28147	C	-4.65912	-0.74454	0.39188
O	5.42427	0.18628	0.18908	O	-5.42882	0.44908	0.63216
C	3.97782	-1.01709	1.7335	C	-5.65593	-1.807	-0.06334
C	5.16439	-2.14346	-0.17725	C	-3.96046	-1.18017	1.68662
H	-0.90596	-0.69557	0.23974	H	1.27678	0.1963	0.89613
H	-1.28035	2.41899	-1.90395	H	1.14591	1.91816	-2.40844
H	-6.33677	-0.01323	0.53442	H	5.85859	-1.8349	-1.2834
H	-5.36657	-0.77748	1.90548	H	4.93371	-2.68675	0.0671
H	-1.65371	-4.066	-0.70615	H	5.11993	1.35546	2.2316
H	-0.59692	-2.92321	0.29647	H	4.90705	1.17301	0.40524
H	-4.34463	0.69015	-0.58694	H	4.03993	-0.33598	-1.68872
H	-2.65821	0.51033	-1.95418	H	3.11593	1.52607	-1.14161
H	-1.03653	-0.08942	-2.1972	H	2.01677	2.38401	-0.08862
H	-1.69796	3.27381	0.4461	H	0.42575	-0.47736	-2.92144
H	-3.06368	2.53716	-0.36348	H	2.14274	-0.15163	-2.90167
H	-2.89227	1.78846	2.01201	H	1.70047	-2.28538	-1.77138
H	-1.26508	1.18001	1.71524	H	0.5441	-1.53395	-0.683
H	-3.24725	-1.63781	-2.60605	H	3.35669	0.86391	3.75319
H	-3.48114	-3.26875	-1.95606	H	1.76709	1.27453	3.07486
H	-4.31019	-1.87928	-1.22887	H	2.25258	-0.40932	3.1905
H	-3.20093	-1.93788	1.8741	H	3.30702	-2.05732	1.62864
H	-1.73213	-1.10071	2.3923	H	1.55416	-2.14413	1.38937
H	-3.31275	-0.51029	2.91848	H	2.62949	-3.17112	0.43149
H	0.70743	-0.20327	-0.99454	H	-1.11658	-0.47319	-1.46485
H	0.8362	3.34988	0.76425	H	-1.51053	3.18526	-0.63695
H	1.78005	3.40068	-0.71193	H	-0.37472	2.80382	0.6424
H	0.09382	3.97921	-0.71281	H	0.2297	3.49756	-0.86806
H	2.56137	-1.40596	-0.65674	H	-4.16997	-0.17586	-1.63601
H	3.64701	-0.48323	-1.68721	H	-3.09533	-1.40114	-0.97358
H	4.90094	1.00498	0.28833	H	-4.76081	1.15732	0.7128
H	4.83136	-1.23126	2.38437	H	-6.4155	-1.96317	0.70983
H	3.25446	-1.83328	1.83265	H	-5.15428	-2.76103	-0.25078
H	3.50455	-0.09557	2.08506	H	-6.1601	-1.49202	-0.98262
H	4.49621	-3.00801	-0.12244	H	-4.70533	-1.36687	2.46662
H	6.03259	-2.33785	0.46109	H	-3.27888	-0.40357	2.04646
H	5.51294	-2.03755	-1.20964	H	-3.38338	-2.0982	1.53349
atom	Con f. 4- 5g			atom	Con f. 4- 5h		

C	-4.90247	2.15563	-0.41826	C	-5.16424	-0.80564	-1.31866
C	-2.91526	-1.21254	2.69012	C	-2.18835	3.04872	-0.26783
C	-3.29686	-1.00663	1.42362	C	-2.76685	1.96349	0.26131
C	-2.33141	-0.38951	0.42591	C	-2.07644	0.61281	0.17448
C	-2.6253	1.11282	0.06537	C	-2.68752	-0.37884	-0.88238
C	-3.92802	1.28411	-0.68886	C	-4.09384	-0.80987	-0.52082
C	-2.17447	-1.26529	-0.83132	C	-1.93263	-0.04296	1.56116
C	-1.00835	-0.78866	-1.7318	C	-0.99327	-1.27293	1.52467
C	-1.21861	0.69357	-2.08152	C	-1.53209	-2.2691	0.48514
C	-1.46543	1.58659	-0.85785	C	-1.77804	-1.64263	-0.89407
C	-4.67119	-1.41118	0.95398	C	-4.08874	2.06385	0.9797
C	-2.59233	1.96176	1.34065	C	-2.63019	0.25697	-2.27547
C	0.33953	-1.17079	-1.14265	C	0.46194	-0.84775	1.38198
C	1.32467	-0.25904	-0.94751	C	1.26001	-1.37164	0.42277
C	0.50771	-2.63598	-0.84414	C	0.92129	0.13401	2.42972
C	2.6603	-0.52327	-0.41638	C	2.66184	-1.09191	0.09824
C	3.67728	0.59425	-0.5799	C	3.36487	0.18027	0.53157
O	2.98714	-1.59526	0.12325	O	3.2657	-1.92036	-0.60826
C	4.81092	0.60582	0.46569	C	4.45167	0.67069	-0.45207
O	5.52585	-0.64408	0.41622	O	5.46921	-0.33637	-0.5993
C	5.82756	1.68839	0.11322	C	3.8462	1.00409	-1.82207
C	4.25373	0.82906	1.87768	C	5.14953	1.89501	0.13518
H	-1.35142	-0.36502	0.91973	H	-1.06022	0.81004	-0.19083
H	-1.07409	-1.37218	-2.66233	H	-1.0542	-1.74528	2.51612
H	-5.78248	2.22106	-1.05325	H	-6.12354	-1.17484	-0.96467
H	-4.87295	2.82934	0.43297	H	-5.13805	-0.43909	-2.34057
H	-3.58334	-1.66232	3.42107	H	-2.6537	4.02996	-0.2088
H	-1.92234	-0.93891	3.03863	H	-1.23037	2.99216	-0.77891
H	-4.04958	0.65331	-1.56689	H	-4.22183	-1.19943	0.48695
H	-3.09018	-1.25424	-1.42903	H	-1.57034	0.70199	2.27414
H	-2.02513	-2.3039	-0.52497	H	-2.90523	-0.37382	1.93628
H	-0.37021	1.07781	-2.65749	H	-0.86198	-3.12974	0.38726
H	-2.08406	0.75071	-2.75075	H	-2.47513	-2.66743	0.87529
H	-1.67774	2.60927	-1.19031	H	-2.22652	-2.38978	-1.55893
H	-0.56002	1.63776	-0.24365	H	-0.82354	-1.35358	-1.34628
H	-5.24868	-1.85743	1.76867	H	-4.85348	1.45005	0.49405
H	-5.22814	-0.55323	0.56566	H	-4.00989	1.70584	2.01284
H	-4.61493	-2.14266	0.13921	H	-4.44308	3.09827	1.0059
H	-3.41739	1.71716	2.01464	H	-3.29178	1.12334	-2.35692
H	-1.65608	1.79075	1.88191	H	-1.61117	0.59261	-2.49371
H	-2.64944	3.0291	1.10365	H	-2.91556	-0.46426	-3.04817
H	1.15326	0.77134	-1.23201	H	0.86379	-2.15242	-0.21726
H	1.52233	-2.97433	-1.0573	H	0.32332	0.01947	3.33809
H	0.32796	-2.83698	0.21917	H	1.96832	-0.00167	2.69827
H	-0.20456	-3.2334	-1.41914	H	0.79154	1.16828	2.08917
H	4.11808	0.46368	-1.57902	H	2.63611	0.97604	0.69383
H	3.16397	1.5603	-0.59302	H	3.84064	-0.02146	1.50093
H	4.82683	-1.32481	0.45586	H	4.97094	-1.15414	-0.79148
H	6.65561	1.67286	0.82952	H	3.38077	0.12295	-2.27296
H	5.36783	2.68082	0.13961	H	4.63135	1.35758	-2.49776
H	6.23592	1.5207	-0.88864	H	3.08579	1.78761	-1.73715
H	5.07241	0.83157	2.60418	H	4.44486	2.72097	0.27019
H	3.55455	0.03465	2.1549	H	5.94754	2.22867	-0.53616
H	3.72806	1.78729	1.94635	H	5.59447	1.65498	1.10625
atom	Con f. 4- 5i			atom	Con f. 4- 5j		
C	5.30736	-0.88426	0.00214	C	-5.01344	2.17283	-0.14389
C	2.96939	2.61795	-1.52015	C	-3.23812	-1.88177	2.18502
C	2.20529	2.2252	-0.49323	C	-3.49244	-1.35309	0.98178
C	1.9581	0.7632	-0.1461	C	-2.45219	-0.48478	0.29422
C	3.05997	0.08835	0.74996	C	-2.76535	1.0561	0.30554
C	4.41639	0.10617	0.07444	C	-3.98818	1.40373	-0.51783
C	1.66812	-0.08924	-1.3987	C	-2.13112	-0.99479	-1.12321
C	1.19693	-1.51543	-1.07574	C	-0.89323	-0.28925	-1.72978
C	2.24432	-2.18713	-0.15289	C	-1.1214	1.23099	-1.70349
C	2.56231	-1.33613	1.07891	C	-1.53307	1.76733	-0.32581
C	1.47311	3.23368	0.3555	C	-4.7935	-1.63365	0.2732
C	3.21587	0.86852	2.07145	C	-2.89861	1.54125	1.75318
C	-0.21646	-1.65732	-0.53572	C	0.3951	-0.79996	-1.10636
C	-1.0399	-0.58978	-0.38607	C	1.31607	0.03709	-0.56762
C	-0.64518	-3.06851	-0.23751	C	0.59015	-2.29013	-1.18295
C	-2.40717	-0.60358	0.12812	C	2.58938	-0.34356	0.03987
C	-3.14565	0.72441	0.06953	C	3.5263	0.7992	0.39742
O	-2.94615	-1.60322	0.63517	O	2.90637	-1.51786	0.30024
C	-4.68353	0.62362	0.09114	C	5.02464	0.43273	0.39586
O	-5.10583	-0.06886	1.28219	O	5.26404	-0.62454	1.34489

C	-5.29175	2.02086	0.17705	C	5.47953	-0.01173	-1.00065
C	-5.20366	-0.11379	-1.14985	C	5.84877	1.62524	0.87351
H	1.06322	0.74782	0.48385	H	-1.53187	-0.58096	0.88455
H	1.19012	-2.09145	-2.01347	H	-0.83538	-0.60891	-2.781
H	6.27823	-0.72642	-0.46123	H	-5.82614	2.39033	-0.83242
H	5.12284	-1.87783	0.40045	H	-5.09229	2.61101	0.84664
H	3.10699	3.67241	-1.74716	H	-3.96099	-2.51555	2.69362
H	3.47598	1.91377	-2.17287	H	-2.29896	-1.69955	2.70173
H	4.69406	1.0749	-0.33688	H	-3.99962	1.01208	-1.53299
H	2.58407	-0.17339	-1.99362	H	-2.9775	-0.82589	-1.79459
H	0.93915	0.42503	-2.03265	H	-1.9804	-2.07687	-1.08871
H	1.9118	-3.18051	0.15991	H	-0.23092	1.76119	-2.05746
H	3.15214	-2.33353	-0.74415	H	-1.9114	1.45503	-2.42849
H	3.30424	-1.8488	1.70152	H	-1.74766	2.83955	-0.40115
H	1.65923	-1.24091	1.6955	H	-0.70111	1.66203	0.37843
H	1.71387	4.26093	0.06637	H	-4.62377	-2.11514	-0.69702
H	0.38782	3.09572	0.25782	H	-5.43068	-2.29243	0.87012
H	1.70329	3.11166	1.41925	H	-5.34528	-0.71047	0.07169
H	3.8953	0.33457	2.74422	H	-3.78239	1.12549	2.24413
H	2.24955	0.97715	2.57656	H	-2.02033	1.23768	2.33203
H	3.63013	1.86752	1.90609	H	-2.96526	2.6332	1.79799
H	-0.68696	0.39281	-0.67409	H	1.13811	1.10508	-0.59323
H	-0.02528	-3.78615	-0.7809	H	1.63319	-2.54789	-1.36998
H	-0.54323	-3.2848	0.83333	H	0.31588	-2.76466	-0.23272
H	-1.69385	-3.22986	-0.48894	H	-0.03748	-2.725	-1.96484
H	-2.81227	1.29896	0.94638	H	3.34409	1.64763	-0.26884
H	-2.81508	1.2873	-0.80922	H	3.24339	1.12865	1.40777
H	-4.56926	-0.8845	1.29	H	4.59157	-1.29939	1.13061
H	-6.3834	1.95204	0.22679	H	6.54471	-0.26286	-0.98238
H	-5.02363	2.61926	-0.69875	H	5.32477	0.78348	-1.7374
H	-4.93765	2.53914	1.07399	H	4.92639	-0.89622	-1.32983
H	-6.29555	-0.18039	-1.11185	H	5.73706	2.47571	0.19435
H	-4.80147	-1.12976	-1.20135	H	6.90894	1.35463	0.91579
H	-4.92176	0.41323	-2.06744	H	5.53227	1.93566	1.87455
atom	Con f. 4- 5k			atom	Con f. 4- 5l		
C	5.2525	-0.73974	0.37988	C	-5.35758	-1.11111	-0.72209
C	2.94022	2.53311	-1.64942	C	-2.40545	2.91858	-0.92539
C	2.10399	2.18934	-0.662	C	-2.88838	1.97277	-0.1102
C	1.87573	0.74936	-0.22238	C	-2.16874	0.64204	0.02588
C	2.90957	0.19152	0.82168	C	-2.86519	-0.56766	-0.69854
C	4.32142	0.20961	0.27089	C	-4.20268	-0.91915	-0.08047
C	1.73883	-0.21	-1.42334	C	-1.83644	0.31954	1.49503
C	1.28804	-1.62547	-1.03207	C	-0.86105	-0.87649	1.61181
C	2.2588	-2.18005	0.03979	C	-1.49052	-2.08813	0.90568
C	2.42991	-1.22103	1.22061	C	-1.91893	-1.79434	-0.53836
C	1.26928	3.2298	0.04096	C	-4.12753	2.21967	0.71263
C	2.91702	1.07848	2.08395	C	-2.99494	-0.26209	-2.19464
C	-0.16833	-1.78953	-0.63135	C	0.54315	-0.48264	1.16995
C	-1.04104	-0.75056	-0.65221	C	1.24228	-1.23021	0.28659
C	-0.5734	-3.19295	-0.27177	C	1.08943	0.73805	1.86769
C	-2.46141	-0.79505	-0.31468	C	2.58501	-1.01701	-0.26773
C	-3.27026	0.44329	-0.66626	C	3.11684	0.36359	-0.59706
O	-3.01605	-1.7763	0.21252	O	3.25029	-2.01495	-0.59701
C	-4.50842	0.68657	0.22108	C	4.63502	0.53551	-0.36191
O	-5.39407	-0.4464	0.13301	O	5.35751	-0.3795	-1.20705
C	-4.09975	0.91327	1.68252	C	5.0644	1.93729	-0.7859
C	-5.29648	1.88202	-0.30713	C	5.00176	0.27847	1.10523
H	0.92476	0.74509	0.32018	H	-1.21104	0.75671	-0.49792
H	1.39742	-2.27182	-1.91619	H	-0.77074	-1.1092	2.68296
H	6.25626	-0.58192	-0.00705	H	-6.25387	-1.40135	-0.17956
H	5.0681	-1.69859	0.85594	H	-5.46484	-0.98772	-1.79571
H	3.06198	3.57199	-1.94646	H	-2.89373	3.88476	-1.03068
H	3.52333	1.80203	-2.20081	H	-1.50598	2.75898	-1.51504
H	4.60033	1.14676	-0.20744	H	-4.19712	-1.07017	0.99702
H	2.71108	-0.3023	-1.91944	H	-2.74366	0.07154	2.05321
H	1.0592	0.2214	-2.16474	H	-1.41982	1.20931	1.97365
H	1.92741	-3.1567	0.40237	H	-0.81078	-2.94662	0.92351
H	3.22465	-2.33935	-0.4473	H	-2.36617	-2.38841	1.4914
H	3.12543	-1.65232	1.94953	H	-2.41509	-2.67626	-0.95948
H	1.4697	-1.11248	1.74146	H	-1.03363	-1.60663	-1.15471
H	1.41058	3.19725	1.12646	H	-3.92259	2.11179	1.78426
H	1.50132	4.24075	-0.30675	H	-4.51729	3.22734	0.54262
H	0.20138	3.0435	-0.13635	H	-4.9152	1.49936	0.47175
H	3.31159	2.07739	1.87549	H	-3.68929	0.56072	-2.3843
H	1.90483	1.18712	2.48936	H	-2.02068	0.02065	-2.60662

H	3.54842	0.62625	2.85605	H	-3.34712	-1.14007	-2.74567
H	-0.69105	0.22265	-0.9713	H	0.81882	-2.16867	-0.05692
H	0.12739	-3.91927	-0.69108	H	2.1759	0.72748	1.94228
H	-0.57959	-3.32674	0.81713	H	0.78743	1.65958	1.3562
H	-1.58249	-3.41913	-0.61822	H	0.68572	0.79424	2.88338
H	-2.62047	1.32358	-0.65879	H	2.92384	0.4882	-1.67208
H	-3.60132	0.31016	-1.7065	H	2.5578	1.14756	-0.09059
H	-4.815	-1.21789	0.28591	H	4.88812	-1.23084	-1.11188
H	-4.99053	1.08156	2.29592	H	6.1485	2.04473	-0.67563
H	-3.44495	1.78593	1.77775	H	4.57847	2.69913	-0.16922
H	-3.57066	0.04298	2.08188	H	4.8054	2.1187	-1.83385
H	-4.69784	2.79671	-0.26211	H	4.49733	0.98717	1.77014
H	-6.19936	2.03073	0.29425	H	6.08218	0.38902	1.24104
H	-5.59842	1.71449	-1.34601	H	4.72386	-0.73543	1.40977
atom	Con f. 4- 5m			atom	Con f. 4- 5n		
C	-5.36342	-0.23373	0.96558	C	-4.87094	-1.6356	-0.58045
C	-1.44889	-3.05315	-0.36702	C	-3.73425	2.36639	1.06289
C	-2.20736	-2.01709	-0.74711	C	-2.67801	2.09597	0.28575
C	-1.87253	-0.61537	-0.26598	C	-2.08023	0.70265	0.15007
C	-2.82278	-0.04275	0.84594	C	-2.76662	-0.21792	-0.92593
C	-4.24161	0.15121	0.35249	C	-4.21907	-0.47158	-0.57588
C	-1.69835	0.36216	-1.45021	C	-1.99432	-0.03433	1.4977
C	-1.17613	1.74758	-1.03846	C	-1.10699	-1.29976	1.40551
C	-2.08561	2.32005	0.0786	C	-1.67654	-2.21948	0.31423
C	-2.24445	1.34546	1.24701	C	-1.92832	-1.5115	-1.0251
C	-3.37268	-2.21497	-1.68306	C	-1.96495	3.19408	-0.46141
C	-2.77029	-0.95372	2.07739	C	-2.75791	0.46902	-2.30661
C	0.29447	1.83769	-0.66825	C	0.36354	-0.92177	1.30061
C	1.11114	0.75419	-0.69134	C	1.13949	-1.3809	0.29089
C	0.77704	3.22025	-0.32343	C	0.85444	-0.05466	2.432
C	2.53349	0.72669	-0.36002	C	2.54963	-1.12831	-0.01847
C	3.26751	-0.57046	-0.65921	C	3.32243	0.03683	0.56898
O	3.14806	1.69194	0.1291	O	3.10372	-1.88859	-0.83441
C	4.45273	-0.87368	0.28143	C	4.46221	0.56892	-0.32858
O	5.42424	0.18625	0.18956	O	5.41076	-0.48128	-0.59208
C	3.97748	-1.01755	1.73332	C	3.91108	1.11757	-1.65129
C	5.16449	-2.14342	-0.17749	C	5.23063	1.65459	0.42065
H	-0.90598	-0.69558	0.23978	H	-1.05354	0.84074	-0.21477
H	-1.2803	2.41908	-1.90384	H	-1.19788	-1.81973	2.37038
H	-6.33676	-0.01354	0.53428	H	-5.93519	-1.67762	-0.36174
H	-5.36655	-0.77825	1.90507	H	-4.39121	-2.58362	-0.80605
H	-1.65427	-4.06603	-0.70589	H	-4.1243	3.37764	1.14965
H	-0.59756	-2.92341	0.297	H	-4.24073	1.6015	1.64343
H	-4.34464	0.6906	-0.58665	H	-4.78557	0.43063	-0.35175
H	-2.6582	0.51045	-1.9541	H	-2.99512	-0.34819	1.8121
H	-1.03653	-0.08933	-2.19716	H	-1.62492	0.64713	2.26732
H	-1.6978	3.27382	0.44628	H	-1.02668	-3.08755	0.15957
H	-3.06356	2.53732	-0.36337	H	-2.62361	-2.61317	0.69483
H	-2.89226	1.78851	2.01208	H	-2.41325	-2.20676	-1.7196
H	-1.26509	1.18001	1.71535	H	-0.97283	-1.24194	-1.48658
H	-3.48139	-3.26847	-1.95616	H	-2.45952	4.16123	-0.3321
H	-4.31	-1.87822	-1.22993	H	-1.90072	2.98025	-1.53351
H	-3.24626	-1.63794	-2.6067	H	-0.93091	3.28667	-0.10298
H	-3.20088	-1.93786	1.87418	H	-3.399	1.35543	-2.32329
H	-1.73212	-1.10064	2.39236	H	-1.74263	0.77399	-2.58359
H	-3.31278	-0.51024	2.91851	H	-3.12701	-0.22223	-3.07175
H	0.70743	-0.20327	-0.99447	H	0.70953	-2.07906	-0.41924
H	0.83651	3.34992	0.76423	H	0.23036	-0.20258	3.31735
H	1.78013	3.4007	-0.71207	H	1.88607	-0.27225	2.70722
H	0.09392	3.97922	-0.71275	H	0.79398	1.00909	2.17193
H	2.56157	-1.40588	-0.6573	H	2.64101	0.85417	0.81054
H	3.64736	-0.48281	-1.68732	H	3.75726	-0.30628	1.51771
H	4.90083	1.00492	0.28862	H	4.86102	-1.22889	-0.89657
H	3.25406	-1.83374	1.83213	H	3.3932	0.33715	-2.21603
H	3.50417	-0.09611	2.08501	H	4.73347	1.49503	-2.26705
H	4.83088	-1.23192	2.38431	H	3.20759	1.93808	-1.47488
H	4.49633	-3.008	-0.12302	H	4.58274	2.50549	0.65124
H	6.03256	-2.33792	0.46097	H	6.06486	2.01215	-0.1917
H	5.51322	-2.03722	-1.20979	H	5.63581	1.26118	1.3585
atom	Con f. 4- 5o			atom	Con f. 4- 5p		
C	4.44284	-2.47897	-0.43149	C	-5.55903	-0.02725	0.5796
C	4.50304	1.97427	0.03279	C	-1.64632	-3.08969	0.17492
C	3.3716	1.66056	0.67648	C	-2.29521	-2.10378	-0.4576
C	2.4203	0.5708	0.2027	C	-1.96247	-0.6539	-0.14646
C	2.78041	-0.88335	0.68333	C	-3.01715	0.09635	0.74464

C	4.12737	-1.31564	0.14018	C	-4.35893	0.23327	0.05585
C	2.21995	0.58786	-1.32193	C	-1.60903	0.13375	-1.42777
C	1.02805	-0.30084	-1.7564	C	-1.08348	1.55147	-1.15404
C	1.25693	-1.73063	-1.24034	C	-2.1085	2.3021	-0.26729
C	1.62257	-1.80834	0.24974	C	-2.44598	1.52142	1.00368
C	2.93504	2.43298	1.89506	C	-3.33247	-2.41163	-1.5077
C	2.88659	-0.93317	2.22108	C	-3.14308	-0.61704	2.09517
C	-0.2921	0.35966	-1.4033	C	0.32721	1.65462	-0.59755
C	-1.21615	-0.24743	-0.61825	C	1.1002	0.55974	-0.39018
C	-0.50191	1.71839	-2.0161	C	0.81284	3.0576	-0.35248
C	-2.5041	0.30626	-0.20675	C	2.46185	0.53856	0.13951
C	-3.41524	-0.63678	0.56279	C	3.15789	-0.81304	0.11423
O	-2.84665	1.48347	-0.41541	O	3.02751	1.53072	0.63141
C	-4.91322	-0.27376	0.53163	C	4.698	-0.75911	0.14628
O	-5.09988	1.05332	1.06119	O	5.13224	-0.06039	1.32944
C	-5.46748	-0.34473	-0.89731	C	5.26242	-2.17264	0.25911
C	-5.69081	-1.20913	1.45366	C	5.24999	-0.05812	-1.1021
H	1.44668	0.78595	0.6628	H	-1.06705	-0.67942	0.48121
H	1.04147	-0.32939	-2.85589	H	-1.04128	2.08924	-2.11313
H	5.4659	-2.69208	-0.73168	H	-6.46576	0.13714	0.00279
H	3.71605	-3.26474	-0.61639	H	-5.69298	-0.41128	1.58648
H	5.15151	2.7703	0.39089	H	-1.85414	-4.13657	-0.0345
H	4.8198	1.46325	-0.87114	H	-0.88655	-2.88286	0.92469
H	4.92409	-0.59135	0.3007	H	-4.32911	0.61354	-0.96299
H	3.11997	0.21065	-1.81875	H	-2.49489	0.22994	-2.06287
H	2.08228	1.61668	-1.66288	H	-0.8825	-0.43825	-2.01482
H	0.38074	-2.35713	-1.44062	H	-1.73986	3.29645	-0.00169
H	2.07506	-2.15332	-1.83093	H	-3.0145	2.45953	-0.86162
H	1.8675	-2.84432	0.50999	H	-3.16929	2.08552	1.60356
H	0.75419	-1.54247	0.86091	H	-1.53959	1.41573	1.61442
H	3.6795	3.17759	2.19167	H	-3.45441	-3.49129	-1.63288
H	2.74717	1.7733	2.7486	H	-4.3051	-1.98376	-1.24623
H	1.9895	2.95438	1.6942	H	-3.05471	-1.98821	-2.47999
H	3.73942	-0.35451	2.58828	H	-3.57962	-1.61379	1.99033
H	1.97719	-0.5391	2.68817	H	-2.15544	-0.73026	2.55377
H	3.01955	-1.96813	2.5539	H	-3.76639	-0.04171	2.7872
H	-1.02178	-1.24737	-0.24979	H	0.70967	-0.41845	-0.64298
H	-0.32372	2.50901	-1.27724	H	0.70085	3.32607	0.70535
H	0.18475	1.87835	-2.85084	H	1.87194	3.16305	-0.58974
H	-1.52856	1.84566	-2.3633	H	0.23549	3.77641	-0.93954
H	-3.26544	-1.66041	0.20502	H	2.80011	-1.35968	0.9992
H	-3.06512	-0.62072	1.60531	H	2.81651	-1.38238	-0.75617
H	-4.46369	1.60261	0.56474	H	4.6226	0.77218	1.31833
H	-4.94204	0.3509	-1.55788	H	6.35526	-2.13657	0.31698
H	-6.52938	-0.07928	-0.8971	H	4.98292	-2.77633	-0.60944
H	-5.36412	-1.35376	-1.31009	H	4.88555	-2.66568	1.16103
H	-5.61023	-2.2469	1.11677	H	6.34303	-0.02352	-1.05555
H	-6.7498	-0.93093	1.46355	H	4.87854	0.96824	-1.17362
H	-5.30821	-1.14508	2.47745	H	4.96014	-0.59173	-2.01338
atom	Con f. 4- 5q			atom	Con f. 4- 5r		
C	4.9919	-2.12041	-0.32559	C	5.20395	-0.60472	1.35072
C	3.01229	1.40327	2.5857	C	2.18615	3.04271	-0.01606
C	3.36098	1.12293	1.32409	C	2.72602	1.90798	-0.47836
C	2.38332	0.41655	0.39979	C	2.04616	0.57599	-0.20694
C	2.70009	-1.10158	0.13831	C	2.70899	-0.28757	0.92787
C	3.98586	-1.30019	-0.63785	C	4.12038	-0.71125	0.57817
C	2.17854	1.19781	-0.91145	C	1.83106	-0.22683	-1.5094
C	1.00114	0.63999	-1.74961	C	0.98894	-1.49769	-1.31768
C	1.22123	-0.86275	-1.98968	C	1.64002	-2.35817	-0.20641
C	1.52439	-1.66086	-0.71451	C	1.83465	-1.56551	1.087
C	4.70875	1.53091	0.78487	C	3.99047	1.93356	-1.29962
C	2.72068	-1.85937	1.47017	C	2.66136	0.4851	2.25077
C	-0.33669	1.05522	-1.15997	C	-0.50101	-1.29686	-1.07293
C	-1.28441	0.15388	-0.80975	C	-1.06201	-0.06542	-1.10812
C	-0.53368	2.54312	-1.03217	C	-1.27029	-2.57756	-0.8733
C	-2.60484	0.45159	-0.23903	C	-2.4503	0.35121	-0.89012
C	-3.5043	-0.76659	-0.10933	C	-3.45189	-0.50293	-0.13635
O	-2.95879	1.58064	0.11957	O	-2.78514	1.478	-1.30014
C	-4.92098	-0.57268	0.44361	C	-4.51832	0.30351	0.63961
O	-5.46995	-1.91267	0.40505	O	-5.27404	1.11934	-0.27412
C	-4.9126	-0.08297	1.89337	C	-5.51837	-0.65979	1.27403
C	-5.76716	0.33399	-0.45334	C	-3.8698	1.18636	1.71421
H	1.41699	0.40838	0.92032	H	1.05773	0.81696	0.19522
H	1.04398	1.1487	-2.72388	H	1.04361	-2.08385	-2.24697
H	5.85679	-2.21369	-0.97763	H	6.16886	-0.97156	1.00971

H	5.00347	-2.72353	0.57739	H	5.18324	-0.15317	2.33808
H	3.68918	1.91742	3.2642	H	2.64554	4.01086	-0.20198
H	2.03773	1.12935	2.98239	H	1.26599	3.04161	0.56311
H	4.06679	-0.73848	-1.56588	H	4.2443	-1.17834	-0.3966
H	2.01538	2.25281	-0.67492	H	2.79613	-0.53492	-1.92296
H	3.08079	1.1617	-1.52842	H	1.37946	0.42485	-2.26487
H	0.35732	-1.29969	-2.50227	H	1.04589	-3.25303	-0.00406
H	2.06283	-0.96167	-2.68414	H	2.60666	-2.71221	-0.5795
H	1.74807	-2.70026	-0.98133	H	2.28739	-2.20736	1.85145
H	0.64041	-1.68638	-0.06878	H	0.85349	-1.25678	1.47164
H	5.27525	0.66473	0.43004	H	4.36132	2.95596	-1.41553
H	4.60634	2.20947	-0.07029	H	4.77941	1.33208	-0.83799
H	5.30025	2.03999	1.55115	H	3.82502	1.51921	-2.30106
H	3.55863	-1.5529	2.10168	H	3.28981	1.37905	2.22463
H	1.79643	-1.67008	2.02577	H	1.63542	0.80257	2.46387
H	2.79472	-2.93923	1.30549	H	2.99438	-0.14247	3.08374
H	-1.09369	-0.90084	-0.96743	H	-0.44146	0.78571	-1.36786
H	0.1713	3.08471	-1.66815	H	-1.32464	-2.84663	0.18851
H	-1.55254	2.83377	-1.29167	H	-2.28863	-2.51767	-1.25577
H	-0.37075	2.87004	0.00202	H	-0.76564	-3.40155	-1.38546
H	-3.57141	-1.23322	-1.10121	H	-3.9649	-1.13505	-0.87375
H	-2.97321	-1.49615	0.51733	H	-2.93256	-1.17303	0.55079
H	-6.37643	-1.85488	0.74329	H	-4.59407	1.58928	-0.79422
H	-5.93635	-0.04432	2.28293	H	-5.99192	-1.28223	0.50799
H	-4.47835	0.91592	1.97015	H	-6.30122	-0.09723	1.79314
H	-4.33196	-0.76658	2.52136	H	-5.02599	-1.31453	1.9991
H	-5.78243	-0.0525	-1.4777	H	-3.30897	0.58154	2.43454
H	-6.79871	0.36974	-0.08463	H	-4.64388	1.73869	2.25615
H	-5.37388	1.35263	-0.47008	H	-3.18328	1.91178	1.26842
atom		Con f. 4- 5s		atom		Con f. 4- 5t	
C	5.26126	-0.88528	0.06308	C	5.20901	-0.72735	0.39126
C	1.84345	3.20436	0.38561	C	1.57396	3.20652	0.08373
C	2.30239	2.21546	-0.3916	C	2.1635	2.18946	-0.5573
C	1.95404	0.76531	-0.08133	C	1.8649	0.75093	-0.15595
C	2.98803	0.01583	0.84318	C	2.84771	0.13391	0.90996
C	4.40056	0.10554	0.30427	C	4.29487	0.24098	0.47616
C	1.65548	-0.02305	-1.37327	C	1.72316	-0.15528	-1.39665
C	1.15385	-1.45379	-1.1363	C	1.25409	-1.58157	-1.07974
C	2.19558	-2.19234	-0.26381	C	2.23411	-2.19711	-0.05432
C	2.48976	-1.43376	1.03208	C	2.39049	-1.31565	1.18696
C	3.1222	2.52615	-1.62139	C	3.07634	2.45195	-1.73154
C	3.01232	0.68918	2.23167	C	2.73197	0.92391	2.23052
C	-0.25786	-1.6023	-0.59508	C	-0.19905	-1.74775	-0.66923
C	-1.06524	-0.53192	-0.39037	C	-1.06322	-0.70165	-0.65265
C	-0.70417	-3.02068	-0.36538	C	-0.61156	-3.15874	-0.351
C	-2.43234	-0.555	0.12504	C	-2.48316	-0.74736	-0.31116
C	-3.17174	0.77312	0.09459	C	-3.29475	0.48865	-0.66374
O	-2.97044	-1.56546	0.61085	O	-3.03429	-1.72923	0.21819
C	-4.70955	0.66893	0.10853	C	-4.54	0.72563	0.21518
O	-5.13448	-0.04663	1.28494	O	-5.41897	-0.41223	0.12275
C	-5.32096	2.06308	0.21833	C	-4.14233	0.95638	1.67898
C	-5.22428	-0.04593	-1.1479	C	-5.33131	1.91593	-0.31982
H	1.04162	0.78998	0.52107	H	0.90226	0.77129	0.36323
H	1.13126	-1.97006	-2.10801	H	1.34433	-2.17936	-1.99958
H	6.27364	-0.67196	-0.27089	H	6.23609	-0.49877	0.11775
H	5.01087	-1.93378	0.19203	H	4.98877	-1.7705	0.59571
H	2.08066	4.24611	0.18247	H	1.7693	4.24161	-0.18685
H	1.21074	3.00718	1.24709	H	0.87305	3.03841	0.89722
H	4.75649	1.12632	0.17217	H	4.62049	1.26126	0.27873
H	2.57265	-0.09848	-1.96738	H	2.69713	-0.2395	-1.89063
H	0.94031	0.53658	-1.9856	H	1.05241	0.31635	-2.12242
H	1.86394	-3.20729	-0.02829	H	1.91317	-3.19818	0.24619
H	3.10624	-2.29269	-0.86058	H	3.19633	-2.31768	-0.5591
H	3.21992	-1.98913	1.63158	H	3.08844	-1.78438	1.89002
H	1.57086	-1.3924	1.63114	H	1.42376	-1.26395	1.70422
H	4.02104	1.9067	-1.69147	H	4.01441	1.89301	-1.6677
H	2.54425	2.3351	-2.53379	H	2.59953	2.14641	-2.67078
H	3.4253	3.57688	-1.63392	H	3.31426	3.51644	-1.81081
H	2.00548	0.72032	2.66347	H	3.07414	1.95601	2.11815
H	3.38947	1.71371	2.17932	H	1.69253	0.94741	2.57705
H	3.65823	0.12434	2.91235	H	3.33961	0.44861	3.00795
H	-0.70154	0.45861	-0.63571	H	-0.70862	0.2778	-0.94786
H	-0.57713	-3.29946	0.68818	H	-0.57822	-3.33543	0.73136
H	-1.76118	-3.15261	-0.5977	H	-1.63546	-3.35924	-0.66793
H	-0.10989	-3.71554	-0.96448	H	0.06412	-3.87635	-0.82349

H	-2.84269	1.32816	0.98544	H	-2.6483	1.37122	-0.65043
H	-2.83904	1.35626	-0.76998	H	-3.61782	0.35633	-1.70664
H	-4.5975	-0.86194	1.27849	H	-4.83722	-1.18034	0.28198
H	-6.41259	1.99102	0.2637	H	-5.0382	1.11919	2.28643
H	-5.0516	2.67783	-0.64567	H	-3.49413	1.83357	1.77757
H	-4.97047	2.56565	1.12558	H	-3.60987	0.09012	2.08257
H	-6.31624	-0.11396	-1.11533	H	-4.73753	2.83371	-0.27374
H	-4.82112	-1.06046	-1.21674	H	-6.23824	2.06143	0.27625
H	-4.93933	0.49819	-2.05452	H	-5.62672	1.74449	-1.35995

atom	Con f. 4- 6a			atom	Con f. 4- 6b		
C	5.29759	-1.57734	1.06666	C	5.19482	-1.79588	1.04199
C	4.1367	2.95256	-0.5854	C	4.3836	2.79806	-0.70338
C	3.67574	1.95678	0.18128	C	3.87651	1.85588	0.1011
C	2.88835	0.81421	-0.4412	C	2.97549	0.77152	-0.46781
C	3.69746	-0.52324	-0.61674	C	3.6592	-0.63513	-0.632
C	4.0901	-1.13066	0.71423	C	4.0233	-1.25384	0.70195
C	1.54491	0.59222	0.28125	C	1.63807	0.6836	0.29273
C	0.65984	-0.42379	-0.46876	C	0.64734	-0.26671	-0.41129
C	1.41371	-1.7544	-0.66645	C	1.2762	-1.66122	-0.60715
C	2.76131	-1.52393	-1.35405	C	2.62083	-1.55992	-1.33088
C	3.89802	1.96437	1.67286	C	4.16192	1.86905	1.58156
C	4.91767	-0.27308	-1.50965	C	4.87758	-0.51053	-1.55304
C	-0.6644	-0.62997	0.22797	C	-0.66589	-0.33831	0.33151
C	-1.80644	-0.314	-0.43367	C	-1.79172	0.09933	-0.287
C	-0.63695	-1.18001	1.62703	C	-0.64672	-0.89337	1.729
C	-3.16787	-0.45243	0.07319	C	-3.13691	0.11726	0.27888
C	-4.28838	-0.0961	-0.88995	C	-4.22673	0.71697	-0.59466
O	-3.43253	-0.86542	1.21733	O	-3.40169	-0.29706	1.42225
C	-5.58282	0.41012	-0.22107	C	-5.65495	0.22051	-0.29221
O	-6.09423	-0.60135	0.66859	O	-5.98581	0.511	1.07953
C	-5.3269	1.70558	0.56056	C	-6.66131	0.99126	-1.14221
C	-6.66113	0.62725	-1.27924	C	-5.77914	-1.28759	-0.54649
H	2.64858	1.12458	-1.46634	H	2.73406	1.07689	-1.49395
H	0.45744	-0.01052	-1.46453	H	0.4501	0.14718	-1.40783
H	5.46114	-2.02874	2.04199	H	5.33627	-2.24548	2.02164
H	6.16373	-1.51345	0.41482	H	6.05242	-1.81466	0.37617
H	4.69714	3.78402	-0.16411	H	5.02603	3.58845	-0.32194
H	3.96937	2.97031	-1.65948	H	4.17409	2.81088	-1.77016
H	3.28372	-1.23783	1.43656	H	3.22367	-1.28468	1.4389
H	1.01641	1.54985	0.35002	H	1.19611	1.68486	0.34909
H	1.71249	0.25309	1.30753	H	1.80084	0.35718	1.3238
H	1.56269	-2.24863	0.29925	H	1.40723	-2.15397	0.36165
H	0.80223	-2.43074	-1.27433	H	0.59197	-2.2891	-1.18891
H	3.28774	-2.47839	-1.46866	H	3.05789	-2.55888	-1.4424
H	2.58335	-1.13428	-2.3651	H	2.45285	-1.17035	-2.34372
H	2.94705	1.99518	2.21772	H	3.23987	1.97577	2.16498
H	4.41969	1.06157	2.00426	H	4.63216	0.93599	1.90622
H	4.48777	2.83419	1.97579	H	4.8244	2.69862	1.84464
H	5.42217	-1.21279	-1.75613	H	5.29072	-1.49581	-1.79175
H	4.60689	0.19387	-2.44994	H	4.59058	-0.03303	-2.49565
H	5.64395	0.38894	-1.03123	H	5.67019	0.09134	-1.10133
H	-1.72132	0.07743	-1.44355	H	-1.70542	0.48339	-1.2997
H	-1.09782	-0.4778	2.32901	H	-1.0363	-0.16148	2.44344
H	-1.22106	-2.1031	1.6939	H	-1.29803	-1.76947	1.80768
H	0.38118	-1.38562	1.96004	H	0.35993	-1.17983	2.03686
H	-3.92995	0.63484	-1.62066	H	-4.18708	1.8042	-0.43345
H	-4.51671	-1.01371	-1.45134	H	-3.98347	0.5527	-1.64888
H	-5.32404	-0.86126	1.20985	H	-5.23044	0.16113	1.58975
H	-4.9564	2.49726	-0.0991	H	-6.5831	2.06629	-0.95048
H	-4.58961	1.5475	1.35329	H	-7.6799	0.67126	-0.89914
H	-6.25668	2.04968	1.02444	H	-6.48973	0.81481	-2.20826
H	-6.8523	-0.29981	-1.8292	H	-5.56008	-1.53078	-1.5915
H	-7.5946	0.9441	-0.80262	H	-5.089	-1.85137	0.08792
H	-6.35909	1.39961	-1.99274	H	-6.79774	-1.61916	-0.32154

atom	Con f. 4- 6c			atom	Con f. 4- 6d		
C	5.19454	-1.79595	1.04255	C	5.29712	-1.57982	1.06635
C	4.38377	2.79763	-0.70452	C	4.13859	2.95177	-0.58688
C	3.87679	1.85569	0.10033	C	3.67774	1.95623	0.1802
C	2.97558	0.77124	-0.46812	C	2.88948	0.81388	-0.44162
C	3.65904	-0.6356	-0.6318	C	3.6977	-0.52414	-0.61674
C	4.02308	-1.25387	0.70235	C	4.09016	-1.13133	0.71441
C	1.63818	0.68381	0.29252	C	1.54607	0.59308	0.28125
C	0.64726	-0.26658	-0.41113	C	0.66016	-0.42269	-0.46823

C	1.27587	-1.66127	-0.60642	C	1.41312	-1.75384	-0.66558
C	2.62048	-1.56047	-1.33027	C	2.76078	-1.52454	-1.35351
C	4.16258	1.86922	1.58071	C	3.90091	1.96392	1.67164
C	4.87741	-0.51156	-1.55294	C	4.91798	-0.27512	-1.50988
C	-0.66596	-0.33756	0.33172	C	-0.66405	-0.62749	0.22892
C	-1.79176	0.09981	-0.28704	C	-1.80608	-0.31134	-0.43268
C	-0.64689	-0.89175	1.72956	C	-0.63664	-1.17625	1.62851
C	-3.13689	0.11829	0.27892	C	-3.16749	-0.44849	0.07471
C	-4.22691	0.71696	-0.59509	C	-4.28775	-0.09359	-0.88927
O	-3.4015	-0.29475	1.4228	O	-3.43204	-0.85939	1.21959
C	-5.65488	0.2199	-0.29239	C	-5.58464	0.40796	-0.22184
O	-5.98605	0.51135	1.07907	O	-6.09402	-0.6058	0.66636
C	-6.66162	0.98933	-1.14316	C	-5.33387	1.70374	0.5609
C	-5.7782	-1.28848	-0.54537	C	-6.6623	0.62241	-1.28124
H	2.73412	1.07625	-1.49436	H	2.64962	1.12397	-1.46681
H	0.45009	0.14694	-1.40784	H	0.45788	-0.00963	-1.46411
H	5.33597	-2.24519	2.02237	H	5.46057	-2.03081	2.04188
H	6.05211	-1.81509	0.3767	H	6.16292	-1.51783	0.41388
H	5.02631	3.58808	-0.32341	H	4.69954	3.7831	-0.16602
H	4.17403	2.81018	-1.77125	H	3.97064	2.96944	-1.66087
H	3.22345	-1.28432	1.43933	H	3.28407	-1.23656	1.43734
H	1.1964	1.68516	0.34856	H	1.01818	1.55107	0.34971
H	1.80093	0.35768	1.32368	H	1.7136	0.25423	1.30762
H	1.40688	-2.15362	0.36259	H	1.56198	-2.24787	0.30024
H	0.5915	-2.2893	-1.18788	H	0.80109	-2.43002	-1.27309
H	3.05736	-2.55955	-1.44143	H	3.28656	-2.4794	-1.46782
H	2.45251	-1.17127	-2.34324	H	2.58289	-1.13518	-2.36468
H	3.24071	1.97582	2.16441	H	4.49083	2.83375	1.97419
H	4.63314	0.93632	1.90538	H	2.95028	1.99469	2.21709
H	4.82497	2.69899	1.84343	H	4.42281	1.06113	2.00274
H	5.29052	-1.49699	-1.79109	H	5.42207	-1.2152	-1.7558
H	4.59042	-0.03459	-2.49582	H	4.60738	0.19137	-2.45046
H	5.67004	0.09054	-1.10158	H	5.64458	0.38687	-1.03189
H	-1.70546	0.48314	-1.30002	H	-1.72099	0.079	-1.44299
H	-1.03552	-0.159	2.44365	H	0.38135	-1.38289	1.96128
H	-1.29911	-1.7671	1.80905	H	-1.09624	-0.47272	2.33002
H	0.35957	-1.17901	2.0373	H	-1.22207	-2.0984	1.69664
H	-4.18794	1.8043	-0.43442	H	-3.93	0.6395	-1.61823
H	-3.98352	0.55223	-1.64919	H	-4.51232	-1.01083	-1.45285
H	-5.23049	0.16241	1.58963	H	-5.32433	-0.86186	1.21011
H	-6.48993	0.81196	-2.20903	H	-4.96527	2.49702	-0.0979
H	-6.58398	2.06458	-0.95244	H	-4.59686	1.5476	1.35427
H	-7.68004	0.66903	-0.89979	H	-6.26525	2.04453	1.024
H	-5.08783	-1.85136	0.08959	H	-7.59722	0.93655	-0.80568
H	-6.79664	-1.62041	-0.32024	H	-6.36155	1.39578	-1.99422
H	-5.5589	-1.5325	-1.59014	H	-6.85022	-0.30503	-1.83169
atom	Con f. 4- 6e			atom	Con f. 4- 6f		
C	-4.0587	2.62149	1.33254	C	4.44919	0.38549	2.55095
C	-5.20172	-1.98077	0.21732	C	4.07165	2.19744	-1.95171
C	-4.17447	-1.26705	0.69407	C	3.29792	1.83098	-0.92254
C	-3.19509	-0.60453	-0.26234	C	2.84579	0.38614	-0.77983
C	-3.42707	0.93664	-0.47542	C	3.65754	-0.45326	0.27599
C	-3.22487	1.72357	0.80268	C	3.48224	0.07659	1.68392
C	-1.73451	-0.93458	0.10131	C	1.32356	0.28629	-0.56123
C	-0.75171	-0.4263	-0.97954	C	0.84233	-1.16427	-0.64747
C	-0.92671	1.08364	-1.16278	C	1.5869	-2.02028	0.40412
C	-2.37755	1.41797	-1.51939	C	3.10254	-1.90642	0.22725
C	-3.94816	-1.13669	2.17953	C	2.83018	2.84508	0.09103
C	-4.82124	1.16904	-1.0684	C	5.13448	-0.5	-0.13044
C	0.64538	-0.91064	-0.66507	C	-0.6485	-1.38115	-0.52629
C	1.61107	-0.05756	-0.24035	C	-1.47134	-0.40696	-0.06347
C	0.86461	-2.38849	-0.84485	C	-1.12803	-2.7464	-0.93877
C	2.97906	-0.42335	0.11289	C	-2.91528	-0.51972	0.11972
C	3.87921	0.70355	0.59269	C	-3.62674	0.72171	0.63389
O	3.41048	-1.58984	0.06267	O	-3.55422	-1.56613	-0.09381
C	5.3825	0.49588	0.3139	C	-5.11289	0.83231	0.2365
O	5.82976	-0.71259	0.95806	O	-5.83145	-0.31467	0.73007
C	5.655	0.41065	-1.19374	C	-5.74034	2.0476	0.91385
C	6.18611	1.63171	0.94103	C	-5.26833	0.92534	-1.28716
H	-3.3768	-1.04682	-1.2502	H	3.06059	-0.10351	-1.73851
H	-1.03891	-0.91557	-1.92294	H	1.12927	-1.55714	-1.63512
H	-3.79719	3.15333	2.24395	H	4.20778	0.73037	3.55327
H	-5.02298	2.86292	0.89532	H	5.50531	0.31435	2.30868
H	-5.91365	-2.4685	0.87926	H	4.40313	3.22548	-2.07877
H	-5.36602	-2.10134	-0.85057	H	4.40014	1.48222	-2.7017

H	-2.28152	1.55133	1.31705	H	2.45356	0.18196	2.02284
H	-1.64085	-2.01898	0.21848	H	0.81667	0.89239	-1.32071
H	-1.45591	-0.48976	1.06221	H	1.05295	0.71127	0.41004
H	-0.64007	1.60954	-0.24558	H	1.28501	-1.68788	1.40418
H	-0.26115	1.44612	-1.95443	H	1.29166	-3.07095	0.31902
H	-2.48927	2.49977	-1.65447	H	3.61134	-2.5033	0.99303
H	-2.62282	0.95023	-2.48216	H	3.37489	-2.33671	-0.74537
H	-3.9295	-0.08873	2.49347	H	3.24074	3.8349	-0.12794
H	-4.73451	-1.64968	2.74059	H	1.73683	2.92712	0.09619
H	-2.98564	-1.57083	2.47474	H	3.12861	2.56562	1.10581
H	-4.95867	2.21934	-1.345	H	5.69628	-1.18213	0.51583
H	-4.95033	0.56677	-1.97339	H	5.2309	-0.86123	-1.15932
H	-5.61384	0.89461	-0.36786	H	5.60469	0.48512	-0.07703
H	1.37888	0.99684	-0.14569	H	-1.05184	0.55781	0.19721
H	1.15419	-2.86659	0.09625	H	-0.31324	-3.33276	-1.3698
H	-0.03689	-2.87482	-1.22436	H	-1.53999	-3.29626	-0.08587
H	1.6854	-2.57548	-1.54415	H	-1.9368	-2.67151	-1.67121
H	3.54297	1.65303	0.16612	H	-3.55452	0.6871	1.73076
H	3.72985	0.77851	1.67959	H	-3.08249	1.61606	0.31601
H	5.17516	-1.38642	0.69065	H	-5.2981	-1.07859	0.43728
H	5.11583	-0.42837	-1.64352	H	-5.25756	2.97249	0.58456
H	6.72494	0.26204	-1.3702	H	-5.64435	1.97309	2.00184
H	5.34491	1.32941	-1.70261	H	-6.80513	2.10688	0.66548
H	6.00043	1.68398	2.01872	H	-4.85896	0.03805	-1.77903
H	7.25675	1.46444	0.78376	H	-6.32842	1.00132	-1.54894
H	5.91844	2.59404	0.49471	H	-4.75078	1.80568	-1.68253
atom	Con f. 4- 6g			atom	Con f. 4- 6h		
C	-3.81434	2.82258	1.06164	C	4.07068	0.82114	2.67752
C	-5.08122	-1.93712	0.75952	C	4.32754	1.75324	-2.12491
C	-4.03414	-1.1457	1.02282	C	3.4282	1.59965	-1.14531
C	-3.15825	-0.63649	-0.11095	C	2.91446	0.21376	-0.78791
C	-3.41273	0.86065	-0.5187	C	3.55919	-0.41133	0.5041
C	-3.06073	1.82349	0.59663	C	3.21783	0.37693	1.75149
C	-1.66734	-0.93077	0.14516	C	1.3744	0.16997	-0.75065
C	-0.80097	-0.5976	-1.09282	C	0.85659	-1.26466	-0.61818
C	-1.00685	0.86687	-1.4897	C	1.44419	-1.91376	0.65769
C	-2.48907	1.16121	-1.7347	C	2.97274	-1.84434	0.65427
C	-3.68576	-0.77248	2.4417	C	2.88396	2.79393	-0.40252
C	-4.86616	1.02982	-0.97501	C	5.07444	-0.54146	0.31291
C	0.62577	-1.0327	-0.84512	C	-0.64538	-1.43444	-0.63694
C	1.62409	-0.12712	-0.68877	C	-1.4803	-0.37443	-0.49236
C	0.83572	-2.52044	-0.76561	C	-1.12395	-2.85142	-0.80068
C	3.02632	-0.43349	-0.42153	C	-2.93843	-0.42921	-0.46536
C	3.98531	0.74601	-0.41738	C	-3.66996	0.90251	-0.41945
O	3.45247	-1.58757	-0.23255	O	-3.58528	-1.49239	-0.5003
C	5.23944	0.56266	0.4621	C	-5.01367	0.87094	0.33947
O	5.97546	-0.59302	0.01647	O	-5.9016	-0.0713	-0.29225
C	6.17495	1.7552	0.28395	C	-4.80517	0.4804	1.80837
C	4.85492	0.39465	1.93804	C	-5.69705	2.23176	0.23756
H	-3.4389	-1.21267	-1.00193	H	3.23109	-0.45331	-1.6
H	-1.17315	-1.22627	-1.91635	H	1.23957	-1.84423	-1.47237
H	-3.44637	3.47703	1.84789	H	3.71153	1.35211	3.55555
H	-4.81395	3.03094	0.69222	H	5.14528	0.68266	2.60577
H	-5.7204	-2.31966	1.55199	H	4.70654	2.73398	-2.40306
H	-5.33481	-2.22906	-0.25659	H	4.71514	0.90383	-2.68198
H	-2.07243	1.69654	1.03336	H	2.15859	0.56511	1.914
H	-1.56225	-1.98859	0.40618	H	0.9862	0.62229	-1.67032
H	-1.29008	-0.35872	0.99921	H	0.9994	0.77727	0.07884
H	-0.62579	1.52547	-0.70168	H	1.03381	-1.39866	1.5341
H	-0.43282	1.09451	-2.39505	H	1.1312	-2.95985	0.73215
H	-2.61814	2.21101	-2.0215	H	3.36571	-2.29089	1.57498
H	-2.83865	0.5547	-2.58081	H	3.34991	-2.44991	-0.18033
H	-4.40096	-1.20554	3.14679	H	3.34508	3.71954	-0.75863
H	-2.68543	-1.12929	2.7137	H	1.79929	2.88443	-0.53469
H	-3.67973	0.31281	2.57993	H	3.06324	2.71234	0.67378
H	-5.03232	2.02936	-1.38952	H	5.5267	-1.0962	1.14136
H	-5.10309	0.30194	-1.75769	H	5.29167	-1.08762	-0.61074
H	-5.57172	0.87966	-0.15388	H	5.5642	0.43337	0.2477
H	1.39636	0.92887	-0.77468	H	-1.05823	0.61865	-0.39439
H	-0.06824	-3.05872	-1.06002	H	-1.88372	-2.92159	-1.5836
H	1.66344	-2.83436	-1.40711	H	-0.29397	-3.5179	-1.04653
H	1.10594	-2.82651	0.25097	H	-1.59919	-3.21436	0.11739
H	4.30279	0.88869	-1.46059	H	-3.0175	1.6734	-0.00006
H	3.45019	1.65572	-0.12919	H	-3.86419	1.18719	-1.46349
H	5.3022	-1.29797	-0.04798	H	-5.36072	-0.87711	-0.40444

H	5.69499	2.68208	0.61182	H	-4.36172	-0.5164	1.8906
H	6.46174	1.86469	-0.7669	H	-5.76715	0.46975	2.3303
H	7.08468	1.60966	0.87571	H	-4.14498	1.19189	2.31553
H	4.21557	-0.48159	2.08038	H	-5.8539	2.50582	-0.81072
H	5.75633	0.26041	2.54427	H	-6.67218	2.1988	0.73468
H	4.31776	1.27433	2.30802	H	-5.09372	3.01005	0.71429
atom	Con f. 4- 6i			atom	Con f. 4- 6j		
C	-3.81404	2.82267	1.06169	C	5.29426	-1.46024	1.14966
C	-5.08141	-1.93705	0.75918	C	3.81348	1.86531	1.74227
C	-4.03433	-1.14567	1.02262	C	3.5737	1.91473	0.42702
C	-3.15833	-0.63646	-0.11106	C	2.83398	0.81355	-0.32014
C	-3.41265	0.86073	-0.51869	C	3.69922	-0.47399	-0.57885
C	-3.06062	1.82341	0.59676	C	4.06781	-1.15739	0.72043
C	-1.66747	-0.93086	0.14513	C	1.47956	0.48199	0.33315
C	-0.80097	-0.59763	-1.09275	C	0.66139	-0.50011	-0.53124
C	-1.00673	0.86689	-1.48949	C	1.47093	-1.78174	-0.8116
C	-2.48891	1.16132	-1.7346	C	2.83279	-1.44682	-1.42681
C	-3.68611	-0.77253	2.44155	C	4.00348	3.09546	-0.40416
C	-4.86605	1.03008	-0.97506	C	4.94231	-0.10258	-1.39478
C	0.62574	-1.0328	-0.84498	C	-0.67914	-0.79817	0.09757
C	1.62405	-0.12725	-0.68845	C	-1.80361	-0.37727	-0.53535
C	0.8356	-2.52055	-0.76559	C	-0.68495	-1.53364	1.40885
C	3.0263	-0.43363	-0.42127	C	-3.17618	-0.55539	-0.07435
C	3.98534	0.74582	-0.4175	C	-4.26523	0.08885	-0.91688
O	3.4524	-1.58769	-0.23204	O	-3.47739	-1.21594	0.93699
C	5.23951	0.56265	0.46196	C	-5.5146	0.52857	-0.12474
O	5.97541	-0.59322	0.01669	O	-6.10534	-0.6173	0.51819
C	6.17511	1.75504	0.28334	C	-5.15386	1.58459	0.9287
C	4.85504	0.3952	1.93799	C	-6.57096	1.0695	-1.08427
H	-3.43897	-1.21256	-1.00209	H	2.60847	1.20266	-1.32223
H	-1.17307	-1.22619	-1.91639	H	0.47921	-0.00857	-1.49492
H	-3.44603	3.47699	1.84804	H	5.44142	-1.97397	2.09648
H	-4.81353	3.03136	0.6921	H	6.19309	-1.21566	0.59147
H	-5.72065	-2.31964	1.55158	H	4.34198	2.67002	2.24797
H	-5.33493	-2.22892	-0.25697	H	3.49772	1.0312	2.36016
H	-2.07241	1.69622	1.03364	H	3.2309	-1.44211	1.35459
H	-1.56249	-1.98872	0.40604	H	0.9112	1.40972	0.46077
H	-1.29022	-0.35893	0.99926	H	1.63124	0.06857	1.33438
H	-0.62569	1.52537	-0.70136	H	1.60429	-2.35191	0.11353
H	-0.43261	1.09461	-2.39477	H	0.90754	-2.42497	-1.49707
H	-2.61793	2.21113	-2.02134	H	3.39912	-2.36965	-1.59827
H	-2.83846	0.55488	-2.58078	H	2.67468	-0.98407	-2.40994
H	-3.68035	0.31276	2.57986	H	4.54289	3.8391	0.18996
H	-2.68571	-1.1291	2.7136	H	4.64769	2.78299	-1.23436
H	-4.40126	-1.20581	3.14655	H	3.13107	3.58466	-0.85764
H	-5.03208	2.02968	-1.38947	H	5.51064	-0.99681	-1.67092
H	-5.103	0.30231	-1.75783	H	4.65	0.4055	-2.31914
H	-5.57165	0.8799	-0.15397	H	5.60943	0.56134	-0.8381
H	1.39632	0.92876	-0.77431	H	-1.69184	0.16456	-1.47057
H	1.6634	-2.83444	-1.40701	H	0.32278	-1.64826	1.81105
H	-0.06834	-3.05876	-1.06019	H	-1.30643	-1.01913	2.14653
H	1.10566	-2.82676	0.25099	H	-1.12051	-2.53183	1.28734
H	4.30277	0.88818	-1.46076	H	-3.85323	0.936	-1.47271
H	3.45031	1.65565	-0.12954	H	-4.5679	-0.66037	-1.66253
H	5.30207	-1.2981	-0.04762	H	-5.35588	-1.05857	0.96297
H	6.46188	1.86411	-0.76757	H	-4.43014	1.19148	1.64888
H	7.08485	1.60966	0.87513	H	-6.05217	1.8834	1.47813
H	5.69523	2.68209	0.61086	H	-4.72262	2.47629	0.46164
H	4.21562	-0.48094	2.08065	H	-6.8354	0.3134	-1.83048
H	5.75646	0.26108	2.54422	H	-7.47635	1.33904	-0.53057
H	4.31797	1.27505	2.30769	H	-6.20731	1.96042	-1.60472
atom	Con f. 4- 6k			atom	Con f. 4- 6l		
C	5.17054	-1.85305	0.95482	C	4.07191	0.82273	2.6768
C	4.22993	1.63636	1.67007	C	4.32979	1.75119	-2.1248
C	3.91737	1.74786	0.37398	C	3.42961	1.59852	-1.1458
C	2.98212	0.7783	-0.33567	C	2.91526	0.21301	-0.7878
C	3.63658	-0.61333	-0.66201	C	3.55956	-0.41157	0.5046
C	3.97739	-1.37025	0.60365	C	3.21873	0.37791	1.7514
C	1.63931	0.63035	0.40309	C	1.37517	0.16987	-0.7507
C	0.635	-0.21844	-0.40503	C	0.85678	-1.26446	-0.6174
C	1.23845	-1.59148	-0.76609	C	1.44367	-1.91282	0.6591
C	2.59013	-1.4328	-1.46739	C	2.97227	-1.84415	0.6559
C	4.45734	2.87412	-0.46839	C	2.88501	2.7935	-0.4044
C	4.87036	-0.40649	-1.54735	C	5.07473	-0.54284	0.3136
C	-0.68009	-0.35742	0.32464	C	-0.64523	-1.43369	-0.6367

C	-1.8003	0.15928	-0.24142	C	-1.47975	-0.37347	-0.4914
C	-0.66965	-1.0667	1.65073	C	-1.12438	-2.85036	-0.8014
C	-3.14684	0.12252	0.31916	C	-2.93791	-0.42765	-0.4645
C	-4.23236	0.82618	-0.47846	C	-3.66825	0.90452	-0.4133
O	-3.41932	-0.42247	1.40478	O	-3.58534	-1.49029	-0.5038
C	-5.65278	0.25632	-0.2841	C	-5.01521	0.87026	0.3394
O	-6.01938	0.33899	1.10679	O	-5.90131	-0.06784	-0.3009
C	-6.66279	1.11565	-1.03951	C	-4.81336	0.47204	1.8073
C	-5.72818	-1.20244	-0.75407	C	-5.69688	2.23223	0.2416
H	2.75316	1.21414	-1.31746	H	3.23175	-0.45463	-1.5994
H	0.44514	0.31285	-1.34585	H	1.23989	-1.84479	-1.4711
H	5.29719	-2.40224	1.88453	H	3.71311	1.35469	3.5544
H	6.06119	-1.72673	0.34651	H	5.14645	0.68382	2.605
H	4.89894	2.34631	2.15062	H	4.70926	2.73164	-2.4033
H	3.83791	0.84138	2.29589	H	4.7176	0.90131	-2.681
H	3.14564	-1.54603	1.28238	H	2.15959	0.56664	1.9139
H	1.21496	1.62658	0.56925	H	0.98726	0.62177	-1.6707
H	1.79806	0.19137	1.3921	H	1.00036	0.77783	0.0783
H	1.35105	-2.20231	0.13558	H	1.03329	-1.3968	1.5349
H	0.54505	-2.129	-1.42273	H	1.13013	-2.9587	0.7343
H	3.00898	-2.42047	-1.69296	H	3.36482	-2.29009	1.5771
H	2.43576	-0.92608	-2.4292	H	3.34931	-2.45064	-0.1779
H	4.99194	2.49255	-1.34616	H	3.06254	2.71229	0.6721
H	3.63682	3.49505	-0.85222	H	3.3473	3.71863	-0.7602
H	5.13769	3.51757	0.09727	H	1.80061	2.88467	-0.5383
H	5.28817	-1.36694	-1.86667	H	5.52658	-1.0972	1.1425
H	4.60105	0.15586	-2.44708	H	5.29163	-1.08992	-0.6095
H	5.65673	0.14505	-1.02451	H	5.56514	0.4316	0.2476
H	-1.70697	0.65647	-1.20288	H	-1.05726	0.61931	-0.3922
H	-1.31977	-1.94702	1.62605	H	-0.29406	-3.51773	-1.0436
H	0.33523	-1.38524	1.93174	H	-1.6039	-3.21236	0.1147
H	-1.06523	-0.4204	2.44033	H	-1.8809	-2.92019	-1.5875
H	-4.22523	1.87628	-0.15222	H	-3.01655	1.67188	0.0137
H	-3.96795	0.8288	-1.53999	H	-3.85716	1.19619	-1.4564
H	-5.25843	-0.0483	1.58111	H	-5.36118	-0.87406	-0.4136
H	-6.62105	2.15281	-0.69148	H	-4.37069	-0.52536	1.8862
H	-7.6764	0.73667	-0.87238	H	-5.77768	0.45908	2.3249
H	-6.46258	1.10136	-2.11503	H	-4.15517	1.18059	2.3211
H	-5.47523	-1.28787	-1.81617	H	-5.84887	2.51188	-0.8058
H	-5.03898	-1.83432	-0.18611	H	-6.67422	2.19762	0.7343
H	-6.7423	-1.58764	-0.60866	H	-5.09492	3.00749	0.725
atom	Con f. 4- 6m			atom	Con f. 4- 6n		
C	-4.05865	2.62167	1.33221	C	-4.44887	-0.38563	2.55123
C	-5.20173	-1.98065	0.21783	C	-4.07183	-2.19704	-1.95207
C	-4.1744	-1.26692	0.6944	C	-3.29811	-1.83081	-0.92281
C	-3.19507	-0.60458	-0.26219	C	-2.84592	-0.386	-0.77985
C	-3.42706	0.93656	-0.47551	C	-3.6575	0.45322	0.27621
C	-3.22482	1.72369	0.80245	C	-3.48202	-0.07683	1.68404
C	-1.73446	-0.93457	0.10143	C	-1.32365	-0.28626	-0.56144
C	-0.75174	-0.42648	-0.97959	C	-0.84239	1.16431	-0.64748
C	-0.92674	1.08345	-1.16304	C	-1.58682	2.02015	0.40437
C	-2.37759	1.41774	-1.51961	C	-3.10248	1.90638	0.22764
C	-3.94794	-1.1364	2.17981	C	-2.83049	-2.84509	0.09062
C	-4.82126	1.16887	-1.06845	C	-5.13449	0.50006	-0.13002
C	0.64537	-0.91078	-0.66517	C	0.64845	1.38112	-0.52641
C	1.61107	-0.05765	-0.24055	C	1.47128	0.40689	-0.06369
C	0.86463	-2.38863	-0.84489	C	1.12802	2.74638	-0.93886
C	2.97907	-0.42341	0.1127	C	2.91525	0.51959	0.11934
C	3.8791	0.70343	0.59285	C	3.62666	-0.72188	0.63349
O	3.41059	-1.58986	0.06219	O	3.55419	1.56596	-0.09434
C	5.38241	0.49596	0.31404	C	5.11296	-0.83228	0.23665
O	5.82976	-0.71266	0.95785	O	5.8312	0.31474	0.7306
C	5.65496	0.4112	-1.19362	C	5.74031	-2.04754	0.91414
C	6.18591	1.63169	0.94151	C	5.26899	-0.92517	-1.28696
H	-3.37684	-1.04703	-1.24996	H	-3.06082	0.10382	-1.73842
H	-1.03903	-0.91588	-1.92288	H	-1.12943	1.55735	-1.63503
H	-3.79712	3.15364	2.24354	H	-4.20734	-0.73062	3.55348
H	-5.02295	2.86301	0.895	H	-5.50502	-0.31427	2.30916
H	-5.91363	-2.46824	0.87989	H	-4.40334	-3.22505	-2.07932
H	-5.36612	-2.10136	-0.85003	H	-4.40028	-1.48168	-2.70193
H	-2.28144	1.55155	1.31681	H	-2.4533	-0.18237	2.02279
H	-1.64079	-2.01896	0.21876	H	-0.81688	-0.89223	-1.3211
H	-1.45579	-0.4896	1.06223	H	-1.05292	-0.71143	0.40971
H	-0.64002	1.60948	-0.24594	H	-1.28485	1.68752	1.40432
H	-0.26123	1.44579	-1.95479	H	-1.29153	3.07082	0.31946

H	-2.4893	2.49952	-1.65484	H	-3.61117	2.50314	0.99357
H	-2.62292	0.94987	-2.4823	H	-3.37493	2.33683	-0.74488
H	-4.73432	-1.64919	2.74101	H	-3.24092	-3.83489	-0.12865
H	-2.98547	-1.57067	2.475	H	-1.73714	-2.92703	0.09601
H	-3.92908	-0.0884	2.49362	H	-3.12915	-2.56588	1.1054
H	-4.9587	2.21913	-1.34519	H	-5.6962	1.18207	0.51645
H	-4.9504	0.56647	-1.97336	H	-5.23104	0.86149	-1.15882
H	-5.61383	0.89452	-0.36784	H	-5.6047	-0.48507	-0.07674
H	1.37885	0.99674	-0.14589	H	1.05175	-0.55787	0.19701
H	-0.03701	-2.87507	-1.22396	H	0.31312	3.33293	-1.36944
H	1.68512	-2.5756	-1.54453	H	1.54052	3.29603	-0.08612
H	1.15467	-2.86663	0.09612	H	1.9364	2.67144	-1.67175
H	3.54278	1.653	0.16655	H	3.55399	-0.68755	1.73034
H	3.7297	0.77806	1.67977	H	3.08259	-1.6162	0.31519
H	5.17523	-1.38648	0.69023	H	5.29797	1.07861	0.43746
H	5.11587	-0.42772	-1.64367	H	5.25773	-2.97246	0.58463
H	6.72492	0.26272	-1.3701	H	5.64393	-1.9731	2.0021
H	5.34483	1.33009	-1.70223	H	6.80519	-2.1067	0.66612
H	6.00023	1.6836	2.01922	H	4.85969	-0.03792	-1.77893
H	7.25657	1.46458	0.78418	H	6.3292	-1.00099	-1.54834
H	5.91813	2.59412	0.49549	H	4.75173	-1.80557	-1.68258
atom		Con f. 4- 6o		atom		Con f. 4- 6p	
C	5.17013	-1.85331	0.95518	C	5.29224	-1.65675	0.97129
C	4.22985	1.63644	1.67002	C	4.06137	1.73899	1.71166
C	3.91747	1.74784	0.37387	C	3.75331	1.83708	0.41341
C	2.98222	0.77829	-0.3358	C	2.90663	0.80229	-0.31524
C	3.63658	-0.61343	-0.66192	C	3.6731	-0.53044	-0.64559
C	3.97712	-1.37025	0.60387	C	4.06672	-1.26605	0.61711
C	1.63935	0.63051	0.40287	C	1.56999	0.54361	0.40454
C	0.63501	-0.21829	-0.40521	C	0.64738	-0.36639	-0.4313
C	1.23838	-1.59138	-0.7662	C	1.35788	-1.6912	-0.77802
C	2.59015	-1.43285	-1.46737	C	2.70262	-1.42918	-1.46181
C	4.45764	2.87398	-0.46854	C	4.2074	3.0119	-0.41328
C	4.87052	-0.40682	-1.5471	C	4.89142	-0.21926	-1.52163
C	-0.68006	-0.35716	0.32452	C	-0.67675	-0.60439	0.25483
C	-1.80029	0.15949	-0.24154	C	-1.81727	-0.2535	-0.39193
C	-0.6696	-1.06637	1.65066	C	-0.64978	-1.2217	1.62501
C	-3.14682	0.12262	0.31906	C	-3.18011	-0.4097	0.10565
C	-4.23242	0.82602	-0.47866	C	-4.29889	-0.09852	-0.87499
O	-3.41921	-0.42226	1.40476	O	-3.44653	-0.80001	1.2574
C	-5.65281	0.25623	-0.28403	C	-5.60632	0.40646	-0.23072
O	-6.01927	0.33918	1.10688	O	-6.10923	-0.5872	0.68344
C	-6.66289	1.11541	-1.03952	C	-5.37584	1.72757	0.51486
C	-5.72826	-1.20264	-0.75371	C	-6.67886	0.57671	-1.30329
H	2.7534	1.21405	-1.31766	H	2.65488	1.22834	-1.2958
H	0.44516	0.31297	-1.34605	H	0.44747	0.15176	-1.37738
H	5.29659	-2.40238	1.88498	H	5.45678	-2.20197	1.8974
H	6.06084	-1.72732	0.34689	H	6.17364	-1.45514	0.36979
H	4.89886	2.34638	2.15058	H	4.6646	2.49718	2.20545
H	3.83769	0.84155	2.29585	H	3.73003	0.90854	2.32642
H	3.14529	-1.54569	1.28259	H	3.24802	-1.51413	1.28939
H	1.21505	1.62679	0.56889	H	1.07117	1.50322	0.57963
H	1.798	0.19162	1.39193	H	1.74926	0.10121	1.38852
H	1.35086	-2.20223	0.13548	H	1.50202	-2.28844	0.12847
H	0.54499	-2.12885	-1.42289	H	0.71754	-2.28187	-1.44275
H	3.00894	-2.42057	-1.69284	H	3.19962	-2.38128	-1.68146
H	2.43593	-0.92617	-2.42922	H	2.51993	-0.93645	-2.42592
H	4.99235	2.49229	-1.34619	H	3.34248	3.56221	-0.80718
H	3.63722	3.49492	-0.85255	H	4.82061	3.70763	0.16709
H	5.13795	3.51745	0.09716	H	4.78683	2.68422	-1.28443
H	5.28826	-1.36735	-1.86627	H	5.38648	-1.14085	-1.8449
H	4.60141	0.15549	-2.44691	H	4.58295	0.32608	-2.41909
H	5.65688	0.14468	-1.02419	H	5.62849	0.38921	-0.9903
H	-1.707	0.65666	-1.20301	H	-1.72898	0.1752	-1.38629
H	0.33525	-1.38507	1.93159	H	-1.36267	-2.04656	1.70174
H	-1.06499	-0.41995	2.44025	H	0.34626	-1.58375	1.88496
H	-1.31988	-1.94657	1.62611	H	-0.95179	-0.48689	2.38025
H	-4.22523	1.87625	-0.1528	H	-3.94651	0.61646	-1.62408
H	-3.96808	0.82829	-1.54021	H	-4.50877	-1.03551	-1.41097
H	-5.25833	-0.04815	1.58119	H	-5.33902	-0.82238	1.23588
H	-6.46274	1.10097	-2.11504	H	-5.01303	2.50532	-0.16536
H	-6.62118	2.15262	-0.69163	H	-4.64128	1.60356	1.31612
H	-7.67648	0.7364	-0.87228	H	-6.31384	2.07061	0.96268
H	-5.03902	-1.8344	-0.18567	H	-6.85272	-0.36906	-1.82655
H	-6.74238	-1.5878	-0.60815	H	-7.62053	0.89485	-0.84393

H	-5.47539	-1.28827	-1.81581	H	-6.38138	1.33123	-2.03749
atom	Con f. 4- 6q			atom	Con f. 4- 6r		
C	-4.03304	2.6263	1.35447	C	4.29136	0.46	2.63561
C	-4.1117	-0.98439	2.08919	C	3.02469	2.75747	0.07851
C	-4.18242	-1.17722	0.76719	C	3.34239	1.81898	-0.82038
C	-3.18348	-0.5726	-0.20965	C	2.85544	0.38023	-0.71737
C	-3.39555	0.96283	-0.47071	C	3.6192	-0.475	0.35962
C	-3.1782	1.77128	0.79016	C	3.37356	0.04979	1.75833
C	-1.72976	-0.90849	0.17183	C	1.32692	0.29671	-0.54736
C	-0.73633	-0.44237	-0.91992	C	0.83379	-1.14923	-0.66019
C	-0.89778	1.06102	-1.16333	C	1.54416	-2.03338	0.39299
C	-2.3455	1.40048	-1.53054	C	3.06614	-1.92471	0.2702
C	-5.25331	-2.04412	0.15802	C	4.18487	2.14278	-2.02667
C	-4.79354	1.19426	-1.05558	C	5.11389	-0.50793	0.02326
C	0.65606	-0.92405	-0.58203	C	0.66088	-1.3552	-0.57118
C	1.63074	-0.06088	-0.19994	C	1.48217	-0.38718	-0.0932
C	0.86112	-2.41105	-0.68834	C	1.14569	-2.70506	-1.02631
C	2.99626	-0.42189	0.16668	C	2.92857	-0.4953	0.07391
C	3.91662	0.72136	0.56221	C	3.63357	0.73669	0.61846
O	3.41158	-1.59514	0.19151	O	3.57228	-1.53105	-0.17369
C	5.41144	0.48033	0.26558	C	5.12698	0.85095	0.25292
O	5.86072	-0.69202	0.9721	O	5.83284	-0.30732	0.73884
C	5.6499	0.30027	-1.23952	C	5.74385	2.05113	0.96592
C	6.2399	1.64344	0.80428	C	5.31402	0.97325	-1.26522
H	-3.36096	-1.04637	-1.18436	H	3.09037	-0.10606	-1.67384
H	-1.0223	-0.96545	-1.84553	H	1.13802	-1.52943	-1.64758
H	-3.76087	3.1724	2.25435	H	4.00094	0.79961	3.62677
H	-5.02538	2.81939	0.95795	H	5.35436	0.47832	2.41435
H	-4.83738	-1.43392	2.76288	H	3.38262	3.77924	-0.02392
H	-3.33756	-0.37763	2.54729	H	2.40434	2.5464	0.94359
H	-2.20865	1.64527	1.26726	H	2.33322	0.0711	2.07599
H	-1.65055	-1.98972	0.3198	H	0.85036	0.91331	-1.31726
H	-1.45638	-0.44186	1.12397	H	1.03673	0.72291	0.41741
H	-0.60169	1.62017	-0.26927	H	1.21076	-1.72531	1.39064
H	-0.22993	1.38366	-1.97015	H	1.24642	-3.07992	0.27258
H	-2.44401	2.47863	-1.70145	H	3.54478	-2.53652	1.04386
H	-2.59724	0.90513	-2.47774	H	3.37113	-2.34173	-0.6986
H	-5.8322	-1.49294	-0.5925	H	5.07071	1.49902	-2.07843
H	-4.80481	-2.89922	-0.36524	H	3.61867	1.96265	-2.95036
H	-5.94537	-2.42933	0.91268	H	4.51585	3.18554	-2.02629
H	-4.93136	2.24282	-1.33933	H	5.65372	-1.17658	0.70188
H	-4.93295	0.58448	-1.9538	H	5.26377	-0.8749	-0.99698
H	-5.58035	0.93324	-0.34239	H	5.56849	0.48385	0.09686
H	1.41017	0.99938	-0.1598	H	1.0586	0.56686	0.19887
H	1.10052	-2.84935	0.2861	H	0.32951	-3.28664	-1.46127
H	-0.03158	-2.90183	-1.08271	H	1.57439	-3.2738	-0.19429
H	1.70869	-2.64139	-1.34036	H	1.9433	-2.6042	-1.76784
H	3.58192	1.64788	0.08691	H	3.53803	0.68794	1.71307
H	3.79071	0.86101	1.64562	H	3.09606	1.63594	0.30222
H	5.19198	-1.37258	0.76265	H	5.30984	-1.06385	0.41092
H	5.09373	-0.55954	-1.62483	H	5.62749	1.95535	2.05028
H	6.71436	0.13055	-1.4296	H	6.81316	2.11328	0.73852
H	5.33649	1.18904	-1.79722	H	5.2687	2.98331	0.6462
H	6.07752	1.76549	1.88007	H	4.8083	1.8636	-1.65341
H	7.30512	1.45366	0.63583	H	4.91141	0.09776	-1.78298
H	5.97324	2.57897	0.30365	H	6.37966	1.05007	-1.50319
atom	Con f. 4- 6s			atom	Con f. 4- 6t		
C	-3.77804	2.82934	1.06384	C	-3.98658	-0.91429	2.68735
C	-3.77741	-0.61325	2.38042	C	-3.05713	-2.7058	-0.36857
C	-4.00672	-1.01808	1.12622	C	-3.43483	-1.61477	-1.04453
C	-3.13545	-0.5972	-0.04888	C	-2.90158	-0.22387	-0.73046
C	-3.38199	0.87823	-0.53062	C	-3.52581	0.4188	0.56218
C	-3.00274	1.87749	0.54106	C	-3.15297	-0.36283	1.80346
C	-1.64536	-0.88939	0.20495	C	-1.36204	-0.18379	-0.71513
C	-0.79148	-0.62554	-1.05934	C	-0.84026	1.25216	-0.60396
C	-0.99365	0.81545	-1.53765	C	-1.41545	1.92647	0.66513
C	-2.47801	1.11125	-1.7738	C	-2.94456	1.85437	0.68508
C	-5.13992	-1.95512	0.799	C	-4.40391	-1.70122	-2.19464
C	-4.84453	1.04012	-0.95997	C	-5.0462	0.52733	0.39901
C	0.63509	-1.05554	-0.80363	C	0.66172	1.41989	-0.63781
C	1.63792	-0.14875	-0.6877	C	1.49631	0.3603	-0.48782
C	0.8398	-2.53961	-0.66268	C	1.14119	2.83395	-0.82286
C	3.03878	-0.45095	-0.41025	C	2.95473	0.41282	-0.47376
C	3.99957	0.72694	-0.42985	C	3.68519	-0.91962	-0.43304
O	3.4625	-1.60114	-0.19375	O	3.60299	1.47499	-0.51497

C	5.25351	0.55868	0.45284	C	5.03122	-0.89036	0.32214
O	5.98793	-0.60596	0.02839	O	5.91815	0.0521	-0.31057
C	6.19058	1.74639	0.25217	C	4.82698	-0.50184	1.79215
C	4.86929	0.41867	1.93186	C	5.71307	-2.25161	0.21618
H	-3.42829	-1.22033	-0.90451	H	-3.22245	0.43377	-1.54958
H	-1.17759	-1.29677	-1.84182	H	-1.22927	1.81829	-1.46461
H	-3.39089	3.50996	1.81811	H	-3.60469	-1.43839	3.56001
H	-4.81457	2.97381	0.77419	H	-5.06705	-0.87313	2.58728
H	-4.41467	-0.935	3.20081	H	-3.45252	-3.68768	-0.61806
H	-2.95574	0.04743	2.63652	H	-2.34718	-2.66412	0.45104
H	-1.97859	1.81287	0.90179	H	-2.08631	-0.46139	1.99364
H	-1.54434	-1.93303	0.51781	H	-0.98878	-0.64519	-1.63591
H	-1.26002	-0.27838	1.02794	H	-0.98154	-0.78957	0.11297
H	-0.59117	1.51617	-0.79858	H	-0.99127	1.43309	1.54718
H	-0.43398	0.98328	-2.46483	H	-1.10428	2.97466	0.71248
H	-2.60207	2.14603	-2.11325	H	-3.3244	2.31457	1.60478
H	-2.84608	0.466	-2.58262	H	-3.33542	2.44775	-0.15197
H	-5.80807	-1.5224	0.04504	H	-3.92797	-1.36208	-3.12452
H	-4.75443	-2.88968	0.36999	H	-5.26878	-1.04678	-2.03505
H	-5.73329	-2.20392	1.68383	H	-4.76658	-2.72187	-2.34836
H	-5.10142	0.29507	-1.71957	H	-5.49348	1.06046	1.24445
H	-5.53011	0.91624	-0.11704	H	-5.29037	1.0823	-0.51235
H	-5.01837	2.03102	-1.39223	H	-5.51951	-0.4564	0.33359
H	1.41422	0.90412	-0.81341	H	1.07339	-0.63093	-0.37598
H	1.64851	-2.88651	-1.31172	H	1.90847	2.89129	-1.59922
H	1.13625	-2.80211	0.35859	H	1.60694	3.21326	0.09362
H	-0.07385	-3.08506	-0.9099	H	0.31295	3.49546	-1.08737
H	4.31727	0.84885	-1.4756	H	3.03367	-1.69114	-0.01348
H	3.46565	1.64284	-0.15949	H	3.87654	-1.20183	-1.47827
H	5.31416	-1.31166	-0.01993	H	5.37706	0.85789	-0.42218
H	6.4771	1.83596	-0.80065	H	4.38492	0.49538	1.87693
H	7.1004	1.61066	0.84613	H	5.7903	-0.49307	2.31166
H	5.71206	2.67987	0.56301	H	4.16723	-1.21327	2.29994
H	4.22789	-0.45323	2.09047	H	5.86625	-2.52436	-0.83299
H	5.77068	0.29348	2.54003	H	6.68986	-2.22009	0.71013
H	4.33441	1.30629	2.28585	H	5.11066	-3.03005	0.69382

atom	Con f. 4- 7a			atom	Con f. 4- 7b		
C	-5.29467	-1.58152	1.06753	C	-5.19403	-1.79713	1.04181
C	-4.13631	2.95045	-0.59275	C	-4.38312	2.79842	-0.70348
C	-3.67661	1.95612	0.17675	C	-3.87666	1.85587	0.10098
C	-2.88805	0.81256	-0.44238	C	-2.97553	0.77153	-0.46782
C	-3.69606	-0.52576	-0.6164	C	-3.65909	-0.63517	-0.63194
C	-4.08778	-1.13305	0.7149	C	-4.02295	-1.25396	0.70204
C	-1.54488	0.59324	0.28102	C	-1.63811	0.68377	0.29273
C	-0.65827	-0.4224	-0.46693	C	-0.6473	-0.26651	-0.4113
C	-1.41068	-1.75436	-0.66568	C	-1.27604	-1.66104	-0.60711
C	-2.75836	-1.52545	-1.3535	C	-2.62066	-1.55984	-1.33088
C	-3.90221	1.96647	1.66784	C	-4.16303	1.86855	1.58125
C	-4.91677	-0.27709	-1.50919	C	-4.87758	-0.5107	-1.55285
C	0.6651	-0.6291	0.23128	C	0.66591	-0.33783	0.33155
C	1.80851	-0.32439	-0.4337	C	1.79171	0.09984	-0.28699
C	0.63613	-1.16119	1.6373	C	0.64679	-0.89273	1.7291
C	3.16855	-0.45728	0.07754	C	3.13686	0.11799	0.27896
C	4.29159	-0.11736	-0.88829	C	4.22685	0.71719	-0.59472
O	3.43029	-0.84951	1.22978	O	3.4015	-0.29576	1.42258
C	5.58106	0.40785	-0.22445	C	5.65486	0.22011	-0.29226
O	6.09063	-0.58059	0.69185	O	5.98598	0.5108	1.07938
C	6.66423	0.60445	-1.28168	C	5.77831	-1.28813	-0.54611
C	5.3168	1.71936	0.52721	C	6.66154	0.99013	-1.14254
H	-2.64785	1.12089	-1.46795	H	-2.73408	1.07689	-1.49396
H	-0.45437	-0.00997	-1.46266	H	-0.4501	0.14738	-1.40785
H	-5.45773	-2.03234	2.04316	H	-5.33532	-2.24672	2.02148
H	-6.16082	-1.51968	0.41556	H	-6.05141	-1.81685	0.37573
H	-4.69778	3.78254	-0.17423	H	-5.02558	3.58882	-0.32212
H	-3.96699	2.96625	-1.66649	H	-4.17303	2.81153	-1.77014
H	-3.28154	-1.23858	1.43749	H	-3.22348	-1.28388	1.4392
H	-1.71197	0.25677	1.30826	H	-1.8008	0.35732	1.3238
H	-1.01738	1.55164	0.34739	H	-1.19623	1.68506	0.34904
H	-0.79777	-2.42916	-1.27378	H	-0.59174	-2.28891	-1.18882
H	-1.55892	-2.24948	0.29959	H	-1.40711	-2.15377	0.36169
H	-3.28331	-2.48056	-1.46893	H	-3.05765	-2.55883	-1.4424
H	-2.58047	-1.13509	-2.36426	H	-2.45274	-1.17024	-2.34371
H	-4.42569	1.06488	1.99968	H	-3.24132	1.97409	2.16543

H	-2.95259	1.99717	2.21496	H	-4.82497	2.69857	1.8443
H	-4.49166	2.83737	1.96796	H	-4.63436	0.93574	1.90507
H	-5.64415	0.38321	-1.03012	H	-5.67017	0.09115	-1.10106
H	-4.60725	0.1911	-2.44924	H	-4.59073	-0.03325	-2.49553
H	-5.41964	-1.21753	-1.7561	H	-5.29071	-1.49601	-1.79147
H	1.7251	0.05542	-1.44807	H	1.70542	0.48366	-1.29979
H	1.07462	-0.43779	2.33278	H	1.29867	-1.76838	1.80806
H	-0.38023	-1.38316	1.96455	H	1.03579	-0.16049	2.44353
H	1.24087	-2.06875	1.72459	H	-0.35975	-1.17975	2.03681
H	4.52582	-1.04586	-1.42878	H	3.9835	0.55293	-1.64891
H	3.93298	0.59699	-1.63509	H	4.18773	1.80445	-0.43355
H	5.31712	-0.83329	1.2322	H	5.23042	0.16153	1.58973
H	7.5946	0.93289	-0.80693	H	6.79677	-1.6201	-0.32115
H	6.3649	1.36123	-2.01271	H	5.55904	-1.53155	-1.59103
H	6.85961	-0.33377	-1.81063	H	5.08797	-1.85141	0.08853
H	4.57068	1.57832	1.31487	H	7.67999	0.66972	-0.89943
H	6.24146	2.07246	0.99441	H	6.48982	0.81346	-2.20853
H	4.95507	2.49744	-0.15305	H	6.58384	2.06526	-0.95111
atom		Con f. 4- 7c		atom		Con f. 4- 7d	
C	-5.19425	-1.79682	1.04183	C	-5.29726	-1.57952	1.06652
C	-4.38323	2.79836	-0.70343	C	-4.13905	2.95141	-0.58725
C	-3.87668	1.85586	0.10102	C	-3.67791	1.95613	0.17997
C	-2.97555	0.77154	-0.46782	C	-2.88954	0.81378	-0.44171
C	-3.65912	-0.63518	-0.63198	C	-3.69769	-0.52432	-0.61673
C	-4.02304	-1.25397	0.70197	C	-4.09021	-1.13132	0.71448
C	-1.63814	0.68375	0.29276	C	-1.54614	0.59313	0.28121
C	-0.64731	-0.26648	-0.41126	C	-0.66016	-0.42266	-0.46813
C	-1.27606	-1.66104	-0.60709	C	-1.41302	-1.75389	-0.66534
C	-2.62066	-1.55984	-1.33088	C	-2.76068	-1.52476	-1.35332
C	-4.16289	1.86859	1.58132	C	-3.90083	1.96412	1.67145
C	-4.87757	-0.51071	-1.55294	C	-4.91793	-0.27549	-1.50999
C	0.66591	-0.33791	0.33156	C	0.66405	-0.6273	0.22906
C	1.79173	0.09961	-0.28704	C	1.80608	-0.31124	-0.43257
C	0.64678	-0.89274	1.72914	C	0.63665	-1.17579	1.62875
C	3.13689	0.11771	0.27888	C	3.16748	-0.44825	0.07487
C	4.22683	0.71706	-0.59476	C	4.28775	-0.09368	-0.88923
O	3.40158	-0.29621	1.42243	O	3.43203	-0.8588	1.21988
C	5.65491	0.22023	-0.29223	C	5.58474	0.40782	-0.22196
O	5.98588	0.51094	1.07944	O	6.09402	-0.60584	0.66643
C	5.77865	-1.28797	-0.54614	C	6.66237	0.6219	-1.28146
C	6.6615	0.99047	-1.14244	C	5.3342	1.70379	0.56054
H	-2.73411	1.07691	-1.49395	H	-2.64968	1.12377	-1.46693
H	-0.45011	0.14741	-1.40781	H	-0.45788	-0.00972	-1.46406
H	-5.33558	-2.24642	2.02149	H	-5.46075	-2.03038	2.04211
H	-6.05169	-1.81626	0.37582	H	-6.16308	-1.51742	0.4141
H	-5.02567	3.58876	-0.32204	H	-4.70012	3.78273	-0.1665
H	-4.17324	2.81145	-1.7701	H	-3.97123	2.96891	-1.66126
H	-3.22351	-1.28417	1.43906	H	-3.28412	-1.23667	1.43738
H	-1.80088	0.35728	1.32381	H	-1.71367	0.25439	1.30762
H	-1.19628	1.68505	0.34912	H	-1.0183	1.55115	0.34956
H	-0.59175	-2.28889	-1.1888	H	-0.80094	-2.4301	-1.27276
H	-1.40711	-2.15376	0.36172	H	-1.56186	-2.24782	0.30054
H	-3.05763	-2.55884	-1.44243	H	-3.28639	-2.47966	-1.46754
H	-2.4527	-1.17024	-2.34371	H	-2.58278	-1.13551	-2.36453
H	-3.24113	1.97419	2.16539	H	-4.49108	2.83379	1.97386
H	-4.82483	2.69861	1.8444	H	-4.42227	1.0612	2.00291
H	-4.63415	0.93578	1.90522	H	-2.95011	1.99549	2.21672
H	-5.67016	0.09117	-1.10121	H	-5.64458	0.38655	-1.03219
H	-4.59067	-0.03329	-2.49562	H	-4.60728	0.19083	-2.45064
H	-5.2907	-1.49602	-1.79154	H	-5.42195	-1.21565	-1.75576
H	1.70544	0.48345	-1.29983	H	1.72101	0.07888	-1.44297
H	-0.3598	-1.17951	2.03695	H	1.22244	-2.09769	1.69717
H	1.29844	-1.76856	1.80806	H	1.09586	-0.47193	2.33019
H	1.03603	-0.16057	2.4435	H	-0.38131	-1.38275	1.96142
H	3.98353	0.55272	-1.64895	H	4.51217	-1.01104	-1.45265
H	4.18752	1.80431	-0.43364	H	3.93006	0.63933	-1.6183
H	5.2304	0.16142	1.58973	H	5.32432	-0.86166	1.21027
H	6.79714	-1.61979	-0.32106	H	7.59736	0.93603	-0.80601
H	5.55956	-1.53136	-1.5911	H	6.3617	1.39515	-1.99459
H	5.08832	-1.85141	0.08837	H	6.85015	-0.30568	-1.83171
H	7.67999	0.67016	-0.89937	H	4.59719	1.54793	1.35397
H	6.48979	0.81388	-2.20844	H	6.26565	2.04452	1.02354
H	6.58366	2.06556	-0.9509	H	4.96571	2.497	-0.0984
atom		Con f. 4- 7e		atom		Con f. 4- 7f	
C	4.05876	2.62156	1.3324	C	-4.44976	0.38593	2.5505

C	5.20176	-1.98073	0.2172	C	-4.07183	2.19745	-1.95144
C	4.17454	-1.26698	0.69399	C	-3.29794	1.83098	-0.9224
C	3.19508	-0.60451	-0.26238	C	-2.84581	0.38613	-0.77979
C	3.42698	0.93668	-0.47546	C	-3.65766	-0.45339	0.27588
C	3.2249	1.7236	0.80266	C	-3.48266	0.07653	1.68381
C	1.73454	-0.93464	0.10137	C	-1.32362	0.28634	-0.56101
C	0.75164	-0.42638	-0.9794	C	-0.84229	-1.16416	-0.64732
C	0.92655	1.08358	-1.16261	C	-1.58687	-2.02023	0.40425
C	2.37734	1.41799	-1.51933	C	-3.10249	-1.90648	0.22722
C	3.94827	-1.13662	2.17945	C	-2.82996	2.84507	0.09107
C	4.82109	1.16913	-1.06857	C	-5.1345	-0.50036	-0.13085
C	-0.6454	-0.91078	-0.66486	C	0.64853	-1.38107	-0.52609
C	-1.61104	-0.05777	-0.23987	C	1.47144	-0.40688	-0.06339
C	-0.86462	-2.3886	-0.8449	C	1.12802	-2.74634	-0.93854
C	-2.97907	-0.42353	0.11326	C	2.91539	-0.51971	0.1198
C	-3.8792	0.70332	0.59322	C	3.62688	0.72177	0.6338
O	-3.4106	-1.58998	0.06273	O	3.55425	-1.56618	-0.09367
C	-5.38242	0.49596	0.31378	C	5.11305	0.83226	0.23641
O	-5.83009	-0.71269	0.95734	O	5.83153	-0.31466	0.7301
C	-6.1861	1.63168	0.94102	C	5.26847	0.92517	-1.28728
C	-5.65436	0.41132	-1.19399	C	5.74055	2.04758	0.91365
H	3.37676	-1.04679	-1.25025	H	-3.0605	-0.10342	-1.73855
H	1.03879	-0.91561	-1.92283	H	-1.12923	-1.55704	-1.63497
H	3.79735	3.15339	2.24385	H	-4.20852	0.73094	3.55282
H	5.02298	2.86302	0.89506	H	-5.50583	0.3151	2.30796
H	5.91373	-2.46842	0.87913	H	-4.40325	3.22552	-2.07846
H	5.36598	-2.10136	-0.85069	H	-4.40051	1.48224	-2.70136
H	2.28163	1.55132	1.31716	H	-2.45405	0.18163	2.02305
H	1.456	-0.48987	1.0623	H	-1.05318	0.71123	0.41035
H	1.64093	-2.01905	0.2185	H	-0.81666	0.89255	-1.32036
H	0.26091	1.44603	-1.9542	H	-1.29152	-3.07087	0.31915
H	0.63993	1.60945	-0.24538	H	-1.28504	-1.68777	1.4043
H	2.48899	2.4998	-1.65438	H	-3.61132	-2.50341	0.99294
H	2.62255	0.95029	-2.48213	H	-3.37471	-2.3368	-0.74543
H	3.92929	-0.08866	2.49334	H	-3.12848	2.56573	1.10587
H	2.9859	-1.57106	2.47471	H	-1.73661	2.92687	0.09628
H	4.73479	-1.64935	2.7405	H	-3.24032	3.83495	-0.12799
H	5.61377	0.89473	-0.36811	H	-5.60486	0.48469	-0.07749
H	4.95012	0.56686	-1.97357	H	-5.23065	-0.86154	-1.15977
H	4.95845	2.21944	-1.34519	H	-5.69629	-1.1826	0.51531
H	-1.37879	0.9966	-0.14497	H	1.052	0.55794	0.19718
H	-1.15439	-2.86686	0.09605	H	1.93703	-2.67152	-1.67072
H	-1.68529	-2.57544	-1.5444	H	1.53963	-3.2963	-0.08552
H	0.03694	-2.87487	-1.22436	H	0.31327	-3.33258	-1.36981
H	-3.73026	0.77776	1.68021	H	3.08267	1.61609	0.31576
H	-3.54267	1.65293	0.16719	H	3.55466	0.68735	1.73067
H	-5.17543	-1.3865	0.69003	H	5.29813	-1.07853	0.43728
H	-7.25671	1.46461	0.78332	H	4.85903	0.03789	-1.77909
H	-5.91815	2.59413	0.49515	H	6.32857	1.00104	-1.54909
H	-6.0008	1.68354	2.0188	H	4.751	1.80553	-1.68269
H	-5.11513	-0.42761	-1.64387	H	6.80532	2.10687	0.66521
H	-6.72425	0.26293	-1.37093	H	5.25773	2.97244	0.58436
H	-5.34394	1.33022	-1.7024	H	5.64462	1.97309	2.00165
atom		Con f. 4- 7g		atom		Con f. 4- 7h	
C	3.8145	2.82233	1.06228	C	-4.07012	0.82164	2.67752
C	5.0814	-1.93736	0.75864	C	-4.32799	1.75246	-2.12517
C	4.0344	-1.14596	1.02231	C	-3.42849	1.59922	-1.14566
C	3.15836	-0.63644	-0.1112	C	-2.91457	0.21347	-0.78798
C	3.41276	0.86082	-0.51855	C	-3.55901	-0.41133	0.50428
C	3.06087	1.82334	0.59708	C	-3.21745	0.37727	1.75139
C	1.66748	-0.93081	0.14502	C	-1.3745	0.16988	-0.7509
C	0.80095	-0.59729	-1.09276	C	-0.85653	-1.26467	-0.6182
C	1.00677	0.86729	-1.48923	C	-1.44386	-1.91351	0.65793
C	2.48895	1.16171	-1.73436	C	-2.97242	-1.84426	0.65472
C	3.68625	-0.77308	2.44133	C	-2.88425	2.79373	-0.40325
C	4.86614	1.03014	-0.97499	C	-5.0743	-0.54163	0.31343
C	-0.62577	-1.03249	-0.84503	C	0.64545	-1.43433	-0.63718
C	-1.62412	-0.12698	-0.68851	C	1.4803	-0.37425	-0.4927
C	-0.83564	-2.52024	-0.76573	C	1.12413	-2.85127	-0.80089
C	-3.02634	-0.43345	-0.4213	C	2.93843	-0.42895	-0.4658
C	-3.98543	0.74597	-0.41728	C	3.66988	0.90278	-0.41923
O	-3.45238	-1.58755	-0.23221	O	3.58534	-1.49208	-0.50129
C	-5.23969	0.5625	0.46198	C	5.01348	0.87091	0.3399
O	-5.97551	-0.59327	0.01624	O	5.90161	-0.07087	-0.29218
C	-4.85542	0.3946	1.938	C	5.69673	2.23186	0.23889

C	-6.17531	1.75493	0.28362	C	4.80474	0.47953	1.80856
H	3.43889	-1.21236	-1.00238	H	-3.23124	-0.45384	-1.59986
H	1.17303	-1.22571	-1.91652	H	-1.23961	-1.84449	-1.47218
H	3.4466	3.47657	1.84874	H	-3.7108	1.35288	3.55531
H	4.81407	3.03083	0.6928	H	-5.14472	0.68301	2.60609
H	5.72068	-2.32014	1.55092	H	-4.70713	2.7331	-2.40353
H	5.33483	-2.22905	-0.25758	H	-4.71556	0.90287	-2.68197
H	2.07262	1.69626	1.03389	H	-2.15819	0.56561	1.9136
H	1.29033	-0.35901	0.99929	H	-0.9995	0.77738	0.07844
H	1.56245	-1.9887	0.40574	H	-0.98647	0.62207	-1.6707
H	0.43262	1.0952	-2.39444	H	-1.13073	-2.95954	0.73264
H	0.62581	1.52566	-0.70097	H	-1.03341	-1.39812	1.53413
H	2.61798	2.21159	-2.02087	H	-3.36521	-2.29061	1.57559
H	2.83842	0.55545	-2.58068	H	-3.34965	-2.45004	-0.17969
H	4.40152	-1.20638	3.14621	H	-1.7996	2.88424	-0.53553
H	3.6803	0.31217	2.57985	H	-3.34543	3.71922	-0.7596
H	2.68593	-1.12991	2.71339	H	-3.06346	2.71245	0.67308
H	5.57179	0.87978	-0.15397	H	-5.56414	0.43314	0.24794
H	5.10299	0.30247	-1.75788	H	-5.29168	-1.08816	-0.60996
H	5.03223	2.02978	-1.38926	H	-5.52636	-1.09608	1.1422
H	-1.39645	0.92904	-0.77432	H	1.05816	0.61878	-0.39464
H	0.0682	-3.05844	-1.06064	H	1.88278	-2.92157	-1.58491
H	-1.10539	-2.82652	0.25091	H	1.60084	-3.21367	0.11662
H	-1.66364	-2.83407	-1.40691	H	0.294	-3.51808	-1.04531
H	-3.45043	1.65572	-0.12899	H	3.86428	1.18793	-1.46311
H	-4.30274	0.88864	-1.46054	H	3.01735	1.67346	0.00042
H	-5.30217	-1.29815	-0.04805	H	5.36082	-0.87666	-0.40504
H	-5.75692	0.26028	2.54407	H	6.67183	2.19868	0.73607
H	-4.31842	1.27435	2.30805	H	5.09329	3.0098	0.71603
H	-4.216	-0.48156	2.08049	H	5.85365	2.50658	-0.80921
H	-7.08513	1.60932	0.87524	H	4.3614	-0.51738	1.89011
H	-5.69551	2.68188	0.61152	H	5.76663	0.4687	2.33066
H	-6.46194	1.86433	-0.76728	H	4.14437	1.19063	2.31602
atom		Con f. 4- 7i		atom		Con f. 4- 7j	
C	3.81473	2.82233	1.06188	C	-5.29404	-1.4606	1.14959
C	5.08146	-1.93696	0.7592	C	-3.81339	1.86532	1.74231
C	4.03424	-1.14577	1.02265	C	-3.57364	1.91476	0.42706
C	3.15828	-0.63649	-0.11104	C	-2.83396	0.81359	-0.32016
C	3.41277	0.86067	-0.51872	C	-3.69919	-0.47394	-0.57886
C	3.06097	1.8234	0.59676	C	-4.06765	-1.15743	0.72042
C	1.6674	-0.93073	0.14516	C	-1.47952	0.48204	0.33308
C	0.80091	-0.59742	-1.09272	C	-0.66136	-0.50011	-0.53134
C	1.00677	0.86709	-1.48945	C	-1.47093	-1.78168	-0.81176
C	2.48898	1.16137	-1.73458	C	-2.83281	-1.4467	-1.42693
C	3.68582	-0.7729	2.44161	C	-4.00342	3.09552	-0.40409
C	4.86615	1.02981	-0.97518	C	-4.94237	-0.10252	-1.39466
C	-0.62582	-1.03256	-0.84495	C	0.67911	-0.79817	0.09757
C	-1.62411	-0.127	-0.68829	C	1.80365	-0.3774	-0.53533
C	-0.83581	-2.5203	-0.76587	C	0.68482	-1.53355	1.4089
C	-3.02638	-0.43348	-0.42144	C	3.17618	-0.55548	-0.0742
C	-3.98553	0.74589	-0.41756	C	4.26534	0.08829	-0.91692
O	-3.45245	-1.58762	-0.23258	O	3.47728	-1.21568	0.93741
C	-5.23958	0.5626	0.46207	C	5.51455	0.52853	-0.1248
O	-5.97552	-0.59324	0.01678	O	6.10523	-0.61695	0.5189
C	-4.8549	0.395	1.93802	C	6.57104	1.069	-1.08443
C	-6.17521	1.75501	0.28369	C	5.15351	1.58512	0.92797
H	3.43887	-1.21263	-1.00207	H	-2.60849	1.20272	-1.32225
H	1.173	-1.22598	-1.91636	H	-0.47913	-0.00852	-1.49499
H	3.4469	3.47668	1.84828	H	-5.44112	-1.97441	2.09638
H	4.81434	3.03066	0.69242	H	-6.19291	-1.21623	0.59135
H	5.72072	-2.31951	1.55159	H	-4.34187	2.67001	2.24804
H	5.33508	-2.22868	-0.25697	H	-3.49762	1.03119	2.36018
H	2.0727	1.69649	1.03357	H	-3.23069	-1.44196	1.35459
H	1.29023	-0.35875	0.99929	H	-1.63113	0.06864	1.33431
H	1.56229	-1.98858	0.40606	H	-0.91112	1.40975	0.46062
H	0.43265	1.09485	-2.39471	H	-0.90758	-2.42489	-1.49729
H	0.62581	1.52562	-0.70133	H	-1.6043	-2.35191	0.11332
H	2.6181	2.21118	-2.02133	H	-3.39914	-2.36952	-1.59843
H	2.83843	0.5549	-2.58077	H	-2.67473	-0.98388	-2.41002
H	2.68566	-1.13021	2.71362	H	-4.54281	3.83915	0.19006
H	4.40125	-1.20581	3.14657	H	-3.13102	3.58471	-0.85758
H	3.67935	0.31235	2.58007	H	-4.64765	2.78307	-1.23428
H	5.57179	0.87951	-0.15414	H	-5.6095	0.56127	-0.83784
H	5.10295	0.30202	-1.75799	H	-4.65016	0.4057	-2.31897
H	5.03231	2.0294	-1.38958	H	-5.51064	-0.99676	-1.67087

H	-1.39637	0.92902	-0.77392	H	1.69195	0.16428	-1.47065
H	-1.10614	-2.82667	0.25058	H	1.12075	-2.5316	1.28762
H	-1.66349	-2.83401	-1.40756	H	1.30595	-1.01876	2.14669
H	0.06814	-3.05853	-1.0604	H	-0.32296	-1.64843	1.81089
H	-3.45054	1.65577	-0.12966	H	4.56816	-0.66134	-1.66209
H	-4.30312	0.88823	-1.46077	H	3.85342	0.93511	-1.47331
H	-5.30218	-1.29809	-0.0478	H	5.35571	-1.05805	0.96374
H	-4.21549	-0.48117	2.08049	H	7.4763	1.33891	-0.53071
H	-5.75625	0.26083	2.54435	H	6.20742	1.9596	-1.60543
H	-4.31778	1.2748	2.30776	H	6.83567	0.31251	-1.83018
H	-6.46206	1.86422	-0.76717	H	4.42968	1.19234	1.64823
H	-7.0849	1.60952	0.87553	H	6.05168	1.88432	1.47741
H	-5.69533	2.68202	0.61132	H	4.72228	2.47651	0.46031
atom		Con f. 4- 7k		atom		Con f. 4- 7l	
C	-5.16999	-1.85353	0.95499	C	-4.07081	0.82152	2.67734
C	-4.22985	1.63619	1.67037	C	-4.3278	1.75296	-2.12482
C	-3.91743	1.74786	0.37426	C	-3.42824	1.59951	-1.1454
C	-2.98225	0.77841	-0.33566	C	-2.91447	0.21365	-0.78789
C	-3.63666	-0.61323	-0.66201	C	-3.55915	-0.41136	0.50417
C	-3.97705	-1.37032	0.60367	C	-3.2179	0.37709	1.75147
C	-1.6393	0.63052	0.40288	C	-1.3744	0.16992	-0.75066
C	-0.63505	-0.21824	-0.40534	C	-0.85654	-1.26468	-0.61808
C	-1.23853	-1.59121	-0.7666	C	-1.44404	-1.91363	0.65791
C	-2.59029	-1.43247	-1.46773	C	-2.9726	-1.84431	0.65452
C	-4.45762	2.87415	-0.46795	C	-2.8838	2.79389	-0.40292
C	-4.87067	-0.40641	-1.54703	C	-5.07439	-0.54165	0.31298
C	0.68	-0.35737	0.32438	C	0.64543	-1.43443	-0.63691
C	1.80029	0.15932	-0.24155	C	1.48033	-0.37439	-0.49247
C	0.6694	-1.06685	1.65038	C	1.12403	-2.85141	-0.80055
C	3.14686	0.12209	0.31898	C	2.93846	-0.42918	-0.46555
C	4.23238	0.82593	-0.47849	C	3.67001	0.90252	-0.41961
O	3.41931	-0.42349	1.4043	O	3.5853	-1.49236	-0.50057
C	5.65291	0.2565	-0.28388	C	5.01362	0.87094	0.33951
O	6.01924	0.33928	1.10708	O	5.90162	-0.07134	-0.292
C	5.72873	-1.20224	-0.75387	C	5.69703	2.23174	0.23765
C	6.66282	1.11611	-1.03912	C	4.80486	0.48048	1.80842
H	-2.75348	1.21434	-1.31745	H	-3.23109	-0.45348	-1.59992
H	-0.44516	0.31316	-1.3461	H	-1.23956	-1.84437	-1.47218
H	-5.29632	-2.40281	1.88469	H	-3.71171	1.35265	3.55529
H	-6.06076	-1.72748	0.34681	H	-5.14541	0.68307	2.60554
H	-4.89885	2.34604	2.15106	H	-4.70685	2.73367	-2.40304
H	-3.83777	0.84112	2.29603	H	-4.71552	0.90348	-2.68167
H	-3.14513	-1.54586	1.28226	H	-2.15867	0.56528	1.91405
H	-1.79787	0.19161	1.39195	H	-0.99941	0.7773	0.07877
H	-1.21498	1.62679	0.56893	H	-0.98625	0.62218	-1.67038
H	-0.54518	-2.12861	-1.4234	H	-1.13098	-2.95969	0.73251
H	-1.35107	-2.20222	0.13495	H	-1.03365	-1.39838	1.53421
H	-3.00912	-2.42012	-1.69342	H	-3.36552	-2.29077	1.57529
H	-2.43609	-0.92555	-2.42945	H	-3.34974	-2.44999	-0.18
H	-3.63721	3.49509	-0.85198	H	-1.79919	2.88445	-0.53554
H	-4.99244	2.4926	-1.34559	H	-3.34512	3.71944	-0.75894
H	-5.13784	3.51759	0.0979	H	-3.06265	2.71239	0.67345
H	-5.657	0.14496	-1.02394	H	-5.56424	0.43312	0.24762
H	-4.60165	0.1561	-2.44675	H	-5.29156	-1.08798	-0.61058
H	-5.28845	-1.36687	-1.86638	H	-5.5266	-1.09631	1.14153
H	1.70704	0.65672	-1.20291	H	1.05826	0.61868	-0.39454
H	1.31896	-1.94758	1.62541	H	1.88342	-2.92167	-1.58384
H	-0.33561	-1.38483	1.93154	H	1.59977	-3.2141	0.11735
H	1.06558	-0.42095	2.44	H	0.294	-3.51801	-1.04588
H	3.96807	0.82827	-1.54005	H	3.86441	1.18713	-1.46364
H	4.22477	1.87613	-0.15252	H	3.01753	1.67347	-0.00036
H	5.25849	-0.04858	1.58124	H	5.3607	-0.87711	-0.40437
H	6.74292	-1.5872	-0.60828	H	6.67212	2.19877	0.73485
H	5.47599	-1.28766	-1.81601	H	5.09368	3.01005	0.71432
H	5.03956	-1.83426	-0.18604	H	5.85398	2.50579	-0.81062
H	7.67649	0.73723	-0.87203	H	4.36143	-0.51634	1.89058
H	6.46263	1.10197	-2.11464	H	5.76676	0.46988	2.3305
H	6.62093	2.1532	-0.69091	H	4.14457	1.19196	2.31544
atom		Con f. 4- 7m		atom		Con f. 4- 7n	
C	4.05869	2.6216	1.33229	C	4.44887	-0.38559	2.55119
C	5.2018	-1.98056	0.21773	C	4.07182	-2.19713	-1.95199
C	4.17441	-1.26693	0.69432	C	3.2981	-1.83084	-0.92275
C	3.19506	-0.60456	-0.26223	C	2.84591	-0.38603	-0.77985
C	3.42704	0.93659	-0.4755	C	3.65751	0.45323	0.27616
C	3.22484	1.72366	0.8025	C	3.48203	-0.07674	1.68402

C	1.73446	-0.93458	0.10141	C	1.32364	-0.28628	-0.5614
C	0.75172	-0.42644	-0.97956	C	0.84238	1.16428	-0.64748
C	0.9267	1.08349	-1.16293	C	1.58683	2.02016	0.40431
C	2.37754	1.4178	-1.51954	C	3.10248	1.9064	0.22753
C	3.9479	-1.13653	2.17974	C	2.83043	-2.84508	0.0907
C	4.82122	1.16892	-1.06848	C	5.1345	0.50007	-0.13007
C	-0.64537	-0.91077	-0.66514	C	-0.64847	1.38111	-0.52639
C	-1.61107	-0.05767	-0.24048	C	-1.47129	0.40691	-0.06356
C	-0.86461	-2.38863	-0.84489	C	-1.12803	2.74633	-0.93895
C	-2.97907	-0.42344	0.11275	C	-2.91525	0.51963	0.11948
C	-3.8791	0.70337	0.59296	C	-3.62668	-0.72184	0.63363
O	-3.4106	-1.58989	0.06219	O	-3.5542	1.566	-0.09417
C	-5.3824	0.49596	0.31401	C	-5.11293	-0.83228	0.23661
O	-5.82982	-0.71269	0.95771	O	-5.83128	0.31472	0.73047
C	-6.18592	1.63166	0.94149	C	-5.26878	-0.92518	-1.28702
C	-5.65483	0.41131	-1.19367	C	-5.74033	-2.04757	0.91401
H	3.37682	-1.04698	-1.25002	H	3.06078	0.10376	-1.73845
H	1.039	-0.91581	-1.92289	H	1.1294	1.55728	-1.63505
H	3.79719	3.15354	2.24365	H	4.20734	-0.73052	3.55347
H	5.02299	2.86295	0.89507	H	5.50502	-0.31434	2.3091
H	5.91372	-2.46815	0.87977	H	4.40331	-3.22515	-2.0792
H	5.36621	-2.10121	-0.85014	H	4.40027	-1.4818	-2.70189
H	2.28146	1.55152	1.31686	H	2.45331	-0.18218	2.02279
H	1.45583	-0.48966	1.06224	H	1.05295	-0.71142	0.40977
H	1.6408	-2.01898	0.21869	H	0.81686	-0.89229	-1.32103
H	0.26116	1.44588	-1.95463	H	1.29154	3.07084	0.31935
H	0.64001	1.60947	-0.24579	H	1.28489	1.68758	1.40428
H	2.48923	2.49959	-1.65473	H	3.6112	2.5032	0.99342
H	2.62284	0.94997	-2.48225	H	3.37491	2.3368	-0.74502
H	3.92876	-0.08856	2.49361	H	1.73708	-2.92706	0.096
H	2.98554	-1.57107	2.47487	H	3.24091	-3.83488	-0.12847
H	4.73439	-1.64915	2.74093	H	3.129	-2.5658	1.10549
H	5.61382	0.89453	-0.36792	H	5.60474	-0.48504	-0.07666
H	4.95032	0.56658	-1.97343	H	5.23104	0.86136	-1.15892
H	4.95865	2.2192	-1.34516	H	5.69619	1.18218	0.51631
H	-1.37885	0.99672	-0.14579	H	-1.05176	-0.55782	0.1972
H	0.037	-2.87504	-1.22403	H	-1.93645	2.67133	-1.6718
H	-1.15458	-2.86665	0.09613	H	-1.5405	3.29607	-0.08623
H	-1.68516	-2.5756	-1.54447	H	-0.31315	3.33284	-1.36961
H	-3.72979	0.7779	1.6799	H	-3.08256	-1.61616	0.31543
H	-3.54274	1.65299	0.16678	H	-3.55414	-0.68745	1.73048
H	-5.17527	-1.3865	0.69011	H	-5.29802	1.07862	0.43747
H	-7.25657	1.46458	0.78408	H	-6.32895	-1.00103	-1.54853
H	-5.91809	2.59411	0.49555	H	-4.75144	-1.80555	-1.68259
H	-6.00033	1.6835	2.01922	H	-4.85945	-0.03791	-1.77894
H	-6.72477	0.26285	-1.37025	H	-6.80518	-2.10675	0.66586
H	-5.34466	1.33022	-1.70221	H	-5.2577	-2.97247	0.58455
H	-5.11571	-0.42759	-1.64373	H	-5.64409	-1.97314	2.00198
atom		Con f. 4- 7o		atom		Con f. 4- 7p	
C	-5.17009	-1.85328	0.95523	C	-5.29223	-1.65675	0.97129
C	-4.22991	1.63635	1.66994	C	-4.06136	1.73899	1.71167
C	-3.91744	1.74782	0.37383	C	-3.75331	1.83709	0.41342
C	-2.9822	0.77827	-0.33585	C	-2.90663	0.80229	-0.31524
C	-3.63656	-0.61346	-0.66192	C	-3.6731	-0.53043	-0.64559
C	-3.97708	-1.37026	0.60389	C	-4.06672	-1.26605	0.61711
C	-1.63931	0.63055	0.4028	C	-1.56999	0.54361	0.40454
C	-0.63498	-0.21831	-0.40524	C	-0.64738	-0.36639	-0.4313
C	-1.23835	-1.59141	-0.76619	C	-1.35788	-1.6912	-0.77803
C	-2.59012	-1.43289	-1.46737	C	-2.70262	-1.42918	-1.46181
C	-4.4574	2.8741	-0.46852	C	-4.20741	3.01189	-0.41327
C	-4.8705	-0.40686	-1.5471	C	-4.89143	-0.21925	-1.52163
C	0.68007	-0.35712	0.32454	C	0.67675	-0.6044	0.25483
C	1.80028	0.15966	-0.24144	C	1.81727	-0.2535	-0.39193
C	0.66961	-1.06641	1.65064	C	0.64978	-1.22172	1.625
C	3.1468	0.12282	0.31921	C	3.18011	-0.40971	0.10564
C	4.23243	0.8263	-0.47841	C	4.29889	-0.09851	-0.875
O	3.41916	-0.42212	1.40489	O	3.44653	-0.80004	1.25739
C	5.65274	0.25616	-0.28406	C	5.60632	0.40647	-0.23071
O	6.01937	0.33877	1.10682	O	6.10923	-0.58721	0.68342
C	5.7278	-1.20263	-0.75403	C	6.67885	0.57675	-1.30329
C	6.66291	1.11526	-1.03951	C	5.37583	1.72755	0.5149
H	-2.75339	1.21401	-1.31772	H	-2.65488	1.22834	-1.2958
H	-0.44511	0.31291	-1.3461	H	-0.44747	0.15176	-1.37738
H	-5.29656	-2.40233	1.88505	H	-5.45676	-2.20198	1.8974
H	-6.0608	-1.72729	0.34694	H	-6.17363	-1.45515	0.36979

H	-4.89888	2.34631	2.15052	H	-4.66459	2.49718	2.20546
H	-3.83785	0.84137	2.29575	H	-3.73001	0.90855	2.32643
H	-3.14524	-1.54568	1.2826	H	-3.24801	-1.51413	1.28939
H	-1.79793	0.19176	1.39192	H	-1.74926	0.1012	1.38852
H	-1.21501	1.62684	0.56871	H	-1.07117	1.50321	0.57963
H	-0.54496	-2.12889	-1.42287	H	-0.71754	-2.28186	-1.44276
H	-1.35084	-2.20224	0.13549	H	-1.50202	-2.28844	0.12846
H	-3.00891	-2.42061	-1.69282	H	-3.19962	-2.38127	-1.68147
H	-2.4359	-0.92621	-2.42922	H	-2.51993	-0.93644	-2.42592
H	-4.99203	2.49256	-1.34628	H	-3.34249	3.56221	-0.80718
H	-5.13772	3.51757	0.09716	H	-4.78683	2.68421	-1.28442
H	-3.63689	3.49502	-0.85237	H	-4.82061	3.70763	0.1671
H	-5.65688	0.14458	-1.02416	H	-5.62849	0.38922	-0.99029
H	-4.60142	0.1555	-2.44688	H	-4.58296	0.3261	-2.41908
H	-5.28821	-1.36739	-1.86632	H	-5.38649	-1.14084	-1.84489
H	1.70701	0.65685	-1.20289	H	1.72898	0.17521	-1.38629
H	-0.33528	-1.38495	1.93162	H	-0.34626	-1.58378	1.88495
H	1.31969	-1.94676	1.62594	H	1.36269	-2.04656	1.70174
H	1.06521	-0.42015	2.44024	H	0.95178	-0.4869	2.38024
H	3.968	0.82896	-1.53993	H	4.50877	-1.03549	-1.411
H	4.2255	1.87641	-0.15218	H	3.9465	0.61649	-1.62407
H	5.25837	-0.04841	1.58115	H	5.33903	-0.82241	1.23586
H	6.74187	-1.58801	-0.60874	H	7.62052	0.89489	-0.84392
H	5.47471	-1.28802	-1.81609	H	6.38136	1.33129	-2.03747
H	5.03856	-1.83437	-0.18597	H	6.85271	-0.369	-1.82656
H	7.67644	0.736	-0.87245	H	4.64127	1.60353	1.31615
H	6.46265	1.10104	-2.11502	H	6.31382	2.0706	0.96272
H	6.62146	2.15241	-0.69145	H	5.01301	2.50532	-0.16531
atom	Con f. 4- 7q			atom	Con f. 4- 7r		
C	4.03306	2.62625	1.35453	C	-4.29137	0.45997	2.63559
C	4.11172	-0.98445	2.08914	C	-3.02468	2.75747	0.07856
C	4.18242	-1.17724	0.76714	C	-3.34237	1.819	-0.82036
C	3.18348	-0.5726	-0.20967	C	-2.85543	0.38024	-0.71736
C	3.39554	0.96285	-0.4707	C	-3.61921	-0.475	0.3596
C	3.17821	1.77125	0.7902	C	-3.37357	0.04976	1.75832
C	1.72976	-0.90849	0.17181	C	-1.32691	0.2967	-0.54733
C	0.73631	-0.44234	-0.91991	C	-0.83379	-1.14923	-0.66018
C	0.89777	1.06106	-1.16327	C	-1.5442	-2.03341	0.39296
C	2.34548	1.40053	-1.53049	C	-3.06617	-1.92471	0.27013
C	5.25331	-2.04412	0.15792	C	-4.18481	2.14283	-2.02666
C	4.79352	1.19429	-1.05558	C	-5.1139	-0.5079	0.02322
C	-0.65607	-0.92402	-0.58201	C	0.66088	-1.35522	-0.57115
C	-1.63075	-0.06087	-0.19989	C	1.48218	-0.3872	-0.09315
C	-0.86112	-2.41103	-0.68834	C	1.1457	-2.70506	-1.02633
C	-2.99626	-0.42189	0.16674	C	2.92857	-0.49532	0.07395
C	-3.91663	0.72134	0.56228	C	3.63357	0.73669	0.61845
O	-3.41156	-1.59515	0.19159	O	3.57228	-1.53107	-0.17363
C	-5.41145	0.48032	0.26555	C	5.12697	0.85095	0.2529
O	-5.86076	-0.69205	0.97201	O	5.83284	-0.30731	0.73884
C	-6.23994	1.64342	0.80424	C	5.31401	0.97324	-1.26524
C	-5.64981	0.30031	-1.23957	C	5.74384	2.05115	0.96589
H	3.36094	-1.04634	-1.18441	H	-3.09035	-0.10603	-1.67384
H	1.02227	-0.96539	-1.84555	H	-1.138	-1.52941	-1.64759
H	3.76091	3.17233	2.25443	H	-4.00097	0.79955	3.62677
H	5.02541	2.81934	0.95801	H	-5.35437	0.47832	2.41432
H	4.83741	-1.43401	2.76281	H	-3.38261	3.77925	-0.02386
H	3.33758	-0.37771	2.54727	H	-2.40436	2.54638	0.94365
H	2.20867	1.64524	1.26731	H	-2.33324	0.07104	2.07599
H	1.45639	-0.4419	1.12397	H	-1.03672	0.72289	0.41744
H	1.65055	-1.98973	0.31973	H	-0.85034	0.91332	-1.31722
H	0.2299	1.38373	-1.97008	H	-1.24648	-3.07995	0.2725
H	0.60169	1.62018	-0.2692	H	-1.21081	-1.72538	1.39062
H	2.444	2.47868	-1.70138	H	-3.54484	-2.53654	1.04376
H	2.59721	0.90521	-2.47771	H	-3.37115	-2.34168	-0.69869
H	5.94538	-2.42934	0.91256	H	-3.61859	1.9627	-2.95034
H	4.80481	-2.8992	-0.36536	H	-5.07066	1.49908	-2.07845
H	5.83219	-1.49291	-0.59259	H	-4.51578	3.1856	-2.02628
H	5.58034	0.93325	-0.3424	H	-5.56849	0.48388	0.09687
H	4.93292	0.58454	-1.95381	H	-5.26378	-0.87482	-0.99703
H	4.93135	2.24286	-1.33929	H	-5.65374	-1.17656	0.70181
H	-1.41018	0.9994	-0.15975	H	1.0586	0.56683	0.19895
H	0.03155	-2.90179	-1.0828	H	1.94336	-2.60416	-1.7678
H	-1.10042	-2.84935	0.28611	H	1.57433	-3.27385	-0.19432
H	-1.70875	-2.64137	-1.34029	H	0.32953	-3.28659	-1.46138
H	-3.7908	0.86093	1.6457	H	3.09606	1.63593	0.30219

H	-3.58192	1.64789	0.08705	H	3.53803	0.68798	1.71307
H	-5.19199	-1.37259	0.76262	H	5.30984	-1.06384	0.41093
H	-7.30515	1.45363	0.63574	H	6.37964	1.05006	-1.50321
H	-5.97327	2.57896	0.30363	H	4.80829	1.86358	-1.65344
H	-6.0776	1.76545	1.88003	H	4.91139	0.09774	-1.78298
H	-6.71426	0.13061	-1.42972	H	6.81315	2.1133	0.73848
H	-5.33635	1.18908	-1.79722	H	5.26867	2.98331	0.64617
H	-5.09363	-0.5595	-1.62487	H	5.6275	1.95537	2.05025
atom	Con f. 4- 7s			atom	Con f. 4- 7t		
C	3.77784	2.8295	1.06319	C	-3.77755	-2.8296	1.06317
C	3.77689	-0.61247	2.38077	C	-3.77727	0.61227	2.38069
C	4.00644	-1.0177	1.12673	C	-4.00658	1.01758	1.12665
C	3.13535	-0.59724	-0.04863	C	-3.13534	0.59717	-0.04862
C	3.38195	0.87802	-0.53087	C	-3.38185	-0.87805	-0.53085
C	3.00253	1.87765	0.54039	C	-3.00231	-1.87771	0.54036
C	1.64521	-0.88934	0.20512	C	-1.64523	0.88939	0.20516
C	0.79145	-0.62584	-1.05933	C	-0.79147	0.62597	-1.0593
C	0.99373	0.81501	-1.53802	C	-0.99367	-0.81485	-1.53814
C	2.47812	1.11059	-1.77422	C	-2.47806	-1.11054	-1.77427
C	5.1397	-1.95483	0.80001	C	-5.1398	1.9547	0.79971
C	4.84455	1.03975	-0.96009	C	-4.84445	-1.03989	-0.95997
C	-0.63515	-1.05574	-0.80367	C	0.63509	1.05591	-0.80354
C	-1.638	-0.14893	-0.68812	C	1.63799	0.14912	-0.68805
C	-0.83991	-2.53978	-0.66236	C	0.8398	2.53994	-0.66211
C	-3.03889	-0.45104	-0.41079	C	3.03883	0.45125	-0.41049
C	-3.99965	0.72687	-0.43034	C	3.99957	-0.72667	-0.43032
O	-3.46272	-1.60121	-0.19436	O	3.4626	1.60138	-0.19374
C	-5.25313	0.55887	0.45305	C	5.25315	-0.55896	0.4529
O	-5.98787	-0.6058	0.02923	O	5.98797	0.60579	0.02923
C	-4.86815	0.41906	1.93188	C	4.86836	-0.41941	1.93179
C	-6.19021	1.74663	0.25266	C	6.19013	-1.74671	0.25215
H	3.4283	-1.22068	-0.904	H	-3.42827	1.22061	-0.904
H	1.17762	-1.2973	-1.84158	H	-1.17765	1.29747	-1.84151
H	3.39058	3.51035	1.81719	H	-3.39021	-3.51046	1.81712
H	4.81451	2.97369	0.77385	H	-4.8142	-2.97387	0.77385
H	4.41397	-0.93399	3.20139	H	-4.41453	0.93373	3.2012
H	2.95513	0.04823	2.63651	H	-2.95555	-0.04841	2.63658
H	1.97827	1.81329	0.90084	H	-1.97804	-1.8133	0.90078
H	1.2598	-0.27808	1.02788	H	-1.25977	0.27819	1.02795
H	1.54414	-1.93289	0.51829	H	-1.54424	1.93295	0.5183
H	0.4341	0.98264	-2.46526	H	-0.4341	-0.9823	-2.46544
H	0.59132	1.51595	-0.79913	H	-0.59111	-1.51584	-0.79938
H	2.60227	2.14523	-2.11406	H	-2.60217	-2.14518	-2.11412
H	2.84621	0.465	-2.58275	H	-2.84624	-0.46495	-2.58277
H	5.80806	-1.52227	0.04614	H	-4.75432	2.88942	0.37106
H	5.73284	-2.20345	1.68505	H	-5.80786	1.52219	0.04556
H	4.75433	-2.88948	0.3711	H	-5.73322	2.20317	1.6846
H	5.53004	0.91617	-0.11703	H	-5.52986	-0.91631	-0.11684
H	5.10154	0.29443	-1.71939	H	-5.10155	-0.29459	-1.71926
H	5.01846	2.0305	-1.39268	H	-5.01833	-2.03065	-1.39252
H	-1.41428	0.90391	-0.81407	H	1.41433	-0.90371	-0.81415
H	0.07387	-3.08531	-0.90897	H	1.13673	2.80206	0.35912
H	-1.13688	-2.80199	0.35883	H	-0.07395	3.0855	-0.90872
H	-1.64828	-2.88687	-1.31172	H	1.64824	2.88708	-1.31137
H	-3.46561	1.64285	-0.16053	H	3.46551	-1.64266	-0.16059
H	-4.3179	0.84842	-1.47597	H	4.31759	-0.8481	-1.47603
H	-5.31419	-1.31155	-0.01952	H	5.31428	1.31155	-0.01925
H	-5.76925	0.29425	2.54057	H	5.76954	-0.29479	2.5404
H	-4.33282	1.30659	2.28539	H	4.33298	-1.30696	2.2852
H	-4.22692	-0.45299	2.09032	H	4.22721	0.45267	2.09047
H	-7.09976	1.61102	0.84708	H	7.09972	-1.61132	0.84656
H	-5.71148	2.68011	0.56315	H	5.71135	-2.68023	0.56241
H	-6.47723	1.83608	-0.80003	H	6.47709	-1.8359	-0.80059

atom	Con f. 4- 8a			atom	Con f. 4- 8b		
C	-5.7609	0.11371	-1.54868	C	5.56421	-0.0465	1.93795
C	-2.8324	2.30223	-0.97467	C	2.96266	2.3323	0.83215
C	-2.86385	1.94202	0.31317	C	3.09913	1.86289	-0.41297
C	-2.98141	0.51805	0.84561	C	3.14694	0.39435	-0.81944
C	-3.85108	-0.50403	0.0339	C	3.81037	-0.62503	0.17044
C	-5.15354	0.15784	-0.36273	C	5.11152	-0.04536	0.68404
C	-1.56756	-0.04679	1.13612	C	1.73247	-0.07218	-1.24816
C	-0.85561	-0.51254	-0.14821	C	0.82771	-0.36494	-0.03679
C	-1.69246	-1.60535	-0.84098	C	1.46582	-1.46392	0.83578

C	-3.0959	-1.09139	-1.17868	C	2.85926	-1.04197	1.31371
C	-2.74911	2.98497	1.39838	C	3.1971	2.816	-1.5795
C	-4.24111	-1.65877	0.9919	C	4.20339	-1.88584	-0.64128
C	0.55349	-0.96901	0.14435	C	-0.574	-0.7245	-0.46668
C	1.58794	-0.27385	-0.3937	C	-1.60044	0.06947	-0.06795
C	0.73101	-2.16712	1.0356	C	-0.75265	-1.95192	-1.31634
C	3.00711	-0.55445	-0.20826	C	-3.0122	-0.11823	-0.38284
C	3.98123	0.37472	-0.91432	C	-3.97507	0.92061	0.16858
O	3.43129	-1.51226	0.4648	O	-3.43657	-1.05324	-1.08711
C	5.34808	0.53091	-0.21557	C	-5.39982	0.39845	0.4481
O	5.98635	-0.7564	-0.11025	O	-5.97571	-0.10769	-0.77165
C	6.27114	1.39274	-1.07268	C	-5.38176	-0.7088	1.51005
C	5.18339	1.14307	1.18166	C	-6.29467	1.55222	0.8931
H	-3.47767	0.61359	1.82025	H	3.7651	0.36593	-1.72632
H	-0.79766	0.34707	-0.82519	H	0.77011	0.54909	0.56474
H	-6.72135	0.59865	-1.70349	H	6.54023	0.36611	2.18037
H	-5.33414	-0.395	-2.40829	H	4.99068	-0.44699	2.76885
H	-2.72096	3.34746	-1.25404	H	2.91519	3.40319	1.01648
H	-2.93126	1.59774	-1.79022	H	2.91384	1.69481	1.70537
H	-5.64584	0.68714	0.45525	H	5.74698	0.37331	-0.0986
H	-1.64807	-0.88423	1.8352	H	1.81908	-0.96943	-1.86783
H	-0.96469	0.71944	1.63417	H	1.27045	0.69651	-1.87609
H	-1.19182	-1.92668	-1.76121	H	0.82865	-1.66247	1.70485
H	-1.75881	-2.48613	-0.19395	H	1.52618	-2.39897	0.26862
H	-3.01368	-0.34123	-1.97007	H	2.74902	-0.22163	2.02807
H	-3.6894	-1.91163	-1.59843	H	3.31625	-1.86981	1.86784
H	-3.67402	3.02793	1.98864	H	4.18424	2.73945	-2.05429
H	-1.94354	2.74333	2.10263	H	2.46105	2.5772	-2.35697
H	-2.55708	3.98048	0.98855	H	3.044	3.85413	-1.27181
H	-4.84136	-2.40309	0.45905	H	4.66796	-2.62703	0.0167
H	-3.36675	-2.16649	1.40708	H	3.34019	-2.35541	-1.11991
H	-4.83636	-1.2831	1.83093	H	4.92332	-1.63391	-1.42713
H	1.35798	0.5847	-1.01844	H	-1.36925	0.93398	0.5483
H	1.34327	-1.91616	1.90732	H	-1.44178	-2.65776	-0.84272
H	1.26639	-2.96479	0.51066	H	0.19617	-2.45765	-1.49991
H	-0.22591	-2.55998	1.38179	H	-1.20177	-1.69283	-2.28066
H	4.14332	-0.04792	-1.91649	H	-3.55494	1.37403	1.07099
H	3.51778	1.35477	-1.06083	H	-4.03476	1.71937	-0.58491
H	5.29734	-1.3385	0.2648	H	-5.2901	-0.70068	-1.13541
H	7.25468	1.47479	-0.59843	H	-6.40044	-1.0652	1.69263
H	5.86321	2.40032	-1.19648	H	-4.96981	-0.34168	2.45594
H	6.40294	0.94639	-2.06361	H	-4.77781	-1.55961	1.181
H	6.16232	1.24448	1.66065	H	-7.31721	1.19375	1.05089
H	4.72205	2.13471	1.12569	H	-5.93394	1.98839	1.82935
H	4.5573	0.50956	1.81691	H	-6.31844	2.33674	0.12993
atom	Con f. 4- 8c			atom	Con f. 4- 8d		
C	5.56421	-0.04648	1.93795	C	5.36947	-2.03214	1.0016
C	2.96261	2.33232	0.83212	C	3.95211	1.34884	1.52115
C	3.09909	1.86288	-0.41299	C	3.81186	1.4513	0.19455
C	3.14692	0.39434	-0.81943	C	3.19664	0.40293	-0.72498
C	3.81036	-0.62502	0.17045	C	3.42734	-1.10879	-0.37929
C	5.11151	-0.04533	0.68405	C	4.88142	-1.32014	-0.01424
C	1.73245	-0.07221	-1.24813	C	1.69221	0.71699	-0.92404
C	0.8277	-0.36497	-0.03674	C	0.84164	0.26689	0.28447
C	1.46582	-1.46395	0.83581	C	0.99711	-1.24455	0.46915
C	2.85927	-1.04198	1.31372	C	2.46359	-1.61599	0.71597
C	3.19713	2.81598	-1.57952	C	4.25026	2.70438	-0.52337
C	4.2034	-1.88582	-0.64128	C	3.19658	-1.93033	-1.67335
C	-0.574	-0.72452	-0.46667	C	-0.56786	0.78774	0.13625
C	-1.60043	0.06946	-0.06795	C	-1.61023	-0.04995	-0.09424
C	-0.75264	-1.9519	-1.31639	C	-0.71142	2.28088	0.25902
C	-3.0122	-0.1182	-0.38288	C	-3.0049	0.34234	-0.26579
C	-3.97506	0.9206	0.16863	C	-4.00737	-0.78553	-0.44951
O	-3.43656	-1.05314	-1.08723	O	-3.38679	1.52685	-0.29084
C	-5.39981	0.39843	0.44812	C	-5.42602	-0.47886	0.07456
O	-5.97571	-0.10764	-0.77166	O	-5.9579	0.66762	-0.61753
C	-5.38175	-0.70887	1.51001	C	-6.3595	-1.64157	-0.25105
C	-6.29466	1.55218	0.89318	C	-5.40721	-0.20897	1.58491
H	3.76507	0.36592	-1.72632	H	3.67203	0.55758	-1.70231
H	0.77011	0.54906	0.56479	H	1.25816	0.76716	1.17142
H	6.54022	0.36614	2.18038	H	6.44145	-2.14761	1.13995
H	4.99069	-0.44699	2.76886	H	4.7336	-2.52101	1.73416
H	2.91513	3.40322	1.01642	H	4.38013	2.16663	2.09644
H	2.91376	1.69486	1.70536	H	3.67506	0.46509	2.08083
H	5.74696	0.37336	-0.0986	H	5.58294	-0.85925	-0.7121

H	1.81906	-0.96947	-1.86779	H	1.31846	0.20591	-1.81779
H	1.27041	0.69647	-1.87606	H	1.5717	1.78881	-1.10472
H	0.82868	-1.66252	1.70488	H	0.39101	-1.58891	1.31466
H	1.5262	-2.399	0.26866	H	0.62304	-1.76552	-0.41974
H	2.74902	-0.22164	2.02808	H	2.76297	-1.22261	1.69151
H	3.31627	-1.8698	1.86785	H	2.55444	-2.70583	0.7877
H	4.18434	2.73947	-2.05419	H	3.44201	3.1167	-1.13997
H	2.46119	2.57711	-2.35708	H	4.57753	3.48041	0.17408
H	3.04394	3.8541	-1.27188	H	5.0801	2.48355	-1.20764
H	4.66795	-2.62703	0.0167	H	3.34245	-2.99657	-1.47339
H	3.34022	-2.35537	-1.11994	H	2.18785	-1.79768	-2.0726
H	4.92337	-1.63388	-1.42709	H	3.9051	-1.63342	-2.45397
H	-1.36925	0.93393	0.54835	H	-1.42498	-1.11655	-0.14657
H	-1.44191	-2.65768	-0.84289	H	0.23087	2.73811	0.57013
H	0.19616	-2.45772	-1.49982	H	-1.48982	2.54126	0.98159
H	-1.20157	-1.69274	-2.28077	H	-1.01775	2.72896	-0.69244
H	-3.55492	1.37394	1.07107	H	-4.06623	-0.97905	-1.53031
H	-4.03474	1.71943	-0.58479	H	-3.62463	-1.70062	0.01158
H	-5.29011	-0.70064	-1.13544	H	-5.24864	1.33663	-0.55994
H	-6.40044	-1.0653	1.69255	H	-7.37773	-1.40788	0.0769
H	-4.96984	-0.34179	2.45593	H	-6.03614	-2.55651	0.25411
H	-4.77779	-1.55965	1.18094	H	-6.37942	-1.82791	-1.32973
H	-7.31719	1.19371	1.05098	H	-5.02343	-1.07454	2.13512
H	-5.93391	1.98834	1.82943	H	-4.77748	0.65374	1.82147
H	-6.31845	2.33672	0.13003	H	-6.42154	0.00242	1.93777
atom	Con f. 4- 8e			atom	Con f. 4- 8f		
C	-5.49332	-1.87996	-0.85887	C	5.69888	0.1381	1.67441
C	-4.10825	1.53785	-1.11777	C	2.82101	2.33506	0.88265
C	-3.78524	1.46055	0.17819	C	2.91016	1.91804	-0.38522
C	-3.05785	0.3021	0.84979	C	3.02605	0.47043	-0.84906
C	-3.35873	-1.15143	0.34253	C	3.84844	-0.52544	0.04039
C	-4.8531	-1.31867	0.16697	C	5.14124	0.13923	0.46348
C	-1.53625	0.59625	0.86494	C	1.61586	-0.08637	-1.17051
C	-0.87682	0.3111	-0.50247	C	0.84748	-0.48805	0.10203
C	-1.0793	-1.16371	-0.85737	C	1.64113	-1.56343	0.87002
C	-2.57154	-1.50293	-0.93904	C	3.03586	-1.05079	1.24375
C	-4.10642	2.59963	1.11461	C	2.87025	2.91304	-1.51949
C	-2.9537	-2.13732	1.46773	C	4.26225	-1.72399	-0.85142
C	0.54657	0.814	-0.49364	C	-0.55683	-0.93839	-0.22176
C	1.59878	-0.0423	-0.46329	C	-1.59914	-0.26265	0.32625
C	0.69207	2.31159	-0.49954	C	-0.71825	-2.11257	-1.14644
C	3.00802	0.33409	-0.43411	C	-3.01596	-0.54303	0.12228
C	4.01067	-0.80879	-0.43601	C	-3.99811	0.26603	0.95359
O	3.40239	1.51472	-0.44001	O	-3.43327	-1.41158	-0.66659
C	5.37116	-0.48418	0.21352	C	-5.36266	0.51753	0.27732
O	5.98078	0.62632	-0.47293	O	-5.99179	-0.74301	-0.02247
C	5.20363	-0.14477	1.7007	C	-6.29587	1.23731	1.24709
C	6.32298	-1.66424	0.03778	C	-5.19405	1.33078	-1.01277
H	-3.38218	0.32149	1.89829	H	3.56127	0.51537	-1.80641
H	-1.40931	0.92049	-1.24747	H	0.77719	0.39701	0.74418
H	-6.57568	-1.98043	-0.85556	H	6.6577	0.61958	1.84893
H	-4.97702	-2.25435	-1.73816	H	5.23146	-0.33064	2.5356
H	-4.60333	2.42192	-1.51306	H	2.71504	3.39345	1.10989
H	-3.91794	0.73988	-1.82379	H	2.86599	1.66654	1.73271
H	-5.4396	-0.96611	1.0174	H	5.67288	0.62929	-0.35435
H	-1.04226	-0.0229	1.6215	H	1.71115	-0.95447	-1.82892
H	-1.37607	1.63681	1.16153	H	1.04593	0.66642	-1.725
H	-0.60238	-1.39237	-1.81711	H	1.09962	-1.84075	1.78134
H	-0.59363	-1.79595	-0.10537	H	1.72117	-2.47093	0.2622
H	-3.00207	-0.98197	-1.79911	H	2.92907	-0.26442	1.99596
H	-2.68986	-2.57348	-1.14176	H	3.60004	-1.85769	1.72543
H	-3.21589	2.92968	1.66378	H	3.82698	2.9185	-2.05837
H	-4.51875	3.46152	0.58257	H	2.10188	2.64903	-2.25666
H	-4.83587	2.28016	1.87056	H	2.67	3.92819	-1.16566
H	-3.14113	-3.16816	1.15074	H	4.82369	-2.4557	-0.2618
H	-1.89584	-2.05259	1.72899	H	3.39876	-2.23327	-1.28714
H	-3.53719	-1.95214	2.37587	H	4.90218	-1.39195	-1.67589
H	1.40851	-1.10947	-0.46174	H	-1.37701	0.56816	0.98994
H	1.23675	2.66565	0.38109	H	0.23663	-2.59759	-1.35417
H	-0.28562	2.79832	-0.52988	H	-1.15254	-1.79098	-2.09992
H	1.27287	2.63913	-1.36776	H	-1.41089	-2.84965	-0.73192
H	3.56612	-1.68943	0.03737	H	-4.16501	-0.30495	1.8783
H	4.17557	-1.07273	-1.49081	H	-3.54276	1.2153	1.24973
H	5.27737	1.30285	-0.51048	H	-5.2926	-1.26451	-0.46276
H	6.17884	0.08556	2.14108	H	-7.28041	1.37321	0.78744

H	4.76655	-0.98529	2.25004	H	-5.9011	2.22245	1.51297
H	4.55504	0.72602	1.83462	H	-6.42168	0.65349	2.1646
H	7.30253	-1.42178	0.46293	H	-6.1711	1.49838	-1.47688
H	5.93955	-2.55565	0.54305	H	-4.737	2.30474	-0.80864
H	6.45628	-1.89607	-1.0238	H	-4.56261	0.80013	-1.7317
atom	Con f. 4- 8g			atom	Con f. 4- 8h		
C	-5.49325	-1.88032	-0.85877	C	-5.40702	-1.96925	-1.00979
C	-4.10847	1.53782	-1.11795	C	-4.0027	1.44184	-1.37196
C	-3.78541	1.4606	0.178	C	-3.81995	1.455	-0.04662
C	-3.05794	0.30222	0.84965	C	-3.16267	0.35397	0.77689
C	-3.35874	-1.15138	0.34253	C	-3.41036	-1.13468	0.3499
C	-4.8531	-1.31872	0.16694	C	-4.87803	-1.32147	0.02831
C	-1.53633	0.59642	0.86483	C	-1.65077	0.66136	0.9237
C	-0.87677	0.31107	-0.50246	C	-0.85791	0.28132	-0.34601
C	-1.07922	-1.1638	-0.85716	C	-1.01639	-1.21929	-0.60183
C	-2.57145	-1.50302	-0.93894	C	-2.49193	-1.57768	-0.81041
C	-4.10686	2.5996	1.11443	C	-4.24528	2.65011	0.77096
C	-2.9537	-2.13711	1.46786	C	-3.13221	-2.03181	1.58282
C	0.54662	0.81397	-0.49358	C	0.55379	0.80485	-0.23817
C	1.59884	-0.04233	-0.4631	C	1.60173	-0.02834	-0.01653
C	0.69209	2.31155	-0.49951	C	0.69011	2.2965	-0.38128
C	3.00808	0.33403	-0.43401	C	2.99701	0.3722	0.12992
C	4.01076	-0.80881	-0.43607	C	3.98229	-0.72955	0.48558
O	3.40247	1.51467	-0.43978	O	3.38861	1.54742	0.00688
C	5.37127	-0.48412	0.21346	C	5.43186	-0.48274	0.01956
O	5.9808	0.6264	-0.47301	O	5.92204	0.74258	0.59759
C	5.20376	-0.1447	1.70063	C	6.34034	-1.5913	0.54433
C	6.32314	-1.66415	0.03773	C	5.51068	-0.40164	-1.51074
H	-3.38229	0.32166	1.89814	H	-3.59173	0.44622	1.78302
H	-1.40921	0.92032	-1.2476	H	-1.32011	0.82304	-1.18413
H	-6.5756	-1.98084	-0.85548	H	-6.48399	-2.07349	-1.11415
H	-4.9769	-2.25493	-1.73794	H	-4.80161	-2.4142	-1.79427
H	-4.60372	2.4218	-1.51324	H	-4.45733	2.29203	-1.87542
H	-3.91811	0.73984	-1.82394	H	-3.73666	0.60129	-1.9999
H	-5.43965	-0.96597	1.01725	H	-5.55115	-0.9022	0.77844
H	-1.04238	-0.02259	1.62153	H	-1.23453	0.10336	1.76938
H	-1.37619	1.63703	1.16127	H	-1.52247	1.72248	1.15673
H	-0.60219	-1.39262	-1.81681	H	-0.44256	-1.5169	-1.48671
H	-0.59365	-1.79594	-0.105	H	-0.60965	-1.78569	0.24365
H	-3.00189	-0.98213	-1.7991	H	-2.82957	-1.12842	-1.74868
H	-2.68978	-2.57358	-1.14155	H	-2.58805	-2.66154	-0.94122
H	-4.8362	2.27997	1.87041	H	-3.42089	3.02542	1.38984
H	-3.21639	2.92989	1.66356	H	-4.60096	3.47028	0.14111
H	-4.51941	3.46139	0.58238	H	-5.05111	2.3731	1.46344
H	-3.14112	-3.16799	1.15102	H	-3.28492	-3.08459	1.32491
H	-1.89583	-2.05234	1.72909	H	-2.10943	-1.92048	1.95185
H	-3.53716	-1.9518	2.37599	H	-3.8114	-1.78403	2.40549
H	1.40855	-1.10949	-0.46152	H	1.41631	-1.09257	0.07351
H	-0.28562	2.79824	-0.52983	H	-0.28792	2.76764	-0.50392
H	1.2729	2.63911	-1.36771	H	1.30486	2.54221	-1.25378
H	1.23673	2.66563	0.38114	H	1.19603	2.74077	0.4808
H	3.56632	-1.68956	0.03721	H	3.97197	-0.80994	1.58231
H	4.17573	-1.07258	-1.49089	H	3.619	-1.68745	0.1017
H	5.27731	1.30285	-0.51066	H	5.22211	1.39683	0.40726
H	6.17898	0.08559	2.14102	H	7.37636	-1.399	0.24649
H	4.76663	-0.98519	2.24997	H	6.04205	-2.56493	0.1443
H	4.55522	0.72613	1.83456	H	6.29914	-1.63663	1.63736
H	7.30269	-1.42164	0.46284	H	6.54557	-0.22509	-1.82046
H	5.93976	-2.55557	0.54303	H	5.166	-1.33253	-1.97333
H	6.45642	-1.896	-1.02385	H	4.89595	0.41862	-1.89312
atom	Con f. 4- 8i			atom	Con f. 4- 8j		
C	5.82884	-1.298	-0.22421	C	-5.98038	-0.82611	0.28183
C	3.97379	1.44674	1.42298	C	-3.70998	1.13258	-1.89362
C	3.41837	1.73646	0.24094	C	-3.19597	1.68979	-0.7914
C	2.78789	0.7381	-0.72383	C	-2.74671	0.94922	0.46368
C	3.41537	-0.69597	-0.8151	C	-3.54864	-0.32824	0.89383
C	4.92305	-0.57655	-0.88474	C	-5.03174	-0.04128	0.79315
C	1.25812	0.67141	-0.47907	C	-1.22547	0.66107	0.38019
C	0.9092	-0.16143	0.75532	C	-0.91532	-0.51974	-0.54077
C	1.43407	-1.60436	0.56018	C	-1.6185	-1.78664	0.00284
C	2.94871	-1.61618	0.33305	C	-3.13377	-1.58123	0.09306
C	3.34966	3.16623	-0.23801	C	-2.98616	3.18274	-0.71952
C	2.98226	-1.3194	-2.16679	C	-3.27046	-0.57827	2.3982
C	-0.55098	-0.19813	1.1398	C	0.54739	-0.81029	-0.7794
C	-1.52031	0.20078	0.27752	C	1.51336	-0.2875	0.01743

C	-0.83523	-0.73325	2.51696	C	0.83302	-1.72666	-1.93836
C	-2.95655	0.17272	0.5313	C	2.94769	-0.52597	-0.1053
C	-3.85049	0.74801	-0.55531	C	3.83604	0.178	0.90805
O	-3.45859	-0.26969	1.58131	O	3.44724	-1.27977	-0.96066
C	-5.24398	0.09164	-0.65584	C	5.2745	0.45915	0.42783
O	-5.94222	0.25019	0.5938	O	5.91843	-0.78206	0.08091
C	-6.08278	0.8159	-1.70522	C	5.27544	1.39969	-0.78466
C	-5.1243	-1.39969	-0.99677	C	6.09372	1.0556	1.56905
H	2.91922	1.17792	-1.72092	H	-2.87835	1.66595	1.28459
H	1.44373	0.26258	1.6175	H	-1.35102	-0.30813	-1.52763
H	6.89253	-1.147	-0.38968	H	-7.02514	-0.52674	0.30345
H	5.55768	-2.05625	0.50483	H	-5.76405	-1.78523	-0.17973
H	4.37751	2.23635	2.05268	H	-3.97794	1.74703	-2.75016
H	4.06935	0.43765	1.80219	H	-3.90126	0.07161	-1.98864
H	5.27338	0.1664	-1.60368	H	-5.32818	0.9082	1.24271
H	0.77626	0.23958	-1.36192	H	-0.84699	0.45252	1.38589
H	0.86352	1.6873	-0.37417	H	-0.70566	1.55961	0.03204
H	1.1988	-2.22101	1.43342	H	-1.41173	-2.64724	-0.64129
H	0.90764	-2.05015	-0.29221	H	-1.19604	-2.02041	0.98722
H	3.44013	-1.3309	1.26744	H	-3.53555	-1.52888	-0.92273
H	3.27258	-2.64124	0.11877	H	-3.58987	-2.46414	0.55567
H	2.32483	3.45016	-0.50728	H	-3.63125	3.62359	0.05201
H	3.71058	3.86782	0.51923	H	-1.95488	3.42918	-0.43829
H	3.95533	3.29636	-1.1446	H	-3.20697	3.67232	-1.67213
H	3.40008	-2.32617	-2.2669	H	-3.81487	-1.46481	2.73845
H	1.89585	-1.39627	-2.25782	H	-2.20841	-0.73772	2.60102
H	3.34533	-0.71798	-3.00699	H	-3.601	0.27346	3.00216
H	-1.23445	0.58268	-0.69544	H	1.22704	0.3736	0.82724
H	-1.39377	-1.67407	2.46941	H	-0.08528	-1.97336	-2.4763
H	-1.46131	-0.03803	3.08363	H	1.30482	-2.65638	-1.60399
H	0.09227	-0.90596	3.06783	H	1.53705	-1.2637	-2.63629
H	-3.98063	1.81417	-0.32045	H	3.35749	1.10653	1.23359
H	-3.34006	0.69972	-1.5214	H	3.87897	-0.47666	1.79059
H	-5.2962	-0.02961	1.27116	H	5.28067	-1.22884	-0.50843
H	-6.17681	1.87786	-1.45636	H	6.30387	1.58512	-1.11025
H	-7.08723	0.3818	-1.74616	H	4.81274	2.3611	-0.53827
H	-5.62952	0.72956	-2.69722	H	4.72673	0.96033	-1.62287
H	-6.12109	-1.84682	-1.06435	H	7.12734	1.21493	1.24464
H	-4.61473	-1.54544	-1.95504	H	5.68057	2.01739	1.88731
H	-4.56262	-1.9353	-0.22555	H	6.10351	0.37905	2.42973
atom	Con f. 4- 8k			atom	Con f. 4- 8l		
C	5.96469	-0.87083	-0.3311	C	-5.7609	0.11371	-1.54868
C	3.74793	1.1211	1.8726	C	-2.8324	2.30223	-0.97467
C	3.22367	1.68159	0.77688	C	-2.86385	1.94202	0.31317
C	2.74958	0.94373	-0.47065	C	-2.98141	0.51805	0.84561
C	3.53051	-0.34428	-0.90847	C	-3.85108	-0.50403	0.0339
C	5.01809	-0.07443	-0.82811	C	-5.15354	0.15784	-0.36273
C	1.22625	0.67321	-0.3672	C	-1.56756	-0.04679	1.13612
C	0.9163	-0.50055	0.56295	C	-0.85561	-0.51254	-0.14821
C	1.59572	-1.7776	0.01294	C	-1.69246	-1.60535	-0.84098
C	3.11195	-1.59116	-0.09983	C	-3.0959	-1.09139	-1.17868
C	3.02764	3.17643	0.70561	C	-2.74911	2.98497	1.39838
C	3.22982	-0.59404	-2.40857	C	-4.24111	-1.65877	0.9919
C	-0.54576	-0.77294	0.82494	C	0.55349	-0.96901	0.14435
C	-1.51494	-0.27697	0.01459	C	1.58794	-0.27385	-0.3937
C	-0.82641	-1.6433	2.01969	C	0.73101	-2.16712	1.0356
C	-2.9485	-0.50739	0.15671	C	3.00711	-0.55445	-0.20826
C	-3.84019	0.08769	-0.92111	C	3.98123	0.37472	-0.91432
O	-3.44591	-1.17596	1.08173	O	3.43129	-1.51226	0.4648
C	-5.27211	0.43311	-0.46251	C	5.34808	0.53091	-0.21557
O	-5.92166	-0.75609	0.02629	O	5.98635	-0.7564	-0.11025
C	-5.25422	1.50258	0.63741	C	6.27114	1.39274	-1.07268
C	-6.09753	0.90597	-1.65616	C	5.18339	1.14307	1.18166
H	2.87925	1.65711	-1.29481	H	-3.47767	0.61359	1.82025
H	1.37	-0.29016	1.54166	H	-0.79766	0.34707	-0.82519
H	7.01246	-0.58365	-0.36729	H	-6.72135	0.59865	-1.70349
H	5.74379	-1.82783	0.13271	H	-5.33414	-0.395	-2.40829
H	4.03385	1.73429	2.72423	H	-2.72096	3.34746	-1.25404
H	3.92973	0.05845	1.9671	H	-2.93126	1.59774	-1.79022
H	5.31924	0.87182	-1.2813	H	-5.64584	0.68714	0.45525
H	0.83382	0.46487	-1.3675	H	-1.64807	-0.88423	1.8352
H	0.71957	1.57816	-0.01634	H	-0.96469	0.71944	1.63417
H	1.38698	-2.63391	0.66233	H	-1.19182	-1.92668	-1.76121
H	1.15613	-2.00828	-0.96461	H	-1.75881	-2.48613	-0.19395
H	3.52977	-1.54292	0.90966	H	-3.01368	-0.34123	-1.97007

H	3.54998	-2.48014	-0.56822	H	-3.6894	-1.91163	-1.59843
H	1.99515	3.43274	0.43806	H	-3.67402	3.02793	1.98864
H	3.26612	3.66498	1.65447	H	-1.94354	2.74333	2.10263
H	3.66663	3.60988	-0.07514	H	-2.55708	3.98048	0.98855
H	3.76106	-1.48642	-2.75432	H	-4.84136	-2.40309	0.45905
H	2.1637	-0.7435	-2.59723	H	-3.36675	-2.16649	1.40708
H	3.56062	0.25339	-3.01843	H	-4.83636	-1.2831	1.83093
H	-1.23012	0.34333	-0.82731	H	1.35798	0.5847	-1.01844
H	-1.3867	-2.54084	1.74086	H	1.34327	-1.91616	1.90732
H	-1.44918	-1.11031	2.74565	H	1.26639	-2.96479	0.51066
H	0.10202	-1.94235	2.51171	H	-0.22591	-2.55998	1.38179
H	-3.35925	0.96913	-1.35508	H	4.14332	-0.04792	-1.91649
H	-3.89873	-0.66281	-1.72284	H	3.51778	1.35477	-1.06083
H	-5.2781	-1.14406	0.65038	H	5.29734	-1.3385	0.2648
H	-4.70145	1.15475	1.51511	H	7.25468	1.47479	-0.59843
H	-6.27798	1.73264	0.94895	H	5.86321	2.40032	-1.19648
H	-4.78574	2.42632	0.28176	H	6.40294	0.94639	-2.06361
H	-7.12625	1.11163	-1.34244	H	6.16232	1.24448	1.66065
H	-5.67875	1.82146	-2.08471	H	4.72205	2.13471	1.12569
H	-6.12214	0.13699	-2.43498	H	4.5573	0.50956	1.81691
atom	Con f. 4- 8m			atom	Con f. 4- 8n		
C	5.56421	-0.0465	1.93795	C	5.56419	-0.04675	1.93808
C	2.96266	2.3323	0.83215	C	2.96262	2.33215	0.83212
C	3.09913	1.86289	-0.41297	C	3.09926	1.86275	-0.41298
C	3.14694	0.39435	-0.81944	C	3.1471	0.39421	-0.81941
C	3.81037	-0.62503	0.17044	C	3.81034	-0.62516	0.17057
C	5.11152	-0.04536	0.68404	C	5.11156	-0.04564	0.68416
C	1.73247	-0.07218	-1.24816	C	1.73262	-0.07226	-1.24821
C	0.82771	-0.36494	-0.03679	C	0.82771	-0.36471	-0.03687
C	1.46582	-1.46392	0.83578	C	1.46563	-1.46362	0.83596
C	2.85926	-1.04197	1.31371	C	2.85912	-1.0418	1.31386
C	3.1971	2.816	-1.5795	C	3.19746	2.81587	-1.57948
C	4.20339	-1.88584	-0.64128	C	4.2032	-1.88611	-0.64104
C	-0.574	-0.7245	-0.46668	C	-0.57398	-0.72421	-0.46685
C	-1.60044	0.06947	-0.06795	C	-1.60046	0.06957	-0.0678
C	-0.75265	-1.95192	-1.31634	C	-0.75252	-1.95127	-1.31703
C	-3.0122	-0.11823	-0.38284	C	-3.01222	-0.11788	-0.38287
C	-3.97507	0.92061	0.16858	C	-3.97509	0.92049	0.16938
O	-3.43657	-1.05324	-1.08711	O	-3.43654	-1.05211	-1.08821
C	-5.39982	0.39845	0.4481	C	-5.3999	0.39815	0.44831
O	-5.97571	-0.10769	-0.77165	O	-5.97566	-0.10705	-0.77189
C	-5.38176	-0.7088	1.51005	C	-5.38199	-0.70992	1.50941
C	-6.29467	1.55222	0.8931	C	-6.29474	1.55162	0.89406
H	3.7651	0.36593	-1.72632	H	3.76535	0.36574	-1.72624
H	0.77011	0.54909	0.56474	H	0.77016	0.5494	0.56454
H	6.54023	0.36611	2.18037	H	6.54026	0.36572	2.18052
H	4.99068	-0.44699	2.76885	H	4.99056	-0.4471	2.76899
H	2.91519	3.40319	1.01648	H	2.91507	3.40303	1.01645
H	2.91384	1.69481	1.70537	H	2.91364	1.69464	1.70532
H	5.74698	0.37331	-0.0986	H	5.74709	0.37287	-0.0985
H	1.81908	-0.96943	-1.86783	H	1.81918	-0.96964	-1.8677
H	1.27045	0.69651	-1.87609	H	1.27072	0.69634	-1.87631
H	0.82865	-1.66247	1.70485	H	0.82838	-1.66187	1.70504
H	1.52618	-2.39897	0.26862	H	1.52585	-2.39881	0.26902
H	2.74902	-0.22163	2.02807	H	2.74897	-0.22134	2.02809
H	3.31625	-1.86981	1.86784	H	3.31597	-1.86962	1.86813
H	4.18424	2.73945	-2.05429	H	4.18477	2.7394	-2.05395
H	2.46105	2.5772	-2.35697	H	2.46169	2.57705	-2.35721
H	3.044	3.85413	-1.27181	H	3.0442	3.85398	-1.27183
H	4.66796	-2.62703	0.0167	H	4.66756	-2.62734	0.01703
H	3.34019	-2.35541	-1.11991	H	3.33996	-2.35555	-1.11973
H	4.92332	-1.63391	-1.42713	H	4.92326	-1.63437	-1.42683
H	-1.36925	0.93398	0.5483	H	-1.36926	0.93377	0.54889
H	-1.44178	-2.65776	-0.84272	H	-1.44362	-2.65628	-0.84513
H	0.19617	-2.45765	-1.49991	H	0.19602	-2.4581	-1.49893
H	-1.20177	-1.69283	-2.28066	H	-1.19934	-1.69128	-2.28223
H	-3.55494	1.37403	1.07099	H	-3.55502	1.37304	1.07225
H	-4.03476	1.71937	-0.58491	H	-4.03465	1.71994	-0.58339
H	-5.2901	-0.70068	-1.13541	H	-5.2899	-0.6996	-1.13614
H	-6.40044	-1.0652	1.69263	H	-6.40069	-1.06655	1.69144
H	-4.96981	-0.34168	2.45594	H	-4.97032	-0.34354	2.4557
H	-4.77781	-1.55961	1.181	H	-4.7779	-1.56042	1.17982
H	-7.31721	1.19375	1.05089	H	-7.31731	1.19309	1.05149
H	-5.93394	1.98839	1.82935	H	-5.93407	1.98709	1.83066
H	-6.31844	2.33674	0.12993	H	-6.31841	2.33671	0.13147

atom	Con f. 4- 8o			atom	Con f. 4- 8p		
C	5.36947	-2.03214	1.0016	C	-5.49332	-1.87996	-0.85887
C	3.95211	1.34884	1.52115	C	-4.10825	1.53785	-1.11777
C	3.81186	1.4513	0.19455	C	-3.78524	1.46055	0.17819
C	3.19664	0.40293	-0.72498	C	-3.05785	0.3021	0.84979
C	3.42734	-1.10879	-0.37929	C	-3.35873	-1.15143	0.34253
C	4.88142	-1.32014	-0.01424	C	-4.8531	-1.31867	0.16697
C	1.69221	0.71699	-0.92404	C	-1.53625	0.59625	0.86494
C	0.84164	0.26689	0.28447	C	-0.87682	0.3111	-0.50247
C	0.99711	-1.24455	0.46915	C	-1.0793	-1.16371	-0.85737
C	2.46359	-1.61599	0.71597	C	-2.57154	-1.50293	-0.93904
C	4.25026	2.70438	-0.52337	C	-4.10642	2.59963	1.11461
C	3.19658	-1.93033	-1.67335	C	-2.9537	-2.13732	1.46773
C	-0.56786	0.78774	0.13625	C	0.54657	0.814	-0.49364
C	-1.61023	-0.04995	-0.09424	C	1.59878	-0.0423	-0.46329
C	-0.71142	2.28088	0.25902	C	0.69207	2.31159	-0.49954
C	-3.0049	0.34234	-0.26579	C	3.00802	0.33409	-0.43411
C	-4.00737	-0.78553	-0.44951	C	4.01067	-0.80879	-0.43601
O	-3.38679	1.52685	-0.29084	O	3.40239	1.51472	-0.44001
C	-5.42602	-0.47886	0.07456	C	5.37116	-0.48418	0.21352
O	-5.9579	0.66762	-0.61753	O	5.98078	0.62632	-0.47293
C	-6.3595	-1.64157	-0.25105	C	5.20363	-0.14477	1.7007
C	-5.40721	-0.20897	1.58491	C	6.32298	-1.66424	0.03778
H	3.67203	0.55758	-1.70231	H	-3.38218	0.32149	1.89829
H	1.25816	0.76716	1.17142	H	-1.40931	0.92049	-1.24747
H	6.44145	-2.14761	1.13995	H	-6.57568	-1.98043	-0.85556
H	4.7336	-2.52101	1.73416	H	-4.97702	-2.25435	-1.73816
H	4.38013	2.16663	2.09644	H	-4.60333	2.42192	-1.51306
H	3.67506	0.46509	2.08083	H	-3.91794	0.73988	-1.82379
H	5.58294	-0.85925	-0.7121	H	-5.4396	-0.96611	1.0174
H	1.31846	0.20591	-1.81779	H	-1.04226	-0.0229	1.6215
H	1.5717	1.78881	-1.10472	H	-1.37607	1.63681	1.16153
H	0.39101	-1.58891	1.31466	H	-0.60238	-1.39237	-1.81711
H	0.62304	-1.76552	-0.41974	H	-0.59363	-1.79595	-0.10537
H	2.76297	-1.22261	1.69151	H	-3.00207	-0.98197	-1.79911
H	2.55444	-2.70583	0.7877	H	-2.68986	-2.57348	-1.14176
H	3.44201	3.1167	-1.13997	H	-3.21589	2.92968	1.66378
H	4.57753	3.48041	0.17408	H	-4.51875	3.46152	0.58257
H	5.0801	2.48355	-1.20764	H	-4.83587	2.28016	1.87056
H	3.34245	-2.99657	-1.47339	H	-3.14113	-3.16816	1.15074
H	2.18785	-1.79768	-2.0726	H	-1.89584	-2.05259	1.72899
H	3.9051	-1.63342	-2.45397	H	-3.53719	-1.95214	2.37587
H	-1.42498	-1.11655	-0.14657	H	1.40851	-1.10947	-0.46174
H	0.23087	2.73811	0.57013	H	1.23675	2.66565	0.38109
H	-1.48982	2.54126	0.98159	H	-0.28562	2.79832	-0.52988
H	-1.01775	2.72896	-0.69244	H	1.27287	2.63913	-1.36776
H	-4.06623	-0.97905	-1.53031	H	3.56612	-1.68943	0.03737
H	-3.62463	-1.70062	0.01158	H	4.17557	-1.07273	-1.49081
H	-5.24864	1.33663	-0.55994	H	5.27737	1.30285	-0.51048
H	-7.37773	-1.40788	0.0769	H	6.17884	0.08556	2.14108
H	-6.03614	-2.55651	0.25411	H	4.76655	-0.98529	2.25004
H	-6.37942	-1.82791	-1.32973	H	4.55504	0.72602	1.83462
H	-5.02343	-1.07454	2.13512	H	7.30253	-1.42178	0.46293
H	-4.77748	0.65374	1.82147	H	5.93955	-2.55565	0.54305
H	-6.42154	0.00242	1.93777	H	6.45628	-1.89607	-1.0238

atom	Con f. 4- 8q			atom	Con f. 4- 8r		
C	5.69888	0.1381	1.67441	C	-5.49325	-1.88032	-0.85877
C	2.82101	2.33506	0.88265	C	-4.10847	1.53782	-1.11795
C	2.91016	1.91804	-0.38522	C	-3.78541	1.4606	0.178
C	3.02605	0.47043	-0.84906	C	-3.05794	0.30222	0.84965
C	3.84844	-0.52544	0.04039	C	-3.35874	-1.15138	0.34253
C	5.14124	0.13923	0.46348	C	-4.8531	-1.31872	0.16694
C	1.61586	-0.08637	-1.17051	C	-1.53633	0.59642	0.86483
C	0.84748	-0.48805	0.10203	C	-0.87677	0.31107	-0.50246
C	1.64113	-1.56343	0.87002	C	-1.07922	-1.1638	-0.85716
C	3.03586	-1.05079	1.24375	C	-2.57145	-1.50302	-0.93894
C	2.87025	2.91304	-1.51949	C	-4.10686	2.5996	1.11443
C	4.26225	-1.72399	-0.85142	C	-2.9537	-2.13711	1.46786
C	-0.55683	-0.93839	-0.22176	C	0.54662	0.81397	-0.49358
C	-1.59914	-0.26265	0.32625	C	1.59884	-0.04233	-0.4631
C	-0.71825	-2.11257	-1.14644	C	0.69209	2.31155	-0.49951
C	-3.01596	-0.54303	0.12228	C	3.00808	0.33403	-0.43401
C	-3.99811	0.26603	0.95359	C	4.01076	-0.80881	-0.43607
O	-3.43327	-1.41158	-0.66659	O	3.40247	1.51467	-0.43978
C	-5.36266	0.51753	0.27732	C	5.37127	-0.48412	0.21346

O	-5.99179	-0.74301	-0.02247	O	5.9808	0.6264	-0.47301
C	-6.29587	1.23731	1.24709	C	5.20376	-0.1447	1.70063
C	-5.19405	1.33078	-1.01277	C	6.32314	-1.66415	0.03773
H	3.56127	0.51537	-1.80641	H	-3.38229	0.32166	1.89814
H	0.77719	0.39701	0.74418	H	-1.40921	0.92032	-1.2476
H	6.6577	0.61958	1.84893	H	-6.5756	-1.98084	-0.85548
H	5.23146	-0.33064	2.5356	H	-4.9769	-2.25493	-1.73794
H	2.71504	3.39345	1.10989	H	-4.60372	2.4218	-1.51324
H	2.86599	1.66654	1.73271	H	-3.91811	0.73984	-1.82394
H	5.67288	0.62929	-0.35435	H	-5.43965	-0.96597	1.01725
H	1.71115	-0.95447	-1.82892	H	-1.04238	-0.02259	1.62153
H	1.04593	0.66642	-1.725	H	-1.37619	1.63703	1.16127
H	1.09962	-1.84075	1.78134	H	-0.60219	-1.39262	-1.81681
H	1.72117	-2.47093	0.2622	H	-0.59365	-1.79594	-0.105
H	2.92907	-0.26442	1.99596	H	-3.00189	-0.98213	-1.7991
H	3.60004	-1.85769	1.72543	H	-2.68978	-2.57358	-1.14155
H	3.82698	2.9185	-2.05837	H	-4.8362	2.27997	1.87041
H	2.10188	2.64903	-2.25666	H	-3.21639	2.92989	1.66356
H	2.67	3.92819	-1.16566	H	-4.51941	3.46139	0.58238
H	4.82369	-2.4557	-0.2618	H	-3.14112	-3.16799	1.15102
H	3.39876	-2.23327	-1.28714	H	-1.89583	-2.05234	1.72909
H	4.90218	-1.39195	-1.67589	H	-3.53716	-1.9518	2.37599
H	-1.37701	0.56816	0.98994	H	1.40855	-1.10949	-0.46152
H	0.23663	-2.59759	-1.35417	H	-0.28562	2.79824	-0.52983
H	-1.15254	-1.79098	-2.09992	H	1.2729	2.63911	-1.36771
H	-1.41089	-2.84965	-0.73192	H	1.23673	2.66563	0.38114
H	-4.16501	-0.30495	1.8783	H	3.56632	-1.68956	0.03721
H	-3.54276	1.2153	1.24973	H	4.17573	-1.07258	-1.49089
H	-5.2926	-1.26451	-0.46276	H	5.27731	1.30285	-0.51066
H	-7.28041	1.37321	0.78744	H	6.17898	0.08559	2.14102
H	-5.9011	2.22245	1.51297	H	4.76663	-0.98519	2.24997
H	-6.42168	0.65349	2.1646	H	4.55522	0.72613	1.83456
H	-6.1711	1.49838	-1.47688	H	7.30269	-1.42164	0.46284
H	-4.737	2.30474	-0.80864	H	5.93976	-2.55557	0.54303
H	-4.56261	0.80013	-1.7317	H	6.45642	-1.896	-1.02385
atom		Con f. 4- 8s		atom		Con f. 4- 8t	
C	-5.40702	-1.96925	-1.00979	C	5.82955	-1.29631	-0.22713
C	-4.0027	1.44184	-1.37196	C	3.97422	1.44358	1.4264
C	-3.81995	1.455	-0.04662	C	3.41834	1.73612	0.24524
C	-3.16267	0.35397	0.77689	C	2.7881	0.73997	-0.72198
C	-3.41036	-1.13468	0.3499	C	3.41589	-0.69376	-0.81673
C	-4.87803	-1.32147	0.02831	C	4.92354	-0.57377	-0.88617
C	-1.65077	0.66136	0.9237	C	1.25828	0.67236	-0.47744
C	-0.85791	0.28132	-0.34601	C	0.90941	-0.16369	0.75479
C	-1.01639	-1.21929	-0.60183	C	1.43481	-1.60597	0.5563
C	-2.49193	-1.57768	-0.81041	C	2.94947	-1.61676	0.32927
C	-4.24528	2.65011	0.77096	C	3.34896	3.16708	-0.22999
C	-3.13221	-2.03181	1.58282	C	2.98298	-1.31425	-2.16991
C	0.55379	0.80485	-0.23817	C	-0.55087	-0.20189	1.13886
C	1.60173	-0.02834	-0.01653	C	-1.52017	0.19845	0.2772
C	0.69011	2.2965	-0.38128	C	-0.8353	-0.74016	2.51476
C	2.99701	0.3722	0.12992	C	-2.95642	0.16988	0.5308
C	3.98229	-0.72955	0.48558	C	-3.85002	0.74775	-0.5547
O	3.38861	1.54742	0.00688	O	-3.45856	-0.27458	1.5799
C	5.43186	-0.48274	0.01956	C	-5.24472	0.09388	-0.65523
O	5.92204	0.74258	0.59759	O	-5.94211	0.25252	0.5949
C	6.34034	-1.5913	0.54433	C	-6.08275	0.82068	-1.70352
C	5.51068	-0.40164	-1.51074	C	-5.12806	-1.39735	-0.99756
H	-3.59173	0.44622	1.78302	H	2.91937	1.1823	-1.71797
H	-1.32011	0.82304	-1.18413	H	1.44359	0.25838	1.61813
H	-6.48399	-2.07349	-1.11415	H	6.8932	-1.1447	-0.39233
H	-4.80161	-2.4142	-1.79427	H	5.55862	-2.05603	0.50046
H	-4.45733	2.29203	-1.87542	H	4.37785	2.23172	2.05801
H	-3.73666	0.60129	-1.9999	H	4.07045	0.43355	1.80293
H	-5.55115	-0.9022	0.77844	H	5.27361	0.17075	-1.60362
H	-1.23453	0.10336	1.76938	H	0.77651	0.24285	-1.36147
H	-1.52247	1.72248	1.15674	H	0.86354	1.6879	-0.36981
H	-0.44256	-1.5169	-1.48671	H	1.19972	-2.22475	1.42808
H	-0.60965	-1.78569	0.24365	H	0.90864	-2.04998	-0.29716
H	-2.82957	-1.12842	-1.74868	H	3.44072	-1.33348	1.26437
H	-2.58805	-2.66154	-0.94122	H	3.27373	-2.64122	0.11261
H	-3.42089	3.02542	1.38984	H	2.3239	3.45133	-0.49805
H	-4.60096	3.47028	0.14111	H	3.70998	3.86683	0.52891
H	-5.05111	2.3731	1.46344	H	3.95414	3.29977	-1.13653
H	-3.28492	-3.08459	1.32491	H	3.40163	-2.32041	-2.27253

H	-2.10943	-1.92048	1.95185	H	1.89663	-1.39193	-2.2609
H	-3.8114	-1.78403	2.40549	H	3.34536	-0.71051	-3.00874
H	1.41631	-1.09257	0.07351	H	-1.23424	0.58255	-0.69488
H	-0.28792	2.76764	-0.50392	H	-1.39171	-1.68213	2.46481
H	1.30486	2.54221	-1.25378	H	-1.4634	-0.04739	3.08215
H	1.19603	2.74077	0.4808	H	0.09204	-0.91209	3.06615
H	3.97197	-0.80994	1.58231	H	-3.97814	1.81395	-0.31896
H	3.619	-1.68745	0.1017	H	-3.34015	0.69926	-1.5211
H	5.22211	1.39683	0.40726	H	-5.29664	-0.03002	1.27161
H	7.37636	-1.399	0.24649	H	-6.17484	1.88256	-1.45352
H	6.04205	-2.56493	0.1443	H	-7.08797	0.38837	-1.74449
H	6.29914	-1.63663	1.63736	H	-5.63006	0.73461	-2.69581
H	6.54557	-0.22509	-1.82046	H	-6.12576	-1.84251	-1.06492
H	5.166	-1.33253	-1.97333	H	-4.61941	-1.5433	-1.95629
H	4.89595	0.41862	-1.89312	H	-4.56693	-1.93466	-0.22713

atom	Con f. 5- 1a			atom	Con f. 5- 1b		
C	-5.27723	-0.96449	-0.52992	C	5.52074	-0.01825	-1.1444
C	-1.52942	-2.14648	1.74207	C	2.8583	1.99836	1.91584
C	-1.74701	-1.92455	0.43969	C	2.72002	1.84677	0.59322
C	-1.69272	-0.53622	-0.18259	C	2.11548	0.5999	-0.03803
C	-3.05944	0.24732	-0.13299	C	3.16328	-0.54704	-0.30406
C	-4.08824	-0.51709	-0.937	C	4.19519	-0.0452	-1.2911
C	-0.5428	0.31255	0.38176	C	0.89099	0.08017	0.73822
C	-0.37049	1.63913	-0.37825	C	0.183	-1.05572	-0.00653
C	-1.68134	2.43709	-0.32024	C	1.1775	-2.20806	-0.25483
C	-2.84761	1.6052	-0.86308	C	2.41375	-1.71198	-1.00882
C	-3.5328	0.49123	1.3041	C	3.81761	-1.04744	0.98851
C	-2.01757	-3.06656	-0.50526	C	3.13303	2.93989	-0.35854
C	0.84552	2.39563	0.13051	C	-1.08013	-1.5632	0.66383
C	2.06918	2.38427	-0.75928	C	-2.09482	-2.20917	-0.25704
C	2.60557	0.98802	-1.11001	C	-2.90235	-1.15667	-1.04159
C	3.19428	0.23186	0.05268	C	-3.82127	-0.35894	-0.14664
C	3.58814	-1.24782	-0.12217	C	-3.95652	1.15502	-0.34221
C	0.85371	3.02673	1.30905	C	-1.32155	-1.44927	1.97501
O	3.39766	0.75981	1.14144	O	-4.46625	-0.89397	0.75006
C	4.50023	-1.42494	-1.34106	C	-4.34441	1.48655	-1.78698
O	4.3145	-1.64476	1.03998	O	-4.9894	1.62591	0.52013
C	2.32729	-2.12129	-0.23931	C	-2.62615	1.81808	0.05742
H	-1.48061	-0.67943	-1.25181	H	1.75425	0.89374	-1.03379
H	-0.19396	1.3897	-1.43392	H	-0.10376	-0.66923	-0.99606
H	-5.939	-1.49061	-1.21327	H	6.16286	0.35047	-1.94029
H	-5.63943	-0.83207	0.48525	H	6.01879	-0.35511	-0.24002
H	-1.56627	-3.15208	2.15395	H	3.28933	2.9042	2.33546
H	-1.3102	-1.34559	2.4409	H	2.54985	1.23685	2.62512
H	-3.79778	-0.6918	-1.97447	H	3.77132	0.31032	-2.23196
H	-0.70426	0.53642	1.44077	H	1.196	-0.26489	1.73066
H	0.37882	-0.27012	0.33131	H	0.19562	0.91052	0.89829
H	-1.88346	2.74313	0.71199	H	1.4607	-2.64443	0.71052
H	-1.58494	3.35864	-0.90631	H	0.69987	-3.00765	-0.83199
H	-2.66649	1.401	-1.92687	H	2.10045	-1.37166	-2.00486
H	-3.7802	2.17926	-0.80959	H	3.11814	-2.53715	-1.16735
H	-3.73773	-0.44828	1.82266	H	4.40456	-0.26128	1.46866
H	-2.78769	1.0357	1.88771	H	3.07506	-1.39357	1.71013
H	-4.45096	1.08749	1.30513	H	4.48477	-1.88846	0.77399
H	-2.97714	-2.93813	-1.01719	H	3.5468	3.8033	0.17088
H	-1.24784	-3.10899	-1.28711	H	3.88365	2.5826	-1.07174
H	-2.03146	-4.02873	0.01517	H	2.27354	3.27887	-0.95185
H	1.8151	2.87489	-1.70827	H	-1.5911	-2.85743	-0.98199
H	2.87018	2.97012	-0.29864	H	-2.78834	-2.83122	0.31475
H	1.84483	0.36577	-1.59243	H	-2.24317	-0.49457	-1.60576
H	3.4076	1.08417	-1.85382	H	-3.54948	-1.65607	-1.77557
H	1.74375	3.53359	1.67289	H	-2.23172	-1.84873	2.41405
H	-0.02071	3.0597	1.95365	H	-0.63048	-0.96182	2.65561
H	5.37322	-0.76884	-1.26936	H	-5.27892	0.98531	-2.05682
H	4.84863	-2.46169	-1.37296	H	-4.49341	2.56717	-1.87382
H	3.97221	-1.212	-2.27381	H	-3.56674	1.19085	-2.49512
H	4.19294	-0.91311	1.67494	H	-5.19654	0.8699	1.102
H	1.70536	-2.02182	0.65458	H	-1.80326	1.51352	-0.59353
H	2.638	-3.16704	-0.32653	H	-2.74227	2.90356	-0.01699
H	1.72869	-1.86715	-1.11803	H	-2.37147	1.56396	1.09025

atom	Con f. 5- 1c			atom	Con f. 5- 1d		
C	5.65395	-0.10418	1.80238	C	-5.75141	-0.00633	-0.69289

C	3.11608	2.62943	-0.73069	C	-2.1087	2.67478	-0.5967
C	3.6685	1.44257	-1.00955	C	-2.68749	1.94397	0.36387
C	3.00201	0.11691	-0.66537	C	-2.38262	0.46699	0.57893
C	3.32149	-0.39022	0.79268	C	-3.27302	-0.49667	-0.29333
C	4.81134	-0.6275	0.91041	C	-4.72281	-0.31188	0.09964
C	1.48741	0.13058	-0.93983	C	-0.8844	0.14977	0.42636
C	0.85223	-1.25768	-0.74905	C	-0.55759	-1.3104	0.79743
C	1.13816	-1.77392	0.67402	C	-1.40764	-2.27238	-0.0493
C	2.64078	-1.77739	0.96403	C	-2.89812	-1.9554	0.09145
C	2.81843	0.57997	1.86764	C	-3.06332	-0.27489	-1.7955
C	4.99253	1.35435	-1.72407	C	-3.66804	2.56717	1.3239
C	-0.63053	-1.28459	-1.07976	C	0.93083	-1.58333	0.70018
C	-1.49377	-0.24793	-0.39585	C	1.52048	-1.68078	-0.69641
C	-2.99816	-0.42909	-0.57011	C	2.97997	-1.24206	-0.8126
C	-3.80667	0.63001	0.12961	C	3.17649	0.22317	-0.51093
C	-5.30788	0.4013	0.36726	C	4.56098	0.73926	-0.09433
C	-1.11426	-2.20078	-1.92681	C	1.66845	-1.72098	1.80781
O	-3.30817	1.6772	0.53202	O	2.25731	1.03214	-0.59467
C	-5.46316	-0.62246	1.50661	C	5.64818	0.26442	-1.0617
O	-5.88452	1.64195	0.77222	O	4.5288	2.16512	-0.12516
C	-6.02015	-0.06086	-0.90552	C	4.843	0.27422	1.34588
H	3.43719	-0.63898	-1.33417	H	-2.6476	0.2374	1.62071
H	1.35175	-1.9455	-1.44482	H	-0.84981	-1.44944	1.84714
H	6.70831	-0.36866	1.79617	H	-6.75836	0.07995	-0.29252
H	5.33834	0.6012	2.56532	H	-5.63948	0.17061	-1.75848
H	3.61737	3.55774	-0.99415	H	-2.34587	3.72889	-0.71936
H	2.15481	2.72455	-0.23574	H	-1.38337	2.26032	-1.2895
H	5.2067	-1.33018	0.17488	H	-4.91757	-0.47546	1.16097
H	0.99513	0.85441	-0.28444	H	-0.55436	0.35121	-0.59612
H	1.31794	0.4751	-1.96641	H	-0.31257	0.8236	1.07313
H	0.61438	-1.15476	1.41072	H	-1.11082	-2.2073	-1.10157
H	0.74204	-2.79051	0.78036	H	-1.22224	-3.30631	0.2657
H	3.1282	-2.48892	0.28428	H	-3.19835	-2.1267	1.1337
H	2.82942	-2.13726	1.98241	H	-3.49136	-2.64314	-0.52273
H	3.33027	1.54304	1.80758	H	-2.01343	-0.37619	-2.07793
H	1.74724	0.76907	1.77333	H	-3.39112	0.72139	-2.10068
H	2.99224	0.1633	2.86505	H	-3.63224	-1.012	-2.37145
H	5.40494	2.34508	-1.93719	H	-3.3125	2.46646	2.35777
H	5.72617	0.79715	-1.13186	H	-3.81972	3.63031	1.11474
H	4.88217	0.81539	-2.67429	H	-4.6409	2.06631	1.27744
H	-1.2588	-0.23238	0.67544	H	1.43666	-2.71569	-1.05101
H	-1.2127	0.74688	-0.76084	H	0.92234	-1.08144	-1.38782
H	-3.27749	-0.37407	-1.6316	H	3.64828	-1.84544	-0.1941
H	-3.32671	-1.41798	-0.23435	H	3.32933	-1.38574	-1.845
H	-2.16797	-2.27817	-2.17571	H	2.73911	-1.89727	1.78447
H	-0.45353	-2.91864	-2.40685	H	1.21227	-1.66334	2.79305
H	-4.94549	-0.27181	2.40498	H	6.60348	0.71055	-0.76871
H	-6.52731	-0.72836	1.73803	H	5.75829	-0.82249	-1.04164
H	-5.06865	-1.60484	1.23388	H	5.41802	0.57974	-2.08388
H	-5.12737	2.20088	1.02974	H	3.58144	2.39547	-0.16846
H	-5.66237	-1.03966	-1.23385	H	4.94	-0.81216	1.41534
H	-7.09283	-0.13859	-0.70368	H	5.78184	0.72749	1.67811
H	-5.87192	0.66113	-1.71426	H	4.04233	0.60044	2.01664

atom	Con f. 5- 1e			atom	Con f. 5- 1f		
C	5.25167	2.18196	-0.2422	C	-4.85028	-1.0755	-1.74108
C	3.92066	-1.88856	-1.54009	C	-3.46985	-0.20273	2.46822
C	4.05	-1.37703	-0.31001	C	-2.92707	-0.96124	1.50822
C	2.95913	-0.55664	0.36557	C	-2.01719	-0.39919	0.42387
C	3.00943	0.97731	0.00889	C	-2.79385	0.1445	-0.83353
C	4.33015	1.54427	0.48136	C	-3.54492	-1.0009	-1.47676
C	1.55406	-1.14222	0.13495	C	-1.01822	0.63512	0.97208
C	0.47623	-0.40636	0.95038	C	0.00089	1.08506	-0.09386
C	0.50249	1.10161	0.63415	C	-0.7357	1.63499	-1.31882
C	1.90256	1.68386	0.84011	C	-1.73064	0.61019	-1.86676
C	2.78785	1.23184	-1.48664	C	-3.73037	1.3048	-0.47869
C	5.29678	-1.61779	0.50166	C	-3.16735	-2.4483	1.46752
C	-0.90584	-0.99907	0.75577	C	1.04902	2.01396	0.49087
C	-1.39137	-1.14395	-0.67589	C	1.877	1.4751	1.6438
C	-2.90788	-1.09709	-0.85595	C	2.49712	0.09279	1.39459
C	-3.50972	0.22862	-0.46207	C	3.44522	0.05309	0.224
C	-5.00895	0.31876	-0.14371	C	3.76055	-1.28774	-0.45593
C	-1.64204	-1.36615	1.81074	C	1.26664	3.25369	0.0385
O	-2.83868	1.25403	-0.39459	O	3.98565	1.06644	-0.20868

C	-5.26754	-0.37563	1.20489	C	2.52833	-1.73566	-1.2622
O	-5.35434	1.69803	-0.02962	O	4.85184	-1.08186	-1.35118
C	-5.85117	-0.29806	-1.26404	C	4.17395	-2.34526	0.57109
H	3.14908	-0.60671	1.44694	H	-1.41616	-1.2395	0.0482
H	0.73522	-0.51634	2.01218	H	0.52667	0.1775	-0.4252
H	6.16039	2.5598	0.21945	H	-5.2721	-1.95114	-2.22787
H	5.14645	2.3529	-1.30944	H	-5.54736	-0.28246	-1.48713
H	4.71806	-2.47364	-1.99203	H	-4.11271	-0.63645	3.23054
H	3.02616	-1.74901	-2.13887	H	-3.29776	0.86695	2.53409
H	4.5109	1.41213	1.54952	H	-2.90982	-1.84096	-1.76358
H	1.30288	-1.10312	-0.92903	H	-1.53949	1.51861	1.35614
H	1.56361	-2.20201	0.41406	H	-0.50116	0.18564	1.82507
H	0.16847	1.27728	-0.39386	H	-1.25614	2.56297	-1.05545
H	-0.21113	1.62082	1.28385	H	-0.0153	1.8925	-2.10363
H	2.1651	1.60567	1.90348	H	-1.17387	-0.27102	-2.21277
H	1.90698	2.75253	0.59448	H	-2.24791	1.01731	-2.74353
H	2.75335	2.3076	-1.68726	H	-4.19431	1.70848	-1.38449
H	3.5895	0.79922	-2.08954	H	-4.52592	0.9823	0.19665
H	1.84576	0.80263	-1.8332	H	-3.19534	2.12212	0.00894
H	5.76933	-0.67316	0.79145	H	-3.61822	-2.75033	0.51618
H	5.05375	-2.14534	1.4335	H	-2.21851	-2.99438	1.55202
H	6.03047	-2.21375	-0.04923	H	-3.82527	-2.77498	2.2783
H	-0.94235	-0.36114	-1.29338	H	2.67074	2.18966	1.8778
H	-1.02175	-2.09237	-1.0856	H	1.25258	1.39341	2.54154
H	-3.15814	-1.23823	-1.917	H	3.08943	-0.20345	2.2719
H	-3.41616	-1.90871	-0.32947	H	1.73628	-0.68406	1.28364
H	-2.64178	-1.77791	1.71658	H	2.04257	3.87873	0.47364
H	-1.25617	-1.26654	2.82233	H	0.69005	3.69399	-0.76796
H	-6.31683	-0.22542	1.47632	H	2.78322	-2.64918	-1.80808
H	-5.07313	-1.44986	1.15628	H	1.66993	-1.94583	-0.61931
H	-4.63972	0.06236	1.98672	H	2.24763	-0.96579	-1.98746
H	-4.50037	2.16972	0.00271	H	4.93139	-0.11222	-1.43172
H	-5.66307	-1.36959	-1.36615	H	3.35511	-2.58452	1.25377
H	-6.91084	-0.15682	-1.03039	H	4.45872	-3.26078	0.04358
H	-5.63893	0.19246	-2.21887	H	5.03326	-2.00046	1.15418

atom	Con f. 5- 1g			atom	Con f. 5- 1h		
C	4.94491	-0.81203	-1.35194	C	5.84745	0.01059	0.54177
C	1.56016	-2.50132	0.90634	C	2.16407	2.65314	0.70235
C	2.34067	-1.46816	1.24541	C	2.69166	1.93449	-0.29598
C	2.10474	-0.04855	0.74794	C	2.39462	0.45428	-0.49699
C	2.70926	0.23176	-0.67943	C	3.351	-0.49944	0.3135
C	4.20632	0.02224	-0.61865	C	4.7686	-0.30166	-0.17804
C	0.62576	0.37257	0.82819	C	0.91338	0.11487	-0.25537
C	0.43935	1.8675	0.49505	C	0.5817	-1.35058	-0.60992
C	1.01856	2.17698	-0.89345	C	1.48697	-2.28956	0.19342
C	2.48153	1.73778	-0.98808	C	2.9632	-1.96193	-0.04409
C	2.06132	-0.63329	-1.76676	C	3.24235	-0.27822	1.82651
C	3.50965	-1.6531	2.1781	C	3.59982	2.57402	-1.31466
C	-0.9988	2.32929	0.69259	C	-0.9079	-1.6001	-0.45949
C	-1.97567	2.28866	-0.46513	C	-1.74332	-1.10877	-1.62783
C	-2.34657	0.89561	-0.99521	C	-3.19207	-0.75297	-1.30065
C	-3.16223	0.06387	-0.04015	C	-3.31549	0.41665	-0.35553
C	-3.39957	-1.42635	-0.34075	C	-4.61433	0.6212	0.43796
C	-1.38716	2.79115	1.88684	C	-1.4453	-2.19158	0.61294
O	-3.67076	0.53717	0.97132	O	-2.40831	1.22854	-0.20121
C	-2.08601	-2.19732	-0.13489	C	-4.69394	-0.45566	1.53457
O	-4.37031	-1.91073	0.58572	O	-4.55851	1.90748	1.0526
C	-3.94778	-1.62293	-1.75744	C	-5.83886	0.57928	-0.4799
H	2.65451	0.61911	1.42599	H	2.59636	0.23232	-1.55454
H	1.04649	2.41837	1.22704	H	0.82648	-1.48678	-1.675
H	6.02302	-0.86347	-1.22284	H	6.82319	0.10549	0.07203
H	4.5169	-1.47167	-2.10083	H	5.80938	0.18532	1.61294
H	1.75784	-3.50171	1.28409	H	2.39214	3.71042	0.81444
H	0.70613	-2.39472	0.24736	H	1.49045	2.22406	1.43747
H	4.71147	0.65314	0.11471	H	4.89108	-0.46227	-1.25067
H	0.0197	-0.24272	0.16041	H	0.63824	0.28404	0.79075
H	0.25832	0.18304	1.84245	H	0.29821	0.79795	-0.84809
H	0.43005	1.67544	-1.66911	H	1.25923	-2.20585	1.26154
H	0.9484	3.25235	-1.09632	H	1.29014	-3.331	-0.08688
H	3.0753	2.33019	-0.2793	H	3.19764	-2.13352	-1.10308
H	2.87751	1.9557	-1.987	H	3.59969	-2.64353	0.53233
H	2.45111	-0.35801	-2.75222	H	3.85614	-1.00965	2.36227
H	2.26263	-1.6947	-1.60638	H	3.58107	0.72125	2.10815

H	0.97725	-0.50379	-1.79461	H	2.21496	-0.38945	2.17874
H	3.39235	-1.02865	3.07352	H	4.57793	2.08171	-1.33848
H	3.61497	-2.6946	2.4962	H	3.17621	2.47727	-2.32302
H	4.44644	-1.34329	1.70151	H	3.75582	3.63707	-1.10824
H	-2.89547	2.8008	-0.1687	H	-1.25763	-0.23404	-2.0713
H	-1.55999	2.85375	-1.30691	H	-1.73686	-1.87861	-2.41087
H	-1.47132	0.32677	-1.32012	H	-3.71683	-0.45427	-2.21915
H	-2.9639	1.01039	-1.89694	H	-3.75289	-1.60688	-0.91259
H	-2.41025	3.10616	2.0746	H	-2.51537	-2.34029	0.7142
H	-0.69235	2.86436	2.72089	H	-0.83663	-2.55806	1.43303
H	-2.2851	-3.26613	-0.25899	H	-5.56903	-0.24997	2.15842
H	-1.32321	-1.90246	-0.8595	H	-4.79552	-1.46012	1.11624
H	-1.69891	-2.03183	0.8744	H	-3.80104	-0.42453	2.16621
H	-4.46604	-1.19834	1.24648	H	-3.6286	2.19	0.96179
H	-3.2199	-1.32491	-2.51598	H	-5.96239	-0.40351	-0.94117
H	-4.18053	-2.68223	-1.90286	H	-6.73366	0.79512	0.11186
H	-4.86553	-1.04401	-1.89894	H	-5.75495	1.33379	-1.26793

atom	Con f. 5- 1i			atom	Con f. 5- 1j		
C	6.24377	-0.06306	-0.54174	C	-4.85452	-0.55631	-1.73519
C	3.05188	2.2674	1.68393	C	-1.6398	-3.11516	-0.35235
C	3.17697	1.96329	0.3865	C	-1.54599	-2.05372	0.45844
C	2.69513	0.64494	-0.20489	C	-1.66168	-0.64478	-0.10898
C	3.76107	-0.51224	-0.12246	C	-3.07775	0.03909	0.00907
C	4.972	-0.11895	-0.94039	C	-4.10173	-0.84552	-0.67247
C	1.33395	0.20529	0.36363	C	-0.5542	0.26613	0.44384
C	0.77796	-1.04111	-0.35568	C	-0.49998	1.61996	-0.28271
C	1.79144	-2.18573	-0.25169	C	-1.86096	2.31588	-0.14085
C	3.14959	-1.76154	-0.81727	C	-2.98109	1.41941	-0.68019
C	4.14205	-0.84653	1.3242	C	-3.54093	0.21165	1.47095
C	3.7869	2.94243	-0.58334	C	-1.24294	-2.23371	1.92577
C	-0.62089	-1.36395	0.14298	C	0.69077	2.43432	0.19307
C	-1.70366	-0.53321	-0.5093	C	1.89062	2.47584	-0.7293
C	-3.09564	-0.65134	0.10112	C	2.46005	1.10799	-1.13687
C	-4.12249	0.2063	-0.58836	C	3.09704	0.32998	-0.01463
C	-5.47848	0.4568	0.09167	C	3.54832	-1.12443	-0.25511
C	-0.86604	-2.28366	1.08299	C	0.70243	3.07063	1.36867
O	-3.91592	0.72902	-1.67979	O	3.29064	0.81998	1.09357
C	-5.26395	1.45306	1.24552	C	4.41765	-1.22775	-1.51198
O	-6.35318	1.04123	-0.87256	O	4.33743	-1.52507	0.8644
C	-6.11496	-0.84449	0.58385	C	2.31913	-2.04557	-0.35024
H	2.54079	0.8176	-1.27932	H	-1.49204	-0.73478	-1.18955
H	0.68733	-0.78059	-1.42134	H	-0.35719	1.4129	-1.35268
H	7.0342	0.21517	-1.23419	H	-5.56571	-1.28019	-2.12564
H	6.55076	-0.28635	0.47567	H	-4.80299	0.39436	-2.2577
H	3.40254	3.21986	2.07416	H	-1.54226	-4.13129	0.02271
H	2.60058	1.58857	2.40067	H	-1.82442	-3.00258	-1.41786
H	4.74469	0.11937	-1.98088	H	-4.22058	-1.82453	-0.20995
H	1.40986	-0.01651	1.43339	H	-0.69792	0.45067	1.51349
H	0.63333	1.04145	0.26881	H	0.40298	-0.24995	0.35155
H	1.90673	-2.49092	0.79392	H	-2.04519	2.56276	0.91072
H	1.42291	-3.06329	-0.79552	H	-1.85553	3.26535	-0.6889
H	3.0336	-1.5423	-1.88709	H	-2.80848	1.26359	-1.75266
H	3.86566	-2.58848	-0.7433	H	-3.94597	1.93195	-0.58902
H	4.63831	-0.00372	1.81056	H	-3.77065	-0.75248	1.93237
H	3.26672	-1.10422	1.92371	H	-2.79245	0.71221	2.09006
H	4.82293	-1.70352	1.34907	H	-4.45573	0.81374	1.49912
H	4.09516	3.86685	-0.08597	H	-1.96813	-1.72429	2.56675
H	4.66233	2.51237	-1.0817	H	-1.23451	-3.29369	2.19462
H	3.0703	3.19851	-1.37468	H	-0.26093	-1.8162	2.17726
H	-1.39704	0.52041	-0.49822	H	1.59626	2.98344	-1.6577
H	-1.75	-0.80159	-1.57286	H	2.68652	3.07418	-0.27584
H	-3.46509	-1.68418	0.03774	H	1.70795	0.47883	-1.62419
H	-3.08616	-0.41278	1.16995	H	3.24066	1.25457	-1.89508
H	-1.86743	-2.48267	1.45111	H	1.57977	3.6159	1.70725
H	-0.07189	-2.8772	1.52447	H	-0.15562	3.07097	2.03557
H	-4.80083	2.37216	0.87294	H	5.2617	-0.53339	-1.45766
H	-6.23849	1.70487	1.6744	H	4.81136	-2.24599	-1.58538
H	-4.63462	1.03729	2.03667	H	3.84265	-1.01724	-2.41697
H	-5.76914	1.34718	-1.5918	H	4.18025	-0.83993	1.54163
H	-5.51965	-1.30967	1.3732	H	1.66501	-1.78029	-1.18455
H	-7.10737	-0.62351	0.98861	H	2.66583	-3.07307	-0.49785
H	-6.22767	-1.55433	-0.24116	H	1.74412	-2.00846	0.57866

atom	Con f. 5- 1k			atom	Con f. 5- 1l		
C	5.93856	-1.7975	-0.22913	C	-4.64157	-1.52849	-0.89332
C	4.42009	2.41193	0.45091	C	-1.42276	-1.80586	2.17344
C	4.20056	1.54768	-0.5472	C	-1.3187	-1.76611	0.8391
C	3.1177	0.47797	-0.49166	C	-1.32601	-0.46713	0.0462
C	3.5925	-0.86479	0.18468	C	-2.76398	0.09268	-0.26866
C	4.71633	-1.45485	-0.63918	C	-3.48266	-0.91536	-1.1386
C	1.80825	0.99462	0.13245	C	-0.43188	0.60955	0.67691
C	0.67463	-0.03151	0.0163	C	-0.27308	1.82275	-0.25765
C	1.10148	-1.35022	0.69255	C	-1.6586	2.43305	-0.50331
C	2.41204	-1.8733	0.10159	C	-2.59366	1.385	-1.12003
C	4.011	-0.66323	1.64543	C	-3.55715	0.40804	1.00418
C	5.0087	1.61091	-1.8177	C	-1.11788	-3.02246	0.03338
C	-0.66786	0.44697	0.5448	C	0.78879	2.77541	0.26092
C	-1.85487	-0.17678	-0.15368	C	2.20747	2.55953	-0.23074
C	-3.22562	0.20027	0.39673	C	2.88975	1.2535	0.22415
C	-4.36223	-0.48437	-0.31373	C	2.56192	0.01862	-0.57994
C	-5.78297	0.08553	-0.18385	C	3.06136	-1.36259	-0.10705
C	-0.78265	1.32058	1.55147	C	0.51383	3.76464	1.11882
O	-4.19719	-1.48433	-1.00637	O	1.98266	0.07028	-1.66105
C	-5.8652	1.36954	-1.02941	C	2.70219	-1.68259	1.34488
O	-6.6956	-0.87747	-0.7079	O	2.44696	-2.3521	-0.93526
C	-6.15271	0.3495	1.27744	C	4.58677	-1.40395	-0.31954
H	2.88569	0.20878	-1.53183	H	-0.88082	-0.69598	-0.93199
H	0.54116	-0.25126	-1.05375	H	0.08135	1.44048	-1.22137
H	6.65603	-2.23449	-0.91903	H	-5.06176	-2.2334	-1.60625
H	6.27645	-1.6591	0.79363	H	-5.21555	-1.3604	0.0133
H	5.19799	3.16827	0.37854	H	-1.40023	-2.75045	2.71163
H	3.84103	2.40156	1.3689	H	-1.53284	-0.90889	2.77457
H	4.45429	-1.62713	-1.68452	H	-2.96616	-1.13907	-2.07367
H	1.97119	1.24618	1.18422	H	-0.83501	0.95334	1.63551
H	1.51952	1.92509	-0.36841	H	0.5364	0.16263	0.90575
H	1.20371	-1.17644	1.77035	H	-2.08051	2.80973	0.43513
H	0.32069	-2.10948	0.57054	H	-1.57804	3.29241	-1.17926
H	2.24292	-2.1241	-0.95406	H	-2.19543	1.10232	-2.10359
H	2.70885	-2.80298	0.60123	H	-3.58567	1.8171	-1.29747
H	3.20832	-0.22269	2.24028	H	-4.52749	0.84464	0.74616
H	4.88003	-0.00619	1.7256	H	-3.73591	-0.4909	1.59787
H	4.26844	-1.6245	2.1017	H	-3.03221	1.12527	1.6389
H	5.52244	0.6626	-2.00822	H	-1.16224	-3.91949	0.65837
H	4.35558	1.79247	-2.68139	H	-1.87213	-3.11218	-0.75638
H	5.75918	2.40622	-1.78123	H	-0.13816	-2.99543	-0.46105
H	-1.80796	0.09305	-1.21793	H	2.22007	2.58762	-1.32733
H	-1.74514	-1.26799	-0.13441	H	2.82831	3.38988	0.11772
H	-3.3095	-0.08451	1.45534	H	3.97878	1.37307	0.1389
H	-3.38517	1.2826	0.37332	H	2.70437	1.06039	1.28484
H	-1.74582	1.63551	1.93998	H	1.29432	4.42309	1.49267
H	0.08728	1.75663	2.03228	H	-0.49301	3.95549	1.47922
H	-6.89597	1.73593	-1.00806	H	1.62154	-1.64282	1.50556
H	-5.20999	2.15674	-0.64713	H	3.04357	-2.6974	1.571
H	-5.59323	1.1578	-2.06827	H	3.19209	-0.99758	2.04055
H	-6.14091	-1.50407	-1.20965	H	2.12604	-1.86523	-1.71773
H	-5.51985	1.12335	1.71852	H	5.10674	-0.70029	0.33617
H	-7.19195	0.68869	1.32534	H	4.9422	-2.41438	-0.09683
H	-6.05925	-0.56593	1.86942	H	4.83549	-1.16835	-1.35913

atom	Con f. 5- 1m			atom	Con f. 5- 1n		
C	5.29161	-2.20576	0.1424	C	-4.54706	-0.52258	2.41724
C	4.0666	1.91381	1.52349	C	-3.1253	2.92757	0.31684
C	4.12688	1.38472	0.29542	C	-2.74585	1.95077	-0.51618
C	2.99281	0.56785	-0.31011	C	-2.14609	0.66061	0.03057
C	3.05692	-0.96458	0.05329	C	-3.17781	-0.51328	0.25373
C	4.33383	-1.54714	-0.51217	C	-4.25724	-0.04153	1.20722
C	1.60706	1.16494	-0.00152	C	-0.93658	0.20772	-0.8093
C	0.48814	0.44092	-0.75787	C	-0.2071	-0.9844	-0.18522
C	0.51232	-1.05994	-0.4	C	-1.2023	-2.14835	-0.0045
C	1.88548	-1.67073	-0.68481	C	-2.40037	-1.7113	0.83987
C	2.94739	-1.20787	1.5632	C	-3.89237	-0.93466	-1.04818
C	5.33332	1.60029	-0.58162	C	-2.85354	2.13468	-2.01071
C	-0.9004	1.01722	-0.5525	C	1.0326	-1.43039	-0.93862
C	-1.86512	0.72445	-1.6876	C	2.04005	-2.23445	-0.14234
C	-3.34681	0.72293	-1.31769	C	2.85921	-1.34976	0.81866
C	-3.71168	-0.36667	-0.33986	C	3.74267	-0.37091	0.08321
C	-4.99748	-0.2444	0.49126	C	3.86367	1.07379	0.5796

C	-1.25157	1.71284	0.53448	C	1.26516	-1.13333	-2.22248
O	-3.01621	-1.36676	-0.19027	O	4.37152	-0.70164	-0.91772
C	-6.20562	0.04481	-0.40434	C	4.30666	1.10815	2.0465
O	-5.21658	-1.48957	1.15221	O	4.8532	1.73375	-0.2065
C	-4.80225	0.85507	1.54946	C	2.51029	1.77678	0.37544
H	3.1196	0.61066	-1.40114	H	-1.77299	0.89223	1.03645
H	0.71321	0.51699	-1.83329	H	0.11187	-0.68402	0.82394
H	6.15993	-2.59539	-0.38286	H	-5.36133	-0.10096	3.00155
H	5.25666	-2.3828	1.21326	H	-4.00205	-1.34176	2.87643
H	4.89211	2.49613	1.9258	H	-3.55072	3.85847	-0.05098
H	3.20297	1.79041	2.16912	H	-3.02465	2.82857	1.39477
H	4.44309	-1.41064	-1.5894	H	-4.85924	0.78125	0.82484
H	1.4144	1.11606	1.07431	H	-1.26468	-0.0549	-1.81987
H	1.6102	2.22727	-0.26917	H	-0.24481	1.05056	-0.91624
H	0.25056	-1.17127	0.65882	H	-1.52628	-2.49357	-0.99376
H	-0.25218	-1.60211	-0.96553	H	-0.7134	-3.00027	0.48023
H	2.0735	-1.62084	-1.76558	H	-2.03297	-1.44034	1.83779
H	1.89102	-2.73345	-0.41499	H	-3.08964	-2.55278	0.97638
H	2.92434	-2.28206	1.77358	H	-4.49661	-1.82979	-0.86445
H	3.79367	-0.77446	2.10088	H	-4.56756	-0.15086	-1.40197
H	2.03531	-0.77374	1.97777	H	-3.19128	-1.1646	-1.85423
H	5.04865	2.11224	-1.51029	H	-3.37262	3.06661	-2.25195
H	6.09751	2.20045	-0.0788	H	-1.86019	2.17543	-2.47358
H	5.78462	0.64673	-0.87603	H	-3.38637	1.31163	-2.4956
H	-1.70142	1.46234	-2.48434	H	1.5341	-2.99855	0.45711
H	-1.61137	-0.24448	-2.12873	H	2.72815	-2.75008	-0.81729
H	-3.68346	1.69183	-0.94042	H	2.21234	-0.82871	1.5261
H	-3.95109	0.53495	-2.21648	H	3.53221	-1.98025	1.41561
H	-2.25665	2.09659	0.675	H	2.15956	-1.48947	-2.72644
H	-0.54555	1.93333	1.32849	H	0.5828	-0.53341	-2.81605
H	-7.10978	0.05772	0.21193	H	5.2609	0.58715	2.17012
H	-6.11557	1.01409	-0.90067	H	4.43925	2.1508	2.35135
H	-6.31585	-0.73458	-1.16449	H	3.56496	0.65097	2.70596
H	-4.38265	-1.98447	1.0407	H	5.06919	1.1036	-0.92022
H	-4.6807	1.8427	1.09796	H	1.72105	1.33509	0.98849
H	-5.68579	0.87587	2.19463	H	2.61818	2.82861	0.65686
H	-3.92552	0.63791	2.16693	H	2.2128	1.72584	-0.67581
atom		Con f. 5- 1o		atom		Con f. 5- 1p	
C	-4.27873	-1.36906	-1.42829	C	5.75081	-1.44769	-0.78579
C	-2.53471	-0.59337	2.63106	C	3.95361	2.22642	1.14694
C	-1.97001	-1.09858	1.52798	C	3.75119	1.68077	-0.05838
C	-1.39243	-0.22996	0.42163	C	2.75825	0.55161	-0.30134
C	-2.4427	0.23613	-0.6556	C	3.36262	-0.88321	-0.06341
C	-3.00198	-0.99335	-1.33839	C	4.48792	-1.10879	-1.04975
C	-0.57405	0.95523	0.96127	C	1.43943	0.75119	0.46787
C	0.20034	1.62875	-0.18698	C	0.37985	-0.30868	0.10201
C	-0.805	2.17933	-1.20261	C	0.94522	-1.70997	0.34812
C	-1.67951	1.04306	-1.74551	C	2.25568	-1.91598	-0.41494
C	-3.55324	1.09932	-0.04757	C	3.83098	-1.08166	1.38246
C	-1.81957	-2.58509	1.3336	C	4.49025	2.18602	-1.27071
C	1.25418	2.59662	0.30444	C	-0.94159	-0.01501	0.78658
C	2.444	1.99066	1.02735	C	-1.58183	1.30547	0.38674
C	3.14525	0.8505	0.25919	C	-3.10963	1.32373	0.43566
C	2.51571	-0.51159	0.44943	C	-3.73738	0.34737	-0.52771
C	2.64219	-1.59842	-0.63274	C	-5.17941	-0.12691	-0.29998
C	1.1769	3.91961	0.12248	C	-1.50462	-0.83869	1.67765
O	1.98424	-0.82671	1.51063	O	-3.13096	-0.08079	-1.50507
C	4.12418	-2.01621	-0.68345	C	-6.12707	1.06088	-0.11082
O	1.84111	-2.70941	-0.22783	O	-5.59352	-0.85274	-1.45612
C	2.14893	-1.15921	-2.0121	C	-5.20182	-1.06916	0.91638
H	-0.67901	-0.8644	-0.1222	H	2.50054	0.58055	-1.36933
H	0.73827	0.82421	-0.70584	H	0.1922	-0.21184	-0.97973
H	-4.55765	-2.27633	-1.95822	H	6.46676	-1.60131	-1.58923
H	-5.0909	-0.80117	-0.98413	H	6.12408	-1.58349	0.22492
H	-2.92202	-1.24152	3.41366	H	4.66842	3.03412	1.28499
H	-2.62763	0.47546	2.79768	H	3.42226	1.89599	2.03381
H	-2.24292	-1.61984	-1.81019	H	4.19077	-0.99164	-2.09337
H	-1.21495	1.69964	1.44622	H	1.60911	0.71555	1.54959
H	0.1096	0.58297	1.72509	H	1.06448	1.75463	0.24544
H	-1.42664	2.95066	-0.73284	H	1.11018	-1.86052	1.4212
H	-0.27996	2.65976	-2.03627	H	0.21885	-2.4681	0.03351
H	-1.03458	0.34914	-2.30065	H	2.0504	-1.85226	-1.49187
H	-2.4092	1.43372	-2.46447	H	2.64578	-2.92386	-0.23048
H	-4.13348	0.54338	0.69241	H	3.02678	-0.89308	2.09643
H	-3.14975	1.9863	0.44548	H	4.65625	-0.41027	1.62983

H	-4.23838	1.44069	-0.8303	H	4.1714	-2.1112	1.53356
H	-2.35944	-2.92116	0.44118	H	3.78471	2.55921	-2.02468
H	-0.76425	-2.84212	1.17333	H	5.18124	2.99524	-1.01627
H	-2.191	-3.15025	2.19358	H	5.06215	1.3832	-1.74802
H	3.17527	2.77862	1.22613	H	-1.2041	2.10884	1.031
H	2.13469	1.59607	2.00238	H	-1.26123	1.55922	-0.62882
H	3.24959	1.08814	-0.80171	H	-3.49714	1.15698	1.44364
H	4.16674	0.74003	0.64939	H	-3.4777	2.31753	0.14363
H	1.95933	4.5846	0.48023	H	-2.44982	-0.60675	2.15787
H	0.33633	4.38916	-0.37892	H	-1.0421	-1.77533	1.96934
H	4.46235	-2.34635	0.30381	H	-7.15148	0.6872	-0.0187
H	4.23221	-2.8482	-1.38561	H	-5.88748	1.62845	0.79167
H	4.76315	-1.19386	-1.01738	H	-6.08005	1.73125	-0.97445
H	1.71455	-2.59466	0.73279	H	-4.77671	-0.98989	-1.97274
H	1.09485	-0.87285	-1.97845	H	-4.94821	-0.54844	1.84299
H	2.2515	-2.0023	-2.70226	H	-6.21006	-1.4824	1.01599
H	2.73202	-0.3248	-2.4074	H	-4.49921	-1.89552	0.77223
atom	Con f. 5- 1q			atom	Con f. 5- 1r		
C	6.03553	-0.98342	-0.48828	C	-5.70686	-1.53206	-0.80533
C	3.60938	2.43161	1.19186	C	-4.73441	2.05361	1.32005
C	3.59038	1.85492	-0.01589	C	-3.58688	1.82003	0.67167
C	2.77607	0.60551	-0.32495	C	-3.02578	0.40598	0.58477
C	3.53522	-0.73768	-0.00733	C	-3.41837	-0.38883	-0.7213
C	4.77041	-0.81902	-0.87796	C	-4.92902	-0.4483	-0.82457
C	1.38337	0.63676	0.33139	C	-1.50409	0.38989	0.8244
C	0.49988	-0.53177	-0.11665	C	-0.93968	-1.03954	0.90527
C	1.21286	-1.8675	0.17947	C	-1.30004	-1.80961	-0.37655
C	2.60912	-1.90525	-0.44537	C	-2.81352	-1.80393	-0.60223
C	3.88425	-0.87062	1.47941	C	-2.91196	0.29501	-2.00949
C	4.36559	2.44465	-1.16604	C	-2.8045	2.95937	0.06485
C	-0.90811	-0.51525	0.45527	C	0.54749	-1.05365	1.21893
C	-1.84228	-1.543	-0.15696	C	1.46862	-0.47423	0.16844
C	-3.33317	-1.33828	0.09944	C	2.94591	-0.43631	0.54248
C	-3.89803	-0.09848	-0.54666	C	3.81335	0.16539	-0.52982
C	-5.26186	0.44224	-0.08874	C	5.32214	0.33937	-0.28121
C	-1.29386	0.32157	1.42497	C	0.98337	-1.55763	2.37964
O	-3.30999	0.49325	-1.44637	O	3.35995	0.52767	-1.61182
C	-5.09619	1.0936	1.29581	C	5.97593	-1.01978	0.0026
O	-5.67769	1.43695	-1.02328	O	5.90018	0.88421	-1.46617
C	-6.32027	-0.66386	-0.06494	C	5.55342	1.32518	0.87348
H	2.61477	0.59396	-1.412	H	-3.48827	-0.16392	1.40057
H	0.39367	-0.46584	-1.21134	H	-1.44799	-1.54284	1.73878
H	6.84017	-1.0449	-1.21664	H	-6.78614	-1.44233	-0.90199
H	6.32367	-1.06061	0.55586	H	-5.31807	-2.54013	-0.69611
H	4.19893	3.32619	1.37765	H	-5.15276	3.05448	1.39804
H	3.04401	2.04328	2.03308	H	-5.29515	1.25111	1.79274
H	4.5634	-0.74968	-1.94732	H	-5.40642	0.52337	-0.94075
H	1.48404	0.62076	1.42106	H	-0.99644	0.94173	0.0276
H	0.8986	1.58583	0.07848	H	-1.28651	0.91963	1.75949
H	1.27129	-2.00572	1.26604	H	-0.79137	-1.36416	-1.23841
H	0.63004	-2.70739	-0.21179	H	-0.94207	-2.84317	-0.29994
H	2.50253	-1.87226	-1.53787	H	-3.29147	-2.32204	0.23883
H	3.10308	-2.85482	-0.20735	H	-3.05915	-2.37544	-1.50489
H	4.58389	-0.09398	1.7964	H	-3.44767	1.22862	-2.20227
H	2.99741	-0.79418	2.11172	H	-1.84354	0.5219	-1.97061
H	4.34453	-1.84457	1.67485	H	-3.0825	-0.36346	-2.86831
H	3.68945	2.71472	-1.98788	H	-3.36818	3.89461	0.12608
H	4.91659	3.34094	-0.86591	H	-1.85357	3.10702	0.59068
H	5.08104	1.72291	-1.57442	H	-2.55274	2.78014	-0.98432
H	-1.66829	-1.56889	-1.23908	H	1.35244	-1.04169	-0.76251
H	-1.56951	-2.54034	0.20876	H	1.13833	0.5418	-0.07835
H	-3.89398	-2.18023	-0.33002	H	3.10674	0.12639	1.4694
H	-3.57647	-1.34654	1.16537	H	3.32693	-1.44457	0.74713
H	-2.30094	0.31396	1.82821	H	2.03278	-1.5846	2.65545
H	-0.61824	1.04884	1.86202	H	0.28545	-1.96759	3.10564
H	-4.33553	1.8791	1.25772	H	5.7672	-1.72374	-0.80912
H	-6.04971	1.54697	1.5832	H	7.05925	-0.88107	0.06982
H	-4.81536	0.36585	2.06152	H	5.62544	-1.45031	0.94399
H	-4.88128	1.63444	-1.55176	H	5.15085	1.00599	-2.08046
H	-6.08599	-1.43049	0.67768	H	5.20755	0.92297	1.82891
H	-7.2882	-0.22281	0.19203	H	6.62635	1.52491	0.95214
H	-6.40602	-1.13641	-1.04815	H	5.0388	2.27163	0.67994

atom	Con f. 5- 1s			atom	Con f. 5- 1t		

C	4.14206	2.02546	-1.54826	C	-5.7079	-0.78199	0.33806
C	3.82839	-0.80484	1.98785	C	-3.40772	2.73233	1.21608
C	3.06553	0.22649	1.60549	C	-2.57056	2.07769	0.40211
C	1.93616	0.09022	0.59354	C	-2.38517	0.5705	0.5367
C	2.40082	0.26701	-0.90233	C	-3.30396	-0.3048	-0.40114
C	2.94393	1.66776	-1.08369	C	-4.75474	0.0309	-0.12074
C	1.12259	-1.20333	0.78691	C	-0.90149	0.18228	0.40451
C	-0.11775	-1.228	-0.11082	C	-0.65039	-1.31154	0.68091
C	0.31036	-1.10114	-1.58566	C	-1.53223	-2.16778	-0.24222
C	1.1373	0.1676	-1.8019	C	-3.00617	-1.78777	-0.08993
C	3.42563	-0.79439	-1.31774	C	-3.06641	-0.01848	-1.89971
C	3.26796	1.59846	2.19572	C	-1.75455	2.82834	-0.62273
C	-1.04285	-2.40935	0.10804	C	0.82505	-1.64695	0.58094
C	-2.44887	-2.28494	-0.44512	C	1.43279	-1.66175	-0.81155
C	-3.26092	-1.13663	0.18228	C	2.90417	-1.25135	-0.8752
C	-3.05869	0.21767	-0.45071	C	3.13271	0.18323	-0.46733
C	-3.34164	1.48432	0.37222	C	4.51708	0.623	0.02917
C	-0.6729	-3.52106	0.75415	C	1.53723	-1.90985	1.68247
O	-2.71451	0.35003	-1.622	O	2.24226	1.02625	-0.52488
C	-2.24735	1.65757	1.43923	C	5.62064	0.18797	-0.93887
O	-3.30594	2.60322	-0.51168	O	4.52725	2.0473	0.10745
C	-4.73531	1.4154	1.00673	C	4.73776	0.04018	1.43649
H	1.24527	0.92486	0.77973	H	-2.7013	0.30633	1.55384
H	-0.68654	-0.32007	0.12785	H	-0.96528	-1.50947	1.71433
H	4.40992	3.07384	-1.65253	H	-6.72273	-0.42008	0.48434
H	4.89895	1.30331	-1.83979	H	-5.5309	-1.82458	0.58471
H	4.62381	-0.67497	2.71787	H	-3.55204	3.80778	1.14216
H	3.69297	-1.80799	1.59585	H	-3.97888	2.2144	1.98252
H	2.23746	2.45419	-0.81242	H	-5.02375	1.06205	-0.34411
H	1.74726	-2.07769	0.58072	H	-0.53418	0.43543	-0.59375
H	0.82212	-1.27503	1.83787	H	-0.31368	0.78077	1.10967
H	0.88147	-1.99376	-1.86807	H	-1.21785	-2.04027	-1.28389
H	-0.56683	-1.06717	-2.23782	H	-1.39979	-3.22948	-0.00214
H	0.49451	1.03515	-1.6012	H	-3.31428	-1.99855	0.94195
H	1.44781	0.24402	-2.85067	H	-3.62395	-2.41999	-0.73832
H	3.03909	-1.80573	-1.17626	H	-3.61484	-0.74672	-2.50721
H	4.34758	-0.7081	-0.73833	H	-3.4282	0.97578	-2.17587
H	3.67712	-0.68429	-2.37753	H	-2.01097	-0.08015	-2.17576
H	4.08389	1.60925	2.92453	H	-2.04756	3.88147	-0.65814
H	3.49357	2.33561	1.41782	H	-0.68509	2.7855	-0.38349
H	2.35316	1.94154	2.69672	H	-1.86173	2.40984	-1.62773
H	-2.4255	-2.13387	-1.53011	H	1.33205	-2.66775	-1.23774
H	-2.98321	-3.22202	-0.26727	H	0.85929	-1.00316	-1.46883
H	-4.33345	-1.34175	0.05315	H	3.54634	-1.91392	-0.29015
H	-3.09804	-1.07858	1.26171	H	3.26699	-1.33113	-1.90993
H	-1.36321	-4.3511	0.88376	H	2.59793	-2.13872	1.65787
H	0.32029	-3.65134	1.17179	H	1.06826	-1.90981	2.66339
H	-2.45635	2.56843	2.00858	H	6.57858	0.57986	-0.58362
H	-2.21224	0.81564	2.13535	H	5.69799	-0.90035	-0.99898
H	-1.26834	1.76513	0.96334	H	5.43326	0.58703	-1.94031
H	-2.94248	2.25394	-1.34761	H	3.59021	2.31063	0.03604
H	-4.8039	0.61307	1.74519	H	4.79568	-1.05083	1.42372
H	-4.93934	2.36444	1.51163	H	5.68031	0.43249	1.83022
H	-5.50019	1.25937	0.24001	H	3.92731	0.34243	2.10667

atom	Con f. 5- 2a			atom	Con f. 5- 2b		
C	5.335	-0.65	-1.14292	C	5.42358	0.96551	-0.29368
C	1.10383	2.10884	1.3457	C	0.87559	-2.3768	-0.26103
C	1.46591	1.96827	0.06395	C	1.63675	-1.88078	0.72306
C	2.15855	0.75839	-0.55719	C	2.36946	-0.54207	0.71259
C	3.30319	0.04238	0.25228	C	3.11887	-0.10577	-0.59938
C	4.07108	-0.81052	-0.7471	C	4.09322	0.99013	-0.19541
C	1.07705	-0.25171	-1.0194	C	1.39653	0.5571	1.21097
C	0.48693	-1.03597	0.1552	C	0.40079	1.00433	0.12379
C	1.60891	-1.83212	0.85381	C	1.17203	1.54198	-1.0867
C	2.73253	-0.90909	1.34057	C	2.1388	0.49174	-1.64615
C	4.23221	1.06939	0.90952	C	3.86725	-1.29009	-1.22227
C	1.12	3.03961	-0.94103	C	1.77876	-2.62964	2.02504
C	-0.68266	-1.92976	-0.20727	C	-0.64663	1.94797	0.6854
C	-1.65258	-2.22851	0.91706	C	-1.54209	1.39789	1.78049
C	-2.51458	-1.00364	1.28131	C	-2.31514	0.13429	1.37142
C	-3.43356	-0.58829	0.15801	C	-3.30451	0.37623	0.25914
C	-3.6619	0.89976	-0.13238	C	-3.69671	-0.7745	-0.67713
C	-0.88556	-2.42118	-1.43492	C	-0.81641	3.20223	0.25226

O	-4.00646	-1.41643	-0.54381	O	-3.81363	1.47757	0.07363
C	-2.35238	1.49846	-0.67529	C	-4.11685	-2.01787	0.11113
O	-4.67325	1.00732	-1.1318	O	-4.80745	-0.34603	-1.46306
C	-4.1416	1.63267	1.12531	C	-2.5043	-1.07014	-1.60378
H	2.64495	1.13543	-1.46515	H	3.15316	-0.63257	1.47471
H	0.11795	-0.3032	0.88265	H	-0.1286	0.1026	-0.20873
H	5.77978	-1.32693	-1.86828	H	6.01975	1.81571	0.02876
H	5.97564	0.14515	-0.77346	H	5.97321	0.11752	-0.6912
H	0.57224	2.99885	1.67455	H	0.36014	-3.3264	-0.13677
H	1.32523	1.37529	2.1109	H	0.75145	-1.88838	-1.21892
H	3.51311	-1.64015	-1.17621	H	3.63819	1.88853	0.21636
H	0.28376	0.28328	-1.55011	H	0.86497	0.17504	2.08704
H	1.51994	-0.94444	-1.74201	H	1.9617	1.43235	1.55162
H	1.20793	-2.38771	1.70886	H	0.47167	1.83845	-1.87581
H	1.99534	-2.58046	0.15088	H	1.71949	2.4481	-0.80075
H	3.55819	-1.51006	1.74112	H	2.72908	0.92784	-2.46112
H	2.35942	-0.31065	2.17763	H	1.55751	-0.32059	-2.09363
H	5.08014	0.57049	1.3902	H	4.45858	-0.96384	-2.08403
H	4.63016	1.77681	0.17397	H	4.54897	-1.75505	-0.50182
H	3.70445	1.64543	1.67075	H	3.17068	-2.0568	-1.56376
H	0.56657	2.61893	-1.78979	H	1.1476	-3.52225	2.05653
H	0.51408	3.83529	-0.49871	H	2.82103	-2.93785	2.17958
H	2.03165	3.48879	-1.35634	H	1.51488	-1.99121	2.87779
H	-1.10633	-2.5306	1.81783	H	-2.25442	2.16875	2.0855
H	-2.31019	-3.05647	0.6397	H	-0.94672	1.14751	2.6656
H	-3.16456	-1.25057	2.13198	H	-1.64659	-0.68977	1.11208
H	-1.89573	-0.1631	1.6	H	-2.90934	-0.22318	2.22452
H	-1.732	-3.06838	-1.64856	H	-1.59228	3.83774	0.67159
H	-0.22191	-2.20306	-2.2661	H	-0.19923	3.64344	-0.52331
H	-2.02537	0.94792	-1.56209	H	-3.29001	-2.41822	0.7029
H	-1.55175	1.481	0.06699	H	-4.95187	-1.7852	0.77885
H	-2.5373	2.53882	-0.95934	H	-4.44055	-2.7917	-0.59165
H	-4.82128	0.0923	-1.43874	H	-4.84804	0.62085	-1.33567
H	-3.38815	1.61523	1.91651	H	-1.64975	-1.47058	-1.05431
H	-4.34669	2.67696	0.87048	H	-2.81714	-1.81157	-2.34523
H	-5.06371	1.18082	1.50351	H	-2.19285	-0.16219	-2.12986
atom		Con f. 5- 2c		atom		Con f. 5- 2d	
C	5.33724	0.42592	0.37867	C	-5.4093	-0.21497	-1.28076
C	0.41957	-2.10317	-0.76125	C	-0.67126	-1.92007	1.33133
C	1.09484	-1.88569	0.37433	C	-1.05464	-1.85952	0.04883
C	1.99386	-0.69068	0.6814	C	-1.97655	-0.81161	-0.57069
C	2.99108	-0.1978	-0.43231	C	-3.31754	-0.46244	0.17369
C	4.02959	0.66598	0.26642	C	-4.21769	0.2063	-0.8532
C	1.11863	0.47103	1.2197	C	-1.14717	0.45595	-0.89462
C	0.35975	1.18028	0.09584	C	-0.89284	1.33818	0.34339
C	1.37189	1.7731	-0.90746	C	-2.23219	1.76388	0.95308
C	2.27981	0.6857	-1.49399	C	-3.08307	0.54542	1.33149
C	3.65802	-1.38935	-1.12798	C	-3.97652	-1.72898	0.73064
C	0.94112	-2.83184	1.53922	C	-0.51042	-2.84683	-0.95356
C	-0.63189	2.23188	0.55288	C	0.06341	2.47547	0.02971
C	-1.74045	2.57327	-0.42561	C	1.41603	2.08752	-0.53642
C	-2.98172	1.66682	-0.27043	C	2.13969	1.01764	0.28931
C	-2.78644	0.25751	-0.77664	C	3.42729	0.55009	-0.33038
C	-3.30013	-0.92824	0.04635	C	4.03984	-0.78056	0.1334
C	-0.5738	2.83856	1.74373	C	-0.23702	3.76474	0.22275
O	-2.2506	0.03455	-1.8593	O	3.99988	1.1806	-1.2144
C	-4.81963	-0.80394	0.23194	C	3.15377	-1.92931	-0.38323
O	-3.01967	-2.12645	-0.67547	O	5.33875	-0.89418	-0.44458
C	-2.56795	-0.98676	1.39518	C	4.18295	-0.82812	1.6568
H	2.63346	-1.01196	1.51248	H	-2.2869	-1.23244	-1.53456
H	-0.20732	0.41808	-0.44675	H	-0.39389	0.70413	1.0886
H	5.98617	1.12172	0.90499	H	-5.96418	0.35225	-2.02414
H	5.81665	-0.45489	-0.03795	H	-5.8818	-1.12452	-0.92159
H	-0.24209	-2.96025	-0.85328	H	0.02129	-2.68954	1.66452
H	0.48848	-1.4575	-1.62765	H	-1.03487	-1.24223	2.09387
H	3.64788	1.57883	0.7191	H	-3.83248	1.13123	-1.27784
H	1.75628	1.18758	1.74796	H	-0.20241	0.13865	-1.34527
H	0.41386	0.07908	1.96069	H	-1.66243	1.0577	-1.65222
H	0.84867	2.2799	-1.72567	H	-2.06196	2.36945	1.85031
H	1.96345	2.54076	-0.39359	H	-2.77304	2.40286	0.24457
H	3.04542	1.14536	-2.13101	H	-4.06021	0.8763	1.70367
H	1.68261	0.04429	-2.15018	H	-2.60213	0.02189	2.16439
H	4.41132	-1.04819	-1.84583	H	-4.95007	-1.49649	1.17436
H	4.15338	-2.04794	-0.40627	H	-4.1334	-2.477	-0.05426
H	2.92161	-1.98495	-1.6688	H	-3.35412	-2.18249	1.50353

H	0.64391	-2.29177	2.44716	H	0.22358	-3.52369	-0.50806
H	0.19443	-3.6062	1.34235	H	-1.32345	-3.44847	-1.38039
H	1.89649	-3.32204	1.76817	H	-0.03429	-2.32799	-1.79552
H	-2.06696	3.60519	-0.26876	H	2.04785	2.9761	-0.618
H	-1.38329	2.49727	-1.45754	H	1.29166	1.7044	-1.5571
H	-3.32329	1.66635	0.76566	H	2.38542	1.40309	1.28893
H	-3.79958	2.07884	-0.87765	H	1.50648	0.14447	0.46365
H	-1.29498	3.60508	2.01855	H	0.48413	4.54701	-0.0015
H	0.1826	2.60003	2.48484	H	-1.19922	4.09199	0.60251
H	-5.08862	0.08531	0.80775	H	3.64381	-2.87901	-0.14841
H	-5.32113	-0.76191	-0.73983	H	2.16659	-1.92512	0.08429
H	-5.18057	-1.68516	0.77097	H	3.03235	-1.86141	-1.46894
H	-2.60501	-1.82954	-1.50797	H	5.37746	-0.19105	-1.12049
H	-2.90806	-1.87489	1.93648	H	3.20941	-0.79965	2.15241
H	-1.49099	-1.06817	1.23772	H	4.68356	-1.75973	1.93785
H	-2.77285	-0.10864	2.0119	H	4.78763	0.01133	2.0133

atom	Con f. 5- 2e			atom	Con f. 5- 2f		
C	5.42353	0.96523	-0.29377	C	-5.33492	0.64959	-1.14305
C	0.87545	-2.37692	-0.26084	C	-1.1036	-2.10876	1.34576
C	1.63662	-1.88078	0.72318	C	-1.46561	-1.96815	0.064
C	2.36934	-0.54207	0.71257	C	-2.15844	-0.75838	-0.55716
C	3.11875	-0.10592	-0.59939	C	-3.3031	-0.04242	0.25232
C	4.09318	0.98994	-0.19554	C	-4.07107	0.81038	-0.74711
C	1.39649	0.5572	1.2109	C	-1.07711	0.25185	-1.01945
C	0.40076	1.00449	0.12373	C	-0.48691	1.03605	0.15515
C	1.1721	1.54206	-1.08676	C	-1.60885	1.83218	0.85383
C	2.13864	0.4916	-1.64616	C	-2.73248	0.90915	1.34057
C	3.867	-1.2903	-1.22235	C	-4.23205	-1.06946	0.90961
C	1.77872	-2.6296	2.02517	C	-1.11945	-3.0394	-0.941
C	-0.64655	1.9482	0.68539	C	0.6827	1.92976	-0.20734
C	-1.54212	1.3981	1.7804	C	1.65254	2.2286	0.91705
C	-2.3151	0.1345	1.37133	C	2.51451	1.00373	1.28142
C	-3.3043	0.37634	0.25888	C	3.43338	0.58825	0.15806
C	-3.69675	-0.77462	-0.677	C	3.66161	-0.8998	-0.13239
C	-0.81594	3.20262	0.25257	C	0.88565	2.42116	-1.43499
O	-3.81304	1.47777	0.07286	O	4.00633	1.41635	-0.54377
C	-4.11679	-2.01775	0.11165	C	2.35204	-1.49834	-0.67534
O	-4.80762	-0.34626	-1.46284	O	4.67299	-1.00739	-1.13178
C	-2.50448	-1.07049	-1.60377	C	4.14125	-1.63282	1.12526
H	3.153	-0.63253	1.47474	H	-2.64485	-1.1355	-1.46508
H	-0.12871	0.10283	-0.20884	H	-0.11796	0.30321	0.88256
H	6.01974	1.81543	0.02862	H	-5.77977	1.32641	-1.86847
H	5.97315	0.1172	-0.69122	H	-5.97543	-0.14569	-0.77366
H	0.36003	-3.32653	-0.13649	H	-0.57186	-2.99871	1.67457
H	0.75125	-1.88862	-1.21879	H	-1.32521	-1.37534	2.11102
H	3.63821	1.88843	0.21613	H	-3.51321	1.64011	-1.17616
H	0.86496	0.17527	2.08705	H	-0.28384	-0.28301	-1.55032
H	1.96175	1.43245	1.5514	H	-1.52017	0.94459	-1.74195
H	0.4718	1.83869	-1.87586	H	-1.20785	2.38772	1.70889
H	1.71972	2.44808	-0.80077	H	-1.99528	2.58057	0.15095
H	2.72895	0.92743	-2.46125	H	-3.55817	1.5101	1.74107
H	1.55716	-0.32071	-2.09341	H	-2.35937	0.31076	2.17766
H	4.45847	-0.96402	-2.08401	H	-5.07995	-0.57058	1.39036
H	4.54859	-1.75547	-0.5019	H	-4.63005	-1.77688	0.1741
H	3.17037	-2.05687	-1.56403	H	-3.70421	-1.64551	1.67078
H	1.14753	-3.52218	2.05675	H	-0.56608	-2.61861	-1.78975
H	2.821	-2.93781	2.17965	H	-0.5134	-3.83498	-0.49869
H	1.51495	-1.99114	2.87794	H	-2.031	-3.48875	-1.35635
H	-2.25447	2.16898	2.0853	H	1.10619	2.53074	1.81775
H	-0.94678	1.1478	2.66556	H	2.31018	3.05653	0.63969
H	-1.64656	-0.68963	1.11216	H	3.16458	1.25073	2.132
H	-2.90946	-0.2229	2.22437	H	1.89564	0.16327	1.60027
H	-1.59164	3.83828	0.67197	H	1.7321	3.06836	-1.64863
H	-0.19851	3.64384	-0.5228	H	0.22204	2.20304	-2.26619
H	-4.44079	-2.79171	-0.59085	H	2.53681	-2.53874	-0.95939
H	-3.2898	-2.41804	0.70325	H	1.55137	-1.48077	0.06689
H	-4.9516	-1.78487	0.77957	H	2.02514	-0.94775	-1.56216
H	-4.84798	0.62069	-1.33588	H	4.82094	-0.09239	-1.43882
H	-1.64977	-1.47058	-1.05432	H	5.06331	-1.18097	1.50356
H	-2.81734	-1.81226	-2.34488	H	3.38775	-1.61552	1.91642
H	-2.19329	-0.16269	-2.13028	H	4.34641	-2.67708	0.8703

atom	Con f. 5- 2g			atom	Con f. 5- 2h		

C	5.5182	-0.13892	0.98904	C	3.44253	3.39107	0.70701
C	0.66049	-1.97821	-1.21078	C	4.18159	-2.11433	-0.4187
C	1.09838	-1.85546	0.04915	C	3.87326	-1.42934	0.68945
C	2.06508	-0.79666	0.5726	C	3.05443	-0.14313	0.7565
C	3.32307	-0.42617	-0.29702	C	3.41835	1.01582	-0.24481
C	4.29937	0.27361	0.63661	C	2.80233	2.2855	0.32216
C	1.25553	0.46419	0.96248	C	1.54332	-0.48792	0.7115
C	0.85325	1.30499	-0.26377	C	1.03854	-0.79455	-0.71106
C	2.11968	1.76791	-1.00012	C	1.31532	0.40623	-1.63303
C	2.96777	0.56677	-1.43937	C	2.80421	0.77026	-1.65052
C	3.95936	-1.68399	-0.89938	C	4.93835	1.16234	-0.37718
C	0.5804	-2.77751	1.12536	C	4.28753	-1.94811	2.04396
C	-0.09389	2.42623	0.13067	C	-0.41891	-1.22439	-0.69735
C	-1.55027	2.20681	-0.20937	C	-1.44113	-0.14052	-0.43612
C	-2.11613	0.92117	0.41132	C	-2.85789	-0.63086	-0.1581
C	-3.52675	0.61742	-0.01286	C	-3.81513	0.47754	0.18923
C	-4.01947	-0.83623	0.02916	C	-5.3199	0.17392	0.288
C	0.31755	3.53549	0.7535	C	-0.74078	-2.50896	-0.89003
O	-4.29415	1.48639	-0.41563	O	-3.44184	1.62925	0.39173
C	-3.71034	-1.50105	1.37248	C	-5.59097	-1.02929	1.19467
O	-5.43436	-0.83359	-0.15171	O	-5.96151	1.3137	0.85843
C	-3.35828	-1.59305	-1.13825	C	-5.87031	-0.05418	-1.13083
H	2.47278	-1.2089	1.50382	H	3.24651	0.27218	1.75315
H	0.30962	0.64508	-0.95026	H	1.62001	-1.64471	-1.08488
H	6.12827	0.45064	1.66911	H	2.89267	4.24217	1.10133
H	5.96061	-1.06221	0.62688	H	4.52126	3.50182	0.65023
H	-0.05486	-2.75378	-1.47363	H	4.73925	-3.046	-0.35518
H	1.00159	-1.34699	-2.02197	H	3.9132	-1.78548	-1.41492
H	3.94951	1.21473	1.05598	H	1.71869	2.27518	0.42067
H	1.84396	1.0942	1.63804	H	1.35391	-1.3505	1.36007
H	0.36901	0.15891	1.52675	H	0.97201	0.34664	1.13106
H	1.84407	2.35984	-1.88064	H	0.98489	0.17481	-2.65272
H	2.70336	2.43024	-0.35089	H	0.72561	1.26905	-1.30478
H	3.9027	0.91683	-1.89379	H	2.95843	1.67178	-2.25589
H	2.43172	0.0259	-2.22621	H	3.36272	-0.02949	-2.14755
H	3.28657	-2.15788	-1.61548	H	5.19161	2.0203	-1.00849
H	4.88849	-1.43517	-1.42248	H	5.41226	1.31015	0.59935
H	4.19573	-2.42202	-0.12506	H	5.37674	0.27123	-0.82918
H	1.39214	-3.40093	1.52262	H	4.77882	-2.92304	1.97838
H	0.1856	-2.20683	1.97542	H	4.97592	-1.24571	2.53198
H	-0.21046	-3.43619	0.75565	H	3.41962	-2.04305	2.70929
H	-2.15168	3.06413	0.10536	H	-1.46253	0.54434	-1.29343
H	-1.64991	2.12688	-1.30101	H	-1.10613	0.4738	0.40707
H	-1.48725	0.05748	0.18588	H	-3.26692	-1.19136	-1.00549
H	-2.12995	1.00068	1.50769	H	-2.86515	-1.3348	0.68488
H	-0.38299	4.31376	1.04638	H	-1.76268	-2.87385	-0.86258
H	1.36358	3.70915	0.99182	H	0.02942	-3.2522	-1.08187
H	-2.63405	-1.59029	1.53924	H	-6.67259	-1.1517	1.30698
H	-4.15228	-0.93209	2.19594	H	-5.18452	-1.951	0.77156
H	-4.14032	-2.50729	1.37681	H	-5.15584	-0.87132	2.18621
H	-5.64364	0.04982	-0.50868	H	-5.30034	2.02961	0.80594
H	-3.75446	-2.61275	-1.16207	H	-5.45002	-0.95017	-1.59489
H	-3.59558	-1.10552	-2.0891	H	-6.9561	-0.17379	-1.06832
H	-2.2717	-1.64596	-1.03201	H	-5.65235	0.8089	-1.76779

atom	Con f. 5- 2i			atom	Con f. 5- 2j		
C	-5.17033	-1.60129	-0.76668	C	3.92434	-2.30715	-2.04641
C	-3.16006	2.64683	1.57388	C	3.19017	2.30846	1.16163
C	-2.31759	1.87061	0.88015	C	2.86131	1.9899	-0.09633
C	-2.6059	0.37814	0.76458	C	2.48521	0.60262	-0.60978
C	-3.09972	-0.10303	-0.65848	C	3.40269	-0.60979	-0.20332
C	-3.90011	-1.37351	-0.42798	C	3.08964	-1.73599	-1.17614
C	-1.4548	-0.50251	1.31163	C	0.99251	0.32366	-0.29974
C	-0.30399	-0.77589	0.31761	C	0.75138	-0.09396	1.16374
C	-0.86663	-1.37585	-0.97328	C	1.58937	-1.3407	1.50014
C	-1.90829	-0.44289	-1.59478	C	3.08129	-1.11348	1.23028
C	-3.95861	0.97288	-1.33122	C	4.8828	-0.22024	-0.28209
C	-1.08416	2.47464	0.25476	C	2.77137	3.0634	-1.15232
C	0.79387	-1.59376	0.97468	C	-0.7278	-0.28115	1.44312
C	1.64554	-0.8793	2.00558	C	-1.38857	-1.50629	0.83752
C	2.49041	0.26233	1.40902	C	-2.88568	-1.3712	0.56642
C	3.48173	-0.21559	0.37771	C	-3.20651	-0.31782	-0.4644
C	3.79734	0.66226	-0.83872	C	-4.62931	0.25371	-0.55163
C	1.04058	-2.87281	0.66971	C	-1.39448	0.61071	2.18456

O	4.04754	-1.2998	0.4806	O	-2.3636	0.09908	-1.25297
C	4.2371	2.06358	-0.40166	C	-4.85957	1.17992	0.65599
O	4.86826	0.05522	-1.55822	O	-4.72506	1.02025	-1.75113
C	2.55274	0.71386	-1.74236	C	-5.67563	-0.86192	-0.61221
H	-3.45868	0.20977	1.43152	H	2.56672	0.66538	-1.70158
H	0.13963	0.18613	0.04637	H	1.10026	0.7276	1.79958
H	-5.64127	-2.55622	-0.54651	H	3.58446	-3.10962	-2.69663
H	-5.79119	-0.86492	-1.26841	H	4.96384	-2.01194	-2.15306
H	-2.98983	3.7152	1.68574	H	3.40273	3.34027	1.43231
H	-4.047	2.24151	2.05462	H	3.2716	1.57999	1.95856
H	-3.36292	-2.17514	0.07529	H	2.06689	-2.10601	-1.14854
H	-1.07008	-0.03496	2.22368	H	0.62635	-0.45985	-0.97049
H	-1.86575	-1.47254	1.61554	H	0.40449	1.21992	-0.52515
H	-0.05492	-1.54742	-1.68939	H	1.44782	-1.60689	2.55446
H	-1.31453	-2.35505	-0.76922	H	1.23031	-2.19435	0.91481
H	-2.31038	-0.88868	-2.51249	H	3.63357	-2.0457	1.40115
H	-1.41573	0.4894	-1.89423	H	3.47124	-0.39259	1.95569
H	-4.34464	0.61304	-2.29059	H	5.52293	-1.08514	-0.07903
H	-4.81093	1.26203	-0.70846	H	5.14227	0.16593	-1.27383
H	-3.37032	1.8726	-1.52757	H	5.11994	0.55392	0.44928
H	-1.09575	3.5642	0.35151	H	2.93837	4.06218	-0.73906
H	-0.17269	2.11004	0.7417	H	3.51081	2.8887	-1.94488
H	-0.99591	2.2286	-0.80769	H	1.78763	3.05196	-1.63926
H	2.31186	-1.59737	2.49077	H	-1.23054	-2.3649	1.50224
H	1.01453	-0.44465	2.78865	H	-0.88654	-1.76292	-0.09934
H	1.86463	1.05461	0.9956	H	-3.27536	-2.31636	0.16269
H	3.08505	0.72943	2.20649	H	-3.46044	-1.18897	1.47808
H	1.83792	-3.42249	1.16299	H	-2.4569	0.52912	2.39078
H	0.46625	-3.41895	-0.07159	H	-0.88518	1.46851	2.6169
H	5.11524	2.00329	0.24833	H	-5.83458	1.66302	0.54106
H	4.50241	2.64508	-1.28989	H	-4.85513	0.63074	1.60082
H	3.44015	2.59169	0.12761	H	-4.0889	1.95578	0.69416
H	4.9889	-0.81883	-1.14001	H	-3.80307	1.14422	-2.04584
H	1.71738	1.22839	-1.26231	H	-5.6875	-1.45323	0.30647
H	2.81088	1.25457	-2.65786	H	-6.6652	-0.41378	-0.74393
H	2.23657	-0.2978	-2.01431	H	-5.4807	-1.52583	-1.45987

atom	Con f. 5- 3a			atom	Con f. 5- 3b		
C	5.27711	-0.96465	-0.53014	C	-5.52081	-0.01786	-1.14387
C	1.5293	-2.1463	1.7423	C	-2.85779	1.99857	1.9157
C	1.74699	-1.92453	0.43991	C	-2.71958	1.84689	0.59307
C	1.69263	-0.5363	-0.18257	C	-2.11529	0.59988	-0.03815
C	3.05937	0.24725	-0.13317	C	-3.16326	-0.54695	-0.30392
C	4.0881	-0.51726	-0.93718	C	-4.19529	-0.04503	-1.29081
C	0.54276	0.31255	0.38178	C	-0.89076	0.08007	0.73798
C	0.3704	1.6391	-0.37829	C	-0.18303	-1.05599	-0.00677
C	1.6813	2.43701	-0.32051	C	-1.17771	-2.20823	-0.2549
C	2.84749	1.60504	-0.86341	C	-2.41399	-1.71203	-1.00873
C	3.53287	0.49129	1.30385	C	-3.81742	-1.04721	0.98878
C	2.01774	-3.06666	-0.50485	C	-3.13245	2.94003	-0.35872
C	-0.84547	2.39572	0.13066	C	1.08009	-1.56364	0.66344
C	-2.06931	2.38452	-0.75889	C	2.09478	-2.2093	-0.25764
C	-2.6059	0.98839	-1.10979	C	2.90215	-1.15646	-1.04188
C	-3.19413	0.23183	0.05288	C	3.82106	-0.35894	-0.14674
C	-3.58842	-1.24769	-0.12244	C	3.95638	1.15507	-0.34202
C	-0.85335	3.02686	1.30918	C	1.32146	-1.45017	1.97467
O	-3.39632	0.75918	1.14215	O	4.46585	-0.8941	0.75001
C	-2.32744	-2.12119	-0.23865	C	2.62632	1.81809	0.05869
O	-4.3159	-1.64447	1.03908	O	4.98994	1.6256	0.51971
C	-4.49964	-1.42491	-1.34189	C	4.34345	1.48714	-1.78687
H	1.48046	-0.67966	-1.25175	H	-1.75415	0.8936	-1.03398
H	0.19369	1.38963	-1.43392	H	0.10366	-0.66963	-0.99637
H	5.93883	-1.49084	-1.21349	H	-6.16301	0.35091	-1.93966
H	5.63937	-0.83216	0.485	H	-6.01875	-0.35459	-0.23938
H	1.56621	-3.15184	2.15432	H	-3.28862	2.9045	2.3353
H	1.30992	-1.34534	2.441	H	-2.54947	1.23703	2.625
H	3.79757	-0.69204	-1.97462	H	-3.77153	0.31036	-2.23177
H	0.70427	0.53647	1.44076	H	-1.19568	-0.26482	1.73051
H	-0.37887	-0.27012	0.33143	H	-0.19523	0.91033	0.89784
H	1.58485	3.3585	-0.90666	H	-0.7002	-3.00788	-0.83209
H	1.88355	2.74315	0.71166	H	-1.46081	-2.64452	0.71051
H	2.66624	1.40071	-1.92714	H	-2.10079	-1.37181	-2.00484
H	3.78009	2.1791	-0.81009	H	-3.11851	-2.53712	-1.16712
H	2.78784	1.03585	1.88748	H	-4.48469	-1.88819	0.77442

H	3.73783	-0.44816	1.82249	H	-3.0748	-1.39338	1.71032
H	4.45105	1.08753	1.30474	H	-4.40423	-0.26098	1.46899
H	1.24807	-3.10931	-1.28673	H	-3.54596	3.80358	0.17068
H	2.97733	-2.93819	-1.01675	H	-2.27298	3.27876	-0.95219
H	2.03173	-4.02874	0.01573	H	-3.88326	2.58285	-1.07177
H	-2.87017	2.97033	-0.29799	H	2.78839	-2.83143	0.31396
H	-1.8154	2.87529	-1.70785	H	1.59107	-2.85741	-0.98272
H	-3.4082	1.08477	-1.85329	H	3.54926	-1.65547	-1.77614
H	-1.84536	0.36625	-1.5927	H	2.24287	-0.49418	-1.60572
H	-1.74327	3.5338	1.67319	H	2.23162	-1.84976	2.41362
H	0.02119	3.05976	1.9536	H	0.63038	-0.96292	2.65541
H	-2.63807	-3.16693	-0.32614	H	2.74242	2.90358	-0.01554
H	-1.72816	-1.86698	-1.11689	H	1.80292	1.51371	-0.59171
H	-1.7062	-2.0217	0.65573	H	2.37236	1.56374	1.09165
H	-4.1915	-0.91465	1.67557	H	5.19603	0.87009	1.10258
H	-5.37258	-0.76867	-1.27096	H	5.27763	0.98575	-2.05756
H	-3.9709	-1.21224	-2.27429	H	3.5652	1.19204	-2.49462
H	-4.84817	-2.46162	-1.37387	H	4.49273	2.56776	-1.87329

atom	Con f. 5- 3c			atom	Con f. 5- 3d		
C	5.66488	-0.116	-1.79297	C	5.75135	-0.00672	-0.6932
C	3.15095	2.6271	0.72432	C	2.10864	2.67493	-0.5964
C	3.68692	1.43451	1.01064	C	2.68771	1.94396	0.3638
C	3.00886	0.11622	0.66268	C	2.38274	0.46699	0.5789
C	3.32588	-0.39408	-0.79512	C	3.27295	-0.49674	-0.2934
C	4.81455	-0.64041	-0.90905	C	4.72281	-0.31213	0.0993
C	1.49387	0.14291	0.93538	C	0.88449	0.14991	0.4265
C	0.85082	-1.24157	0.748	C	0.55757	-1.31025	0.7976
C	1.13319	-1.76263	-0.67442	C	1.40743	-2.27231	-0.0491
C	2.63577	-1.77585	-0.96559	C	2.89796	-1.95546	0.0913
C	2.83207	0.5792	-1.87131	C	3.06312	-0.27491	-1.7955
C	5.00146	1.3313	1.74039	C	3.66859	2.56701	1.3236
C	-0.63138	-1.25952	1.08099	C	-0.93092	-1.58291	0.7005
C	-1.49449	-0.23447	0.37905	C	-1.52059	-1.68111	-0.6959
C	-2.99809	-0.41185	0.56204	C	-2.98005	-1.24237	-0.8123
C	-3.81254	0.61592	-0.17575	C	-3.17652	0.22294	-0.511
C	-5.32386	0.39647	-0.35326	C	-4.56096	0.73921	-0.0945
C	-1.11496	-2.15811	1.94684	C	-1.66854	-1.71977	1.8083
O	-3.31242	1.63004	-0.65387	O	-2.25732	1.03186	-0.595
C	-6.00312	0.07745	0.98113	C	-4.84295	0.27465	1.3458
O	-5.88686	1.60384	-0.86385	O	-4.52866	2.16505	-0.1257
C	-5.53874	-0.73109	-1.37875	C	-5.64823	0.26412	-1.0616
H	3.43575	-0.64435	1.33151	H	2.64782	0.23737	1.6207
H	1.34739	-1.93005	1.4452	H	0.84988	-1.4493	1.8473
H	6.71756	-0.38701	-1.78301	H	6.75837	0.07941	-0.293
H	5.35793	0.59641	-2.55303	H	5.63931	0.17028	-1.7588
H	3.65959	3.5502	0.99203	H	2.34585	3.72903	-0.719
H	2.19588	2.73162	0.21924	H	1.38303	2.2606	-1.289
H	5.20233	-1.35045	-0.17649	H	4.91768	-0.47574	1.1606
H	1.00735	0.86881	0.2778	H	0.55436	0.35137	-0.5959
H	1.32568	0.49063	1.96108	H	0.31278	0.82377	1.0733
H	0.73114	-2.77708	-0.77927	H	1.222	-3.30622	0.2658
H	0.61297	-1.14153	-1.41207	H	1.11051	-2.20727	-1.1014
H	3.1189	-2.4907	-0.28632	H	3.19831	-2.12679	1.1335
H	2.82085	-2.13735	-1.98409	H	3.49107	-2.64324	-0.5228
H	3.35068	1.53854	-1.81042	H	3.39109	0.7213	-2.1007
H	3.00548	0.16114	-2.86825	H	3.63181	-1.01213	-2.3716
H	1.76214	0.77592	-1.77944	H	2.01319	-0.37598	-2.0778
H	5.73489	0.76338	1.15802	H	3.82025	3.63016	1.1146
H	5.42401	2.317	1.95692	H	3.31337	2.46618	2.3576
H	4.87294	0.79543	2.69007	H	4.64141	2.06611	1.2768
H	-1.21049	0.76705	0.72325	H	-0.92244	-1.08216	-1.3876
H	-1.26254	-0.24079	-0.69288	H	-1.4368	-2.71622	-1.0499
H	-3.32305	-1.41237	0.25828	H	-3.32929	-1.38625	-1.8447
H	-3.27615	-0.32474	1.62134	H	-3.64842	-1.84562	-0.1938
H	-2.16799	-2.22782	2.20063	H	-2.73923	-1.89587	1.7851
H	-0.45471	-2.86858	2.43838	H	-1.21231	-1.66172	2.7935
H	-7.0836	0.00941	0.82166	H	-5.78175	0.72811	1.6779
H	-5.65713	-0.87448	1.39109	H	-4.94004	-0.8117	1.4156
H	-5.81019	0.86962	1.71095	H	-4.04223	0.60102	2.0164
H	-5.12199	2.13706	-1.15233	H	-3.58129	2.39532	-0.1693
H	-5.1706	-1.69354	-1.01403	H	-5.75843	-0.82277	-1.0412
H	-6.61097	-0.82285	-1.57646	H	-6.60351	0.71041	-0.7688
H	-5.032	-0.49251	-2.31919	H	-5.41807	0.57904	-2.084

atom	Con f. 5- 3e			atom	Con f. 5- 3f		
C	-5.25043	2.18071	-0.25498	C	4.85023	-1.07548	-1.74107
C	-3.90163	-1.88189	-1.55999	C	3.46985	-0.2026	2.46821
C	-4.04345	-1.37731	-0.32844	C	2.92707	-0.96116	1.50825
C	-2.96072	-0.5584	0.36203	C	2.01714	-0.39919	0.42387
C	-3.0093	0.9768	0.01008	C	2.79378	0.14454	-0.83357
C	-4.33454	1.54066	0.47359	C	3.54485	-1.00085	-1.47682
C	-1.55281	-1.14238	0.14442	C	1.01812	0.63508	0.97207
C	-0.48387	-0.40725	0.97167	C	-0.00101	1.08504	-0.09386
C	-0.50811	1.10112	0.65654	C	0.73556	1.63497	-1.31886
C	-1.91013	1.68201	0.85244	C	1.73055	0.61023	-1.86679
C	-2.77513	1.23712	-1.48253	C	3.73034	1.3048	-0.4787
C	-5.29775	-1.62426	0.46969	C	3.16753	-2.44819	1.46752
C	0.90312	-0.99328	0.79121	C	-1.04913	2.01395	0.49092
C	1.39021	-1.16615	-0.63631	C	-1.877	1.47507	1.64395
C	2.90643	-1.12435	-0.81639	C	-2.49704	0.09276	1.39466
C	3.50679	0.21539	-0.47023	C	-3.44501	0.05305	0.22396
C	5.00805	0.32136	-0.16594	C	-3.76048	-1.28779	-0.45588
C	1.64322	-1.32446	1.85525	C	-1.26693	3.25361	0.03845
O	2.83247	1.23993	-0.42838	O	-3.98532	1.0664	-0.20887
C	5.84902	-0.36232	-1.24681	C	-4.17409	-2.3452	0.57118
O	5.35672	1.70452	-0.13781	O	-4.85159	-1.08174	-1.35129
C	5.26587	-0.29181	1.22205	C	-2.52828	-1.73592	-1.26209
H	-3.16233	-0.61248	1.44108	H	1.41621	-1.23957	0.04822
H	-0.75337	-0.51803	2.03078	H	-0.5268	0.17749	-0.42521
H	-6.16333	2.55582	0.20064	H	5.27206	-1.95113	-2.22785
H	-5.13632	2.35635	-1.32054	H	5.54733	-0.28249	-1.48703
H	-4.69392	-2.46555	-2.02265	H	4.11279	-0.63626	3.23049
H	-3.00193	-1.73771	-2.14988	H	3.29774	0.86709	2.53404
H	-4.52423	1.40381	1.53962	H	2.90976	-1.84092	-1.76366
H	-1.2918	-1.10052	-0.91699	H	1.53938	1.51859	1.3561
H	-1.56474	-2.20293	0.42066	H	0.50105	0.1856	1.82503
H	0.20005	1.61954	1.31283	H	0.01516	1.89244	-2.10368
H	-0.16531	1.2775	-0.36854	H	1.25597	2.56298	-1.05552
H	-2.18103	1.60052	1.91349	H	1.17384	-0.27099	-2.21285
H	-1.91362	2.75135	0.60975	H	2.24785	1.01741	-2.74352
H	-3.57173	0.80737	-2.09406	H	4.52585	0.98225	0.19666
H	-2.73845	2.3137	-1.67835	H	4.1943	1.70851	-1.38447
H	-1.83025	0.80883	-1.82262	H	3.19534	2.12214	0.00894
H	-5.77395	-0.6818	0.7606	H	3.82551	-2.77481	2.27829
H	-6.02552	-2.21755	-0.09186	H	2.21877	-2.99439	1.55201
H	-5.06319	-2.15728	1.40057	H	3.61844	-2.75016	0.51617
H	1.01929	-2.1218	-1.02776	H	-1.25249	1.39343	2.54164
H	0.94249	-0.39473	-1.26917	H	-2.67073	2.18962	1.87799
H	3.41512	-1.91678	-0.26144	H	-1.73608	-0.68401	1.28376
H	3.15793	-1.30237	-1.87167	H	-3.08938	-0.20366	2.27188
H	2.64891	-1.72403	1.77238	H	-2.0429	3.8786	0.47362
H	1.25486	-1.20476	2.86373	H	-0.6905	3.69392	-0.76812
H	6.90893	-0.20596	-1.02408	H	-4.45887	-3.26074	0.04369
H	5.66191	-1.43819	-1.28143	H	-3.35535	-2.5845	1.25397
H	5.63497	0.0676	-2.23001	H	-5.03344	-2.0003	1.15415
H	4.50502	2.17821	-0.08714	H	-4.93135	-0.11207	-1.43139
H	5.0647	-1.36582	1.23817	H	-1.66993	-1.94626	-0.6192
H	6.31658	-0.13195	1.48226	H	-2.78331	-2.64939	-1.80799
H	4.64189	0.19609	1.97705	H	-2.24741	-0.96611	-1.98736

atom	Con f. 5- 3g			atom	Con f. 5- 3h		
C	-4.94488	-0.81217	-1.35191	C	-6.24377	-0.06292	-0.54167
C	-1.56017	-2.50117	0.90666	C	-3.05233	2.26709	1.68412
C	-2.34076	-1.46801	1.24554	C	-3.17722	1.96315	0.38663
C	-2.1048	-0.04845	0.74797	C	-2.69526	0.64491	-0.20485
C	-2.70924	0.23169	-0.67948	C	-3.76111	-0.5124	-0.12256
C	-4.20631	0.02219	-0.61873	C	-4.97205	-0.11907	-0.94044
C	-0.62583	0.37269	0.82824	C	-1.33408	0.20531	0.36369
C	-0.43939	1.86759	0.4949	C	-0.77795	-1.04095	-0.35574
C	-1.01849	2.17682	-0.89369	C	-1.7913	-2.18569	-0.25199
C	-2.48147	1.73764	-0.98832	C	-3.14948	-1.76156	-0.81753
C	-2.06127	-0.63355	-1.76663	C	-4.14208	-0.84691	1.32404
C	-3.50993	-1.65291	2.178	C	-3.78704	2.94239	-0.58318
C	0.99877	2.32937	0.69252	C	0.62091	-1.3637	0.14299
C	1.97569	2.28866	-0.46514	C	1.70366	-0.53297	-0.50929
C	2.34655	0.89556	-0.99512	C	3.09561	-0.65105	0.10115
C	3.16225	0.06386	-0.04007	C	4.12248	0.20658	-0.58823

C	3.39969	-1.42635	-0.3407	C	5.47871	0.45653	0.09159
C	1.38701	2.79132	1.88677	C	0.86602	-2.28325	1.08317
O	3.67075	0.53714	0.97142	O	3.91577	0.72985	-1.67937
C	3.94766	-1.62296	-1.75748	C	6.11487	-0.84487	0.58375
O	4.37061	-1.91063	0.5856	O	6.35349	1.04063	-0.87276
C	2.0862	-2.19743	-0.13467	C	5.26457	1.45292	1.24545
H	-2.65463	0.61927	1.42591	H	-2.54091	0.81764	-1.27927
H	-1.04658	2.41858	1.22674	H	-0.68729	-0.78027	-1.42136
H	-6.023	-0.86356	-1.22285	H	-7.03421	0.21537	-1.23408
H	-4.51686	-1.47198	-2.10066	H	-6.55071	-0.28607	0.47578
H	-1.7579	-3.50156	1.28441	H	-3.40307	3.21948	2.07444
H	-0.70605	-2.39458	0.24779	H	-2.6011	1.58818	2.40082
H	-4.71148	0.65323	0.1145	H	-4.7448	0.11907	-1.98098
H	-0.01971	-0.24266	0.16058	H	-1.41004	-0.01659	1.43342
H	-0.25845	0.18329	1.84255	H	-0.6335	1.04152	0.26898
H	-0.94831	3.25216	-1.09678	H	-1.42263	-3.06308	-0.796
H	-0.42995	1.67512	-1.66924	H	-1.9066	-2.49113	0.79354
H	-3.07524	2.33018	-0.27966	H	-3.03353	-1.54214	-1.88731
H	-2.87742	1.95544	-1.98729	H	-3.86546	-2.58859	-0.74367
H	-2.26261	-1.69493	-1.60611	H	-4.63837	-0.00418	1.81053
H	-2.45098	-0.35842	-2.75217	H	-4.82296	-1.70391	1.34877
H	-0.97719	-0.50408	-1.79444	H	-3.26676	-1.10467	1.92352
H	-4.44662	-1.34301	1.70126	H	-4.09531	3.86679	-0.08577
H	-3.61536	-2.69441	2.49604	H	-3.07037	3.1985	-1.37446
H	-3.39273	-1.02851	3.07347	H	-4.66245	2.51239	-1.08164
H	1.56006	2.8537	-1.30697	H	1.75003	-0.80138	-1.57285
H	2.8955	2.80078	-0.16871	H	1.39699	0.52064	-0.49828
H	1.4713	0.32673	-1.32	H	3.08609	-0.41247	1.16999
H	2.9639	1.01028	-1.89686	H	3.4651	-1.6839	0.03787
H	2.41008	3.10636	2.0746	H	1.86739	-2.4822	1.45138
H	0.69213	2.86457	2.72075	H	0.07183	-2.87672	1.52469
H	4.18045	-2.68224	-1.9029	H	7.10751	-0.62412	0.98808
H	3.21963	-1.32501	-2.51591	H	5.51968	-1.30963	1.37342
H	4.86536	-1.04401	-1.89917	H	6.22704	-1.55496	-0.24112
H	4.46618	-1.19836	1.2465	H	5.76941	1.34725	-1.59169
H	1.32332	-1.90267	-0.85925	H	4.63497	1.03746	2.03655
H	2.2854	-3.26622	-0.25874	H	6.23918	1.70431	1.67442
H	1.69915	-2.03192	0.87462	H	4.80191	2.37221	0.87276

atom	Con f. 5- 3i			atom	Con f. 5- 3j		
C	-5.84208	-1.98021	-0.00427	C	4.8559	-0.55442	-1.73512
C	-4.53022	2.33538	0.46869	C	1.64231	-3.11533	-0.35267
C	-4.32575	1.44768	-0.51196	C	1.54792	-2.05403	0.45823
C	-3.18206	0.44135	-0.49297	C	1.66283	-0.64498	-0.1091
C	-3.53586	-0.90329	0.25056	C	3.07851	0.03976	0.00888
C	-4.66429	-1.58629	-0.49103	C	4.10306	-0.84421	-0.6726
C	-1.87069	1.05293	0.0337	C	0.55479	0.26525	0.44373
C	-0.68581	0.09288	-0.11918	C	0.49967	1.61912	-0.28264
C	-0.99387	-1.21995	0.62841	C	1.86035	2.31579	-0.1412
C	-2.30181	-1.8416	0.13393	C	2.98096	1.41993	-0.6805
C	-3.88671	-0.67723	1.72573	C	3.54165	0.21272	1.47075
C	-5.21521	1.42256	-1.72852	C	1.24523	-2.23444	1.92558
C	0.65645	0.66408	0.30881	C	-0.69114	2.4331	0.19371
C	1.84264	-0.00646	-0.34903	C	-1.89089	2.47559	-0.72868
C	3.21274	0.40885	0.17335	C	-2.46091	1.10806	-1.13654
C	4.34978	-0.33257	-0.47671	C	-3.09843	0.33015	-0.01453
C	5.79685	-0.04576	-0.03604	C	-3.54996	-1.12414	-0.25532
C	0.77603	1.65734	1.19717	C	-0.70283	3.06826	1.36994
O	4.16839	-1.16931	-1.35669	O	-3.29211	0.82	1.09371
C	5.97923	-0.40468	1.4454	C	-2.32086	-2.0455	-0.34948
O	6.6614	-0.87228	-0.81387	O	-4.34005	-1.52453	0.86358
C	6.15208	1.42312	-0.30513	C	-4.41842	-1.22741	-1.51278
H	-2.9981	0.15254	-1.53745	H	1.4932	-0.73507	-1.18966
H	-0.6018	-0.1605	-1.18724	H	0.35658	1.4121	-1.35258
H	-6.56887	-2.48113	-0.63874	H	5.56761	-1.27782	-2.12552
H	-6.13277	-1.82283	1.03015	H	4.80388	0.39626	-2.25757
H	-5.35287	3.04486	0.42111	H	1.5453	-4.13156	0.02229
H	-3.89489	2.39259	1.34693	H	1.82688	-3.00256	-1.41817
H	-4.44763	-1.78108	-1.54278	H	4.2224	-1.82322	-0.21018
H	-1.98702	1.32847	1.08587	H	0.69834	0.44974	1.51341
H	-1.66943	1.9821	-0.51063	H	-0.40207	-0.25144	0.35128
H	-0.18003	-1.94018	0.491	H	1.85428	3.26515	-0.68943
H	-1.04696	-1.00844	1.7031	H	2.04458	2.56301	0.91029
H	-2.17726	-2.12303	-0.92026	H	2.80845	1.26385	-1.75296

H	-2.51269	-2.76741	0.68224	H	3.94552	1.93311	-0.58942
H	-4.78393	-0.06368	1.83434	H	3.77214	-0.75123	1.93219
H	-4.06801	-1.63579	2.22257	H	4.45599	0.8155	1.49883
H	-3.0781	-0.17756	2.26309	H	2.79282	0.71272	2.08989
H	-4.62916	1.60804	-2.63831	H	1.23647	-3.29453	2.194
H	-5.68729	0.44252	-1.85509	H	1.97092	-1.72564	2.56649
H	-6.00425	2.17808	-1.66825	H	0.26353	-1.8166	2.17767
H	1.73746	-1.09382	-0.25091	H	-2.68653	3.07418	-0.27507
H	1.79165	0.18465	-1.42944	H	-1.59628	2.98327	-1.65695
H	3.38584	1.48154	0.02445	H	-3.24129	1.25506	-1.8949
H	3.28343	0.25334	1.25657	H	-1.70895	0.47865	-1.62374
H	1.74137	2.0391	1.51372	H	-1.58009	3.61339	1.70893
H	-0.09061	2.13105	1.64665	H	0.15513	3.06772	2.03696
H	7.03711	-0.29804	1.70392	H	-2.66761	-3.07297	-0.49715
H	5.39801	0.25184	2.09766	H	-1.66617	-1.78042	-1.1834
H	5.68081	-1.44162	1.6289	H	-1.74645	-2.00828	0.5798
H	6.06611	-1.38301	-1.39568	H	-4.18223	-0.84005	1.54132
H	5.57368	2.10324	0.32501	H	-3.84277	-1.017	-2.41738
H	7.21414	1.57219	-0.08825	H	-4.81221	-2.24561	-1.58642
H	5.97503	1.67577	-1.35529	H	-5.26243	-0.53294	-1.45907

atom	Con f. 5- 3k			atom	Con f. 5- 3l		
C	4.64062	-1.53061	-0.89339	C	4.54816	-0.5227	2.41624
C	1.42229	-1.80601	2.17348	C	3.12435	2.92788	0.31767
C	1.31823	-1.76614	0.83914	C	2.74511	1.95122	-0.51561
C	1.32617	-0.46716	0.04624	C	2.14595	0.66064	0.0308
C	2.76439	0.09208	-0.26857	C	3.17814	-0.51302	0.25321
C	3.48238	-0.91624	-1.13874	C	4.25774	-0.04129	1.20651
C	0.43233	0.60981	0.67688	C	0.93633	0.20763	-0.80886
C	0.27403	1.82306	-0.25773	C	0.20741	-0.98488	-0.18482
C	1.65979	2.43292	-0.50299	C	1.20305	-2.1486	-0.00499
C	2.59468	1.38469	-1.11967	C	2.40126	-1.71151	0.83915
C	3.55792	0.40666	1.00424	C	3.89237	-0.93371	-1.0491
C	1.11644	-3.02228	0.03333	C	2.85245	2.13568	-2.0101
C	-0.78776	2.77597	0.26048	C	-1.0325	-1.431	-0.93773
C	-2.20664	2.55972	-0.23059	C	-2.03974	-2.2347	-0.14081
C	-2.88889	1.25384	0.2246	C	-2.85904	-1.34946	0.81957
C	-2.56186	0.01884	-0.57966	C	-3.74258	-0.37116	0.08345
C	-3.06241	-1.36207	-0.10708	C	-3.86392	1.07381	0.57904
C	-0.51272	3.76577	1.1177	C	-1.26526	-1.13452	-2.22169
O	-1.9822	0.07017	-1.66058	O	-4.37127	-0.70258	-0.91736
C	-4.58782	-1.40233	-0.31968	C	-2.51066	1.77696	0.37469
O	-2.44861	-2.35181	-0.93546	O	-4.85347	1.73316	-0.20757
C	-2.7035	-1.68254	1.34483	C	-4.30713	1.10896	2.04585
H	0.881	-0.69586	-0.93199	H	1.77304	0.89175	1.03686
H	-0.08027	1.44081	-1.22152	H	-0.11115	-0.68492	0.82459
H	5.06023	-2.23576	-1.60643	H	5.36251	-0.10102	3.0004
H	5.21458	-1.36338	0.01341	H	4.00362	-1.34223	2.87534
H	1.39926	-2.75062	2.71162	H	3.54934	3.85908	-0.0499
H	1.53286	-0.90912	2.77464	H	3.02395	2.82847	1.39559
H	2.96583	-1.13922	-2.07396	H	4.85936	0.7818	0.82422
H	0.83555	0.95348	1.63549	H	1.26421	-0.05465	-1.81959
H	-0.5361	0.1632	0.90564	H	0.24426	1.05027	-0.91541
H	1.57966	3.29245	-1.17878	H	0.71459	-3.00092	0.47948
H	2.08165	2.80928	0.43562	H	1.5268	-2.49322	-0.99454
H	2.19661	1.10237	-2.1034	H	2.03409	-1.44107	1.8373
H	3.58686	1.81654	-1.29676	H	3.09084	-2.55283	0.9751
H	3.73627	-0.49251	1.59772	H	4.56722	-0.1496	-1.40286
H	4.52848	0.84273	0.74615	H	4.49694	-1.82873	-0.86593
H	3.03352	1.12404	1.63924	H	3.19105	-1.16355	-1.85499
H	1.16063	-3.91946	0.65811	H	3.38573	1.3131	-2.49531
H	0.13645	-2.99459	-0.46057	H	1.85901	2.17593	-2.47281
H	1.87021	-3.11225	-0.75686	H	3.37091	3.06801	-2.25114
H	-2.82735	3.39015	0.11793	H	-2.72778	-2.75104	-0.81529
H	-2.21956	2.58777	-1.3272	H	-1.53354	-2.99819	0.45921
H	-2.70294	1.06055	1.28516	H	-3.53201	-1.97962	1.41691
H	-3.97796	1.37366	0.14006	H	-2.21225	-0.82787	1.52668
H	-1.29323	4.42429	1.49139	H	-2.15975	-1.49083	-2.72537
H	0.49418	3.95702	1.47774	H	-0.58292	-0.5349	-2.8156
H	-4.94401	-2.4125	-0.09696	H	-2.61883	2.82894	0.65545
H	-5.10736	-0.69828	0.33595	H	-1.72144	1.33582	0.98817
H	-4.83629	-1.1666	-1.3593	H	-2.21292	1.72543	-0.67645
H	-2.12687	-1.8649	-1.7176	H	-5.0691	1.10262	-0.92106
H	-3.19292	-0.99726	2.04058	H	-3.56544	0.65233	2.70571

H	-3.04568	-2.69713	1.57075	H	-4.43999	2.15176	2.35005
H	-1.62282	-1.64364	1.5056	H	-5.26129	0.58785	2.16968

atom	Con f. 5- 3m			atom	Con f. 5- 3n		
C	-5.29144	-2.206	0.14234	C	4.27778	-1.36778	-1.42926
C	-4.06661	1.91384	1.52359	C	2.53753	-0.58802	2.63101
C	-4.12694	1.38464	0.29557	C	1.97222	-1.09539	1.52923
C	-2.99284	0.56784	-0.31	C	1.39243	-0.22896	0.4223
C	-3.05685	-0.96464	0.05327	C	2.44125	0.2368	-0.65647
C	-4.33373	-1.54723	-0.5122	C	3.00081	-0.99308	-1.33831
C	-1.60711	1.16499	-0.00143	C	0.57336	0.95603	0.96141
C	-0.48818	0.44112	-0.75789	C	-0.20238	1.62785	-0.18695
C	-0.51226	-1.05979	-0.40022	C	0.80171	2.17814	-1.20397
C	-1.88541	-1.67063	-0.68498	C	1.67641	1.04188	-1.74654
C	-2.94725	-1.20812	1.56315	C	3.55166	1.10161	-0.05057
C	-5.33333	1.60031	-0.58151	C	1.82377	-2.5823	1.33673
C	0.90036	1.01748	-0.55258	C	-1.25671	2.59536	0.30419
C	1.86511	0.72455	-1.6876	C	-2.44575	1.98916	1.02815
C	3.34677	0.72304	-1.31758	C	-3.14687	0.84826	0.26097
C	3.71164	-0.36665	-0.33986	C	-2.51493	-0.51292	0.44999
C	4.99743	-0.24449	0.49127	C	-2.63966	-1.59942	-0.63275
C	1.25163	1.71307	0.53439	C	-1.18053	3.91822	0.12085
O	3.0161	-1.36669	-0.19024	O	-1.98277	-0.82787	1.51089
C	4.80217	0.8548	1.54963	C	-2.15286	-1.15783	-2.01355
O	5.21646	-1.48976	1.15202	O	-1.83232	-2.70704	-0.23089
C	6.20559	0.04485	-0.40428	C	-4.12002	-2.02364	-0.67878
H	-3.11965	0.61071	-1.40102	H	0.67918	-0.86491	-0.12001
H	-0.7133	0.51728	-1.83329	H	-0.73997	0.82228	-0.70452
H	-6.15977	-2.59562	-0.38292	H	4.55685	-2.2755	-1.95834
H	-5.25642	-2.38316	1.21318	H	5.08998	-0.79861	-0.9868
H	-4.8921	2.49622	1.92587	H	2.92659	-1.23466	3.414
H	-3.20295	1.79056	2.16918	H	2.62922	0.48115	2.79618
H	-4.44305	-1.41062	-1.58941	H	2.24173	-1.62085	-1.80838
H	-1.4144	1.11605	1.07439	H	1.21392	1.70139	1.44537
H	-1.61034	2.22735	-0.269	H	-0.10955	0.58378	1.72592
H	0.25222	-1.60186	-0.96587	H	0.27561	2.65747	-2.03759
H	-0.25039	-1.17123	0.65857	H	1.4233	2.95026	-0.73544
H	-2.07351	-1.62064	-1.76572	H	1.03135	0.34688	-2.30016
H	-1.89086	-2.73338	-0.41528	H	2.40511	1.43217	-2.46671
H	-3.79357	-0.77491	2.10091	H	3.14791	1.98897	0.44158
H	-2.92406	-2.28235	1.77337	H	4.13293	0.54712	0.68969
H	-2.03523	-0.77395	1.97776	H	4.23589	1.44239	-0.83436
H	-5.7846	0.64678	-0.87607	H	2.19709	-3.14595	2.19689
H	-6.09756	2.2004	-0.07866	H	0.76867	-2.84111	1.17794
H	-5.04862	2.11238	-1.5101	H	2.36309	-2.91844	0.44399
H	1.61135	-0.24439	-2.12868	H	-2.1356	1.59518	2.00316
H	1.7015	1.46241	-2.48438	H	-3.17734	2.77681	1.22701
H	3.95113	0.53522	-2.21635	H	-4.16755	0.73647	0.65288
H	3.68337	1.69191	-0.94018	H	-3.25316	1.08598	-0.79972
H	2.25678	2.09667	0.6749	H	-1.96328	4.583	0.4783
H	0.5457	1.93357	1.32846	H	-0.34058	4.38786	-0.38153
H	5.68567	0.87547	2.19486	H	-2.2534	-2.00145	-2.70336
H	4.68068	1.84252	1.09829	H	-2.74145	-0.32641	-2.40698
H	3.9254	0.63759	2.16703	H	-1.10016	-0.86607	-1.98337
H	4.38251	-1.9846	1.04045	H	-1.70698	-2.59439	0.73012
H	6.11554	1.0142	-0.90048	H	-4.45353	-2.35575	0.30941
H	7.10973	0.05767	0.21202	H	-4.76365	-1.20388	-1.01015
H	6.31584	-0.73444	-1.16453	H	-4.22673	-2.85571	-1.38103

atom	Con f. 5- 3o			atom	Con f. 5- 3p		
C	-5.75103	-1.4476	-0.78556	C	5.70654	-1.47504	-0.78926
C	-3.95352	2.22649	1.14703	C	4.6662	2.16332	1.22119
C	-3.75129	1.68079	-0.05831	C	3.51737	1.87784	0.59617
C	-2.7584	0.55161	-0.30137	C	2.99164	0.44739	0.56929
C	-3.36276	-0.88319	-0.06337	C	3.39041	-0.38816	-0.70875
C	-4.48815	-1.10877	-1.04962	C	4.90081	-0.41242	-0.82991
C	-1.4395	0.75119	0.46771	C	1.47366	0.40191	0.82924
C	-0.37996	-0.30872	0.1018	C	0.94548	-1.03764	0.97127
C	-0.94534	-1.70999	0.34797	C	1.3154	-1.84896	-0.28156
C	-2.25587	-1.91599	-0.41499	C	2.82521	-1.81276	-0.52541
C	-3.83101	-1.08162	1.38253	C	2.8509	0.23119	-2.01623
C	-4.49049	2.18604	-1.27055	C	2.70083	2.97419	-0.04412
C	0.94153	-0.01506	0.78627	C	-0.53926	-1.06409	1.29324
C	1.58183	1.30534	0.38626	C	-1.47837	-0.6665	0.17631

C	3.10961	1.32365	0.43539	C	-2.94049	-0.49893	0.57659
C	3.73757	0.34717	-0.52773	C	-3.82808	-0.10803	-0.57438
C	5.17967	-0.12681	-0.29979	C	-5.24305	0.4245	-0.29377
C	1.50454	-0.8387	1.6774	C	-0.9575	-1.41836	2.51404
O	3.13127	-0.08133	-1.50501	O	-3.46063	-0.18995	-1.74288
C	5.20214	-1.06887	0.91672	C	-5.12248	1.85877	0.25237
O	5.59401	-0.85274	-1.45579	O	-5.95496	0.45581	-1.53003
C	6.12711	1.06117	-0.11073	C	-6.00634	-0.4839	0.67248
H	-2.50079	0.58055	-1.36939	H	3.47703	-0.07828	1.40128
H	-0.1924	-0.21191	-0.97997	H	1.46995	-1.49254	1.82247
H	-6.46704	-1.60122	-1.58894	H	6.78169	-1.36075	-0.90445
H	-6.12423	-1.58332	0.22519	H	5.34562	-2.48887	-0.64401
H	-4.66828	3.03421	1.28516	H	5.06087	3.17613	1.25682
H	-3.42203	1.89608	2.03382	H	5.25217	1.39236	1.71522
H	-4.19106	-0.99168	-2.09327	H	5.35144	0.56692	-0.9826
H	-1.60909	0.71558	1.54944	H	0.93983	0.91066	0.02063
H	-1.06455	1.75462	0.24522	H	1.25482	0.95929	1.74757
H	-0.21901	-2.46814	0.03331	H	0.98573	-2.88791	-0.16166
H	-1.11021	-1.86053	1.42106	H	0.78711	-1.45164	-1.15503
H	-2.05068	-1.85229	-1.49193	H	3.32702	-2.28385	0.32928
H	-2.64598	-2.92385	-0.23047	H	3.07527	-2.41303	-1.40797
H	-4.65626	-0.41022	1.62996	H	1.77844	0.43627	-1.97026
H	-4.17143	-2.11116	1.53367	H	3.36275	1.1679	-2.25329
H	-3.02676	-0.89304	2.09644	H	3.02467	-0.45724	-2.85052
H	-5.18146	2.99525	-1.01603	H	3.24055	3.92515	-0.0208
H	-3.78504	2.55923	-2.0246	H	2.44604	2.75102	-1.08418
H	-5.06245	1.38321	-1.74781	H	1.7503	3.11603	0.48389
H	1.26135	1.55888	-0.62939	H	-1.1242	0.26721	-0.27746
H	1.20399	2.10882	1.03031	H	-1.41374	-1.41177	-0.62513
H	3.47769	2.31742	0.14326	H	-3.34683	-1.43983	0.97263
H	3.49699	1.15709	1.44344	H	-3.05471	0.22912	1.38651
H	2.44977	-0.60679	2.15757	H	-2.00481	-1.43426	2.79876
H	1.04193	-1.77526	1.9692	H	-0.24665	-1.71021	3.28333
H	6.21045	-1.4819	1.0165	H	-6.12854	2.27193	0.3718
H	4.94834	-0.54806	1.84323	H	-4.61838	1.88901	1.2217
H	4.49971	-1.89539	0.77262	H	-4.57006	2.48812	-0.45245
H	4.77723	-0.99031	-1.97235	H	-5.26964	0.36505	-2.21874
H	5.88736	1.62881	0.79167	H	-6.05382	-1.50582	0.28428
H	7.15158	0.68769	-0.0185	H	-5.54057	-0.50141	1.66065
H	6.08002	1.73142	-0.97445	H	-7.02801	-0.10758	0.78216

atom	Con f. 5- 3q			atom	Con f. 5- 3r		
C	-4.142	2.02561	-1.54821	C	5.4129	-0.76873	-1.75798
C	-3.82861	-0.80479	1.98765	C	3.60227	-1.11912	2.36912
C	-3.06566	0.22653	1.60544	C	3.15047	-1.52057	1.17477
C	-1.93621	0.09028	0.59359	C	2.38371	-0.60545	0.22902
C	-2.4008	0.2671	-0.90234	C	3.30953	0.24966	-0.71696
C	-2.94387	1.66786	-1.08368	C	4.08939	-0.6912	-1.61036
C	-1.12266	-1.20327	0.78697	C	1.3543	0.27061	0.96683
C	0.11769	-1.228	-0.11074	C	0.47195	1.07472	-0.01053
C	-0.3104	-1.10114	-1.5856	C	1.36253	1.94206	-0.90562
C	-1.13724	0.16766	-1.80185	C	2.38605	1.08243	-1.65048
C	-3.4256	-0.79428	-1.31785	C	4.24072	1.18046	0.06814
C	-3.26792	1.59844	2.19586	C	3.34878	-2.94046	0.71019
C	1.04275	-2.4094	0.10808	C	-0.61442	1.82887	0.73448
C	2.44878	-2.28501	-0.44505	C	-1.79671	1.00476	1.19767
C	3.26085	-1.1367	0.18232	C	-2.7338	0.63897	0.03894
C	3.05868	0.2176	-0.45069	C	-3.90194	-0.21106	0.462
C	3.34181	1.48426	0.37219	C	-4.85828	-0.7794	-0.60068
C	0.67264	-3.52118	0.75397	C	-0.56182	3.14135	0.98751
O	2.71429	0.34997	-1.62192	O	-4.12286	-0.47515	1.64024
C	4.73554	1.41526	1.00657	C	-4.10581	-1.77638	-1.49531
O	3.30617	2.60316	-0.51174	O	-5.9015	-1.47569	0.07956
C	2.24765	1.65761	1.43929	C	-5.48213	0.35415	-1.42458
H	-1.24534	0.92492	0.7798	H	1.80923	-1.25678	-0.44458
H	0.68652	-0.32009	0.12794	H	-0.02478	0.33965	-0.66138
H	-4.40981	3.07399	-1.65252	H	5.85648	-1.48112	-2.44896
H	-4.89894	1.3035	-1.83969	H	6.10362	-0.13922	-1.20495
H	-4.6241	-0.67493	2.7176	H	4.13973	-1.80043	3.02442
H	-3.69319	-1.80789	1.59557	H	3.455	-0.10962	2.73944
H	-2.23735	2.45427	-0.81251	H	3.46231	-1.36038	-2.20214
H	-1.74737	-2.07762	0.5808	H	1.85231	0.97062	1.64581
H	-0.82221	-1.275	1.83794	H	0.73371	-0.37973	1.59155
H	0.56681	-1.06721	-2.23773	H	0.74777	2.49112	-1.62833

H	-0.88153	-1.99374	-1.868	H	1.88057	2.69325	-0.29928
H	-0.49438	1.03514	-1.60108	H	1.84881	0.3927	-2.31515
H	-1.44768	0.24417	-2.85063	H	3.01446	1.71023	-2.2931
H	-4.34771	-0.70784	-0.73874	H	3.68401	1.84551	0.7316
H	-3.67678	-0.68431	-2.37773	H	4.94452	0.61541	0.68314
H	-3.0392	-1.80563	-1.1761	H	4.81688	1.80786	-0.61958
H	-4.08444	1.60946	2.92401	H	3.89811	-3.53438	1.44674
H	-2.35336	1.9409	2.69772	H	2.38076	-3.4259	0.52901
H	-3.49248	2.33591	1.41796	H	3.89783	-2.97415	-0.2369
H	2.98311	-3.22208	-0.26714	H	-1.45105	0.07832	1.67109
H	2.42541	-2.13403	-1.53005	H	-2.36343	1.55521	1.954
H	3.09789	-1.0786	1.26174	H	-3.13595	1.54649	-0.42979
H	4.33338	-1.34186	0.05329	H	-2.19829	0.11404	-0.75959
H	1.36285	-4.35131	0.88352	H	-1.35336	3.63582	1.54564
H	-0.32061	-3.65143	1.17146	H	0.25853	3.76947	0.65518
H	4.93967	2.36428	1.51146	H	-4.8246	-2.25222	-2.16922
H	4.80416	0.61291	1.745	H	-3.34003	-1.28511	-2.10074
H	5.50034	1.25921	0.23976	H	-3.63278	-2.55492	-0.8886
H	2.94234	2.25398	-1.34756	H	-5.66821	-1.42044	1.0262
H	2.21261	0.8157	2.13546	H	-5.97996	1.07678	-0.77054
H	2.45675	2.56847	2.00859	H	-4.73406	0.87697	-2.02564
H	1.26857	1.76518	0.96357	H	-6.22926	-0.07229	-2.10087

atom	Con f. 5- 3s			atom	Con f. 5- 3t		
C	5.70794	-0.78181	0.33802	C	-5.46289	-0.07902	-2.0141
C	3.40755	2.73238	1.21609	C	-4.08767	1.42055	2.00016
C	2.57042	2.07769	0.40211	C	-3.58961	1.62482	0.77479
C	2.38511	0.5705	0.53671	C	-2.61668	0.66361	0.10505
C	3.30394	-0.30475	-0.40112	C	-3.32498	-0.50749	-0.67766
C	4.75471	0.03103	-0.12075	C	-4.15809	0.08704	-1.79235
C	0.90144	0.18222	0.40452	C	-1.54831	0.13231	1.07749
C	0.65041	-1.31162	0.68088	C	-0.47308	-0.68754	0.35529
C	1.53231	-2.1678	-0.24225	C	-1.13471	-1.85773	-0.40097
C	3.00623	-1.78774	-0.0899	C	-2.21365	-1.35266	-1.36094
C	3.06636	-0.01846	-1.89969	C	-4.16805	-1.39481	0.24522
C	1.75434	2.8283	-0.6227	C	-3.94745	2.86113	-0.00954
C	-0.82503	-1.64709	0.5809	C	0.64862	-1.18751	1.24634
C	-1.43279	-1.66182	-0.81157	C	1.97184	-1.4327	0.55499
C	-2.90417	-1.2514	-0.87517	C	2.74852	-0.1254	0.34635
C	-3.13266	0.18321	-0.46733	C	4.03237	-0.3047	-0.41866
C	-4.51701	0.62304	0.02918	C	5.09165	0.80871	-0.39338
C	-1.53717	-1.91009	1.68243	C	0.51253	-1.41139	2.55792
O	-2.24218	1.0262	-0.52488	O	4.273	-1.32194	-1.06195
C	-4.73777	0.04018	1.43647	C	5.75495	0.81949	0.99567
O	-4.52703	2.04733	0.10753	O	6.07711	0.50256	-1.37857
C	-5.62059	0.18815	-0.93891	C	4.48108	2.17033	-0.73375
H	2.70125	0.30635	1.55386	H	-2.08314	1.24023	-0.66363
H	0.96529	-1.50955	1.7143	H	-0.02152	-0.03541	-0.40748
H	6.72275	-0.41984	0.48426	H	-5.94858	0.39086	-2.86557
H	5.53102	-1.82441	0.58465	H	-6.09687	-0.67868	-1.36781
H	3.55181	3.80784	1.14218	H	-4.7771	2.13017	2.45127
H	3.97875	2.21447	1.98252	H	-3.83169	0.5508	2.59708
H	5.02365	1.06219	-0.34413	H	-3.58913	0.70576	-2.48866
H	0.53412	0.43539	-0.59373	H	-2.01861	-0.48089	1.85169
H	0.31361	0.78065	1.1097	H	-1.08418	0.98076	1.59228
H	1.39991	-3.22952	-0.00221	H	-0.38207	-2.41908	-0.96594
H	1.21797	-2.04026	-1.28393	H	-1.56121	-2.55424	0.33045
H	3.31429	-1.99848	0.94199	H	-1.73353	-0.73663	-2.13283
H	3.62405	-2.41994	-0.73825	H	-2.68342	-2.19607	-1.88075
H	3.42804	0.97584	-2.17586	H	-4.9936	-0.83607	0.69156
H	3.61485	-0.74665	-2.5072	H	-4.58915	-2.23393	-0.31783
H	2.01092	-0.08024	-2.17574	H	-3.57195	-1.81088	1.05991
H	1.86157	2.40984	-1.62771	H	-4.40447	2.60257	-0.97073
H	0.68489	2.78535	-0.38346	H	-4.64302	3.50256	0.53979
H	2.04726	3.88146	-0.65807	H	-3.04669	3.4459	-0.23823
H	-0.85931	-1.0032	-1.46884	H	1.80887	-1.9041	-0.42022
H	-1.33208	-2.6678	-1.23781	H	2.58662	-2.11962	1.14521
H	-3.26702	-1.3312	-1.90988	H	2.14822	0.59803	-0.22292
H	-3.54632	-1.91394	-0.29008	H	2.95858	0.36084	1.30441
H	-2.59786	-2.13903	1.65782	H	1.33946	-1.79986	3.1479
H	-1.06819	-1.91011	2.66334	H	-0.4135	-1.22203	3.09178
H	-5.68029	0.43256	1.8302	H	6.56818	1.5514	0.98767
H	-4.79577	-1.05082	1.42366	H	5.05033	1.09384	1.78504
H	-3.9273	0.34233	2.10668	H	6.17574	-0.16467	1.22456

H	-3.58997	2.31059	0.0361	H	5.91492	-0.42955	-1.61779
H	-5.69807	-0.90016	-0.99905	H	3.75651	2.48596	0.02074
H	-6.57849	0.58015	-0.58369	H	5.27997	2.917	-0.77492
H	-5.43313	0.58721	-1.94033	H	3.98678	2.13796	-1.70944

atom	Con f. 5- 4a			atom	Con f. 5- 4b		
C	-3.76228	-1.7856	2.17263	C	-5.50759	-0.96526	-0.22359
C	-3.13218	-1.51929	-2.25413	C	-3.0628	2.63677	0.94842
C	-2.24443	-1.5197	-1.2525	C	-2.98021	1.83483	-0.12025
C	-1.85228	-0.2603	-0.49468	C	-2.20282	0.52609	-0.11551
C	-2.80631	0.08458	0.71101	C	-3.03869	-0.70755	0.39979
C	-2.74938	-1.05113	1.7099	C	-4.21095	-0.92101	-0.53367
C	-1.67053	0.95706	-1.41738	C	-0.86032	0.63935	0.62767
C	-1.00876	2.14003	-0.67821	C	0.01975	-0.61378	0.43981
C	-1.91989	2.51959	0.50214	C	-0.76717	-1.83581	0.94135
C	-2.21926	1.33379	1.42977	C	-2.13958	-1.97044	0.2712
C	-4.24391	0.36262	0.25817	C	-3.4998	-0.53265	1.85078
C	-1.53362	-2.78321	-0.84128	C	-3.63469	2.21206	-1.424
C	0.45297	1.87392	-0.31359	C	0.57771	-0.74036	-0.97566
C	1.31612	1.36851	-1.455	C	1.21028	0.50026	-1.5766
C	2.74327	0.95729	-1.10475	C	2.36899	1.06864	-0.74203
C	2.82326	-0.309	-0.28842	C	3.54944	0.1355	-0.64556
C	4.13709	-0.67731	0.41843	C	4.49529	0.22634	0.55975
C	0.96408	2.13139	0.89547	C	0.58861	-1.88267	-1.67213
O	1.87385	-1.07969	-0.1802	O	3.78514	-0.70333	-1.50988
C	4.32302	0.24905	1.63329	C	3.76872	-0.34085	1.79271
O	4.02951	-2.02497	0.87401	O	5.64099	-0.57774	0.28505
C	5.32836	-0.59109	-0.53909	C	4.96272	1.66534	0.79273
H	-0.87363	-0.46084	-0.03995	H	-1.96577	0.29557	-1.16335
H	-0.99171	2.98608	-1.38188	H	0.89148	-0.48882	1.09669
H	-3.59298	-2.57263	2.90319	H	-6.25835	-1.14252	-0.98957
H	-4.78893	-1.64072	1.84962	H	-5.87569	-0.82779	0.78888
H	-3.38288	-2.43474	-2.78489	H	-3.62108	3.56917	0.90958
H	-3.63646	-0.61605	-2.58356	H	-2.58343	2.40127	1.89358
H	-1.74523	-1.2604	2.08292	H	-3.92309	-1.06925	-1.57597
H	-1.0919	0.66176	-2.29501	H	-0.34123	1.53926	0.28843
H	-2.63862	1.29402	-1.7999	H	-1.03022	0.7764	1.69966
H	-1.48705	3.34054	1.08375	H	-0.19255	-2.75637	0.79451
H	-2.85811	2.90538	0.08737	H	-0.89702	-1.73018	2.02429
H	-1.29818	1.03083	1.93749	H	-2.00209	-2.1845	-0.79378
H	-2.91948	1.64527	2.21411	H	-2.67903	-2.82592	0.69481
H	-4.6889	-0.51185	-0.22219	H	-4.03291	-1.42748	2.18762
H	-4.86491	0.6311	1.11877	H	-2.65692	-0.37999	2.5279
H	-4.29211	1.19189	-0.45075	H	-4.17027	0.32297	1.95816
H	-0.44769	-2.66235	-0.94722	H	-2.88356	2.29996	-2.22014
H	-1.71952	-3.01538	0.21303	H	-4.34386	1.4428	-1.7475
H	-1.84763	-3.64161	-1.44269	H	-4.16964	3.1635	-1.34946
H	0.83296	0.50721	-1.923	H	1.57578	0.26897	-2.58036
H	1.35056	2.14246	-2.23362	H	0.46024	1.29105	-1.6879
H	3.29234	0.74185	-2.03214	H	2.75036	1.98338	-1.21789
H	3.30855	1.75505	-0.61492	H	2.04457	1.36879	0.25685
H	2.01419	1.9858	1.12294	H	1.0491	-1.93068	-2.6554
H	0.36249	2.51788	1.70947	H	0.1553	-2.80488	-1.30112
H	5.21856	-0.06664	2.17711	H	4.47173	-0.36202	2.63098
H	4.44925	1.29409	1.33845	H	2.9077	0.2683	2.07917
H	3.4639	0.17221	2.30648	H	3.4304	-1.36323	1.59779
H	3.07904	-2.23769	0.81272	H	5.3919	-1.11463	-0.49109
H	5.50067	0.43392	-0.8758	H	4.12968	2.32291	1.05224
H	6.22673	-0.9364	-0.01847	H	5.67897	1.67685	1.62001
H	5.16771	-1.23009	-1.41271	H	5.45911	2.05679	-0.10034

atom	Con f. 5- 4c			atom	Con f. 5- 4d		
C	5.06548	0.64927	1.98768	C	-5.57193	0.46939	-0.67958
C	2.99875	2.45053	-1.58215	C	-2.12028	2.79208	1.01172
C	2.50003	1.98256	-0.43159	C	-2.19532	2.02828	-0.08484
C	2.1823	0.51123	-0.20648	C	-2.00259	0.51888	-0.05473
C	3.41381	-0.33448	0.29934	C	-3.32001	-0.28297	0.27275
C	3.85492	0.20899	1.64099	C	-4.33461	-0.01	-0.81638
C	1.52231	-0.14535	-1.43104	C	-0.84628	0.08917	0.86442
C	0.99415	-1.56206	-1.11982	C	-0.51054	-1.4096	0.71604
C	2.17566	-2.41626	-0.63291	C	-1.77552	-2.22139	1.03857
C	2.91232	-1.7847	0.555	C	-2.98011	-1.79887	0.18897
C	4.56797	-0.345	-0.70925	C	-3.87901	0.05633	1.65937
C	2.18575	2.91157	0.71249	C	-2.44279	2.63863	-1.44027

C	-0.23001	-1.55356	-0.20422	C	0.14245	-1.73748	-0.62557
C	-1.31633	-0.58242	-0.61942	C	1.2524	-0.81802	-1.09197
C	-2.65735	-0.74327	0.08873	C	2.42939	-0.74729	-0.11287
C	-3.72039	0.185	-0.43585	C	3.52106	0.1847	-0.56606
C	-5.00939	0.39164	0.3762	C	4.74827	0.41932	0.3325
C	-0.36951	-2.37011	0.84687	C	-0.18212	-2.79982	-1.37189
O	-3.59574	0.79816	-1.49187	O	3.46466	0.78228	-1.63685
C	-4.67823	1.27074	1.59587	C	5.46364	-0.90372	0.63015
O	-5.94705	1.07818	-0.45172	O	5.64934	1.27017	-0.37408
C	-5.6319	-0.94128	0.79691	C	4.31123	1.12607	1.62552
H	1.44943	0.46775	0.61108	H	-1.72664	0.21511	-1.07387
H	0.64016	-1.98176	-2.07368	H	0.23972	-1.64405	1.48505
H	5.26271	1.00348	2.99632	H	-6.2157	0.60804	-1.5445
H	5.90092	0.67884	1.29453	H	-5.99268	0.74342	0.28327
H	3.20885	3.50916	-1.71502	H	-2.25561	3.86965	0.95757
H	3.21299	1.80581	-2.4288	H	-1.92175	2.37889	1.99567
H	3.06724	0.21194	2.39644	H	-3.98477	-0.26131	-1.8192
H	0.72102	0.50139	-1.79594	H	0.02683	0.71125	0.65191
H	2.24113	-0.22436	-2.25212	H	-1.10241	0.27884	1.91101
H	1.8453	-3.42607	-0.36741	H	-1.59329	-3.29383	0.91326
H	2.86979	-2.5372	-1.47223	H	-2.006	-2.07705	2.10008
H	2.2464	-1.76679	1.4238	H	-2.78733	-2.0484	-0.85962
H	3.77201	-2.40599	0.83308	H	-3.86623	-2.37095	0.48845
H	5.3812	-0.98073	-0.3446	H	-4.7626	-0.55476	1.86987
H	4.25585	-0.73797	-1.67911	H	-3.15031	-0.13903	2.449
H	4.96821	0.65834	-0.87171	H	-4.16843	1.10733	1.72941
H	2.73403	2.62577	1.61658	H	-2.56181	3.72462	-1.38101
H	2.43528	3.94962	0.47329	H	-1.60751	2.4196	-2.11851
H	1.11818	2.86303	0.96488	H	-3.34142	2.21975	-1.90611
H	-0.96246	0.44412	-0.46269	H	1.61939	-1.15795	-2.06445
H	-1.4695	-0.66825	-1.70221	H	0.86462	0.19555	-1.24523
H	-3.04423	-1.7643	-0.03589	H	2.10825	-0.42772	0.88449
H	-2.56431	-0.59763	1.16939	H	2.87032	-1.74178	0.03113
H	-1.26861	-2.38105	1.45402	H	0.34502	-3.01034	-2.29928
H	0.40318	-3.07006	1.14345	H	-0.96614	-3.49817	-1.09948
H	-5.60823	1.49446	2.12711	H	6.38445	-0.68968	1.18127
H	-3.99515	0.77236	2.28878	H	4.84645	-1.56938	1.23846
H	-4.22705	2.21432	1.27309	H	5.7278	-1.41625	-0.30003
H	-5.41939	1.43394	-1.19138	H	5.17166	1.52421	-1.18702
H	-4.98419	-1.48733	1.48693	H	3.68163	0.48612	2.2488
H	-6.58236	-0.74527	1.30255	H	5.20549	1.39146	2.19735
H	-5.82824	-1.56846	-0.07793	H	3.76305	2.04501	1.3947

atom	Con f. 5- 4e			atom	Con f. 5- 4f		
C	5.28161	-0.92552	1.65649	C	-5.32148	-1.54579	-0.38227
C	3.91812	2.21638	-1.23931	C	-3.61318	2.25935	1.42034
C	3.34525	1.76747	-0.11586	C	-3.43356	1.6915	0.22166
C	2.45427	0.53437	-0.07704	C	-2.40838	0.59568	-0.0355
C	3.2472	-0.81585	0.10928	C	-2.94375	-0.85661	0.26344
C	3.9754	-0.77015	1.43486	C	-4.08238	-1.15665	-0.68697
C	1.50023	0.45716	-1.28137	C	-1.06598	0.86788	0.673
C	0.44999	-0.66479	-1.1298	C	0.03367	-0.14508	0.3142
C	1.18478	-1.99941	-0.92934	C	-0.48666	-1.56247	0.63317
C	2.19903	-1.95915	0.2209	C	-1.80263	-1.85365	-0.08902
C	4.20949	-1.09167	-1.05125	C	-3.36956	-1.0336	1.72503
C	3.52649	2.49684	1.19053	C	-4.2338	2.1399	-0.9742
C	-0.61501	-0.33154	-0.09039	C	0.59078	-0.01372	-1.10116
C	-1.26031	1.03193	-0.27276	C	1.56258	-1.08461	-1.55859
C	-2.66614	1.18337	0.30351	C	2.79112	-1.23682	-0.64827
C	-3.68513	0.30694	-0.38116	C	3.66923	-0.01275	-0.6135
C	-5.03147	0.02699	0.30267	C	4.56822	0.24192	0.60435
C	-0.99381	-1.17039	0.88023	C	0.31844	1.0118	-1.91663
O	-3.47918	-0.18966	-1.48433	O	3.69643	0.79511	-1.5369
C	-5.69044	1.31896	0.79154	C	5.41857	-0.98929	0.92994
O	-5.89696	-0.57808	-0.65686	O	5.44381	1.32221	0.28819
C	-4.79491	-0.96089	1.45878	C	3.67956	0.64733	1.79374
H	1.82721	0.62573	0.82067	H	-2.20913	0.60149	-1.1126
H	-0.09529	-0.7166	-2.08453	H	0.88156	0.04441	0.9869
H	5.68493	-0.88556	2.66518	H	-6.04943	-1.75184	-1.16287
H	5.99734	-1.09442	0.85746	H	-5.66299	-1.67276	0.64076
H	4.54471	3.10508	-1.23433	H	-4.35148	3.04442	1.56547
H	3.78691	1.72382	-2.19771	H	-3.03959	1.96998	2.29526
H	3.32134	-0.60306	2.2924	H	-3.81696	-1.05291	-1.74056
H	1.01703	1.42811	-1.4142	H	-0.74109	1.88699	0.44144

H	2.0645	0.27265	-2.20022	H	-1.20524	0.83699	1.75808
H	0.47081	-2.8134	-0.76329	H	0.25331	-2.32373	0.36992
H	1.70451	-2.23869	-1.86408	H	-0.62834	-1.63944	1.71677
H	1.66691	-1.84089	1.17042	H	-1.62889	-1.81628	-1.17249
H	2.73168	-2.91591	0.27937	H	-2.14352	-2.87047	0.1388
H	4.97928	-0.32021	-1.12665	H	-4.21694	-0.39056	1.97295
H	4.70729	-2.05597	-0.90673	H	-3.66266	-2.07173	1.91259
H	3.68746	-1.13361	-2.00947	H	-2.55917	-0.79098	2.41541
H	2.55647	2.83003	1.58279	H	-4.94506	2.92979	-0.71473
H	3.96379	1.84314	1.95288	H	-3.56996	2.51911	-1.76235
H	4.17189	3.37331	1.07963	H	-4.79173	1.30494	-1.41158
H	-0.61739	1.80084	0.17308	H	1.06025	-2.05727	-1.60394
H	-1.29872	1.26073	-1.34313	H	1.89853	-0.85567	-2.57316
H	-2.69797	1.02077	1.38399	H	2.51514	-1.52646	0.36822
H	-3.01239	2.2163	0.15785	H	3.4274	-2.0503	-1.02502
H	-1.78361	-0.92241	1.58165	H	0.7883	1.08546	-2.89389
H	-0.53646	-2.1438	1.01508	H	-0.36311	1.81266	-1.65239
H	-6.67576	1.08107	1.204	H	6.09074	-0.74703	1.7588
H	-5.0996	1.80146	1.57386	H	4.80003	-1.83945	1.22809
H	-5.8225	2.02071	-0.03753	H	6.02446	-1.2767	0.06532
H	-5.31153	-0.85292	-1.38762	H	5.08362	1.70405	-0.53499
H	-4.1707	-0.53038	2.24579	H	4.32522	0.91144	2.63681
H	-5.76367	-1.22751	1.89196	H	3.0729	1.52077	1.53522
H	-4.31708	-1.87272	1.08795	H	3.01715	-0.16346	2.10677

atom	Con f. 5- 4g			atom	Con f. 5- 4h		
C	3.466	-1.98156	-1.98279	C	-4.78992	-1.94088	-0.63217
C	2.60916	-1.49068	2.38822	C	-3.57307	2.02846	-1.72074
C	1.77772	-1.47165	1.3395	C	-2.94945	1.86603	-0.54725
C	1.52253	-0.22078	0.51	C	-2.23872	0.5569	-0.23136
C	2.57229	0.00561	-0.64191	C	-3.08017	-0.48769	0.60134
C	2.48578	-1.16084	-1.60216	C	-4.37338	-0.77504	-0.13497
C	1.35707	1.03503	1.38178	C	-0.8616	0.80203	0.4121
C	0.90233	2.27104	0.57636	C	-0.02218	-0.48714	0.51946
C	1.90415	2.50895	-0.56465	C	-0.82693	-1.52222	1.32155
C	2.12314	1.26515	-1.43456	C	-2.22313	-1.76469	0.73652
C	3.99635	0.19287	-0.10778	C	-3.47661	0.03558	1.99862
C	1.00469	-2.70258	0.94222	C	-2.89243	2.9957	0.45208
C	-0.56463	2.22118	0.1618	C	0.49542	-0.9709	-0.83184
C	-1.5649	1.9577	1.27844	C	1.17192	0.06246	-1.71364
C	-2.09984	0.51323	1.2915	C	2.33586	0.79918	-1.03298
C	-3.1408	0.25412	0.22762	C	3.47718	-0.10374	-0.64158
C	-3.22661	-1.12282	-0.44296	C	4.43129	0.32271	0.48372
C	-1.00013	2.45463	-1.0826	C	0.43561	-2.24296	-1.2417
O	-3.9432	1.11756	-0.1153	O	3.67839	-1.17772	-1.20043
C	-3.30139	-2.24138	0.60068	C	3.68579	0.22605	1.82665
O	-4.41592	-1.16058	-1.22874	O	5.5311	-0.58542	0.49844
C	-2.00709	-1.29028	-1.36622	C	4.97399	1.73411	0.24173
H	0.56807	-0.37104	-0.00771	H	-2.06316	0.05997	-1.1937
H	0.9667	3.12694	1.26594	H	0.87073	-0.23386	1.10745
H	3.27972	-2.7811	-2.69539	H	-5.75343	-2.01977	-1.12982
H	4.48136	-1.89813	-1.60663	H	-4.20695	-2.85497	-0.57005
H	2.76566	-2.39918	2.96494	H	-4.08063	2.95622	-1.97475
H	3.15908	-0.61265	2.71203	H	-3.6	1.23673	-2.46529
H	1.49113	-1.31049	-2.02616	H	-5.02621	0.09007	-0.24089
H	0.65191	0.81606	2.18819	H	-0.32973	1.56566	-0.16215
H	2.30456	1.28151	1.87034	H	-0.98431	1.21379	1.41865
H	1.58364	3.34684	-1.19291	H	-0.28226	-2.47005	1.38644
H	2.85742	2.81334	-0.11786	H	-0.91618	-1.15449	2.35023
H	1.19442	1.02152	-1.96118	H	-2.11813	-2.21576	-0.25447
H	2.87293	1.47757	-2.20597	H	-2.76071	-2.49108	1.35744
H	4.35252	-0.70336	0.40511	H	-4.20889	0.84443	1.92948
H	4.68388	0.40911	-0.93189	H	-3.93687	-0.77334	2.57642
H	4.05771	1.024	0.59796	H	-2.61895	0.40408	2.56701
H	1.25603	-3.56053	1.57305	H	-3.2678	2.70082	1.43639
H	-0.07472	-2.52136	1.02416	H	-1.86132	3.33627	0.60331
H	1.19744	-2.97427	-0.10108	H	-3.47985	3.85091	0.10617
H	-1.10725	2.16705	2.24905	H	1.54016	-0.42434	-2.62047
H	-2.41907	2.63351	1.17763	H	0.44483	0.81736	-2.03365
H	-1.29448	-0.21886	1.22135	H	2.7615	1.53382	-1.73121
H	-2.61266	0.31608	2.24448	H	2.0052	1.3769	-0.16699
H	-2.06193	2.48221	-1.30858	H	0.87214	-2.54326	-2.19074
H	-0.32972	2.64125	-1.91407	H	-0.03544	-3.0292	-0.66213
H	-3.4167	-3.20017	0.08614	H	4.39248	0.44541	2.63285

H	-2.39447	-2.28663	1.20811	H	2.85774	0.93619	1.89175
H	-4.16325	-2.09513	1.2588	H	3.29701	-0.78625	1.97367
H	-4.72994	-0.23674	-1.25492	H	5.25983	-1.31852	-0.08636
H	-1.94885	-0.46429	-2.08147	H	4.17775	2.48196	0.26782
H	-1.07367	-1.33403	-0.80285	H	5.69735	1.97604	1.02642
H	-2.11951	-2.22655	-1.92119	H	5.48138	1.78974	-0.72612

atom	Con f. 5- 4i			atom	Con f. 5- 4j		
C	2.23314	0.9789	3.09832	C	-1.75157	-1.44834	2.85437
C	1.53306	2.83075	-0.75839	C	-0.98983	-2.69286	-1.05125
C	2.23801	1.73476	-1.06683	C	-1.71525	-1.59686	-1.31059
C	1.88231	0.39935	-0.43032	C	-1.51967	-0.33321	-0.48408
C	2.76197	-0.03489	0.80607	C	-2.48373	-0.17446	0.75417
C	2.70168	1.06021	1.85197	C	-2.31443	-1.38938	1.64584
C	1.80626	-0.727	-1.47749	C	-1.50776	0.9254	-1.36805
C	1.14615	-1.99499	-0.89986	C	-1.08743	2.19502	-0.60007
C	2.00744	-2.45519	0.28977	C	-2.03342	2.36386	0.59949
C	2.17281	-1.3565	1.34765	C	-2.06896	1.11735	1.49089
C	4.25329	-0.22594	0.4544	C	-3.9707	-0.11127	0.35175
C	3.33747	1.79291	-2.09882	C	-2.69724	-1.57841	-2.45593
C	-0.34051	-1.80818	-0.58337	C	0.40197	2.24918	-0.26864
C	-1.15577	-1.13147	-1.6701	C	1.36122	1.96881	-1.41598
C	-2.60286	-0.79141	-1.32398	C	1.92237	0.53363	-1.40232
C	-2.73297	0.32708	-0.31849	C	2.99804	0.32123	-0.36324
C	-4.07651	0.55985	0.39063	C	3.11335	-1.02571	0.36411
C	-0.9075	-2.26218	0.54	C	0.88877	2.5842	0.93291
O	-1.80276	1.08404	-0.05696	O	3.80499	1.2021	-0.08052
C	-4.2879	-0.5531	1.43172	C	3.06687	-2.20426	-0.61095
O	-4.0092	1.81662	1.06222	O	4.37042	-1.05763	1.03843
C	-5.23179	0.61495	-0.61288	C	1.98051	-1.10514	1.40382
H	0.87462	0.52045	-0.01716	H	-0.5283	-0.41314	-0.03055
H	1.19479	-2.76797	-1.68174	H	-1.25228	3.03919	-1.28744
H	2.25864	1.84256	3.7584	H	-1.68868	-2.39191	3.39081
H	1.81115	0.06958	3.51568	H	-1.33783	-0.58155	3.36062
H	1.7507	3.79617	-1.20978	H	-1.10724	-3.6049	-1.63207
H	0.71236	2.79672	-0.04631	H	-0.26504	-2.71897	-0.24243
H	3.10902	2.01391	1.51899	H	-2.70232	-2.31243	1.21755
H	1.27066	-0.37085	-2.36085	H	-0.8469	0.75394	-2.22284
H	2.80945	-0.99354	-1.82451	H	-2.50325	1.09978	-1.78847
H	1.59074	-3.3551	0.75405	H	-1.75249	3.23805	1.19613
H	2.99004	-2.74428	-0.1005	H	-3.03742	2.57128	0.21198
H	1.19463	-1.14201	1.78618	H	-1.07346	0.96647	1.91668
H	2.80939	-1.72278	2.16173	H	-2.75113	1.28141	2.33345
H	4.73919	0.72833	0.2345	H	-4.31492	-1.06847	-0.04964
H	4.7773	-0.66822	1.30879	H	-4.58422	0.11485	1.23095
H	4.40165	-0.88527	-0.40479	H	-4.16769	0.65839	-0.39873
H	4.2912	1.4194	-1.71511	H	-2.78957	-2.57023	-2.90745
H	3.09111	1.17412	-2.96978	H	-3.69295	-1.25136	-2.14175
H	3.48963	2.81776	-2.44923	H	-2.37529	-0.88196	-3.23936
H	-0.66377	-0.20149	-1.9656	H	0.86256	2.13769	-2.37445
H	-1.1426	-1.76704	-2.56585	H	2.20559	2.66235	-1.37024
H	-3.11381	-0.43208	-2.22828	H	1.13442	-0.2107	-1.29019
H	-3.17837	-1.66093	-0.99451	H	2.41202	0.31825	-2.36366
H	-1.97027	-2.16608	0.73168	H	1.9582	2.68191	1.09419
H	-0.33975	-2.76274	1.31568	H	0.25566	2.78542	1.78993
H	-5.20711	-0.34049	1.98602	H	3.21837	-3.13227	-0.0511
H	-4.385	-1.53738	0.96628	H	3.86203	-2.11766	-1.35769
H	-3.45351	-0.57541	2.13907	H	2.10405	-2.26981	-1.12279
H	-3.0635	2.05732	1.05517	H	4.66829	-0.12874	1.06035
H	-5.37239	-0.344	-1.11745	H	2.01757	-0.24579	2.08021
H	-6.15446	0.85951	-0.07788	H	2.1142	-2.01877	1.99104
H	-5.05273	1.38921	-1.3651	H	0.99617	-1.13888	0.93604

atom	Con f. 5- 4k			atom	Con f. 5- 4l		
C	4.02349	-0.81933	2.6939	C	4.64918	-1.76384	2.05717
C	2.24107	2.76543	1.19837	C	4.97776	1.63719	-0.89838
C	2.51034	2.11478	0.05975	C	4.10784	1.4272	0.09691
C	2.2319	0.62196	-0.05488	C	2.85724	0.57191	-0.05822
C	3.46247	-0.32065	0.24508	C	3.10828	-0.96595	0.17731
C	3.98885	-0.01179	1.63251	C	3.54191	-1.16693	1.61318
C	1.57477	0.26996	-1.4017	C	2.12889	0.83711	-1.39195
C	1.05714	-1.18237	-1.43898	C	0.78736	0.09575	-1.51728
C	2.24743	-2.11495	-1.16143	C	1.04894	-1.41213	-1.31407
C	2.96026	-1.77802	0.15356	C	1.73912	-1.68779	0.02215

C	4.63906	-0.10194	-0.73022	C	4.1289	-1.55151	-0.80504
C	3.03633	2.85972	-1.14276	C	4.30826	2.06398	1.448
C	-0.16752	-1.40363	-0.54968	C	-0.3407	0.63814	-0.63886
C	-1.24686	-0.34967	-0.68905	C	-1.60564	-0.19572	-0.65589
C	-2.58144	-0.67486	-0.02777	C	-2.838	0.45617	-0.03862
C	-3.61918	0.39605	-0.23211	C	-4.07252	-0.40005	-0.13368
C	-5.02539	0.21614	0.36746	C	-5.40578	0.12034	0.4338
C	-0.31077	-2.4676	0.24927	C	-0.25453	1.78608	0.04413
O	-3.38606	1.42394	-0.86166	O	-4.05982	-1.51406	-0.64983
C	-4.93869	0.10782	1.8966	C	-5.84129	1.38788	-0.31566
O	-5.79265	1.37227	0.03593	O	-6.39096	-0.89063	0.22804
C	-5.70823	-1.01552	-0.24393	C	-5.27721	0.37674	1.9418
H	1.5063	0.3809	0.73189	H	2.17398	0.86769	0.74512
H	0.70858	-1.36646	-2.46657	H	0.43242	0.22148	-2.55149
H	4.44127	-0.47758	3.63777	H	4.84148	-1.86255	3.12254
H	3.64854	-1.83837	2.67712	H	5.40277	-2.17339	1.39121
H	2.42322	3.83245	1.30392	H	5.85876	2.25787	-0.75331
H	1.83179	2.25154	2.06457	H	4.84837	1.20638	-1.88617
H	4.39082	0.99442	1.74153	H	2.83173	-0.78133	2.34687
H	0.76655	0.97702	-1.6044	H	1.98827	1.91577	-1.51318
H	2.2945	0.39509	-2.21679	H	2.76156	0.52228	-2.22745
H	1.93007	-3.163	-1.15203	H	0.12112	-1.98672	-1.37634
H	2.95012	-2.02065	-1.99739	H	1.67709	-1.76391	-2.1395
H	2.27163	-1.95816	0.98395	H	1.07937	-1.36306	0.8375
H	3.80912	-2.45697	0.29709	H	1.89374	-2.76508	0.15478
H	5.10844	0.87401	-0.57945	H	4.21454	-2.63386	-0.66358
H	5.40706	-0.86363	-0.55635	H	3.83912	-1.37731	-1.84337
H	4.33424	-0.17065	-1.77747	H	5.11791	-1.11096	-0.66202
H	2.30892	2.84491	-1.96302	H	3.46032	2.71586	1.69661
H	3.2394	3.90551	-0.89518	H	4.36115	1.30616	2.2372
H	3.95588	2.41811	-1.53785	H	5.22367	2.66226	1.48403
H	-0.87951	0.59911	-0.28043	H	-1.82809	-0.47507	-1.69379
H	-1.41144	-0.15382	-1.75654	H	-1.41932	-1.14538	-0.14043
H	-2.99102	-1.62195	-0.39895	H	-2.67675	0.68321	1.02182
H	-2.46418	-0.81433	1.05353	H	-3.05556	1.42145	-0.51128
H	-1.20915	-2.63412	0.83419	H	-1.08633	2.18043	0.6183
H	0.45933	-3.22384	0.34774	H	0.64721	2.38747	0.05453
H	-5.95364	0.09286	2.30516	H	-6.84171	1.67041	0.02593
H	-4.42801	-0.80572	2.21128	H	-5.16476	2.2251	-0.12693
H	-4.41046	0.97089	2.31397	H	-5.88375	1.20193	-1.39345
H	-5.18711	1.93799	-0.48084	H	-5.92033	-1.61157	-0.23289
H	-5.20719	-1.94335	0.04319	H	-4.59577	1.20341	2.15745
H	-6.74129	-1.05714	0.1143	H	-6.26426	0.6323	2.33901
H	-5.7233	-0.94395	-1.33601	H	-4.91987	-0.52105	2.45587

atom	Con f. 5- 4m			atom	Con f. 5- 4n		
C	-4.99845	-0.73904	-1.63044	C	4.81895	2.29583	-1.05481
C	-2.40655	2.67107	-1.43838	C	4.66322	-1.39771	1.58673
C	-2.22108	2.13305	-0.22666	C	4.19596	-1.17365	0.35259
C	-2.06599	0.62604	-0.07044	C	2.84235	-0.53219	0.07884
C	-3.39248	-0.1567	0.27716	C	2.88085	1.04343	0.0502
C	-4.42405	0.12892	-0.79589	C	3.76135	1.48401	-1.09903
C	-0.93504	0.27407	0.91294	C	1.74075	-1.06192	1.01959
C	-0.60061	-1.23092	0.90808	C	0.3361	-0.54152	0.66998
C	-1.88094	-2.00264	1.26498	C	0.3761	1.00136	0.67227
C	-3.04589	-1.66107	0.32873	C	1.44375	1.53764	-0.28196
C	-4.01856	0.28856	1.61661	C	3.34978	1.64157	1.38085
C	-2.10292	3.01366	0.9934	C	4.98915	-1.5843	-0.8612
C	0.08578	-1.68102	-0.38086	C	-0.26471	-1.12404	-0.60803
C	1.19176	-0.79545	-0.9147	C	-1.54733	-0.49661	-1.11502
C	2.35665	-0.6254	0.06933	C	-2.68914	-0.55275	-0.09407
C	3.41409	0.3245	-0.4264	C	-3.95696	0.09676	-0.58016
C	4.83302	0.25005	0.15632	C	-5.17988	0.17245	0.35151
C	-0.20703	-2.81975	-1.0197	C	0.25006	-2.17325	-1.2604
O	3.18324	1.16302	-1.29261	O	-4.05244	0.58291	-1.70335
C	5.52064	-1.00498	-0.41153	C	-4.84268	1.01411	1.59118
O	5.54788	1.40842	-0.26994	O	-6.23382	0.81619	-0.36272
C	4.8118	0.23728	1.68667	C	-5.6428	-1.23786	0.74112
H	-1.77123	0.24179	-1.05481	H	2.55911	-0.82244	-0.93883
H	0.12816	-1.39677	1.71492	H	-0.33634	-0.84651	1.48492
H	-5.74099	-0.40684	-2.35192	H	5.36163	2.55525	-1.96032
H	-4.76548	-1.79961	-1.63672	H	5.18778	2.73068	-0.13058
H	-2.51216	3.74461	-1.57705	H	5.63252	-1.86485	1.7443
H	-2.4608	2.05516	-2.33255	H	4.10667	-1.12915	2.47915

H	-4.72163	1.17442	-0.85909	H	3.45259	1.08852	-2.06834
H	-0.05092	0.87128	0.67325	H	1.76087	-2.15607	1.01136
H	-1.21983	0.55586	1.93148	H	1.95779	-0.76664	2.05077
H	-1.7023	-3.08283	1.25528	H	-0.59745	1.42514	0.4097
H	-2.14919	-1.74968	2.29716	H	0.58472	1.33682	1.69387
H	-2.79507	-1.99391	-0.6828	H	1.19524	1.22822	-1.3056
H	-3.93855	-2.22106	0.63165	H	1.44068	2.63397	-0.27701
H	-4.40486	1.30984	1.55786	H	3.29161	2.73443	1.3476
H	-4.86164	-0.36598	1.86335	H	2.73232	1.30473	2.21616
H	-3.30896	0.24375	2.44671	H	4.38266	1.36288	1.60083
H	-2.78307	2.70966	1.794	H	5.19537	-0.72426	-1.5073
H	-1.09092	2.96846	1.413	H	5.94337	-2.04413	-0.58712
H	-2.31382	4.05721	0.74305	H	4.42154	-2.30239	-1.46769
H	1.57852	-1.21959	-1.8465	H	-1.37419	0.55038	-1.38922
H	0.79881	0.19553	-1.1648	H	-1.8616	-1.00678	-2.0298
H	2.00822	-0.20969	1.02483	H	-2.91825	-1.59285	0.16991
H	2.808	-1.59096	0.31688	H	-2.41151	-0.07015	0.84914
H	0.34245	-3.11629	-1.9098	H	-0.24337	-2.57891	-2.14024
H	-0.98775	-3.4977	-0.69202	H	1.16337	-2.67038	-0.95274
H	6.55644	-1.02511	-0.05958	H	-5.75547	1.15205	2.17853
H	5.02714	-1.92558	-0.08916	H	-4.09702	0.5264	2.22405
H	5.52572	-0.97217	-1.50553	H	-4.46839	1.99974	1.29699
H	5.0157	1.78029	-0.99847	H	-5.85306	1.03255	-1.23549
H	4.32058	-0.65858	2.07431	H	-4.90226	-1.75168	1.35902
H	5.84157	0.25059	2.05644	H	-6.57163	-1.15625	1.31366
H	4.29408	1.12105	2.07162	H	-5.83835	-1.83921	-0.15216

atom	Con f. 5- 4o			atom	Con f. 5- 4p		
C	3.73695	1.04717	2.48248	C	5.24172	-0.77377	1.79288
C	1.54415	2.4602	-1.94801	C	4.01002	2.1132	-1.45256
C	1.29971	1.96992	-0.72685	C	3.41859	1.80158	-0.29307
C	1.61345	0.53598	-0.32836	C	2.49201	0.60472	-0.12657
C	3.10636	0.29162	0.11838	C	3.24696	-0.74063	0.19196
C	3.47204	1.29036	1.19763	C	3.94659	-0.59769	1.52626
C	1.18748	-0.48374	-1.39863	C	1.51663	0.44869	-1.31252
C	1.2753	-1.9389	-0.88724	C	0.48118	-0.67103	-1.12256
C	2.70144	-2.20533	-0.37684	C	1.22847	-1.98915	-0.8218
C	3.19117	-1.15977	0.63421	C	2.16765	-1.84433	0.37634
C	4.1066	0.5128	-1.0349	C	4.22727	-1.13882	-0.9173
C	0.64427	2.81226	0.33705	C	3.62008	2.65457	0.93283
C	0.15497	-2.28724	0.08519	C	-0.62901	-0.37574	-0.11892
C	-1.26098	-2.03071	-0.40529	C	-1.59542	-1.52752	0.09797
C	-1.8701	-0.75041	0.18131	C	-2.96845	-1.15458	0.65221
C	-3.24506	-0.44325	-0.34768	C	-3.78559	-0.30376	-0.28761
C	-4.10192	0.63133	0.34237	C	-4.99801	0.47376	0.24754
C	0.34263	-2.83361	1.29192	C	-0.78822	0.80447	0.49048
O	-3.7191	-1.02908	-1.31667	O	-3.52302	-0.21928	-1.48325
C	-3.31482	1.9245	0.56312	C	-4.49138	1.66878	1.07457
O	-5.20953	0.92337	-0.50847	O	-5.73631	0.96295	-0.87116
C	-4.62456	0.0557	1.6708	C	-5.91889	-0.429	1.07189
H	1.01824	0.32475	0.56932	H	1.88746	0.80057	0.76542
H	1.10366	-2.58651	-1.76039	H	-0.03824	-0.81026	-2.08286
H	3.99983	1.85656	3.15897	H	5.62433	-0.65908	2.8039
H	3.70711	0.05108	2.91369	H	5.96801	-1.03502	1.02901
H	1.30014	3.48941	-2.20035	H	4.66349	2.97846	-1.53537
H	1.98604	1.8604	-2.73755	H	3.86838	1.5272	-2.3552
H	3.53611	2.32031	0.84615	H	3.27911	-0.33628	2.34932
H	0.17368	-0.25066	-1.73503	H	1.01674	1.40634	-1.48981
H	1.82343	-0.38971	-2.28388	H	2.0793	0.22894	-2.22508
H	2.76662	-3.20453	0.06736	H	0.5256	-2.80752	-0.64283
H	3.37185	-2.21574	-1.24355	H	1.80048	-2.26978	-1.71289
H	2.59289	-1.23612	1.54672	H	1.57341	-1.60589	1.26834
H	4.227	-1.3815	0.9178	H	2.67108	-2.79665	0.5803
H	4.07575	1.54599	-1.3907	H	4.66546	-2.11937	-0.70401
H	5.12366	0.30842	-0.68324	H	3.73362	-1.20299	-1.88915
H	3.91335	-0.14075	-1.88863	H	5.04079	-0.41586	-1.00981
H	-0.33907	2.3993	0.59562	H	4.03188	2.06607	1.75993
H	1.23071	2.81093	1.26274	H	4.29536	3.49331	0.73866
H	0.5054	3.84796	0.0132	H	2.66124	3.05857	1.28363
H	-1.28065	-1.96871	-1.4973	H	-1.73334	-2.05149	-0.8545
H	-1.90262	-2.87365	-0.12803	H	-1.14046	-2.26001	0.77582
H	-1.9053	-0.79992	1.27457	H	-3.55516	-2.06987	0.81404
H	-1.24743	0.12349	-0.0463	H	-2.90858	-0.67654	1.63351
H	-0.50499	-3.10845	1.91609	H	-1.6141	0.99566	1.16728

H	1.32469	-3.03751	1.7035	H	-0.1069	1.63358	0.33846
H	-3.98756	2.68477	0.97166	H	-5.35337	2.26901	1.38132
H	-2.49633	1.77677	1.27141	H	-3.95621	1.34881	1.97218
H	-2.9059	2.29517	-0.38157	H	-3.82797	2.29595	0.47148
H	-5.23799	0.18637	-1.14733	H	-5.13698	0.85725	-1.63419
H	-3.81495	-0.15265	2.37519	H	-5.42368	-0.78808	1.97708
H	-5.30046	0.78596	2.12586	H	-6.80355	0.14248	1.36892
H	-5.18235	-0.86881	1.49105	H	-6.24571	-1.2896	0.48035

atom	Con f. 6- 1a			atom	Con f. 6- 1b		
C	-44.42772	-0.67125	-1.07816	C	-44.42964	-0.62189	-1.0972
C	-46.60591	2.28483	1.53974	C	-46.62832	2.28993	1.56373
C	-46.2519	1.01528	1.77228	C	-46.26413	1.02002	1.77808
C	-47.09624	-0.17424	1.33402	C	-47.09983	-0.16996	1.32442
C	-46.80643	-0.6468	-0.13995	C	-46.80736	-0.61978	-0.15601
C	-45.36613	-1.10052	-0.233	C	-45.36476	-1.06497	-0.25548
C	-48.59913	0.05132	1.57672	C	-48.60425	0.04104	1.57144
C	-49.43029	-1.21446	1.27321	C	-49.42634	-1.22637	1.2498
C	-49.17611	-1.68024	-0.16942	C	-49.1709	-1.66707	-0.2004
C	-47.68087	-1.90456	-0.40572	C	-47.67441	-1.87866	-0.4406
C	-44.98639	0.68848	2.5225	C	-44.99473	0.69273	2.52142
C	-47.12314	0.44292	-1.17021	C	-47.12923	0.48299	-1.17063
C	-50.88848	-0.96175	1.59502	C	-50.88538	-0.98808	1.5765
C	-51.42407	-1.20797	2.81817	C	-51.4242	-1.27452	2.79009
C	-51.73916	-0.34698	0.52103	C	-51.73432	-0.34054	0.52058
C	-50.71959	-1.74034	3.95621	C	-50.72501	-1.84871	3.90851
C	-51.2878	-1.92218	5.17403	C	-51.27976	-2.0706	5.12532
C	-50.58712	-2.443	6.34574	C	-50.50608	-2.64885	6.22942
C	-49.12378	-2.81623	6.24469	C	-51.23378	-2.83439	7.53915
O	-51.19237	-2.57461	7.41938	O	-49.31623	-2.97185	6.11228
H	-46.80359	-1.0217	1.96955	H	-46.80034	-1.02365	1.94815
H	-49.05547	-2.00682	1.9304	H	-49.04494	-2.02489	1.89525
H	-43.42621	-1.09351	-1.06104	H	-43.42609	-1.03948	-1.08619
H	-44.60989	0.10785	-1.81242	H	-44.61673	0.16437	-1.82254
H	-45.98098	3.11137	1.86948	H	-46.00928	3.11666	1.90398
H	-47.52012	2.54983	1.01822	H	-47.54539	2.555	1.04732
H	-45.10397	-1.8889	0.4746	H	-45.09802	-1.8599	0.44296
H	-48.96945	0.88301	0.96965	H	-48.98098	0.87892	0.97696
H	-48.74687	0.33732	2.62323	H	-48.75294	0.30993	2.62231
H	-49.72066	-2.61219	-0.36148	H	-49.71026	-2.59888	-0.40726
H	-49.55888	-0.94027	-0.8792	H	-49.55814	-0.91768	-0.89784
H	-47.33606	-2.71379	0.25185	H	-47.3246	-2.69557	0.20463
H	-47.50871	-2.24119	-1.43472	H	-47.50029	-2.19845	-1.47464
H	-44.43965	1.59216	2.80767	H	-44.45479	1.59664	2.81867
H	-45.21418	0.12064	3.43422	H	-45.21637	0.10988	3.42508
H	-44.32063	0.06009	1.92106	H	-44.32494	0.07856	1.90981
H	-46.46923	1.30915	-1.04764	H	-46.47558	1.34811	-1.03914
H	-46.98972	0.05558	-2.18556	H	-46.99949	0.10928	-2.19158
H	-48.15406	0.79312	-1.08537	H	-48.16	0.83152	-1.07723
H	-52.47295	-0.96255	2.979	H	-52.47309	-1.03175	2.9553
H	-51.87512	-1.03782	-0.31998	H	-51.8705	-1.0069	-0.33996
H	-52.72666	-0.07201	0.8998	H	-52.7219	-0.07544	0.90615
H	-51.26171	0.55072	0.10967	H	-51.25513	0.56774	0.13544
H	-49.67132	-1.99511	3.82967	H	-49.67808	-2.11772	3.79015
H	-52.33455	-1.67127	5.33545	H	-52.32015	-1.81798	5.31568
H	-48.52076	-1.94837	5.95725	H	-51.6079	-1.8689	7.90001
H	-48.78066	-3.18981	7.21087	H	-52.1085	-3.47961	7.39547
H	-48.97013	-3.58695	5.4822	H	-50.57035	-3.27397	8.28596

atom	Con f. 6- 1c			atom	Con f. 6- 1d		
C	-44.63019	-2.03764	-0.06981	C	-44.72857	-2.0225	-0.55889
C	-46.60914	2.69512	0.25194	C	-45.09291	1.07586	2.37058
C	-46.18734	1.65247	0.97736	C	-46.24842	1.212	1.70869
C	-46.9773	0.35853	1.11414	C	-47.0488	-0.01861	1.29967
C	-46.78333	-0.65239	-0.0805	C	-46.77603	-0.53723	-0.16517
C	-45.30207	-0.90409	-0.27799	C	-45.29868	-0.84235	-0.31009
C	-48.46934	0.60943	1.39558	C	-48.5525	0.17729	1.57008
C	-49.20965	-0.69875	1.72842	C	-49.36608	-1.10764	1.30978
C	-49.02186	-1.72681	0.59175	C	-49.1217	-1.59728	-0.12692
C	-47.53694	-1.94635	0.28831	C	-47.62712	-1.80855	-0.37257
C	-44.89824	1.71296	1.75583	C	-46.80126	2.5837	1.40601
C	-47.31291	-0.08894	-1.41556	C	-47.12144	0.5125	-1.24337
C	-50.67681	-0.48048	2.02435	C	-50.82505	-0.87628	1.63788
C	-51.32164	-1.04671	3.07691	C	-51.37353	-1.1927	2.83892

C	-51.4477	0.39102	1.0722	C	-51.671	-0.21478	0.58734
C	-50.74254	-1.9039	4.07661	C	-50.6883	-1.79702	3.95243
C	-51.44327	-2.45164	5.09961	C	-51.27906	-2.06046	5.14424
C	-50.80915	-3.32162	6.09547	C	-50.59986	-2.66359	6.28885
C	-51.70461	-3.86003	7.1855	C	-49.13718	-3.03947	6.18488
O	-49.60511	-3.60928	6.06168	O	-51.22241	-2.86446	7.34165
H	-46.57158	-0.16503	1.99033	H	-46.71243	-0.83812	1.9472
H	-48.72535	-1.11908	2.61566	H	-48.96855	-1.87927	1.97732
H	-43.56115	-2.09345	-0.2593	H	-43.64877	-2.10787	-0.65316
H	-45.10209	-2.94679	0.29065	H	-45.29377	-2.94175	-0.68006
H	-46.02	3.60649	0.18288	H	-44.50458	1.93813	2.67573
H	-47.54968	2.68597	-0.28998	H	-44.69501	0.09725	2.62722
H	-44.75115	-0.0401	-0.64998	H	-44.6521	0.02756	-0.206
H	-48.93568	1.09798	0.53631	H	-48.9511	0.98937	0.95375
H	-48.55835	1.30581	2.237	H	-48.68625	0.48144	2.61425
H	-49.48072	-2.67708	0.88659	H	-49.65808	-2.53845	-0.29563
H	-49.54936	-1.38905	-0.30689	H	-49.52093	-0.87494	-0.84592
H	-47.06447	-2.38969	1.17357	H	-47.26947	-2.58954	0.31011
H	-47.4283	-2.6734	-0.52503	H	-47.46808	-2.18325	-1.39019
H	-45.10206	1.65215	2.83338	H	-46.07918	3.36104	1.67116
H	-44.2492	0.86267	1.5179	H	-47.06269	2.7049	0.35093
H	-44.34636	2.63765	1.56324	H	-47.71863	2.77212	1.97654
H	-46.74317	0.79094	-1.72502	H	-46.41873	1.34997	-1.22252
H	-47.21267	-0.84668	-2.20015	H	-47.05765	0.05593	-2.23717
H	-48.36575	0.19749	-1.36303	H	-48.12983	0.91675	-1.12543
H	-52.38708	-0.84721	3.18569	H	-52.42524	-0.9587	2.99893
H	-52.52283	0.33633	1.26031	H	-51.81703	-0.87762	-0.27448
H	-51.1409	1.43999	1.16755	H	-52.65499	0.05716	0.97723
H	-51.26103	0.10698	0.03025	H	-51.18617	0.69031	0.20244
H	-49.68249	-2.1398	4.02101	H	-49.63824	-2.045	3.8293
H	-52.50744	-2.25575	5.20769	H	-52.32843	-1.81953	5.3039
H	-52.5337	-4.42695	6.74592	H	-48.52436	-2.15892	5.96495
H	-52.15042	-3.03053	7.74731	H	-48.81154	-3.4787	7.12928
H	-51.1385	-4.50085	7.86377	H	-48.97646	-3.7594	5.37565

atom	Con f. 6- 1e			atom	Con f. 6- 1f		
C	-44.36147	-1.1836	-0.89819	C	-44.72769	-1.98121	-0.61068
C	-44.94826	1.66212	1.61221	C	-45.10642	1.04523	2.39266
C	-46.16494	1.55154	1.06586	C	-46.26042	1.19289	1.73066
C	-46.95745	0.25832	1.20736	C	-47.05665	-0.03029	1.29276
C	-46.70457	-0.77344	0.04315	C	-46.78159	-0.51446	-0.18322
C	-45.25409	-1.20221	0.09189	C	-45.30286	-0.80948	-0.33521
C	-48.45806	0.52267	1.42645	C	-48.56106	0.15427	1.56664
C	-49.23086	-0.77224	1.72908	C	-49.36838	-1.12875	1.27833
C	-49.03136	-1.78004	0.57907	C	-49.12295	-1.58437	-0.16949
C	-47.54181	-2.04668	0.35272	C	-47.62726	-1.78452	-0.41868
C	-46.78502	2.71615	0.33272	C	-46.81523	2.56947	1.45462
C	-47.08248	-0.2186	-1.3357	C	-47.13217	0.55622	-1.23903
C	-50.69907	-0.52516	1.99636	C	-50.82723	-0.91155	1.61486
C	-51.37262	-1.07296	3.0401	C	-51.37079	-1.25715	2.81072
C	-51.43451	0.359	1.02776	C	-51.6785	-0.22987	0.582
C	-50.82536	-1.93775	4.0537	C	-50.68048	-1.88513	3.90563
C	-51.55843	-2.45161	5.07225	C	-51.2506	-2.17799	5.10003
C	-51.03037	-3.3279	6.11568	C	-50.48918	-2.8133	6.18112
C	-49.57079	-3.72829	6.09827	C	-51.23603	-3.08105	7.46571
O	-51.77908	-3.73994	7.01318	O	-49.29496	-3.11975	6.06484
H	-46.58518	-0.24135	2.11139	H	-46.71817	-0.86351	1.92129
H	-48.77466	-1.21524	2.62022	H	-48.96631	-1.91235	1.9287
H	-43.3484	-1.54597	-0.74286	H	-43.64757	-2.05974	-0.70736
H	-44.59232	-0.8109	-1.89185	H	-45.28881	-2.90011	-0.75175
H	-44.35945	2.57123	1.51404	H	-44.52094	1.90202	2.71808
H	-44.4981	0.84835	2.17458	H	-44.70688	0.06225	2.62911
H	-44.94195	-1.59304	1.06173	H	-44.65991	0.06064	-0.21193
H	-48.88562	1.01096	0.54654	H	-48.96174	0.97802	0.96755
H	-48.57728	1.22244	2.26196	H	-48.69629	0.43493	2.6171
H	-49.53957	-2.71971	0.82318	H	-49.65573	-2.52354	-0.35943
H	-49.49547	-1.39778	-0.33675	H	-49.52493	-0.84704	-0.87168
H	-47.13246	-2.52091	1.25447	H	-47.26718	-2.57807	0.24798
H	-47.40694	-2.76193	-0.46704	H	-47.46588	-2.13719	-1.44377
H	-46.07243	3.54041	0.23852	H	-47.06511	2.71482	0.39974
H	-47.12668	2.44389	-0.67033	H	-47.73951	2.74129	2.01908
H	-47.66482	3.09389	0.86774	H	-46.09894	3.34289	1.74589
H	-46.42186	0.59896	-1.63435	H	-46.4322	1.39548	-1.20323
H	-47.00242	-1.00344	-2.09513	H	-47.0687	0.12006	-2.24198

H	-48.10895	0.15369	-1.3565	H	-48.1417	0.95501	-1.11157
H	-52.43485	-0.85037	3.13326	H	-52.42259	-1.02879	2.97867
H	-51.24012	0.06349	-0.00961	H	-52.66309	0.02825	0.97975
H	-52.51317	0.33176	1.20093	H	-51.19857	0.68608	0.21713
H	-51.10347	1.40076	1.12076	H	-51.82293	-0.87336	-0.29466
H	-49.77122	-2.19115	3.9923	H	-49.63032	-2.14096	3.78748
H	-52.61799	-2.21794	5.15902	H	-52.29565	-1.94419	5.28868
H	-49.32203	-4.25277	5.16972	H	-52.10263	-3.72355	7.27009
H	-48.9248	-2.84612	6.1585	H	-51.62426	-2.14141	7.87648
H	-49.36856	-4.38252	6.94793	H	-50.58068	-3.55854	8.19621

atom	Con f. 6- 1g			atom	Con f. 6- 1h		
C	-44.62945	-2.13252	-0.02422	C	-45.46892	2.33993	0.49219
C	-44.99245	1.79517	1.5675	C	-45.39485	-1.76753	2.1726
C	-46.1977	1.65377	1.00255	C	-45.68861	-1.37126	0.92834
C	-46.95658	0.33819	1.12811	C	-47.07637	-0.89909	0.51367
C	-46.73395	-0.67529	-0.06146	C	-47.34004	0.62526	0.81095
C	-45.2517	-0.96444	-0.19169	C	-46.34209	1.44711	0.02366
C	-48.45444	0.57728	1.39608	C	-48.19005	-1.79212	1.08802
C	-49.20209	-0.73163	1.70141	C	-49.58343	-1.39009	0.56241
C	-49.0103	-1.72601	0.53746	C	-49.8708	0.10544	0.80531
C	-47.52175	-1.95976	0.27073	C	-48.74375	0.97297	0.24215
C	-46.84432	2.80764	0.27545	C	-44.64704	-1.39791	-0.16029
C	-47.19458	-0.10883	-1.42196	C	-47.27808	0.95062	2.30821
C	-50.66748	-0.50689	1.99872	C	-50.69227	-2.30458	1.05506
C	-51.31012	-1.06084	3.05924	C	-51.06343	-2.46769	2.35117
C	-51.4382	0.36265	1.0444	C	-51.40151	-3.07041	-0.02314
C	-50.72859	-1.90468	4.0689	C	-50.48306	-1.81389	3.4936
C	-51.42421	-2.43555	5.10413	C	-50.85492	-2.01503	4.78052
C	-50.78226	-3.28602	6.11197	C	-50.18862	-1.30281	5.87733
C	-51.66825	-3.80206	7.22036	C	-50.67337	-1.608	7.27364
O	-49.57855	-3.57442	6.07346	O	-49.27211	-0.49162	5.68594
H	-46.54692	-0.17634	2.00651	H	-47.13011	-0.99057	-0.58021
H	-48.72396	-1.17757	2.57916	H	-49.54184	-1.51446	-0.52795
H	-43.5538	-2.21269	-0.16108	H	-44.80887	2.88121	-0.18086
H	-45.14635	-3.04725	0.24991	H	-45.37251	2.56801	1.5495
H	-44.43133	2.72368	1.49144	H	-44.393	-2.10089	2.43245
H	-44.52475	0.98529	2.12173	H	-46.12858	-1.7763	2.97242
H	-44.65187	-0.09893	-0.46812	H	-46.3777	1.27404	-1.05333
H	-48.91286	1.07196	0.53552	H	-48.16968	-1.76172	2.17789
H	-48.55846	1.26374	2.24461	H	-47.98718	-2.83158	0.80699
H	-49.49277	-2.67749	0.78739	H	-50.81751	0.3751	0.32291
H	-49.50621	-1.3467	-0.363	H	-49.99985	0.3065	1.87158
H	-47.0815	-2.42161	1.1631	H	-48.71593	0.85501	-0.8493
H	-47.40049	-2.67774	-0.5487	H	-48.95199	2.03136	0.43826
H	-47.18479	2.53067	-0.72644	H	-44.50699	-0.4034	-0.59775
H	-47.72909	3.16601	0.81536	H	-43.67968	-1.74852	0.21155
H	-46.14955	3.64694	0.18068	H	-44.96187	-2.05877	-0.97864
H	-46.54835	0.70915	-1.75199	H	-46.2777	0.7784	2.71125
H	-47.14412	-0.89415	-2.18411	H	-47.53486	2.00171	2.47614
H	-48.22174	0.26303	-1.39447	H	-47.97458	0.3473	2.89383
H	-52.37457	-0.8573	3.16984	H	-51.87543	-3.16271	2.56153
H	-52.5125	0.31604	1.23916	H	-52.1938	-3.7128	0.36988
H	-51.12412	1.41026	1.13153	H	-51.84043	-2.37985	-0.75559
H	-51.25904	0.07163	0.00316	H	-50.68838	-3.69303	-0.57974
H	-49.66878	-2.14104	4.01384	H	-49.68149	-1.1023	3.335
H	-52.48783	-2.23847	5.21531	H	-51.65036	-2.71129	5.03484
H	-52.49856	-4.38119	6.79942	H	-51.74263	-1.38056	7.35774
H	-52.11268	-2.96164	7.76666	H	-50.11405	-1.02688	8.00887
H	-51.09534	-4.42596	7.90863	H	-50.55931	-2.6781	7.48359

atom	Con f. 6- 1i			atom	Con f. 6- 1j		
C	-45.32211	2.22292	0.49238	C	-46.24448	2.3896	-0.68373
C	-45.42302	-1.91478	2.1107	C	-44.68069	-1.49109	0.01884
C	-45.72339	-1.47954	0.88112	C	-45.66379	-1.45238	0.92642
C	-47.09415	-0.92989	0.50729	C	-47.02367	-0.86952	0.56122
C	-47.27042	0.60193	0.83137	C	-47.21897	0.65398	0.92611
C	-46.25115	1.38399	0.03178	C	-46.11913	1.46129	0.26611
C	-48.23895	-1.77277	1.09692	C	-48.16992	-1.73798	1.1124
C	-49.62101	-1.28952	0.61269	C	-49.55214	-1.24829	0.63809
C	-49.82354	0.21352	0.88886	C	-49.75844	0.24343	0.96641
C	-48.6688	1.03112	0.3069	C	-48.60767	1.07922	0.40414
C	-44.70867	-1.53835	-0.2314	C	-45.45668	-2.03087	2.30536
C	-47.15745	0.89717	2.33139	C	-47.1204	0.92211	2.44333

C	-50.7656	-2.1527	1.11529	C	-50.69142	-2.13635	1.10893
C	-51.11694	-2.32381	2.41518	C	-51.05036	-2.34776	2.40077
C	-51.54217	-2.85403	0.03906	C	-51.4524	-2.81662	0.00824
C	-50.48452	-1.73384	3.56777	C	-50.43369	-1.7834	3.57436
C	-50.87858	-1.96081	4.84492	C	-50.83601	-2.04744	4.84164
C	-50.23474	-1.37168	6.01728	C	-50.20667	-1.48251	6.03377
C	-49.04333	-0.45364	5.83949	C	-49.02638	-0.5442	5.88874
O	-50.66179	-1.6259	7.15248	O	-50.63706	-1.7731	7.1588
H	-47.17933	-1.00268	-0.58593	H	-47.09134	-0.89879	-0.53364
H	-49.6114	-1.39545	-0.48034	H	-49.53919	-1.31603	-0.45773
H	-44.65132	2.74107	-0.18818	H	-45.37105	2.90922	-1.07009
H	-45.18877	2.42792	1.55044	H	-47.19994	2.67387	-1.11452
H	-44.43424	-2.30368	2.34166	H	-43.70203	-1.90287	0.25439
H	-46.13886	-1.90287	2.92666	H	-44.82022	-1.10816	-0.98896
H	-46.32172	1.23055	-1.04644	H	-45.12018	1.24238	0.63972
H	-48.19105	-1.76157	2.18655	H	-48.13039	-1.76079	2.203
H	-48.09797	-2.81669	0.79565	H	-48.02229	-2.7717	0.77857
H	-50.76827	0.54071	0.43945	H	-50.70622	0.58245	0.53266
H	-49.91185	0.4007	1.9623	H	-49.84251	0.39333	2.0463
H	-48.67735	0.92974	-0.78649	H	-48.61593	0.99095	-0.68945
H	-48.81572	2.09562	0.52432	H	-48.77021	2.13794	0.63693
H	-44.52771	-0.54431	-0.65455	H	-46.07622	-2.92333	2.45497
H	-43.75251	-1.9444	0.112	H	-44.41281	-2.3222	2.45186
H	-45.0748	-2.16715	-1.05363	H	-45.72969	-1.32864	3.09851
H	-46.16129	0.66128	2.71177	H	-46.09715	0.79134	2.80535
H	-47.35364	1.95685	2.5247	H	-47.41385	1.95599	2.6559
H	-47.87727	0.31945	2.91371	H	-47.76923	0.26533	3.02693
H	-51.96296	-2.97644	2.62714	H	-51.8907	-3.01535	2.58739
H	-51.95972	-2.12419	-0.66734	H	-51.8715	-2.07254	-0.68212
H	-50.87853	-3.50256	-0.54829	H	-50.7775	-3.44253	-0.59077
H	-52.35933	-3.46036	0.43839	H	-52.26628	-3.44147	0.38487
H	-49.64399	-1.07089	3.40328	H	-49.59816	-1.10862	3.43583
H	-51.72027	-2.61758	5.0566	H	-51.67291	-2.7174	5.02972
H	-49.30671	0.412	5.22315	H	-48.2009	-1.03541	5.36316
H	-48.70665	-0.11008	6.81902	H	-48.69058	-0.23557	6.8801
H	-48.22175	-0.97567	5.33777	H	-49.30304	0.34216	5.3085

atom	Con f. 6- 1k			atom	Con f. 6- 1l		
C	-46.35951	2.44407	-0.69669	C	-44.42382	-0.78777	-0.57183
C	-44.62114	-1.33928	0.11085	C	-46.75451	2.22907	1.8054
C	-45.63237	-1.33861	0.98784	C	-46.47187	0.94949	2.07754
C	-47.00825	-0.83134	0.57298	C	-47.30391	-0.21202	1.55221
C	-47.29304	0.68396	0.91159	C	-46.88637	-0.69441	0.11215
C	-46.21625	1.53788	0.27183	C	-45.459	-1.19345	0.165
C	-48.12387	-1.75159	1.10148	C	-48.81558	0.05826	1.64514
C	-49.51643	-1.34113	0.58211	C	-49.63729	-1.19849	1.28468
C	-49.80811	0.14349	0.87857	C	-49.27528	-1.65666	-0.13785
C	-48.68423	1.0295	0.34004	C	-47.77118	-1.92236	-0.2446
C	-45.43968	-1.88581	2.38144	C	-45.31126	0.58169	2.96523
C	-47.25225	0.98153	2.42677	C	-47.06165	0.40387	-0.94256
C	-50.62144	-2.27821	1.04056	C	-51.11004	-0.94895	1.50354
C	-50.99772	-2.4846	2.32906	C	-51.76704	-1.28077	2.64433
C	-51.32171	-3.01372	-0.06423	C	-51.87038	-0.29727	0.38195
C	-50.42761	-1.8621	3.49366	C	-51.27205	-1.91374	3.85289
C	-50.80757	-2.09776	4.77223	C	-50.02307	-1.90551	4.38107
C	-50.14781	-1.41232	5.88988	C	-49.64973	-2.5965	5.61768
C	-50.64218	-1.74703	7.27604	C	-50.67511	-3.40375	6.38182
O	-49.22814	-0.59933	5.72248	O	-48.48466	-2.51575	6.03077
H	-47.03952	-0.87654	-0.52306	H	-47.10156	-1.06771	2.21164
H	-49.47058	-1.4265	-0.51161	H	-49.32252	-1.99779	1.96497
H	-45.50232	3.00251	-1.06489	H	-43.44248	-1.23967	-0.45182
H	-47.3145	2.67224	-1.16066	H	-44.50398	0.00032	-1.3146
H	-43.63031	-1.69661	0.38188	H	-46.14338	3.03621	2.20213
H	-44.74947	-0.97814	-0.90646	H	-47.59642	2.52081	1.18555
H	-45.21935	1.37671	0.67882	H	-45.29711	-1.992	0.89093
H	-48.11348	-1.75908	2.19253	H	-49.1069	0.88104	0.98439
H	-47.91346	-2.78005	0.78524	H	-49.05282	0.38153	2.66347
H	-50.75737	0.42679	0.40938	H	-49.82549	-2.57144	-0.38792
H	-49.93405	0.3059	1.95226	H	-49.57258	-0.8991	-0.87004
H	-48.6522	0.92678	-0.75187	H	-47.50934	-2.74835	0.42973
H	-48.90861	2.08136	0.55173	H	-47.51838	-2.25357	-1.25852
H	-45.78534	-1.19234	3.15335	H	-44.76715	1.46627	3.30924
H	-46.00805	-2.8132	2.52177	H	-45.66164	0.02743	3.84592
H	-44.38577	-2.1097	2.5692	H	-44.60618	-0.07412	2.44285

H	-46.23399	0.90696	2.81797	H	-46.40394	1.25423	-0.74894
H	-47.59745	2.00527	2.60894	H	-46.82789	0.01312	-1.93818
H	-47.88498	0.31025	3.01262	H	-48.08675	0.77886	-0.96968
H	-51.80672	-3.19048	2.51319	H	-52.841	-1.09984	2.65629
H	-52.11261	-3.67152	0.30524	H	-52.88613	-0.03153	0.68506
H	-51.76028	-2.30291	-0.77719	H	-51.35928	0.61233	0.04331
H	-50.603	-3.61544	-0.6365	H	-51.93622	-0.95825	-0.49066
H	-49.62726	-1.14427	3.3607	H	-52.04435	-2.43316	4.41852
H	-51.60372	-2.80103	5.00385	H	-49.21103	-1.3477	3.92991
H	-50.08537	-1.1841	8.02715	H	-50.20411	-3.83545	7.26642
H	-50.53285	-2.82184	7.46328	H	-51.08191	-4.20651	5.75827
H	-51.71118	-1.51776	7.35886	H	-51.51478	-2.77211	6.69044

atom	Con f. 6- 2a			atom	Con f. 6- 2b		
C	-2.64658	2.11414	1.16382	C	-2.62885	1.97775	1.27887
C	-6.31438	3.26934	5.3037	C	-6.35884	3.46053	5.2329
C	-5.94732	2.13895	4.68587	C	-6.09204	2.27813	4.66389
C	-5.51429	2.00237	3.22789	C	-5.57414	2.04892	3.24602
C	-4.50845	3.06074	2.64299	C	-4.5148	3.04451	2.64354
C	-3.92037	2.44567	1.382	C	-3.90203	2.34149	1.44144
C	-6.77837	1.86269	2.34403	C	-6.80049	1.90297	2.3114
C	-7.46994	3.22037	2.09874	C	-7.44493	3.27228	2.01193
C	-6.49394	4.17978	1.39911	C	-6.43072	4.15692	1.27383
C	-5.2268	4.37791	2.24064	C	-5.17418	4.35844	2.13332
C	-6.00746	0.82097	5.4176	C	-6.39464	1.00014	5.40854
C	-3.40288	3.3765	3.65659	C	-3.43542	3.38572	3.67697
C	-8.78459	3.01227	1.3746	C	-8.78137	3.08793	1.32266
C	-9.92876	2.70961	2.04121	C	-9.8992	2.778	2.03161
C	-8.78282	3.07828	-0.12409	C	-8.83187	3.18202	-0.17238
C	-10.02892	2.54946	3.46888	C	-9.92314	2.58911	3.45781
C	-11.16001	2.19778	4.12852	C	-10.99522	2.19997	4.18934
C	-11.22788	2.00836	5.57587	C	-10.87879	1.99895	5.63823
C	-9.98011	2.19243	6.41574	C	-12.13704	1.59591	6.3685
O	-12.29938	1.69159	6.11236	O	-9.8098	2.14417	6.24841
H	-4.9861	1.04323	3.16859	H	-5.07486	1.07204	3.26867
H	-7.68648	3.65175	3.07983	H	-7.63378	3.75236	2.97625
H	-2.34153	1.67601	0.21662	H	-2.30672	1.47879	0.36811
H	-1.86124	2.25916	1.8998	H	-1.86058	2.15375	2.02596
H	-6.64174	3.24986	6.34046	H	-6.78651	3.50761	6.23143
H	-6.28652	4.2427	4.82978	H	-6.16426	4.41029	4.75083
H	-4.63031	2.25942	0.57866	H	-4.59279	2.11869	0.63076
H	-6.514	1.41326	1.38068	H	-6.50788	1.42571	1.37001
H	-7.47827	1.17413	2.82782	H	-7.53397	1.24111	2.77973
H	-6.23259	3.79196	0.40883	H	-6.16644	3.70574	0.31102
H	-6.97266	5.15254	1.23758	H	-6.86906	5.1366	1.05127
H	-4.51408	5.00587	1.69262	H	-4.42329	4.91986	1.56453
H	-5.48601	4.93313	3.14762	H	-5.43406	4.98401	2.99334
H	-5.00701	0.37526	5.49236	H	-6.89802	1.19348	6.35928
H	-6.6283	0.09805	4.87293	H	-5.47086	0.4424	5.61065
H	-6.41166	0.92938	6.42795	H	-7.03515	0.33682	4.81437
H	-2.89411	2.46562	3.99046	H	-2.96559	2.48029	4.07623
H	-2.65164	4.03928	3.2149	H	-2.65048	4.00256	3.22721
H	-3.81207	3.87075	4.53893	H	-3.8574	3.9383	4.51725
H	-10.83756	2.53878	1.46622	H	-10.83273	2.61693	1.49463
H	-9.75898	2.81529	-0.53914	H	-9.83074	2.96123	-0.55699
H	-8.51727	4.08212	-0.477	H	-8.54099	4.1818	-0.51667
H	-8.03231	2.39597	-0.54267	H	-8.12111	2.48126	-0.62874
H	-9.12666	2.70302	4.05239	H	-9.00281	2.74783	4.01343
H	-12.09033	2.02924	3.58949	H	-11.96171	2.01881	3.72545
H	-9.57672	3.20346	6.29963	H	-11.93804	1.47291	7.43454
H	-10.22609	2.01913	7.46472	H	-12.9153	2.35436	6.22244
H	-9.19732	1.49211	6.10642	H	-12.52648	0.65821	5.95458

atom	Con f. 6- 2c			atom	Con f. 6- 2d		
C	-2.68544	2.58407	0.86678	C	-2.65493	2.85852	0.85281
C	-4.87175	0.8451	5.30555	C	-4.68985	0.75274	5.20472
C	-5.52135	1.8147	4.64847	C	-5.41642	1.6987	4.59596
C	-5.37909	1.90812	3.13378	C	-5.29449	1.87717	3.08748
C	-4.47752	3.10161	2.61762	C	-4.47413	3.15044	2.62648
C	-3.95893	2.68495	1.25131	C	-3.92999	2.83074	1.24439
C	-6.73467	1.83534	2.38681	C	-6.65357	1.75645	2.35133
C	-7.48761	3.17898	2.23585	C	-7.48909	3.05653	2.27414
C	-6.57045	4.24389	1.61468	C	-6.64482	4.1965	1.68262
C	-5.29786	4.40837	2.44695	C	-5.37951	4.40682	2.5154

C	-6.40082	2.77476	5.41105	C	-6.37169	2.54574	5.39862
C	-3.3218	3.37462	3.5852	C	-3.33883	3.45646	3.60829
C	-8.76805	2.94359	1.45938	C	-8.76942	2.79351	1.50868
C	-9.95257	2.66647	2.06407	C	-9.95783	2.53984	2.1147
C	-8.68316	2.94962	-0.03985	C	-8.68582	2.77307	0.00835
C	-10.16038	2.58634	3.48404	C	-10.19549	2.51311	3.53459
C	-11.31838	2.22254	4.08596	C	-11.38878	2.20224	4.09826
C	-11.41946	2.1462	5.54765	C	-11.63088	2.1634	5.53887
C	-12.75681	1.73174	6.11193	C	-10.50481	2.47749	6.50134
O	-10.46505	2.39981	6.29557	O	-12.75413	1.86687	5.97046
H	-4.8324	1.00163	2.85154	H	-4.69583	1.02176	2.75559
H	-7.7467	3.5346	3.23416	H	-7.74577	3.35761	3.29047
H	-2.43319	2.27125	-0.1435	H	-2.38164	2.60648	-0.16888
H	-1.84909	2.80299	1.52404	H	-1.83792	3.12752	1.51583
H	-4.94384	0.74215	6.38589	H	-4.74924	0.59203	6.27873
H	-4.24742	0.12109	4.7877	H	-4.01129	0.10782	4.65191
H	-4.72636	2.44001	0.51945	H	-4.67678	2.54487	0.50584
H	-6.55761	1.42891	1.38473	H	-6.46316	1.4107	1.32972
H	-7.38232	1.11565	2.89872	H	-7.24833	0.97278	2.83314
H	-6.31316	3.97309	0.58598	H	-6.38055	3.9743	0.64406
H	-7.10116	5.20164	1.56183	H	-7.23287	5.1215	1.66587
H	-4.64868	5.16279	1.98673	H	-4.78296	5.22141	2.0879
H	-5.56615	4.79353	3.43716	H	-5.66584	4.72748	3.52364
H	-6.26426	2.64787	6.48896	H	-7.41129	2.31285	5.14286
H	-7.46222	2.60358	5.19711	H	-6.2325	3.61572	5.21787
H	-6.19018	3.81897	5.16359	H	-6.2482	2.36205	6.46981
H	-2.7125	2.48125	3.75302	H	-2.6686	2.60072	3.7344
H	-2.67073	4.16231	3.19225	H	-2.74521	4.30607	3.25546
H	-3.69735	3.70856	4.55568	H	-3.73635	3.71544	4.59273
H	-10.81657	2.45783	1.43474	H	-10.81816	2.32131	1.48355
H	-9.61849	2.61531	-0.49555	H	-9.61342	2.40794	-0.43943
H	-8.45946	3.95501	-0.41715	H	-8.49115	3.77824	-0.38558
H	-7.87289	2.29986	-0.39193	H	-7.86016	2.14029	-0.33739
H	-9.329	2.8245	4.14081	H	-9.365	2.75333	4.19015
H	-12.19906	1.96763	3.50156	H	-12.24666	1.9537	3.47628
H	-13.53875	2.42158	5.77337	H	-10.88426	2.43008	7.52343
H	-13.02749	0.73719	5.73776	H	-9.68687	1.75812	6.38678
H	-12.72405	1.71733	7.20277	H	-10.09195	3.47373	6.31294

atom	Con f. 6- 2e			atom	Con f. 6- 2f		
C	-2.72335	2.1821	1.28103	C	-6.23264	-0.15235	3.43657
C	-6.46778	3.04568	5.39134	C	-4.18654	5.09295	2.21516
C	-6.16918	1.94419	4.69257	C	-4.47375	4.62449	3.43616
C	-5.66566	1.89203	3.25218	C	-5.28524	3.37534	3.76452
C	-4.64522	2.98653	2.76898	C	-4.98509	2.05316	2.97866
C	-4.01197	2.45627	1.4913	C	-5.52744	0.91067	3.82687
C	-6.88899	1.79882	2.30388	C	-6.79943	3.69154	3.73013
C	-7.59274	3.16084	2.12012	C	-7.34222	3.77625	2.29104
C	-6.59314	4.1715	1.52791	C	-7.0786	2.46744	1.51796
C	-5.35949	4.32411	2.42596	C	-5.59774	2.06977	1.56249
C	-6.40415	0.58086	5.29673	C	-4.03222	5.38658	4.66155
C	-3.57238	3.24241	3.83392	C	-3.463	1.8222	2.8804
C	-8.85337	3.00306	1.29938	C	-8.79224	4.22384	2.23393
C	-10.09552	2.87224	1.83107	C	-9.85407	3.54982	2.74439
C	-8.70152	3.00472	-0.19764	C	-9.01311	5.53837	1.54334
C	-10.51486	2.83007	3.22059	C	-9.83437	2.28475	3.4349
C	-9.8215	2.39046	4.29839	C	-10.94747	1.67994	3.91862
C	-10.33314	2.39195	5.66955	C	-10.95053	0.39523	4.61512
C	-11.7078	2.94491	5.97081	C	-9.65131	-0.35691	4.80972
O	-9.62866	1.94206	6.58523	O	-12.01584	-0.07836	5.03548
H	-5.13043	0.93807	3.16312	H	-5.04646	3.14797	4.8107
H	-7.8756	3.52338	3.1129	H	-6.76121	4.55845	1.79002
H	-2.38656	1.80598	0.31806	H	-6.50307	-0.92922	4.14665
H	-1.95663	2.31437	2.03839	H	-6.55585	-0.30609	2.41136
H	-6.88143	2.96813	6.39323	H	-3.62989	6.01997	2.09776
H	-6.3245	4.04891	5.01049	H	-4.47338	4.59071	1.30011
H	-4.69839	2.28817	0.6642	H	-5.23987	0.97278	4.8781
H	-6.57247	1.42618	1.32398	H	-7.32745	2.9129	4.28739
H	-7.59573	1.06042	2.69521	H	-6.99136	4.63576	4.25178
H	-6.28774	3.85014	0.52686	H	-7.69134	1.66241	1.92793
H	-7.07569	5.14865	1.40886	H	-7.39119	2.59426	0.47492
H	-4.63291	4.98807	1.94221	H	-5.46865	1.08192	1.10507
H	-5.65469	4.82138	3.35493	H	-5.01983	2.75828	0.93964
H	-6.99149	-0.05441	4.62243	H	-4.88535	5.60281	5.31754

H	-6.93594	0.64517	6.24937	H	-3.54795	6.33299	4.40494
H	-5.45119	0.06104	5.46191	H	-3.32892	4.7881	5.25533
H	-3.06646	2.31412	4.12104	H	-3.00412	1.79679	3.87553
H	-2.81419	3.93843	3.46047	H	-3.25438	0.86529	2.39061
H	-4.01003	3.67347	4.73462	H	-2.97928	2.61702	2.30722
H	-10.92396	2.83734	1.12454	H	-10.83864	4.00104	2.62666
H	-9.63796	2.74319	-0.69651	H	-8.43314	6.32954	2.03698
H	-8.38229	3.98697	-0.56623	H	-8.64619	5.49186	0.50925
H	-7.92983	2.2911	-0.51139	H	-10.06537	5.83378	1.52756
H	-11.54065	3.1594	3.38175	H	-8.88	1.79069	3.57447
H	-8.82435	1.98261	4.20467	H	-11.92397	2.14644	3.8019
H	-11.91451	2.83407	7.03653	H	-9.21478	-0.6225	3.84137
H	-11.76543	4.00391	5.69822	H	-8.91575	0.25627	5.33938
H	-12.47547	2.41737	5.39517	H	-9.84454	-1.26714	5.37992

atom	Con f. 6- 2g			atom	Con f. 6- 2h		
C	-5.1858	-0.26332	3.95134	C	-2.74545	2.40651	1.28723
C	-4.19172	5.05286	2.19291	C	-6.54733	2.9797	5.41143
C	-4.42881	4.49079	3.38485	C	-6.14338	1.91383	4.70955
C	-5.30997	3.27375	3.6548	C	-5.66803	1.91304	3.25874
C	-5.10668	1.99798	2.75701	C	-4.70593	3.06762	2.79426
C	-5.7738	0.83903	3.48294	C	-4.04803	2.59326	1.50737
C	-6.79691	3.70625	3.70624	C	-6.8885	1.76683	2.31516
C	-7.41237	3.88754	2.30359	C	-7.65808	3.09149	2.11254
C	-7.25385	2.61457	1.44406	C	-6.7025	4.16793	1.56372
C	-5.79241	2.15925	1.37352	C	-5.49147	4.37033	2.48044
C	-3.85436	5.10175	4.63892	C	-6.18913	0.53837	5.32759
C	-3.61332	1.72279	2.54881	C	-3.64768	3.36034	3.86383
C	-8.84127	4.40288	2.35108	C	-8.86294	2.85546	1.22841
C	-9.90275	3.7412	2.88062	C	-10.11243	2.59093	1.69021
C	-9.03793	5.76144	1.7452	C	-8.64439	2.91392	-0.25921
C	-9.87145	2.44122	3.49513	C	-10.60939	2.45839	3.04402
C	-10.93983	1.78861	4.01201	C	-9.96196	2.16174	4.19682
C	-10.78458	0.45976	4.61611	C	-10.69962	2.04033	5.4637
C	-12.03501	-0.18718	5.15982	C	-9.89445	1.60217	6.66275
O	-9.68998	-0.11647	4.68448	O	-11.90961	2.27865	5.56327
H	-5.05255	2.94833	4.66971	H	-5.08396	0.99132	3.14629
H	-6.82247	4.66762	1.80962	H	-8.0083	3.43047	3.09252
H	-5.76888	-1.02924	4.4569	H	-2.39064	2.06209	0.31889
H	-4.12084	-0.45403	3.85756	H	-1.98446	2.58242	2.04164
H	-3.57912	5.94852	2.11834	H	-6.89015	2.86695	6.43671
H	-4.57427	4.65798	1.26014	H	-6.54503	3.98806	5.01755
H	-6.84812	0.92719	3.63116	H	-4.7273	2.38728	0.68292
H	-7.36256	2.96735	4.2769	H	-6.5547	1.40134	1.3382
H	-6.87879	4.64765	4.26098	H	-7.56221	1.00117	2.71345
H	-7.87653	1.80758	1.8376	H	-6.36211	3.88646	0.56249
H	-7.62108	2.8169	0.43128	H	-7.23787	5.11837	1.45521
H	-5.73371	1.20022	0.84461	H	-4.80045	5.08761	2.02141
H	-5.21793	2.87233	0.77475	H	-5.82149	4.82437	3.41968
H	-3.31454	6.03064	4.43426	H	-6.7848	-0.14799	4.71204
H	-3.16548	4.40115	5.12879	H	-6.6167	0.55526	6.33372
H	-4.6466	5.3166	5.36779	H	-5.18162	0.10636	5.38664
H	-3.08751	1.6242	3.50472	H	-3.09124	2.45595	4.13337
H	-3.46355	0.79662	1.98441	H	-2.92918	4.10393	3.50394
H	-3.14459	2.53644	1.99282	H	-4.10878	3.75014	4.77196
H	-10.87553	4.22994	2.84233	H	-10.89024	2.48929	0.93474
H	-10.07544	6.10086	1.80117	H	-9.52924	2.58078	-0.8069
H	-8.39882	6.49856	2.2494	H	-8.40408	3.93204	-0.58832
H	-8.72569	5.75736	0.69228	H	-7.79576	2.28499	-0.55457
H	-8.92244	1.92345	3.56032	H	-11.68989	2.57228	3.13588
H	-11.93421	2.22782	3.99273	H	-8.89954	1.9613	4.23656
H	-12.77797	-0.29631	4.36086	H	-9.12022	2.34757	6.87741
H	-11.80569	-1.1655	5.58568	H	-10.54029	1.482	7.53433
H	-12.48588	0.45405	5.92646	H	-9.37619	0.66067	6.44854

atom	Con f. 6- 3a			atom	Con f. 6- 3b		
C	-4.74817	4.33325	2.58921	C	-4.04832	4.16983	3.37505
C	-4.58707	-0.03269	0.11163	C	-5.01809	1.14464	-0.37515
C	-5.296	0.68392	0.99267	C	-5.54828	1.58605	0.77205
C	-4.96455	0.61452	2.47426	C	-5.21281	0.89697	2.0855
C	-4.22604	1.87881	3.04411	C	-4.15534	1.65409	2.97206
C	-5.09954	3.11632	3.0108	C	-4.67107	2.99195	3.45963
C	-6.19013	0.21822	3.32002	C	-6.48261	0.53378	2.87802
C	-5.80065	-0.05327	4.79013	C	-6.15392	-0.35111	4.09287

C	-5.08818	1.17313	5.38517	C	-5.12546	0.35664	5.00215
C	-3.88168	1.56827	4.53032	C	-3.87757	0.75671	4.21267
C	-6.44536	1.54727	0.53739	C	-6.51129	2.7467	0.78151
C	-2.91058	2.09	2.28657	C	-2.84331	1.80138	2.19365
C	-7.01806	-0.49812	5.57189	C	-7.37418	-0.75594	4.89014
C	-7.3551	-1.80318	5.73523	C	-7.53218	-1.9937	5.42647
C	-7.91539	0.57314	6.123	C	-8.41855	0.29583	5.13505
C	-6.62331	-2.93806	5.23428	C	-6.61396	-3.09489	5.30783
C	-7.03574	-4.22179	5.37933	C	-6.7915	-4.30135	5.89971
C	-6.31277	-5.38434	4.86834	C	-5.81505	-5.38553	5.74918
C	-5.01085	-5.19259	4.11998	C	-6.12823	-6.67869	6.46291
O	-6.76645	-6.52362	5.04826	O	-4.78169	-5.2652	5.07724
H	-4.23591	-0.19856	2.5888	H	-4.72073	-0.04856	1.82318
H	-5.06818	-0.86742	4.77825	H	-5.6667	-1.25211	3.70602
H	-5.44632	5.16474	2.64446	H	-4.49768	5.06827	3.79072
H	-3.76911	4.55457	2.17456	H	-3.08226	4.29328	2.8948
H	-4.80786	-0.00768	-0.95307	H	-5.24687	1.61596	-1.32826
H	-3.76147	-0.6673	0.4243	H	-4.33767	0.29703	-0.40027
H	-6.10368	2.99393	3.41101	H	-5.63676	2.96963	3.96037
H	-6.9647	0.99123	3.28394	H	-6.99888	1.44066	3.2036
H	-6.63442	-0.68779	2.89329	H	-7.17454	0.00244	2.21388
H	-5.78276	2.01576	5.45791	H	-5.58659	1.23642	5.46499
H	-4.75776	0.9495	6.40626	H	-4.84174	-0.31608	5.81898
H	-3.38615	2.44392	4.96519	H	-3.16937	1.27488	4.86968
H	-3.14991	0.7492	4.54238	H	-3.37706	-0.15497	3.86033
H	-6.27663	2.59936	0.78707	H	-7.49691	2.44298	1.15381
H	-6.58809	1.46932	-0.54416	H	-6.16361	3.55161	1.43604
H	-7.38326	1.25648	1.02462	H	-6.64285	3.15393	-0.22506
H	-2.30871	2.87443	2.75639	H	-2.05319	2.21073	2.83141
H	-3.08191	2.36879	1.2435	H	-2.95481	2.45587	1.32553
H	-2.31927	1.1685	2.2944	H	-2.50709	0.8233	1.83457
H	-8.27406	-2.03024	6.2741	H	-8.42527	-2.18127	6.02117
H	-8.17268	1.30755	5.3502	H	-9.16828	-0.04312	5.85429
H	-8.84097	0.15297	6.52419	H	-7.96658	1.22107	5.5121
H	-7.41732	1.13046	6.92584	H	-8.93481	0.56077	4.20401
H	-5.69451	-2.75051	4.70346	H	-5.71127	-2.9663	4.71541
H	-7.964	-4.44674	5.90131	H	-7.67008	-4.49575	6.51022
H	-5.15839	-4.56225	3.23685	H	-5.3421	-7.41416	6.28341
H	-4.26798	-4.69762	4.7543	H	-6.22674	-6.49831	7.53995
H	-4.62879	-6.1664	3.8093	H	-7.09084	-7.07455	6.1182

atom	Con f. 6- 3c			atom	Con f. 6- 3d		
C	-4.82788	4.29984	2.48975	C	-4.57775	3.72162	4.35788
C	-6.34827	1.52783	0.56528	C	-6.53143	2.38594	0.55527
C	-5.34912	0.75749	1.01019	C	-5.58697	1.44946	0.70944
C	-4.99254	0.63494	2.48521	C	-5.21329	0.8592	2.06294
C	-4.23697	1.88576	3.0623	C	-4.14443	1.681	2.87488
C	-5.11476	3.1188	3.04029	C	-4.65494	3.0718	3.19539
C	-6.20399	0.23046	3.34431	C	-6.44453	0.56867	2.93805
C	-5.79489	-0.05597	4.80675	C	-6.063	-0.27239	4.16846
C	-5.06692	1.15903	5.4071	C	-4.99157	0.46567	4.99904
C	-3.872	1.56196	4.53869	C	-3.7904	0.86103	4.1348
C	-4.51844	-0.07196	0.06611	C	-4.87309	0.86877	-0.48476
C	-2.93537	2.10437	2.28273	C	-2.86062	1.86345	2.03939
C	-7.0031	-0.50478	5.59992	C	-7.25109	-0.63577	5.03135
C	-7.34432	-1.81019	5.75237	C	-7.42808	-1.8673	5.57481
C	-7.88863	0.56408	6.17421	C	-8.25066	0.44935	5.31699
C	-6.6235	-2.94239	5.23024	C	-6.56165	-3.00568	5.40806
C	-7.04067	-4.22597	5.36312	C	-6.78664	-4.21003	5.9894
C	-6.32791	-5.38529	4.83114	C	-5.92421	-5.37904	5.83295
C	-5.03102	-5.19035	4.07474	C	-4.67451	-5.28591	4.98367
O	-6.7856	-6.52465	5.0003	O	-6.21745	-6.44343	6.39601
H	-4.26892	-0.18696	2.57146	H	-4.72835	-0.10519	1.85839
H	-5.06639	-0.87329	4.77427	H	-5.60196	-1.19124	3.79133
H	-5.52823	5.12894	2.55421	H	-4.93051	4.74623	4.447
H	-3.90116	4.49491	1.95827	H	-4.16057	3.27875	5.25767
H	-6.57929	1.59225	-0.49544	H	-6.78294	2.7737	-0.42896
H	-6.96716	2.11953	1.23181	H	-7.09123	2.79271	1.39163
H	-6.07179	3.02285	3.54853	H	-5.07427	3.59938	2.34055
H	-6.97645	1.00581	3.31916	H	-6.89938	1.51187	3.25689
H	-6.65483	-0.67083	2.91535	H	-7.19537	0.03686	2.34346
H	-5.75638	2.00301	5.50561	H	-5.43789	1.35412	5.45558
H	-4.72037	0.91856	6.41902	H	-4.65827	-0.18077	5.8187
H	-3.36738	2.43097	4.9769	H	-3.06306	1.41544	4.73883

H	-3.14175	0.74143	4.53229	H	-3.27932	-0.05196	3.80246
H	-3.45237	0.16418	0.16475	H	-3.78546	0.94419	-0.38419
H	-4.62072	-1.13994	0.30094	H	-5.10084	-0.20142	-0.57936
H	-4.81063	0.07884	-0.97735	H	-5.16842	1.36388	-1.41446
H	-2.33974	2.90589	2.73186	H	-2.07974	2.33018	2.64912
H	-3.12734	2.37122	1.23964	H	-3.03033	2.50557	1.17004
H	-2.32976	1.19265	2.29487	H	-2.48496	0.89721	1.68468
H	-8.25802	-2.03917	6.29931	H	-8.30011	-2.02515	6.20815
H	-8.15067	1.30913	5.4133	H	-8.80239	0.72108	4.40856
H	-8.81177	0.14374	6.58086	H	-8.97539	0.13795	6.07333
H	-7.37853	1.10895	6.97805	H	-7.75633	1.36443	5.66339
H	-5.69881	-2.75285	4.69302	H	-5.67975	-2.88834	4.78519
H	-7.9652	-4.45315	5.89073	H	-7.65938	-4.36124	6.62199
H	-4.27956	-4.71065	4.7107	H	-3.99157	-4.52704	5.37999
H	-4.65814	-6.16169	3.74566	H	-4.1723	-6.25473	4.97737
H	-5.18128	-4.54514	3.20288	H	-4.9212	-4.99869	3.95628

atom	Con f. 6- 3e			atom	Con f. 6- 3f		
C	-4.88503	4.313	2.46084	C	-4.17174	4.23424	3.20489
C	-6.35375	1.50356	0.56136	C	-6.39895	2.63825	0.7656
C	-5.34464	0.75138	1.01494	C	-5.6008	1.5667	0.83553
C	-4.98793	0.64746	2.4914	C	-5.22775	0.89868	2.15209
C	-4.254	1.91537	3.05926	C	-4.1571	1.69311	2.98728
C	-5.15139	3.13387	3.02568	C	-4.69584	3.03104	3.44557
C	-6.19393	0.22909	3.35183	C	-6.46819	0.53846	2.98926
C	-5.7817	-0.03972	4.81683	C	-6.08785	-0.30734	4.21856
C	-5.07593	1.19269	5.40832	C	-5.04266	0.43692	5.07802
C	-3.88656	1.60947	4.53894	C	-3.82458	0.83683	4.24169
C	-4.5019	-0.07583	0.07968	C	-5.03923	0.9263	-0.40695
C	-2.95506	2.14805	2.27945	C	-2.87634	1.85171	2.1605
C	-6.98202	-0.50099	5.61388	C	-7.28193	-0.7116	5.05324
C	-7.28963	-1.81108	5.79864	C	-7.46556	-1.96868	5.53359
C	-7.89766	0.55806	6.15852	C	-8.28315	0.35902	5.3849
C	-6.53581	-2.93679	5.31498	C	-6.60492	-3.1004	5.3144
C	-6.91034	-4.22978	5.47483	C	-6.82166	-4.33264	5.83588
C	-6.10231	-5.33648	4.95179	C	-5.90856	-5.45084	5.576
C	-6.6392	-6.7293	5.17874	C	-6.26312	-6.773	6.21288
O	-5.03422	-5.15693	4.35108	O	-4.89543	-5.33479	4.87344
H	-4.25066	-0.16121	2.58548	H	-4.73409	-0.04989	1.90154
H	-5.04089	-0.84578	4.79115	H	-5.59708	-1.20927	3.83899
H	-5.59867	5.13128	2.51742	H	-4.63436	5.13319	3.60471
H	-3.9628	4.51698	1.92494	H	-3.27583	4.37988	2.60872
H	-6.58444	1.55408	-0.50021	H	-6.65167	3.09013	-0.19075
H	-6.98174	2.09319	1.22115	H	-6.82687	3.10292	1.64777
H	-6.10576	3.0284	3.53712	H	-5.59783	2.98993	4.05244
H	-6.97886	0.99151	3.31923	H	-6.98996	1.44812	3.29876
H	-6.62863	-0.68289	2.92887	H	-7.1686	-0.02317	2.36068
H	-5.78054	2.02495	5.49926	H	-5.50002	1.32039	5.53691
H	-4.72715	0.96637	6.42276	H	-4.72347	-0.21261	5.90074
H	-3.39766	2.49034	4.97132	H	-3.10697	1.38354	4.86481
H	-3.14262	0.80138	4.5402	H	-3.31735	-0.07539	3.90019
H	-3.43916	0.17536	0.17719	H	-3.94445	0.88109	-0.37031
H	-4.59012	-1.14269	0.32487	H	-5.38818	-0.11121	-0.49576
H	-4.79495	0.06103	-0.96548	H	-5.33241	1.4647	-1.31313
H	-2.37311	2.96342	2.72166	H	-2.08155	2.31468	2.75465
H	-3.1503	2.40192	1.2337	H	-3.03833	2.47191	1.27445
H	-2.33466	1.24651	2.30062	H	-2.51708	0.87264	1.82815
H	-8.20099	-2.04811	6.34627	H	-8.34175	-2.15288	6.15411
H	-7.4059	1.13325	6.95265	H	-8.84193	0.66169	4.49065
H	-8.17368	1.27916	5.37981	H	-9.00257	0.01728	6.1332
H	-8.81242	0.12426	6.56997	H	-7.79022	1.26231	5.76234
H	-5.60769	-2.75686	4.77769	H	-5.71909	-2.97716	4.69612
H	-7.8315	-4.48021	5.99557	H	-7.68684	-4.52383	6.46628
H	-7.62589	-6.8309	4.71116	H	-7.25701	-7.09526	5.88047
H	-5.95781	-7.4739	4.76352	H	-5.52369	-7.53264	5.95329
H	-6.77661	-6.90992	6.25134	H	-6.31329	-6.66325	7.30268

atom	Con f. 6- 3g			atom	Con f. 6- 3h		
C	-7.12325	0.47234	0.8103	C	-7.25705	0.52381	0.89172
C	-2.46008	1.97988	1.75921	C	-2.56431	2.00139	1.62946
C	-3.36669	1.04852	2.0795	C	-3.45059	1.067	1.99486
C	-4.38181	1.31455	3.17976	C	-4.41041	1.33127	3.1443
C	-5.83018	1.65615	2.66737	C	-5.88168	1.67547	2.70492
C	-6.48599	0.47467	1.98322	C	-6.57233	0.50306	2.03763

C	-4.38816	0.18616	4.22812	C	-4.36513	0.20168	4.19006
C	-5.29475	0.51866	5.43232	C	-5.2065	0.53729	5.43981
C	-6.71636	0.92794	4.99168	C	-6.65049	0.9375	5.06917
C	-6.6708	2.02325	3.9244	C	-6.66172	2.0345	4.00301
C	-3.38178	-0.2878	1.38076	C	-3.49501	-0.27054	1.29977
C	-5.78024	2.88586	1.7547	C	-5.87244	2.91532	1.80375
C	-5.28862	-0.57097	6.49198	C	-5.13936	-0.54374	6.50609
C	-5.73223	-1.8422	6.31699	C	-5.58334	-1.8193	6.3621
C	-4.73292	-0.15384	7.82213	C	-4.51928	-0.11309	7.80268
C	-6.28997	-2.39821	5.11043	C	-6.19444	-2.37677	5.18537
C	-6.70522	-3.68131	4.97703	C	-6.60693	-3.65901	5.04452
C	-7.26586	-4.22844	3.74237	C	-7.21176	-4.11821	3.78815
C	-7.40216	-3.34325	2.51944	C	-7.64106	-5.56384	3.73318
O	-7.6271	-5.41273	3.69683	O	-7.36806	-3.37221	2.81138
H	-4.05315	2.22707	3.69366	H	-4.05596	2.24283	3.64258
H	-4.85816	1.4111	5.89936	H	-4.75016	1.435	5.87721
H	-7.58541	-0.43654	0.4327	H	-7.74276	-0.37528	0.52066
H	-7.21021	1.35407	0.18222	H	-7.36265	1.4154	0.28079
H	-1.72075	1.8124	0.97924	H	-1.86375	1.83612	0.81409
H	-2.42647	2.94123	2.26608	H	-2.5096	2.96333	2.13333
H	-6.45616	-0.46247	2.53184	H	-6.52819	-0.44481	2.56901
H	-4.69011	-0.75602	3.76901	H	-4.69542	-0.73786	3.74555
H	-3.36402	0.03283	4.58785	H	-3.32393	0.04438	4.49511
H	-7.26991	0.06368	4.61658	H	-7.21504	0.07026	4.71865
H	-7.27067	1.28978	5.86531	H	-7.16506	1.29509	5.96857
H	-7.68957	2.2744	3.60712	H	-7.69575	2.28196	3.73619
H	-6.23848	2.93339	4.3614	H	-6.21258	2.94558	4.42073
H	-3.18165	-1.10691	2.08177	H	-3.25835	-1.08735	1.99191
H	-4.3574	-0.49279	0.93009	H	-4.49044	-0.48091	0.89746
H	-2.6235	-0.32726	0.59353	H	-2.77542	-0.30851	0.4769
H	-6.78949	3.22244	1.49647	H	-6.89177	3.25787	1.59857
H	-5.24018	2.6835	0.82625	H	-5.37866	2.72211	0.84805
H	-5.27436	3.71302	2.26328	H	-5.33969	3.7351	2.29652
H	-5.66468	-2.52355	7.16413	H	-5.46745	-2.49586	7.20792
H	-4.74886	-0.96374	8.55605	H	-4.49498	-0.91645	8.5435
H	-3.69863	0.19767	7.70974	H	-3.49347	0.24146	7.63519
H	-5.30212	0.69496	8.22399	H	-5.07101	0.7374	8.22486
H	-6.38433	-1.74743	4.25123	H	-6.34631	-1.73414	4.32714
H	-6.63545	-4.37956	5.80886	H	-6.49901	-4.37937	5.85164
H	-6.42571	-2.96147	2.20349	H	-8.07618	-5.7974	2.75994
H	-7.84229	-3.92315	1.70654	H	-6.78052	-6.21714	3.92003
H	-8.03853	-2.47837	2.73289	H	-8.3721	-5.76841	4.52428
atom	Con f. 6- 3i			atom	Con f. 6- 3j		
C	-7.2783	-0.3617	1.48217	C	-4.66878	4.23354	2.44237
C	-2.84059	0.07819	1.81323	C	-6.34406	1.5129	0.57425
C	-3.24642	1.26278	2.2876	C	-5.37315	0.70302	1.0114
C	-4.47539	1.42924	3.17262	C	-4.99775	0.57995	2.48169
C	-5.84158	1.54971	2.4017	C	-4.17723	1.80143	3.03239
C	-6.12585	0.30332	1.58707	C	-4.99904	3.07245	3.01078
C	-4.55769	0.34537	4.25994	C	-6.21152	0.23941	3.36495
C	-5.66703	0.6608	5.28319	C	-5.79118	-0.04993	4.82358
C	-7.03126	0.84277	4.58878	C	-4.99947	1.13726	5.3984
C	-6.93782	1.8571	3.44503	C	-3.80246	1.47693	4.50614
C	-2.45241	2.51538	2.0171	C	-4.59632	-0.17265	0.06325
C	-5.78453	2.7228	1.40097	C	-2.88037	1.95299	2.22935
C	-5.70413	-0.3208	6.44109	C	-7.00474	-0.43558	5.64134
C	-5.96682	-1.64928	6.34946	C	-7.40149	-1.72263	5.8141
C	-5.40904	0.28315	7.78362	C	-7.83162	0.67803	6.2181
C	-6.27092	-2.40178	5.1581	C	-6.74102	-2.89155	5.29309
C	-6.49689	-3.73881	5.16005	C	-7.21294	-4.15361	5.44722
C	-6.81071	-4.52465	3.96865	C	-6.56182	-5.34929	4.91673
C	-6.93633	-3.83624	2.62681	C	-5.27043	-5.2208	4.13707
O	-6.98275	-5.74838	4.06249	O	-7.06717	-6.46509	5.10608
H	-4.36064	2.39275	3.68803	H	-4.31037	-0.27276	2.56533
H	-5.41006	1.6378	5.71361	H	-5.10069	-0.89952	4.78836
H	-7.37255	-1.2036	0.80143	H	-5.33013	5.09391	2.50882
H	-8.16998	-0.09771	2.04359	H	-3.74323	4.38114	1.89383
H	-1.9486	-0.00414	1.19692	H	-6.58969	1.57666	-0.48323
H	-3.36859	-0.84681	2.02287	H	-6.92452	2.13878	1.24412
H	-5.29075	-0.02377	0.9706	H	-5.9508	3.025	3.53549
H	-4.73059	-0.62913	3.79678	H	-6.94874	1.04845	3.34379
H	-3.5949	0.27864	4.77811	H	-6.70948	-0.64506	2.95343
H	-7.39097	-0.11576	4.21035	H	-5.64864	2.01238	5.4988
H	-7.76944	1.18506	5.32349	H	-4.64701	0.89199	6.40713
H	-7.90779	1.94156	2.9419	H	-3.25208	2.32685	4.92635

H	-6.72375	2.84853	3.86508	H	-3.10997	0.62433	4.49699
H	-3.06951	3.29639	1.56041	H	-3.51927	0.01648	0.14179
H	-2.07109	2.93424	2.95797	H	-4.7422	-1.23245	0.31164
H	-1.59988	2.32169	1.35945	H	-4.89901	-0.01981	-0.97693
H	-6.76908	2.87608	0.94668	H	-2.24202	2.73161	2.65968
H	-5.0719	2.53258	0.593	H	-3.07759	2.21721	1.18657
H	-5.49232	3.65078	1.90513	H	-2.31612	1.01511	2.24191
H	-5.94831	-2.22786	7.27233	H	-8.31519	-1.90448	6.37847
H	-5.44807	-0.45138	8.59216	H	-8.07225	1.42654	5.45357
H	-4.41501	0.75008	7.78157	H	-8.76591	0.30403	6.64407
H	-6.12361	1.08713	8.0049	H	-7.28423	1.20722	7.0078
H	-6.32224	-1.873	4.21356	H	-5.81794	-2.74943	4.73873
H	-6.44937	-4.30446	6.08882	H	-8.1377	-4.33343	5.99237
H	-7.12154	-4.58456	1.85432	H	-4.48758	-4.769	4.75535
H	-7.7649	-3.12073	2.6428	H	-4.94717	-6.2113	3.81266
H	-6.02911	-3.27559	2.38198	H	-5.40621	-4.57844	3.26075

atom	Con f. 6- 3k			atom	Con f. 6- 3l		
C	-7.30602	-0.50123	1.71511	C	-4.0392	4.15502	3.11365
C	-2.88278	0.17643	1.70275	C	-6.42942	2.63074	0.78361
C	-3.3186	1.34832	2.18192	C	-5.67229	1.52854	0.82962
C	-4.49844	1.47208	3.13723	C	-5.26981	0.85637	2.13529
C	-5.91211	1.52332	2.4477	C	-4.13406	1.61384	2.917
C	-6.19374	0.23586	1.69905	C	-4.59975	2.97527	3.3861
C	-4.47711	0.39553	4.23394	C	-6.48653	0.55224	3.02766
C	-5.5311	0.68022	5.32419	C	-6.08783	-0.29899	4.24734
C	-6.94301	0.85073	4.72607	C	-4.97779	0.4089	5.05466
C	-6.94792	1.83218	3.55066	C	-3.78195	0.75395	4.16352
C	-2.60777	2.63377	1.84236	C	-5.19081	0.85634	-0.42952
C	-5.96076	2.66149	1.40783	C	-2.88471	1.71568	2.03503
C	-5.48457	-0.32665	6.46024	C	-7.2601	-0.64822	5.13615
C	-5.77147	-1.64995	6.35507	C	-7.47319	-1.89311	5.63578
C	-5.08351	0.24171	7.79013	C	-8.20159	0.46479	5.50141
C	-6.17368	-2.35953	5.1684	C	-6.66902	-3.06049	5.39067
C	-6.48902	-3.67719	5.13716	C	-6.9123	-4.27878	5.9328
C	-6.90893	-4.33256	3.8936	C	-6.05689	-5.43508	5.6449
C	-7.23582	-5.80367	3.98346	C	-6.43607	-6.73654	6.30964
O	-6.99957	-3.72626	2.81745	O	-5.07125	-5.36576	4.89843
H	-4.39405	2.44555	3.63617	H	-4.82498	-0.11294	1.8729
H	-5.25962	1.65244	5.75648	H	-5.65046	-1.22287	3.85551
H	-7.39811	-1.3815	1.08497	H	-4.44862	5.07431	3.52508
H	-8.16568	-0.26554	2.33562	H	-3.16436	4.26095	2.47905
H	-2.02685	0.12681	1.03397	H	-6.70495	3.08494	-0.16532
H	-3.34996	-0.76913	1.95946	H	-6.80012	3.11908	1.6788
H	-5.39026	-0.07113	1.03177	H	-5.47618	2.97416	4.03064
H	-4.64331	-0.58779	3.78796	H	-6.95803	1.48437	3.35062
H	-3.48361	0.36564	4.69455	H	-7.23492	0.01443	2.43456
H	-7.33285	-0.11525	4.4044	H	-5.37968	1.31316	5.52485
H	-7.6212	1.21285	5.50783	H	-4.64952	-0.24705	5.86866
H	-7.95057	1.87403	3.10943	H	-3.01708	1.27595	4.75057
H	-6.73572	2.84208	3.92546	H	-3.32647	-0.18019	3.8086
H	-3.29218	3.37496	1.4164	H	-5.50086	1.39902	-1.3275
H	-2.18785	3.08701	2.7503	H	-4.09814	0.7672	-0.43916
H	-1.79107	2.47107	1.13295	H	-5.5845	-0.16691	-0.49345
H	-6.97679	2.7646	1.01236	H	-2.04782	2.15184	2.5904
H	-5.29352	2.4677	0.56272	H	-3.06046	2.33479	1.15082
H	-5.67219	3.61655	1.86069	H	-2.57821	0.72075	1.69698
H	-5.70261	-2.25105	7.26106	H	-8.3283	-2.03671	6.29521
H	-4.092	0.70893	7.72152	H	-8.78602	0.7839	4.62955
H	-5.77677	1.03966	8.08798	H	-8.90104	0.15791	6.28301
H	-5.05976	-0.51377	8.57977	H	-7.6564	1.34971	5.84938
H	-6.23898	-1.82526	4.22678	H	-5.8066	-2.97816	4.73371
H	-6.44618	-4.28324	6.03901	H	-7.7564	-4.42977	6.60153
H	-8.03992	-5.96525	4.71117	H	-7.45572	-7.02092	6.02398
H	-7.53938	-6.18813	3.00819	H	-5.73983	-7.52764	6.02577
H	-6.36369	-6.36093	4.34561	H	-6.43398	-6.61592	7.39945

atom	Con f. 6- 3m			atom	Con f. 6- 3n		
C	-4.65784	4.02668	2.37588	C	-4.48242	3.96085	2.4464
C	-4.75396	-0.48754	0.27678	C	-4.73296	-0.54067	0.33454
C	-5.42668	0.31554	1.11023	C	-5.39367	0.30193	1.13815
C	-5.081	0.34015	2.59006	C	-5.11459	0.30663	2.63179
C	-4.26346	1.59986	3.05511	C	-4.2521	1.51786	3.14189
C	-5.06424	2.87945	2.92435	C	-4.9733	2.84061	2.98146

C	-6.31812	0.09252	3.47408	C	-6.40147	0.12212	3.45822
C	-5.92195	-0.1017	4.95449	C	-6.07527	-0.10977	4.95001
C	-5.14274	1.1287	5.45019	C	-5.26201	1.07757	5.49265
C	-3.92489	1.3905	4.56018	C	-3.99423	1.28522	4.65953
C	-6.55334	1.18208	0.60731	C	-6.44631	1.23013	0.58717
C	-2.94407	1.67327	2.27878	C	-2.89703	1.51935	2.42556
C	-7.13952	-0.43592	5.78373	C	-7.3357	-0.40598	5.72735
C	-7.51479	-1.70161	6.09931	C	-7.78253	-1.66293	5.98178
C	-7.97063	0.71605	6.27817	C	-8.1231	0.76899	6.23672
C	-6.89745	-2.9711	5.76327	C	-7.21418	-2.94268	5.60954
C	-6.11251	-3.30008	4.70779	C	-6.39653	-3.2721	4.57982
C	-5.54557	-4.63396	4.49348	C	-5.93554	-4.65549	4.38769
C	-5.79677	-5.74183	5.49222	C	-5.07592	-4.904	3.17177
O	-4.85327	-4.84625	3.48875	O	-6.22183	-5.57388	5.16499
H	-4.40282	-0.50556	2.76328	H	-4.49132	-0.57502	2.8312
H	-5.23306	-0.95231	4.99785	H	-5.42607	-0.99057	5.0068
H	-5.30599	4.89945	2.36633	H	-5.07859	4.86934	2.41298
H	-3.68125	4.14803	1.91666	H	-3.4849	4.02446	2.02185
H	-4.98522	-0.53025	-0.78515	H	-4.91985	-0.57253	-0.73645
H	-3.95003	-1.1297	0.62813	H	-3.98313	-1.22709	0.72021
H	-6.0608	2.85591	3.36021	H	-5.98423	2.87665	3.38234
H	-7.02965	0.92089	3.39737	H	-7.06037	0.99105	3.36363
H	-6.84107	-0.79819	3.10973	H	-6.95977	-0.73234	3.0614
H	-5.7904	2.01076	5.46344	H	-5.86726	1.98939	5.48572
H	-4.81246	0.96625	6.4829	H	-4.98517	0.89177	6.537
H	-3.37946	2.26953	4.92237	H	-3.42313	2.13427	5.05223
H	-3.23898	0.53609	4.63413	H	-3.35546	0.39738	4.75852
H	-6.69351	1.05552	-0.47	H	-6.20372	2.27693	0.79361
H	-7.49939	0.93397	1.10265	H	-6.54903	1.10723	-0.49475
H	-6.36303	2.24076	0.80788	H	-7.42461	1.03895	1.04361
H	-2.297	2.46086	2.67818	H	-2.22552	2.26926	2.85593
H	-3.10692	1.87125	1.2162	H	-3.00113	1.72756	1.35752
H	-2.40364	0.72496	2.36415	H	-2.41448	0.54219	2.53097
H	-8.3838	-1.80044	6.7483	H	-8.67633	-1.74397	6.59873
H	-8.21746	1.39944	5.45645	H	-8.31835	1.4852	5.42895
H	-8.90213	0.37239	6.73463	H	-9.08008	0.45735	6.66241
H	-7.42515	1.30991	7.02143	H	-7.56761	1.31635	7.00773
H	-7.14846	-3.76999	6.45964	H	-7.52535	-3.77574	6.24083
H	-5.87426	-2.59187	3.92276	H	-6.08056	-2.55292	3.835
H	-5.30363	-6.65209	5.14724	H	-4.17548	-4.2798	3.21814
H	-5.40826	-5.47095	6.47946	H	-4.79222	-5.95603	3.11081
H	-6.8696	-5.93123	5.60364	H	-5.61645	-4.6117	2.26374
atom	Con f. 6- 4a			atom	Con f. 6- 4b		
C	-3.74801	0.05764	4.86144	C	-3.75959	-0.02962	4.81036
C	-3.61818	2.93602	2.5214	C	-3.74363	3.01835	2.71074
C	-3.80503	2.06971	1.51823	C	-3.90353	2.26857	1.61375
C	-4.86293	0.97209	1.46369	C	-4.86668	1.09527	1.46132
C	-5.24706	0.24499	2.79942	C	-5.22103	0.24396	2.73092
C	-3.98183	-0.09547	3.55793	C	-3.95123	-0.07458	3.49171
C	-6.11658	1.51637	0.73465	C	-6.15041	1.59998	0.75898
C	-6.96555	2.41432	1.65667	C	-7.04636	2.39051	1.73344
C	-7.44578	1.59189	2.86107	C	-7.50626	1.4518	2.85535
C	-6.24741	1.0563	3.65285	C	-6.2891	0.91936	3.62364
C	-2.95302	2.15296	0.27498	C	-3.12606	2.59114	0.35968
C	-5.89455	-1.11288	2.4254	C	-5.77	-1.12121	2.24221
C	-8.0641	3.09539	0.86879	C	-8.14344	3.1097	0.97631
C	-7.85073	4.26345	0.20917	C	-7.89467	4.27751	0.32612
C	-9.39467	2.40902	0.77503	C	-9.49325	2.46404	0.89021
C	-6.59578	4.96911	0.17406	C	-6.60929	4.92322	0.29187
C	-6.37494	6.11219	-0.52054	C	-6.29272	6.02183	-0.43548
C	-5.08531	6.79754	-0.56743	C	-4.92836	6.56187	-0.423
C	-3.89893	6.21497	0.17324	C	-4.68187	7.79552	-1.25732
O	-4.96214	7.84588	-1.21713	O	-4.01063	6.04295	0.2286
H	-4.4398	0.19254	0.81695	H	-4.37686	0.40135	0.76514
H	-6.29878	3.18677	2.05081	H	-6.41359	3.15005	2.20253
H	-2.80714	-0.26207	5.3021	H	-2.80932	-0.32467	5.248
H	-4.46973	0.50561	5.53842	H	-4.52621	0.3042	5.50363
H	-2.8474	3.7003	2.45521	H	-3.05796	3.86233	2.7066
H	-4.18657	2.90873	3.44202	H	-4.25825	2.82444	3.64293
H	-3.20844	-0.5567	2.94088	H	-3.13358	-0.42325	2.85819
H	-6.73073	0.68484	0.37256	H	-6.71564	0.75523	0.35087
H	-5.80545	2.08215	-0.14857	H	-5.87768	2.2299	-0.09119
H	-8.07908	0.76447	2.52331	H	-8.09079	0.62336	2.43982
H	-8.06293	2.21108	3.52226	H	-8.16317	1.98183	3.55441
H	-5.73851	1.90189	4.1248	H	-5.84091	1.75195	4.17348

H	-6.60456	0.42188	4.47218	H	-6.62014	0.19848	4.3799
H	-2.36036	1.23676	0.15308	H	-2.52311	3.4946	0.47902
H	-3.5701	2.24444	-0.62752	H	-2.45796	1.76037	0.09696
H	-2.26581	3.0029	0.3074	H	-3.79019	2.74056	-0.49986
H	-6.1805	-1.65584	3.33185	H	-6.03733	-1.74697	3.09959
H	-6.79059	-0.9865	1.81225	H	-6.66008	-1.00845	1.61755
H	-5.19197	-1.73853	1.86466	H	-5.01658	-1.65724	1.65524
H	-8.6687	4.69452	-0.36618	H	-8.69687	4.74019	-0.24677
H	-9.27812	1.38367	0.40196	H	-9.41286	1.45112	0.47545
H	-9.86808	2.32584	1.76079	H	-9.94387	2.35397	1.88401
H	-10.07719	2.94383	0.10984	H	-10.17739	3.0412	0.26303
H	-5.76651	4.54228	0.72973	H	-5.80236	4.49397	0.87971
H	-7.17362	6.57815	-1.0946	H	-7.03272	6.52298	-1.05473
H	-4.11202	6.12122	1.24294	H	-3.6408	8.11225	-1.17393
H	-3.664	5.21311	-0.20078	H	-4.92251	7.59137	-2.30747
H	-3.03393	6.8652	0.03235	H	-5.34318	8.60751	-0.93262

atom	Con f. 6- 4c			atom	Con f. 6- 4d		
C	-2.92879	-0.23488	3.58431	C	-2.89862	-0.13467	3.54602
C	-3.79766	3.06861	2.73442	C	-3.92395	3.12263	2.8798
C	-3.8677	2.15492	1.75923	C	-3.97038	2.30555	1.8215
C	-4.88018	1.0162	1.65042	C	-4.89128	1.09965	1.6444
C	-5.35282	0.28393	2.94643	C	-5.33874	0.27692	2.89437
C	-4.21973	-0.09013	3.88194	C	-4.19922	-0.07536	3.83091
C	-6.09586	1.52779	0.83531	C	-6.12976	1.56873	0.83732
C	-7.01541	2.43321	1.68111	C	-7.09237	2.39427	1.71678
C	-7.55694	1.6389	2.87914	C	-7.62128	1.50751	2.85194
C	-6.40313	1.10816	3.73761	C	-6.45454	0.99157	3.70413
C	-2.91623	2.22299	0.58962	C	-3.09664	2.58148	0.62092
C	-6.00094	-1.06334	2.5319	C	-5.89382	-1.08694	2.40441
C	-8.0713	3.07109	0.80337	C	-8.14289	3.07281	0.86333
C	-7.82801	4.21396	0.11082	C	-7.8657	4.22277	0.19307
C	-9.38708	2.36628	0.65446	C	-9.47917	2.41028	0.71436
C	-6.58036	4.93341	0.12288	C	-6.59005	4.88717	0.22388
C	-6.33004	6.04653	-0.60958	C	-6.24749	5.98071	-0.49937
C	-5.04857	6.74842	-0.61173	C	-4.89692	6.54707	-0.40932
C	-3.90291	6.22089	0.22713	C	-4.62615	7.78211	-1.23411
O	-4.89891	7.76523	-1.30472	O	-4.0096	6.0504	0.29934
H	-4.39513	0.25151	1.03159	H	-4.34435	0.40259	0.9969
H	-6.38749	3.22924	2.09074	H	-6.49279	3.17757	2.1892
H	-2.21543	-0.56894	4.33354	H	-2.17659	-0.46479	4.28863
H	-2.52761	-0.02935	2.59606	H	-2.49789	0.14169	2.57491
H	-3.05251	3.85977	2.69746	H	-3.26511	3.98765	2.88028
H	-4.43602	3.0551	3.60889	H	-4.50425	2.96335	3.78008
H	-4.54663	-0.3275	4.89597	H	-4.52521	-0.3787	4.82745
H	-6.6737	0.68129	0.44942	H	-6.66201	0.70594	0.42349
H	-5.73583	2.08161	-0.03673	H	-5.80167	2.16786	-0.01563
H	-8.1794	0.80841	2.52942	H	-8.19375	0.66853	2.441
H	-8.2015	2.27631	3.4954	H	-8.30755	2.07352	3.49241
H	-5.91415	1.95072	4.23539	H	-6.02844	1.83237	4.25911
H	-6.80249	0.47485	4.5392	H	-6.8312	0.29044	4.45889
H	-2.2679	3.102	0.64052	H	-2.53758	3.51371	0.73277
H	-2.27928	1.32902	0.56057	H	-2.37972	1.76331	0.47094
H	-3.45688	2.24805	-0.3646	H	-3.6866	2.65021	-0.3007
H	-6.29347	-1.63243	3.4208	H	-6.15347	-1.71853	3.2607
H	-6.89633	-0.92435	1.92102	H	-6.79337	-0.97657	1.79378
H	-5.2898	-1.67052	1.96309	H	-5.14092	-1.61674	1.8125
H	-8.61236	4.61041	-0.53223	H	-8.63549	4.65738	-0.4428
H	-10.03064	2.86622	-0.07374	H	-10.12559	2.95679	0.02306
H	-9.23804	1.32817	0.33159	H	-9.36467	1.38305	0.34531
H	-9.92163	2.31738	1.61091	H	-9.99195	2.33366	1.6809
H	-5.78302	4.54371	0.74859	H	-5.81433	4.47971	0.86638
H	-7.09699	6.47269	-1.25356	H	-6.95705	6.46151	-1.16838
H	-3.04272	6.88332	0.11681	H	-3.59812	8.11972	-1.09184
H	-4.18355	6.16267	1.28357	H	-4.8016	7.56981	-2.29549
H	-3.62495	5.21089	-0.09162	H	-5.32078	8.58209	-0.95182

atom	Con f. 6- 4e			atom	Con f. 6- 4f		
C	-3.60715	0.29269	4.71426	C	-3.74556	0.05203	4.82831
C	-3.55313	3.04748	2.26999	C	-2.63217	1.80791	0.77471
C	-3.83	2.1831	1.28755	C	-3.67662	1.95448	1.60152
C	-4.87645	1.07437	1.33155	C	-4.85302	0.98667	1.47527
C	-5.19478	0.40872	2.7138	C	-5.28002	0.24472	2.79208
C	-3.89679	0.09531	3.42796	C	-4.03118	-0.16443	3.54373

C	-6.17087	1.56971	0.63614	C	-6.06753	1.63166	0.7637
C	-6.98862	2.51405	1.5414	C	-6.97031	2.486	1.68128
C	-7.39107	1.75394	2.81784	C	-7.44051	1.64089	2.87292
C	-6.15147	1.26409	3.57409	C	-6.23495	1.10039	3.65054
C	-3.10224	2.27975	-0.03234	C	-3.67204	3.07432	2.61333
C	-5.86036	-0.96337	2.43526	C	-5.99134	-1.07072	2.39107
C	-8.16258	3.10439	0.79367	C	-8.07695	3.12244	0.86267
C	-8.16289	4.34348	0.24034	C	-7.86548	4.2611	0.15291
C	-9.40246	2.2585	0.68778	C	-9.40125	2.42264	0.79134
C	-7.10547	5.33821	0.202	C	-6.60887	4.96219	0.09369
C	-5.7668	5.13374	0.16917	C	-6.36061	6.04882	-0.67726
C	-4.76388	6.19972	0.14995	C	-5.0592	6.71079	-0.74682
C	-5.19074	7.6494	0.19605	C	-3.89955	6.17948	0.07206
O	-3.56051	5.90619	0.10313	O	-4.90557	7.70131	-1.47576
H	-4.4741	0.2695	0.70154	H	-4.49445	0.20289	0.79846
H	-6.32925	3.33313	1.84433	H	-6.36447	3.29252	2.09859
H	-2.64882	-0.01428	5.12534	H	-2.81915	-0.31324	5.26417
H	-4.29772	0.76815	5.40481	H	-4.41074	0.59411	5.49414
H	-2.81757	3.83392	2.12134	H	-1.78287	2.48647	0.80427
H	-4.02436	3.01119	3.24326	H	-2.59073	1.00678	0.04064
H	-3.15194	-0.39087	2.79512	H	-3.31373	-0.71991	2.93762
H	-6.79308	0.7124	0.35988	H	-6.6823	0.83889	0.32174
H	-5.91316	2.07416	-0.2999	H	-5.70156	2.24002	-0.06885
H	-8.03434	0.90603	2.56051	H	-8.0702	0.81441	2.52746
H	-7.97838	2.40607	3.47488	H	-8.05862	2.24842	3.54387
H	-5.62212	2.13358	3.97343	H	-5.69079	1.94671	4.08073
H	-6.46611	0.67412	4.44274	H	-6.58471	0.49649	4.49577
H	-3.79897	2.26632	-0.87867	H	-3.69678	2.68259	3.63504
H	-2.50704	3.19351	-0.1007	H	-2.76776	3.6809	2.50847
H	-2.43538	1.41745	-0.16593	H	-4.53438	3.73802	2.50663
H	-6.105	-1.46163	3.37872	H	-6.33479	-1.60319	3.28416
H	-6.78277	-0.86447	1.85755	H	-6.85847	-0.8924	1.75005
H	-5.18426	-1.61679	1.87366	H	-5.30718	-1.73054	1.84716
H	-9.10052	4.68051	-0.20031	H	-8.6791	4.66165	-0.44971
H	-9.869	2.10796	1.66853	H	-10.08642	2.92833	0.1064
H	-10.14326	2.71327	0.02546	H	-9.27583	1.38586	0.45433
H	-9.16026	1.25998	0.30368	H	-9.8756	2.36997	1.77879
H	-7.46305	6.36606	0.1529	H	-5.79489	4.5754	0.69764
H	-5.34996	4.13606	0.13543	H	-7.13886	6.47811	-1.30528
H	-5.85374	7.88735	-0.64245	H	-3.01008	6.77456	-0.14142
H	-5.74264	7.85967	1.11814	H	-4.12362	6.23952	1.14236
H	-4.30616	8.28669	0.15054	H	-3.6985	5.12857	-0.16103

atom	Con f. 6- 4g			atom	Con f. 6- 4h		
C	-3.72392	0.15076	4.89602	C	-3.90725	-1.60338	2.15324
C	-2.56209	1.08305	0.81378	C	-6.00179	0.33923	4.54635
C	-3.6097	1.57067	1.49156	C	-5.1631	1.36468	4.36
C	-4.9128	0.77725	1.50629	C	-4.67714	1.90074	3.01833
C	-5.34428	0.23477	2.92068	C	-4.44049	0.87787	1.8533
C	-4.10512	-0.20743	3.66922	C	-3.67298	-0.31208	2.3883
C	-6.07712	1.49474	0.77863	C	-5.58305	3.07325	2.56543
C	-6.8698	2.50697	1.63715	C	-6.90004	2.58232	1.93507
C	-7.35338	1.8339	2.93038	C	-6.62408	1.65346	0.73547
C	-6.16657	1.27029	3.71465	C	-5.75233	0.46515	1.15098
C	-3.485	2.88938	2.21231	C	-4.62433	2.13166	5.54326
C	-6.19832	-1.03792	2.70182	C	-3.50917	1.55367	0.8143
C	-7.98045	3.11412	0.80458	C	-7.85657	3.71898	1.62183
C	-7.82866	4.27393	0.11331	C	-7.64703	4.70726	0.7151
C	-9.27165	2.35403	0.70678	C	-9.12825	3.70266	2.41918
C	-6.63748	5.07834	0.08975	C	-6.49	4.88499	-0.1256
C	-6.47042	6.20729	-0.64062	C	-6.35625	5.89831	-1.0165
C	-5.20665	6.95212	-0.6033	C	-5.18954	6.08623	-1.87596
C	-5.12998	8.19862	-1.4514	C	-4.03571	5.10834	-1.80211
O	-4.24045	6.59032	0.08226	O	-5.14759	7.04124	-2.66466
H	-4.6964	-0.11547	0.9089	H	-3.69145	2.33847	3.22387
H	-6.18729	3.30416	1.93651	H	-7.39276	1.96404	2.69537
H	-2.81738	-0.25349	5.3393	H	-3.25889	-2.3732	2.56386
H	-4.28616	0.85231	5.50527	H	-4.74523	-1.94831	1.55439
H	-1.6146	1.61517	0.76946	H	-6.30759	0.05146	5.54971
H	-2.61524	0.13751	0.2795	H	-6.4005	-0.25788	3.73672
H	-3.48682	-0.91848	3.11987	H	-2.80885	-0.04237	2.99841
H	-6.77687	0.73365	0.41515	H	-5.03551	3.69734	1.85615
H	-5.68118	1.99986	-0.10877	H	-5.80635	3.7137	3.42526
H	-8.06492	1.03457	2.6997	H	-7.57481	1.28553	0.33306

H	-7.88865	2.5626	3.55012	H	-6.14202	2.20838	-0.07368
H	-5.52486	2.10145	4.02278	H	-6.3371	-0.1911	1.80116
H	-6.52632	0.80026	4.63731	H	-5.50498	-0.12922	0.26378
H	-4.15401	3.64952	1.79579	H	-4.79423	3.20969	5.43202
H	-3.72697	2.79545	3.27511	H	-5.08094	1.8083	6.48281
H	-2.4639	3.27383	2.13374	H	-3.53756	1.99658	5.6239
H	-6.56455	-1.42168	3.66	H	-3.32233	0.87274	-0.02216
H	-7.0643	-0.84876	2.06185	H	-3.94104	2.4726	0.40919
H	-5.60313	-1.8258	2.22824	H	-2.54371	1.80987	1.26349
H	-8.66221	4.62441	-0.49384	H	-8.42883	5.45691	0.59886
H	-9.94464	2.80013	-0.02966	H	-8.90347	3.73169	3.4938
H	-9.09408	1.30834	0.42793	H	-9.78711	4.54053	2.17698
H	-9.78894	2.33238	1.67386	H	-9.67407	2.76533	2.24748
H	-5.79086	4.76569	0.69366	H	-5.68123	4.16905	-0.03915
H	-7.26653	6.58862	-1.27538	H	-7.14459	6.63959	-1.13319
H	-5.92034	8.90072	-1.16074	H	-3.25679	5.41965	-2.5001
H	-4.15454	8.6758	-1.34198	H	-3.62023	5.06891	-0.7899
H	-5.30136	7.94626	-2.50465	H	-4.36795	4.09717	-2.05982

atom	Con f. 6- 4i		
C	-3.93174	-1.60625	2.07602
C	-5.94565	0.3144	4.5566
C	-5.10837	1.33746	4.35137
C	-4.66864	1.88779	2.99945
C	-4.4736	0.87794	1.81565
C	-3.68759	-0.31691	2.3117
C	-5.58688	3.0662	2.5904
C	-6.92618	2.58694	1.99823
C	-6.69591	1.6627	0.78481
C	-5.81033	0.47105	1.15882
C	-4.52303	2.08779	5.52316
C	-3.57851	1.56263	0.74996
C	-7.88266	3.73302	1.71843
C	-7.68595	4.72452	0.81114
C	-9.13283	3.72286	2.54843
C	-6.54434	4.88371	-0.04999
C	-6.38009	5.88365	-0.94907
C	-5.17446	5.95481	-1.78294
C	-5.0918	7.10549	-2.75613
O	-4.26168	5.12094	-1.70515
H	-3.67589	2.32242	3.17533
H	-7.40022	1.96869	2.77039
H	-3.26917	-2.3796	2.45619
H	-4.79187	-1.94611	1.5064
H	-6.2161	0.0167	5.56721
H	-6.37722	-0.27119	3.75548
H	-2.8009	-0.05254	2.89106
H	-5.0605	3.69603	1.87099
H	-5.78001	3.69743	3.46433
H	-6.24401	2.22015	-0.0393
H	-7.66196	1.2981	0.41716
H	-6.37055	-0.19129	1.82419
H	-5.59617	-0.11534	0.25775
H	-4.94674	1.75547	6.47494
H	-3.43474	1.9468	5.56252
H	-4.69151	3.16784	5.43067
H	-3.42001	0.88595	-0.09586
H	-4.01853	2.48547	0.36182
H	-2.59799	1.81362	1.16861
H	-8.46159	5.4836	0.71682
H	-9.6904	2.79064	2.3869
H	-8.87972	3.7449	3.61692
H	-9.79102	4.56712	2.32742
H	-5.73987	4.15968	0.00927
H	-7.13501	6.65548	-1.07759
H	-4.16167	7.05953	-3.32517
H	-5.94691	7.0791	-3.44187
H	-5.14809	8.05785	-2.21564

atom	Con f. 7- 1a			atom	Con f. 7- 1b		
C	5.38456	1.08127	1.3788	C	5.88015	0.33752	1.45712
C	2.41631	2.01636	-1.76888	C	2.88565	2.51551	-1.10996
C	2.20361	1.79002	-0.46622	C	2.75391	1.96162	0.10117
C	2.08563	0.39156	0.12265	C	2.44723	0.48376	0.30652

C	3.45772	-0.31179	0.4371	C	3.72531	-0.43605	0.31814
C	4.18121	0.51463	1.47846	C	4.59902	-0.0326	1.48614
C	1.17233	-0.50688	-0.72179	C	1.38132	-0.02808	-0.67825
C	0.87527	-1.82975	0.01899	C	0.96349	-1.48131	-0.35612
C	2.18338	-2.57442	0.28225	C	2.18663	-2.40001	-0.36974
C	3.14053	-1.68948	1.09024	C	3.25346	-1.89052	0.60112
C	4.3125	-0.51217	-0.81914	C	4.49338	-0.38112	-1.00761
C	1.99268	2.92532	0.50038	C	2.87055	2.79362	1.35204
C	-0.15011	-2.63807	-0.75406	C	-0.10428	-1.92096	-1.3402
C	-1.50986	-2.06309	-0.91538	C	-1.48931	-1.42227	-1.19366
O	0.13518	-3.70043	-1.30821	O	0.16468	-2.65226	-2.29642
C	-1.95724	-1.03561	-0.18337	C	-1.9496	-0.72733	-0.14596
C	-3.21859	-0.27933	-0.46291	C	-3.35614	-0.224	-0.04105
O	-2.82004	0.99505	-1.10873	O	-3.28922	1.2327	0.03457
C	-4.15956	-0.11139	0.75799	C	-4.07743	-0.6717	1.25045
C	-5.16756	1.01544	0.51772	C	-5.49855	-0.10439	1.28568
C	-4.87435	-1.44749	0.97197	C	-4.09011	-2.19491	1.33177
O	-3.42268	0.12854	1.96226	O	-3.33351	-0.21487	2.39202
C	-2.02091	1.88309	-0.50274	C	-3.14606	1.91283	-1.12332
C	-1.51404	2.92265	-1.45594	C	-3.02701	3.38666	-0.87099
O	-1.7103	1.84209	0.6831	O	-3.10981	1.37364	-2.21471
H	1.58699	0.50268	1.09534	H	2.0125	0.38636	1.31099
H	0.42514	-1.5686	0.98636	H	0.52996	-1.48669	0.65131
H	5.80269	1.65201	2.20406	H	6.40925	0.58903	2.3728
H	5.99787	1.00454	0.48597	H	6.45059	0.40737	0.53589
H	2.47977	3.02887	-2.16005	H	3.09857	3.5759	-1.22212
H	2.53654	1.21154	-2.48721	H	2.78715	1.94365	-2.02712
H	3.62556	0.63873	2.40953	H	4.09454	-0.07885	2.4528
H	1.6274	-0.73611	-1.69099	H	1.75082	0.00927	-1.70809
H	0.25009	0.03462	-0.93251	H	0.51225	0.63387	-0.63205
H	1.98848	-3.50094	0.83354	H	1.89172	-3.4182	-0.09164
H	2.63471	-2.86737	-0.67186	H	2.58421	-2.45822	-1.38769
H	2.69577	-1.50981	2.0779	H	2.85098	-1.93301	1.62193
H	4.08477	-2.21824	1.26424	H	4.12726	-2.55195	0.58142
H	4.58877	0.4423	-1.27244	H	4.89658	0.61646	-1.19536
H	5.23331	-1.04782	-0.5666	H	5.32849	-1.08875	-0.99036
H	3.78804	-1.09904	-1.57623	H	3.85723	-0.64477	-1.85504
H	2.10128	3.89965	0.01495	H	3.0847	3.8416	1.12232
H	0.98662	2.86649	0.9361	H	1.93904	2.75089	1.93172
H	2.70226	2.87161	1.33384	H	3.66391	2.41618	2.00625
H	-2.11152	-2.49848	-1.71057	H	-2.14827	-1.66857	-2.02353
H	-1.35779	-0.64069	0.62658	H	-1.3106	-0.47705	0.69545
H	-3.78304	-0.75297	-1.26663	H	-3.94495	-0.50605	-0.91548
H	-5.88538	1.03681	1.34285	H	-5.99072	-0.41153	2.21286
H	-4.67018	1.98821	0.47371	H	-5.48554	0.98854	1.24458
H	-5.71668	0.86659	-0.41745	H	-6.08777	-0.47278	0.44009
H	-4.15285	-2.26192	1.08885	H	-4.62771	-2.50933	2.2309
H	-5.5336	-1.68068	0.13158	H	-3.07325	-2.59398	1.37901
H	-5.47767	-1.38958	1.88233	H	-4.5911	-2.62368	0.45933
H	-2.80421	0.85864	1.76587	H	-3.30803	0.75361	2.34928
H	-0.56237	2.56576	-1.86777	H	-3.04122	3.92594	-1.81792
H	-2.21101	3.08293	-2.27969	H	-2.08509	3.58561	-0.34876
H	-1.32814	3.85297	-0.91769	H	-3.84238	3.72788	-0.22729

atom	Con f. 7- 1c			atom	Con f. 7- 1d		
C	5.19136	0.12366	2.2268	C	-5.50815	-1.92896	-1.52976
C	2.32476	2.99265	0.97196	C	-5.0603	1.89437	0.82573
C	2.20015	2.10373	-0.02085	C	-4.47107	1.4902	-0.30585
C	2.22401	0.60849	0.27633	C	-3.22167	0.61928	-0.32161
C	3.6447	-0.07164	0.19596	C	-3.52873	-0.92528	-0.25919
C	4.58461	0.63289	1.15338	C	-4.32509	-1.31452	-1.48573
C	1.18563	-0.13852	-0.57888	C	-2.20285	1.03748	0.75265
C	1.06679	-1.62382	-0.17244	C	-0.88689	0.26325	0.62741
C	2.43394	-2.30287	-0.26293	C	-1.14516	-1.25906	0.69184
C	3.46806	-1.55629	0.58186	C	-2.17114	-1.67482	-0.36335
C	4.27388	0.04515	-1.20834	C	-4.25243	-1.32166	1.03257
C	1.97052	2.56453	-1.4397	C	-5.00643	1.90723	-1.65139
C	0.02283	-2.30692	-1.03835	C	0.09715	0.65217	1.71547
C	-1.4021	-1.93507	-0.89485	C	1.54978	0.48096	1.503
O	0.33825	-3.12802	-1.90291	O	-0.29313	1.07895	2.80558
C	-1.89462	-1.17089	0.08831	C	2.12103	0.02629	0.38052
C	-3.3309	-0.71938	0.12122	C	3.59891	-0.15904	0.23008
O	-3.60926	0.13222	-1.05119	O	4.03716	0.64891	-0.90291
C	-3.75814	-0.09317	1.47234	C	4.00393	-1.61139	-0.11623

C	-5.0621	0.69394	1.32073	C	5.52493	-1.71252	-0.25531
C	-3.92259	-1.23618	2.47686	C	3.48627	-2.56404	0.95698
O	-2.73398	0.75204	2.01477	O	3.36617	-2.00374	-1.34208
C	-2.86633	1.21988	-1.28762	C	4.26044	1.96209	-0.68227
C	-3.14105	1.79479	-2.6433	C	4.68157	2.6563	-1.94366
O	-2.04537	1.67407	-0.49846	O	4.12786	2.48803	0.40827
H	1.92017	0.4914	1.32421	H	-2.73769	0.77663	-1.29575
H	0.71887	-1.65742	0.86872	H	-0.43937	0.48399	-0.34859
H	5.85836	0.73584	2.82894	H	-5.97417	-2.17426	-2.48071
H	5.05986	-0.90364	2.55301	H	-6.05892	-2.20896	-0.63674
H	2.30818	4.06368	0.78414	H	-5.95044	2.51845	0.80354
H	2.44825	2.67908	2.00537	H	-4.68366	1.62472	1.80728
H	4.77861	1.67388	0.90054	H	-3.83466	-1.06659	-2.42857
H	1.44905	-0.08673	-1.64072	H	-2.61065	0.88657	1.75515
H	0.22145	0.36216	-0.4732	H	-2.00807	2.11113	0.65667
H	2.35961	-3.34143	0.07829	H	-0.21147	-1.80705	0.53022
H	2.74686	-2.3434	-1.31149	H	-1.49664	-1.51817	1.69698
H	3.16139	-1.61222	1.63383	H	-1.74485	-1.48982	-1.35804
H	4.43809	-2.06165	0.51024	H	-2.3571	-2.75304	-0.29604
H	4.52463	1.08313	-1.44444	H	-5.23984	-0.85878	1.09351
H	5.20216	-0.53513	-1.2477	H	-4.38209	-2.40781	1.07612
H	3.61308	-0.32843	-1.99424	H	-3.6924	-1.02151	1.92063
H	2.10766	3.64623	-1.52452	H	-5.89868	2.53308	-1.556
H	2.64191	2.07645	-2.15235	H	-4.2478	2.4711	-2.21004
H	0.94751	2.33235	-1.76124	H	-5.2619	1.03551	-2.26319
H	-2.04909	-2.31201	-1.68486	H	2.16292	0.7498	2.36075
H	-1.26151	-0.80643	0.88866	H	1.53127	-0.24374	-0.49044
H	-3.99439	-1.56239	-0.07935	H	4.13033	0.16982	1.12463
H	-5.39798	1.02883	2.30636	H	5.79882	-2.73693	-0.52329
H	-4.91817	1.57652	0.69136	H	5.8943	-1.03908	-1.03403
H	-5.85004	0.07876	0.87482	H	6.02092	-1.45389	0.68542
H	-4.11655	-0.81763	3.46851	H	3.8179	-3.58202	0.73373
H	-3.01452	-1.84497	2.53466	H	2.39375	-2.55716	0.99515
H	-4.75951	-1.88354	2.20139	H	3.86905	-2.28186	1.94176
H	-2.41861	1.3158	1.28349	H	3.70073	-1.42036	-2.04062
H	-2.74271	2.80741	-2.70454	H	5.55845	2.15902	-2.3686
H	-2.64748	1.16482	-3.39214	H	4.90816	3.70106	-1.73224
H	-4.21281	1.78828	-2.85446	H	3.8748	2.5929	-2.68087

atom	Con f. 7- 1e			atom	Con f. 7- 1f		
C	6.14064	2.01297	-0.62551	C	5.0831	-0.12216	2.36169
C	5.09821	-1.86512	1.39333	C	2.42255	2.88049	0.88283
C	4.91133	-1.46984	0.12842	C	2.31291	1.95945	-0.08363
C	3.70334	-0.6538	-0.3123	C	2.23801	0.47851	0.27047
C	3.89132	0.90104	-0.13253	C	3.60606	-0.30288	0.27811
C	5.04303	1.3556	-1.00255	C	4.55357	0.37834	1.24427
C	2.39723	-1.1456	0.33598	C	1.18872	-0.23052	-0.59802
C	1.1739	-0.42724	-0.23123	C	0.93253	-1.67768	-0.12671
C	1.31249	1.10444	-0.0666	C	2.24382	-2.46346	-0.1286
C	2.61846	1.59396	-0.6945	C	3.30244	-1.75229	0.72022
C	4.10206	1.29362	1.33415	C	4.29887	-0.29654	-1.1009
C	5.88986	-1.84615	-0.95408	C	2.17938	2.37565	-1.52874
C	-0.1216	-0.87377	0.41123	C	-0.1409	-2.31157	-0.99719
C	-1.36728	-0.47382	-0.28967	C	-1.48262	-1.68309	-1.02721
O	-0.14732	-1.49325	1.47558	O	0.10293	-3.2635	-1.74054
C	-2.57146	-0.60922	0.27649	C	-1.94989	-0.87266	-0.07115
C	-3.84523	-0.17984	-0.38219	C	-3.20923	-0.06973	-0.17661
O	-4.37746	0.94636	0.38242	O	-2.82433	1.31756	0.08971
C	-4.9523	-1.25612	-0.35727	C	-4.27535	-0.40738	0.88941
C	-6.21291	-0.73446	-1.05109	C	-5.50133	0.49276	0.7177
C	-4.4455	-2.53209	-1.0227	C	-4.65456	-1.88119	0.78733
O	-5.24628	-1.60255	1.00557	O	-3.70778	-0.2285	2.19663
C	-3.88092	2.17281	0.1147	C	-2.10393	1.96999	-0.84735
C	-4.50198	3.21091	1.00183	C	-1.60075	3.28727	-0.33561
O	-3.0362	2.38178	-0.73814	O	-1.8685	1.50983	-1.95112
H	3.59602	-0.80644	-1.39535	H	1.88438	0.42468	1.30801
H	1.09115	-0.634	-1.30819	H	0.54769	-1.62916	0.90159
H	6.89523	2.30195	-1.35261	H	5.7649	0.47099	2.96624
H	6.33592	2.28692	0.40695	H	4.87169	-1.12446	2.72253
H	5.96973	-2.45065	1.676	H	2.4696	3.94279	0.65536
H	4.39885	-1.62614	2.18817	H	2.47866	2.6015	1.93191
H	4.91978	1.11665	-2.06023	H	4.82415	1.39354	0.95786
H	2.4225	-1.00161	1.41962	H	1.50342	-0.25371	-1.64687
H	2.30322	-2.22418	0.17077	H	0.26535	0.34759	-0.56847

H	0.46461	1.61771	-0.5328	H	2.07889	-3.47436	0.2599
H	1.28179	1.34995	1.00125	H	2.5909	-2.5805	-1.16084
H	2.57373	1.41752	-1.77704	H	2.9547	-1.73357	1.76067
H	2.71526	2.67687	-0.55475	H	4.23301	-2.3312	0.71074
H	5.03339	0.88022	1.72743	H	4.62302	0.71111	-1.37637
H	4.14427	2.38314	1.43305	H	5.1906	-0.93197	-1.07005
H	3.28883	0.93691	1.96951	H	3.64996	-0.67136	-1.8961
H	6.72314	-2.43539	-0.55985	H	2.3541	3.44925	-1.64178
H	5.39187	-2.4343	-1.73617	H	2.87668	1.84335	-2.18241
H	6.30012	-0.95718	-1.44522	H	1.17156	2.16011	-1.90419
H	-1.26629	-0.03391	-1.27779	H	-2.06804	-1.88436	-1.92221
H	-2.65796	-1.02764	1.27627	H	-1.38449	-0.69498	0.83896
H	-3.66604	0.14837	-1.40732	H	-3.64283	-0.13472	-1.17653
H	-6.99636	-1.4962	-1.00413	H	-6.23633	0.25825	1.49295
H	-6.58122	0.17292	-0.56389	H	-5.22882	1.54835	0.8059
H	-6.01433	-0.50328	-2.1023	H	-5.96645	0.33908	-0.26109
H	-5.24133	-3.28238	-1.03093	H	-5.43328	-2.11057	1.52025
H	-3.58874	-2.94392	-0.48217	H	-3.79098	-2.52135	0.98638
H	-4.14295	-2.33514	-2.05503	H	-5.03726	-2.11427	-0.2103
H	-5.56037	-0.79676	1.44439	H	-3.45154	0.70376	2.27227
H	-4.14306	4.20045	0.7196	H	-1.39321	3.9518	-1.17501
H	-4.23665	3.0034	2.04351	H	-2.31366	3.74547	0.35255
H	-5.59205	3.16779	0.92242	H	-0.66535	3.10779	0.20872

atom	Con f. 7- 1g			atom	Con f. 7- 1h		
C	6.72581	-0.45836	0.3759	C	5.82784	0.44043	1.38491
C	3.56256	2.73282	-0.08503	C	2.97941	2.05556	-1.67834
C	3.69264	1.75197	0.81634	C	2.79476	1.8096	-0.3759
C	3.1924	0.33328	0.5763	C	2.42029	0.43542	0.16269
C	4.23064	-0.5888	-0.16832	C	3.65546	-0.50639	0.42321
C	5.46146	-0.73197	0.70094	C	4.53363	0.13151	1.47786
C	1.81366	0.31308	-0.10834	C	1.34916	-0.25313	-0.70037
C	1.25111	-1.1232	-0.21286	C	0.85849	-1.56732	-0.0493
C	2.23122	-2.03206	-0.95215	C	2.03631	-2.51272	0.19393
C	3.60474	-2.00782	-0.27946	C	3.11413	-1.8281	1.03727
C	4.58225	-0.05695	-1.56203	C	4.43859	-0.80374	-0.86023
C	4.32713	2.01163	2.15832	C	2.91401	2.90955	0.64702
C	-0.11585	-1.07001	-0.86161	C	-0.21552	-2.18805	-0.9248
C	-1.20553	-0.46706	-0.05261	C	-1.55144	-1.56032	-0.99034
O	-0.31159	-1.47832	-2.00711	O	0.0191	-3.17182	-1.63159
C	-2.41738	-0.23322	-0.56849	C	-1.97758	-0.59697	-0.16329
C	-3.53915	0.39412	0.19697	C	-3.32513	0.04852	-0.2398
O	-4.64121	-0.56261	0.23509	O	-3.20911	1.43554	0.1879
C	-4.1104	1.6631	-0.476	C	-4.39748	-0.5684	0.69925
C	-5.25364	2.23559	0.36586	C	-4.76675	-1.96651	0.21754
C	-2.99884	2.69098	-0.66577	C	-3.93372	-0.58233	2.15752
O	-4.58189	1.3338	-1.79221	O	-5.58757	0.22072	0.56545
C	-4.61016	-1.50796	1.1986	C	-2.50116	2.27266	-0.59925
C	-5.78795	-2.43042	1.08788	C	-2.4385	3.65053	-0.01089
O	-3.72689	-1.58161	2.03432	O	-1.97443	1.9191	-1.63965
H	3.05462	-0.12688	1.5646	H	1.96539	0.59536	1.15033
H	1.10693	-1.49316	0.81292	H	0.40954	-1.31056	0.91856
H	7.53225	-0.62202	1.08619	H	6.35683	0.88508	2.22399
H	7.01111	-0.06432	-0.59501	H	6.4098	0.26901	0.48418
H	3.92762	3.73595	0.12166	H	3.24051	3.05083	-2.0298
H	3.09308	2.58026	-1.05161	H	2.87799	1.28691	-2.43791
H	5.25575	-1.12632	1.69749	H	4.01846	0.33508	2.41817
H	1.87113	0.73907	-1.115	H	1.73943	-0.48134	-1.69739
H	1.12998	0.94877	0.46276	H	0.5137	0.43729	-0.84235
H	1.84747	-3.05825	-0.97213	H	1.68923	-3.41811	0.70449
H	2.30656	-1.70826	-1.99533	H	2.44576	-2.83629	-0.76814
H	3.51032	-2.42612	0.73139	H	2.69768	-1.60555	2.02873
H	4.30222	-2.65577	-0.82264	H	3.95636	-2.51097	1.19746
H	5.08933	0.9086	-1.50435	H	4.87827	0.1038	-1.27971
H	5.24384	-0.75923	-2.07923	H	5.24882	-1.51041	-0.65377
H	3.69306	0.07384	-2.18204	H	3.80264	-1.24833	-1.62854
H	3.61773	1.79745	2.96846	H	3.6614	2.6626	1.40864
H	5.19215	1.35954	2.31892	H	3.19338	3.86215	0.18734
H	4.65694	3.05017	2.25561	H	1.96145	3.04815	1.17551
H	-0.98233	-0.20775	0.97872	H	-2.19278	-1.93454	-1.78548
H	-2.62634	-0.49707	-1.60236	H	-1.343	-0.2285	0.63809
H	-3.23711	0.62246	1.22035	H	-3.71229	0.03363	-1.2615
H	-5.66351	3.12074	-0.12898	H	-5.58442	-2.35384	0.83226
H	-6.05653	1.50265	0.48686	H	-5.09913	-1.94204	-0.82478

H	-4.90046	2.52496	1.36059	H	-3.91935	-2.65012	0.30016
H	-3.41308	3.60048	-1.1101	H	-3.66842	0.42409	2.49494
H	-2.21744	2.30772	-1.32742	H	-3.06178	-1.23046	2.28856
H	-2.54385	2.94964	0.29454	H	-4.74181	-0.95897	2.79078
H	-5.29324	0.68307	-1.68817	H	-5.35708	1.12839	0.81799
H	-6.71728	-1.85357	1.09683	H	-3.43946	3.99175	0.26638
H	-5.74	-2.9675	0.13517	H	-1.83204	3.62182	0.90074
H	-5.77871	-3.14143	1.91374	H	-1.98774	4.33711	-0.72711

atom	Con f. 7- 1i			atom	Con f. 7- 1j		
C	-4.21699	2.68204	1.84516	C	3.5529	3.11066	1.47146
C	-5.01866	-1.52453	1.921	C	5.25052	1.00159	-1.83698
C	-4.49239	-1.38729	0.69784	C	4.65886	-0.00397	-1.18077
C	-3.26883	-0.50463	0.483	C	3.26818	0.17972	-0.58471
C	-3.58424	0.97813	0.04273	C	3.24527	0.6279	0.92876
C	-4.48077	1.61975	1.08238	C	4.01466	1.92619	1.06709
C	-2.25351	-1.17592	-0.45959	C	2.38853	-1.06093	-0.82526
C	-0.94691	-0.38491	-0.55209	C	0.94183	-0.83486	-0.38518
C	-1.23124	1.06484	-1.00459	C	0.89589	-0.42081	1.10429
C	-2.23961	1.72714	-0.06561	C	1.76921	0.81247	1.33723
C	-4.34003	1.05153	-1.30174	C	3.92956	-0.39395	1.86184
C	-5.06887	-2.14497	-0.47368	C	5.33658	-1.34782	-1.06554
C	0.03486	-1.02469	-1.51501	C	0.08268	-2.06706	-0.60457
C	1.48575	-0.77895	-1.37	C	-1.39075	-1.94332	-0.57437
O	-0.35548	-1.72106	-2.4559	O	0.58258	-3.18468	-0.75478
C	2.05791	-0.08803	-0.37585	C	-2.05224	-0.78124	-0.50194
C	3.53512	0.13898	-0.27692	C	-3.53658	-0.69583	-0.33453
O	4.0068	-0.53083	0.93208	O	-4.10671	0.20597	-1.34414
C	3.91574	1.6268	-0.09987	C	-3.94713	-0.3671	1.13713
C	5.43633	1.77512	-0.01392	C	-5.37862	0.17439	1.19218
C	3.34755	2.44734	-1.25374	C	-3.82618	-1.64993	1.96062
O	3.30025	2.13478	1.09469	O	-3.03595	0.56199	1.73853
C	4.26864	-1.85305	0.84918	C	-3.81064	1.51163	-1.3809
C	4.717	-2.39738	2.17319	C	-4.3951	2.16736	-2.59575
O	4.14614	-2.49483	-0.17855	O	-3.15014	2.09723	-0.53119
H	-2.78425	-0.40191	1.46223	H	2.80513	1.01538	-1.12472
H	-0.49073	-0.33959	0.44318	H	0.51724	-0.01019	-0.97177
H	-4.95649	3.05698	2.54854	H	4.21572	3.96966	1.54189
H	-3.27523	3.22133	1.80839	H	2.51666	3.28153	1.74759
H	-5.88972	-2.15024	2.10106	H	6.24115	0.89462	-2.27273
H	-4.59727	-1.01371	2.78317	H	4.76483	1.96659	-1.95789
H	-5.45434	1.14343	1.18492	H	5.06995	1.84613	0.8118
H	-2.67296	-1.28927	-1.46254	H	2.78972	-1.92746	-0.29259
H	-2.04373	-2.18775	-0.0941	H	2.41314	-1.3146	-1.89106
H	-0.30313	1.64514	-1.01603	H	-0.13148	-0.20354	1.41042
H	-1.6087	1.047	-2.03363	H	1.23876	-1.2629	1.71705
H	-1.78806	1.79094	0.93211	H	1.34375	1.64442	0.76226
H	-2.42719	2.75547	-0.39493	H	1.72234	1.10122	2.39348
H	-5.36002	0.66961	-1.20541	H	5.00922	-0.42495	1.69225
H	-4.40981	2.09425	-1.6304	H	3.76952	-0.10559	2.90644
H	-3.84161	0.48467	-2.09206	H	3.53911	-1.40603	1.73056
H	-5.99147	-2.65818	-0.18816	H	6.354	-1.30528	-1.4645
H	-5.28878	-1.49421	-1.3248	H	5.38984	-1.70163	-0.03185
H	-4.36444	-2.90267	-0.83733	H	4.78907	-2.11403	-1.62726
H	2.09601	-1.21787	-2.15645	H	-1.9277	-2.88951	-0.58753
H	1.46974	0.34703	0.42681	H	-1.52013	0.16266	-0.50635
H	4.05742	-0.28115	-1.13784	H	-4.00615	-1.64331	-0.60128
H	5.69145	2.82718	0.14206	H	-5.67944	0.28155	2.23843
H	5.84111	1.19182	0.81816	H	-5.45294	1.1557	0.71706
H	5.91518	1.43361	-0.93695	H	-6.07995	-0.50411	0.69675
H	3.65981	3.4905	-1.15014	H	-4.03604	-1.41995	3.00905
H	2.2546	2.41367	-1.2595	H	-2.81712	-2.06582	1.8972
H	3.71148	2.06933	-2.21323	H	-4.54041	-2.40449	1.62035
H	3.6599	1.62963	1.84015	H	-2.97925	1.32049	1.12601
H	4.96775	-3.45315	2.07268	H	-4.20568	3.23989	-2.56441
H	5.58518	-1.83664	2.53189	H	-3.94484	1.73451	-3.49463
H	3.91545	-2.27384	2.90836	H	-5.47096	1.97437	-2.63801

atom	Con f. 7- 1k			atom	Con f. 7- 1l		
C	6.03469	0.65854	1.37024	C	-5.39851	-1.57511	-2.01194
C	2.98522	2.48871	-1.41703	C	-5.11897	1.53895	1.22912
C	2.87164	2.05444	-0.15619	C	-4.49789	1.45763	0.04648
C	2.60176	0.59787	0.19697	C	-3.21779	0.65837	-0.16124

C	3.90446	-0.28032	0.31152	C	-3.47199	-0.86229	-0.48908
C	4.76161	0.26554	1.43281	C	-4.23595	-0.95946	-1.79172
C	1.56131	-0.04262	-0.73842	C	-2.22629	0.83066	1.00263
C	1.17757	-1.46573	-0.27167	C	-0.88322	0.15516	0.70952
C	2.4248	-2.34682	-0.17195	C	-1.09199	-1.34502	0.4032
C	3.46802	-1.70789	0.74625	C	-2.08877	-1.52406	-0.74234
C	4.67689	-0.34558	-1.01105	C	-4.19997	-1.58684	0.64887
C	2.97444	3.00903	1.00528	C	-5.02582	2.19191	-1.15892
C	0.14031	-2.04059	-1.21884	C	0.08975	0.29888	1.8646
C	-1.26351	-1.57814	-1.14673	C	1.54442	0.2058	1.6228
O	0.44684	-2.86377	-2.08453	O	-0.30989	0.44995	3.02239
C	-1.75399	-0.74607	-0.21969	C	2.12103	0.08151	0.41987
C	-3.18697	-0.31504	-0.18089	C	3.59585	-0.07164	0.22699
O	-3.09351	1.14897	-0.24068	O	4.01714	0.74169	-0.90495
C	-3.95648	-0.80753	1.08068	C	4.05036	-1.51567	-0.11946
C	-3.62969	-2.27933	1.33857	C	3.82197	-2.43006	1.07898
C	-3.6291	0.05113	2.30522	C	3.35567	-2.05119	-1.37394
O	-5.36713	-0.77553	0.82039	O	5.4703	-1.47695	-0.32037
C	-4.13197	1.86773	-0.68857	C	4.14164	2.07121	-0.69855
C	-3.75867	3.30437	-0.89303	C	4.5995	2.77041	-1.94401
O	-5.2412	1.39567	-0.90266	O	3.90318	2.6027	0.37056
H	2.16046	0.59208	1.20338	H	-2.72948	1.06804	-1.05646
H	0.72628	-1.37947	0.72465	H	-0.44379	0.61946	-0.18106
H	6.55183	1.01648	2.25685	H	-5.84184	-1.59141	-3.00431
H	6.61031	0.64364	0.44962	H	-5.95371	-2.08041	-1.2273
H	3.17363	3.53739	-1.63382	H	-6.0301	2.12066	1.34634
H	2.89505	1.82765	-2.27316	H	-4.74881	1.03669	2.11719
H	4.25227	0.31065	2.39691	H	-3.73929	-0.47386	-2.63343
H	1.94224	-0.1016	-1.76293	H	-2.63718	0.41928	1.92798
H	0.67527	0.59708	-0.76954	H	-2.07108	1.90031	1.18016
H	2.15237	-3.33642	0.21197	H	-0.13928	-1.81326	0.13629
H	2.83702	-2.50404	-1.1734	H	-1.449	-1.84583	1.31057
H	3.05817	-1.65223	1.76347	H	-1.65446	-1.0911	-1.65304
H	4.35848	-2.34456	0.80282	H	-2.23937	-2.59144	-0.94176
H	5.05118	0.6378	-1.30435	H	-5.20282	-1.18238	0.8022
H	5.53247	-1.02181	-0.91597	H	-4.2933	-2.65354	0.42095
H	4.05318	-0.71804	-1.82647	H	-3.66076	-1.49858	1.5942
H	3.77943	2.71676	1.68798	H	-5.94381	2.74084	-0.92884
H	3.16273	4.03366	0.67134	H	-4.28079	2.90731	-1.53136
H	2.04687	3.00279	1.59278	H	-5.23561	1.50173	-1.98305
H	-1.90935	-1.97696	-1.92629	H	2.15399	0.24201	2.52338
H	-1.12226	-0.33908	0.56496	H	1.52736	0.06249	-0.48978
H	-3.72462	-0.66311	-1.06623	H	4.14449	0.25594	1.11238
H	-4.30251	-2.65417	2.11556	H	4.22975	-3.42106	0.85964
H	-3.78041	-2.87734	0.43443	H	4.32558	-2.03693	1.96749
H	-2.60016	-2.41292	1.67873	H	2.75728	-2.53494	1.29812
H	-3.9644	1.08184	2.16246	H	3.76786	-3.0331	-1.6227
H	-2.55383	0.06387	2.51118	H	3.51298	-1.38263	-2.22561
H	-4.14259	-0.3592	3.17956	H	2.27843	-2.1605	-1.21678
H	-5.55207	0.02634	0.29675	H	5.63931	-0.85407	-1.04434
H	-4.64584	3.88639	-1.14098	H	4.70388	3.8378	-1.75091
H	-3.28743	3.69815	0.01194	H	3.87326	2.60505	-2.74585
H	-3.02787	3.37543	-1.7052	H	5.55632	2.35341	-2.27254

atom	Con f. 7- 1m			atom	Con f. 7- 1n		
C	-5.28585	2.26194	1.80166	C	-6.78104	-0.25125	-0.0422
C	-5.87286	-1.83755	0.79032	C	-3.36678	2.43229	1.11712
C	-4.94175	-1.49335	-0.10759	C	-3.66121	1.86616	-0.05922
C	-3.76519	-0.6157	0.30273	C	-3.24508	0.44685	-0.42242
C	-3.96871	0.9307	0.05572	C	-4.25824	-0.65711	0.06325
C	-5.20898	1.38746	0.7972	C	-5.5899	-0.42403	-0.61695
C	-2.44694	-1.12253	-0.31121	C	-1.80424	0.13435	0.02159
C	-1.23484	-0.35582	0.21206	C	-1.33897	-1.25242	-0.4794
C	-1.40418	1.1605	-0.03778	C	-2.30117	-2.34673	-0.02047
C	-2.71153	1.65509	0.58248	C	-3.7349	-2.02776	-0.44994
C	-4.19013	1.27192	-1.43341	C	-4.39986	-0.68403	1.58934
C	-5.01504	-2.00934	-1.52407	C	-4.40954	2.62819	-1.1223
C	0.06614	-0.82007	-0.40651	C	0.08532	-1.48069	-0.01818
C	1.30754	-0.36981	0.27164	C	1.1234	-0.62117	-0.64256
O	0.09905	-1.50223	-1.43163	O	0.36808	-2.30573	0.85114
C	2.51892	-0.59479	-0.24884	C	2.36452	-0.5511	-0.14772
C	3.79222	-0.14949	0.39926	C	3.41577	0.35663	-0.70591
O	4.42121	0.83125	-0.48175	O	4.63225	-0.41724	-0.9962
C	4.82972	-1.28238	0.56792	C	3.64956	1.63762	0.1546

C	6.09533	-0.74411	1.23943	C	4.9893	2.28929	-0.20256
C	4.21971	-2.42152	1.37949	C	2.49386	2.60324	-0.11345
O	5.14797	-1.83313	-0.72003	O	3.58845	1.34816	1.55466
C	3.98316	2.10609	-0.40725	C	5.35586	-1.00105	-0.03231
C	4.71646	2.98515	-1.37673	C	6.43704	-1.86354	-0.61103
O	3.10765	2.46847	0.3584	O	5.16165	-0.85277	1.16812
H	-3.67249	-0.70777	1.39225	H	-3.25316	0.38392	-1.51942
H	-1.14623	-0.4989	1.29881	H	-1.32141	-1.21351	-1.57854
H	-6.24643	2.51563	2.24334	H	-7.67527	-0.10972	-0.64381
H	-4.41843	2.7595	2.22497	H	-6.91828	-0.24177	1.03501
H	-6.72072	-2.46372	0.52234	H	-3.67558	3.4501	1.34264
H	-5.82023	-1.50167	1.8229	H	-2.81298	1.91253	1.89248
H	-6.13296	0.93887	0.43642	H	-5.53562	-0.42135	-1.70695
H	-2.47259	-1.04191	-1.40176	H	-1.71797	0.16059	1.11257
H	-2.33407	-2.18854	-0.08339	H	-1.14363	0.91731	-0.36311
H	-0.56122	1.71494	0.38788	H	-1.99582	-3.31198	-0.43974
H	-1.3913	1.34557	-1.11825	H	-2.23896	-2.44827	1.06791
H	-2.64968	1.51406	1.66853	H	-3.78501	-2.02967	-1.54685
H	-2.8172	2.73197	0.40762	H	-4.41473	-2.8149	-0.10406
H	-5.16038	0.90971	-1.78388	H	-4.82841	0.24695	1.96697
H	-4.17763	2.35884	-1.56895	H	-5.05293	-1.50814	1.89394
H	-3.41842	0.84537	-2.07881	H	-3.4369	-0.82844	2.08321
H	-5.94988	-2.55139	-1.69224	H	-4.66531	3.63911	-0.79159
H	-4.94743	-1.20706	-2.26445	H	-3.80745	2.70599	-2.0371
H	-4.18728	-2.69654	-1.73612	H	-5.33543	2.11325	-1.40014
H	1.1986	0.16658	1.21018	H	0.82958	-0.02104	-1.49974
H	2.61445	-1.11848	-1.19685	H	2.62679	-1.15058	0.71865
H	3.5968	0.32243	1.36372	H	3.13149	0.68658	-1.70596
H	6.82873	-1.54998	1.33402	H	5.07364	3.2436	0.32548
H	6.54004	0.06245	0.64942	H	5.83152	1.66123	0.09917
H	5.87315	-0.35632	2.23855	H	5.06718	2.47925	-1.27766
H	4.96602	-3.20759	1.5262	H	2.60813	3.48066	0.52936
H	3.35852	-2.85488	0.86363	H	1.53216	2.13581	0.1146
H	3.89336	-2.06506	2.36059	H	2.48793	2.93429	-1.1554
H	5.53871	-1.12091	-1.24957	H	4.19797	0.59962	1.70192
H	4.34288	4.00635	-1.30498	H	7.08946	-2.22187	0.18477
H	5.78836	2.96281	-1.15723	H	7.01253	-1.29715	-1.34839
H	4.5788	2.60623	-2.39401	H	5.98092	-2.71437	-1.12758
atom	Con f. 7- 1o			atom	Con f. 7- 1p		
C	6.30822	1.60942	-1.23047	C	5.5478	1.80123	-1.77249
C	5.04679	-1.32382	1.95349	C	5.2835	-1.78431	0.90645
C	4.882	-1.34648	0.62557	C	4.64263	-1.51039	-0.23614
C	3.72095	-0.65314	-0.07538	C	3.36563	-0.68225	-0.29106
C	4.00264	0.85992	-0.4163	C	3.62194	0.8705	-0.37143
C	5.16898	0.93173	-1.37838	C	4.37804	1.17276	-1.64726
C	2.39228	-0.82786	0.6808	C	2.38784	-1.03744	0.84234
C	1.20894	-0.26255	-0.10405	C	1.04399	-0.32099	0.67931
C	1.44067	1.22735	-0.44482	C	1.25067	1.20897	0.60681
C	2.76715	1.40486	-1.18589	C	2.23832	1.56516	-0.50525
C	4.25486	1.70167	0.83949	C	4.36103	1.40197	0.8619
C	5.83856	-2.10087	-0.26161	C	5.14332	-2.04438	-1.55323
C	-0.10471	-0.40783	0.63439	C	0.09052	-0.64202	1.81486
C	-1.3361	-0.22579	-0.17254	C	-1.369	-0.49999	1.62227
O	-0.15185	-0.63754	1.84422	O	0.5077	-0.98394	2.92439
C	-2.55199	-0.26982	0.38511	C	-1.97076	-0.19777	0.46526
C	-3.82772	-0.15034	-0.38279	C	-3.45256	-0.03477	0.33392
O	-4.76295	0.67721	0.36645	O	-3.80602	-1.00495	-0.70888
C	-4.57033	-1.49452	-0.61344	C	-3.8917	1.41709	-0.03069
C	-3.75631	-2.37794	-1.55228	C	-3.08254	2.4238	0.78886
C	-4.88132	-2.21195	0.70179	C	-3.74014	1.68535	-1.53055
O	-5.7868	-1.19158	-1.31109	O	-5.25383	1.61573	0.37255
C	-4.57309	2.01437	0.32721	C	-5.0634	-1.45706	-0.80404
C	-5.62983	2.73351	1.11188	C	-5.16844	-2.58884	-1.78053
O	-3.65632	2.53783	-0.27971	O	-6.00118	-0.99641	-0.1655
H	3.59801	-1.14795	-1.04907	H	2.86268	-0.93921	-1.23408
H	1.10683	-0.8029	-1.05634	H	0.58787	-0.64087	-0.26493
H	7.06979	1.59607	-2.00602	H	5.98612	1.97455	-2.75201
H	6.53216	2.1964	-0.34477	H	6.11376	2.16409	-0.91979
H	5.88481	-1.83202	2.42434	H	6.19149	-2.38256	0.91109
H	4.36138	-0.80663	2.61737	H	4.93337	-1.43103	1.8712
H	5.01888	0.36443	-2.29846	H	3.86976	0.83794	-2.5531
H	2.43396	-0.34206	1.65944	H	2.81221	-0.78378	1.81667
H	2.23228	-1.89405	0.8739	H	2.22901	-2.12125	0.84666
H	0.62202	1.61015	-1.06326	H	0.2951	1.71069	0.42404
H	1.43516	1.80916	0.48401	H	1.61539	1.56456	1.57713

H	2.69977	0.88622	-2.15115	H	1.79705	1.28117	-1.46956
H	2.93016	2.46583	-1.40802	H	2.38971	2.65044	-0.53454
H	5.15894	1.37885	1.36031	H	5.36049	0.96942	0.94463
H	4.37474	2.75601	0.5703	H	4.46459	2.49018	0.80118
H	3.42573	1.6357	1.54709	H	3.82521	1.17442	1.78571
H	6.29268	-1.43987	-1.00752	H	5.34594	-1.23182	-2.25905
H	6.64096	-2.57053	0.31497	H	6.0598	-2.62921	-1.43124
H	5.30961	-2.88536	-0.81878	H	4.38515	-2.68532	-2.02229
H	-1.21874	-0.05706	-1.23955	H	-1.95845	-0.66393	2.522
H	-2.64202	-0.42467	1.45834	H	-1.39873	-0.04439	-0.44571
H	-3.65812	0.31536	-1.35577	H	-3.96158	-0.32067	1.25775
H	-4.33146	-3.27706	-1.79173	H	-3.52388	3.41563	0.65366
H	-3.53307	-1.85091	-2.48513	H	-3.11151	2.17571	1.85426
H	-2.81561	-2.68218	-1.08851	H	-2.03923	2.46348	0.46687
H	-5.46295	-3.1142	0.49261	H	-4.40877	1.04695	-2.11435
H	-5.46403	-1.57115	1.37024	H	-2.71361	1.50877	-1.86776
H	-3.96357	-2.50522	1.22012	H	-3.99635	2.72863	-1.73639
H	-6.28881	-0.57769	-0.75246	H	-5.74343	0.80116	0.15262
H	-6.61021	2.54458	0.66289	H	-4.72658	-2.29543	-2.73707
H	-5.65572	2.3523	2.13686	H	-4.60241	-3.44663	-1.40331
H	-5.42404	3.80364	1.1142	H	-6.21365	-2.86479	-1.91668

atom	Con f. 7- 1q			atom	Con f. 7- 1r		
C	6.13266	-1.69189	1.0251	C	6.7289	-0.43877	0.3541
C	4.33479	1.99645	2.15414	C	3.55635	2.58862	-0.64064
C	3.74452	1.78972	0.9707	C	3.70683	1.81334	0.43974
C	3.25976	0.39591	0.58976	C	3.20179	0.37751	0.50429
C	4.28361	-0.46353	-0.24989	C	4.21902	-0.67852	-0.07146
C	5.57538	-0.58407	0.53343	C	5.47761	-0.64319	0.76815
C	1.87368	0.45342	-0.08056	C	1.80439	0.22737	-0.12445
C	1.29887	-0.95767	-0.3373	C	1.23913	-1.19935	0.06928
C	2.2758	-1.79159	-1.164	C	2.20294	-2.24424	-0.49002
C	3.6444	-1.84916	-0.48443	C	3.59339	-2.08744	0.12869
C	4.64152	0.18954	-1.60228	C	4.52326	-0.4425	-1.55568
C	3.5052	2.93833	0.02088	C	4.36985	2.33824	1.68687
C	-0.06064	-0.81616	-0.98922	C	-0.13747	-1.26267	-0.55899
C	-1.16696	-0.35666	-0.11256	C	-1.21561	-0.53282	0.1511
O	-0.23594	-1.04498	-2.18695	O	-0.34624	-1.85911	-1.61681
C	-2.39397	-0.11916	-0.58997	C	-2.44059	-0.40362	-0.37221
C	-3.53951	0.33603	0.25773	C	-3.54274	0.37223	0.27217
O	-4.56604	-0.70219	0.20946	O	-4.80765	-0.31569	0.05851
C	-4.22717	1.61849	-0.26376	C	-3.75325	1.79661	-0.31122
C	-5.37314	2.01771	0.66856	C	-2.55191	2.67518	0.01993
C	-3.20179	2.74141	-0.38716	C	-4.02538	1.76338	-1.81642
O	-4.72708	1.3823	-1.58957	O	-4.86867	2.37666	0.3799
C	-4.43315	-1.74945	1.05112	C	-5.05938	-1.39923	0.82482
C	-5.54704	-2.73646	0.86285	C	-6.40121	-1.98755	0.50401
O	-3.51831	-1.85396	1.84844	O	-4.27675	-1.82053	1.65719
H	3.13807	-0.16053	1.52745	H	3.09291	0.1252	1.56808
H	1.14399	-1.43446	0.6412	H	1.11164	-1.35711	1.14999
H	7.07575	-1.64576	1.56416	H	7.55864	-0.45492	1.05615
H	5.69053	-2.67776	0.91754	H	6.97979	-0.24731	-0.68489
H	4.68959	2.98072	2.45089	H	3.92445	3.6116	-0.65324
H	4.48597	1.18733	2.86412	H	3.06638	2.24038	-1.54432
H	6.09166	0.3619	0.68798	H	5.3069	-0.82931	1.82989
H	1.92791	0.98537	-1.03617	H	1.83453	0.44836	-1.19608
H	1.1948	1.02612	0.56043	H	1.13561	0.9651	0.33009
H	1.88294	-2.80663	-1.2913	H	1.82008	-3.25078	-0.28722
H	2.35699	-1.36286	-2.16803	H	2.25124	-2.14448	-1.57904
H	3.52892	-2.35341	0.48318	H	3.52561	-2.28726	1.2062
H	4.33007	-2.46297	-1.07945	H	4.27702	-2.83684	-0.28646
H	5.23549	1.09647	-1.46058	H	5.02407	0.51525	-1.71318
H	5.24157	-0.50525	-2.1999	H	5.17441	-1.23473	-1.939
H	3.7563	0.45353	-2.18611	H	3.61457	-0.44327	-2.16104
H	3.9922	3.84846	0.38204	H	4.7005	3.37385	1.56429
H	3.87362	2.72999	-0.98769	H	3.6782	2.29519	2.53847
H	2.4337	3.15014	-0.07813	H	5.23932	1.73003	1.95899
H	-0.94708	-0.22296	0.94308	H	-0.97368	-0.09358	1.11513
H	-2.60246	-0.26311	-1.64735	H	-2.66164	-0.85588	-1.33688
H	-3.22994	0.47456	1.2949	H	-3.37849	0.46716	1.34769
H	-5.87097	2.90751	0.27278	H	-2.75498	3.70005	-0.30386
H	-6.11071	1.21421	0.74982	H	-2.36327	2.68389	1.09793
H	-5.00009	2.24463	1.6721	H	-1.65292	2.32335	-0.49037
H	-3.69874	3.65667	-0.72147	H	-4.23632	2.77707	-2.16864

H	-2.42433	2.48552	-1.11214	H	-4.88717	1.12955	-2.04536
H	-2.72573	2.93743	0.57776	H	-3.16096	1.37998	-2.36676
H	-5.37113	0.6597	-1.52817	H	-5.6306	1.797	0.22432
H	-5.4457	-3.54922	1.5817	H	-6.42643	-2.29028	-0.5474
H	-6.51218	-2.23772	0.99186	H	-6.58954	-2.84939	1.14372
H	-5.51306	-3.13428	-0.15632	H	-7.18091	-1.23392	0.65011

atom	Con f. 7- 1s			atom	Con f. 7- 1t		
C	5.1739	-0.37186	2.3119	C	5.69348	-1.14984	2.38562
C	2.74643	2.59976	-1.12997	C	5.09668	1.04488	-2.23173
C	2.6235	2.02225	0.07103	C	4.94241	1.24041	-0.91689
C	2.41267	0.52581	0.25655	C	3.83501	0.5856	-0.10289
C	3.73	-0.33769	0.19791	C	4.15203	-0.88915	0.35763
C	4.72247	0.21354	1.20145	C	5.47729	-0.9039	1.09233
C	1.3319	-0.02488	-0.68945	C	2.46499	0.67589	-0.79726
C	0.99475	-1.4977	-0.35709	C	1.33991	0.18193	0.11257
C	2.25767	-2.36084	-0.40102	C	1.61832	-1.25814	0.60508
C	3.33824	-1.79467	0.52406	C	2.9945	-1.34624	1.26913
C	4.41441	-0.26571	-1.18244	C	4.30224	-1.85494	-0.83589
C	2.63755	2.83841	1.33773	C	5.84719	2.17304	-0.15355
C	-0.0823	-1.99198	-1.30533	C	-0.01692	0.2219	-0.5571
C	-1.4608	-1.46924	-1.17667	C	-1.19597	0.12941	0.34169
O	0.17448	-2.77558	-2.22231	O	-0.14126	0.29661	-1.7801
C	-1.91569	-0.76113	-0.13589	C	-2.4435	0.07244	-0.13903
C	-3.29526	-0.17829	-0.06726	C	-3.65402	0.02032	0.74039
O	-3.12685	1.26346	0.08191	O	-4.41995	-1.20279	0.45076
C	-4.12231	-0.65988	1.13741	C	-4.49441	1.33342	0.7218
C	-5.46956	0.06408	1.18577	C	-5.90052	1.08271	1.27617
C	-4.31613	-2.17532	1.04411	C	-3.75836	2.37063	1.57226
O	-3.34323	-0.33076	2.30056	O	-4.56557	1.89594	-0.5922
C	-2.83748	1.96603	-1.0337	C	-5.01265	-1.41265	-0.73152
C	-2.54889	3.40237	-0.71068	C	-5.56943	-2.80191	-0.82115
O	-2.79378	1.47014	-2.14598	O	-5.09066	-0.57992	-1.62643
H	2.03264	0.38651	1.27748	H	3.75495	1.1568	0.83179
H	0.58935	-1.51894	0.66249	H	1.28149	0.82481	1.00279
H	5.89935	0.12808	2.94877	H	6.70163	-1.14015	2.79222
H	4.85198	-1.35788	2.6331	H	4.89475	-1.3698	3.08769
H	2.88702	3.67379	-1.2252	H	5.8966	1.53418	-2.7823
H	2.70643	2.03349	-2.05507	H	4.43598	0.40362	-2.80662
H	5.10771	1.20122	0.94776	H	6.33936	-0.7019	0.45665
H	1.65659	0.03948	-1.73304	H	2.45872	0.09778	-1.72531
H	0.43787	0.59799	-0.6016	H	2.27891	1.71709	-1.08201
H	2.01582	-3.38669	-0.1013	H	0.84749	-1.57095	1.31722
H	2.62085	-2.41739	-1.43183	H	1.55606	-1.94281	-0.24842
H	2.96929	-1.8388	1.5564	H	2.98224	-0.72172	2.17086
H	4.23237	-2.42717	0.48172	H	3.17785	-2.37555	1.5984
H	4.75602	0.74948	-1.39922	H	5.16418	-1.58682	-1.45197
H	5.28952	-0.92432	-1.19459	H	4.45724	-2.87461	-0.46711
H	3.75313	-0.57621	-1.9945	H	3.42153	-1.86481	-1.48214
H	2.85531	3.89262	1.14307	H	6.66476	2.55075	-0.77464
H	1.66328	2.77553	1.84084	H	5.27655	3.03291	0.22251
H	3.37662	2.45466	2.05016	H	6.27557	1.68011	0.72636
H	-2.11381	-1.69811	-2.01631	H	-1.00929	0.11673	1.41217
H	-1.2825	-0.53541	0.71625	H	-2.60215	0.08304	-1.21286
H	-3.85172	-0.37589	-0.98496	H	-3.35587	-0.15877	1.77403
H	-6.04382	-0.28624	2.04976	H	-6.42596	2.03837	1.3622
H	-5.32735	1.14329	1.2783	H	-6.48187	0.43613	0.61357
H	-6.0559	-0.13716	0.28361	H	-5.86242	0.61754	2.26621
H	-4.91417	-2.52001	1.89393	H	-4.29195	3.32347	1.51266
H	-3.35423	-2.69448	1.06579	H	-2.73921	2.52465	1.20697
H	-4.84083	-2.45245	0.12453	H	-3.71387	2.06218	2.62025
H	-3.82924	-0.65614	3.0728	H	-4.86531	1.17541	-1.17929
H	-1.5393	3.46642	-0.28873	H	-6.1938	-3.01103	0.05189
H	-2.59392	4.00161	-1.62026	H	-4.74387	-3.52114	-0.81934
H	-3.24967	3.78282	0.03596	H	-6.1504	-2.90906	-1.73675
atom	Con f. 7- 2a			atom	Con f. 7- 2b		
C	5.5676	-2.07594	0.89088	C	6.73851	-0.33628	0.22212
C	3.65256	1.2056	2.85962	C	3.52498	2.78252	0.18118
C	3.34616	1.46544	1.58285	C	3.67885	1.71715	0.97639
C	2.9241	0.337	0.64903	C	3.20007	0.32257	0.59441
C	4.09095	-0.29573	-0.20553	C	4.23426	-0.48763	-0.27457
C	5.15906	-0.81564	0.73518	C	5.49273	-0.68967	0.54173
C	1.73278	0.76168	-0.22996	C	1.80304	0.34666	-0.05201
C	1.19189	-0.41192	-1.07516	C	1.26619	-1.08232	-0.29968

C	2.3151	-1.00621	-1.92833	C	2.2447	-1.88554	-1.1551
C	3.48506	-1.44053	-1.0452	C	3.6355	-1.90129	-0.51892
C	4.77699	0.73367	-1.12912	C	4.53219	0.19773	-1.61292
C	3.36309	2.8822	1.06237	C	4.32012	1.84768	2.33385
C	0.02562	0.07507	-1.9165	C	-0.11615	-0.98895	-0.91081
C	-1.32396	0.18993	-1.32859	C	-1.20515	-0.54395	-0.00747
O	0.188	0.4107	-3.09341	O	-0.32232	-1.24173	-2.09905
C	-1.64375	-0.16698	-0.07685	C	-2.44829	-0.33172	-0.45948
C	-3.0083	-0.08022	0.52981	C	-3.60216	0.04406	0.4154
O	-3.81648	0.71998	-0.37624	O	-4.58134	0.78727	-0.35371
C	-3.64774	-1.48313	0.80472	C	-4.35028	-1.19067	1.00522
C	-4.26386	-2.05368	-0.47523	C	-5.4817	-0.71416	1.91122
C	-2.60433	-2.44194	1.38115	C	-4.86209	-2.12495	-0.09095
O	-4.64533	-1.3609	1.82674	O	-3.4269	-1.87522	1.86075
C	-4.88267	1.39061	0.07969	C	-4.26565	2.05804	-0.68035
C	-5.43814	2.31012	-0.96385	C	-5.3704	2.70892	-1.45828
O	-5.33793	1.25329	1.20779	O	-3.21031	2.58074	-0.36793
H	2.57387	-0.48415	1.28689	H	3.09998	-0.24705	1.52863
H	0.83003	-1.18463	-0.38676	H	1.1537	-1.56438	0.68231
H	6.35971	-2.31909	1.59486	H	7.56838	-0.55263	0.89008
H	5.146	-2.90973	0.33749	H	6.98493	0.1774	-0.70231
H	3.95577	1.99374	3.54489	H	3.87489	3.76557	0.48688
H	3.61373	0.19618	3.26117	H	3.04863	2.72113	-0.79222
H	5.64073	-0.0422	1.33098	H	5.32697	-1.20321	1.49038
H	2.01922	1.57407	-0.90611	H	1.82505	0.88138	-1.00692
H	0.94012	1.15443	0.41478	H	1.12253	0.90005	0.60266
H	1.93851	-1.86668	-2.49284	H	1.88096	-2.91261	-1.27283
H	2.64075	-0.2665	-2.66603	H	2.28393	-1.45356	-2.16002
H	3.1361	-2.22951	-0.36718	H	3.57748	-2.42484	0.44452
H	4.27256	-1.88456	-1.6649	H	4.3303	-2.47304	-1.14477
H	5.35575	1.46053	-0.55257	H	5.0172	1.16512	-1.46619
H	5.47262	0.21972	-1.80127	H	5.19563	-0.42792	-2.21866
H	4.0627	1.28523	-1.74509	H	3.62244	0.36906	-2.19216
H	2.34859	3.22705	0.82864	H	3.62177	1.53561	3.12151
H	3.78304	3.56468	1.80656	H	5.1994	1.20024	2.42102
H	3.94318	2.981	0.1406	H	4.63002	2.87688	2.53782
H	-2.07434	0.59659	-1.99948	H	-0.95785	-0.38654	1.03861
H	-0.88793	-0.56486	0.59136	H	-2.66571	-0.48429	-1.51482
H	-2.94578	0.44131	1.49137	H	-3.26563	0.66017	1.2527
H	-4.63748	-3.06236	-0.27672	H	-5.9663	-1.57776	2.37602
H	-5.10083	-1.4387	-0.81609	H	-5.09098	-0.06673	2.7027
H	-3.52475	-2.10881	-1.28052	H	-6.22969	-0.15853	1.34214
H	-3.11987	-3.34137	1.7298	H	-5.39382	-2.96651	0.36239
H	-2.08523	-1.99375	2.2343	H	-4.03267	-2.52278	-0.68502
H	-1.86693	-2.73722	0.631	H	-5.54624	-1.60265	-0.76514
H	-5.1086	-0.51323	1.69357	H	-2.75832	-2.30095	1.30211
H	-6.31751	2.82321	-0.57598	H	-5.56223	2.13845	-2.37226
H	-4.67504	3.03701	-1.2576	H	-6.29075	2.7048	-0.86632
H	-5.7041	1.73167	-1.85407	H	-5.09267	3.73233	-1.70927

atom	Con f. 7- 2c			atom	Con f. 7- 2d		
C	-5.20124	2.58467	0.62487	C	5.31487	2.00704	-1.71087
C	-5.18609	-1.87941	-0.00422	C	5.20236	-1.53227	1.07324
C	-4.51117	-1.11767	0.86489	C	4.60371	-1.33192	-0.10692
C	-3.19337	-0.43583	0.52034	C	3.27072	-0.60773	-0.24706
C	-3.37125	0.97725	-0.15479	C	3.41203	0.95811	-0.36229
C	-4.07461	1.8975	0.81927	C	4.19745	1.2852	-1.61369
C	-2.26135	-1.34489	-0.29941	C	2.27082	-1.00776	0.85177
C	-0.88102	-0.71385	-0.50314	C	0.88621	-0.39951	0.61419
C	-1.01436	0.67086	-1.17787	C	0.98225	1.13986	0.5072
C	-1.95215	1.57127	-0.3726	C	1.9875	1.54108	-0.57295
C	-4.11733	0.89437	-1.4913	C	4.05447	1.57552	0.8851
C	-5.01482	-0.91448	2.27061	C	5.21466	-1.85081	-1.38291
C	0.02544	-1.59769	-1.33922	C	-0.08882	-0.7611	1.71873
C	1.49313	-1.41314	-1.28751	C	-1.54525	-0.67621	1.4772
O	-0.4347	-2.44913	-2.10367	O	0.30574	-1.08104	2.843
C	2.13564	-0.57536	-0.46367	C	-2.121	-0.45401	0.289
C	3.62829	-0.44193	-0.45196	C	-3.60435	-0.37997	0.09684
O	3.99587	0.93002	-0.84166	O	-3.91444	0.94387	-0.43401
C	4.29024	-0.93853	0.86713	C	-4.14343	-1.3819	-0.95005
C	4.29766	-2.46822	0.83607	C	-3.75482	-2.80288	-0.55326
C	5.71537	-0.39229	0.99464	C	-5.66181	-1.24127	-1.07917
O	3.5116	-0.58271	2.01463	O	-3.51364	-1.13056	-2.21659
C	3.66693	1.99579	-0.10113	C	-3.98981	1.96587	0.44533

C	4.00644	3.27392	-0.80759	C	-4.27631	3.26014	-0.25672
O	3.15426	1.93792	1.01013	O	-3.83674	1.82658	1.64553
H	-2.68296	-0.23621	1.47297	H	2.83497	-0.9286	-1.20365
H	-0.4131	-0.56385	0.47718	H	0.49323	-0.77583	-0.3378
H	-5.6006	3.23181	1.40163	H	5.77993	2.1878	-2.67663
H	-5.76908	2.53397	-0.29927	H	5.81033	2.4423	-0.84815
H	-6.12273	-2.35517	0.27647	H	6.15273	-2.0566	1.13849
H	-4.83743	-2.06006	-1.01606	H	4.77587	-1.1886	2.01032
H	-3.56216	2.00319	1.77698	H	3.76023	0.88193	-2.52866
H	-2.69658	-1.57046	-1.27618	H	2.63122	-0.69943	1.83618
H	-2.15553	-2.30326	0.22011	H	2.19357	-2.09997	0.87962
H	-0.03292	1.14742	-1.2626	H	0.0013	1.56444	0.27242
H	-1.3897	0.53429	-2.19863	H	1.27867	1.54558	1.48138
H	-1.49704	1.76002	0.60848	H	1.60962	1.20017	-1.54585
H	-2.04915	2.54455	-0.8675	H	2.05824	2.63367	-0.6278
H	-5.14029	0.53634	-1.3563	H	5.08066	1.22654	1.01921
H	-4.16175	1.88206	-1.96132	H	4.07319	2.66683	0.80061
H	-3.62109	0.21971	-2.19181	H	3.50027	1.32605	1.79224
H	-4.28097	-1.28246	2.9997	H	6.16771	-2.35581	-1.19937
H	-5.16561	0.14839	2.48761	H	4.53678	-2.56174	-1.87343
H	-5.96076	-1.43699	2.44129	H	5.38805	-1.0395	-2.0978
H	2.04771	-2.03304	-1.98936	H	-2.15686	-0.82189	2.36516
H	1.59295	0.04124	0.24357	H	-1.53187	-0.31108	-0.6119
H	4.06045	-1.01629	-1.27195	H	-4.13095	-0.51311	1.04346
H	4.68599	-2.8427	1.78744	H	-4.17428	-3.51191	-1.2727
H	3.28623	-2.86127	0.69934	H	-2.66836	-2.92453	-0.54062
H	4.93266	-2.84357	0.02907	H	-4.14249	-3.04524	0.44015
H	6.19664	-0.84707	1.86549	H	-6.02828	-1.93308	-1.84298
H	5.71519	0.69176	1.13637	H	-5.93824	-0.22383	-1.37032
H	6.31239	-0.62424	0.10714	H	-6.15796	-1.47398	-0.13185
H	3.3442	0.37645	1.94235	H	-3.76595	-0.23469	-2.48886
H	5.05202	3.25572	-1.12756	H	-5.18446	3.16554	-0.85916
H	3.38621	3.36606	-1.70499	H	-4.39224	4.05952	0.47481
H	3.82833	4.12086	-0.14564	H	-3.45073	3.49524	-0.93609

atom	Con f. 7- 2e			atom	Con f. 7- 2f		
C	6.59193	-0.07729	0.25159	C	-5.64524	-1.35107	1.06757
C	3.07742	2.53226	-0.80775	C	-2.59806	-1.75747	-2.19996
C	3.34649	1.84746	0.3102	C	-2.45341	-1.81937	-0.87097
C	3.00275	0.3739	0.4824	C	-2.27308	-0.58378	0.00078
C	4.10946	-0.60415	-0.06705	C	-3.62666	0.08961	0.44117
C	5.38201	-0.38367	0.72187	C	-4.40709	-0.90171	1.27695
C	1.60932	0.0344	-0.07668	C	-1.30345	0.43104	-0.62955
C	1.21435	-1.4283	0.23341	C	-1.00204	1.60015	0.33821
C	2.26199	-2.39899	-0.30742	C	-2.3006	2.28753	0.76371
C	3.64936	-2.05738	0.23926	C	-3.27277	1.27855	1.37788
C	4.34093	-0.43226	-1.57253	C	-4.44534	0.58824	-0.75542
C	3.98988	2.51945	1.49557	C	-2.42317	-3.14369	-0.15312
C	-0.17466	-1.68449	-0.31172	C	-0.02747	2.56318	-0.31502
C	-1.27647	-0.94185	0.34894	C	1.39076	2.17249	-0.46891
O	-0.37728	-2.42527	-1.27443	O	-0.40002	3.64719	-0.77037
C	-2.48119	-0.8119	-0.21667	C	1.93277	1.06879	0.06166
C	-3.58822	-0.0012	0.38183	C	3.33118	0.62361	-0.22813
O	-3.7822	1.16956	-0.47269	O	4.03947	0.33352	1.02535
C	-4.94985	-0.72814	0.4063	C	3.37267	-0.53134	-1.28085
C	-4.82078	-2.03092	1.18992	C	3.1781	0.08204	-2.668
C	-6.02343	0.17767	1.01412	C	4.7046	-1.28405	-1.20851
O	-5.31619	-1.09971	-0.93224	O	2.27516	-1.4365	-1.1051
C	-2.95619	2.22124	-0.28778	C	3.66086	-0.65705	1.84334
C	-3.2591	3.32234	-1.2605	C	4.45086	-0.64217	3.11724
O	-2.08544	2.24756	0.5643	O	2.78304	-1.47198	1.58549
H	2.95914	0.18204	1.56343	H	-1.80161	-0.92169	0.93436
H	1.15271	-1.52453	1.32765	H	-0.52002	1.1773	1.22874
H	7.43866	0.03731	0.92356	H	-6.10591	-2.05665	1.75429
H	6.79161	0.07218	-0.80532	H	-6.2459	-1.04442	0.21657
H	3.33217	3.58568	-0.89519	H	-2.71738	-2.6602	-2.79415
H	2.59818	2.07918	-1.66983	H	-2.59978	-0.81801	-2.74373
H	5.26357	-0.51811	1.79834	H	-3.86821	-1.25889	2.15613
H	1.57871	0.18235	-1.16095	H	-1.71547	0.84169	-1.55712
H	0.87838	0.72716	0.35256	H	-0.38079	-0.0898	-0.89822
H	1.99798	-3.42593	-0.03083	H	-2.08336	3.07847	1.49042
H	2.25665	-2.3616	-1.40176	H	-2.75046	2.77918	-0.10462
H	3.64399	-2.19958	1.32813	H	-2.82977	0.87593	2.29837
H	4.39534	-2.75222	-0.16328	H	-4.20123	1.78174	1.67137

H	4.72863	0.56167	-1.80629	H	-4.75716	-0.23718	-1.39923
H	5.06276	-1.17287	-1.93157	H	-5.34461	1.10689	-0.40778
H	3.4194	-0.56955	-2.14197	H	-3.87835	1.29064	-1.36986
H	3.33541	2.45932	2.37503	H	-1.46394	-3.27843	0.36395
H	4.9281	2.02493	1.76885	H	-3.20288	-3.19587	0.61435
H	4.2027	3.57396	1.29674	H	-2.56238	-3.98165	-0.84251
H	-1.05396	-0.45296	1.29318	H	1.98442	2.84478	-1.08481
H	-2.68563	-1.27524	-1.17872	H	1.34679	0.40995	0.69156
H	-3.32817	0.33923	1.3855	H	3.92124	1.45093	-0.62382
H	-5.79047	-2.5358	1.22401	H	3.13108	-0.72218	-3.40763
H	-4.09811	-2.70347	0.71951	H	2.24544	0.65001	-2.72073
H	-4.49581	-1.83378	2.21536	H	4.00938	0.74368	-2.92579
H	-6.98533	-0.34289	1.00929	H	4.7537	-2.00125	-2.033
H	-6.12655	1.10338	0.44076	H	4.80024	-1.8396	-0.2722
H	-5.77476	0.4401	2.04721	H	5.55377	-0.59887	-1.29324
H	-5.3922	-0.28181	-1.44747	H	2.29788	-1.70778	-0.16734
H	-3.09968	2.96114	-2.28142	H	5.52064	-0.64772	2.88979
H	-4.30889	3.61787	-1.17375	H	4.23329	0.27848	3.66815
H	-2.61162	4.17664	-1.06422	H	4.18968	-1.50843	3.72438
atom	Con f. 7- 2g			atom	Con f. 7- 2h		
C	5.96244	0.1362	1.49602	C	-6.34843	-1.46111	-1.29077
C	3.22011	2.36613	-1.25529	C	-5.02016	1.38697	1.93735
C	3.00977	1.90915	-0.01506	C	-4.83326	1.41881	0.61257
C	2.57183	0.47998	0.27724	C	-3.69001	0.68759	-0.07903
C	3.76418	-0.5437	0.38069	C	-4.02638	-0.8093	-0.44108
C	4.65617	-0.13119	1.53147	C	-5.18299	-0.82499	-1.41747
C	1.48716	-0.00236	-0.70203	C	-2.36678	0.80139	0.69783
C	0.93419	-1.39047	-0.30293	C	-1.19471	0.19741	-0.0753
C	2.07116	-2.41003	-0.20022	C	-1.48094	-1.27694	-0.44281
C	3.15712	-1.92368	0.76073	C	-2.80342	-1.39229	-1.20277
C	4.54935	-0.65509	-0.93052	C	-4.32528	-1.6548	0.80187
C	3.16445	2.81184	1.18129	C	-5.74485	2.22055	-0.28024
C	-0.12399	-1.81649	-1.30506	C	0.11035	0.27165	0.6886
C	-1.47939	-1.22893	-1.24744	C	1.34866	0.04557	-0.09892
O	0.13182	-2.62421	-2.20185	O	0.14654	0.47885	1.90248
C	-1.90539	-0.35869	-0.3234	C	2.54891	-0.0336	0.4864
C	-3.27181	0.25107	-0.33821	C	3.824	-0.26911	-0.25919
O	-3.91489	-0.04758	0.93666	O	4.69077	0.88404	-0.03284
C	-3.24694	1.79553	-0.43661	C	4.62066	-1.49236	0.25027
C	-2.49562	2.2251	-1.69305	C	3.75474	-2.74554	0.16419
C	-4.67592	2.34383	-0.43664	C	5.90935	-1.65538	-0.55937
O	-2.50999	2.33032	0.67432	O	4.92969	-1.31278	1.64151
C	-4.4856	-1.26321	1.07601	C	4.50066	1.96683	-0.81639
C	-5.09126	-1.42064	2.43936	C	5.4528	3.06832	-0.45531
O	-4.49319	-2.10227	0.19315	O	3.65941	2.01464	-1.69605
H	2.11028	0.48689	1.27451	H	-3.53246	1.18731	-1.04522
H	0.45883	-1.28872	0.68055	H	-1.05202	0.74694	-1.01694
H	6.50069	0.4096	2.40002	H	-7.09994	-1.4101	-2.07453
H	6.54556	0.09737	0.58074	H	-6.60442	-2.04928	-0.4146
H	3.52614	3.39478	-1.42999	H	-5.84483	1.92258	2.40139
H	3.0953	1.74397	-2.13588	H	-4.36646	0.83523	2.60535
H	4.14066	-0.06998	2.49146	H	-5.00056	-0.25306	-2.32887
H	1.87993	-0.06037	-1.7221	H	-2.44216	0.30904	1.67118
H	0.6791	0.73392	-0.71838	H	-2.16706	1.85843	0.90377
H	1.67678	-3.37199	0.14647	H	-0.66959	-1.68188	-1.05688
H	2.4881	-2.58663	-1.19621	H	-1.50968	-1.87294	0.47656
H	2.73128	-1.84949	1.77019	H	-2.70352	-0.86461	-2.16029
H	3.96707	-2.66029	0.81561	H	-3.00468	-2.44322	-1.44084
H	5.03158	0.29036	-1.18827	H	-5.22109	-1.30163	1.3172
H	5.32656	-1.42099	-0.84203	H	-4.48441	-2.70033	0.51913
H	3.90509	-0.93625	-1.76596	H	-3.50135	-1.62957	1.51802
H	3.47799	3.81886	0.89068	H	-6.53921	2.71249	0.28881
H	2.21635	2.89075	1.72944	H	-5.17703	2.9917	-0.8174
H	3.90153	2.41241	1.88629	H	-6.20986	1.58659	-1.04266
H	-2.1431	-1.55004	-2.04756	H	1.24743	-0.0594	-1.17573
H	-1.2636	-0.03132	0.48917	H	2.63377	0.07436	1.56501
H	-3.8789	-0.16188	-1.14574	H	3.64101	-0.36515	-1.33066
H	-2.53345	3.31403	-1.78801	H	4.33249	-3.61357	0.49437
H	-1.44709	1.91917	-1.64951	H	2.87027	-2.65636	0.80064
H	-2.94922	1.78279	-2.58453	H	3.42614	-2.91816	-0.86455
H	-4.64588	3.43644	-0.47576	H	6.47561	-2.50872	-0.17539
H	-5.21282	2.04259	0.4673	H	6.53533	-0.76153	-0.48491
H	-5.23401	1.97943	-1.30474	H	5.68734	-1.83195	-1.61646
H	-2.98712	2.08465	1.48195	H	5.4792	-0.51707	1.7129
H	-5.8315	-0.63294	2.60916	H	5.30316	3.35479	0.59023

H	-5.56161	-2.40001	2.52404	H	6.48347	2.71527	-0.55776
H	-4.31259	-1.31339	3.20093	H	5.28652	3.92768	-1.10446

atom	Con f. 7- 2i			atom	Con f. 7- 2j		
C	5.92914	0.14243	1.536	C	-3.94711	2.30112	2.46937
C	3.13407	2.49777	-1.06976	C	-5.36079	-1.57233	1.33947
C	2.93744	1.94391	0.1326	C	-4.70064	-1.22188	0.22899
C	2.53867	0.48569	0.31761	C	-3.35331	-0.51516	0.32488
C	3.75772	-0.51025	0.35373	C	-3.42702	1.06182	0.29068
C	4.63056	-0.16216	1.54026	C	-4.30053	1.53769	1.43394
C	1.47166	0.04592	-0.70024	C	-2.36153	-1.06459	-0.71711
C	0.95547	-1.38106	-0.40517	C	-0.95789	-0.48255	-0.54443
C	2.11961	-2.37437	-0.37693	C	-1.0129	1.0605	-0.61122
C	3.18889	-1.93169	0.6235	C	-1.98811	1.5999	0.43482
C	4.55469	-0.5013	-0.95563	C	-4.06594	1.59854	-1.00839
C	3.06884	2.75893	1.39301	C	-5.25405	-1.56498	-1.13278
C	-0.09135	-1.75609	-1.43882	C	0.0041	-1.00217	-1.59643
C	-1.46354	-1.21856	-1.33383	C	1.46276	-0.92982	-1.36291
O	0.18884	-2.47658	-2.40053	O	-0.40093	-1.45121	-2.6718
C	-1.92313	-0.45865	-0.33075	C	2.0424	-0.53208	-0.22324
C	-3.30047	0.12254	-0.30854	C	3.52673	-0.47105	-0.03424
O	-3.84707	0.0144	1.03631	O	3.88101	0.92898	0.18424
C	-3.36105	1.63567	-0.65221	C	4.02329	-1.22524	1.21986
C	-2.49167	2.47516	0.28716	C	3.57971	-2.68346	1.15522
C	-2.97219	1.84899	-2.11099	C	5.54562	-1.11536	1.3309
O	-4.73356	2.03937	-0.54578	O	3.40013	-0.66691	2.3882
C	-4.31372	-1.19532	1.41762	C	4.05066	1.70567	-0.90742
C	-4.8722	-1.14524	2.80862	C	4.36437	3.12017	-0.51875
O	-4.27547	-2.17777	0.69968	O	3.94973	1.28758	-2.04677
H	2.07306	0.40668	1.3098	H	-2.94682	-0.74913	1.31697
H	0.48041	-1.36688	0.58353	H	-0.5759	-0.76024	0.44511
H	6.45412	0.36118	2.46239	H	-4.67734	2.58628	3.22274
H	6.51839	0.18856	0.62516	H	-2.93777	2.67405	2.61564
H	3.41226	3.54444	-1.16645	H	-6.32227	-2.07875	1.29631
H	3.02542	1.94019	-1.99456	H	-4.96169	-1.36374	2.32887
H	4.10708	-0.18751	2.49756	H	-5.33694	1.21128	1.36724
H	1.87082	0.06987	-1.71924	H	-2.70991	-0.85112	-1.73115
H	0.64365	0.7595	-0.67093	H	-2.32021	-2.15612	-0.62739
H	1.75169	-3.37034	-0.10545	H	-0.01847	1.48398	-0.44063
H	2.54331	-2.46294	-1.3819	H	-1.3175	1.36071	-1.62063
H	2.75799	-1.94751	1.63338	H	-1.60695	1.33059	1.42763
H	4.01959	-2.64687	0.6264	H	-2.00736	2.69489	0.38914
H	5.0175	0.47162	-1.13529	H	-5.13232	1.36114	-1.05337
H	5.34789	-1.2549	-0.91994	H	-3.97106	2.68931	-1.04281
H	3.92231	-0.73086	-1.81562	H	-3.59226	1.19295	-1.9059
H	3.35162	3.79365	1.17813	H	-5.29984	-0.69628	-1.79588
H	2.1206	2.76838	1.94647	H	-4.6235	-2.30665	-1.63744
H	3.8195	2.32957	2.06514	H	-6.2605	-1.98456	-1.04881
H	-2.1142	-1.48149	-2.16527	H	2.07041	-1.2372	-2.21137
H	-1.29389	-0.20094	0.51646	H	1.45476	-0.21992	0.63486
H	-3.96207	-0.40932	-0.99526	H	4.05179	-0.83282	-0.91962
H	-2.6328	3.53491	0.05677	H	2.48923	-2.7635	1.1573
H	-2.76694	2.30788	1.33271	H	3.96087	-3.16279	0.24915
H	-1.43035	2.23652	0.16796	H	3.96743	-3.22342	2.02377
H	-3.11453	2.9016	-2.37229	H	5.88339	-1.63039	2.23472
H	-3.5969	1.24011	-2.77183	H	5.8598	-0.0692	1.38886
H	-1.92548	1.58869	-2.28102	H	6.03505	-1.57296	0.46548
H	-5.01531	1.84878	0.36243	H	3.68375	0.25845	2.44983
H	-5.72989	-0.46534	2.83354	H	5.21343	3.14112	0.1704
H	-5.18246	-2.14299	3.11808	H	4.58717	3.70725	-1.40947
H	-4.11881	-0.7534	3.49815	H	3.50301	3.55085	0.00224
atom	Con f. 7- 2k			atom	Con f. 7- 2l		
C	-6.23539	-0.73427	-0.95792	C	5.10545	-0.76774	2.51463
C	-3.63516	2.58364	-1.44134	C	3.25436	2.80471	1.10798
C	-2.96916	1.92524	-0.48504	C	3.0625	1.99505	0.05963
C	-2.86245	0.40427	-0.52855	C	2.64392	0.54558	0.27593
C	-4.00079	-0.37257	0.2377	C	3.82711	-0.49732	0.31299
C	-5.34563	0.03543	-0.329	C	4.79902	-0.09487	1.40424
C	-1.46406	-0.0574	-0.08494	C	1.5469	0.13268	-0.72313
C	-1.2642	-1.57953	-0.25146	C	0.99355	-1.27783	-0.42268
C	-2.36047	-2.34981	0.48093	C	2.13275	-2.29927	-0.40132
C	-3.74731	-1.88258	0.03413	C	3.21187	-1.88347	0.59944
C	-4.0332	-0.03235	1.74264	C	4.62876	-0.53473	-1.00513
C	-2.26222	2.6788	0.61614	C	3.20351	2.51762	-1.3495

C	0.13315	-1.94158	0.21293	C	-0.06802	-1.62493	-1.45101
C	1.23495	-1.21809	-0.46797	C	-1.42657	-1.05212	-1.33972
O	0.34172	-2.71972	1.14318	O	0.18833	-2.35295	-2.4134
C	2.38509	-0.94549	0.15624	C	-1.86185	-0.2805	-0.33564
C	3.42675	-0.03298	-0.41334	C	-3.24039	0.30118	-0.2878
O	3.45421	1.16048	0.43472	O	-3.90583	-0.22173	0.90142
C	4.86031	-0.60211	-0.37063	C	-3.24957	1.83886	-0.12362
C	4.90635	-1.92732	-1.12519	C	-2.45208	2.48722	-1.25121
C	5.84992	0.40551	-0.96031	C	-4.69006	2.35513	-0.09898
O	5.21183	-0.90201	0.98985	O	-2.57653	2.18858	1.09625
C	2.48848	2.08541	0.26048	C	-4.48705	-1.43686	0.80503
C	2.59458	3.1779	1.28274	C	-5.1041	-1.84447	2.11015
O	1.63669	2.01464	-0.60942	O	-4.49151	-2.095	-0.21962
H	-2.98328	0.1092	-1.57834	H	2.20027	0.49358	1.27803
H	-1.30857	-1.80314	-1.32873	H	0.52425	-1.25084	0.56834
H	-7.17838	-0.3202	-1.3065	H	5.83403	-0.37305	3.21864
H	-6.07564	-1.79062	-1.15204	H	4.65863	-1.72313	2.7737
H	-3.72347	3.66755	-1.43236	H	3.55124	3.84308	0.98005
H	-4.11591	2.0605	-2.26397	H	3.12449	2.45546	2.12915
H	-5.59243	1.0847	-0.1758	H	5.29889	0.85603	1.22677
H	-1.29076	0.20325	0.96449	H	1.93244	0.14494	-1.74797
H	-0.71695	0.48943	-0.66597	H	0.73826	0.86869	-0.6821
H	-2.25373	-3.42388	0.29395	H	1.74107	-3.28695	-0.13309
H	-2.23625	-2.21017	1.56036	H	2.55228	-2.39383	-1.40771
H	-3.86627	-2.11805	-1.03097	H	2.76952	-1.87159	1.60337
H	-4.51779	-2.4496	0.56896	H	4.01103	-2.63331	0.6163
H	-4.3299	1.00751	1.90623	H	5.19481	0.38912	-1.15106
H	-4.76683	-0.66783	2.25045	H	5.34863	-1.35989	-0.97611
H	-3.06607	-0.18678	2.22749	H	3.98912	-0.68007	-1.87911
H	-2.53455	3.73786	0.59844	H	3.59586	3.53837	-1.34758
H	-2.49332	2.28146	1.60894	H	3.86555	1.89738	-1.96055
H	-1.17316	2.61322	0.49758	H	2.23359	2.5335	-1.86088
H	1.02915	-0.79864	-1.44856	H	-2.08774	-1.30118	-2.16704
H	2.56877	-1.32378	1.15887	H	-1.22439	-0.02766	0.50629
H	3.17261	0.26842	-1.43115	H	-3.81834	0.01666	-1.16855
H	5.92754	-2.31905	-1.11704	H	-2.50343	3.57571	-1.15696
H	4.24852	-2.66673	-0.65981	H	-1.40129	2.18713	-1.21339
H	4.59515	-1.79096	-2.16479	H	-2.85929	2.20317	-2.22566
H	6.86344	-0.00148	-0.90142	H	-4.68668	3.43886	0.04861
H	5.82283	1.35	-0.40932	H	-5.25771	1.89567	0.71534
H	5.61866	0.61362	-2.00965	H	-5.20118	2.13368	-1.0411
H	5.16972	-0.07027	1.48663	H	-3.07433	1.78524	1.82423
H	2.265	2.78645	2.25152	H	-5.80719	-1.07711	2.44693
H	3.63152	3.50566	1.39045	H	-5.61686	-2.799	1.99389
H	1.95751	4.01458	0.99593	H	-4.32123	-1.93339	2.87019

atom	Con f. 7- 2m			atom	Con f. 7- 2n		
C	5.31019	2.40304	-0.83784	C	6.69345	-0.17651	0.264
C	5.13441	-1.94094	0.28443	C	3.31513	2.71187	-0.30049
C	4.51206	-1.2767	-0.69672	C	3.53313	1.81568	0.66933
C	3.20301	-0.52655	-0.48748	C	3.12477	0.35311	0.55334
C	3.3977	0.9474	0.0355	C	4.19982	-0.5536	-0.15679
C	4.16851	1.73341	-1.00301	C	5.47063	-0.52797	0.66426
C	2.21104	-1.31366	0.3865	C	1.73173	0.18697	-0.08017
C	0.84473	-0.62721	0.45662	C	1.26521	-1.28724	-0.04625
C	0.99217	0.81974	0.9817	C	2.28453	-2.19536	-0.73199
C	1.99057	1.601	0.12665	C	3.67459	-2.01654	-0.11768
C	4.0873	0.99081	1.40386	C	4.45443	-0.1248	-1.60613
C	5.06915	-1.26036	-2.09673	C	4.1784	2.22133	1.96929
C	-0.12622	-1.38352	1.34472	C	-0.11892	-1.38023	-0.65336
C	-1.57606	-1.12837	1.23111	C	-1.2167	-0.75234	0.12285
O	0.27407	-2.16724	2.21002	O	-0.3216	-1.91585	-1.74404
C	-2.15588	-0.41412	0.25761	C	-2.44892	-0.61484	-0.38047
C	-3.63011	-0.17545	0.16655	C	-3.58115	0.00489	0.37237
O	-3.87158	1.14618	-0.39318	O	-4.22752	0.99814	-0.47715
C	-4.38876	-1.1512	-0.77481	C	-4.70618	-0.97769	0.78574
C	-3.81518	-1.13227	-2.19359	C	-5.33182	-1.6828	-0.41987
C	-4.37642	-2.55721	-0.18598	C	-4.16222	-1.97949	1.79838
O	-5.76447	-0.74531	-0.79186	O	-5.70256	-0.21317	1.48025
C	-3.51378	2.21325	0.35158	C	-3.62899	2.20559	-0.57923
C	-3.84263	3.49804	-0.3487	C	-4.4116	3.12126	-1.47302
O	-2.98714	2.11305	1.44584	O	-2.59062	2.4847	-0.00774
H	2.73736	-0.42034	-1.47739	H	3.04883	-0.03747	1.57744
H	0.42674	-0.57298	-0.55584	H	1.17068	-1.57351	1.01178

H	5.76218	2.94728	-1.66316	H	7.53692	-0.21474	0.94866
H	5.83901	2.43811	0.10983	H	6.90712	0.16101	-0.74572
H	6.06647	-2.4701	0.10118	H	3.61509	3.7503	-0.18222
H	4.7448	-1.98614	1.29644	H	2.83303	2.45324	-1.23791
H	3.69773	1.74689	-1.9875	H	5.33762	-0.85615	1.69658
H	2.59924	-1.43909	1.40027	H	1.73271	0.53192	-1.11905
H	2.09712	-2.32153	-0.02694	H	1.02289	0.82231	0.45956
H	0.02296	1.32822	0.97441	H	1.97276	-3.24186	-0.64005
H	1.32257	0.7845	2.02619	H	2.30384	-1.97149	-1.80355
H	1.58226	1.69618	-0.88812	H	3.64389	-2.34666	0.92907
H	2.09998	2.61889	0.51878	H	4.39757	-2.66258	-0.629
H	5.10461	0.59705	1.35041	H	4.87499	0.88186	-1.65739
H	4.13894	2.02134	1.76988	H	5.15785	-0.81307	-2.0856
H	3.54541	0.40424	2.14862	H	3.5359	-0.13079	-2.19621
H	4.35187	-1.70026	-2.80212	H	4.43687	3.28433	1.97825
H	5.25454	-0.23603	-2.43733	H	3.50375	2.02368	2.81266
H	6.00648	-1.82023	-2.16639	H	5.09044	1.64434	2.15611
H	-2.17666	-1.57062	2.02322	H	-0.98135	-0.38291	1.1173
H	-1.56753	0.02639	-0.54277	H	-2.65977	-0.96521	-1.38862
H	-4.09583	-0.22077	1.15412	H	-3.21997	0.50698	1.27202
H	-4.4198	-1.77778	-2.8368	H	-6.1652	-2.30459	-0.08065
H	-3.82718	-0.11998	-2.6082	H	-5.71249	-0.95766	-1.14502
H	-2.78404	-1.498	-2.20945	H	-4.60626	-2.32664	-0.92553
H	-4.99287	-3.21223	-0.80841	H	-4.98033	-2.60734	2.16297
H	-4.78709	-2.55683	0.82834	H	-3.71445	-1.46222	2.65261
H	-3.36334	-2.96317	-0.15458	H	-3.40634	-2.62423	1.34446
H	-5.78932	0.16615	-1.12201	H	-6.0172	0.46799	0.86563
H	-3.56031	4.34314	0.2786	H	-5.41972	3.25777	-1.06967
H	-3.3049	3.54366	-1.30084	H	-3.90686	4.08409	-1.54854
H	-4.91329	3.53599	-0.57087	H	-4.51027	2.67098	-2.46543

atom	Con f. 7- 2o			atom	Con f. 7- 2p		
C	6.86452	-0.40761	0.09605	C	-6.43639	-1.85686	-0.28411
C	3.73582	2.80119	0.11068	C	-5.01104	2.16134	1.21338
C	3.88932	1.74542	0.91875	C	-4.92334	1.59567	0.00361
C	3.35988	0.3584	0.57852	C	-3.80176	0.64	-0.38315
C	4.34119	-0.4937	-0.31196	C	-4.09676	-0.8623	-0.00845
C	5.62089	-0.71873	0.46433	C	-5.31628	-1.32508	-0.77565
C	1.94153	0.40968	-0.01828	C	-2.43248	1.10047	0.14724
C	1.36054	-1.00869	-0.22077	C	-1.29318	0.22306	-0.37
C	2.28467	-1.84853	-1.10103	C	-1.53919	-1.26041	-0.01011
C	3.69633	-1.89376	-0.51408	C	-2.90564	-1.71652	-0.52497
C	4.61206	0.16309	-1.67025	C	-4.2702	-1.05966	1.50171
C	4.58254	1.88067	2.24988	C	-5.93272	1.90647	-1.07156
C	-0.04397	-0.89725	-0.77457	C	0.0587	0.6496	0.16084
C	-1.08425	-0.39892	0.16106	C	1.2456	0.06956	-0.5177
O	-0.31009	-1.18271	-1.94264	O	0.17425	1.41615	1.11771
C	-2.35505	-0.23736	-0.22562	C	2.48544	0.28286	-0.06196
C	-3.43872	0.21897	0.70098	C	3.70231	-0.31912	-0.69209
O	-4.07873	1.42528	0.15252	O	4.65438	0.74648	-1.04265
C	-4.42964	-0.91507	1.10436	C	4.31367	-1.49044	0.13643
C	-3.73622	-1.795	2.14632	C	3.43458	-2.72406	-0.07752
C	-5.72458	-0.32672	1.67364	C	5.75322	-1.77188	-0.30572
O	-4.71393	-1.77946	-0.00025	O	4.26216	-1.22375	1.54163
C	-4.76989	1.41001	-0.99458	C	5.25338	1.50389	-0.11483
C	-5.16246	2.79598	-1.41029	C	6.03762	2.61554	-0.74524
O	-5.04728	0.39674	-1.62453	O	5.17388	1.31495	1.09282
H	3.27753	-0.19257	1.52559	H	-3.73755	0.65485	-1.48018
H	1.27751	-1.47256	0.77312	H	-1.24513	0.28851	-1.46702
H	7.71052	-0.63763	0.73872	H	-7.24157	-2.16969	-0.94399
H	7.09335	0.08341	-0.84505	H	-6.60127	-2.0011	0.7795
H	4.12345	3.77905	0.38597	H	-5.82233	2.843	1.45704
H	3.22323	2.7366	-0.84391	H	-4.28747	1.9722	1.99994
H	5.47333	-1.21165	1.42682	H	-5.22675	-1.21393	-1.85765
H	1.94285	0.92761	-0.98261	H	-2.41639	1.09401	1.24052
H	1.30017	0.99204	0.65057	H	-2.26598	2.13946	-0.15632
H	1.88978	-2.86659	-1.19251	H	-0.75667	-1.89368	-0.44114
H	2.29984	-1.42914	-2.11209	H	-1.47739	-1.37758	1.0778
H	3.65843	-2.4021	0.45842	H	-2.89648	-1.67799	-1.62203
H	4.35296	-2.49286	-1.15529	H	-3.07667	-2.76304	-0.24725
H	5.13265	1.11623	-1.55583	H	-3.40063	-0.70411	2.05824
H	5.23278	-0.49201	-2.28996	H	-5.14387	-0.52153	1.87573
H	3.68791	0.35612	-2.21898	H	-4.39852	-2.12192	1.73319
H	3.9037	1.60475	3.06754	H	-6.69722	2.60504	-0.71878

H	5.44427	1.20777	2.3185	H	-5.43944	2.34879	-1.94711
H	4.93091	2.90322	2.42284	H	-6.43385	0.99747	-1.42112
H	-0.77983	-0.18126	1.18136	H	1.07203	-0.5462	-1.39613
H	-2.6324	-0.46209	-1.25086	H	2.62921	0.89994	0.81967
H	-3.00194	0.61167	1.61987	H	3.45619	-0.71449	-1.67838
H	-4.38735	-2.63943	2.38962	H	3.8046	-3.53774	0.55275
H	-2.79251	-2.19042	1.76051	H	2.3974	-2.51921	0.20182
H	-3.53491	-1.23573	3.06396	H	3.46107	-3.05254	-1.12004
H	-6.34583	-1.1399	2.06037	H	6.11432	-2.66914	0.2054
H	-6.29741	0.19805	0.90476	H	6.41987	-0.94508	-0.04691
H	-5.52043	0.37221	2.4908	H	5.81346	-1.9392	-1.38567
H	-4.98487	-1.1964	-0.73531	H	4.6586	-0.33974	1.66376
H	-5.81783	2.74985	-2.27959	H	6.59067	3.15858	0.02061
H	-5.66495	3.30449	-0.58277	H	6.72537	2.20782	-1.49156
H	-4.26152	3.36825	-1.65413	H	5.35188	3.2949	-1.2616

atom	Con f. 7- 2q			atom	Con f. 7- 2r		
C	5.1307	-0.80345	2.41671	C	5.43188	-1.52914	2.39183
C	2.90308	2.71587	1.46448	C	5.72318	2.15435	0.24223
C	2.77235	2.02424	0.32592	C	4.85494	1.48607	-0.52667
C	2.50786	0.52358	0.36636	C	3.73888	0.66555	0.1092
C	3.78802	-0.39307	0.27624	C	4.07553	-0.86137	0.32628
C	4.73001	-0.02975	1.40654	C	5.32017	-0.97485	1.18352
C	1.44212	0.12427	-0.67026	C	2.40785	0.85348	-0.64234
C	1.02952	-1.35706	-0.52918	C	1.24196	0.16869	0.06765
C	2.2617	-2.25599	-0.64115	C	1.54239	-1.33353	0.27489
C	3.31535	-1.85814	0.3942	C	2.86277	-1.5115	1.02552
C	4.57357	-0.19239	-1.03724	C	4.38099	-1.59724	-0.99651
C	2.82416	2.72606	-1.00932	C	4.93474	1.57548	-2.03111
C	-0.02631	-1.6893	-1.56855	C	-0.0653	0.31309	-0.68187
C	-1.39484	-1.14848	-1.42642	C	-1.30059	0.01316	0.08541
O	0.24318	-2.36629	-2.56391	O	-0.10523	0.63338	-1.87079
C	-1.85578	-0.48844	-0.35573	C	-2.50515	0.00847	-0.49626
C	-3.21155	0.13657	-0.29566	C	-3.77931	-0.28594	0.23015
O	-3.80311	-0.11754	1.00994	O	-4.62361	0.90265	0.14546
C	-3.19423	1.68296	-0.44321	C	-4.60434	-1.42667	-0.4085
C	-2.30447	2.35059	0.60851	C	-3.76415	-2.69877	-0.46731
C	-2.76457	2.06134	-1.85643	C	-5.89405	-1.65273	0.38471
O	-4.54723	2.13857	-0.30636	O	-4.91336	-1.08677	-1.7694
C	-4.33761	-1.34141	1.21672	C	-4.40113	1.88926	1.03991
C	-4.91955	-1.45064	2.59466	C	-5.32992	3.04462	0.80986
O	-4.32869	-2.22103	0.37477	O	-3.54945	1.82473	1.90853
H	2.08533	0.30811	1.35593	H	3.59916	1.06175	1.12298
H	0.58468	-1.48831	0.46589	H	1.10005	0.61381	1.06279
H	5.82567	-0.42559	3.16266	H	6.39198	-1.56059	2.90106
H	4.79697	-1.82838	2.54895	H	4.59392	-1.97135	2.92231
H	3.08811	3.78752	1.46331	H	6.52682	2.74846	-0.18667
H	2.83325	2.23284	2.4358	H	5.6608	2.12722	1.32721
H	5.11842	0.98597	1.35237	H	6.21531	-0.55782	0.7253
H	1.80952	0.28935	-1.68852	H	2.48105	0.4608	-1.66056
H	0.5702	0.77162	-0.54336	H	2.20069	1.92532	-0.73882
H	1.97508	-3.30311	-0.49326	H	0.73292	-1.81057	0.83736
H	2.66857	-2.18535	-1.6549	H	1.58129	-1.82559	-0.70396
H	2.89369	-2.01231	1.39532	H	2.75112	-1.0717	2.02424
H	4.18369	-2.52176	0.31268	H	3.06136	-2.57969	1.16979
H	5.04368	0.7942	-1.07238	H	5.3266	-1.2588	-1.42846
H	5.37277	-0.93838	-1.10654	H	4.47211	-2.67256	-0.80857
H	3.94233	-0.29707	-1.92318	H	3.59842	-1.45357	-1.7456
H	1.84473	2.70333	-1.50195	H	5.82952	2.12253	-2.34119
H	3.11099	3.77427	-0.8872	H	4.95377	0.59053	-2.50651
H	3.52916	2.25658	-1.70111	H	4.06307	2.09999	-2.44034
H	-2.03745	-1.31433	-2.2886	H	-1.19385	-0.20374	1.14484
H	-1.23782	-0.34098	0.52544	H	-2.59482	0.23073	-1.55684
H	-3.87215	-0.27406	-1.06178	H	-3.5913	-0.50798	1.28194
H	-2.39923	3.43666	0.5223	H	-4.35976	-3.51222	-0.89134
H	-2.60114	2.05567	1.61957	H	-2.87896	-2.55589	-1.09293
H	-1.25197	2.08598	0.46759	H	-3.43785	-2.99251	0.53439
H	-2.84716	3.14494	-1.98073	H	-6.47775	-2.44743	-0.08862
H	-3.40573	1.57723	-2.59953	H	-6.50257	-0.74429	0.41233
H	-1.72922	1.76965	-2.04432	H	-5.67331	-1.94893	1.41494
H	-4.8529	1.86247	0.57166	H	-5.44176	-0.2739	-1.75115
H	-4.13229	-1.29072	3.33796	H	-6.36792	2.70061	0.84127
H	-5.67596	-0.67368	2.7407	H	-5.1652	3.80823	1.56974
H	-5.36419	-2.43619	2.73072	H	-5.14967	3.46435	-0.18488

atom	Con f. 7- 2s			atom	Con f. 7- 2t		
C	-6.33706	-1.69565	-0.93795	C	5.18509	-0.48475	2.60106
C	-5.02744	1.79211	1.61601	C	3.01091	2.89268	1.11597
C	-4.84986	1.548	0.31217	C	2.92563	2.05613	0.07462
C	-3.70306	0.69956	-0.2219	C	2.64638	0.57546	0.30184
C	-4.02363	-0.8434	-0.25795	C	3.92489	-0.34561	0.37772
C	-5.18454	-1.07756	-1.20027	C	4.82474	0.15726	1.48865
C	-2.37509	0.98821	0.50036	C	1.61767	0.04524	-0.71329
C	-1.20327	0.24874	-0.14398	C	1.19689	-1.40747	-0.40126
C	-1.47256	-1.27316	-0.18363	C	2.42989	-2.31163	-0.35722
C	-2.79877	-1.56201	-0.88933	C	3.44329	-1.78521	0.66004
C	-4.3053	-1.40963	1.13816	C	4.76074	-0.31308	-0.92033
C	-5.7773	2.13642	-0.71958	C	3.0501	2.57272	-1.33817
C	0.11114	0.50442	0.56227	C	0.17682	-1.86367	-1.42848
C	1.33703	0.10786	-0.17236	C	-1.21793	-1.37451	-1.35181
O	0.1622	0.99031	1.69349	O	0.49129	-2.60866	-2.35969
C	2.55002	0.18982	0.38705	C	-1.72099	-0.63732	-0.35391
C	3.80792	-0.24576	-0.29147	C	-3.12645	-0.12419	-0.33925
O	4.87018	0.70417	0.00701	O	-3.66341	-0.64253	0.92495
C	4.35011	-1.62334	0.17716	C	-3.21842	1.43	-0.42643
C	4.61089	-1.65522	1.68454	C	-2.92257	2.08148	0.92718
C	3.38815	-2.72801	-0.24626	C	-2.26154	1.94651	-1.50254
O	5.56868	-1.86546	-0.54024	O	-4.52746	1.80452	-0.87938
C	4.87132	1.86592	-0.68263	C	-4.98616	-0.78837	1.0821
C	6.03159	2.72919	-0.2852	C	-5.31181	-1.53409	2.34013
O	4.02559	2.14688	-1.51242	O	-5.81442	-0.36056	0.28812
H	-3.55967	0.98752	-1.27278	H	2.18714	0.49234	1.2949
H	-1.08207	0.58538	-1.18398	H	0.71984	-1.41394	0.58682
H	-7.09175	-1.81961	-1.71043	H	5.85248	-0.01646	3.32039
H	-6.57915	-2.09783	0.04129	H	4.84427	-1.48628	2.84547
H	-5.85487	2.4055	1.96462	H	3.20767	3.9537	0.98103
H	-4.36404	1.39737	2.379	H	2.89076	2.54311	2.13825
H	-5.01646	-0.70189	-2.21105	H	5.21743	1.15935	1.32411
H	-2.43554	0.70647	1.55517	H	2.02265	0.08042	-1.7301
H	-2.18791	2.06708	0.48079	H	0.7425	0.70092	-0.70114
H	-0.66111	-1.79231	-0.70453	H	2.13478	-3.33268	-0.09059
H	-1.48907	-1.6584	0.84238	H	2.87418	-2.36671	-1.3561
H	-2.71175	-1.24899	-1.93788	H	2.98325	-1.8145	1.65575
H	-2.98885	-2.64155	-0.89657	H	4.3126	-2.4516	0.69623
H	-5.2063	-0.97047	1.57218	H	5.25101	0.65502	-1.05401
H	-4.44595	-2.49408	1.08625	H	5.54652	-1.07461	-0.87109
H	-3.47999	-1.21684	1.82654	H	4.1586	-0.51096	-1.81056
H	-6.56773	2.73581	-0.25814	H	3.34799	3.6249	-1.34196
H	-5.2218	2.77696	-1.41719	H	3.77909	2.0076	-1.92596
H	-6.24855	1.35251	-1.32209	H	2.09451	2.49438	-1.87065
H	1.21754	-0.25818	-1.18841	H	-1.84314	-1.66045	-2.19507
H	2.64685	0.56792	1.40272	H	-1.11391	-0.35921	0.5031
H	3.6744	-0.28495	-1.37457	H	-3.70818	-0.54777	-1.16155
H	5.06309	-2.61431	1.95236	H	-2.9417	3.16931	0.81425
H	5.29425	-0.8542	1.98176	H	-3.67284	1.79992	1.67077
H	3.68134	-1.54254	2.25045	H	-1.9365	1.79136	1.30411
H	3.82359	-3.70035	0.00154	H	-2.47788	3.00449	-1.67806
H	3.20877	-2.69387	-1.32537	H	-2.40065	1.40566	-2.44364
H	2.43066	-2.6357	0.2707	H	-1.21687	1.8552	-1.19664
H	6.17675	-1.14232	-0.32114	H	-5.16907	1.19647	-0.46757
H	5.97732	2.94743	0.78578	H	-4.80731	-1.06491	3.18961
H	6.96911	2.19499	-0.46739	H	-6.38981	-1.5411	2.49852
H	6.014	3.65729	-0.85601	H	-4.94033	-2.56072	2.2596

8. The 1D and 2D NMR, MS, UV and IR spectra of 1–7

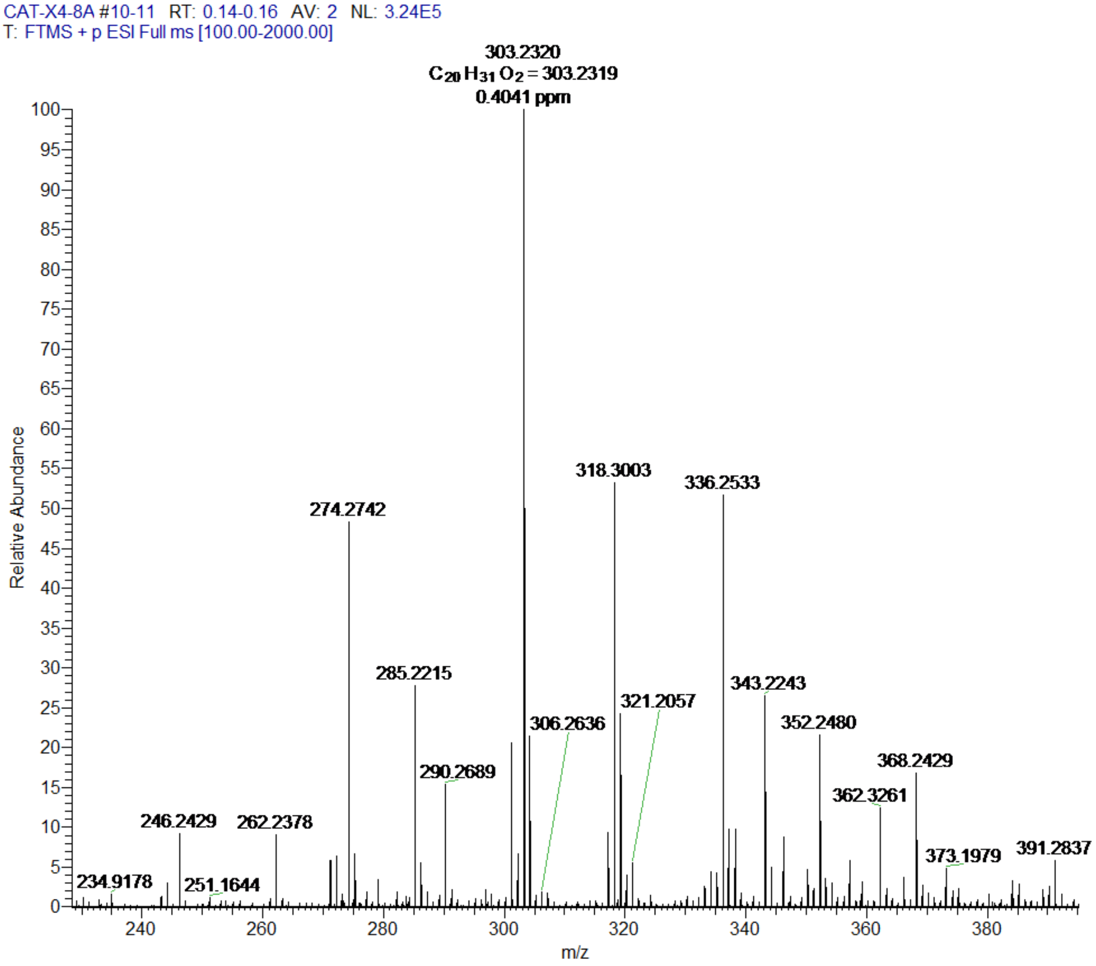

Figure S16. The positive HRESIMS spectrum of compound **1**

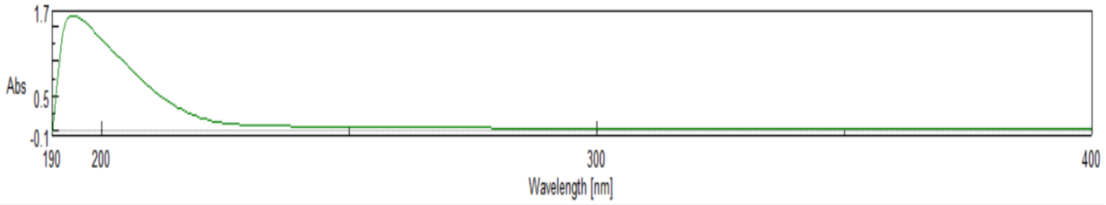

Figure S17. UV spectrum of compound **1**

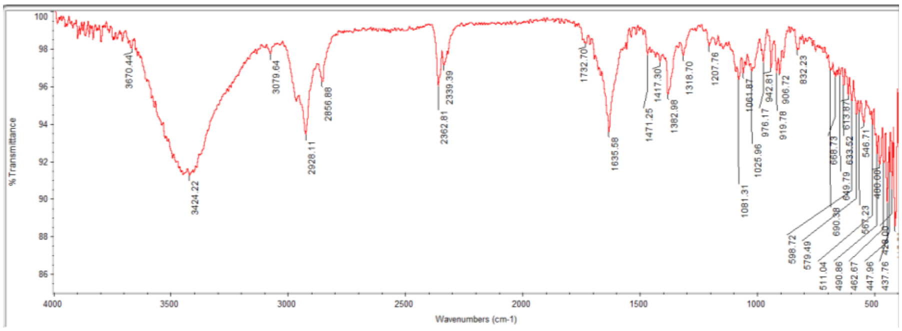

Figure S18. IR spectrum of compound **1**

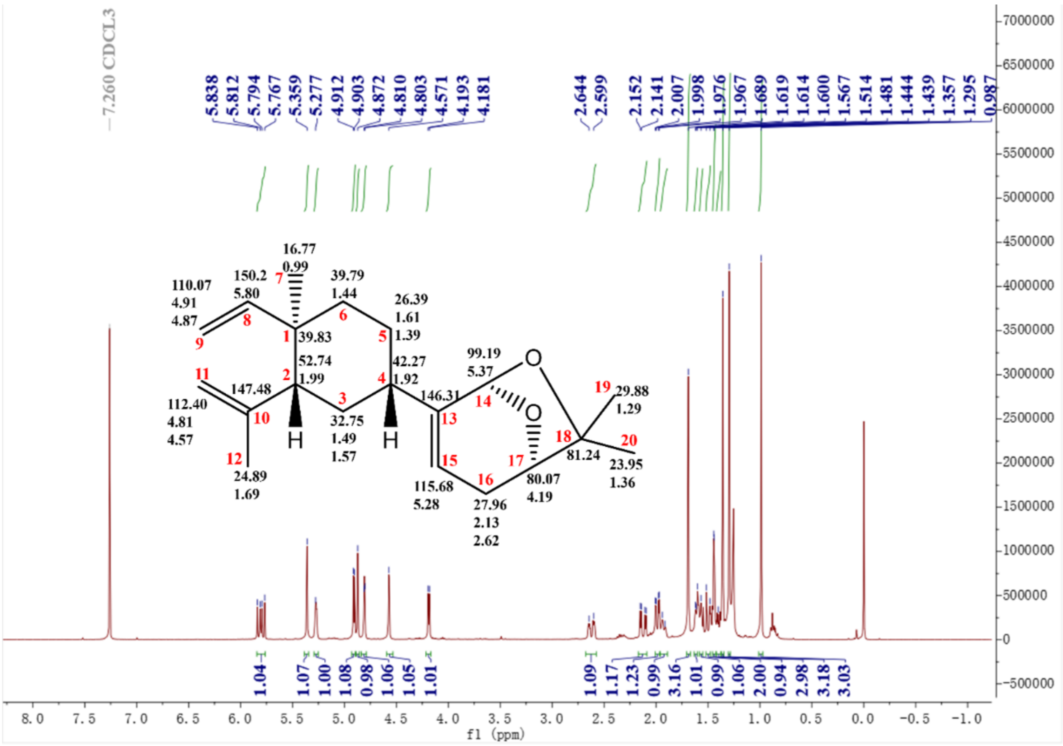

Figure S19. ^{1}H NMR spectrum (500 MHz, $CDCl_3$) of compound **1**

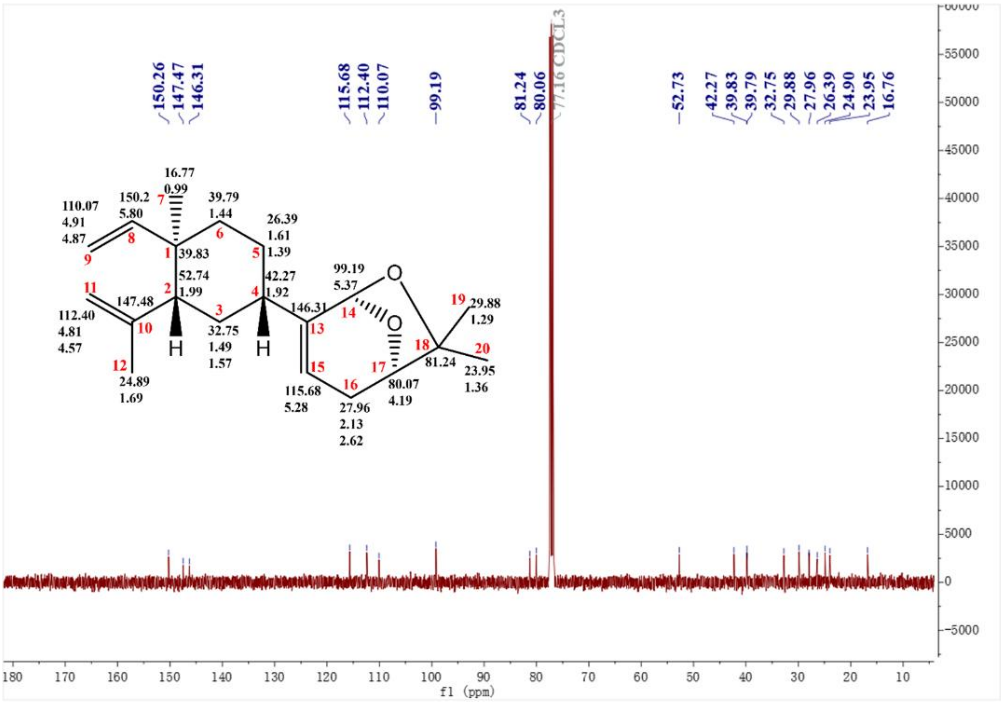

Figure S20. ^{13}C NMR spectrum (125 MHz, $CDCl_3$) of compound **1**

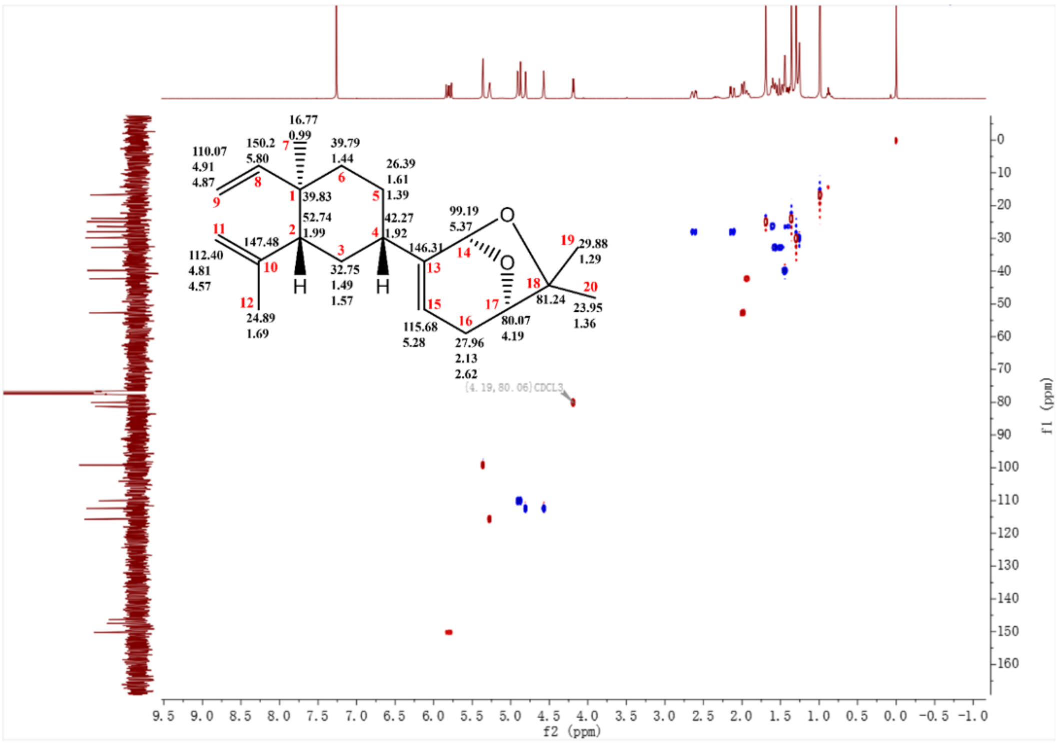

Figure S21. HSQC (500 M Hz, $CDCl_3$) of compound **1**

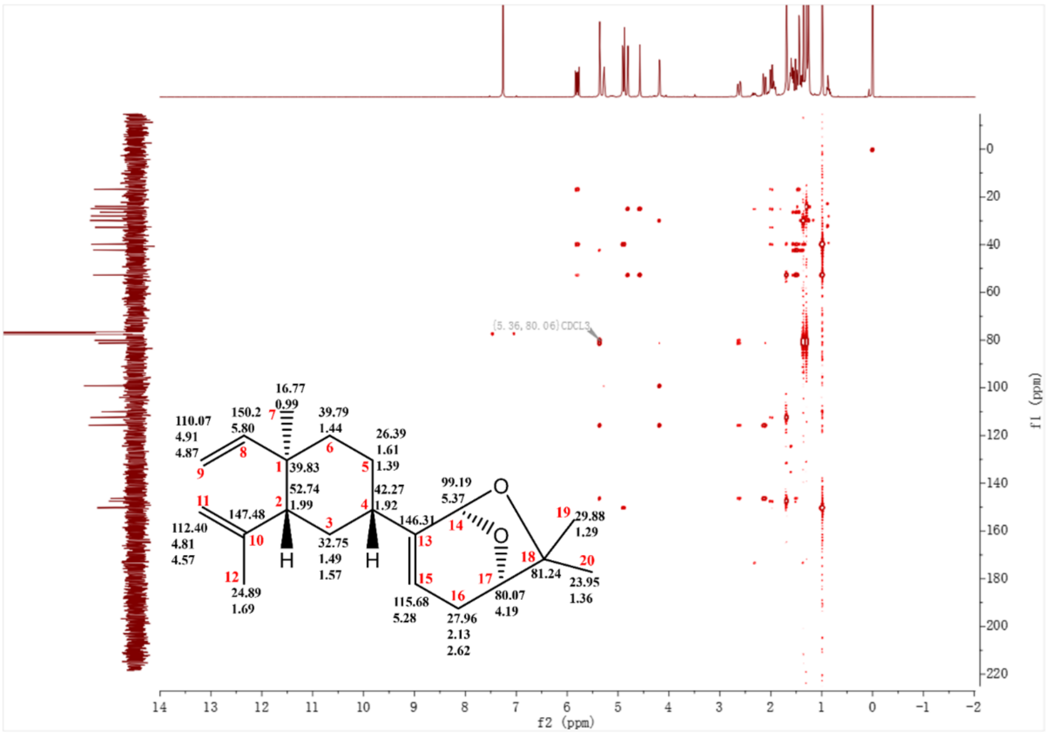

Figure S22. HMBC (125 MHz, $CDCl_3$) of compound **1**

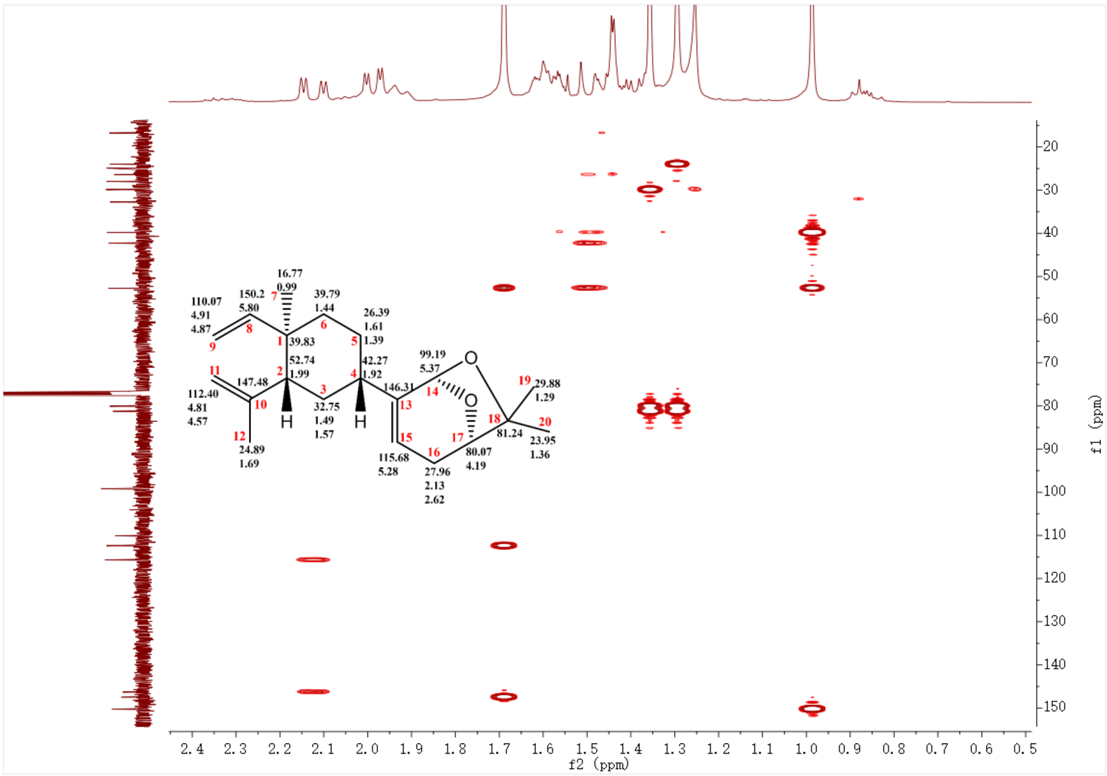

Figure S23. HMBC (125 MHz, $CDCl_3$) of compound **1**

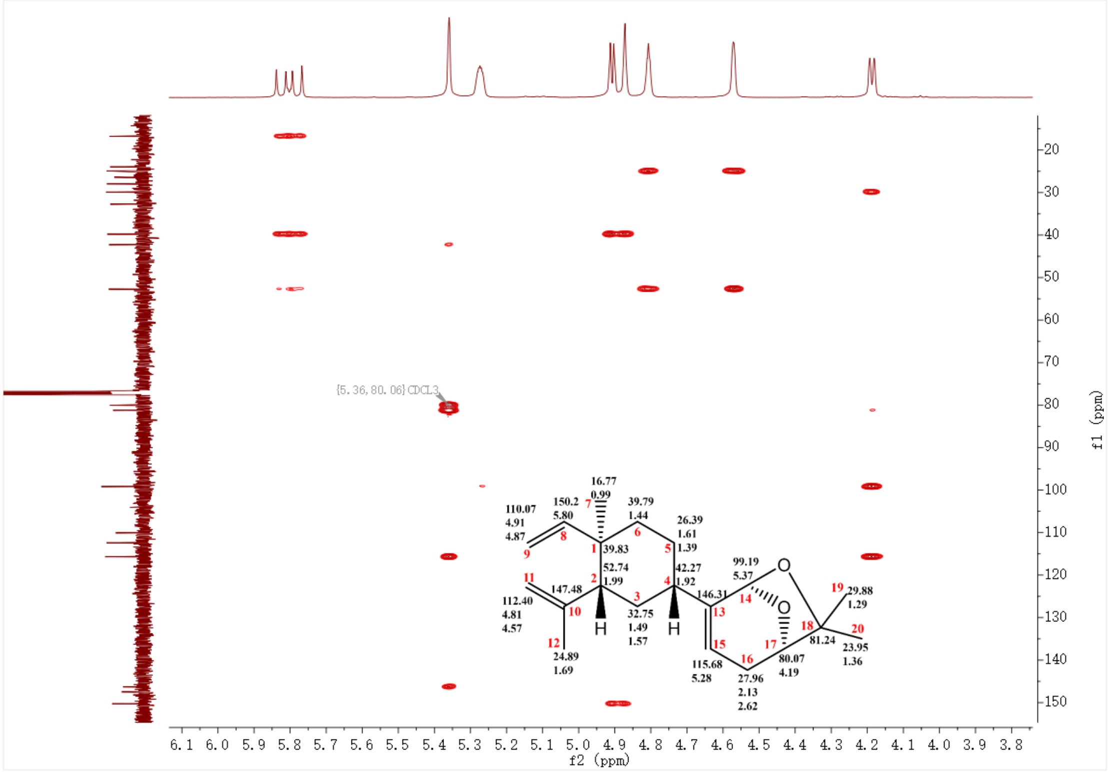

Figure S24. HMBC (125 MHz, $CDCl_3$) of compound **1**

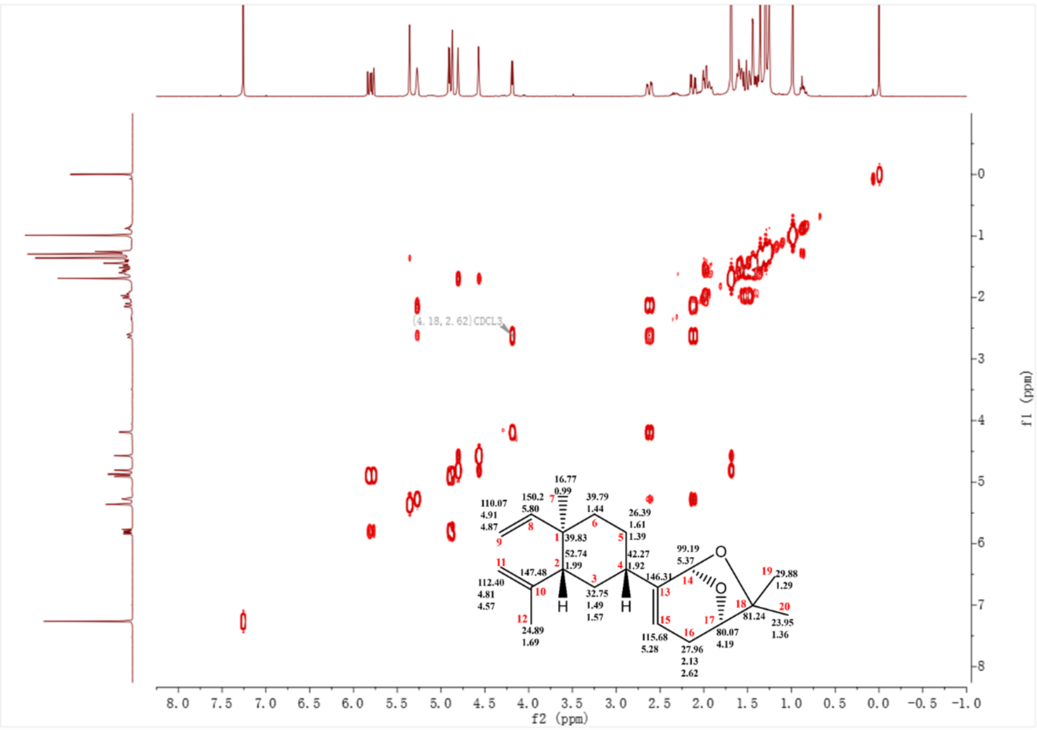

Figure S25. ^1H-^1H COSY (500 MHz, $CDCl_3$) of compound **1**

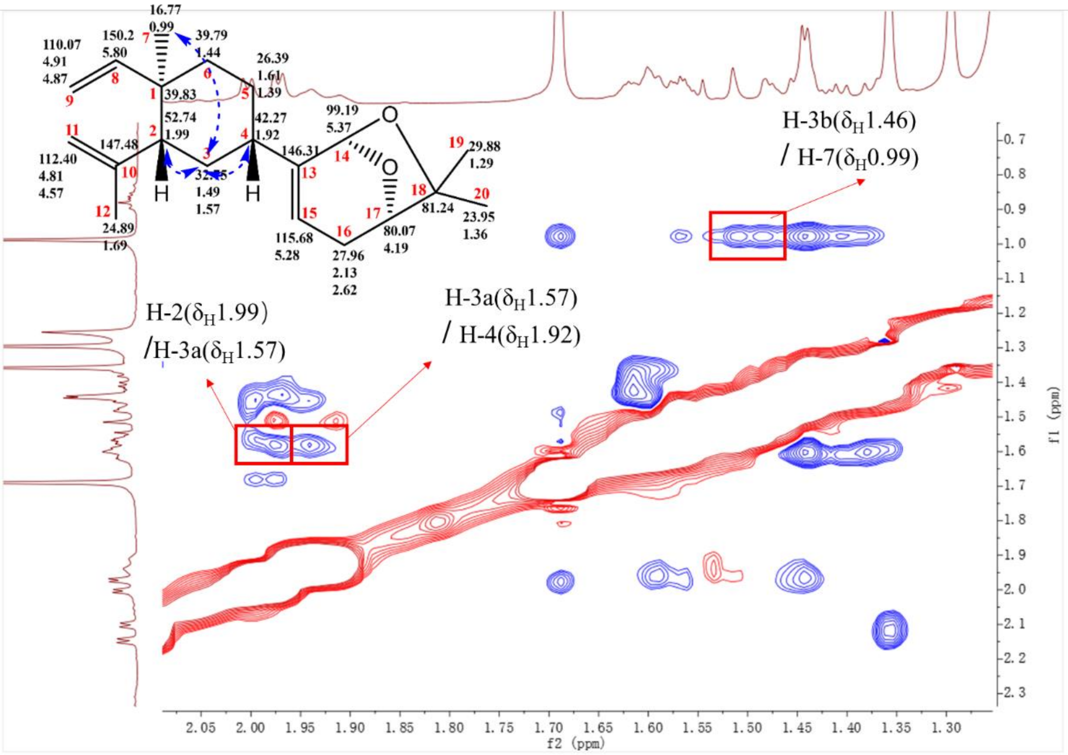

Figure S26. NOESY (500 MHz, $CDCl_3$) of compound **1**

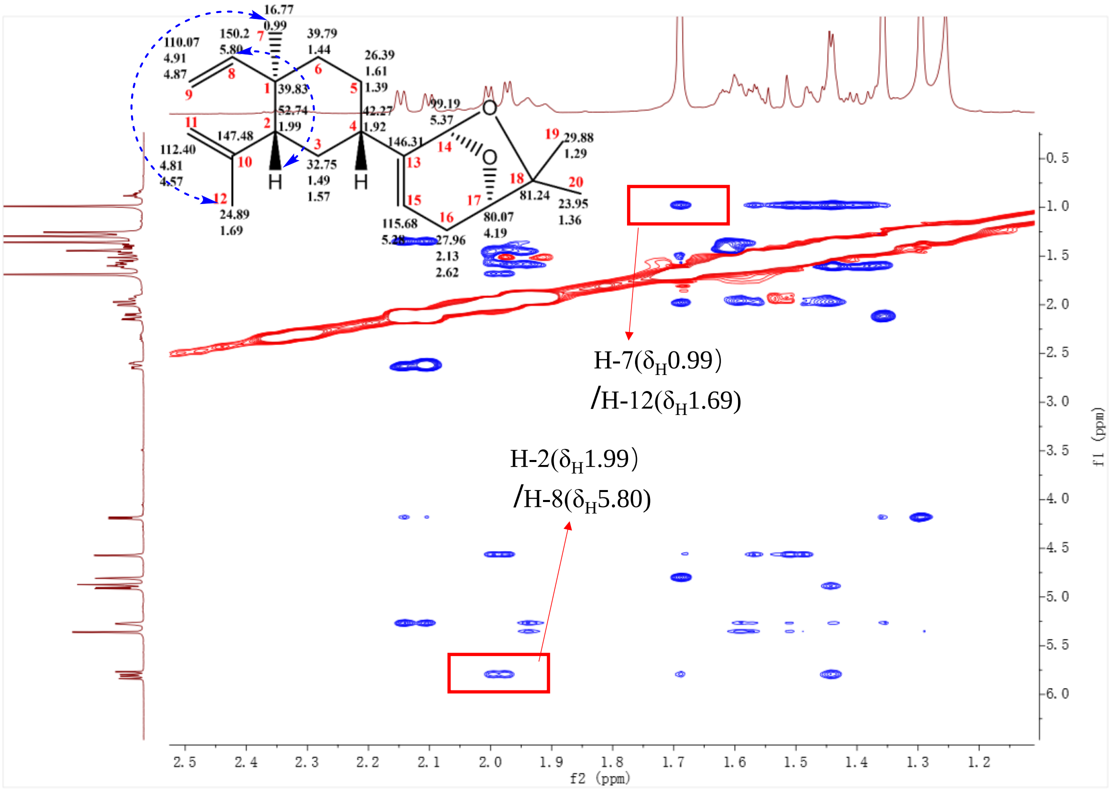

Figure S27. NOESY (500 MHz, CDCl3) of compound **1**

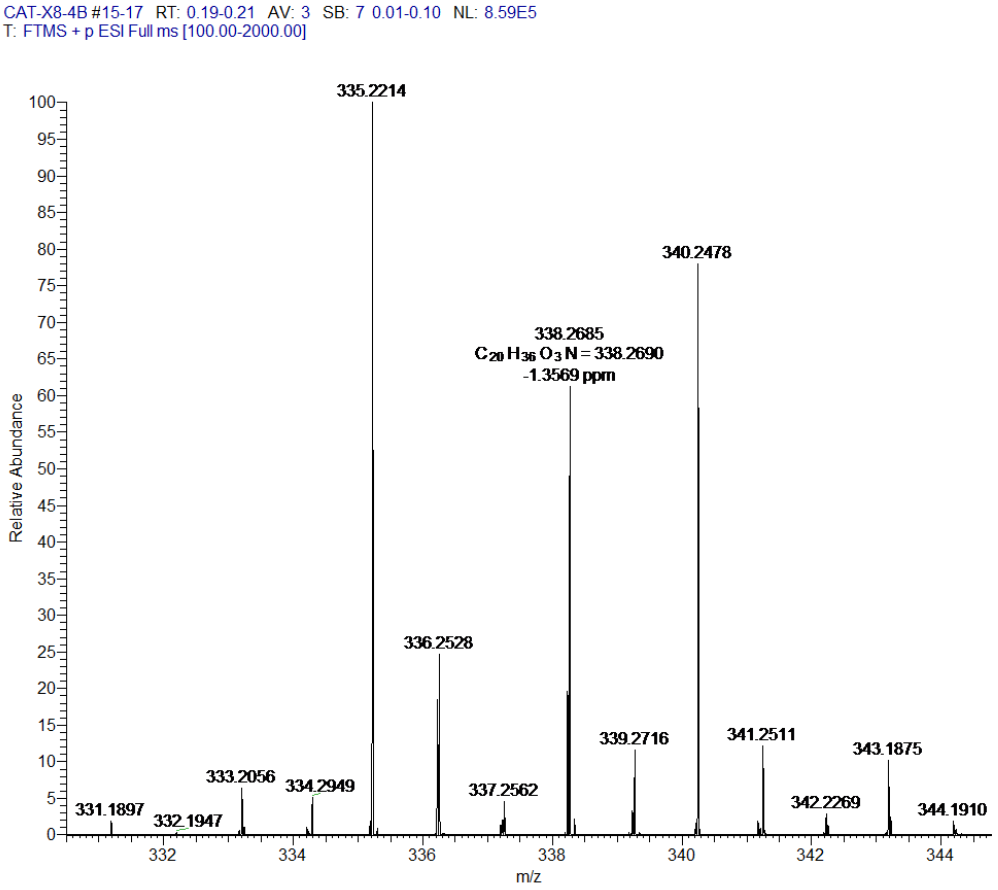

Figure S28. The positive HRESIMS spectrum of compound **2**

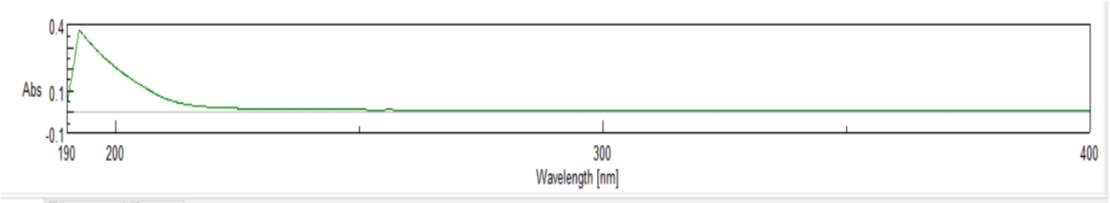

Figure S29. UV spectrum of compound **2**

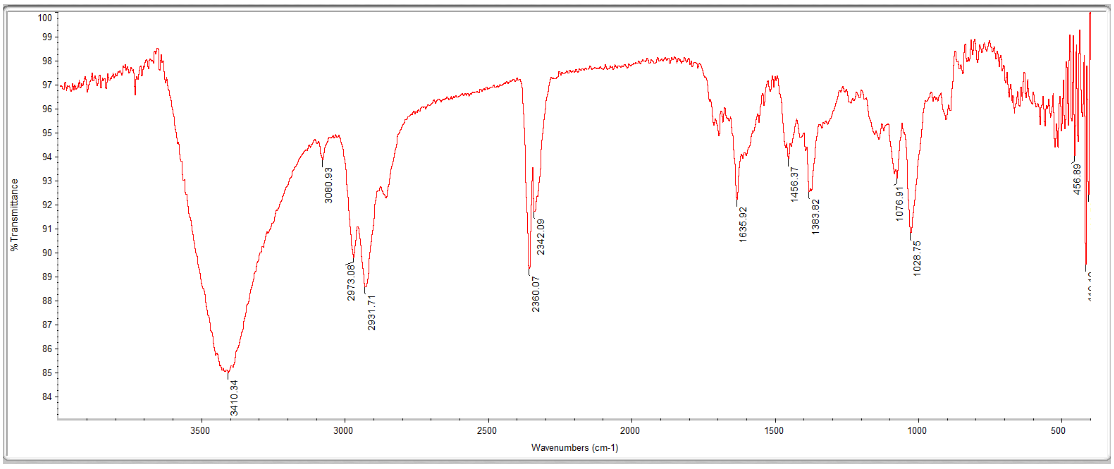

Figure S30. IR spectrum of compound **2**

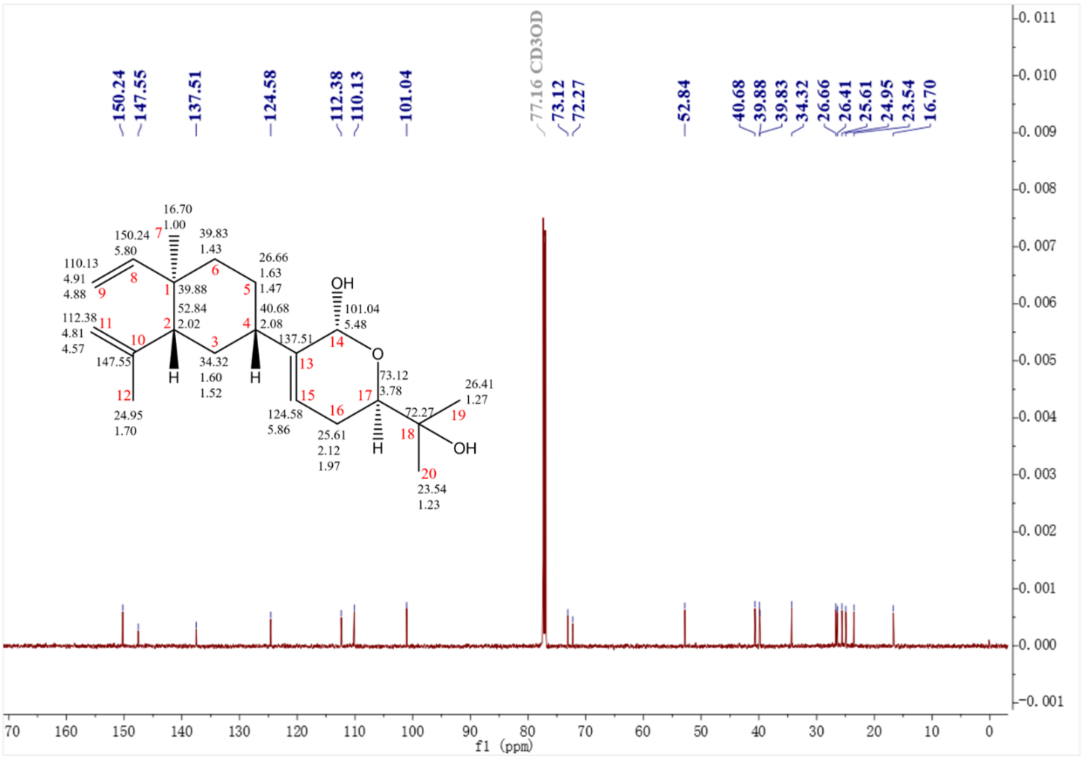

Figure S31. ^{1}H NMR spectrum (600 MHz, $CDCl_3$) of compound **2**

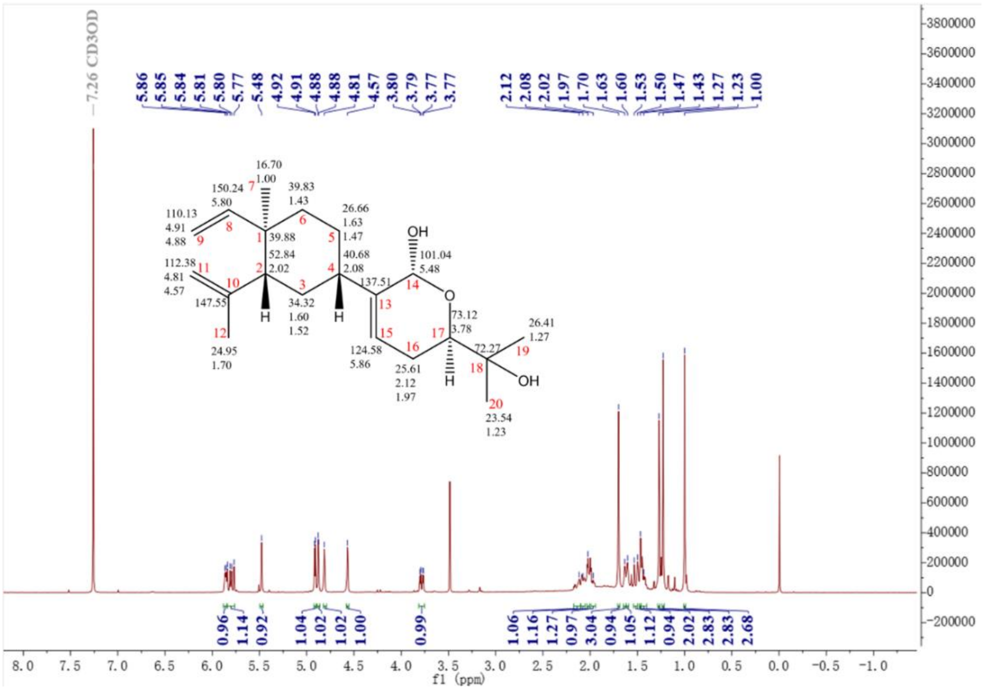

Figure S32. ^{13}C NMR spectrum (150 MHz, $CDCl_3$) of compound **2**

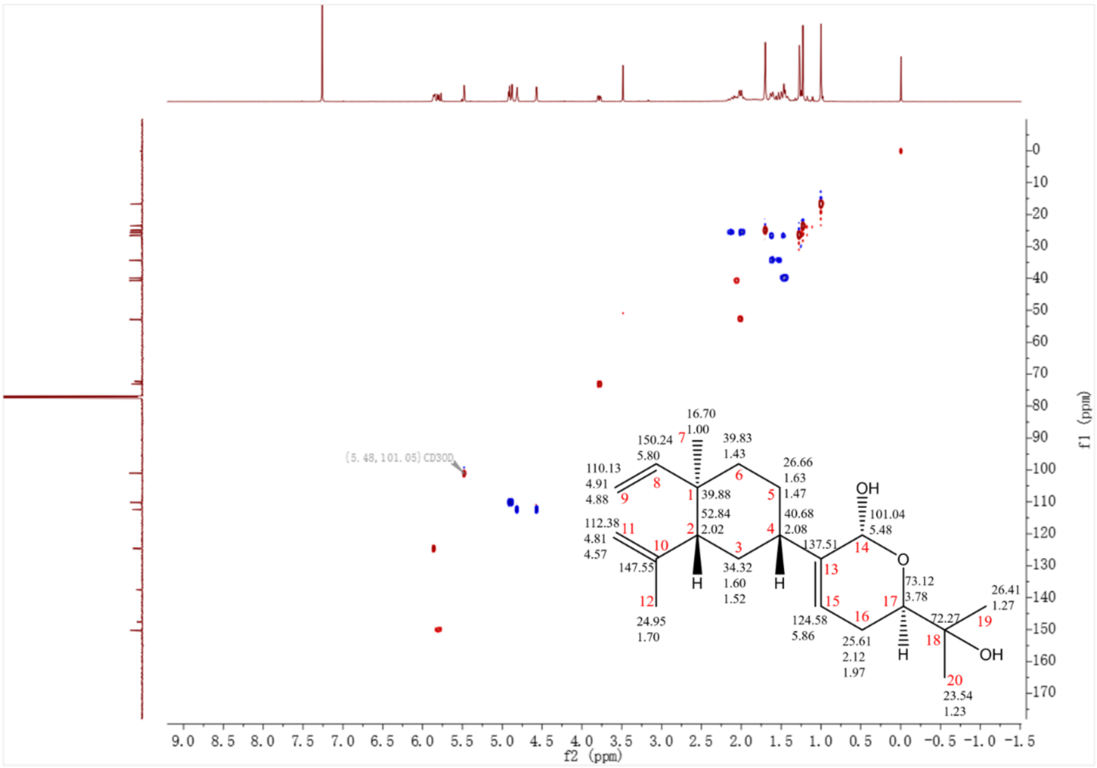

Figure S33. HSQC (600 MHz, $CDCl_3$) of compound **2**

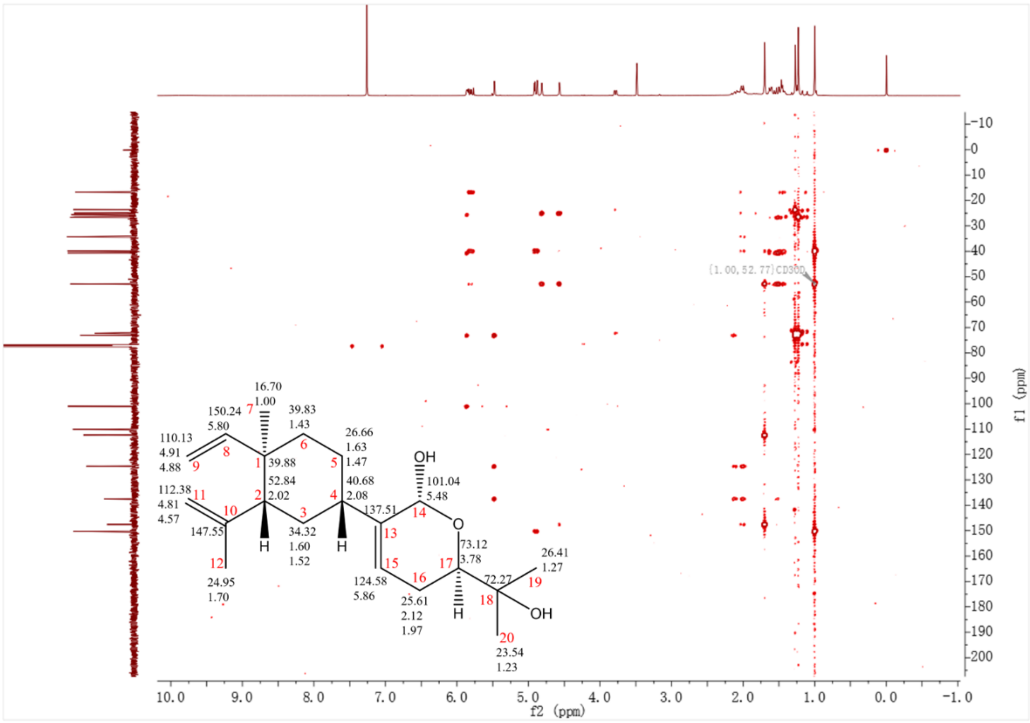

Figure S34. HMBC (150 MHz, $CDCl_3$) of compound **2**

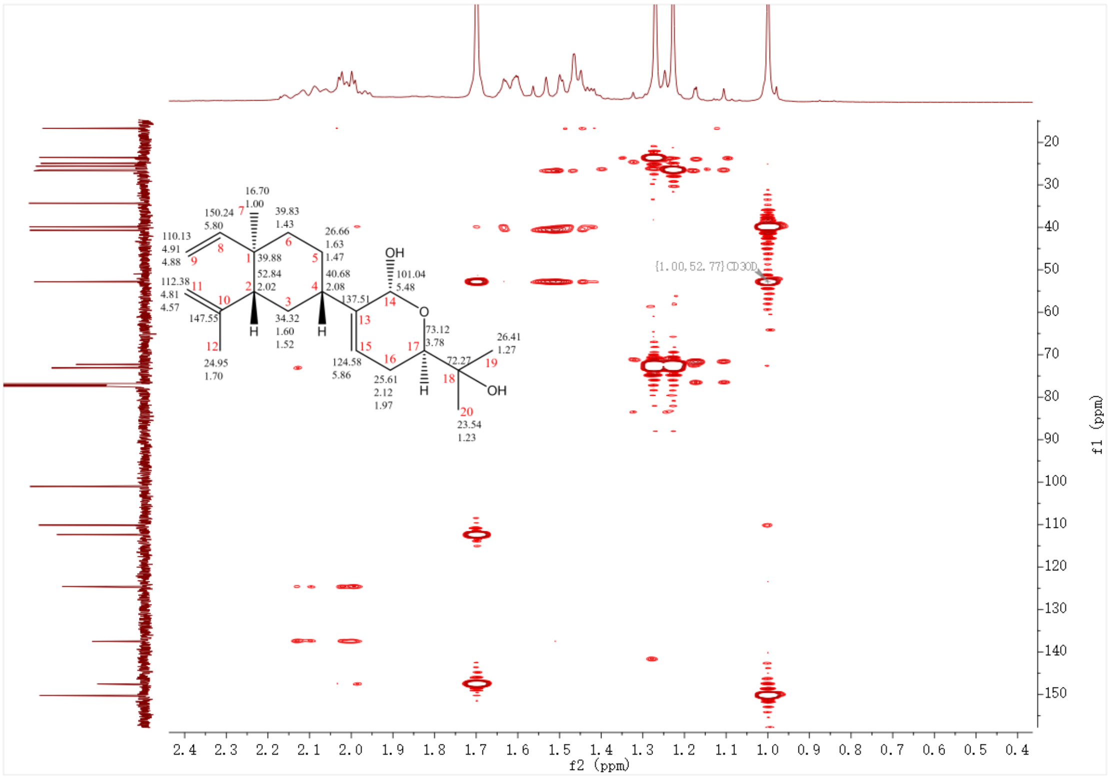

Figure S35. HMBC (150 MHz, $CDCl_3$) of compound **2**

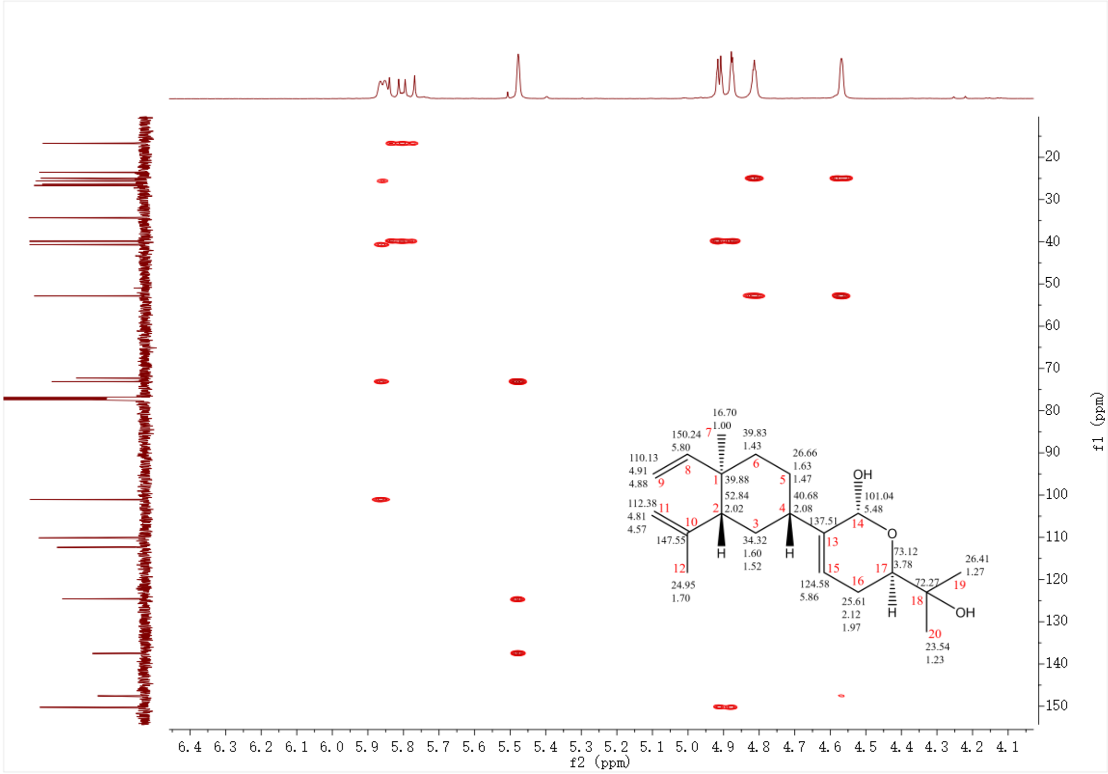

Figure S36. HMBC (150 MHz, CDCl3) of compound **2**

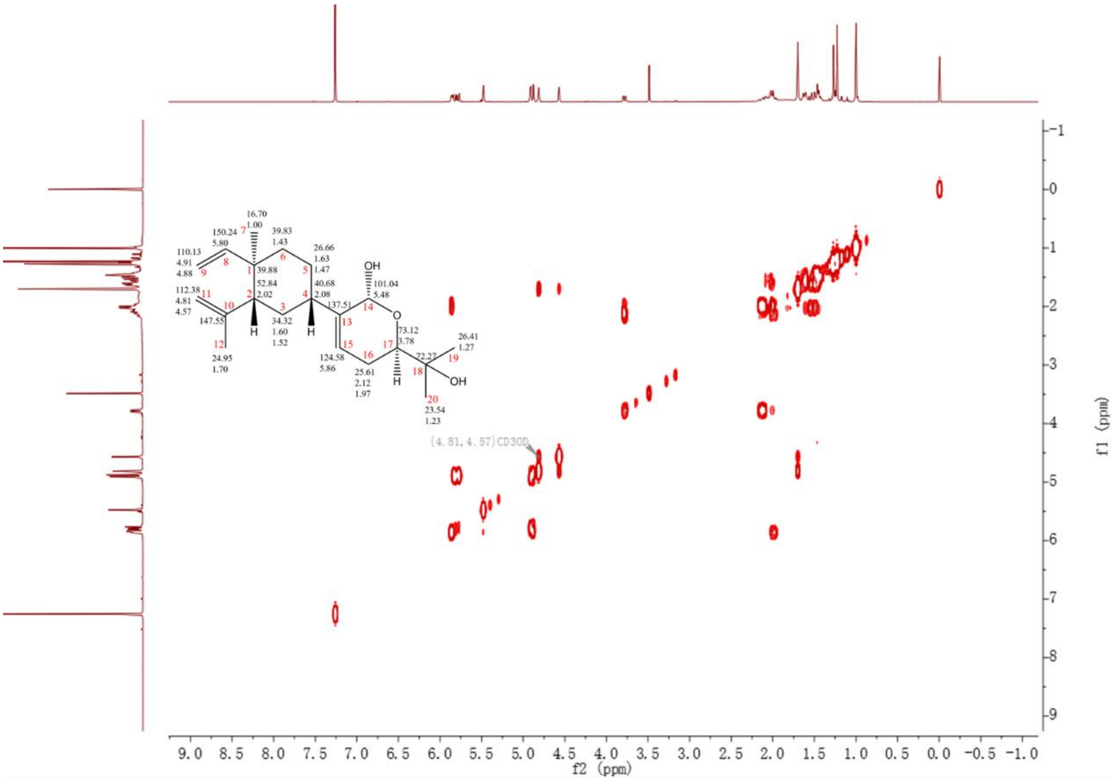

Figure S37. ^{1}H-^{1}H COSY (600 MHz, CDCl3) of compound **2**

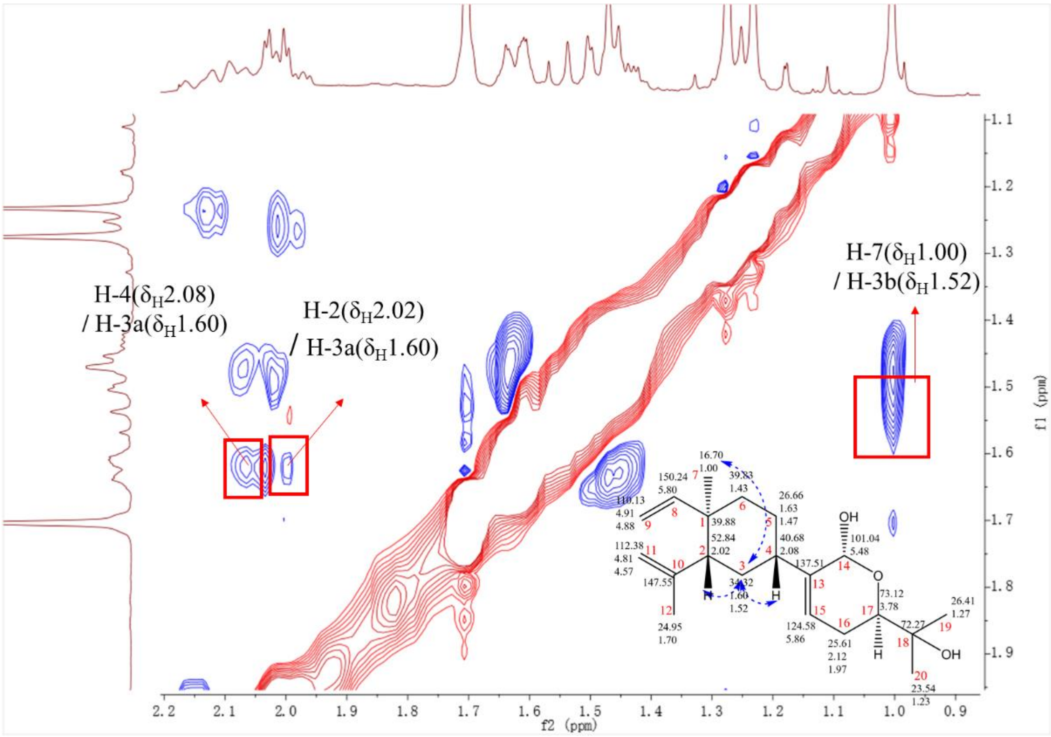

Figure S38. NOESY (600 MHz, CDCl3) of compound **2**

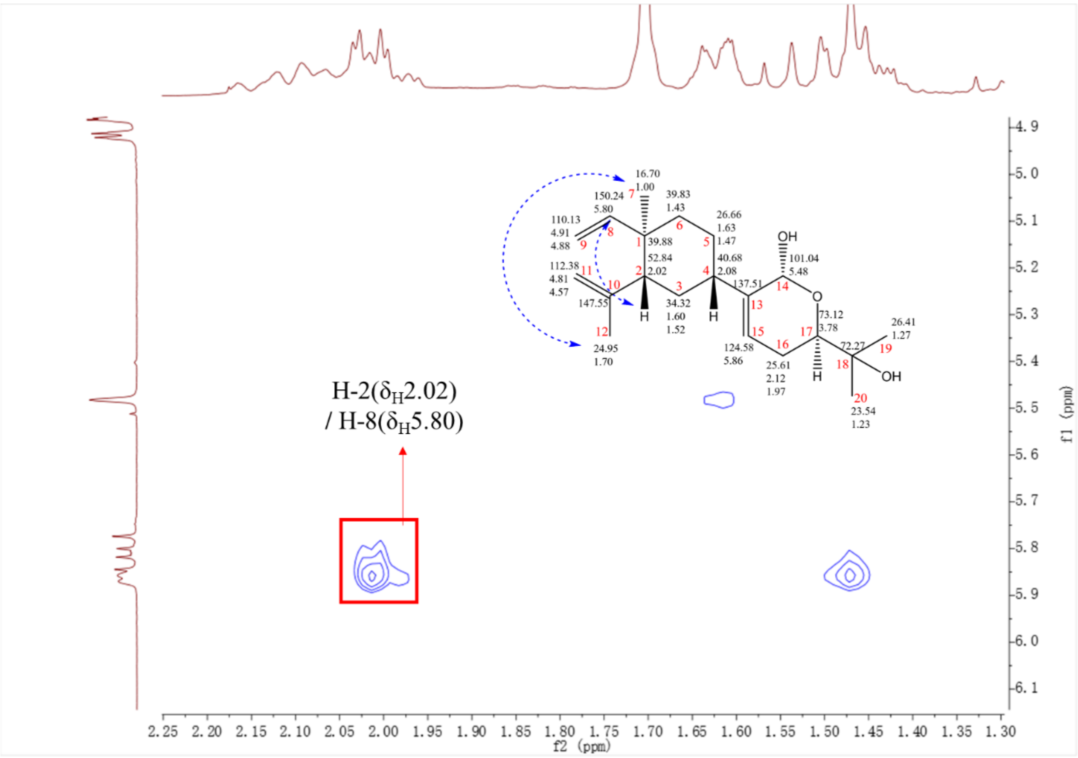

Figure S39. NOESY (600 MHz, CDCl3) of compound **2**

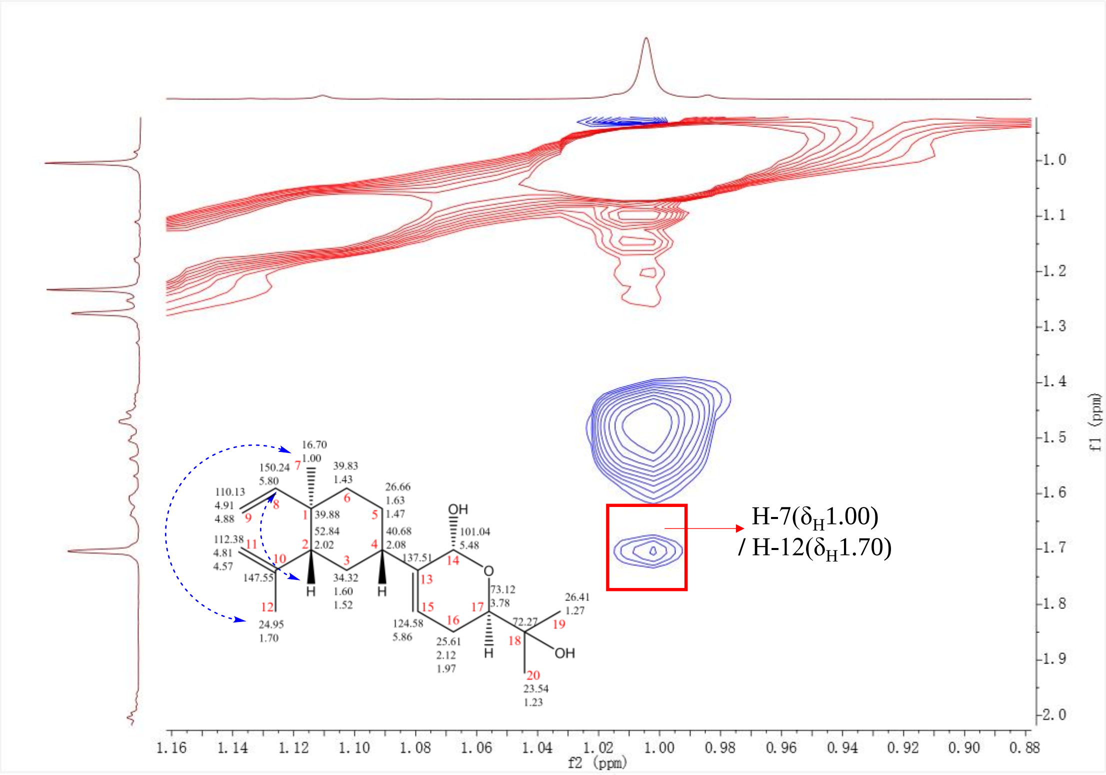

Figure S40. NOESY (600 MHz, CDCl₃) of compound **2**

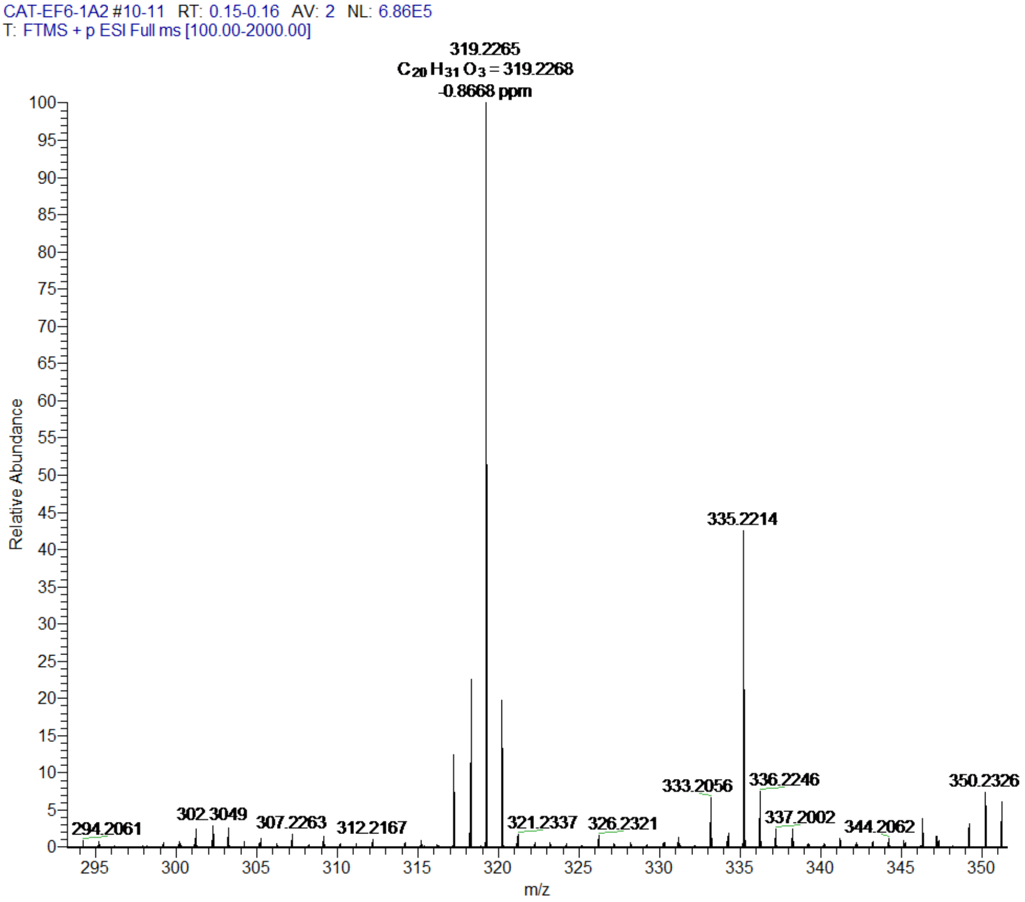

Figure S41. The positive HRESIMS spectrum of compound **3**

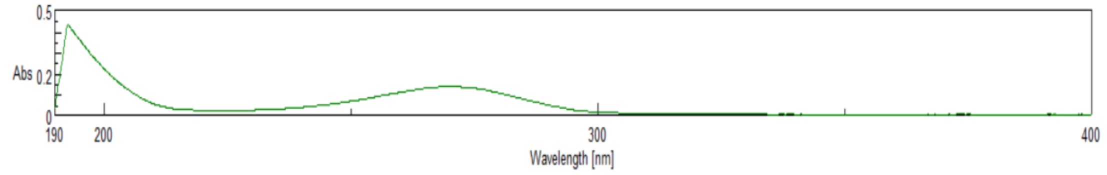

Figure S42. UV spectrum of compound **3**

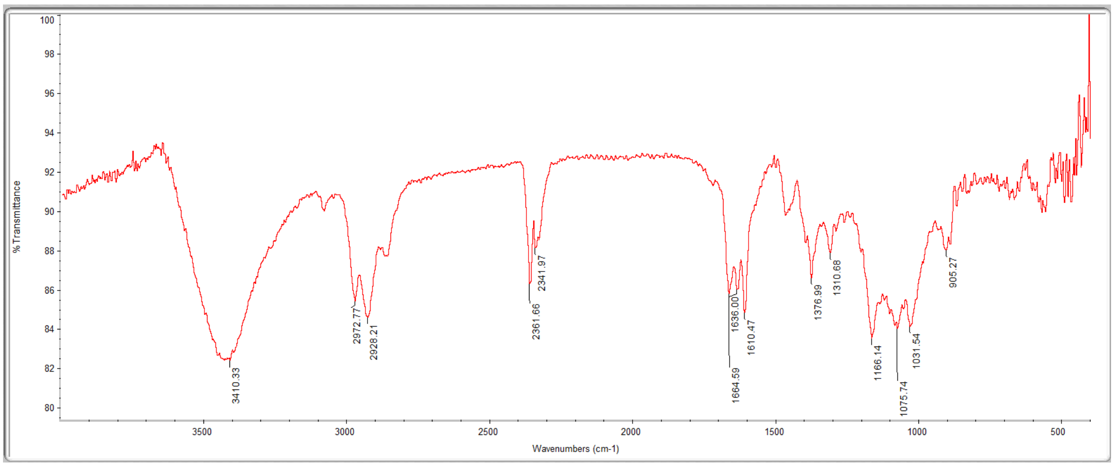

Figure S43. IR spectrum of compound **3**

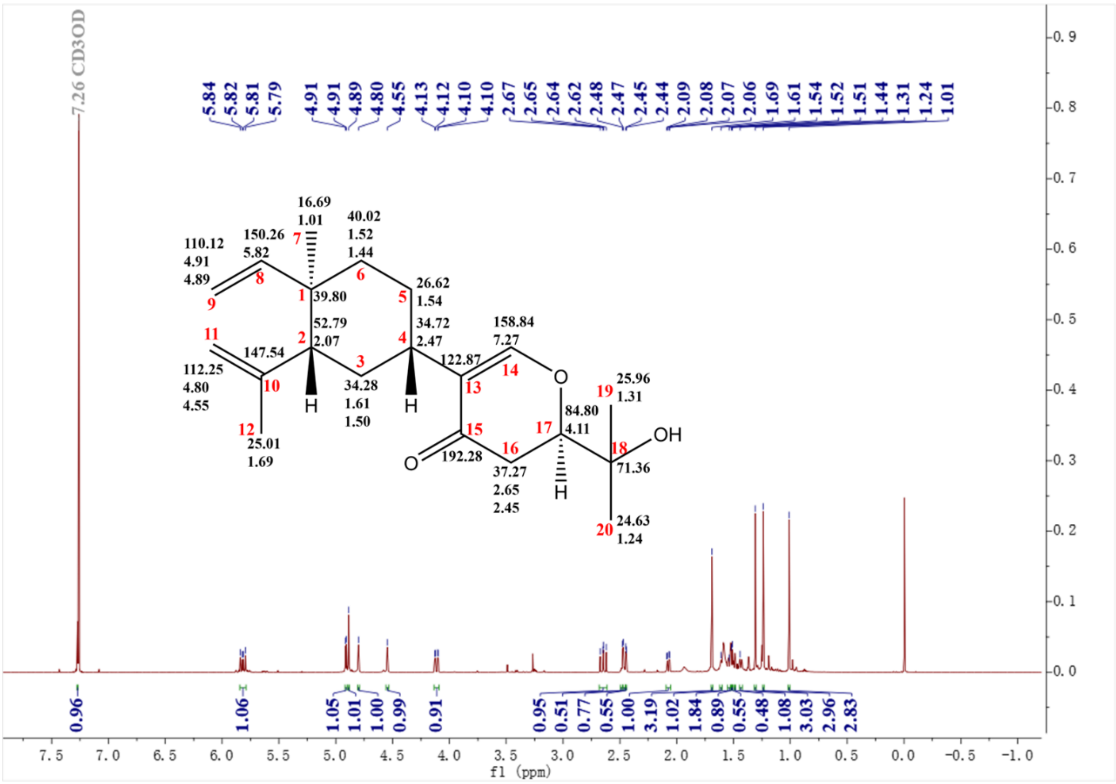

Figure S44. ^{1}H NMR spectrum (600 MHz, $CDCl_3$) of compound **3**

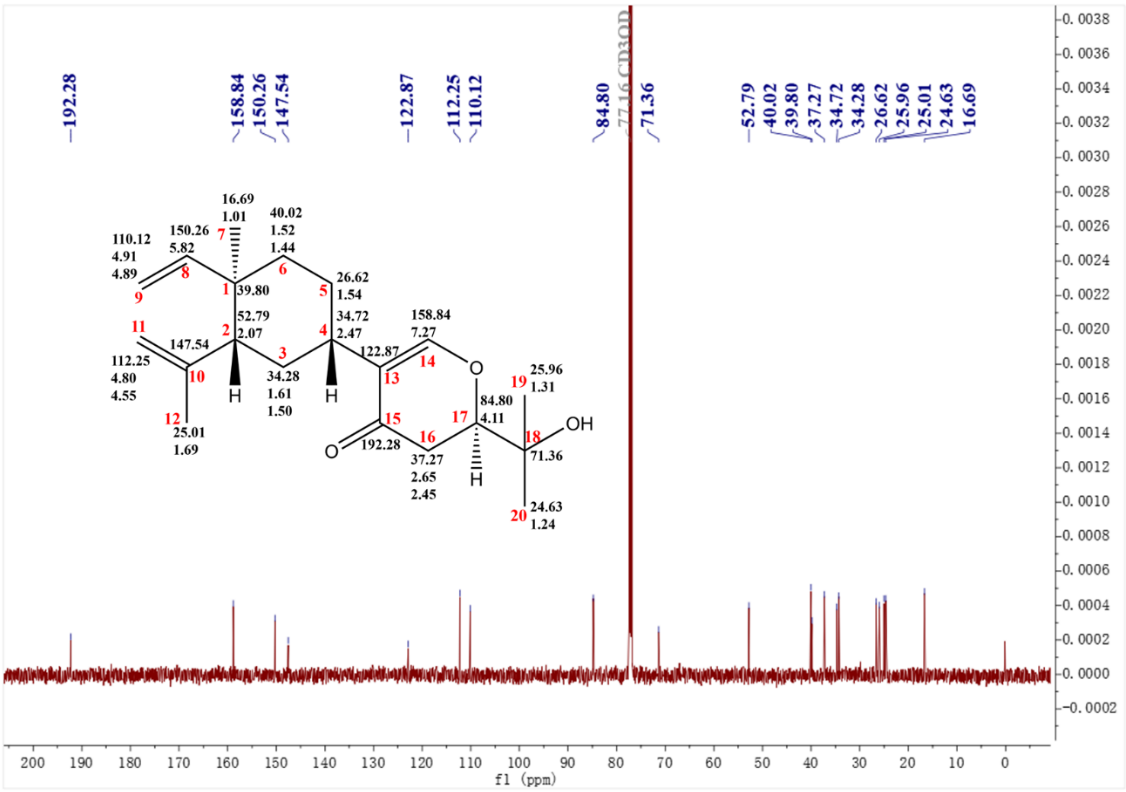

Figure S45. ^{13}C NMR spectrum (150 MHz, $CDCl_3$) of compound **3**

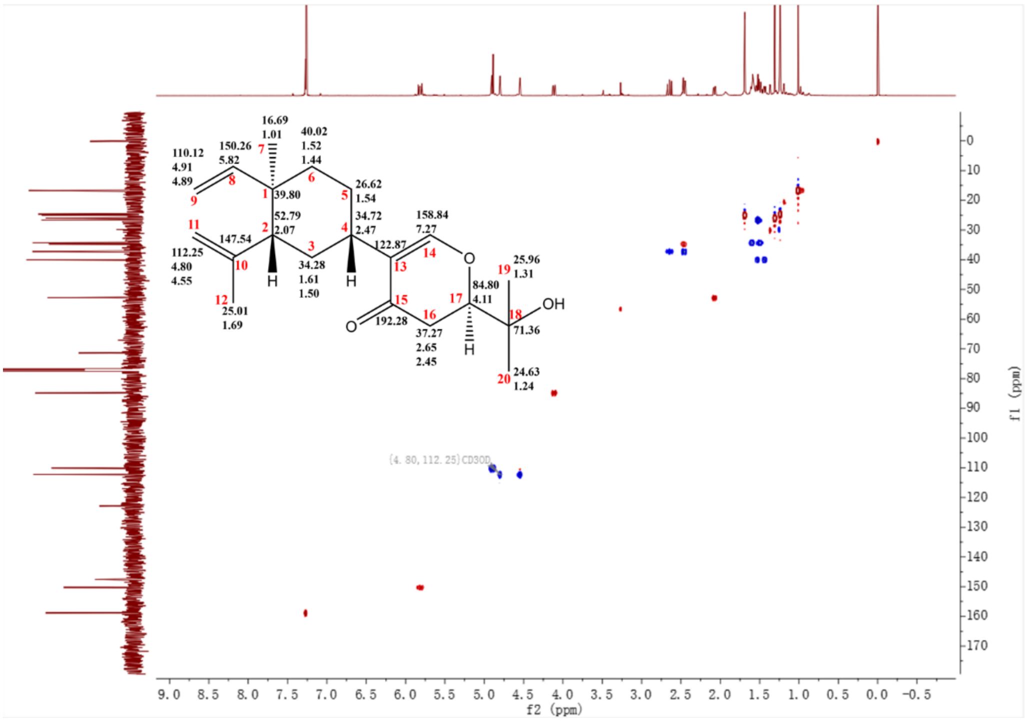

Figure S46. HSQC (600 MHz, $CDCl_3$) of compound **3**

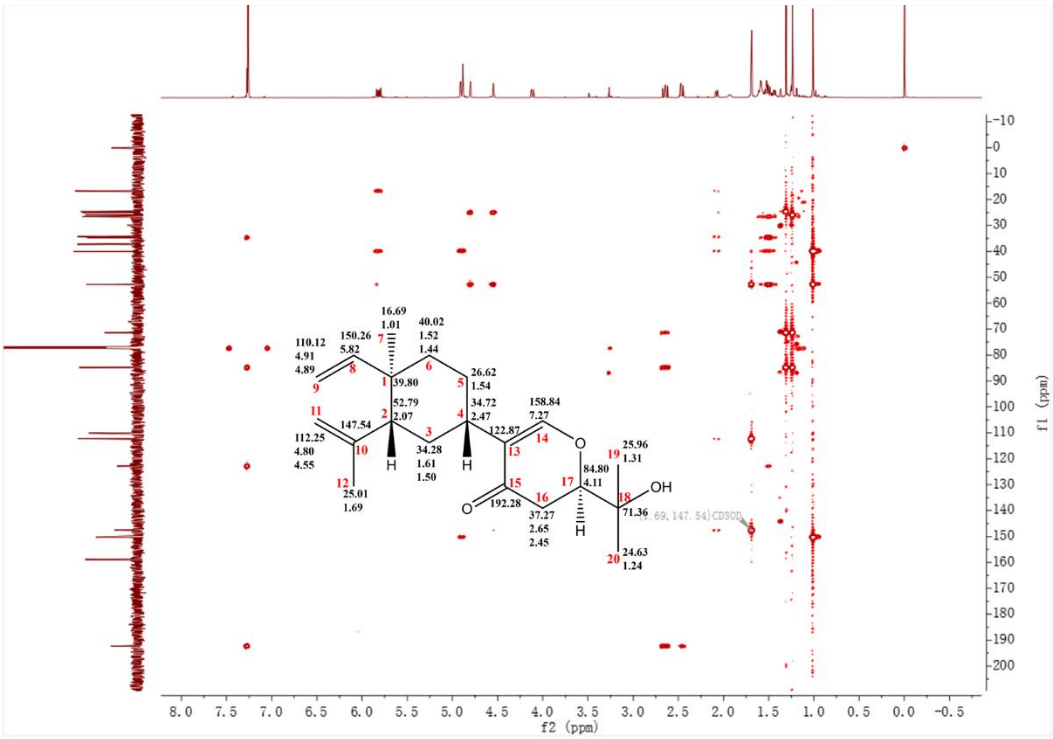

Figure S47. HMBC (150 MHz, $CDCl_3$) of compound **3**

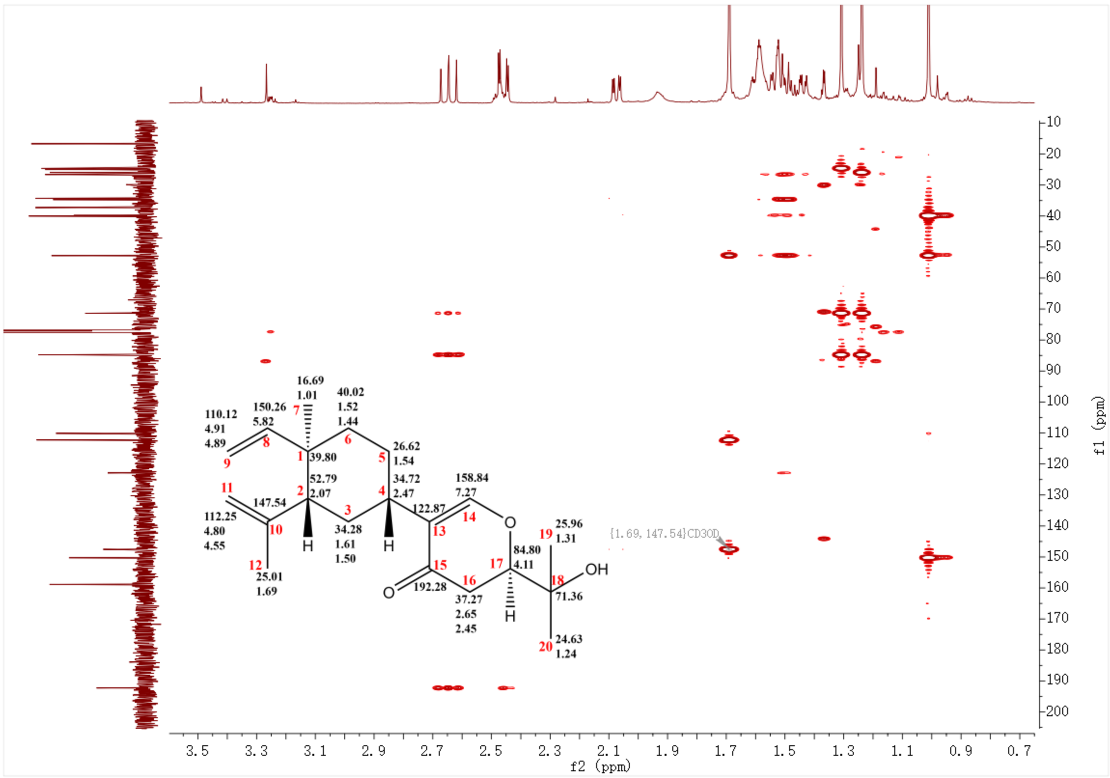

Figure S48. HMBC (150 MHz, $CDCl_3$) of compound **3**

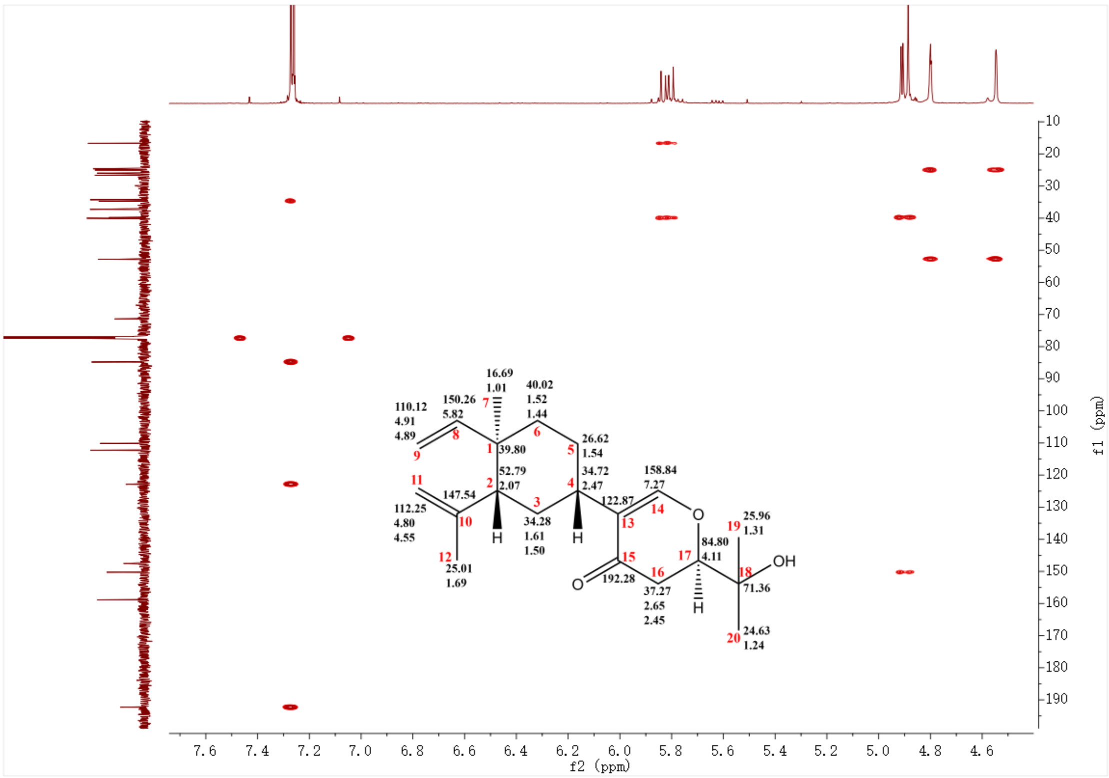

Figure S49. HMBC (150 MHz, $CDCl_3$) of compound **3**

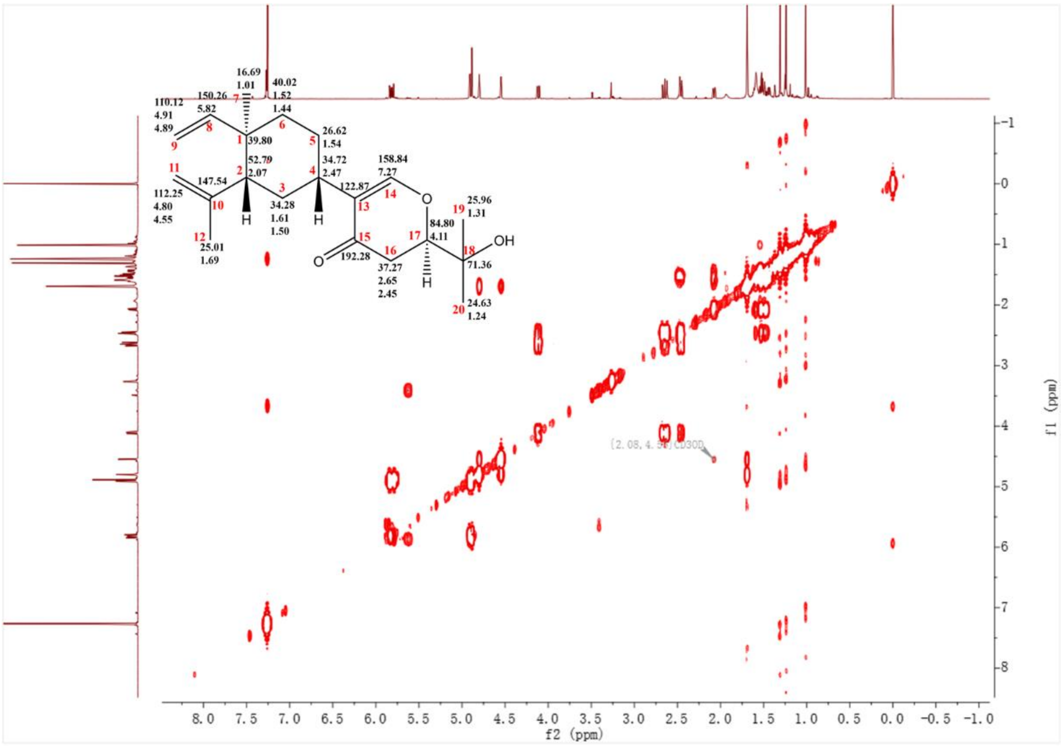

Figure S50. ^1H-^1H COSY (600 MHz, $CDCl_3$) of compound **3**

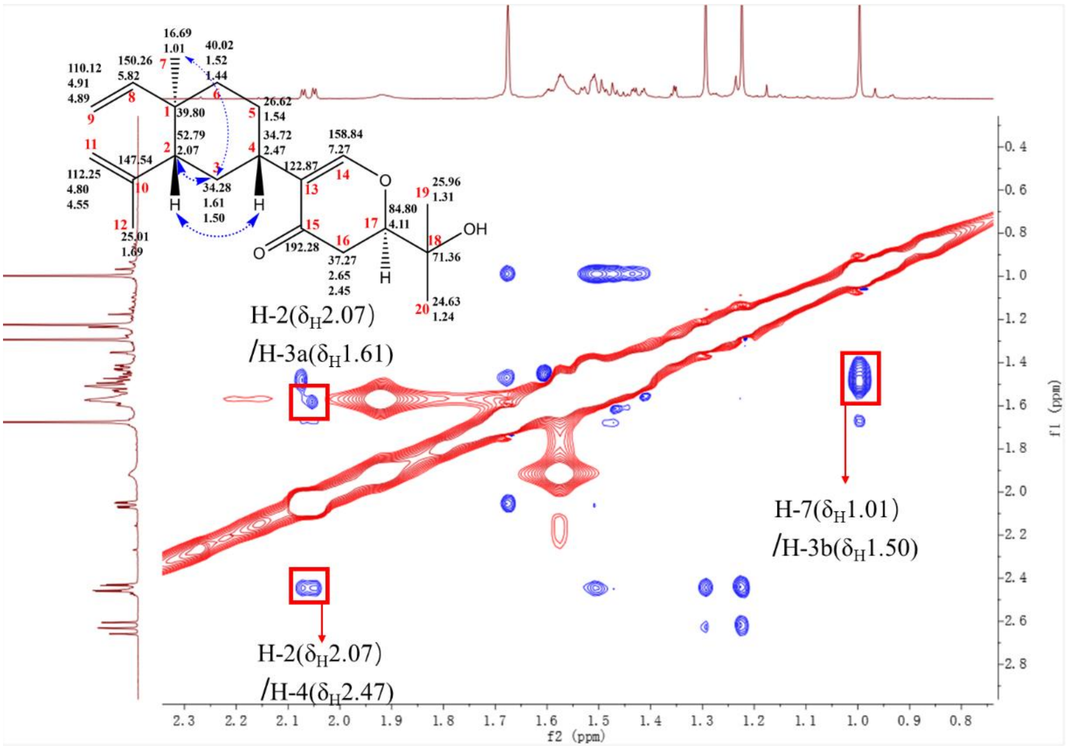

Figure S51. NOESY (600 MHz, $CDCl_3$) of compound **3**

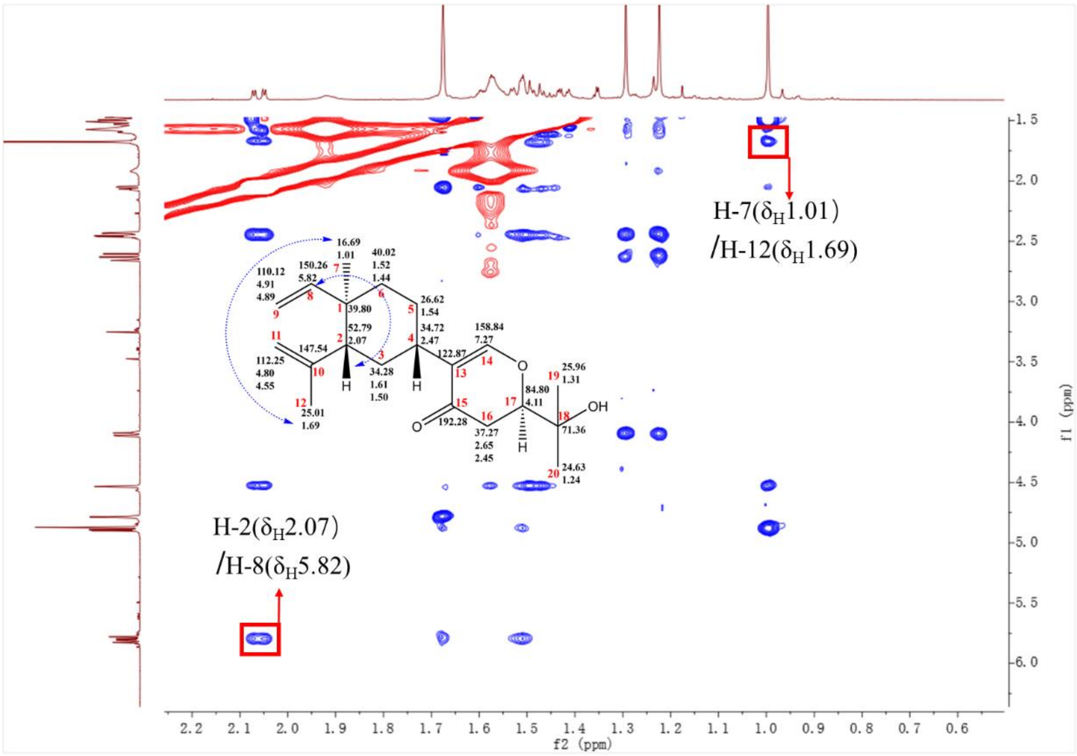

Figure S52. NOESY (600 MHz, $CDCl_3$) of compound **3**

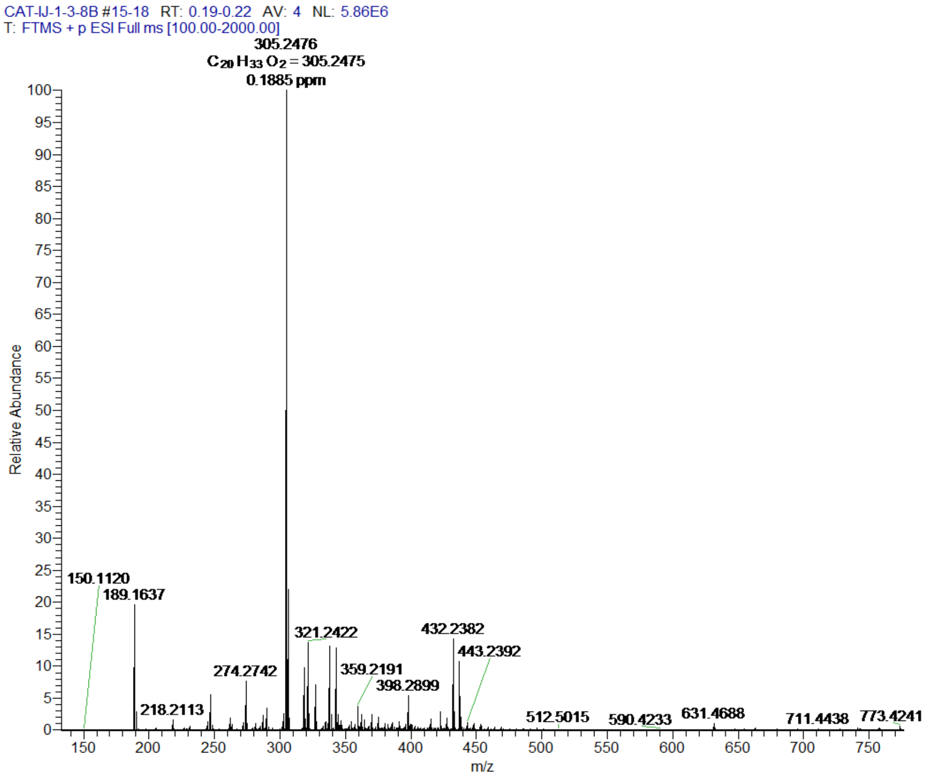

Figure S53. The positive HRESIMS spectrum of compound **4**

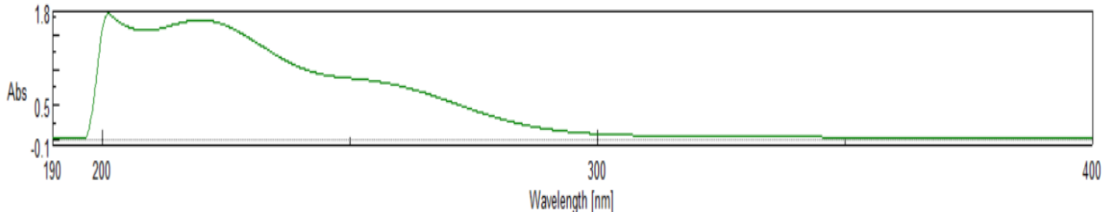

Figure S54. UV spectrum of compound **4**

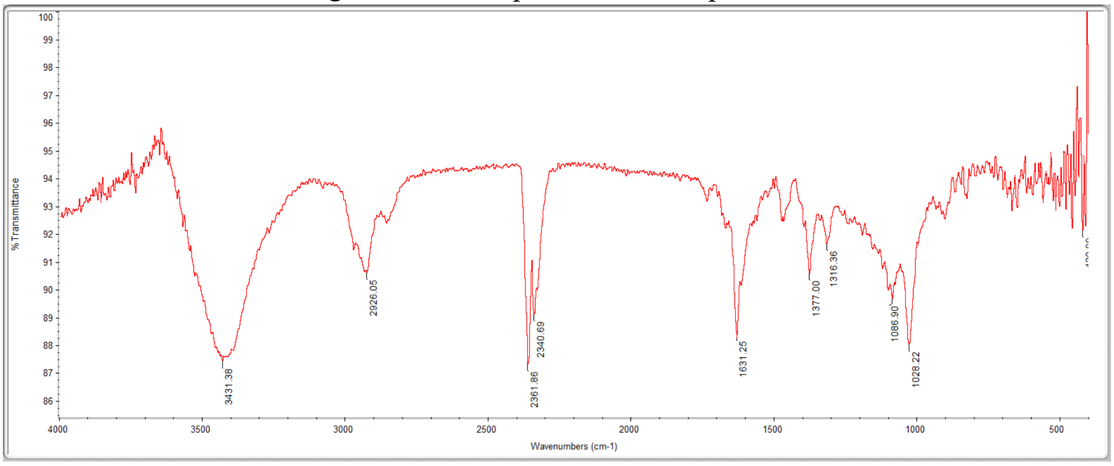

Figure S55. IR spectrum of compound **4**

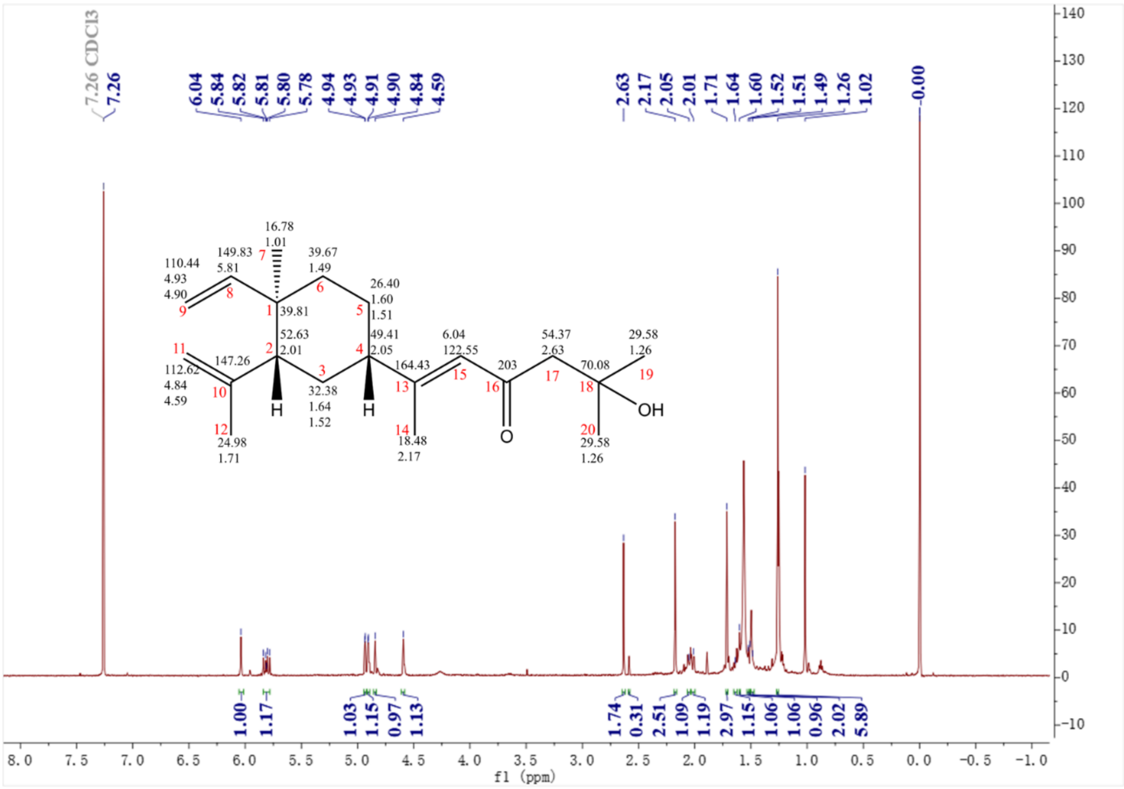

Figure S56. ^{1}H NMR spectrum (500 MHz, $CDCl_3$) of compound **4**

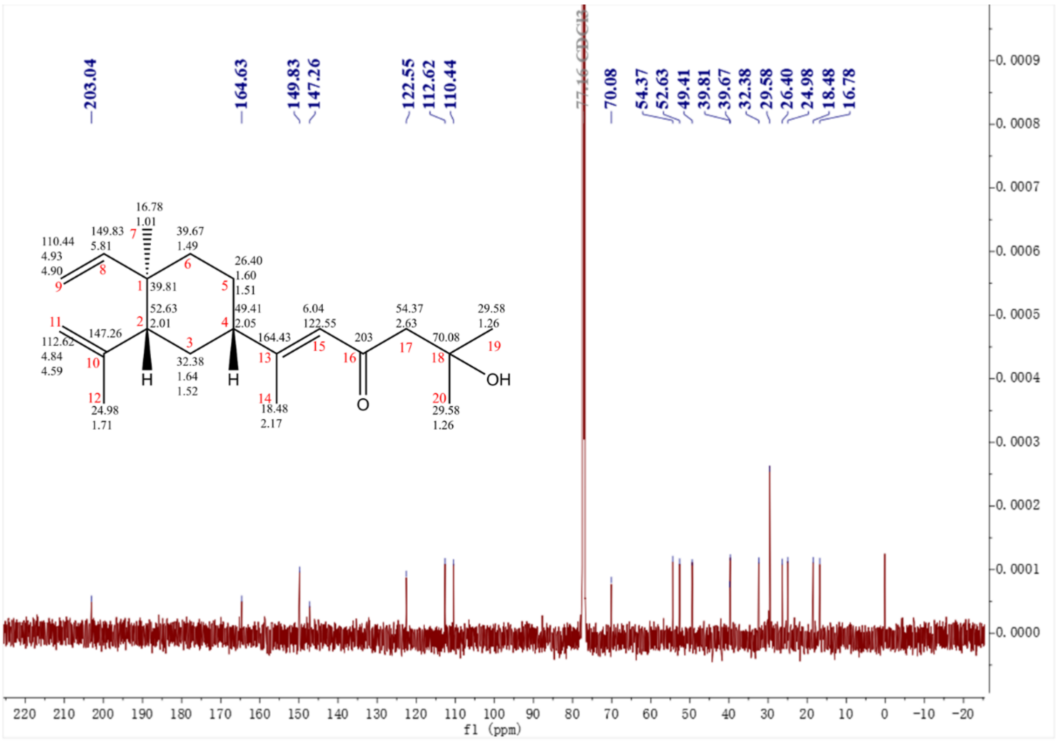

Figure S57. ^{13}C NMR spectrum (125 MHz, $CDCl_3$) of compound **4**

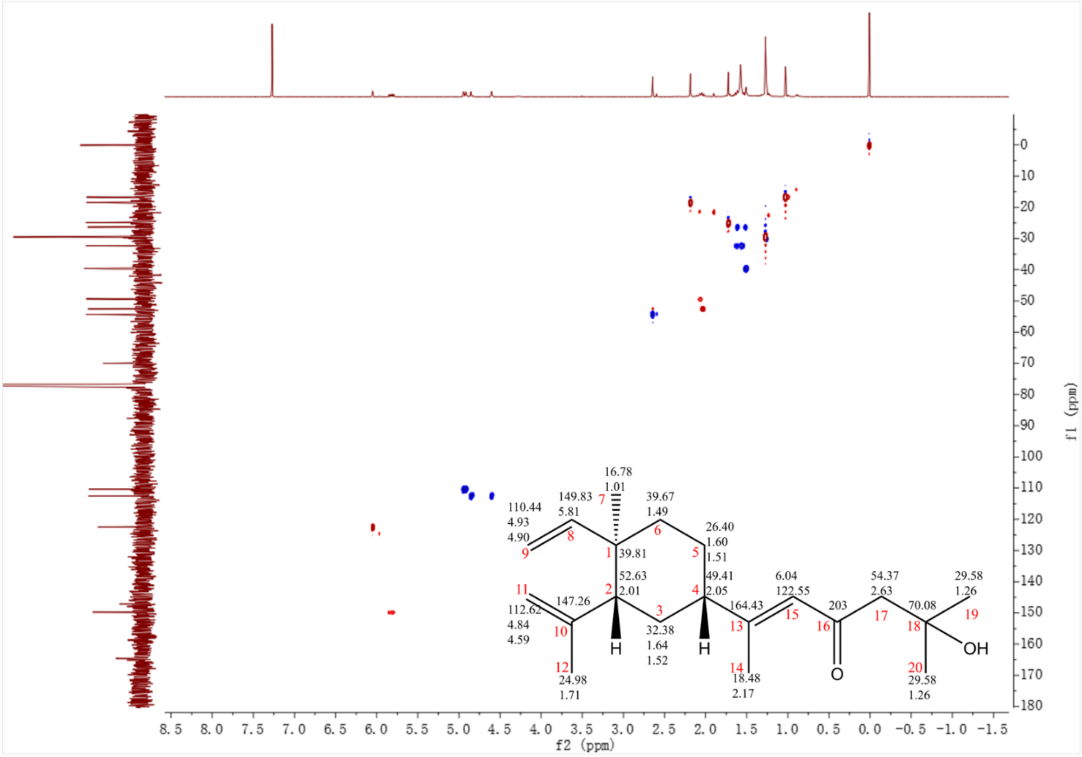

Figure S58. HSQC (500 MHz, $CDCl_3$) of compound **4**

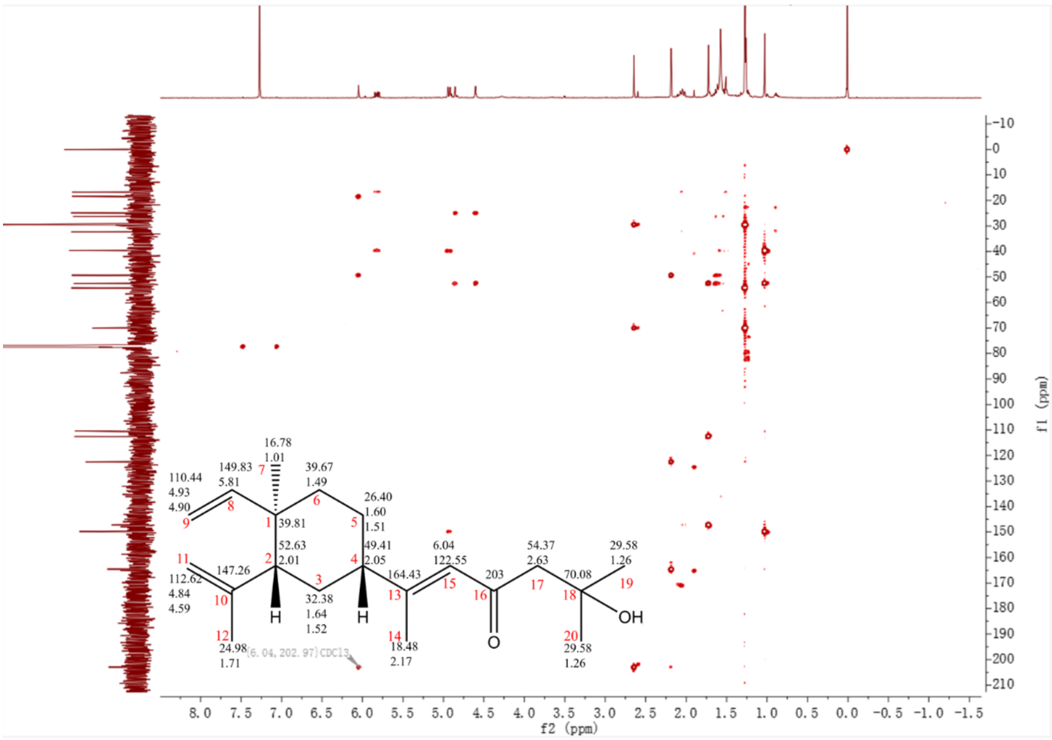

Figure S59. HMBC (125 MHz, $CDCl_3$) of compound **4**

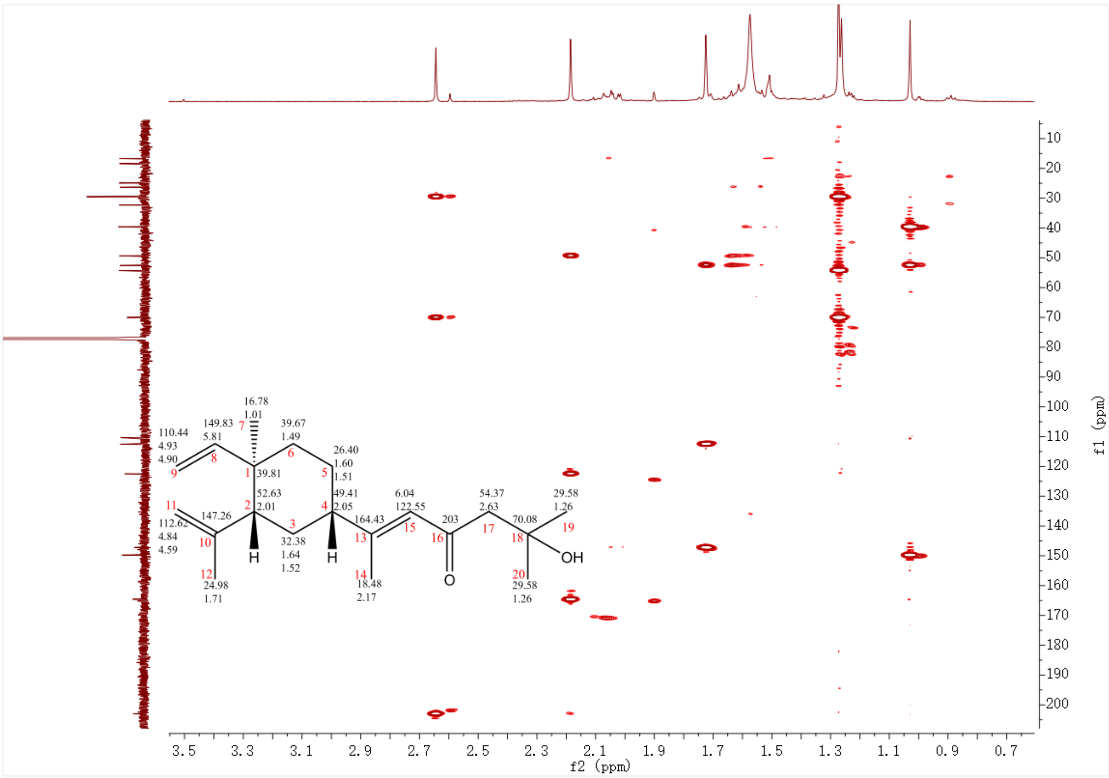

Figure S60. HMBC (125 MHz, $CDCl_3$) of compound **4**

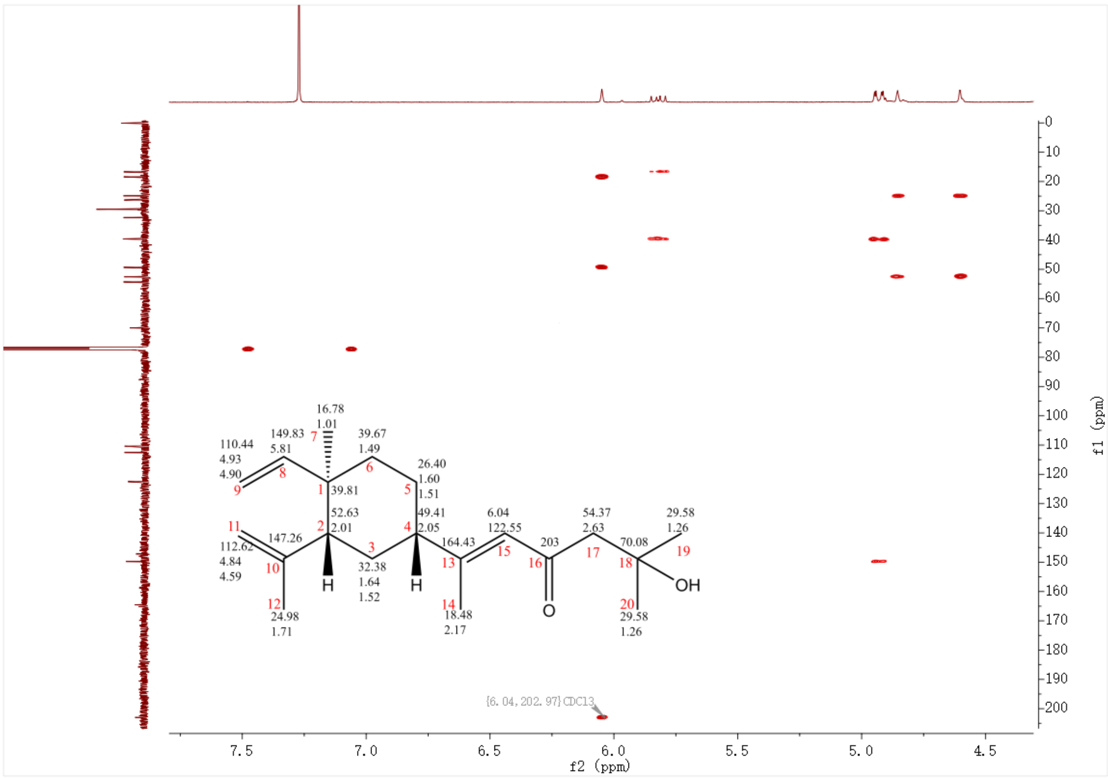

Figure S61. HMBC (125 MHz, $CDCl_3$) of compound **4**

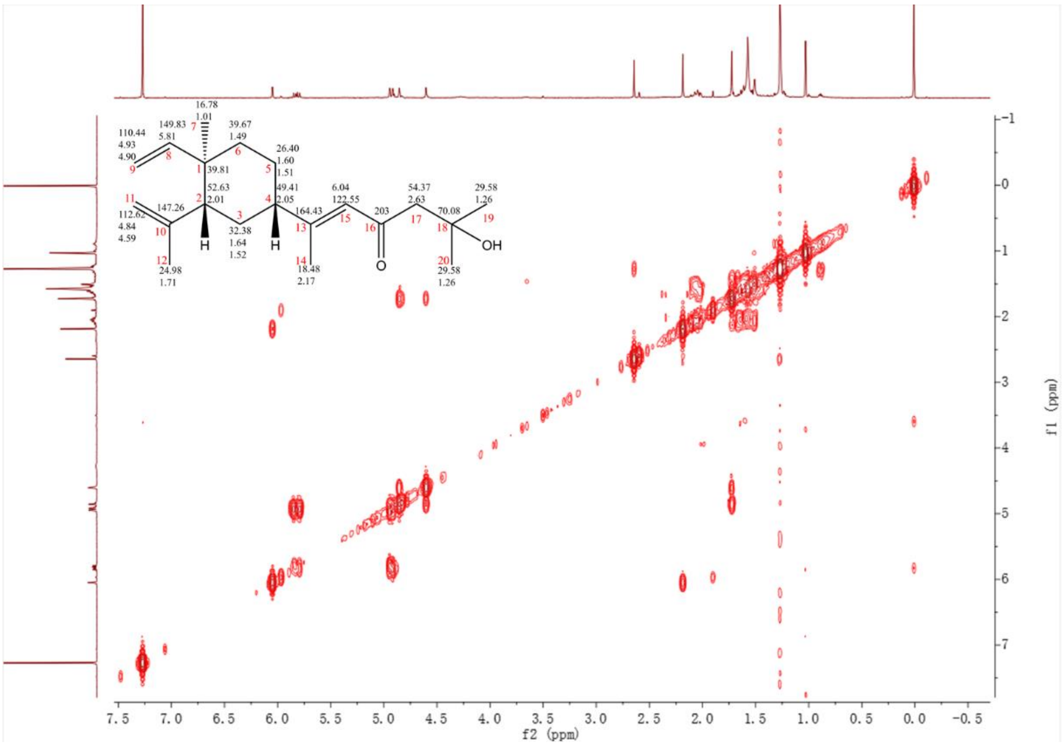

Figure S62. ^{1}H-^{1}H COSY (500 MHz, $CDCl_3$) of compound **4**

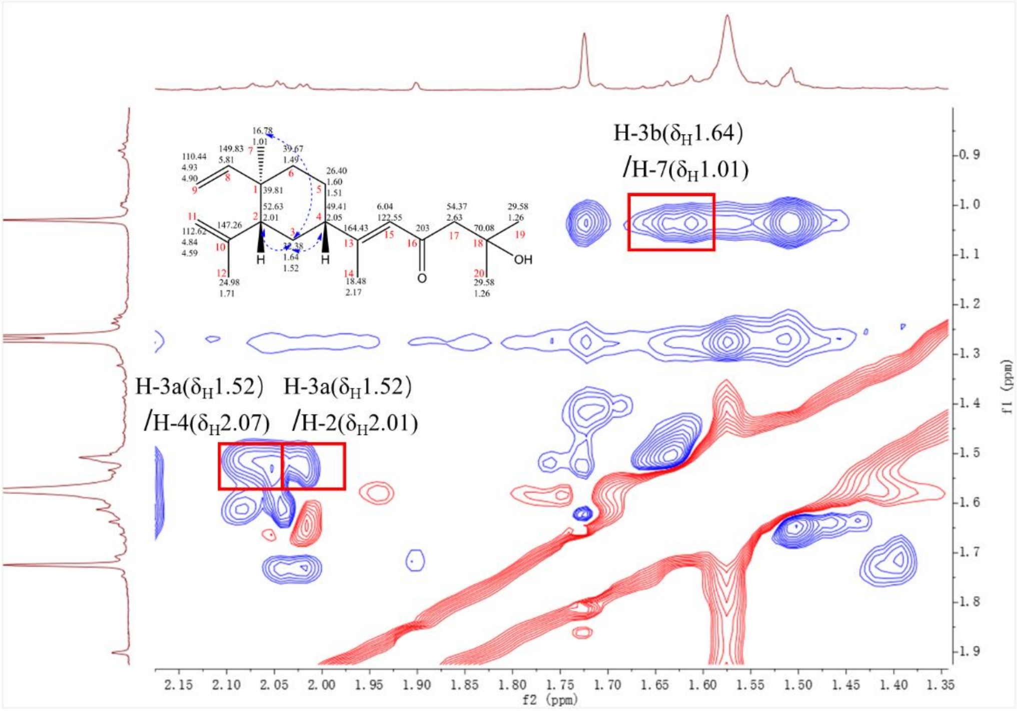

Figure S63. NOESY (500 MHz, $CDCl_3$) of compound **4**

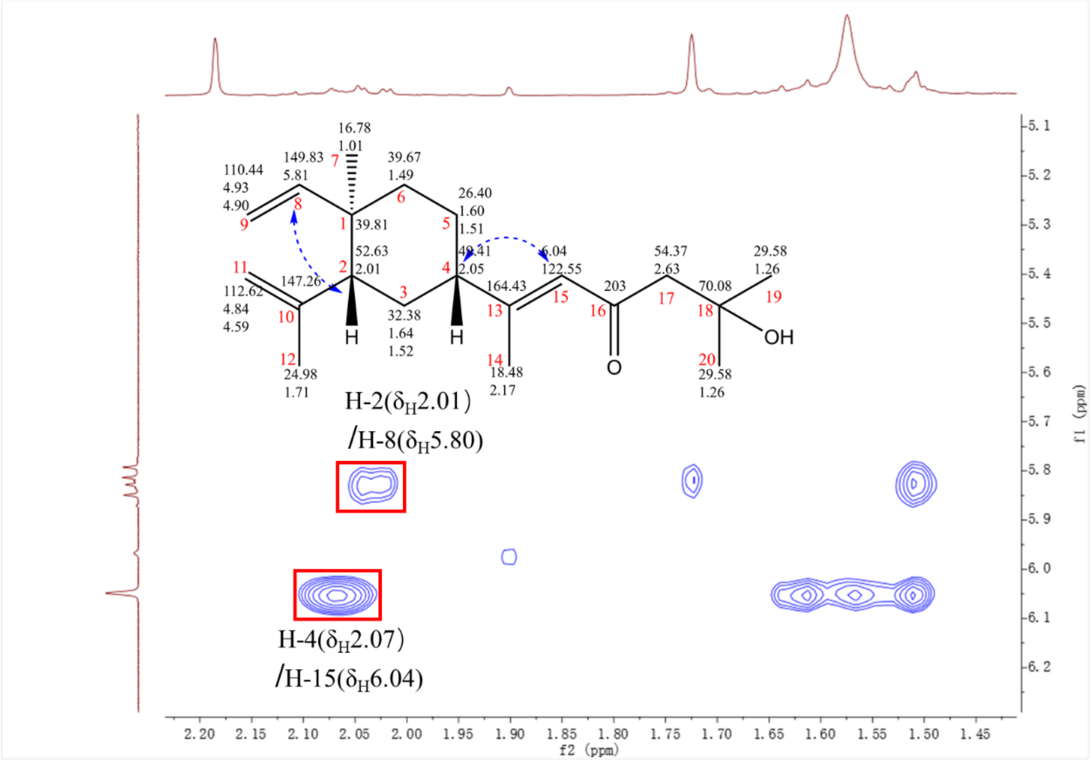

Figure S64. NOESY (500 MHz, CDCl3) of compound **4**

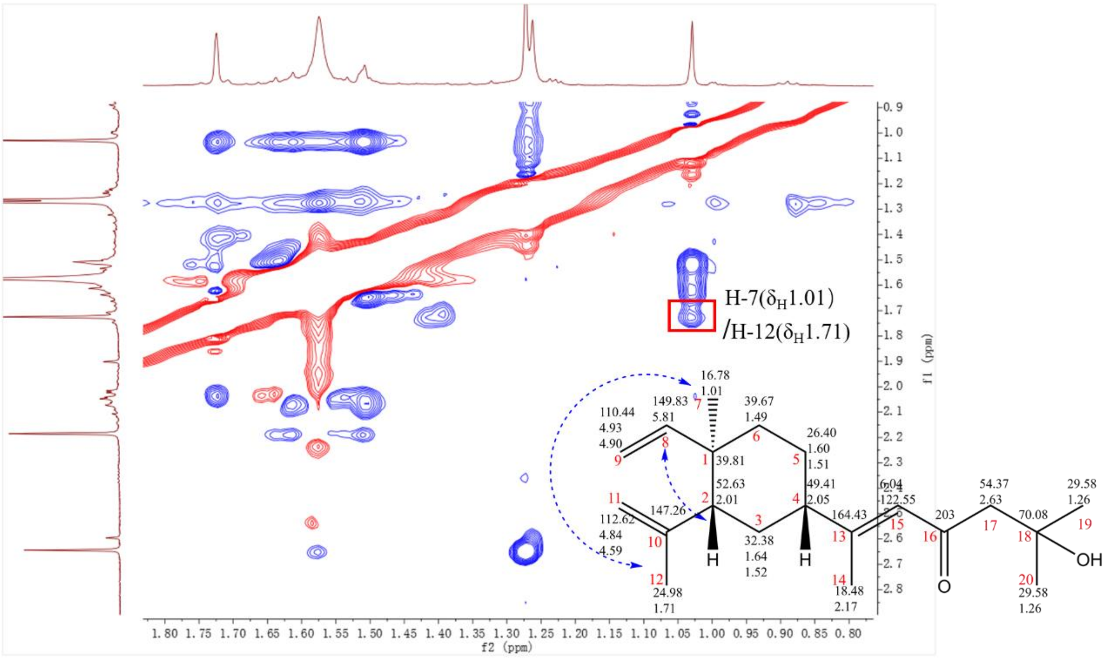

Figure S65. NOESY (500 MHz, CDCl3) of compound **4**

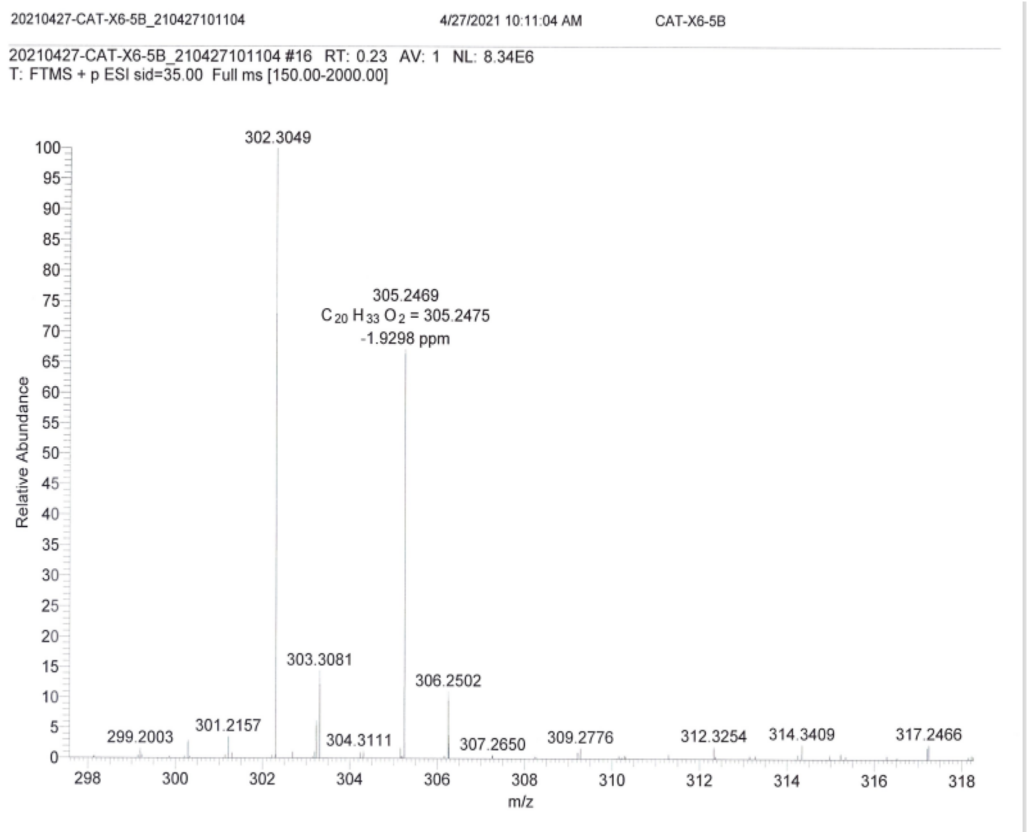

Figure S66. The positive HRESIMS spectrum of compound **5**

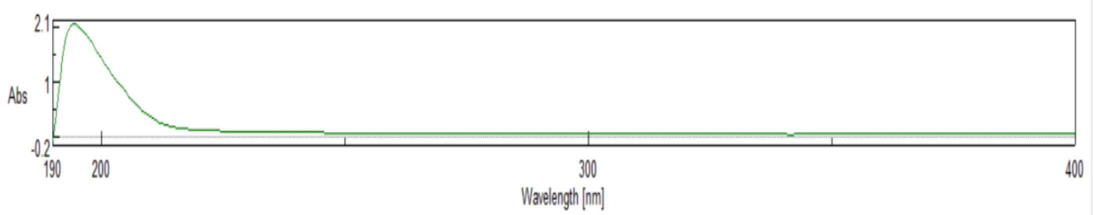

Figure 67. UV spectrum of compound **5**

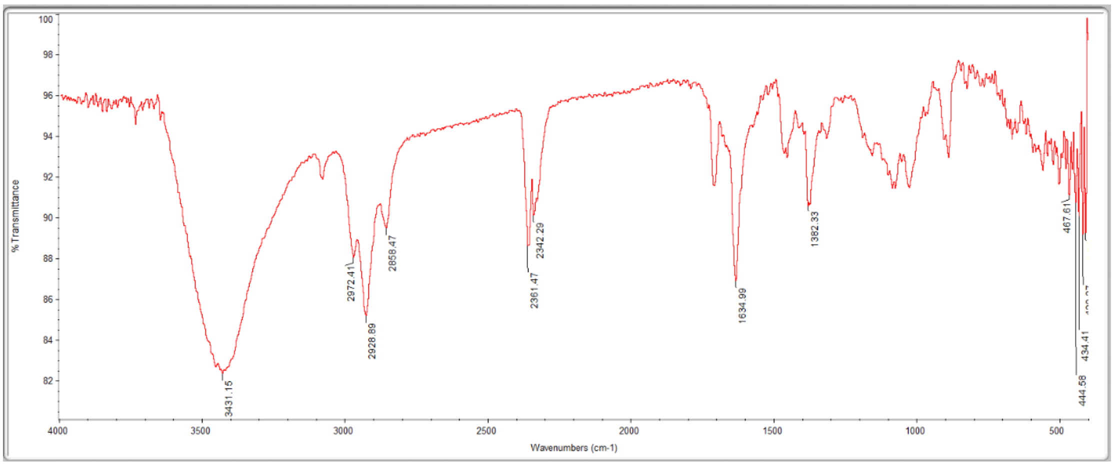

Figure S68. IR spectrum of compound **5**

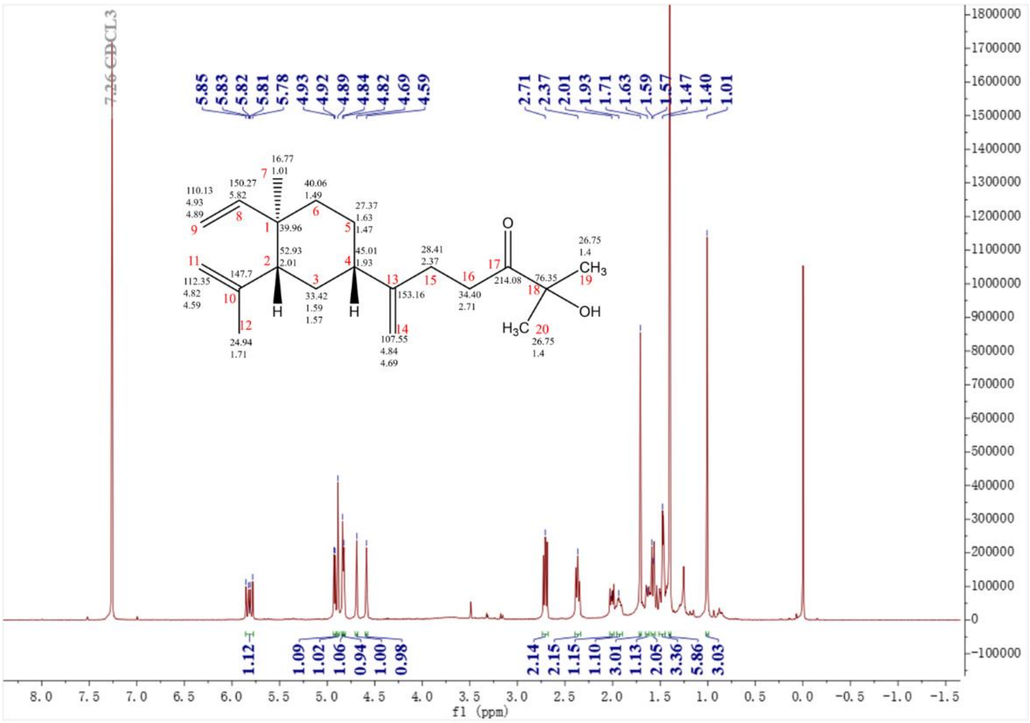

Figure S69. ^{1}H NMR spectrum (500 MHz, $CDCl_3$) of compound **5**

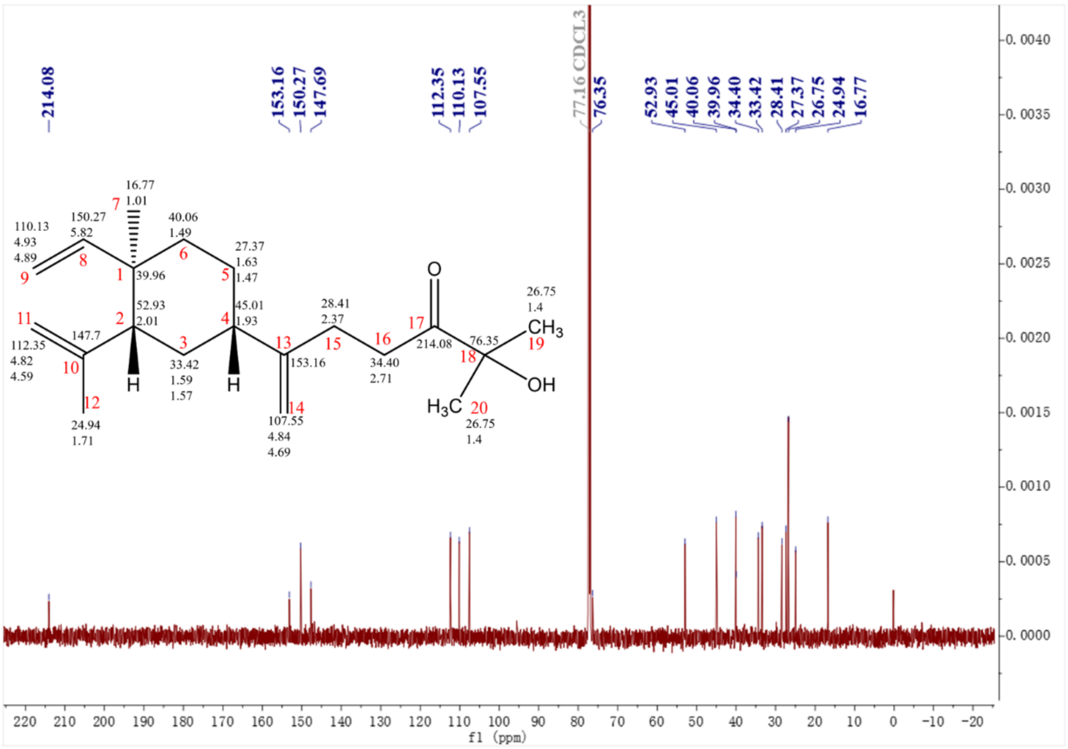

Figure S70. ^{13}C NMR spectrum (125 MHz, $CDCl_3$) of compound **5**

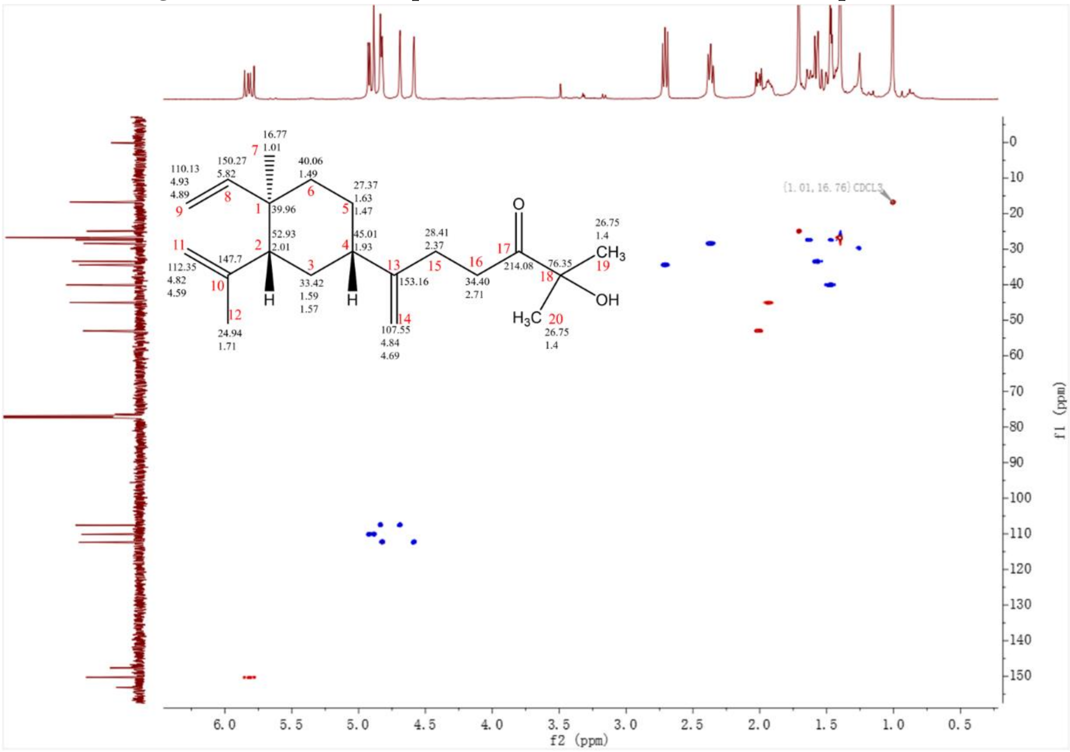

Figure S71. HSQC (500 M Hz, $CDCl_3$) of compound **5**

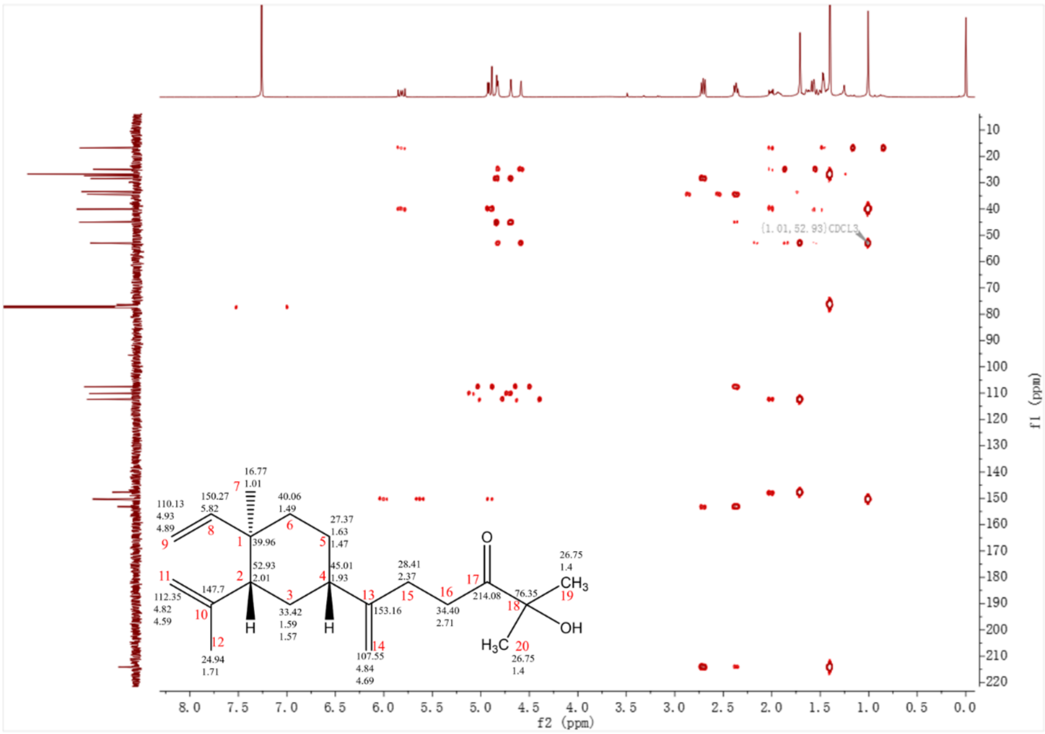

Figure S72. HMBC (125 MHz, $CDCl_3$) of compound **5**

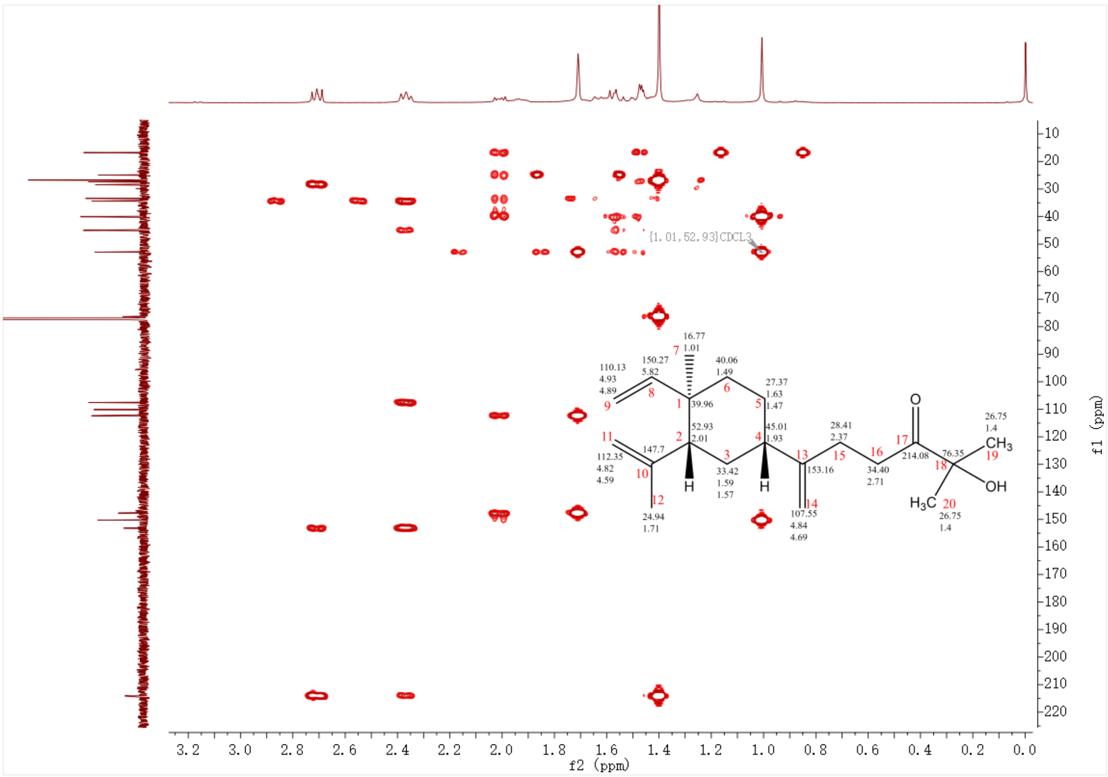

Figure S73. HMBC (125 MHz, $CDCl_3$) of compound **5**

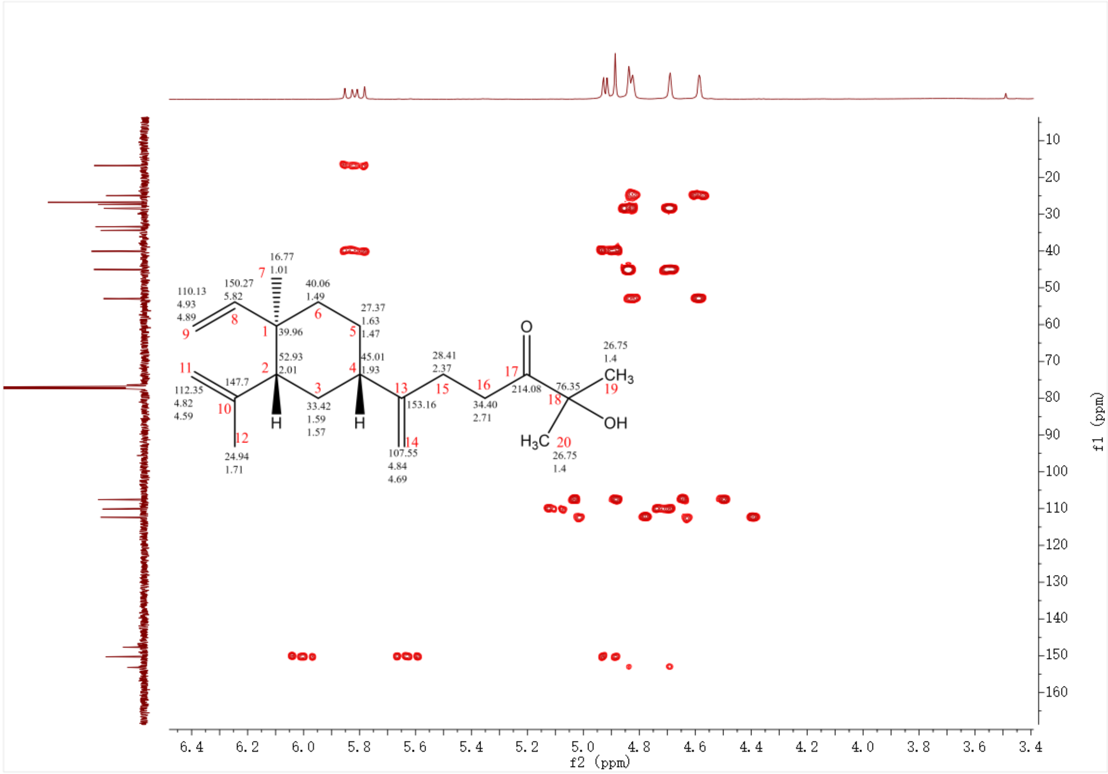

Figure S74. HMBC (125 MHz, $CDCl_3$) of compound **5**

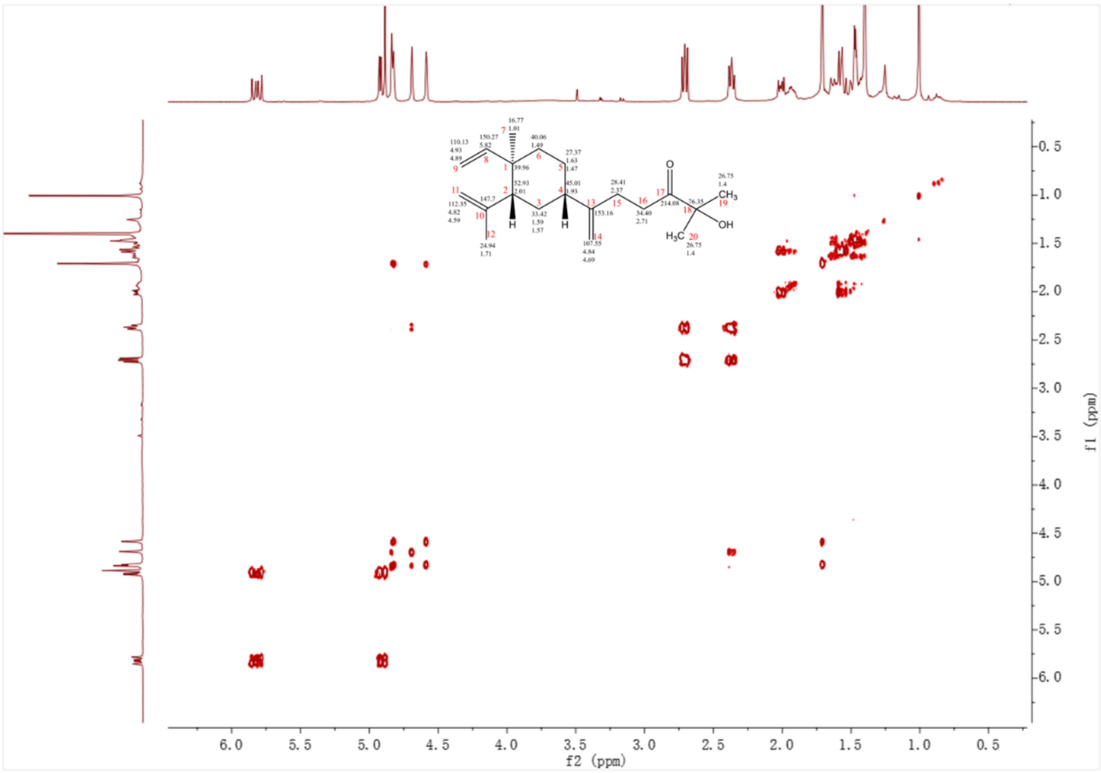

Figure S75. ^1H-^1H COSY (500 MHz, CDCl3) of compound **5**

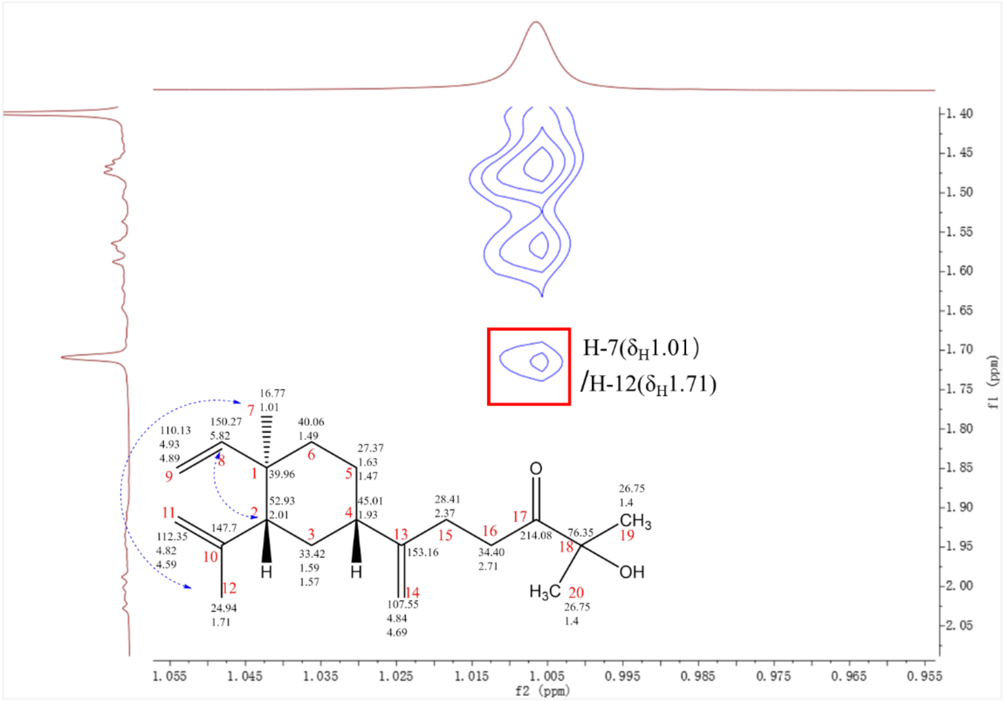

Figure S76. NOESY (500 MHz, CDCl3) of compound **5**

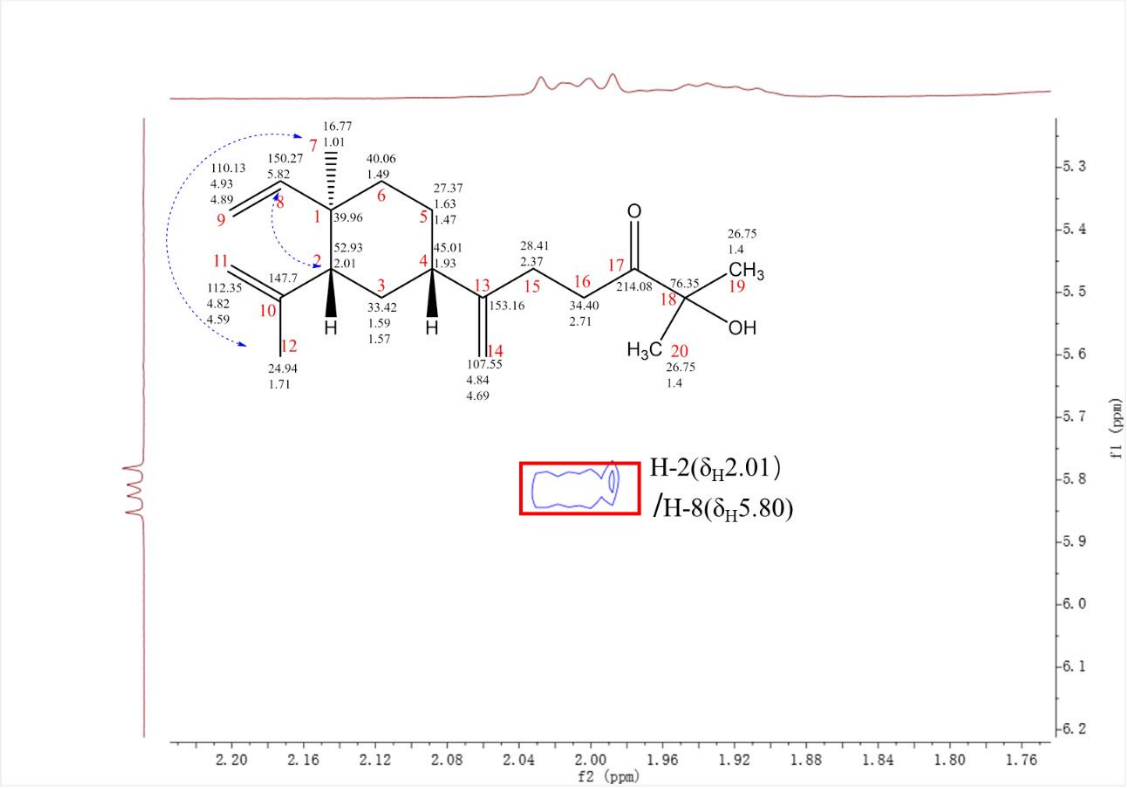

Figure S77. NOESY (500 MHz, CDCl3) of compound **5**

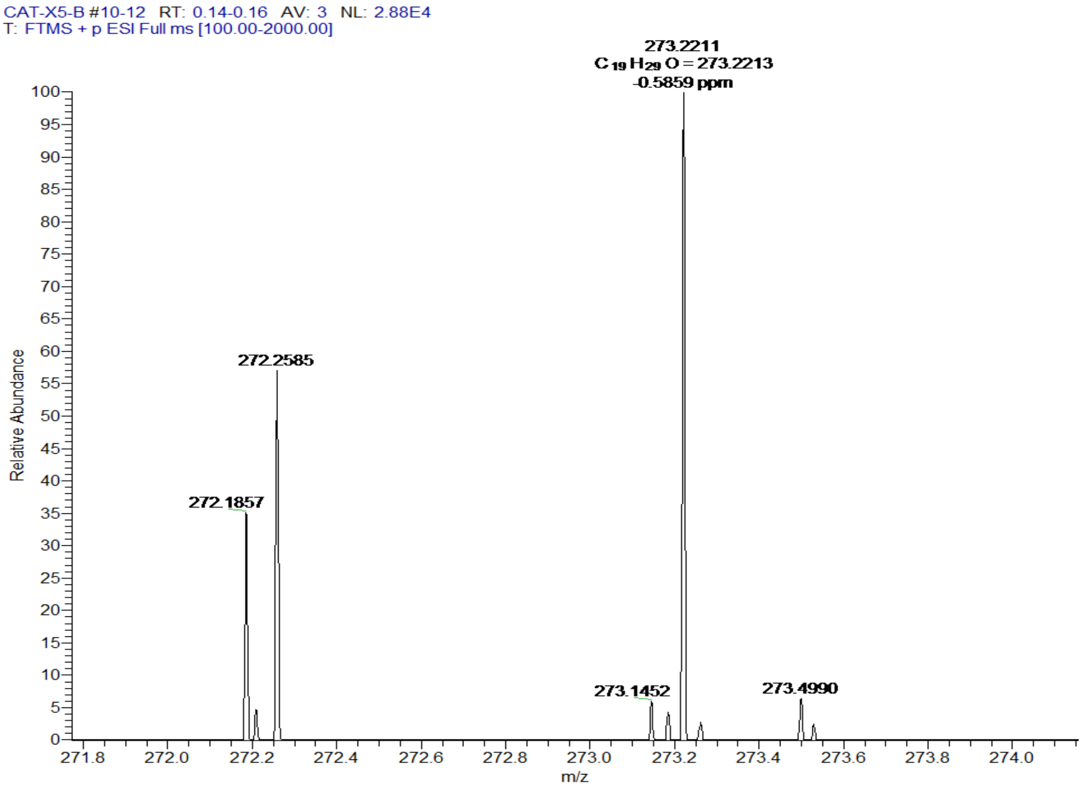

Figure S78. The positive HRESIMS spectrum of compound **6**

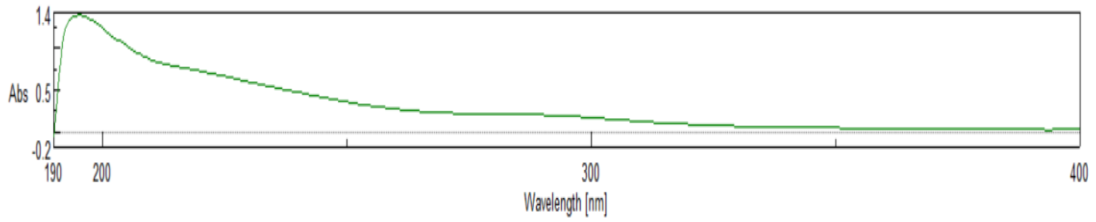

Figure 79. UV spectrum of compound **6**

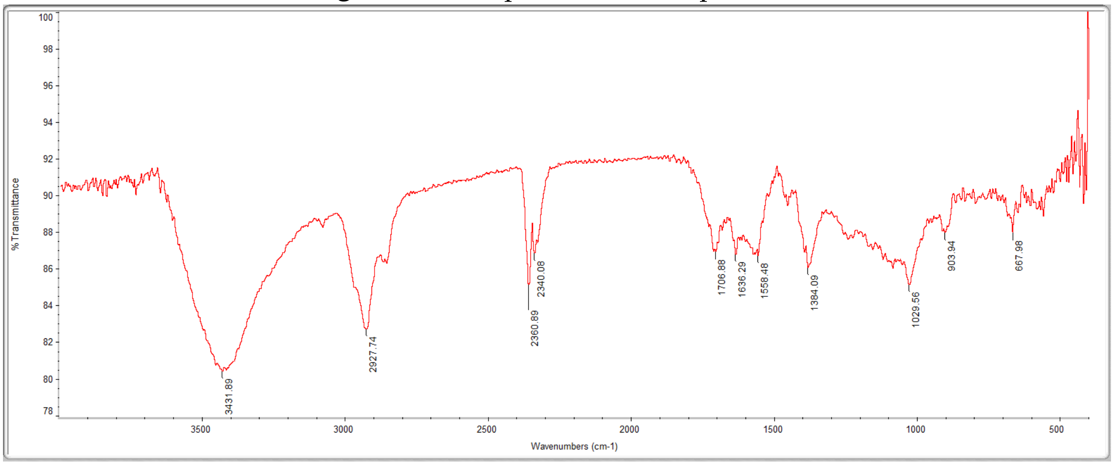

Figure S80. IR spectrum of compound **6**

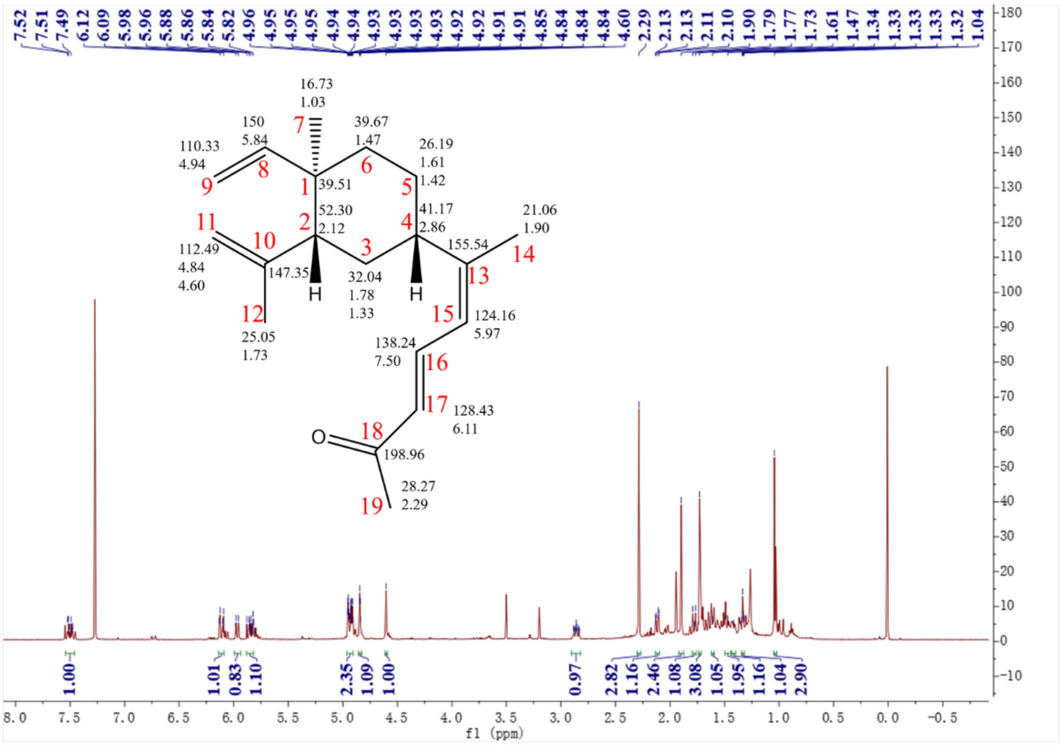

Figure S81. ^{1}H NMR spectrum (500 MHz, $CDCl_3$) of compound **6**

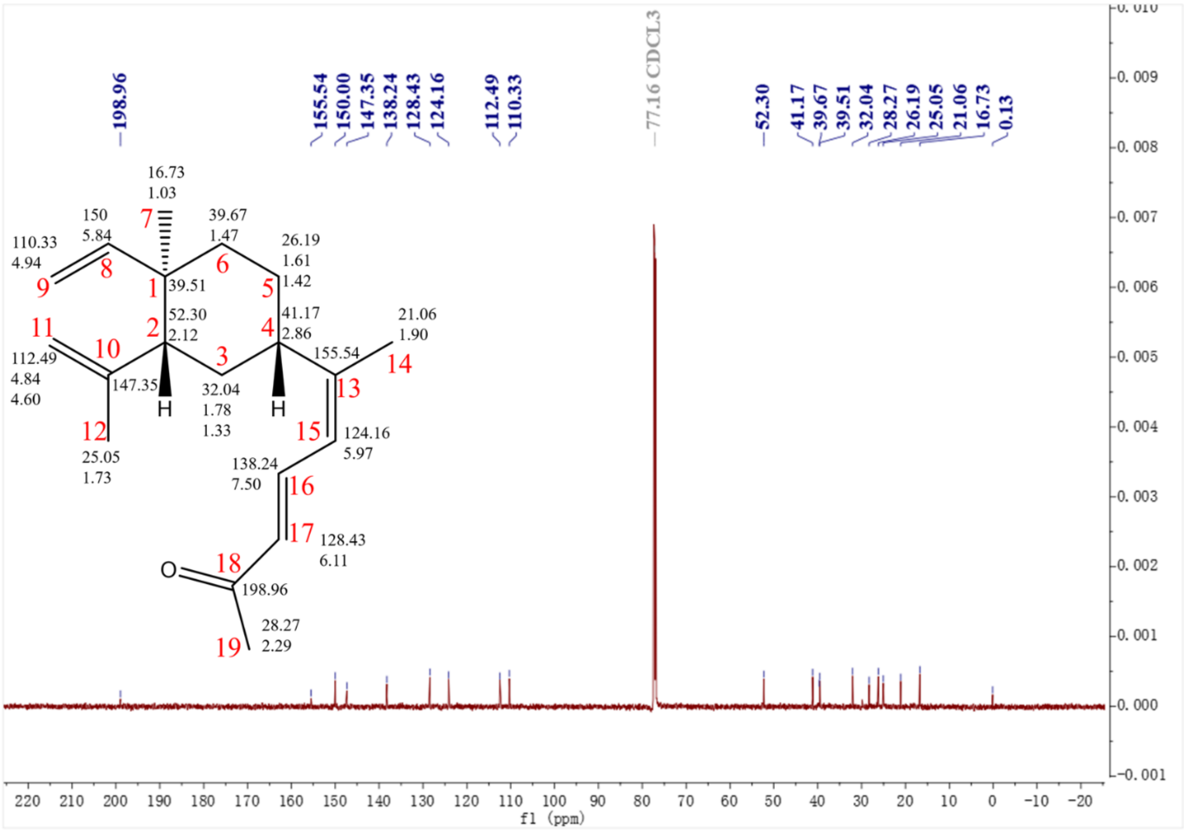

Figure S82. ^{13}C NMR spectrum (125 MHz, $CDCl_3$) of compound **6**

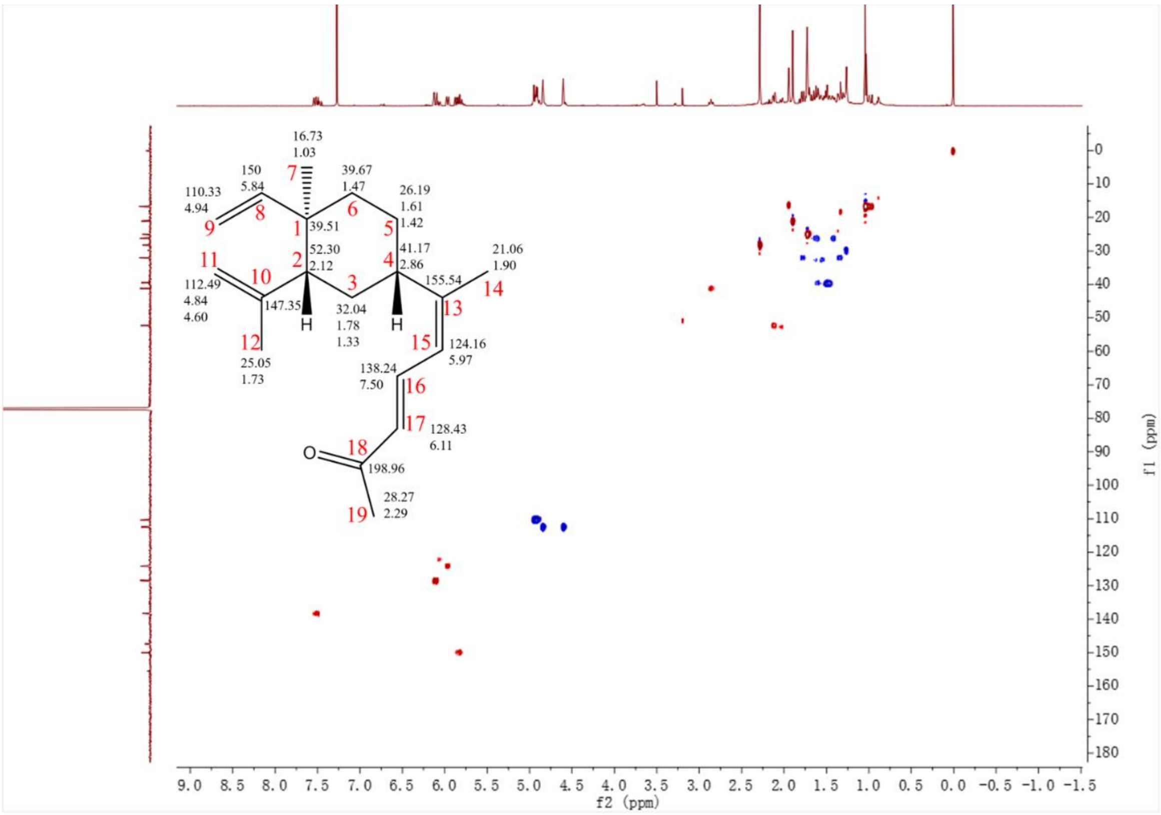

Figure S83. HSQC (500 M Hz, CDCl3) of compound **6**

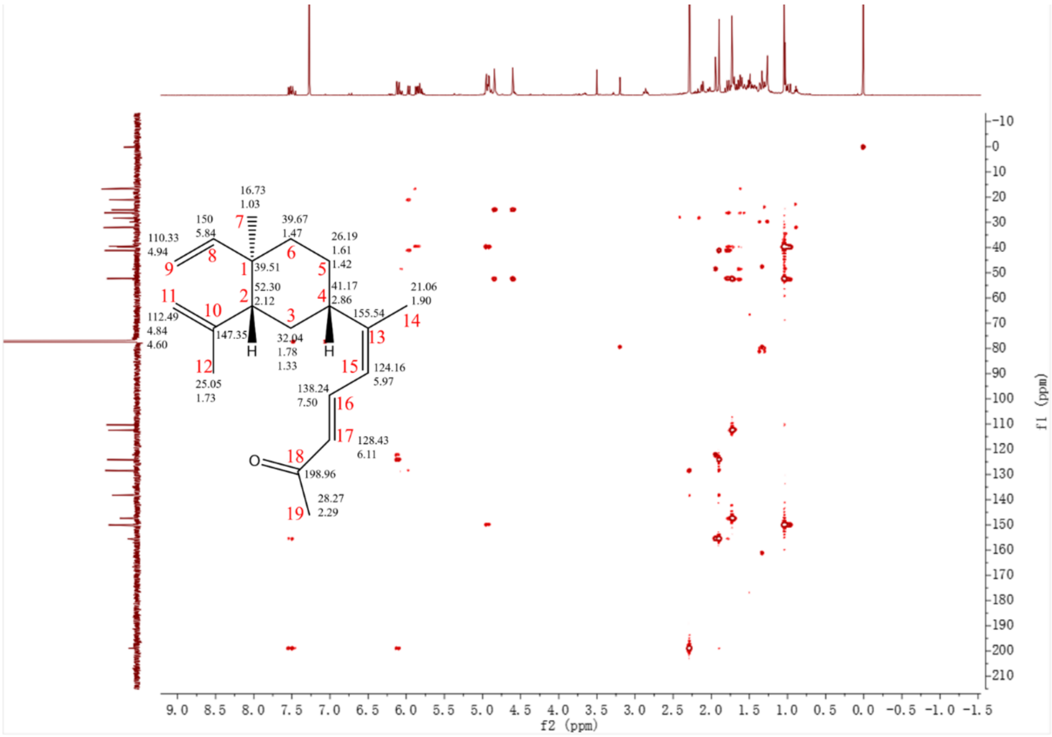

Figure S84. HMBC (125 MHz, CDCl3) of compound **6**

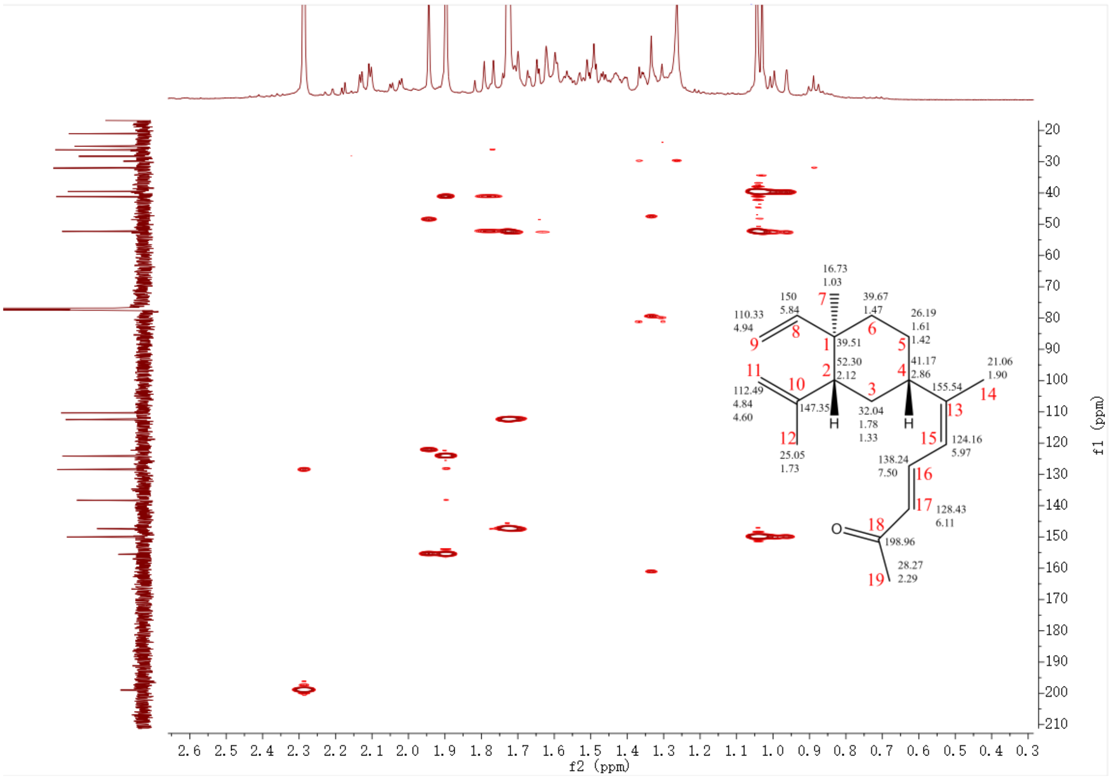

Figure S85. HMBC (125 MHz, CDCl3) of compound **6**

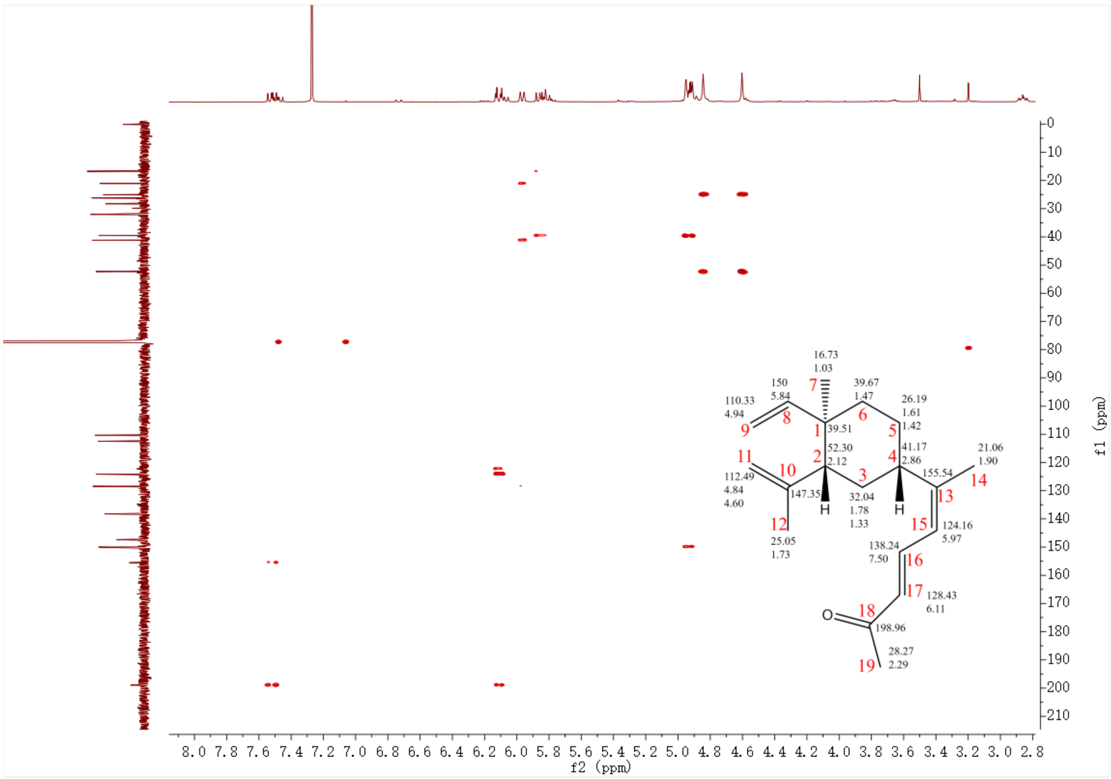

Figure S86. HMBC (125 MHz, CDCl3) of compound **6**

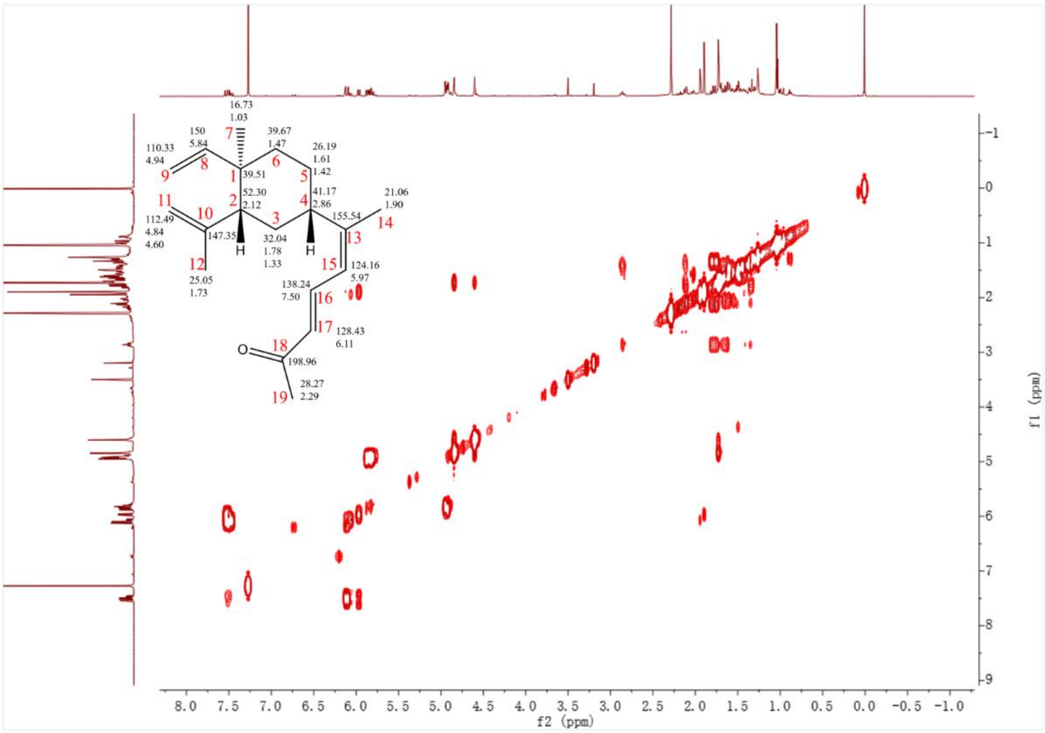

Figure S87. ^1H-^1H COSY (500 MHz, CDCl3) of compound **6**

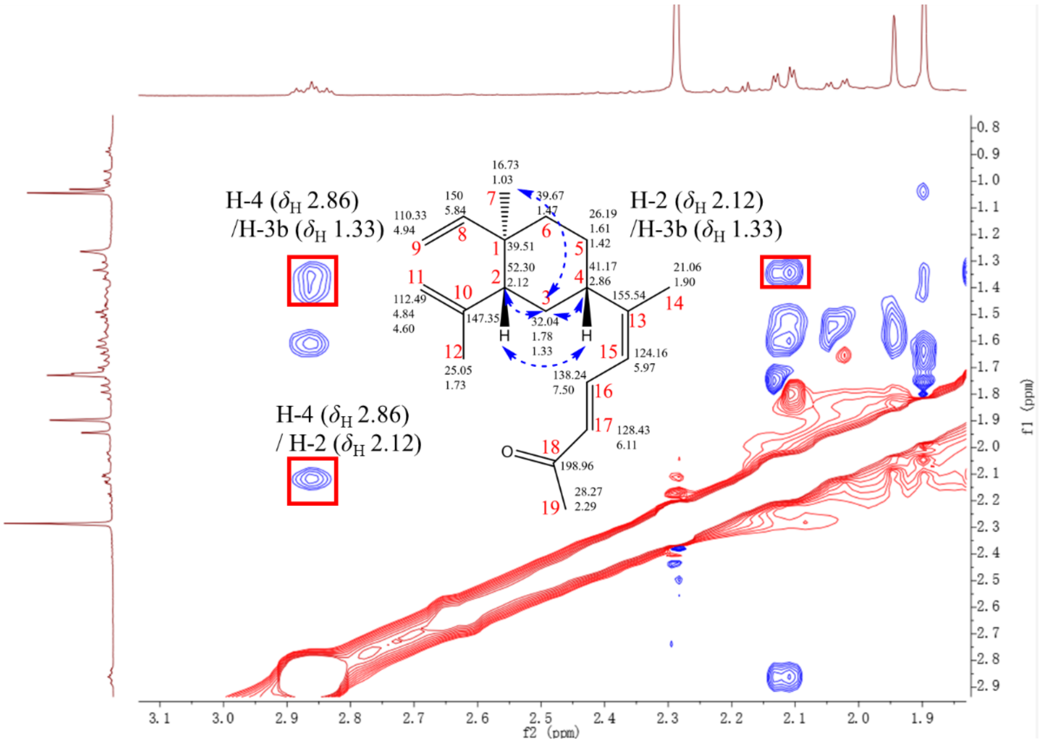

Figure S88. NOESY (500 MHz, $CDCl_3$) of compound **6**

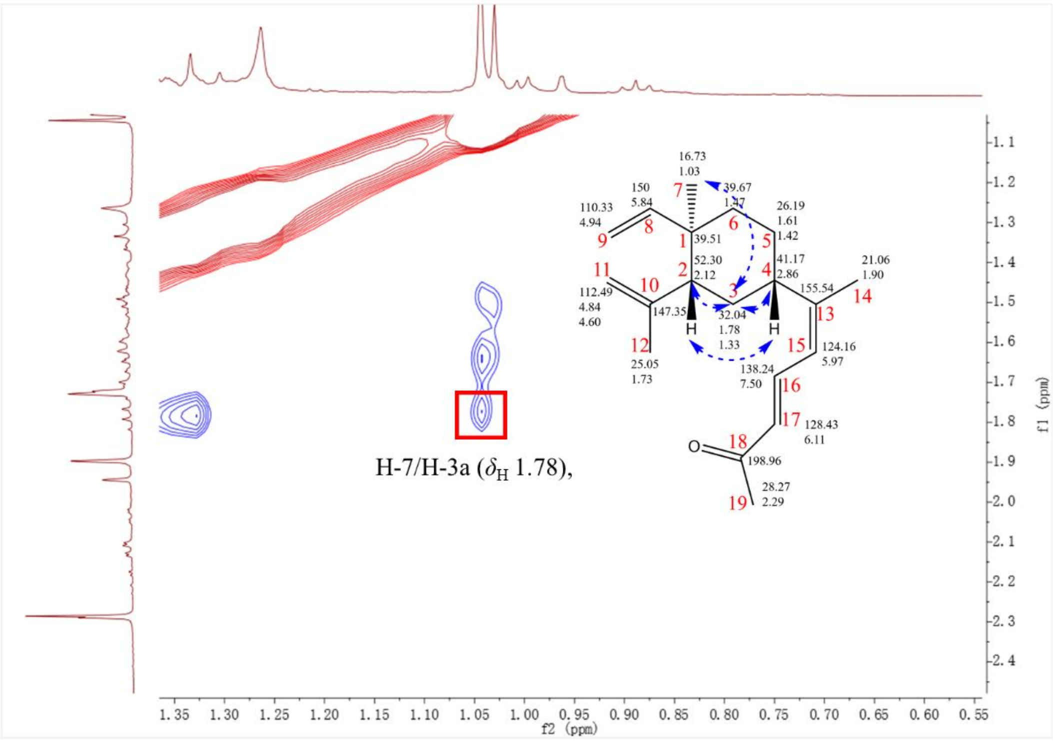

Figure S89. NOESY (500 MHz, $CDCl_3$) of compound **6**

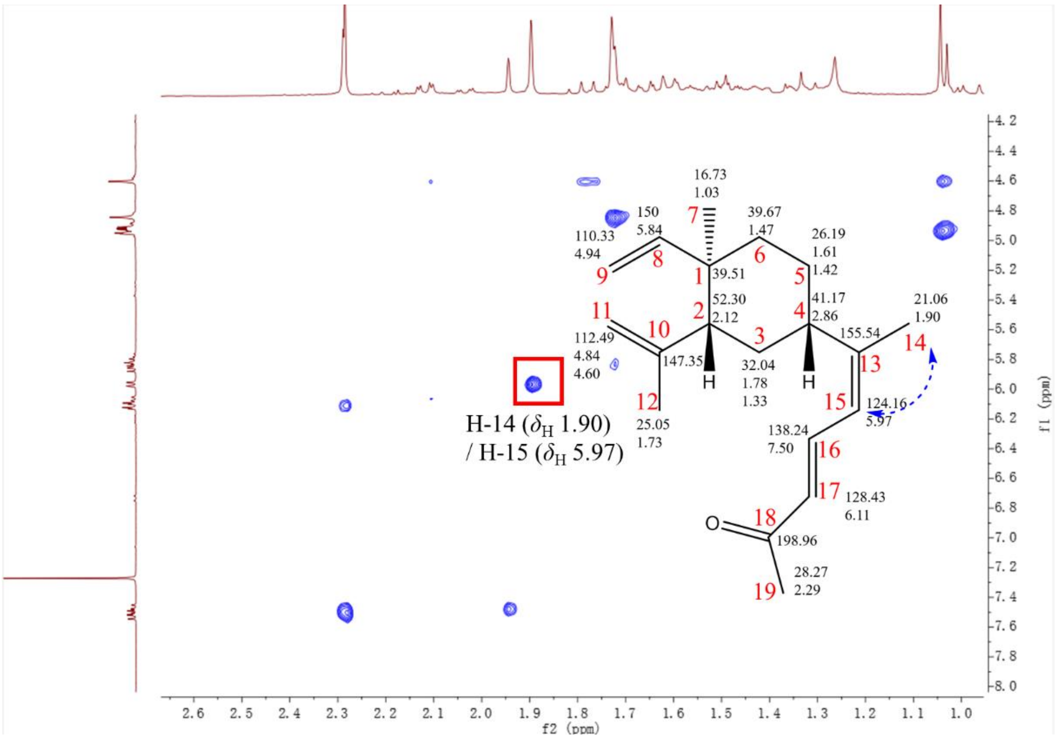

Figure S90. NOESY (500 MHz, $CDCl_3$) of compound **6**

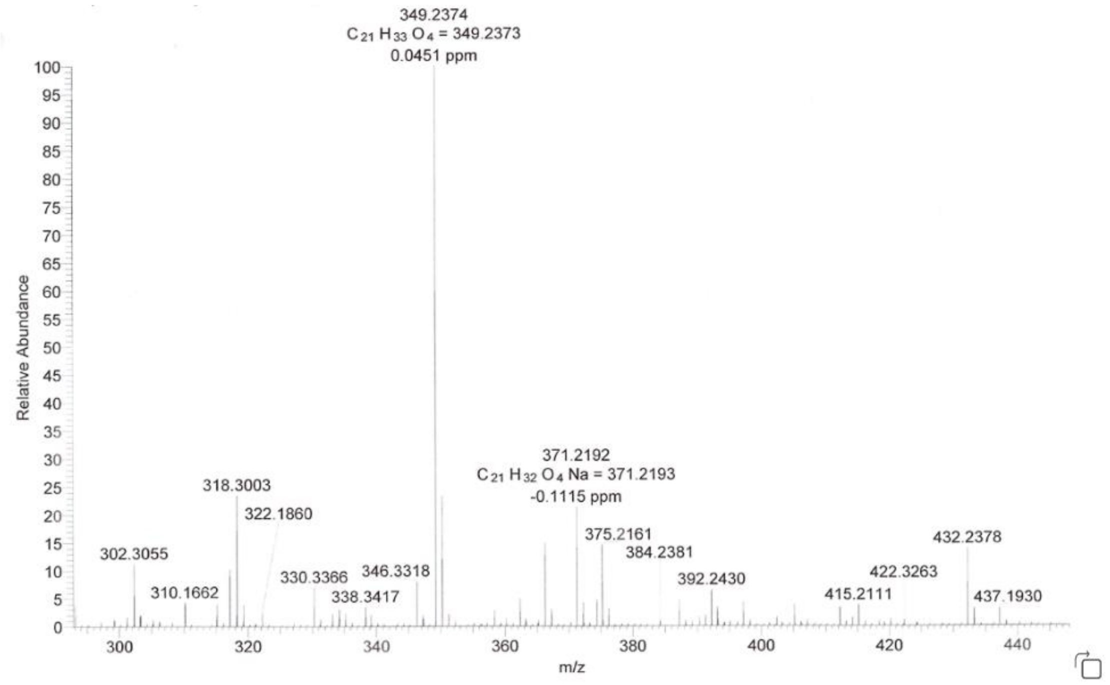

Figure S91. The positive HRESIMS spectrum of compound **7**

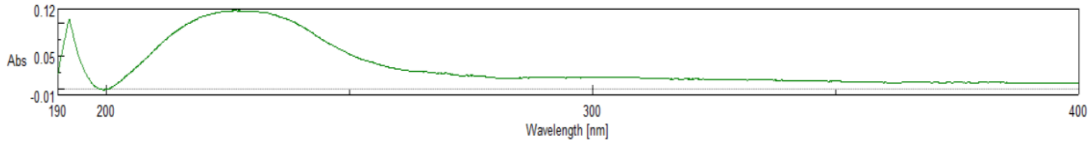

Figure 92. UV spectrum of compound **7**

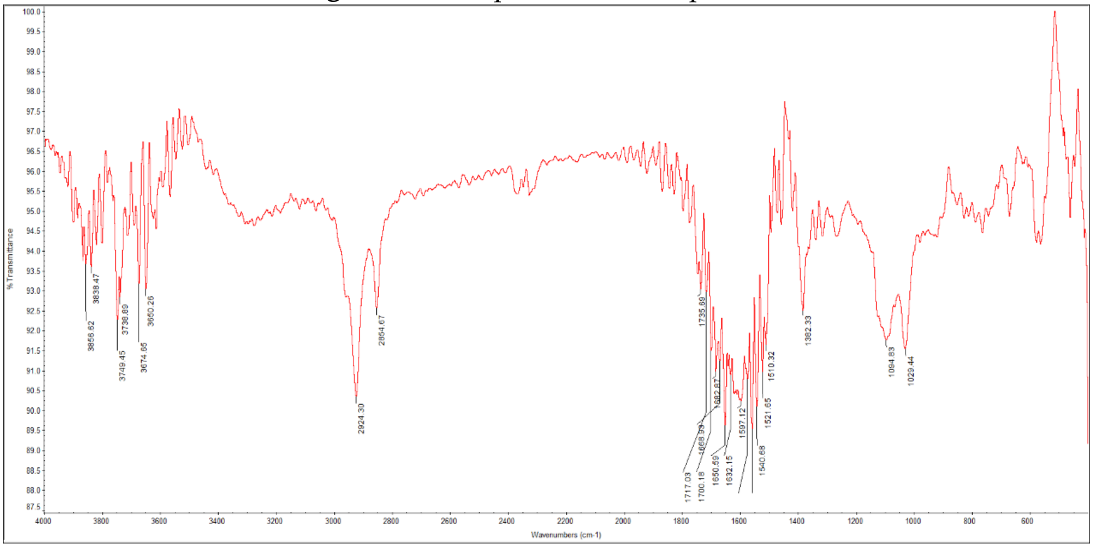

Figure S93. IR spectrum of compound **7**

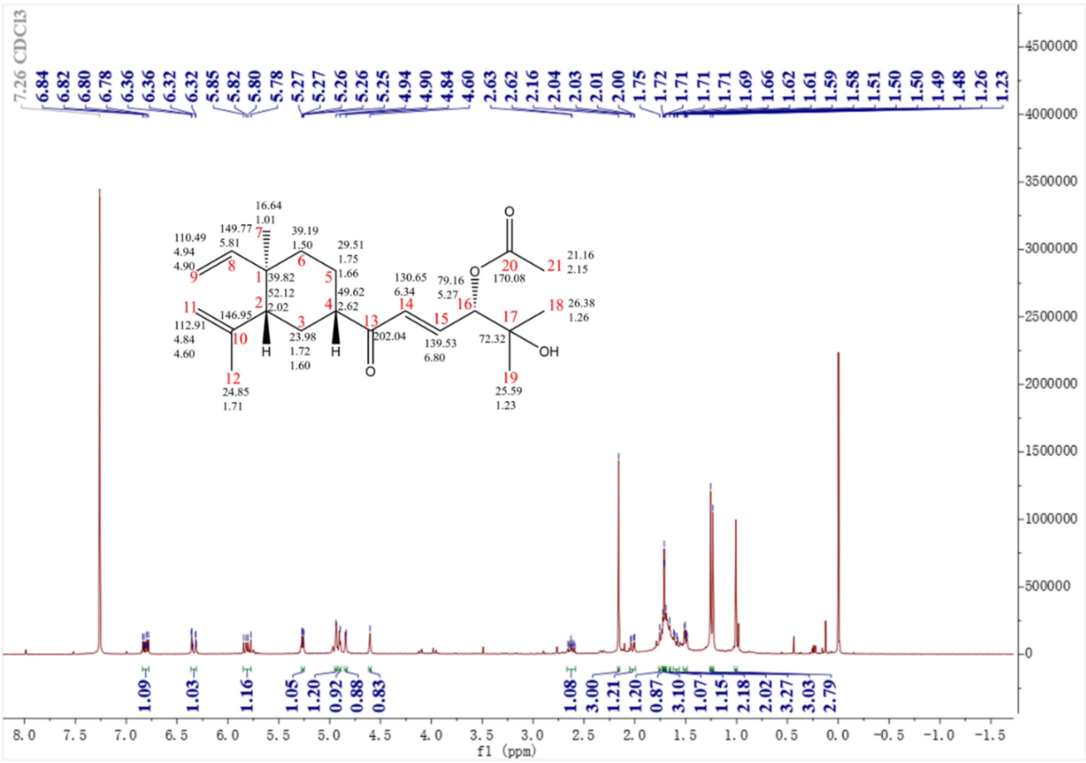

Figure S94. ^{1}H NMR spectrum (600 MHz, $CDCl_3$) of compound **7**

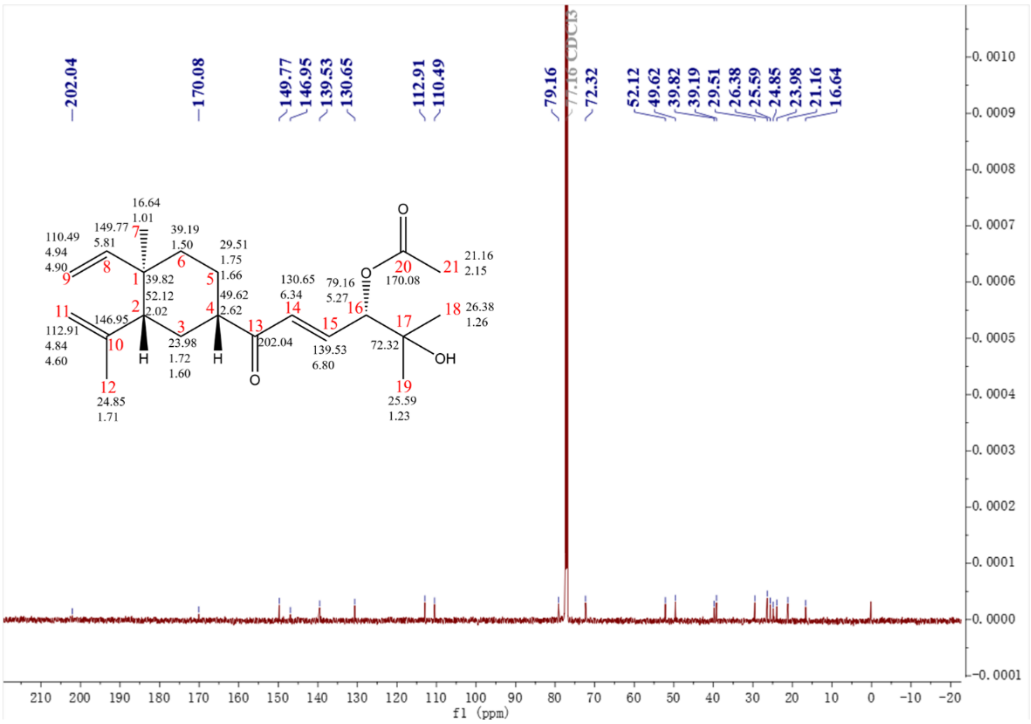

Figure S95. ^{13}C NMR spectrum (150 MHz, $CDCl_3$) of compound **7**

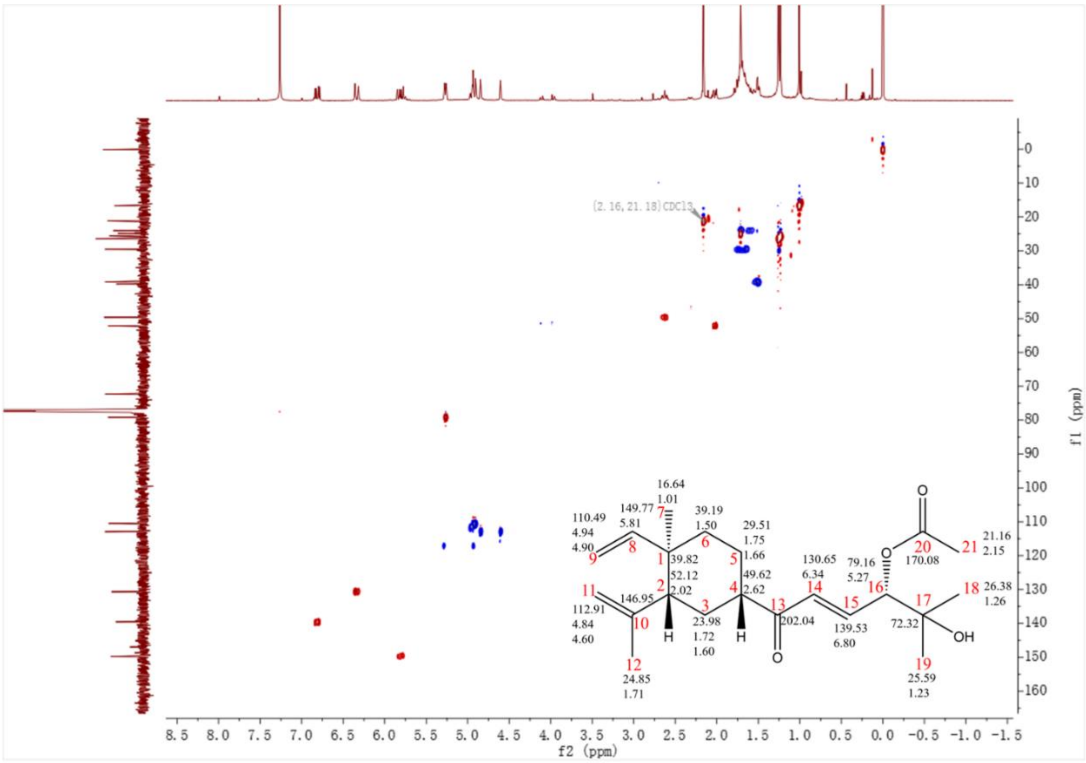

Figure S96. HSQC (600 M Hz, $CDCl_3$) of compound **7**

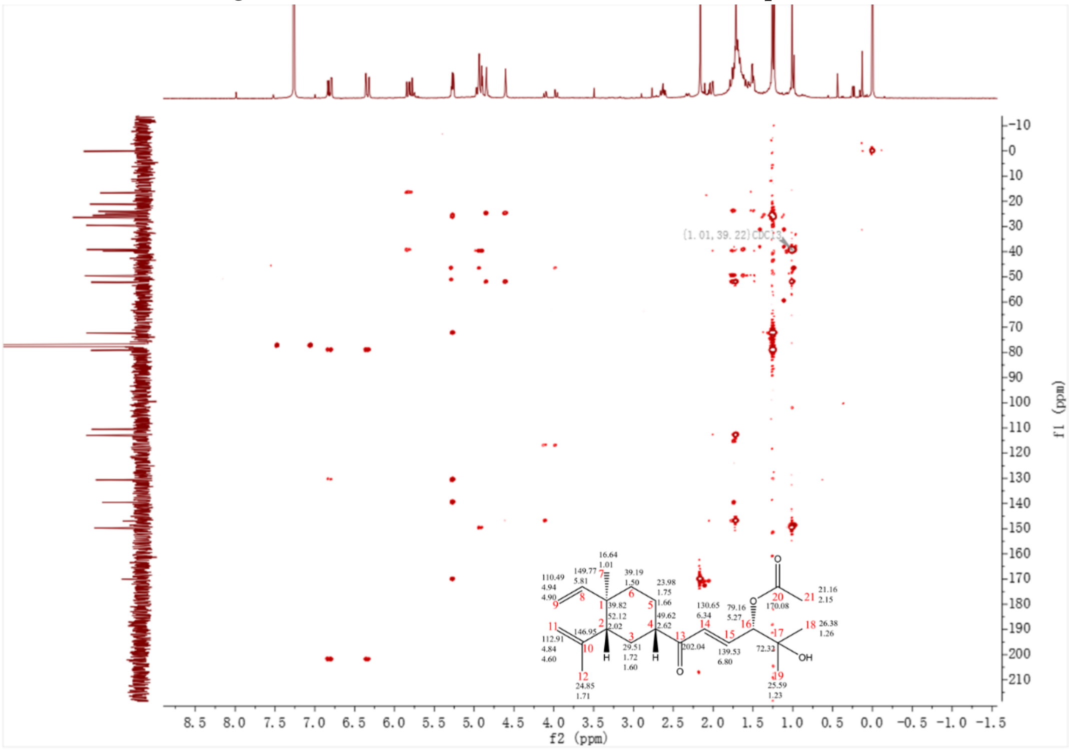

Figure S97. HMBC (150 MHz, $CDCl_3$) of compound **7**

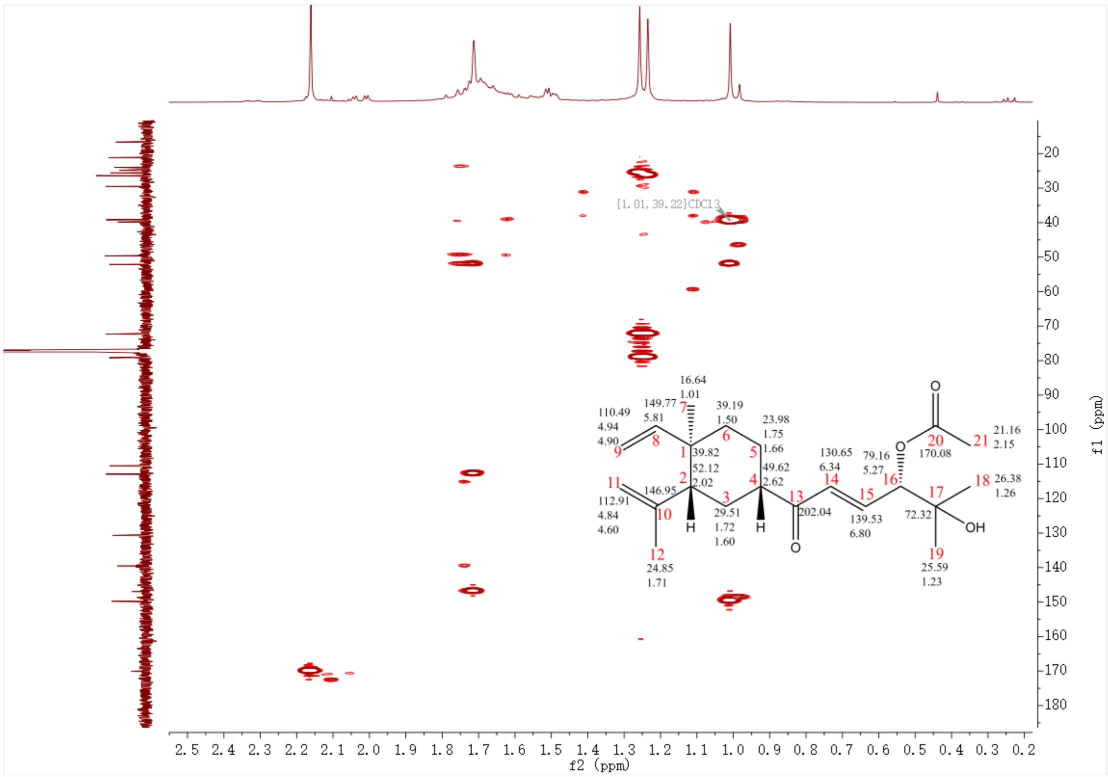

Figure S98. HMBC (150 MHz, CDCl3) of compound **7**

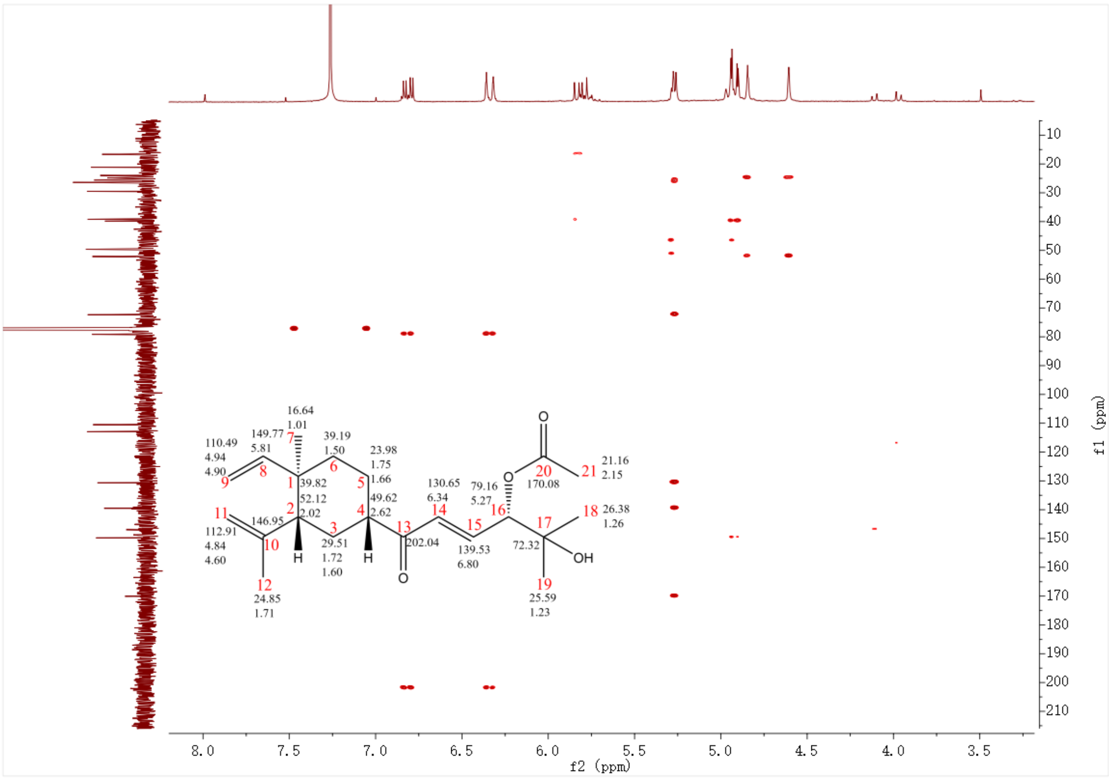

Figure S99. HMBC (150 MHz, CDCl3) of compound **7**

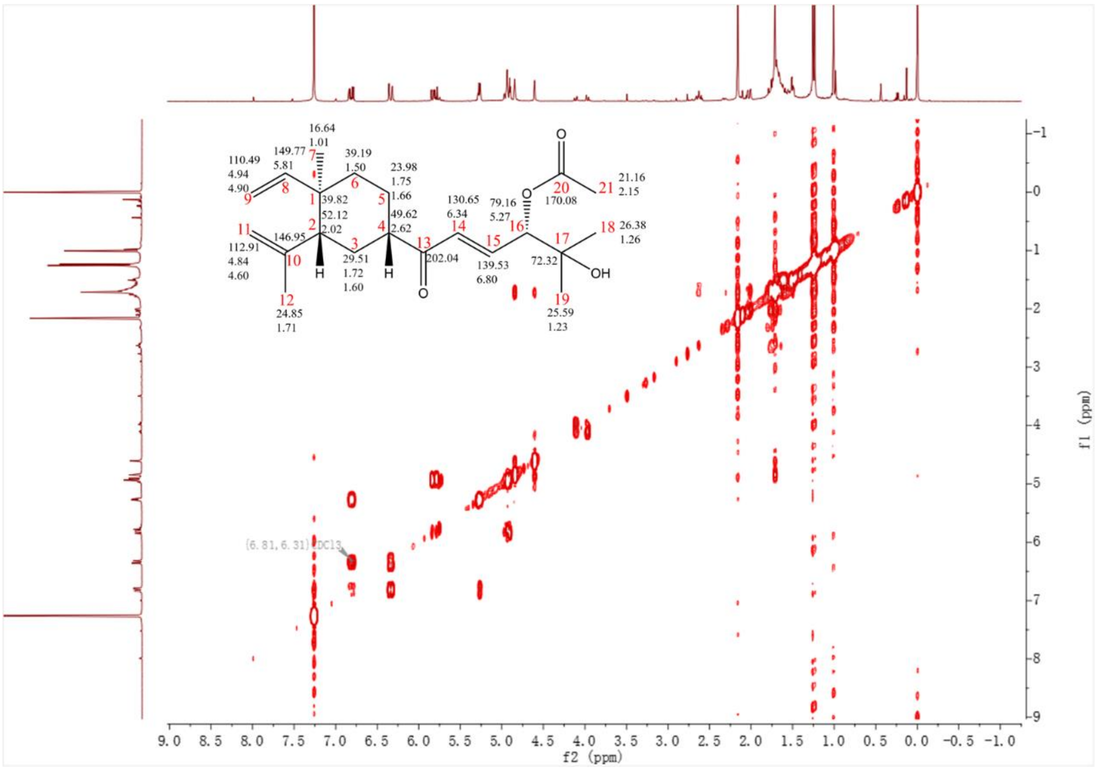

Figure S100. ^{1}H-^{1}H COSY (600 MHz, CDCl3) of compound **7**

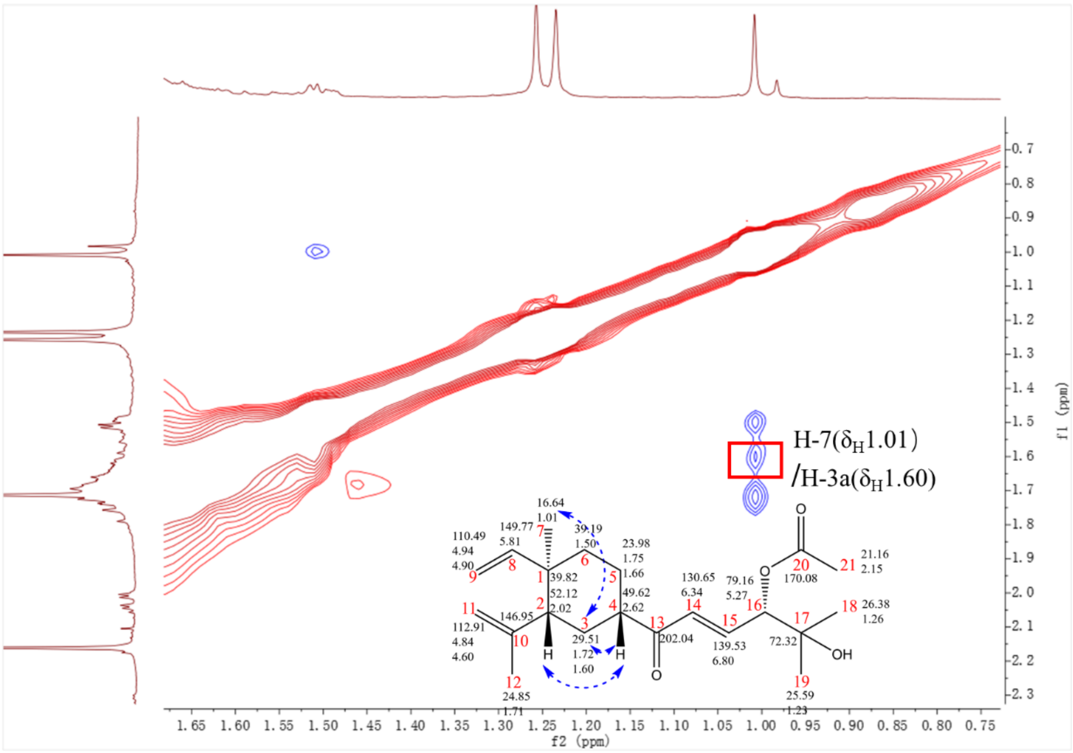

Figure S101. NOESY (600 MHz, $CDCl_3$) of compound **7**

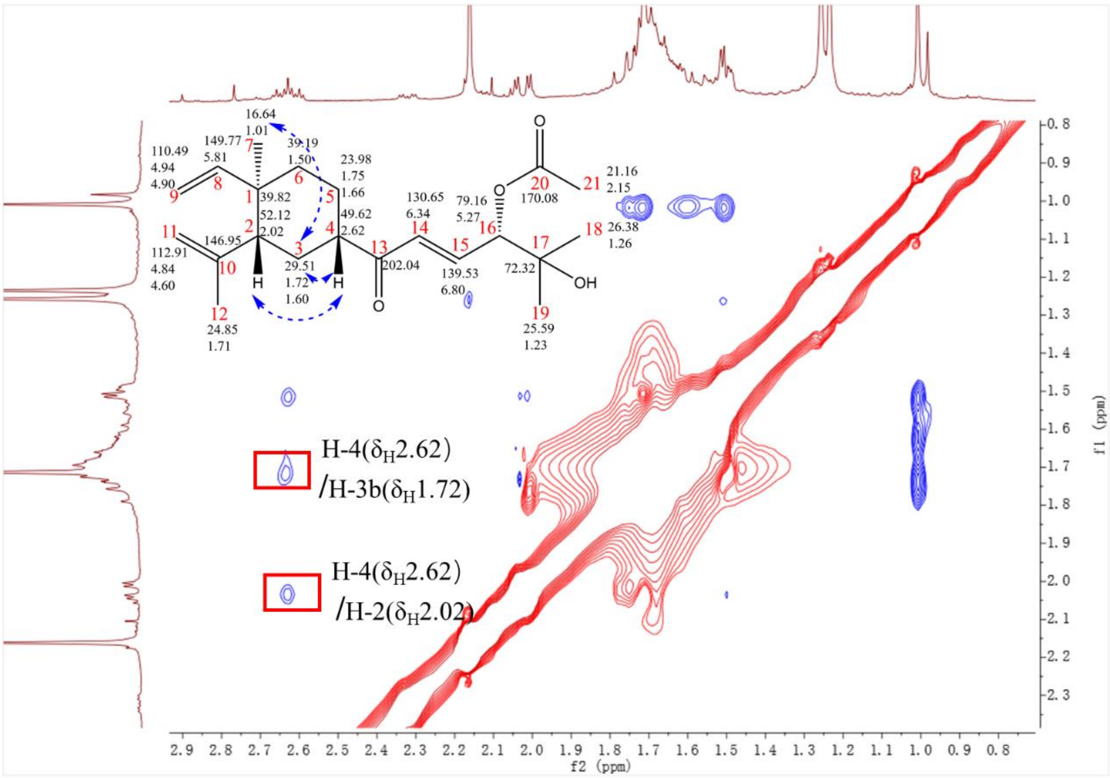

Figure S102. NOESY (600 MHz, CDCl3) of compound **7**

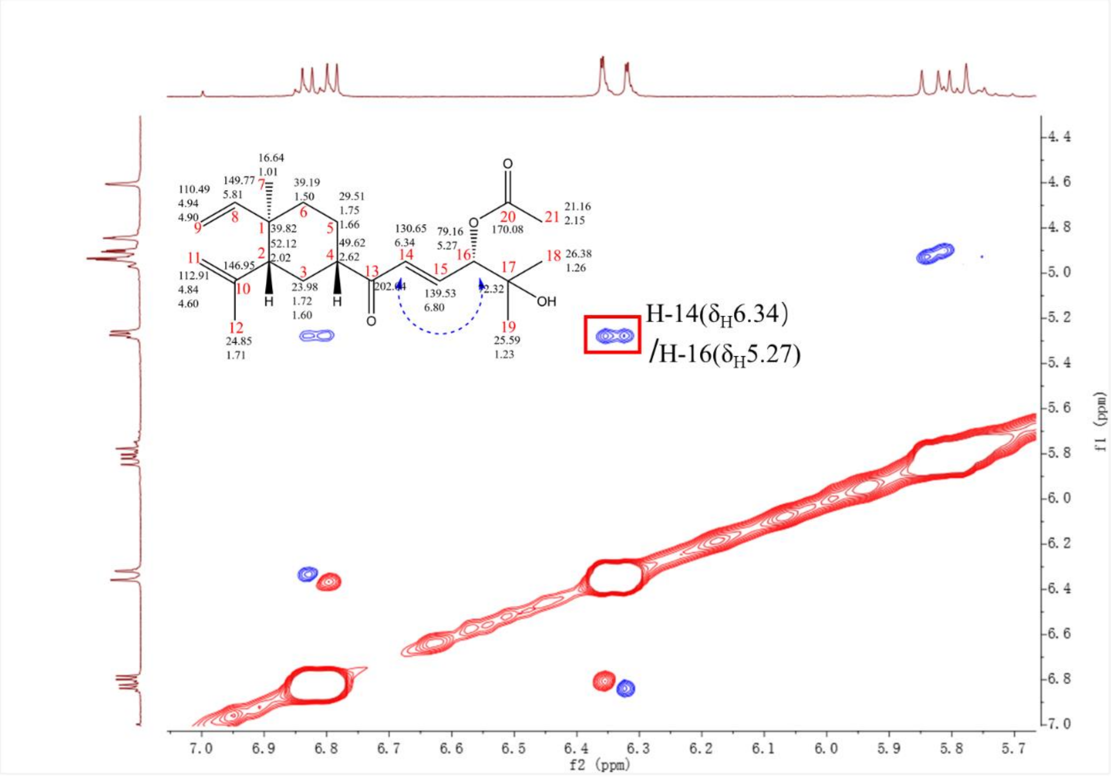

Figure S103. NOESY (600 MHz, CDCl3) of compound **7**
